# Supplementary material for: Why do you choose this program?—A decision-making model of medical students based on grounded theory
Source: PLoS One. 2023 Sep 15;18(9):e0291634. doi: 10.1371/journal.pone.0291634 (PMC10503722; doi:10.1371/journal.pone.0291634)
Supplement: S1 File — (ZIP) [file pone.0291634.s001.zip › RAW DATA/P1 CHINESE.docx]

05月11日_2.wav

00:01

好，可以开始了，你好，我们两位是医学教育研究所的研究人员。然后我在谈话之前要跟你讲一下，就是实验道德伦理须知，本次访谈中受访者是在平等自愿的原则上参与的受访者必须要真实的表达自我的想法和认知，并且确认自己符合社保的条件。在整个访谈的过程中会被录音，但是所有的录音资料将以匿名的形式用于科研，不会泄露给任何的第三方。在访谈的过程中，假如你有觉得不舒服的问题，你可以直接跳过，在访谈结束以后，你也有权联系我们取消整个访谈录音资料的使用权，啊你是否知晓并同意？

00:44

可以。在访谈之前我想先问一下您现在是什么年级什么专业的？我是17级基础医学工程是在国中班里面，对吧？更基础方向。好的，在访谈的开始之前，我先讲一下我们整个访谈的一个目的。首先我们整个目的并不是说呃要访问出什么问题的答案，主要的目的呢是还原你从国中班的宣传，就是从你大一开始，从宣传到报名录取到你整个股东班学习的整个环节，就是我们想要通过访谈来把整个过程给真实的还原出来，所以从信息获取到思想转变，从你的情绪从事情或者说任何的方方面面的事情你都可以去聊，尽量去聊出你的想法和影响，你的每一件事情就是说，其实现在这个并不是一个很严肃的场合，简单的聊天，好吧？

01:49

下面我们从因为我们这是关于国中班的一个访谈，所以想问一下你大一的时候从，大一时候在报名国中班的时候，你是从什么渠道去了解他的？以及怎么了解的？当时是我大一的时候是护理学院，然后因为我大一的时候就开始在准备转专业，也在了解这方面。

02:14

然后当时我们这是第一届国中班，然后是由辅导员护理的辅导员，然后有一个国重招生的一个宣传，一个微信的推文，然后转到了班群里，然后我就看到然后我当时就了解了一下，当时其实在转专业的时候你有很多选择对吧？

02:36

不仅仅是国中班选择，其实你在啊我想问一下，当时你你选国中的时候，你还可以同时报名其他的转专业？对我还报名了儿科转专业。假如当时同时要考上儿科辅助，你当时会怎么选？因为当时其实我也是有一点了解的，然后我的成绩在我们学院是排前30%，然后儿科的当时一个转专业的情况是我感觉儿科竞争很激烈，然后国中当时是第一届招生，然后招生的人数也很多，然后这个专业我感觉还是不错的。

03:14

所以我当时儿科的考试我并没有怎么准备，只是当时虽然去参加了，是因为儿科他会考一个细胞生物学，然后国中转专业也是有一个细胞生物学，然后我想去看一下细胞生物学的卷子是什么样子，所以我就去参加了课的。

03:31

所以你本来儿科只是试一试，主要还是想去沟通，对。

03:39

你当时对股东方了解的多吗？就是报这个专业主要是在微信公众号看，然后这个也是和家长商量过，然后肯定转专业不是我自己一个人的事情，然后跟我爸家里人也是了解了一下，创业，然后也去在百度上然后看了一下南医大的生殖医学，然后是国家重点实验室，发现他的对学生的培养模式，当时说的是本硕，可能是连读，也有可能是本硕博连读。

04:10

然后我把家里然后跟我一致同意，觉得是可以一个很好的选择。

04:17

你刚刚提到一点，你说他是本硕博连读，然后你跟家长传递的信息是吧？没有传递一，本硕博连读，他因为这个东西我当时因为隔得有点远，当时反正我想一想然后当时是把推文给我爸看的，然后当时好像说的是研究生优先录取，然后当时是没有本硕连读这个说法，是研究生优先录取后来，后来也没有本书连读这个说法。

04:48

然后但是因为现在成绩还行，可以保研走国中这边保研，所以就直接跟我爸说是跟我说给你，然后基本上这种说法你在也就是说你父母其实都是通过你来了解的。

05:03

是的，本来这个就是我自己的事情啊给他们看，也只是征求一下他们的意见，然后他们也是希望我转专业，然后我自己对选择了他，我父母只是给一些参考的意见，希望你转专业是因为觉得本来护理学就业不是，当时因为我高考的分数不是很高，然后报了这个学校就是报以转专业的目的去先进护理。

05:32

你护理是第一志愿，对第一志愿我第一志愿就填了护理，为的是进这个学校，然后进这个学校再通过转专业转到别的专业去，一开始就已经存了，进来之前就已经存在了整个一个想法，对。

05:46

你当时在高中报名就是进学校，当时是想转到哪个专业有考虑过吗当时？

05:52

是当时也没有多想，是因为当时当时我爸是打电话给招生办的，问南医大转专业好不好转，然后招生办的说法是转专业好转，然后我爸说那就去整装修，他说护理读的虽然工作很好找，但是对男生来说还是可能家里人还是不太在大一过程当中，其实你本身还是比较想转，更想转一个5年制的对吧？5年制的专业。你喝我不用其实。

06:34

你是更想分享离开护理专业，从一开始就是这个样子，对，当时你在当时选择选择转专业的时候，包括你选择儿科和国重是基于一个什么样的考虑？是觉得他就业更好，还是说觉得我想知道的是你在选择专业的时候，更多的考虑的是以后的就业的方向更好，还是说有其他方面的一些考虑？

07:07

当时的第一个想法就是要转走，没有其他的先不想再护理咱要转走，然后还要因为我成绩也不差，所以就要转一个相对好的专业。嗯你，父母对你转专业也是很支持的，也是希望你专做，对。你当时在选活动的时候有跟同学之间交流过这个事情？

07:30

有交流过，因为他是在他们和你的想法。同学的话，我舍友有一个也是报名的过程，但是他没有考上。当时这个是整个学院都是应该是知道，因为班群里面都有通知，因为我们是第一届当时宣传的力度还是很大的，他专门给我们办了一个宣讲，就是护士给护理办了一个宣讲。护理和其他学院当时他有三个，我记得是三个批次，就是分别不同的三分了三个批次去宣讲，然后护理有一场。

08:04

所以你们当时其实报名的应该说热情都很高，你看你说的我记得我们第一届人数非常多，在后来的时候我还有个小问题想问一下，你的学弟学妹有有问过你关于活动班的事情我问过，18级问我的比较多，19级我19级就可能不太认识这个人。18级人寿的，然后我也介绍了很多学历学生来试一试考虑考虑，所以你还是鼓励他们进来对。

08:36

你们当初选择读中班最主要是什么管理？想离开护理，对一个首先是转专业，第二个感觉国中也是一个很不错的选择，我想问一下，或者说换一个问法，当时国中宣传的时候，有很多方面就是最吸引你的是哪一方面，我可以帮你回忆一下，因为我也看了他的，宣传的PPT，一个是以奖金，还有第二个是科研导师，还有一个特色的见识见实习，第4个是免疫优先录取，第5个是5+1+3的本硕博的培养模式。

09:14

第6个是出国深造，我总结了一下，基本上它和其他专业比的优势就是在这6点上面，你觉得哪一点是让你印象最深的，或者说是让你心动的一个？

09:26

一个是5+1+3，那个东西就是在硕博连读的，当时，因为我爸可能也是看到了，因为这有点闲了，可以随便聊是吧？然后因为我们老杜家到目前为止只有一个博士，所以我爸是非常希望我继续读书，然后他说在这个方面好像也可以读到博士的距离好像很大。

09:53

你自己本人对读博我很想读路径也是认同的事情。你觉得你想读博更重要的是你觉得你读博士是受父母的影响更大，还是受你自己的影响更大？以前是我父母的影响最大，现在感觉还是蓝图对我自己的优势，还是现在不管外面的就业或者什么就业压力还是很大，如果能赌博，确实对我以后的发展是很好的，就是说提高学历主要是为了自己就业所为以后的发展对，你本身对专业的兴趣。

10:35

其实大一的时候我对也不是很了解，因为当时他出现了宣传的时候是已经5月份了吧。我记不太清了，反正我那时候大大一已经要结束了。然后其实我也是那段时间，因为那段时间已经转专业的时候，大家都在准备了，然后所以就匆匆忙忙的了解了一下，然后当时说对科研有什么兴趣的话，凭良心说，我确实当时科研室有是什么样子的，都了解的不太清楚，对大一学生很难，对，因为当时基本上也没有因为转专业主要靠成绩说话，然后所以其实也没有多了解其他专业，当时想的就是自己成绩在哪个方面，在哪个等级，然后就往哪个专业自己能去的往那边去靠，都是根据自己成绩来定的。

11:29

根据自己的成绩往最好的一个路上去走，有可能的路上去走。所以从一开始坚定的要离开护理专业，然后家长也支持你去走科研的道路，你自己本人也是愿意去走道路对。吧是在这个过程中，当时报名的过程当中，没有印象比较深刻的事情，或者说坚定了你离开护理的决心，或者说整个大一过程都可以坚定了你离开护理就报国中的决心，或者说改变的决定，没有的话你就说没有。

12:13

这个经历从一开始就肯定要转到，一开始就很坚定，然后规划很明确，人生规划就是要转专业，反正你去活动也要去别的专业，对。也不是吧，差不多也是这个想法，如果自己成绩够不上活动的话，肯定也不会在活动这方面浪费时间。

12:32

既然自己有或者说我们换一个说法，可能假如说啊当时你的成绩不够，不够转到国中或者转到儿科的话，只能在4年之内转，你还会选择离开护理是吗？对，我应当。你读读中专业之后，有没有就觉得有心态上的这个是一个什么变化？心态我本来想一下想法其实感觉还是好的，感觉在国中待的也还是蛮顺心的，也没有说什么后悔。

13:08

看什么就整体还是符合你的预期。有没有一两件让你印象比较深刻的事情，可以是高兴的事情，可以是不高兴的？是栽在果种，从果种以后对果种的话，当时这种当然说当时因为我们第一次去实验室嘛，开始第一次去实验室，然后当时是什么都不懂，然后科研对陌生的环境下，然后我们又是第一届，也没有什么学长学姐给我们指导，然后导师也只能看看简介，说实话都不太清楚，然后就选了一个导师，当时是王晓明老师，也是国栋董事。

13:56

然后进去以后选择他随便选的吗？还是也当时因为是第一轮。

14:03

当时大二第一轮，然后我们又是第一届，基本上大家都是摸黑着走，也没有什么想法。直接差不多也是也不算随便选，也有基础的学长学姐，是认识王晓明老师，然后推荐我可以去他那边转一转。然后当时一个很陌生的环境，然后进去以后发现里面的老师、学生、师姐、师兄都特别的好，然后当时就感觉非常的开心。

14:34

你会和实验室也师兄师姐一起经常交流是吗？一般的话晚上白天没有课的话，也会去实验室，晚上一般当时都是在实验室，然后一起吃饭啊什么的。你和实验室的师兄师姐关系可能会更亲密一点，和同学比的话，这也倒没有可能。半斤八两，差不多。

14:59

对两边关系都挺好的，你去做实验室的项目的时候，我说大二就是科研轮转的时候，你是去做什么内容？当时和他们当时是刚进实验室，其实什么都不懂，然后师姐也没有让我们马上跟着做课题，你们在你说你经常去实验室去干什么，是做一些基础的实验，虽然没有做课题，但是一些零碎的实验你可以开始先学起来，像一些WiFi什么的，然后做职业这些都是很长期的一个步骤，然后你如果哪天有空了，然后像我们可能是学习压力也比较大，也不可能经常去，也不可能抽出时间连续去，往往一个大实验室做不完的，然后我们抽时间去，然后做到哪一步了，然后师姐就教我们，然后下次如果再做到这一步了，然后就让我们来做。

15:48

然后就先培养一下，这样做实验就是熟悉一下实验的步骤，对就给后面的科研更好，工作更好开展奠定一下基础。你当时去做这些像你说像课外时间，其实课外时间学生是可以自主选择的。当时是你自己主动说师姐我要去那还是说她师姐说你们可以过来学一学这些东西？Ooo事件是没有要求的。然后他是自己主动去的，对主动去的，然后我因为是转专业的原因，然后所以到了新的学院，我也没有去参加什么学生会什么的，因为当时已经大二了，感觉也没有必要，然后大一的时候因为要转专业，然后校会什么的那些也都退掉了。

16:33

所以大一你是参加了学生活动的，大一参加了很多，然后等到大二的时候，感觉该参加的想参加的也都参加了，我就没必要了。

16:43

然后专门从大二的时候进入国中班以后，你就退掉了这些学生活动。对也没有全退的话，比如自己打球什么的，还是个人兴趣这方面的。社团还是在对，但是像学生会这种这种职务上现在这种工作就都推掉了，对。

17:02

为什么是觉得它太长时间没意思，是指它没有带来你想要的东西，还是说里边的人很好，里边的学长学习对我都很好，但是里面的工作做起来感觉意思没有含金量，我可以这么说吗？算是吧，因为简单的事务性的工作，这种工作是谁都可以做的对。

17:30

所以你会感觉可能成就感可能没有那么高，我可以这样说是可以你的如果中国以来就是最大给你最大的感受是什么？感受的话，我感觉当时没有选择然后我，也当时国中是支持你，如果在国中待的不满意的话，是可以自己退出的。有想过退出吗？这个是我没有想过的，因为有的人确实退出了，但是我是没有退出，而且准备如果没有意外的话，应该会在这边读一读。

18:14

继续在他在老师里面对，你们现在应该是还在确定保研的名单还没确定出来是吧？还是已经，因为这学期还有课，结束了以后才能确定保险名单。但是假如说你就是确定了保研了，你会会在还在做现在做的方向是吗？应该是没有意外的话，你可能是你从你当时大二的时候选了一个导师，到现在都没有换过吗？

18:42

没有，后来我换了老师，虽然因为他那边在升值方向做的不是很多，王晓明老师是免疫学系，它在生殖方面做的不是很多，它主要偏免疫，然后我还是比较，因为我来国中班，所以我还是想去一个甚至做了更好的一个东西。

19:03

所以当时在换导师的时候，你的想法是什么呢，是因为你我我是因为你觉得我想做生殖方面的研究，就是说免疫可能给我带来的免疫和我的兴趣点不是很重合，还是说有什么其他的原因？虽然第一个实验室非常开心，因为我们轮转本来就是要选不同的导师，他就是让我们通过轮船，然后来选择一个自己的心理方向，对选择一个适合自己的导师嗯，然后最后也选到这个实验室。

19:34

现在我是去了4个导师和别人进行论证，最后选择了他，我最后去选择了我现在待在实验室是王强老师，你可以简要的说一说你现在做的科研的一个内容，就是大概的说一下，因为现在我是好好我身体好，于处长在在革命学校里面因为我现在是在王强老师那边，然后也跟着师兄做他的课题，然后因为是敲出手方向，然后导师给我。

20:15

对我怎么讲，我以后如果在他那边读研的话，可能也是做销售这个东西。

20:21

然后具体的内容的话就是做一些实验，就是智力够智力或者去做阿斯达这些师兄会因为我还是有很多这学期开始我才真正的写下来时间更多了，以前特别是像上学期整天的课，就是星期一到星期五全满课，然后压力还是蛮大的。

20:46

所以在实验室的时间其实不是很多，然后师兄也是根据我的时间，然后给我分配一下时间。刚说前一段从上个学期到之前，都是马克给你带来很多压力。大三的时候，课也还稍微好一些是吧？但是大三的时候我刚到这个实验室，然后也还没有跟课题，然后在自己了解。然后大三下学期因为疫情的原因回到学校，已经快四五月份了，其实那个学期已经快结束了，其实我对差不多。

21:18

你说上学期课很多，给你带来很多压力来源于是是哪方面的呢？学业压力我觉得作为医学生都会面对的是是考试太多了，还是说你没有时间去实验室，没有实验去实验室的时候就给你分布？这倒没有师兄对我的态度，有时间就去，他是根据我自己的想法，他也没有强制要求，还是期末比结业考试的压力比较多？

21:53

你觉得这些压力会影响到你学习生活吗？或者说科研生活。

22:00

科研生活肯定是会影响的，因为上学期的话课实在太多了，然后因为是内外妇传染病流行病，然后还有什么临床进展，然后我自己还要重修重修了三四门。重修是为了保研是吗？对更稳定的保研。所以上上学期如果单纯的只是上那学期的课的话，可能压力也不是那么大，然后因为我又重修了好多年，所以上学期会比较累。然后上学期其实我到后半段师兄也让我专门准备考试，也没有让我去实验室。

22:41

说到课我就看到了，我就想到之前看那个PPT他其实其实是比基础医学要多开了很多门课，像生殖生物学、发育生物学这些企业论坛科研实践，你觉得这些课程有给你带来什么收获吗？或者说？这个课多开倒是没有，因为他也把基础的课删掉了，就行。所以两边上的课其实是对的，也差不多。然后有这些课我也觉得是必须要上的，因为我是读生殖的像这些、生殖生物学、生殖发育学这些东西是绕不开的，肯定要学的，然后科研轮转的话，它是一个去实验室学习的过程，没有专门的上课，然后所以他本来就是要去实验室，所以其实多开的课对我来说没有什么压力，什么，我觉得都是应该的。

23:34

你觉得还是有必要的，毕竟是给你后面的科研来说也是一个知识的准备。

23:46

刚刚你说到了王强老师就是做消除，你觉得他和你的兴趣点是重合的吗？或者说你在或者说你在报股东之前，你刚说了你父亲也觉得家里要出一个博士比较有面子，你也认可就是走博士这条道路，你在选择这条道路的时候肯定知道不是毕竟是做科研，你有想过自己要做什么方向之类的吗？

24:14

他就说看现实情况能选哪些就就哪些，因为我觉得在本科或研究生，其实说自己选方向其实也不是很现实，因为我们也没有这个水平和能力，然后在研究生之前这个阶段有导师给你安排，然后根据导师的思路，先把一个实验正常的走走完一个课题，正常的走完，了解一下这里边的什么路数嗯，然后你这样你才能在博士阶段或者才能走得更好，或者你就在读研这个期间发现自己好像不太合适，研究生读完你再去想做从事别的，我觉得也是可以理解的，这以后的路我也不可能现在就完全行了，对，所以还是先熟悉一下科研的套路，然后后面再在到出国的时候才去选自己真正感性或者说适合感性和感兴趣的方向。

25:22

你在选择方向方面，我觉得主要是受老师的影响，还是说学长的这个影响？你不是刚说了4个老师，你有跟同学讨论过，跟辅导员或者跟家里讨论过？

25:36

跟家里是没事，在网上，因为我爸妈确实不太了解这些，然后跟同学的话讨论还是蛮多的。因为国中我室友都是国中，然后也会互相讨论一下老师，然后轮转，然后差不多我就想不起来这个事情他们有影响到你吗？还是说你跟他讨论完觉得我的想法还是没有变？他们没有影响到，然后因为这个导师也是我自己选的，他们也没有来过导师这边，其实我们去的一般都不会在同一个导师。

26:07

所以你们4好像也没有4个，就是你和你舍友转的导师都不一样，有时候会偶尔重合，但最后定下来的都不是一样。我发现你主要还是做自己做决定。一般也不会太受就是外界对你的影响好像比较低，可以听一听，但是最后肯定我自己的事情，不管是后悔还是什么的，肯定都是我自己决定以后也不会抱怨别人。

26:39

的时候你从往小明老师转到王强老师那边去，有和前一个王晓明老师讨论过吗？或者说跟王强老师表达过，你要去他课题组的意愿，就是有跟老师交流过，那也是王晓明老师推荐我去王晓明老师那边，因为我当时他为什么要推荐你去，因为我说我想要更多的做生殖方面的东西，然后他说他实验室可能本科毕业可以，但是你如果研究生读他那边的话可能会不是很好。然后他就给我推荐了王强老师，然后王强老师在活动也是非常强的一个老师。

27:19

你在小明老师给你推荐了王强老师，你有去从别的方面去了解他吗？在去之前我有在果中能够导师介绍里面，我看一下他的介绍，然后之前有学生在王强老师那边轮转过，我也去问了问，然后你问了他什么，问了实验室的一个氛围，老师对学生的态度，然后师兄师姐这些，所以其实你对你整个科研工作的氛围，还有这些整个人和人之间的和谐程度也是蛮看重的是吗？

28:00

对，因为我觉得在实验室里面，如果我跟别的人处的不开心的话，很影响我的心情。

28:08

之前有发生过类似的事情影响到你的选择的？没有，但是我听过。不同的，不管是国中还是别的实验室啊，还是别的学校的实验室，是从同学听的吗？还是从老师上网上什么各种同学都会各种渠道其实都会。因为你在这一行，然后慢慢就会接触到一些然后，网上也是一个你比较重要的获取信息的来源是吗？你会比较常上吗？去查这些信息也不是查就忘了，看到了就顺便点开看看。

28:43

是帮我们，比如说知乎之类的网站，也不算微博什么乱七八糟的东西，也都有时候会刷到，然后就顺便看看，从微博获取跟学术有关系不是，说明他关注的是就是学术的学术的学术是学术，对实际上是就是圈子实验室乱七八糟的杂事，都会讨论，一一般情况下社交媒体就是你关注啥的给你推啥，我懂了，对就是大数据运算。

29:17

所以他可能关注的这种人比较多，就会给他推荐这种消息或者这种故事。

29:25

然后就看到了高兴吗？可信度高吗？你就当一个杂事了解，当一个有时候也蛮搞笑的这东西，整个国中班读下来有没有我是不是问过这个问题，有没有让你高兴或者不高兴，印象最深的一件事情可以讲一件给我们听一下。就讲印象最深的事情。好像刚刚我就是印象最深的。印象最深。没什么印象。因为印象最深的当时第一次去刚刚我好像讲过去实验室，因为是第一次，然后陌生环境下那个印象是真的非常的深，一直到现在我都很接受，因为他比你的触动，对，因为我感觉在国中这边好像还是很不错的。

30:21

因为第一次接触的事情，然后给自己的感觉不错，是因为你觉得这个实验室很高大上吗？不是高大上，我比较在一种人与人相处之间的氛围，你觉得人与人之间的相处我觉得是非常重要的。然后对你觉得做什么事情会给你带来成就感？成功感的话，一个是你你在整个过程当中有有感觉到成就感这个感觉吗？我每次把实验做成功，我都感觉很有成就感，我成就感这种大的小的很多，然后在实验室跟师兄师姐处的都非常好，这些都给我成就感，所以和人和人之间交往比较成功，你也会感觉到非常的开心对。

31:12

在当时现在目前为止，回顾您整个学习的过过程和当时报名的意愿，你还是你还是觉得他达到了你的预期对吧？所以我就想仔细的再再问一下刚刚问过的一个问题，像比如说你的一当时18级啊18级学弟学妹来问你，你是说你会推荐他们去报18级是指护理的学弟学妹吧还是基础的？有别的专业来问我，我也会推荐。

32:03

但是你给他们推荐的理由是什么？然后当时你觉得你我我还想请问一下当时这些学弟学妹来问你，他们都问你国中办的什么事情呢？做什么的它的内容是什么？对因为说实话他们就跟大一的时候我一样，不知道什么活动到底要干些什么，他们也很多都是盲目的想要转专业，他做什么这个问题真的很大，对我也很难回答，我这个问题很难回答。

32:34

但是我说你如果我说你如果不清楚国中的话，他有两次转专业，你先可以转进来行，不是，他是股东是在后面，我说你要把要不你先把你清楚的专业你先试试。然后反正我给他们的一律回答就是做科研。如果你科研你还是不懂，你就去网上自己去查查，看什么是可以先试试，是指先转像你之前报儿科。

33:03

对，他们是在之前的国中出台之后，国中只有国中在最后，其他的所有专业都在前面。国重有没有可能会出现，我可能找人会转成功了，转股东也转成功了这种情况。

33:16

前面专业全部流程结束以后才会开始果冻班的报名，是这样吗？到目前为止我没有听说谁先转了别的专业又来转过来，只有两种情况，一个是转到了基础，然后再从基础考核中或者转到预防，再从预防考核上，因为这两个专业本来就是基础的两个方向，但其实转专业比如说从护理转到继续医学，再从继续医学转到国中，就没有必要，对，所以还是很少有这种情况出现，偶尔有个别所以其实大部分的学生就是说还就是说我想两条路都走试试看，我也想转向临床医学试试看，能不能转上去，就对于那些成绩达标的学生对，当然要是考不上的话再试试过重。

34:06

来医科大学我觉得不想读临床的应该是没有的。所以说国重还是说算是自己的一个呃？

34:18

选择之一就是说可能比自己原来专业会更好的话，还是会选择来的。比如说可能4年制的专业，因为5年制专业，我记得分数线是应该比4年制要稍微高一点的，应该是高很多高20分左右学弟。

34:42

学妹除了问你干什么，还会问什么？该怎么复习。复习是指转专业考试学习是吧？考细胞生物学和组织皮带学，问的问题都好大，这两本书还是蛮好，回答的。可以说然后问这个专业是干什么，会问你这个专业以后干什么吗？也不会问你，因为你也是当时大二。对，我当时了解的。还算了解，但是他们没有问这个问题，估计他们也没有会了解他以后的出路嘛嗯，我大二的时候就了解的差不多，走科研这条道路，你是坚定不移的要走出科研这条道路，以后，比如说保研成功了以后，你也是想继续走股市道路，走5+1+3路线能走，能走的话肯定是往那个方向走，对，不能走那就再说。

35:39

肯定先读研，读完研走不了博。那个套路的话，也可以在转播研二的时候，研三的时候也会有本来就会有一个这样的转播机制，我也会争取。我说假如转博的时候，你突然发现可能升值那一块名额没有了，肯定要转到别的方向。

36:09

比如说啊免疫的，或者说其他的基础医学的方向你能接受吗？当时假如说出现了这种情况，做不了升值，但是你还是可以读博的，但是可能需要换一个方向，就是你研究生之前做的像这种情况出现的概率基本上很少，基本不可能。但是说的话我可能会考虑一下去外销，或者因为在从研究生在重新换一个方向到博士，其实就是一个就是研二的时候，研二的时候，你如果到博士再换方向，其实对自己以前的一些工作一些学习都会影响太大了，还不如想办法再考别的学校。

36:56

所以你还是比较应该说比较在意或者重视之前做，包括到现在为止积累下来的科研的经验方法也好，包括资源也好，都是还是蛮重视的，不想去放弃它。其他的我觉得我问的差不多了，你没有什么问题，基本上反正如果大一的时候再给你一次机会你还是会。行，我就肯定选，因为我大一的时候，对，差不多算是我最好的选择。不谈诚信。成熟好奇了，如果不谈成绩什么专业都可以去，就想去哪个专业临床，临床医学。

38:12

学医是你自己选的，就是选这个方向是你自己选的，我当时高中的时候高二的时候就准备学医来着，然后一直也想当时高二定的目标就是南京银行，但是高考不太如人意吧，因为当时我选一是因为什么是是觉得是收入比较高，地位比较高，还是说感兴趣还是什么？

38:43

我觉得都不一样，当时发生了一件事情，是我爸得了一个皮肤病，然后当时他看了很久都没看，然后突然遇到了一个医生，然后一两个星期就给他治好。

38:58

那时候我就感觉我爸非常的开心，然后说实话医生感觉对我爸的态度不是很好。然后我爸还就是往他那边靠，说好不容易认识到一个好医生，一定要好好跟他打交道什么的，然后感觉老师给你们成为一个女生就好。当时是高二时发生了这件事情，我记忆还蛮深的。

39:22

所以我当时高二的时候正好也让我们开始要定考什么大学，需要选什么专业，然后让我们老师说让我们可以先定好，所以这件事情让你促成你有这个想法，对你当时在报志愿的时候，父母有在影响你的志愿吗？你有或者没有影响，然后我说我学医，然后我爸爸就是他把我的8个志愿全都报了医学院对。你当时了解像专业这些信息，主要就是通过网站对当时了解的其实不是很多。

39:55

然后因为我高考的成绩说实话也没有太多的选择，能进医学院进去，也没有太多的选择。然后当时都是说要先进，然后当时打电话的话是南亚的招生办说，听他的说法是南医大最好转转，所以就第一个志愿填的那一大嗯那，你如果你自己的话，比较希望能够通过什么渠道来获得跟专业学校有关的信息？

40:28

这个我除了说实话在高中那个阶段，我除了在百度上面看一看，公众号上面看一看，其实我也没有别的什么途径，我感觉我可以多了解这个学校，因为我们家好像也没有，我周围的人也没有什么学，好像学医的很少，基本上没有。你当时报的时候和爸妈通知了一下，然后也是自己好像也是自己上网搜信息。是你是指国中吗？对报国中的时候，对，当时是把推广给我爸看了一下，其实主要还是你自己做的一个决定对。其他的。Ok没有什么没有什么问题，好的，感谢没事谢谢你。

41:22

那么这里我们这边附属老年医院就是我们一个老师，他建设的教师资格，然后要盖。
