# Supplementary material for: Why do you choose this program?—A decision-making model of medical students based on grounded theory
Source: PLoS One. 2023 Sep 15;18(9):e0291634. doi: 10.1371/journal.pone.0291634 (PMC10503722; doi:10.1371/journal.pone.0291634)
Supplement: S1 File — (ZIP) [file pone.0291634.s001.zip › RAW DATA/P1.docx]

Male, grade 17, nursing transfer

00:01

Well, okay. Hello, the two of us are researchers at the Institute of Medical Education. Then I would like to talk to you before I talk, which is the instructions of experimental ethics and ethics. In this interview, the principle of equal and voluntary respondents must truly express their own ideas and cognition, and confirm that they meet the conditions of social security. It will be recorded throughout the interview, but all the recorded data will be used anonymously for scientific research and will not be leaked to any third party. During the interview, if you feel uncomfortable, you can just skip it over. After the interview, you also have the right to contact us to cancel the right to use the whole interview recording materials. Do you know and agree?

00:44

can. Before the interview, I would like to ask you what grade and major are you in now? I'm a grade 17 basic medical engineering that is in the national middle class, right? More basic direction. Ok, before the interview begins, I'll talk about one purpose of our whole interview. First our whole purpose is not to access the answer to the question, the main purpose is to restore you from the class propaganda, is from your freshman, from publicity to enroll to your whole shareholders class learning whole link, is we want to interview the whole process to the real reduction, so from information to thought change, from your emotions from things or any aspects of things you can talk, try to talk about your ideas and influence, every thing you is, now this is not a very serious occasion, simple chat, ok?

01:49

Now, because we have an interview about the national middle class, so I would like to ask you from the freshman year, when you registered for the national middle class, what channel did you go to know him? And how to understand it? It was my freshman year, a nursing school, and then I was preparing to change my major because I was a freshman year.

02:14

Then it was the first national middle class, then the counselor to nurse, and then there was a publicity of national enrollment, a WeChat tweet, and then transferred to the class group, and then I saw and then I learned, actually you had a lot of choices when you switched majors, right?

02:36

Not only is the country middle class choice, in fact, you are in ah I want to ask, when you you choose the country, you can also sign up for other change of major at the same time? I also signed up for a new major in pediatrics. If you had to be pediatrics at the same time, what would you choose? Because at that time, I also had a little understanding, and then my grades were in the top 30% in our college, and then pediatrics changed my major is that I felt the competition in pediatrics was very fierce, and then the first enrollment, and then the number of students was also very large, and then I felt good in this major.

03:14

So I did not prepare for the pediatrics exam at that time, but although I went to attend it, because he would take a cell biology exam, and then the national transfer major also had a cell biology. Then I wanted to see what the cell biology paper looked like, so I went to the class.

03:31

So you originally just try the pediatrics, mainly still want to communicate, right.

03:39

Did you know so much about the shareholders? Is to report this major is mainly in WeChat public number, and then this is and parents discussed, and then certainly turn professional is not myself, and then with my dad family is to understand, entrepreneurship, and then go to baidu and then look at the south of reproductive medicine, and then the state key laboratory, found his training mode for students, then said the master, may be read, also may be read.

04:10

Then I took my home and agreed with me, thinking it was a good choice.

04:17

You just mentioned a point, you said he was a master and doctoral student, and then you communicated to your parents, right? No pass, read, he because of this thing I was because a little far, anyway, I thought and then was the tweet to my dad, then said is a graduate student priority, and then is not read the statement, is a graduate student priority admission later, then there is no book to read the statement.

04:48

Then, but because now the grades are ok, you can protect the country to protect the postgraduate, so I directly told my father to tell me to you, and then basically you say that is, your parents are actually all through you to understand.

05:03

Yes, originally this is my own thing to show them, also just for their opinions, and then they also hope me to turn professional, and then I chose him, my parents just give some reference, hope you turn professional because think nursing employment is not, because I was the college entrance examination score is not very high, and then reported the school is with the purpose of the professional to advanced nursing.

05:32

Your nursing is the first choice, to the first choice I have the first choice to fill in the nursing, in order to enter this school, and then into this school through the transfer of the major to another major, at the beginning has been saved, come in before there has been a whole idea, right.

05:46

You signed up in high school because you entered school. Did you ever have considered which major you wanted to transfer to?

05:52

Is also didn't think much, because when my dad was calling admissions office, asked south big professional good, then admissions office is professional better, and then my dad said that would go to the whole decoration, he said the nursing reading work is very good to find, but for boys or may the family is not quite in the freshman process, actually you still want to turn, more want to turn a five-year system, right?5 Year Professional. You don't have to drink it.

06:34

You want to share more about leaving nursing, which was like this from the very beginning. Yes, when you chose to change your major, including what did you consider in choosing pediatrics and national weight? Do you think he has a better employment, or do I think what I want to know is that when you choose a major, you think more about the future employment direction is better, or do you have some other considerations?

07:07

At that time, the first idea was to transfer, no other first do not want to care for us to transfer, and then because my grades are not bad, so I have to transfer a relatively good major. Well, you, your parents are also very supportive of your professional transition, and they hope you will specialize, right. Did you communicate with your classmates during the selection event?

07:30

Have communicated because he was in them and your thoughts. If my classmate said, my roommate had a registration process, but he did not pass the exam. At that time, this is the whole college should be known, because the class group has a notice, because we were the first time of the propaganda is still very strong, he specially did a lecture for us, that is, the nurse to the nursing did a lecture. Nursing and he had three other schools, and I remember three batches, just three different batches to preach, and then there was one nursing.

08:04

So you were actually sign up should say enthusiasm is very high, you see you said I remember our first number is very much, in the later when I have a little question to ask, your junior have asked you about activity class I asked, 18 level asked me more, level 19 I level 19 may not know this person. Grade 18 life insurance, and then I also introduced a lot of academic students to try it out, so you still encourage them to come in, right.

08:36

What was the main management when you chose the middle class? Want to leave nursing, for a first is professional, the second feeling countries is also a very good choice, I want to ask, or to ask, at that time, when the propaganda, there are many aspects is the most attract you, I can help you recall, because I also saw his, propaganda PPT, one is bonus, and the second is scientific research mentor, there is a characteristic insight see internship, fourth is immune priority, fifth is 5 + 1 + 3 training mode.

09:14

The sixth one is to go abroad for further study. I have concluded that basically its advantage compared with other majors is above these six points. Which point do you think impresses you the most, or the one that makes you move?

09:26

One is 5 + 1 + 3, that thing is read in the master and doctoral program, at that time, because my father may have seen, because it is a little idle, you can casually chat, right? Then because we only have one doctor so far, so my father is very eager for me to continue to study, and then he said that there seems to be a big distance to read the doctor in this respect.

09:53

You myself, I really want to read the path and agree with the blog. Do you think you want to be more important to your PhD? Do you think you have a PhD is more influenced by your parents or yourself?Before is my parents' biggest influence, now feel or blueprint for my own advantage, or now regardless outside employment or what employment pressure is very big, if can gambling, really for my future development is very good, that is to improve education mainly for their employment for future development, your own interest in professional.

10:35

In fact, I didn't know that much as a freshman, because it was May when he started doing publicity. I can't remember, I was over by that time. And then I was actually at that time, Because when I had already changed my major, Everyone was getting ready, And so I hurried to understand, And then he said any interest in scientific research, in all conscience, I did do what the research office was like, Not very clearly understood, It was very difficult for the freshman year, Yeah, because I basically didn't rely on my grades because I changed my major, And then I didn't know more about other majors, What I was thinking about was what aspect of my grades were in, At which level, Then to which major they can go to go over there, Are based on their own grades.

11:29

According to their own results to the best road to go, possibly on the road to go. So from the very beginning firmly to leave the nursing major, and then the parents also support you to take the road of scientific research, you yourself are willing to take the road right. In this process, in the registration process, there was no more impressive things, or strengthen your determination to leave the nursing, or the whole freshman process can strengthen your determination to serve the country, or the decision to change the country, if there are no words, you will say no.

12:13

This experience must be transferred from the beginning, very firm from the beginning, and then the planning is very clear, life planning is to change the major, anyway, you have to go to an other major, right. No, almost the same idea, if your grades aren't enough for the activity, you certainly won't waste your time in the activity.

12:32

Since oneself have or say we change a statement, perhaps if say ah at that time your achievement is not enough, not enough to transfer to the country or transfer to pediatrics, can only turn within 4 years, can you still choose to leave care? Yes, I should. After you read the major, do you feel that this is a change? I originally thought about the idea is actually feel good, feel in the country is still quite comfortable, also did not say what regret.

13:08

The whole thing is still in line with your expectations. Are there one or two things that impress you, can be happy things, can be unhappy things? Is planted in the fruit, from fruit after fruit, when this said, of course, because we went to the laboratory for the first time, began to go to the laboratory, and then what don't understand, then scientific research to the unfamiliar environment, then we are the first, no senior give us guidance, and then mentor can only see introduction, to tell the truth, is not very clear, then chose a teacher, xiao-ming wang was the teacher, also the building director.

13:56

Then go in and choose what he just wants? Or because it was the first round.

14:03

At that time, the first round of the sophomore year, and then we were the first round, basically everyone walked in the dark, and had no idea. Direct is almost also is not casually choose, also have the basic senior elder sister, is to know Wang Xiaoming teacher, and then recommend me to go to his side for a turn. Then at that time a very strange environment, and then went in to find that the teachers, students, senior sisters, senior brothers are very good, and then feel very happy.

14:34

Do you often communicate with the lab, do you? Generally, if there is no class during the day at night, I will go to the laboratory. At night, I are usually in the laboratory and then eating together for dinner. You may be closer to your classmates in the lab, and it's impossible to compare yourself with your classmates. Half a catty, almost.

14:59

It's good for both sides. When you went to do a lab project, I said my sophomore year was a research rotation. What were you doing? They were just in the lab, I don't know anything, Then I didn't let us follow the project immediately, What do you do when you say you often go to the lab, To do some basic experiments, Although no subject work was done, But some fragmentary experiments you can start learning first, Like some WiFi or something, And then doing a career is a long-term step, And then if you are free someday, Then like we may be more stressed to study, It is also impossible to go very often, It is also impossible to take time to go, Often a big laboratory can not finish, Then we took the time to go, And then, what step do you take, And then I taught us, And then the next time if you do that step again, Then let's do it.

15:48

Then the first training, so that the experiment is familiar with the steps of the experiment, to the following scientific research better, better work to lay the foundation. You were doing this like you said like extracurricular time, but students can choose extracurricular time. Did you voluntarily say that I want to go or that you could come and learn these things? The Ooo incident is not requested. Then he is his initiative to go, to take the initiative to go, and then I because is professional, and then so to the new college, I didn't go to attend what students, because was sophomore, feel not necessary, and then freshman because to major, and then the school will be what those are back.

16:33

So freshman you participated in student activities, freshman attended a lot, and then wait until sophomore, feel that you want to participate, I do not need.

16:43

Then, after you entered the national middle class from your sophomore year, you quit these student activities. If you don't all quit, such as what you play, or personal interest. The club is still right, but the job is pushed off, right.

17:02

Why do you think it is too long and boring, does it mean that it does not bring what you want, or say that the people inside are very good, the seniors inside the study are very good for me, but the work inside feels no gold content, can I say so? That is, because of simple transactional work, who can do it right.

17:30

So you may feel that the sense of achievement may not be so high, I can say that it can be you if China is the biggest to give you what is the biggest feeling? If I feel about it, I feel that I had no choice at that time, and I also supported you in the country. If you are not satisfied with your staying in the country, I can quit by myself. Have you ever thought about quitting? This is something I didn't think about, because some people did quit, but I didn't quit, and I am prepared to read it here if there was no accident.

18:14

Continue to be in the teacher, you should still determine the list of graduate protection has not been confirmed, right? Still already, because this semester still has the class, after the end can determine the insurance list. But if you were sure of the research program, you would still be doing the current direction, right? If there was no surprise, did you probably have chosen a mentor since your sophomore year, and you haven't changed it yet?

18:42

No, later I changed the teacher, although because he did not do a lot in the appreciation direction, Teacher Wang Xiaoming is the Department of immunology, it does not do a lot in reproduction, it is mainly immune, and then I still compare, because I came to the national middle class, so I still want to go to one or even do a better thing.

19:03

So when I was changing your mentor, what did you think, because you thought you and I were doing it because I wanted to do reproductive research, which means that immunity may bring me immunity that does not overlap with my interest, or are there any other reasons? Although the first laboratory was very happy, because we had to choose different mentors, he was just to let us through the ship, and then to choose a psychological direction, to choose a suitable mentor for them, and then finally to choose this laboratory.

19:34

Now I went to the four mentors and others to argue, finally chose him, I finally went to choose I am now stay in the laboratory is wang qiang teacher, you can briefly say you now do a content of scientific research, is probably say, because now I am good health, in the director in the revolutionary school because I am now in wang qiang teacher, then also follow the brother to do his subject, and then because is knock direction, and then the teacher to me.

20:15

How to tell me, if I attend graduate school in the future, I may also do sales of this thing.

20:21

Then the specific content is to do some experiments, is enough intelligence or to do Asda these brothers because I still have a lot of this semester began I really write down more time, especially like last semester all day class, is Monday to Friday full class, and then the pressure is quite big.

20:46

So my time in the laboratory was not very much, and then the senior brother allocated my time to me according to my time. Just said that from the last semester, Mark brought you a lot of pressure. In my junior year, the class was still a little better, right? But when I was a junior, I just came to this lab, and then I didn't follow the subject yet, and then I learned by myself. Then in the second semester of the junior year, I returned to school due to the epidemic. It was nearly April or May. In fact, that semester was coming to an end, but I was almost the same.

21:18

You said you were a lot of classes last semester. What put a lot of pressure on you? I think as a medical student, there face too many exams, or do you have no time to go to the laboratory and give you distribution when you go to the laboratory without experiments? This is no elder brother's attitude to me, have time to go, he is according to my own ideas, he did not mandatory, or the final than the final exam pressure is more?

21:53

Do you think these pressures will affect your study and life? Or rather, the scientific research and life.

22:00

Scientific research life will definitely affect it, because last semester, I had too many lessons, and then because of the internal and external female infectious disease epidemic, and then what is the clinical progress, and then I have to rebuild three or four doors myself. Reconstruction is to protect the research institute, is it? For a more stable research guarantee. So last semester, if only the class of that semester, the pressure may not be so big, and then because I took it for many years, so last semester will be more tired. Then last semester, I actually asked me to prepare for the exam in the second half of the year, but I did not ask me to go to the laboratory.

22:41

Speaking of the class, I saw, I thought that PPT before, he actually opened more courses than basic medicine, such as reproductive biology, developmental biology and other corporate forum research practice. Do you think these courses will bring you anything?or say? This class is not open, because he also deleted the basic class, ok. So the classes on both sides are actually right and about the same. Then have these classes I also think is a must be, because I read the reproductive like these, reproductive biology, reproductive development these things is unavoidable, must learn, and then scientific research rotation, it is a go to laboratory learning process, no special class, then so he is to go to the laboratory, so actually more class for me no pressure, what, I think all should be.

23:34

You think it is necessary, after all, it is also a knowledge preparation for your subsequent research.

23:46

Just now you said that Mr.Wang Qiang is to eliminate, do you think he and your interest points overlap? Or before you or you reported to the shareholder, you just said that your father also thought that a doctor at home is more dignified, and you also recognized that you should follow the same path of a doctor. When you choose this path, you must know that it is not doing scientific research after all, have you ever thought about what direction you want to do?

24:14

He said he could choose according on the reality, Because I feel that in an undergraduate or graduate degree, In fact, it is not very realistic to choose your own direction, Because we don't have that level or that ability, Then this stage before you before graduate school, Then, according to the mentor's thinking, First walk an experiment through a subject, Normal walk, Learn what the of on here, And then you can make you PhD or go better, Or if you find that you don't fit right during graduate school, After graduate school, if you want to do something else, I think it is understandable, I can't go completely right now, Yes, so get familiar with the routine of scientific research first, Then later when you go abroad to choose their real emotional or suitable for emotional and interested direction.

25:22

In terms of choosing the direction, I think it is mainly influenced by the teacher, or the seniors? Didn't you just say four teachers, you discussed with classmates, with counselors or at home?

25:36

With the home is ok, on the Internet, because my parents really do not know much about these, and then discuss with my classmates or quite much. Because my roommates are all Chinese students, and then I will discuss the teachers with each other, and then rotate, and then almost I can't remember this thing, do they affect you? Or do you discuss with him that I still haven't changed? They didn't affect it, and then because I chose the mentor by myself, they didn't come to the mentor, so we didn't usually go to the same mentor.

26:07

So you 4 also don't seem to have 4, is you and your roommate transfer mentor are different, sometimes occasionally overlap, but the final decision is not the same. I find out that you mainly make your own decisions. Generally, it is not too affected by the outside world on you, which seems to be relatively low, so you can listen to it, but in the end, I will affirm my own things, no matter regret or what, it is certainly I decide that I will not complain about others in the future.

26:39

When you went there from Mr.Xiao Ming to Mr.Wang Qiang, have you ever discussed it with the former teacher, Wang Xiaoming? Or expressed with wang qiang teacher, you want to go to his team, is to communicate with the teacher, it is also wang xiaoming teacher recommend me to wang xiaoming teacher, because I was why he recommend you, because I said I want to do more reproductive things, and then he said he laboratory may be undergraduate course graduation can, but if you graduate student read him there may not be very good. Then he recommended Mr.Wang Qiang to me, and then Mr.Wang Qiang was also a very strong teacher in the activity.

27:19

You recommended Mr.Wang Qiang to you in Teacher Xiaoming. Have you got to know him from other aspects? Before I have to mentor in the fruit to introduce, I look at his introduction, and then before the students in wang qiang teacher rotation, I also asked, then you ask him what, you ask an atmosphere of the laboratory, teacher attitude towards students, and then the elder sister, so actually you to you the atmosphere of the scientific research work, and the degree of harmony between the whole people and people is pretty value?

28:00

Yes, because I think in the lab, if I'm not happy with other people, it affects my mood.

28:08

Has something similar happened before that has affected your choice? No, but I have heard of it. Different, whether it is the country or other laboratory, or other school laboratory, is from the students? Or from the teacher on the Internet what all kinds of students will be all kinds of channels will actually be. Because you're in this business, and then you gradually get there, and then online is also an important source of information, right? Would you do more often? Go to check these information is not to check to forget, see the incidentally point open to see.

28:43

Is to help us, such as zhihu website, not weibo what messy things, also sometimes brush to, and then, by the way, from weibo access to do with academic is not, that his focus is the academic academic is academic, to actually is circle laboratory mess, will discuss, a general social media is what you push you, I understand, is the big data operation.

29:17

So he might pay more attention to this kind of people, so he will recommend such news or such stories to him.

29:25

And then I saw it, was I happy? High credibility? You when a chores to understand, when a sometimes also pretty funny this thing, the whole country middle class read down whether I have asked this question, whether to make you happy or not happy, the most impressive thing can tell us a thing to listen to. What's impressed me the most. It seems that it just impressed me the most. The deepest impression. No impression. Because the first time I went to the laboratory, I seemed to talk about the past, because it was the first time, and then under the strange environment, the impression is really very deep, until now I have accepted, because he is more touched than you, yes, because I feel like a very good side in the country.

30:21

Because of the first contact with things, and then give yourself feel good, because you think this laboratory is very high? Not lofty, I compare in an atmosphere between people get along with, you think people get along with, I think it is very important. And then what do you feel like doing gives you a sense of accomplishment? One sense of success is that do you have a sense of achievement in the whole process? Every time I succeed in the experiment, I feel a sense of achievement. My sense of achievement is very big and small, and then good in the laboratory, which all give me a sense of achievement, so you are more successful, and you will feel very happy.

31:12

At the time and so far now, reviewing your whole study process and your willingness to sign up, and you still think he has met your expectations, right? So I just want to carefully ask a question just asked, for example, your first grade 18 ah grade 18 junior to ask you, do you say that you will recommend them to the grade 18 is refers to the nursing junior or basic? Have a other professional to ask me, I will recommend.

32:03

But what is your reason for recommending them? Then at that time, you think you and I and I would like to ask me about these younger students to ask you, they all ask you what the country does? What is it doing? What is it doing? To tell you the truth, they are just like when I was a freshman, they don't know what activities to do, and many of them blindly want to change their majors. It is really a big question, and it is difficult to answer to me, which is difficult to answer.

32:34

But I say you if I say if you do not know the country, he has changed his major twice, you can first transfer in the line, no, he is a shareholder is in the back, I say you want to put whether you first you put your clear major you try first. And then all I answer to them is to do scientific research. If you have scientific research you still do not understand, you go to the Internet to check by yourself, see what can try first, is to turn like you before the report to pediatrics.

33:03

Yes, they are introduced in the previous countries after, only the country in the last, all the other majors in the front. Is it possible to appear, I may find someone to turn successfully, transfer shareholders also turn successfully this situation.

33:16

After the end of the whole process of the jelly class, is it? So far I haven't heard of anyone coming back to another major first, There are only two cases where, One is moving to the foundation, Then, from the basic assessment or from the transfer to prevention, And then, from the prevention assessment, Because these two majors are originally the two basic directions, But for example, from nursing to continuing medicine, Then, the transfer from continuing medicine to the country, There is no need for it, Yeah, so it's still very rare, Occasionally there are a few so most of the students mean that I want to try both roads, I also want to turn to clinical medicine to try it out, Can you turn it up? For those students who meet the grade right, Of course, if you don't, try too much.

34:06

I don't think I don't want to study to medical university. So is it a heavy country or one of their own, well?

34:18

One choice is that it may be better than their original major, or will choose. For example, the major of possible 4 years system, because 5 years system major, I remember the score line should be slightly higher than 4 years system, should be a lot of high high 20 points about younger brother.

34:42

What can you ask what you do? How to review. Review is to refer to the transfer of professional examination study, right? Test cell biology and tissue belt science, ask questions are very big, these two books are pretty good, answer. Can say then ask this major is what, will ask you this major later what to do? I won't ask you either, because you were also a sophomore. Yeah, I knew about it at that time. Still understand, but they didn't ask this question, estimates they don't know his future way well, my sophomore will know almost, take the road, scientific research, you are determined to walk out of scientific research, later, such as research after success, you also want to continue to take the stock market road, go 5 + 1 + 3 route can go, can go in that direction, right, can't go that say.

35:39

Surely read graduate school first, read graduate school can not go bo. If that routine, can also be in the broadcast research two, research three will also have such a broadcast mechanism, I will also strive for. I said if transfer bo, you suddenly found that possible appreciation that piece of quota did not have, must turn to other direction.

36:09

Like ah immune, or other basic medicine direction you can accept? If said at that time appeared this kind of situation, cannot do appreciation, but you still can read bo, but may need to change a direction, be the probability that you graduate student does like this kind of situation appears basically very few, basic impossible. But said I may consider to export, or because from the graduate student in change a direction to Dr, is a is research two, research two, if you go to Dr To change direction, actually for his work before some study will affect too big, as to think of a way to take an examination of other schools.

36:56

Therefore, you should still say that you care about or pay more attention to the previous work, including the accumulated scientific research experience and methods, including the resources, and you don't want to give up on it. The rest I think I'm asking something, you don't have any problems, basically if you gave you another chance as a freshman. Okay, I'm sure, because when I was a freshman, yes, it was almost my best choice. Don't talk about integrity. Mature and curious, if you do not talk about the results of what major can go to, you want to go to which professional clinical, clinical medicine.

38:12

Medical is your choice, is to choose this direction is your choice, when I was in high school school is ready to study medicine, and then also want to then high school goal is the bank of Nanjing, but the university entrance exam is not very satisfactory, because I choose one because what is think is higher income, status is higher, or say interested in or what?

38:43

I thought it was different. Somehappened when my father had a skin disease, which he watched for a long time, suddenly met a doctor and treated him in a week or two.

38:58

At that time, I felt very happy, and to be honest, the doctor didn't feel a very good attitude towards my dad. Then my father is still to his side, said very not easy to know a good doctor, must deal with him well, and then feel the teacher to you to become a girl. It happened when I was in my second year of high school, and I remember it pretty well.

39:22

So when I was in the second year of high school, I also asked us to decide what university to take, need to choose what major, and then let our teacher say that we could decide first, so this thing let you make you have this idea, when you were in the volunteer, did your parents affect your volunteer? You had or had no influence, and then I said I studied medicine, and then my dad was he who signed up with all eight of my volunteers to medical school. You know such information like professional at that time, mainly through the website at that time is not a lot.

39:55

Then because I don't have many choices in the college entrance examination, I didn't have many choices to enter the medical school. Then at that time is said to want to be advanced, and then at that time call is the admission office of South Asia said, listen to his view is the south medical university best turn, so the first volunteer to fill in that big well that, if you own words, more hope to be able to obtain information with professional schools?

40:28

This I in addition to tell the truth in the high school that stage, I in addition to have a look above baidu, the public number, in fact, I have no other way, I feel I can know more about the school, because our home seems to have no, the people around me also have no learning, as if learning medicine is very little, basically no. You told your parents, and then you seem to search for information online. Are you referring to the country? When serving the country, yes, it was to promote to my father, in fact, you mainly made a decision right.else. Ok nothing nothing no problem ok thank you all right thank you.

41:22

So here our affiliated geriatric hospital is one of our teachers, he built the teacher qualification, and then to cover.
