# Supplementary material for: Why do you choose this program?—A decision-making model of medical students based on grounded theory
Source: PLoS One. 2023 Sep 15;18(9):e0291634. doi: 10.1371/journal.pone.0291634 (PMC10503722; doi:10.1371/journal.pone.0291634)
Supplement: S1 File — (ZIP) [file pone.0291634.s001.zip › RAW DATA/P10 CHINESE.docx]

05月19日_1.wav

00:00

可以讲对你影响比较大的事情，也可以讲你的呃看法或者讲你的情绪，高兴不高兴，其实都可以跟我们讲。然后主要我们是想聊出主要是想聊出对你想法影响比较大的事情，或者说你印象比较深刻的这种事情或者情绪都可以。可以的，在时间到的在开始之前我们需要讲一下时间道德伦理须知。

00:24

本次访谈中受访者是在平等自愿的原则上参与的，释放者必须真实表达自我想法和认知，确认自己符合社保条件，访谈的过程会被录音，录音资料将以匿名的形式用于科研，不会泄露给任何第三方，在访谈过程中和结束后，你都有权取消研究人员录音资料的使用权，你是否知晓并同意？

00:46

可以的，然后请你先介绍一下你的专业和年级。我是基础医学院基础医学专业的17级学生带月，基础你在进入国中之前也是技术，我在进入国中之前是护理学的。数理学对。我们想先问的第一个问题就是我们想先知道一下你在报名前对股东办的了解有哪些，以及了解的途径是什么？报名前的是有一个宣讲，然后宣讲的时候我当时去了，因为我当时是大一的时候是护理，然后想转专业，然后一开始是想转临床的，后来就想着因为当时国中那边宣讲说这个算是第一次转专业没过，这算是二次转专业的机会。

01:36

然后我就这是他们宣传的时候就说的对宣传之后说你因为这个的考试是想在转专业考试之后，所以就是相当于你这个是一个二次转专业的机会，我一开始转临床的时候没有转过去，然后就选择了当时还你继续说选择了国中这边，因为我一开始两个是同时报名的，因为万事都会有不同的选择，而不就会有不同的结果，所以我就做了两手的准备。

02:04

然后两个同时准备的，你应该是学理科的对吧？对，我是学理科的。临床我想问一下在转专业的时候，你当时报的是临床医学。儿科是吧？报儿科是因为想做儿科，还是说因为其他的原因，因为当时想学临床的，我一开始是想学临床的，我当时报考男一，我当时报考南医大的时候，是因为当时凭我的分数线，当时我是天津人，当时在天津的招生分数线，按照以往的规律的话，我分数线是可以上临床的，但是因为我们那年南医在天津的招生分数线特别高，所以我就被调剂到了护理，然后就想着因为一开始就想着报临床，后来想在因为有这个机会，然后又抓了一把。

02:55

我想问一下你在高中时候报名的时候，你是理科生，你当时报了专业的时候是说一定要报医科吗？还是说要？理工科都想。我当时想的是全部报医学。全部报医学。是什么事情，或者说有什么其他的考虑吗？或者说兴趣或者说事情。其实当时为了学医学，也是因为我爷爷奶奶是因为心血管去世的，所以就是想学，医学，所以想为家里人做一点贡献，所以知道一定要去学医学。

03:34

对嗯。然后进了学校以后，其实从进学校那一刻开始，你就是一心要转临床，反正不想学护理，其实也不是不想学护理，可能因为各种各种原因觉得自己不适合护理。其实我当时大一的时候也在纠结过，我是不是一定要非要转临床，但是一年下来之后发现自己不真的不大适合护理。可以具体说一下，护理的各种什么，人家都是那种小姑娘比较新鲜，或者小姑娘就是小姑娘的那种性格什么，我觉得我不适合。

04:15

你说你觉得什么性格比较适合做护理啊，就是温文尔雅的那种。你觉得你是属于那种女汉子的性格，然后后来你在你觉得学临床或者是去基础做科学研究，都可以对，所以你两个都报名了，是的。你有从其他的渠道去了解过吗？还是只从去年会了解的？当时国中班就是宣讲会，还有微信公众号上。公众号是你们护理的公众号吗？护理我记不太清了，反正当时当时他们是学校的公众号，好像是南医大的，官微好像是。

05:06

你有跟你的同学或者说不仅仅是高大学，还有高中的，或者说你的家里面的人有去讨论过这个事情，当时选择转专业和然后讨论我好朋友好朋友讨论过。朋友是指高中的好朋友对高中朋友嗯。他们的意见对你有什么影响吗？他们就觉得我开心就好。所以也没有什么意见，对他们没有什么意见，你没有跟父母讨论过这个事，讨论过我爸妈就是属于那种我做的决定，只要是对的话，他们就会支持，只要我想做的决定一般没有特别违背什么伦理道德或者什么，他们都会支持。

05:46

主要是对的，就是就是就是。

05:49

比如说你高中时候报一定要学医学，他们也是无条件支持，他们当时是无条件支持，但是当时我那个成绩下来，各种都是茶店，他们也在考虑让我报一下，

06:22

当时他们也就是看到我那个分数有点然后我想报一下什么理工类的，但是最后还是最终协调一致之后还可以，有有他们有具体要求你报什么理工类吗？或者计算机还是什么东西的？他就说想单纯的想当，因为我是天津人，然后我爸妈就觉得我尽量是在京津冀地区多一些噢那种是不是，他们是从事理工类学科的工作。

07:26

不，我爸我爸爸是做土木的啊，所以他就觉得因为我是理科生，然后可以选择一些理工类的。

07:40

他们是觉得像京津冀那一块可能一个哥哥理工科主要是离家近，他们觉得离家近就是说你报什么学科其实无所谓，要离家近一点是这个意思，学科是我自己定的，他们不干涉，但是他们的首要决定是尽量离家近一点。

08:00

怎么所以当时你是不是来南医大也是微调，是不，我第一志愿我第一志愿是哪一个？

08:07

当时因为综合所有的成绩，我又不想去东北，因为我的分数线如果当时去沈阳或者去哈尔滨医科大的话，都是可以直接上临床的，但是我又不想去多买，所以就选择天津可能京津冀那一块的医科大学可能也我能上，但是上不了好专业。因为当时就想冲一下南医的临床。明白。所以在和家里讨论好像也没有对你的意见造成什么影响，没有。然后你后面进来了以后，你可以跟我们说一下，当时你是有没有什么吸引你的点，因为毕竟国用班它不是一个临床的专业，你为什么说一定要离开护理来到基础医学，我给您讲一下我当时转专业的心路历程，我当时是因为想要转临床，然后当时那个暑假应该就是复习的阶段，暑假我各种事情是暑假的时候我去参加了一个支教的社团活动，然后占去了，大班个月的时间，然后回家之后又突然生了一场病，然后又过去了一段时间，我觉得我的复习时间不大够，然后我那段时间整个人就特别早早看不下去书，然后在这之前我的成绩又有一点悬，因为是转临床的话要求是排名在前21%，开始我去找辅导员查成绩的时候，辅导员说我有点悬，然后我就在临床和基础这个地方徘徊过，因为最开始的时候还这得倒到我当年报志愿的时候，最开始因为报男一也是冲一下的感觉。

10:01

我朋友当时跟我说，他跟我因为他爸爸是医生，然后他就给我分析这些，然后他说你也可以实在不行你也可以上基础，因为基础医学的未来发展还是很好的，那个时候我就去了解了一下基础医学，然后最后虽然这边上岸的也是护理学，然后当时也在因为当时辅导员跟我说你的成绩有点不那啥，然后我就考虑在基础医学跟临床医学拍坏了一下，然后最后还是成绩出来之后，我在前20%，然后最后还是冲了一下临床。

10:40

然后后来因为我那段时间又看不下去，出人特别糟糟，我那个时候就觉得我自己上不了临床，我转不过去，然后我就因为我又是想转专业，我不想学护理学了，然后其实我当时转国中就是想转专业的那种，你能明白我的意思吧？

11:01

我明白。你说那个时候和你同学的爸爸去聊了一下，他们是做医学这个专业的，说基础医学发展也比较好，发展好是指哪方面将来的前景比较好，因为基础还是算是比较有前景的，只不过前景在前景好是指未来可能社会对专业的认可和需求。

11:29

需求还有认可，可能主要是对它的认可度比较高对。我刚刚有一个事情给打了个岔，忘了问了。你觉得学护理的人需要问问尔雅是什么事情，让你有这个感觉，还是说这个判断是我们主观的。

11:54

对，我主观觉得是这个判断，其实是从大一刚入学的时候，你就觉得是这个样子，是因为我所有的我周边我朋友，我的以前的老师，然后我朋友的爸妈或者什么，当时都问我你学什么？

12:10

我说我学护理，他说你这个性格还去学护理，他们我周围的人的一致意见全是你说周围人的一致意见是你觉得你自己想一下，你觉得是不是有一点点受社会影响，还是说在这之前其实你自己也是这么认为的？然后周围人的观点又加深了你的，印象就确认了你的主观判断，我觉得是这样讲是对的。

12:37

你说基础医学的未来发展前景比较好，比较受认可，可不可以说我只是自己的一个猜测，你也可以否认它可不可以说是可能做这个东西，它可以给人类社会带来一个什么东西，或者说它的社会地位比较高，所以你觉得它的前景比较好，你比较能接受它。嗯还是说你觉得自己也很难说，都可以。我对基础我就说一下我对基础医学的看法，我对因为临床的话，它从人你是从只是去治一个病，你只会能保证他这个病在表面上是看好的，而基础医学的话，你是可以去进一步慢一点的去挖它的根本。

13:34

我可能是理科生的想法，比较喜欢去挖他的根本，去找它的根本，我觉得基础医学可以让我去找这个根本，根本是你自己比较喜欢去探究一个事情的本质，还是你觉得像基础医学这种学科，可能它的影响力会比临床医学更大一点，你觉得哪个我比较喜欢想去挖它的根本，还是和你自己比较相关。但是在做东西的时候，你有没有考虑过他可能它的确是可以探究本源的一个东西，但是其实它也是很难的，你当时有考虑过问题吗，其实也考虑过，但是你现在随便说说没有什么事情就简单了，如果你想他不能你做都没做过，所以当时报名临转专业转临床，包括到国政班，其实可我可不可以说和学生父亲那次的交谈坚定了你的想法，就是让你更加说我想我觉得转到技术医学是可以的，还是说可能临床希望不大，所以我觉得我需要多考虑一下技术依据，觉得哪个成分会更重一点。

15:10

我觉得这两个成分都不一样都不太重，因为都是我主要的是随我，所以我行可能经过一段时间的沉淀后发现，因为我本来就是有一点虽然外表看起来我很就是活泼的那种，但是其实我内心还是有一点人群交流恐惧症的感觉，所以我觉得临床跟人打交道太多了，我可能有点冲不下来。在我们就现在先讲一讲就读了国中班以后，你可以我们给我讲一个影响比较深的事情。

15:54

你可以先说一下你是大几退出的吗？我是大四去年暑假的时候推出的学了大二和大三两年以后对，其实我大三的时候就想退出，然后因为一些原因就没有退出。疫情不是第二学期不是因为疫情，因为其他一些原因可以先跟我讲一讲，就是大二大三这段就是你在国中班的这段时间给你带来影响。

16:24

最开心的一件事情，最开心的一件事，没有的话可以不说，开心的事可能说董总概括一下。

16:35

以前从面上知道基础医学，后来是因为我护理没有接触过基础医学那个时候，然后大二的时候可能就是真正的去进入实验室去学习去真的去去接触专业了，从面到里面去了解一点东西，我觉得让我我说让我从无知变成有一点了解的感觉，我觉得这个让我挺那啥的。

17:12

其实我换一个说法来说，假如说你没有转专业的话去，去学护理学，护理学其实你也是可以是不断学习，然后不断深入去了解护理学上的一些知识的。

17:25

你觉得假如说你当时转国中转失败了，你这样的感觉在护理学专业一样也可以，护理学专业的学习也可以给你带来这种感受吗？也可能，因为也可能因为后面对不好说就可以不说。没有关系的。只要想说一下你自己的看法就可以。你可以你现在说一下国中班，当时让你发生不开心的事情，我可能觉得在国中班里它没有一个统一的标准，你说的标准是什么标准？

18:08

他给我们的考核要求是一致的，但是它对每个导师他让每个导师对我们的他是让每个导师去要求，每个不同的导师对学生的要求是不同，但是他最后的考核标准是一致的，所以这里面会有冲突，你就像我之前待过的实验室，老师会不让你去细胞，不让你去细胞房，但是我们最后的考核会要求你细胞的实验以及细胞的相关知识，然后你就会比较吃亏对就会很吃亏，然后还有也就是这种，因为不同导师对有些导师是放养，有些导师就会对你严格要求，你像有些实验室有钱，就咱们这样说，有些实验室有钱你招而且一些，东西无所谓，而另外有一些实验室就是比较普通的那种实验室，你去做实验做不出结果或者什么的话，老师可能就会对你印象不好或者什么的。

19:14

所以我可不可以说去那些呃资金比较雄厚的实验室，他更愿意对你严格要求，给你提很多要求，也不是那种资金雄厚的实验室，它就是你想做什么就随便做，就是那种放养的。对放养的，然后你想学的东西的话就是想学实验或者想学其他的就是很方便的那种比较普通的实验室就会带给你带来很大的压力，就做的不好会可能被老师说对，然后自己内心又有点那啥了。

19:47

你说的这种情况是你自己亲身经历，还是说听别的同学有传说这个事情？

19:53

其实也差不多算经历。也经历了也算经历因为，同学，其实你有和同学讨论过你们的经历吗？因为我的舍友们都是国中的，所以他们其实也比较认可你这个经历，他们也经历过类似的这种情况吗。差不多，也就是因为有些人我当时选择的面是基础和预防那边同时都选了，然后我就是有这么一个落差，然后有些要是同时待在同一个实验室没有走的话，可能就没有这个落差，你说的应该是指大二的科研轮转。

20:27

对。对大二科研轮转不是每个人都要转4个实验室吗？他不是强制的，你可以留在一个实验室留一年，所以说你的有的室友可能整个大二一个学年都留在一个实验室。我是当时按了轮了4个轮的，所以他其实对你轮转也没有强制要求，你也可以选择。在你一开始比如说像你舍友他只在实验室里面待着。你们当时选东西有是一个双向选择的过程吗？还是说当时是跟我们说是必须轮4个，但是有些人他就会私下和老师说，说完之后，因为我们当时是一个导师，最多带两个或三个学生，如果他一直留在这儿的话，然后老师的意愿又是我只带他一个的话，别的人有可能会进不去。

21:19

虽然是别的，同学都会对，我只是假设，因为我不太清楚真实情况会不会对这个比较有意见，比如说你在这个时间是一直待着，可能我想去我就去不了，因为像我不会跟国中班的管理的老师去反映这个情况的，因为毕竟是管理老师要求我们每个人要轮4个实验室，但是一开始说过这个事情，你了解的有人其实最后的选择权还是在于双向选择跟老师选择，对，如果老师不愿意多带一个的话，你也没有用。

21:54

其实就是老师最终的解释，有些时候是因为是2~3个，有些老师觉得我带两个就够了，我不想要第三个，有些时候可能是这样，所以说我可不可以说从表面上的管理上来说，它是要求你们轮33~4，对要求我们4轮4，但是实际情况我们在转的时候，可能导师就是不想换来换去的，我就带他一个对其他人进来我也不要是的，然后遇到这种情况，可能管理的老师也不会去干涉这个导师的选择，你可以跟老师去商，你可以跟管理的老师去反映，有些如果管理的老师跟另外一个跟导师协调好的话，你是可以的，但是好像最后大家也没有怎么去协调，好像也也没有这种协调成功的案例，可能跟老师反映协调一下，然后就进去，我没有去协调过，我也不大清楚。

22:48

像我还有一个比较好奇的，我不知道你知不知道，比如说你那个同学或者朋友就留在那一个实验室，一直留在那里面，他比较喜欢肯定是喜欢才留下来。

23:03

嗯你觉得从你个人主观感受来说，你觉得他留下来主要是因为什么？是因为精神压力比较小，可能资金比较雄厚，放养状态。他是说您说的情况有资金雄厚，这个是有觉得最大的是最大的情况是因为轮转的时候，老师只会让你看看，如果你长期在那边的时候，你可以真正的去动手做一些实验，可以跟着某些是一些师姐师兄的课题，就跟他们一起去做课题。

23:35

因为对于我们基础医学来说，科研还是很重要的，我可不可以理解就是说他在这一个实验室里面待一整年，其实慢慢熟悉了以后，他就可以跟着师兄师姐去做实验，就可以学东西了。但假如说我另外一个同学，我的4个实验室，这个实验室刚混熟了又出来了，刚混熟又出来，可能他们也不会带你干什么。

23:56

您说的就是我。我就是这样，就是这样对我真的是4轮了，4个之后，其实真正上手做实验的没有过几次。你当时在轮转的时候有没有考虑，因为你应该也就是比如说到第二个实验室，你已经发现有的同学就一直在一个实验室里呆着，你有没有想过我也要在这个实验室里继续待着，跟老师说一说之类的，有做过这个尝试吗？

24:23

之前是跟老师说过，但是老师说你们还是要轮转，因为才是大二，你们真正去做东西的时候也做不出什么，真正去学实验的话，做也可能做不出什么，当时感受怎么样？

24:39

其实也不是很沮丧，就是还好，因为毕竟你大二各种东西都没有学的太你其实你跟妈妈去做一个东西，就是换个说说的太直白的一点，有photo call，你按着那玩意儿加什么都可以加出来，但是如果你又不是你是学基础医学，你是学这个专业，你又不是做一个技巧，你说不是做一个机器，所以说你是不是可不可以说你还是比较想去学一些科研思维什么之类，的东西。

25:12

我还是想去学一些思维嗯，但是比如说啊，你在和同学讨论的时候会发现某一个同学他一直在实验室里，他们在一个像这种待的时间是比较轮的，不是很多的同学会不会跟你表达一种必要积极向上的一个情绪，比如说我在这个实验室里又学了什么之类的，你有听过他们讲过这种类似的事情，据我了解，当时有一个人一直待在同一个实验室，其实那个实验室的老板也没有，他让他做过很多东西，他就想因为在那边待的时间长，他可能大概动手的机会比较多，所以说不轮转其实只是说同学们自己认为我是有可能会去有很多上述的机会，实际操作其实并没有，也分实验室，有一些对也分实验室，所以说不一定看情况，一切都认为有的同学可能待了很久，也跟你差不多，也没学到什么东西，有的人可能就学到很多东西。

26:17

明白了。所以这是在你整个大二的时候，去轮了4个实验室，你觉得在轮转的时之中，我还有一个问题想问你，觉得你自己个人对。基础医学本身学科的兴趣很大吗？其实我觉得我兴趣还是挺大的。

26:39

所以在大二轮转的时候，你不会觉得说想去探探寻一下，比如说实验室做的课题我是否感兴趣，你觉得可以达到这个目的，比如说我在这个实验室以后，我去大致的了解，我就可以了解到这个实验室做的东西我是否感兴趣，或者说是否能做，或者说是否适合做。是在轮转的时候，就是在轮转的过程中，大家其实都是为了去看你对感兴趣吗就是轮转的目的。垄断其实最重要的一个目的就是看你对他有兴趣，对。

27:13

你觉得你你觉得轮转达到了他当初设置的目的吗，其实能达到的是因为你将来想去做一个东西的时候，你对这个兴趣，还有你想在这边做东西的时候，对实验室的了解，其实在轮转的过程中是是挺有作用的。在转活动之前，你有对基础医学某一个特别的领域有特别的感兴趣吗？还是说免疫和肿瘤？因为我妈妈有免疫，我妈妈之前得过免疫性疾病，所以我之前对免疫就很感兴趣。

27:50

你后来在冷战实验室的时候，是不是这两个方向？国中没有这两个方向。然后我现在待的实验室是肿瘤免疫实验室，国重好像因为我之前访问过别的同学，好像也有同学待过免疫的实验室，但是他可能有免疫，他可能跟各种关系不大，这个实验室他不做，他们显然是做b细胞的。

28:13

我知道你说的那个人，我们两个在同一个楼层，他们实际上是做b细胞的，我们实际上是做肿瘤免疫的。

28:21

好的。你说然后我再回到你刚刚一开始说的，那个东西就是考核要求，你们轮转的考核要求统一的，但是你们在冷战时候学习的东西可能并不一样，所以这个时候给你带来这个是让你比较不开心的一个事情。是可以这样说是吧？轮转考核的成绩算是你们的核心课程算。

28:51

按国中这边是算的，其实你们在轮转的时候，有那种理论学习应该是没有的，人生的时候，理论学习其实就是在实验室学习，有一段时间开了一次学习一些操作啊什么之类的，操作的话，也不是统一的看各个实验室带的成果，你们考核是什么？考核统一考核，统一考核某一个操作对。

29:17

我们当时同意考核了一个p3r然后又本来想考核western blot，结果那天就没有考核，没有考核行为什么，就是最后只考核 P加r。你说第二个是什么？Y三Bot蛋白组蛋白质硬件，为什么当时想考又没考？

29:37

因为其实很多人都没有去做过western b我们当时如果按当时来说，我自己去做western blot，我只有自己单独做过一次Vicente blot，而且west floor的它至少需要一一整天，一般情况下是两天，我们当时考核只是半天，所以我可不可以说是因为其实因为大部分人反映我没有做过 website block，所以他决定不考这个，当时有些人也没有做过PCI，因为有些实验室是做细胞的，所以这个考什么内容是他们那边定是国中班管理的老师对国中班管理老师定的。

30:19

你有没有跟老师反映过我或者说别的同学或者说你有没有同学反映过这个情况，就是你考虑这个东西我们其实根本就没有学过，有同学反映过他怎么是回应的，但是我也不大记得，不是你反映的，不是我反映。

30:36

因为这两个我都做过，虽然做的也不多，所以其实大二这44个实验室的轮转，这4个实验室有没有给你印象比较好的不好的体验就是不同？抛开考核这个东西来说，就单纯的轮转，整个过程没有的话可以不说过程其实还。挺好的。也没有什么让你印象很深的，开心或不开心，大二暂时没有。到大三你就有了，是吗对。其实大二像考核事情是给你带来一点不满的情绪，但是其实还好对还好，因为最后毕竟大家最后这个合同对我也过了，毕竟大家什么毕竟我们这个课程其实还是靠临床实践的，包括自己提交的东西，还有老师给你的评价，一般老师的评价，毕竟老师给的评价其实分还是可以的。

31:41

但是很主观。对，老师给的是真的很主观。所以说可能有的同学得了高分，你或者说排除你们可能周围别的同学可能对这个高分并不认可。

31:55

对，因为有些你像老师给的评价会跟实验室很有关系，你跟实验室里面成员关系不大，没有搞好关系或者很陌生的感觉的话，因为他们会问你实验室内部人，有些实验室会根据不只是老师自己打分，会根据自己研究生带教的情况，或者研究生对学生的印象什么的，这种情况下会打会根据这些，你是指比如说我是一个老师，我带了一个研究生，你是过来轮转的，我会问研究生这个人怎么样，然后根据他的他的影响到我的推出，可能会你们根据周围同学的分数的判断，你们觉得这可能是一个影响因素，因为这个成绩特别主观，只是老师给的。

32:44

而且一般情况下你在实验室的话都是研究生带教吗？有些时候都是研究生带教，你一般情况下一周去找老师签一个字或者去聊一些情况的话，老师对你的了解也也几乎很少，都是研究生带教。我可不可以说你们在实验室轮转里和老师接触的机会其实很有限，差不多也能接触，只是说没有那么多，主要还是和。

33:12

对，主要还是和那些研究生的学长学姐比较多。

33:16

你觉得从你的认知来说，是你个人这个情况，还是说你觉得周围同学都是这个情况，其实大家都是跟师兄师姐接触的多，跟老师接触也不是很多，所以最后造成的情况可能是老师打分的时候可能会问研究生的学生对你的影响，因为其实老师对你的了解并不是很好，你跟我讲讲你大三的事情。

33:40

大三我就先讲讲为什么大三的时候想退没退，因为大二后来国中突然按照搞了一个什么创新跟基础医学的视力计划差不多的，搞了一个要我们去做那种小课题研究，当时我们是组队了之后，当时他们让我当组长，因为当时已经把这个队组好了，然后课题已经选定了。

34:07

他们让你当组长是为什么可以了解一下推荐了，因为当时我所在的对，当时是我所在正好找的是我所在的实验室老师，然后就一起去我那边。嗯噢。所以挑了这个头就不能那个时候就退。然后当时就想大三的时候把这个东西做出来一点苗头，然后后面就让他们去做，我退就退了。你就退出。对，因为我大三的时候就想退。想退是因为什么？是因为还是因为各个实验室，还有一个原因就是大二一年轮转下来，发现自己对这边也不大也没有太大的兴趣，我还是想去做肿瘤和免疫。

34:58

当时你说你在到国中之前有去了解一下，你觉得他的发展情景也很好什么的，你难你当时没有发现肿瘤和免疫其实跟生殖关系不大吗？你可以换一个说法说，因为我当时临床没转过去，想拿跳板去转基础，因为这是二次转专业的机会。

35:21

你可以这么理解我当时带的目的想想离开。活力对。只是基础。会不会说技术可能会让你更接近肿瘤或者是对可以这么说。因为我们当时很多有两个选择，一个选预防，一个选基础，如果你退出预防，如果我从这边退出，我去做预防的话，将来我还可以靠执业证或者干啥，但是我最后还是选择基础，基础就不能考虑这些东西，基础不能。

36:01

基础主要还是做科研，对基础还是做科研的。大三的时候，你说的做了一个创新的计划，你做了一个组长，组队，他们推选你是是不是因为觉得你在实验室和他们组队的方向差不多，所以推选你做组长。

36:26

是的，我们当时是对具体想做啥就不跟您方便透露，对，我不跟您透露，毕竟关于有些导师我也听不懂，其实那其实可不可以说其实对你退出影响最大的还是大二，这些科研室科研轮转的体验不是很好，可以这么说，但是大三其实嗯没有给你带来一些什么不太好的体验，就是从整个大三的上学期，我觉得其实还是大三对我影响比较大。

37:00

那是什么事情？是因为我们当时做好对了，要开始开题都已经开题过了，然后要真正的开始去在这方面做一个小课题的研究。

37:11

但是当时我所在所指导的老师就一直不让我们开展进行，然后就一直虽然名义上虽然是给我们指导，但是每次去找他聊完之后，他都一直会让我们去自己去看文献，自己去找一些东西，就是没有一些实质性的指导，让我觉得我在这边还有当时说的过的细胞实验或者一些实验，我们那个实验需要用到这些技术，但是他跟我们说，说你们真正开展起来之后，真正去做这些实验学习的起来的时间会很短。

37:47

你去学技术需要的时间很短，所以你现在不着急，但是就这样一直拖到了疫情的时候，我们还是没有真正开始着手实验。然后我们这个是有一年，他完成的时候需要两年，然后去年7月份的时候需要进行一个中期考核，一年结束了以后对要有一个中期考核，你想想去年2月份的时候，我们什么都没有开展，还处于一个0的状态。

38:18

所以对我影响挺大的，我就觉得中期考核的时候，其实你们还是没有什么什么，都没有什么都没有。

38:31

所以对你影响还是挺大，的对我就觉得而且在实验室学习的时候，其实学的东西一直都是之前学过的那些东西，然后开展创新计划以后，跟这个老师去对还有什么一些我们需要学到的东西，他一直在跟我们说说你们后面后面真正开展起来学的，能够学的其实很快就能掌握，但是一直这么跟我们往后拖，不让我们学。

39:01

然后当时我跟我同一个实验室，有一个组员和我同一个实验室，我们两个同一个实验室，然后又因为这个状况，我们两个我们中途又退出了两个人。对，因为一直进行不下去。然后老师也一直不让我们往下进行，所以我想了解一下你们创新计划，它是所有的国中班的学生都要参与了吗？还是自我自行组织的？

39:29

当时是说是尽量大家都组队去参加，尽量组队，但我不强求你，但实际大家都走了。其实实际上一开始也有几个人没有组，但是第二期国中班开始组这个的时候，2017级的其实几乎全都组队了，嗯几乎都组队了。

39:49

没有组队的话，也是老师自己给了小课题，让他们自己去做。因为有些实验室老师不会给他们实验室自己的小课题让你去做，你只有去争取一个小课题。所以我可不可以说那些没组队，还是因为老师给了小课题，不用老师对老师给了小课题。

40:09

好的，比如说你们创新计划，你们组了一个队，课题的资金来源是哪里呢？果种播3万块钱，每个课题3万，对，国中每个课题播3万，然后中期考核合格之后再追加3万，中期考核优秀再追加3万。这个老师是你们自己沟通的吗是，对当时沟通之后他不分配，然后又要求我们自己尽量组队，然后老师那边需要自己去联系。其实很多老师其实这边很多老师都不大愿意去带这种大学生创业的人，创业创新的这种，因为为什么你你的感觉是为什么？

40:51

因为我觉得可能有些老师是新来的，他没有这些经验，还有一些觉得你带这些可能最后发不出什么文章来，对他也没有太大的用处。所以从包括你周围同学的感受来说，大部分老师你们创新计划可能大家都轰轰烈烈对足起来，但是找老师的话，可能很多老师并不是非常对，不是很愿意对很普遍的一个想一想。有没有那种组了队，结果找不到老师的？

41:27

这倒没有，你要找不到的话直接去找负责人，然后你想找哪个老师，负责人可能会帮你去协调一下，实在不行让你换个老师。所以也有不少的了，同学组了队找负责人帮你协调出来，对，你如果想找老师帮忙协调一下，你们这个老师是你们自己找还是协调出来的？

41:48

他当时自己找的吗？当时就问一下，同意我们说了一下我们想法，问老师愿意吗？老师当时说愿意。然后可以了。然后就开始准备一系列资料什么你说后来开题开题了以后，你们开题要答辩吗？要的，开题要答辩，就是在国中班的组里面答辩。该题当时你说老师不让开题，你今天不是老师不是不让开题，是老师不让根据你开题的东西往后做，开题只是一个理论性的东西，啊嗯开题其实不需要经过老师同意，你们自己在活动班里面负责人他们那边找老师来给你们审一下。

42:29

我们会答辩的，我们当时答辩然后答辩，然后就可以开了，开了以后，到了老师那边的话可能进展就没有进展。就没有进展对。你每次找他的时候，他都不让开的理由是什么？就是你们的文献研究不够到位吗？还是？对，他就觉得我们我们没有我们所做最近阅读的文献所总结出来的东西，不支持我们可以往下做，不能支持我们做。每一次都是这个理由是吗？所以他拒绝你们继续往下走这是，嗯有几次，我们一般是半个月左右去找他一次，每半个月找一次，找了一年都是这个样子。

43:26

其实也差不多就疫情期间就没有，第一个学期的时候，一个学期12月份不算，因为12月份考试。

43:34

考试的时候不算，然后从9月到11月11月底左右，每一次他都跟你们说这个问题是吗？每次他就是说他各种理由让我们开展不下去，让你印象最深的理由就是文献的问题，就是让我最深其实就是他说我们所阅读的文献所总结出来的不支持啊就是不支持，他要给你们一些知识，就是说你们应该给我们的指导，让我们去看这篇文章，然后去看某篇文章，看完之后最后去来把我们的想法跟他交流一下，他还是觉得我们不可行。

44:15

你有就是单独私下或者说别的组员有单独私下和老师交流，他拒绝交流之后，拒绝之后，老师会给你实质性的指导，而我们当时就没有什么太实质性的理论，相当于你说的实质性知道是别的组对吧？别的组你觉得哪一些知识性指导可能算算是实质性的指导？他们要怎么做，对。直接给你摆明了你要怎么弄。

44:46

第一步你要读什么文章，第二步要也要写什么东西或者做什么东西，别的组的老师是可以做到这些的，你和同学交流的时候，所以其实像我可是可可不可以说你们这个组算是蛮特别的一个组，因为其他同学可能并没有遇到像你这样的情况，其实有另外一个组以后，我们差不多他们开题一年了，也没有什么结果，也没有太多的进展，但是他们还是有点进展，只要他没有预实验结果，我们连预实验结果都没有。

45:25

他们像他们进展跟你们一样慢，他们有组员退出这种情况没有。你们老师当时是带你们一个创新组，因为你有老师只能带一个，好一个老师只能带一个，对。所以从从开题到后面疫情，然后到包括到6月份就是返校，五六月份返校。五六年返学校你们有找过他吗？找过也是还是一样，找过之后他就上我们改课题。就是方向改掉，对。理由是什么？理由是他觉得我们之前想的对他没有任何帮助。对他没有这种感觉。

46:11

对他的实验室没有任何帮助，然后让我们去做一个跟他实验室有些像虽然我们当时做的跟他实验室是相关的，但是他说对当事人是没有任何帮助，然后他就让我们做一个跟他实际上是有点关联的，只是去构建两个智力，然后他想把作为我们两个的作为我跟另外一个同学的毕业设计，所以他其实是比较想给你们提出一个方向，按他的方向来走。

46:41

然后最后的目的只是让我们在他那待，你算从大三大四大五就待三年的话，只在他那边做一个的毕业设计，他最后给我们挑明的是这个样子的。

46:55

你们有没有人就是私下里或者后面或者说微信或者电话也跟他讲过，我们一开始提的方向，你觉得不满意，为什么一开始不说了，有人提出过这一点吗？

47:08

这个问题真的没有人去问过他，好对，真的不太敢。然后真的就相当于大概一年在那边，所以相当于大三一年到了快要中期考核的时候，导师提出让你们换换一个方向，然后其实这一点就是让你已经无法接受了，是的。在别人都在往前走的时候，我跟我的另外一个组员我们两个一直在原地踏步。

47:39

所以在比如说像疫情之后，学期你们组就剩下你们的另外1个组员，对，本来是4个人知道。

47:50

像另外两个推出的同学是加入了别的组吗？还是说有一个您已经访谈过了？我访谈很多人，我我不知道就是免疫的。退出的是吗？对退出免疫退出当时我不在。他也是退出了那个组以后，然后就直接退出了国中。你觉得他也是跟你的想法是一样的，也不是吧，他主要是想做民意。其实我其实我跟他也有相同之处，有相通之处。

48:21

可能果冻甚至这个方向可能跟他不太大。对。但我记得当时因为访谈之后我是问了访谈是另外一个刚刚女生访谈，她说她是比较想保研去外校，但是国中的保研是必须本校，所以他想通过普通的保研方式，拿了保研名额去外销，但是他现在还是想保本校的，又想保本校对。

48:47

国中是一开始对我们承诺全部保研，但是现在没有成果，一直在变。所以这也是让很多人其实我也有点不高兴，让很多人不高兴，因为一开始给大家的保证是每年只要你不挂科，全额的1万块钱的奖学金，然后全班30全部保研，然后也不限制你保外，一开始最初的宣讲是这样子，你对这个印象很深是吧？

49:20

因为我对保研印象很深，所以我可不可以说其实保研这个政策对你信心也蛮大的，是的。也可以这么说。

49:30

你觉得是基础专业前景好，对吸引更大的是保研前者后者对我也没有太多影响，还是前景对你的心理会更大一点。然后之前一开始在宣讲的时候，说是可以把外向后来逐渐的变成了不可以把玩笑稍等一下。你周围有人有想过，因为这个是保研的东西想退出吗？有。有人真的退出了吗？只是有些想法还没有，不是去免疫那个吗？不就真退了吗？它其实是因为主要应该是保研政策才推出的，其实他也对升职这边也不大感兴趣。

50:15

你们组另外一个学生退出你们组推进退出的另外一个同学，他没有推出过中。没有退出过，他是去了别的实验室，对他在别的实验室。我可以你们创新的团队，大部分的计划都比较顺利，还是说像你们这种中期停下来的还蛮多的。

50:39

其实剩下的各个实验室之间还是挺顺利的，如果老师一直在指导的话，一直让你在推进的话其实都挺顺利的，你有和别的同学别的组的同学交流过他们创新计划的一个进展的情况吗？我舍友他们都和他们交流过他们的感受，他们的感受你有什么比较深刻的印象？他们虽然有困难，但是还是能往前走，虽然有困难，但还是能往前走，困难是指什么困难？

51:10

做不下去或者也不是走不下去，到这个点有点往下做不了的时候，但是一些指导或者换一些方向，换换一些方式啊，还是能往下走，可能是他们自己本人做实验做不太下去，但是老师的指导还是对还是能让他们往下走的。

51:31

可能他们的困难跟你们困难不太一样，你们老师可能指导不怎么知道，他们可能就自己做实验做不下去，跟老师没什么太大关系。你有跟几个组的同学交流过啊？就是这个创新其实最多交流的就是一个组，因为方便对。所以创新计划没有持续的推进下去，给你带来了比较大的挫败感。可以这样说对可以的。所以你就是开开始怀疑，我在这儿我是对我还有我不适合在这边继续呆下去，就是说我假如继续待在国中的话，我可能还要在这个实验室在这边继续做，他也给我的方向可能我学学到的东西或者说我能得到的，东西太少了，或者得不到我想要的东西，是的。

52:32

你可以这么理解。我只是提一个假设，提你进了那个实验室以后，有没有可能就直接退出这个实验项目，然后去加入到别的组，你有想过事情吗我有想过，但是因为我当时是组长，我不能不能在我组员还没有退，完全退出的时候我就先退。

52:54

你当时有跟组员讨论过你的这个想法吗？可能我们俩一起到别的组去算了，这个都根本推不下去俩，所以现在我们俩我退了郭总他退了实验室，最后就剩两个人，组员推了实验室以后是去了别的实验室，对他去了别的实验室，就加上相当于去了别的组了。

53:20

对。你当时退出的时候有跟组员想讲过，讲过我们两个商量好的，我就是说他因为我们两个可能说的有点过分，就已经耽误了一年，所以就不想接着在这耗下去。然后讨论了一下，然后他说他还是待在锅中，我说我就直接退了。他有圈里留下来吗？你跟我一起没有。这倒没有。因为我们当时都是轮转的，他回到了他之前轮转过的实验室，他轮了几个？三个。

54:01

就回到了原来人的实验室里面，有一个创新计划，他就没有接着做课题，他还是你现在做什么？当时给他的一个课题没有，他跟着师兄师姐他们让他做什么他做什么，所以他其实现在也没有参加任何的项目计划是的。我可不可以说其实7级他现在没有任何计划，那也不一定，其实我从那边退出之后，我对那边的了解也不是很没有怎么了解过，所以你对他后面的事情就不是很了解，我对后面就不了解了。好的。你有想过转别的组，但是你可能考虑到组员说算了还是不要直接转，后来又直接又想退。

54:47

我倒没有，我是一直想大不了算了直接退。其实我这个挣扎是在疫情期间，我已经挣扎了半年了，在疫情期间我就开始在挣扎，你在挣扎的时候有没有父母讨论过这个事情？讨论过。他们怎么讲他们说，你开心就好。他们一直只要我不做违反道德伦理的事，我肯定就好。好朋友什么之类的有讨论过。你开心就好，对，真的就是我开心就好。

55:17

所以其实可不可以说像大三被耽误了一年，让你很不开心？可以这么说，大二的话考核要求不是很清晰透明，主观性太强，可能让你有一点不开心，但倒也不至于说说想想怎么样，但是大三这一年下来让你觉得非常的不开心知道。

55:42

挣扎的时候有没有和你的就是现活动班的同学比较好的舍友之类的讨论过，说你想推出讨论过跟我的他们也说，其实如果你真的不敢相信，你还是想做肿瘤或者想做免疫的话，你还是可以试试。虽然现在挺晚，但是你退了之后，毕竟本科生要求也没有太高，你退了之后，虽然你什么都没有，但是你有学习经，你可以再去一个新的实验室去学习。

56:09

我想问一下你当时组队创新计划和肿瘤免疫有关系吗？没关系，因为国重没有什么做肿瘤，也没有什么做免疫的话，老师也不大愿意戴大床。你刚刚说你其实对比较感兴趣，做了创新计划，我想提一个假设提到创业计划，你说其实跟你的兴趣点不是非常的重合。

56:35

对，因为但是也是有重合的地儿，因为肿瘤也有一点点，但假如可以顺利推进的话，假如说这个老师像别的组的老师一样，给你很多的实质性的指导，其实你也可以继续做下去的，你还是愿意待在国中去做这个东西，愿意待在国中，可能不大愿意吧，为什么会因为我之前您可能忽略了忽略一个点，我当时说了，其实我大三的时候就想退，然后但是因为组队，然后想着说对这个事情如果真的能推展下来，那个时候我自己再退，是能够有那种就有余地的让我退，因为你事情都可以推荐下去了，最后换个组长无所谓，因为这件事你可以继续往下走，但是你不能往下走的时候，我退就不合适。

57:31

所以说你大三有退的想法，其实主要还是来源于大二。那个时候不公平的考核方式。可以这么说吗？也不只只是，也不仅仅是不公平的考核方式，就是活动总会果中总会突然想出一个什么东西让你立，也不算立即执行，就是在很短的时间限制内，你要把它搞突然的感觉给你一种措手不及，就像突然组这个课题的时候，就突然给你措手不及，6月份给你通知，7月份就要答辩。

58:11

当然有什么这种突然让你布置的任务一定要短时间内完成，其实当时考核突然给你的一开始考核的要求都写了，当时没有说要考实验或者干啥，然后在这个课通知你们要进行小课题，然后就突然通知你们，通知完了之后，在头考试之前要不是在头答辩之前又突然通知你们有这一个考核，各种通知都让你措手不及的感觉，这种措手不及的通知，主要是跟教学相关，会不会有没有其他的比如说类似什么实践相关的，都是也让你比较感觉不太开心的。

58:51

包没有？主要还是考试方面。

58:53

老师给你突然给你布置一个任务，或者说突然要说考一个什么东西是吧？突然让你措手不及。其实像这个东西在开学初的时候就给你们讲清楚比较好，哪些要考，哪些不要考考哪些内容。

59:09

对，就突然给你措手不及，像这个体验让你不太开心。像你的同学有相同的感觉吗？你有跟他们吐槽过吗？这个事情我们之间都相互吐槽过。他们也觉得比较。所以我可不可以说这两点构成了你有谋生了，想要退出的想法可以突然性的突击性的考核工作和不太透明的考核要求。可以说还有没有有没有什么其他的。谁说没有了，现在就没有了。暂时就没有对暂时就没有。

59:52

我可不可以说进了基础，就是让你更加接近了你的兴趣点。但其实你进来了以后，你进了这个班其实跟你的兴趣点只是说有一点接壤，并不是完全重合是吧？然后再加上有一些让你这些包括考核这些东西，让你也给你造成了很多不开心的一个情绪，所以你就更加坚定的谋生的要推出的一个想法。

01:00:26

可以的，你现在在基础医学学的怎么样感觉，我觉得我挺开心的，虽然有些时候有有点累，但是我觉得我挺开心的，累是指什么？累啊？

01:00:36

也不是累，有些时候也不能说累，有些时候想学的东西比较多，然后安排的比较紧，然后觉得有点累，然后但是我觉得我还挺开心的，安排的比较紧，说明是你自己安排我自己安排的自主安排的一些时创新计划吗？还是实验是什么东西？也不是，就是一些什么生性就是生性相关的，想想去学一些生性相关的东西，然后就拍了自己你自己去找老师去跟他学吗？还是有网课或者网课或者别的？

01:01:23

另外一个我想问问你觉得不只是国中班就是从上大学以来，有没有让你觉得最有成就感，或者你觉得你做的最自豪的一件事情？就最做的最做的好的一件事儿。嗯最有成就感，讲不出来的话也可以，你觉得也没有什么的话也可以不说。最后成就感就是我大一那年暑假去支教了，我就去完成了我高中的时候的一个什么药就高中的时候就想去支教，对。

01:02:03

是在哪里啊我在徐州徐州c娘那边，农村地区这叫教理科吗？我当时给他们上的就是跟医学相关的那种医学小常识，一些小城市暑假他们不是都放假了，还是可以的。还是可以的。在学期末相当于或者学期初。不暑假就是暑假对。你当时想做支教是受什么影响吗？还是自己想我当年的一个愿望，一直都想对我当年的一个愿望，没有说什么事情，没有什么事情，就是我当年的一个愿望。

01:02:49

在上大二以后进国中班以后，有没有学弟学妹来问你王玉国的事情？有。他们问的啥问题你还记得吗？他们问印象最深的或者问的最多的。能保研吗？能保研吗？能保外吗？能保研吗？能保外吗？当时应该那个时候的政策应该是可以保研保外你都是这么说的，你也是比较推荐他们来的。我当时也不是推荐，我当时我只是给他一个理性的看法，就是就是什么让他们还是自己决定，因为我那个时候我就其实我在我退出之后也有人来问过我，有人来问过我，你为什么退出？

01:03:42

也有人来问我，20级的学妹也有人来问我学姐怎么考过程，你都会给他们知道，对，我只是给他们说一些你说的理性的看法是指什么，你可以跟我大概说一下，你来国中你可以学到什么东西，但是你会有哪些限制？

01:04:05

在国中的话其实限制还蛮多的，当时我们进国中的时候，要求我们不可以去做其他那种什么校学生会的成员，内部的任职不可以，有，然后校都不可以吗？学生会的任职各种任职尽量都不要有，包括社团这些都对，但是我当年还是偷偷摸摸去做。

01:04:31

做了一个社团的社长，所以理论上来说他是不允许你们做的，理论上是不能让我们有任何认知，然后他的理由是什么？他有说理由，吗还是专心科研，然后还有理论上是要求我们其他学院的，你像什么市旅计划这些的说我们可以不必参加，也不是说可以尽量，然后我们参加自己的项目，对参加自己国中内部的项目，不要参加那些事情对不对？

01:05:02

大二有什么项目吗？能让你们参加。嗯也就是旅计划还有什么？他不是不让你们参加是有计划吗？那个时候我我是说国中班，他既然不让你们参加外面的，他自己从而有吧？没有。只有那个课题的还是大二末下学期才出来的，所以相当于大二你们可能想参加那些实体计划，大创不行。我当时还是泡沫参加了一个当时我们学院自己的一个寒假的学员，就是基础医学院的寒假的一个培训活动。

01:05:37

其实寒假什么培训什么培训什么就是旅游计划的一个寒假班，参加培训那些学生怎么去参加市里计划，类似这种东西的培训也不是听听讲座，然后做实验的那种啊相当于是与计划一个导向性的讲座之类的。

01:05:55

对。因为当时暑假了，他又不能限制我跟寒假不就大二的那会儿，像他归发了这些规定以后，你有跟国中班的同学吐槽这些事情吗？不能参加学生会，参加社团，不能参加市里计划或者大创这些东西，其实我也不算吐槽，因为我自己偷摸的也去做了社团了，对我自己也从没去了，有在你面前也算吐槽吧，他们吐槽的学生会这个事情比较多，还是不能参加别的学院项目比较多？

01:06:28

有些人都做到了，部长，然后你让他直接退了，他其实很不甘心的，也不是很不甘心。

01:06:35

你像如果现在他又说不能保研，要前40%才能保研，你要是那啥的话，你这些考研的话，这些也算你一个加分的亮点，然后你突然退了，然后证明什么各种证明都没有。

01:06:50

有些人真的就是刚生完部长就退了，你会把这些东西跟学学弟学妹来讲，我会跟他们说很有限制，学到什么东西你会给他们讲。会跟他们讲，因为其实也能学到一些东西。你觉得你最大的收获是什么？这两年大二大三最大的收获真的就是因为之前护理对基础医学虽然以前了解过，但是只是面上最后弄到一些礼仪里面啊。有面积里是指理论吗？真正的从一开始表面知道吗？

01:07:32

最后算是一个也不算真正的明白就更，明白了，就明白了。所以一般像他们给你呃学弟学妹来问你，你主要是讲这三方面的事情，理性分析，不能把我自己的主观带给他们，就是你理性分析它好处和不好的地方，你自己来判断你愿不愿意来。是的，所以你跟他们讲这些好处和不好，主要就是这三点对吧？一个好处就是可以真正的学到一些比较内在逻辑上面的知识，还有一个更实际的东西，一年1万的奖学金。

01:08:07

嗯噢，对还有奖学金不让我们评。评学院的只能拿他的奖学金。学院奖学金会比这1万块钱更多吗？也不多，但是你学院的不能评，你其他的什么杨子江什么玩意儿你也不能评。校长你也不能评，校长什么之类，的对你只能拿他的奖金。所以其实对于某些学生来说，他是有可能拿到比你们更多的奖学金。

01:08:40

所以其实表面上看起来还不错，实际上表面也是有一点点对表面风光，您也会把这个消息跟学弟学妹讲要注意这一点。对我会跟他们说他们会在意吗？就是奖学金，有些人会冲着奖学金来。

01:08:54

有些人你像那种因为当时转这个的时候，好像是要求你成绩在前50%就可以，你前50%的话，很多人你在前50%你在有些学院就只能拿300块钱的奖学金，在有些学院前50%也是，比如说我比较好奇，像他们冲着那些奖学金来，可能50%只能拿个几百块钱奖学金来，这一下子拿1万，你在跟他说了限制以后，他们有动摇过吗？还是说？

01:09:25

这我倒不清楚，你不知道我对我不清楚。你只跟他们说了，判断还在于他们自己，我只是把这个事实讲给他们听。有学历你说刚刚说有学历有没有问你，为什么退？你有。如实的跟他讲了，就说我跟他说的是我不感兴趣，还有我觉得我待在这边，是我可能是我适应不了他，我不能让他去来适应，我适应不了他，然后我也不开心，我在这边不开心。

01:09:55

他们不会问你哪里哪些事情不开心，或者是哪些让你感到不感兴趣吗？还是说他们就不问了？他们就关了。你也没有跟他们讲，这个老师不让你们推进。 Cca这个课题的事情其实也甜的说过，浅浅的说过一次，只是没有深入的跟他们说，可能你推进创业计划的时候，可能不会像你想的那样顺利，也会跟学弟学妹讲这个事情。

01:10:26

你说到了什么20级的都都来找你，是不是？因为你算是第一个退的吗？还是也不是第一个退，因为我当年成绩我是笔试第二境的，然后我当年曾经推出让很多人很惊讶，也不算很多人很惊讶，很多人其实都觉得很正常，我退国中班的学生对同学对他们已经能感受到你有很多的不开心了，至少我身边的朋友是我舍友，还有是因为他是基础和预防，基础的很多也能感受到我的不开心，预防那边因为不大熟，虽然我们是虚拟班不在同一级一起上课，只有开会的时候会在一起，所以他们其实很早的就能感受到你已经有很多不开心了，所以到最后你退出的那一刻，他们其实也并没有很惊讶，也没有很清楚。

01:11:34

你觉得国中班有没有让你感觉到很有压力的事情？或者学习过程中，或者说科研过程中，其实你到国中班的时候你就外他给你画的，他给你的那种什么一开始给你的保研给你的奖学金，这些就会把你圈到一个笼子里，让你觉得你没有什么压力，但是到后面他的政策一直在改的时候，就会让你压力很大。

01:12:03

因为说的很现实的话，各种保研的东西，你在这里面待的时间久了，你是你没有什么东西，你没有什么资本拿出去跟别人去竞争，因为各种像限制，没有什么资本拿出去，可能我的学生活动，我的其他的科研项目或者支教项目这些东西，因为我来到你这个项目里，我没有办法去参加，你本来承诺我可以保研，后来又什么时候不一定。

01:12:31

其实我是损失了很多的，对，如果你是想读南医大本校的话，其实这些也不算东西，但是其实很多人都是想保外或者还有一个要求，当时我们暑假的活动，因为对于本科生要求你是要有多少个活动你才能毕业，寒暑假要有什么活动，三下乡的活动或者其他活动能毕业对对对第二个课堂。

01:12:55

对第二课堂，然后他是不让我们走别的第二课堂，让我们走国中的课堂，你们国中第二课堂是啥？实验室就是暑假的实验室学习，大二的暑假对二的暑假的实验室学习，你只要大二暑假那个时候在实验室学习，你们第二个课堂的分就可以修满吗？也不是吧，第二课堂要4个还是3个，所以要每个暑假都在那搞。

01:13:20

其实对，而且就是说我可不可以说团委的那些其他的第二课堂的分你们都不能修，他是要求你们必须修自己的，那个也不是要求我们必须修自己，他就尽量要求不要出去参加活动什么的，他会经常要求你们，但也不会说阻止你们，但是你不去实验室肯定就跟老师印象不好，就让老师觉得印象不好，印象不好你主观分不就低。

01:13:46

但是会间接的影响你期末考试的分数，但是我当年你那，你其实大二中间寒假其实已经有第二课堂的论证了吗，我二大二暑假是走了过重的，大一暑假是我就走了支教的，所以只有暑假才有，寒假没有。

01:14:06

对，寒假没有，因为寒假时间太短。然后还有还有第二课堂的话，平时的活动其实它也不大愿意让我们参加，但是我当年我是属于偷偷摸摸，你不让我干啥，我可能还会干，我只不过偷偷摸摸干了。因为毕竟当年我是某个社团社长，你觉得退出了，这个社团还是很可惜的。也没有退出，我其实假如对他让我退，其实还挺可惜的。

01:14:40

你说会影响主观分。我有一个点我没有搞太明白，当时大二轮转两个学期要考试，你说那两个学期的分数考核是比较主观的，但是支教像二第二课堂啊就是支教，第二课堂你们是暑假才有的，所以其实对你们大二的分数其实影响不是非常大。

01:15:04

还有一点，我们那个分数是根据你的考勤，我们一开始是一周5次，后来因为实在是5次很难达标，就改成了一周三次。

01:15:19

大二的轮转是吗？对，在实验室。一周至少老板要一周看到你三次。你不是说老板一般都让研究生来带你，对，但是老板可能会在转一圈，对。我们当时有严格的考勤是谁来考？老板自己扣。老板来转，一圈自己勾一下。你在那上面写，然后你去找老板签字，老板考勤其实占挺大部分的，是吧？所以像第二课堂的话，假如说你不参加他的暑期实验那你，其实他也不是很愿意让你去参加国中以外的第二课堂的项目，所以会不会影响到毕业啊？

01:16:13

也不是，假如的话，不可能，因为他给你你过不了他就给你安排实验室，你看有实验室，这不就算一个项目，有项目你不就可以毕业了。主要还是我们要求会要求你在实验室暑假要留在实验室，如果你留在实验室，你就没有时间去做别的地儿，可能在时间方面就会有一个冲突，所以大部分的股东班的学生是不是暑假都会留在？

01:16:41

是的，我那年留到了8月20号，周围同学和你自己有什么想法？啥想法？不要做这个暑假软件，自己外面悄悄去外面做，感兴趣也有人去做。

01:17:00

那那暑假就没参加，暑假也参加了，他不用教总数，他拿因为他们有些就三三四天。搞定了吗？你三四天不来，你那个时候学校又没有完全放假，你可以说你没有完全放假，你就去搞那玩意。短时间是可以，你像长时间就不行，对七八天这种就不行了。三四天你怎么样都可以过去了。所以其实我可不可以说其实想去做一做外卖，这种跟科研不太相关的时间的同学还是蛮多的。

01:17:38

可以说因为但是它又要求你一定要做科研方面的第二课堂，其实给你们限制蛮多的，其实那个暑假也有人做的不是那个了，也不是第二这个了。第二个因为当时那就直接跟老师说我不参加熟悉的论断了，也不是因为当时要求我们至少暑假在学校待四中，要求对要求我们至少得4周，有的人可能就没有带这4周或者带了3周，然后去做别的，类似这种可以这么理解。

01:18:12

明白了。嗯嗯嗯。其他的我其实没有什么。很感谢您的配合。好的。来看看我有没有讲漏的。好的。其他没有什么。你稍微等我一下好的。没事了好。你放这吧，我来人拜拜。拜拜。
