# Supplementary material for: Why do you choose this program?—A decision-making model of medical students based on grounded theory
Source: PLoS One. 2023 Sep 15;18(9):e0291634. doi: 10.1371/journal.pone.0291634 (PMC10503722; doi:10.1371/journal.pone.0291634)
Supplement: S1 File — (ZIP) [file pone.0291634.s001.zip › RAW DATA/P10.docx]

At 00:00

, you can talk about things that have a big impact on you, and you can also talk about your er views or your emotions, whether you are happy or not, you can actually tell us about it. Then we mainly want to talk about things that have a greater impact on your thoughts, or things or emotions that you are more impressed by. Yes, before the time is up, we need to talk about the ethics of time.

00:24

In this interview, the interviewees participated on the principle of equality and voluntariness. The releaser must truthfully express their thoughts and cognitions, and confirm that they meet the social security conditions. The interview process will be recorded, and the recorded materials will be anonymous. The form is used for scientific research and will not be disclosed to any third party. During and after the interview, you have the right to cancel the right to use the researcher's recording materials. Do you know and agree?

00:46

Yes, then please introduce your major and grade first. I am a grade 17 student majoring in basic medicine at the School of Basic Medicine. You are also technical before entering the middle school, and I studied nursing before entering the middle school. Mathematical pair. The first question we want to ask is that we want to know what you know about the Shareholders' Office before you sign up, and what are the ways to know it? Before signing up, there was a lecture, and then I went there during the lecture, because I was in nursing when I was a freshman, and then I wanted to change my major, and then I wanted to switch to clinical at first, and then I thought because I was in middle school at that time. It was preached over there that this was the first time to change majors, and this was an opportunity to change majors for the second time.

01:36

Then I just said this when they advertised and said after the propaganda that you want to take the exam after transferring to a major, so it is equivalent to your opportunity to change majors for the second time. I started When I transferred to the clinic, I didn’t transfer it, and then I chose it. You continued to say that I chose the middle school side, because I signed up for the two at the same time at the beginning, because everything will have different choices, not different. As a result, so I prepared both hands.

02:04

Then the two prepared at the same time, you should be a science major, right? Yes, I am a science student. In clinical practice, I would like to ask if you applied for clinical medicine when you changed your major. Pediatrics, right? I applied for pediatrics because I wanted to do pediatrics, or because of other reasons, because I wanted to study clinical. I wanted to study clinical at first. I applied for the male first exam. I was from Tianjin at that time. According to the previous rules, I could be admitted to the clinic. However, because the admission score of Southern Medical University in Tianjin was very high that year, I was transferred. When I got to nursing, I thought because I wanted to apply for the clinic at the beginning, and then I thought it was because I had this opportunity, and then I grabbed it again.

02:55

I would like to ask when you signed up in high school, you were a science student, did you say you must apply for medicine when you signed up for a major? Or do you want to? Engineering thinks. What I was thinking at the time was to report all to medicine. All medical reports. What is it, or are there any other considerations? Or say interest or say things. In fact, in order to study medicine at that time, it was also because my grandparents died of cardiovascular diseases, so I just wanted to study medicine, so I wanted to make a little contribution to my family, so I knew that I must study medicine.

03:34

Right um. Then after entering the school, in fact, from the moment you entered the school, you just wanted to switch to clinical practice. Anyway, you didn’t want to study nursing, but it wasn’t that you didn’t want to study nursing. You might feel that you were not suitable for nursing for various reasons. In fact, when I was a freshman at the time, I was also struggling with whether I had to transfer to a clinic, but after a year, I found that I was not really suitable for nursing. I can be more specific about what kind of nursing care, people are the kind of little girls who are relatively fresh, or the little girl is the kind of character of a little girl, I don't think I am suitable.

04:15

You said what kind of personality do you think is more suitable for nursing, the kind that is gentle and elegant. You think you belong to that kind of female character, and then later you think you can study clinical or basic scientific research, so you signed up for both, yes. Have you heard from other sources? Or is it only from last year? At that time, the middle school class was a publicity meeting, and it was also on the WeChat public account. Is the official account your nursing official account? I don’t remember exactly about nursing, but they were the school’s public account at the time, and they seemed to be from Southern Medical University and the official WeChat account.

05:06

You have discussed this matter with your classmates, not only in high school, but also in high school, or in your family. At that time, you chose to change majors and then discussed with my good friends and good friends. Pass. A friend is a good friend from high school to a high school friend um. Does their opinion matter to you? They just think I'm happy. So I don't have any opinions, I don't have any opinions on them. You haven't discussed this with my parents. My parents are the kind of decisions I make. As long as it's right, they will support it, as long as I want to do it. Decisions generally do not violate any ethics or anything in particular, and they will support them.

05:46

Mainly right, just that.

05:49

For example, when you applied to study medicine in high school, they also supported them unconditionally. They supported them unconditionally at the time, but when my grades were down, all kinds were tea shops. They were also considering letting me apply.

06:22

At that time, they just saw that my score was a little bit, so I wanted to apply for a science and engineering degree, but in the end, it was ok after the final coordination. Do they have any specific requirements for you to apply for a science and engineering degree? Or a computer or something? He said that he just wanted to be, because I am from Tianjin, and then my parents thought that I should try to be in the Beijing-Tianjin-Hebei area as much as possible, right? They are engaged in science and engineering work.

07:26

No, my dad is a civil engineer, so he thought that because I was a science student, he could choose some science and engineering majors.

07:40

They think that in the Beijing-Tianjin-Hebei area, a brother’s major in science and engineering is mainly close to home. They think that being close to home means that it doesn’t matter what subject you apply for. It means that you want to be closer to home. The subject is my own. Sure, they don't interfere, but their first decision is to stay as close to home as possible.

08:00

Why did you come to Nanjing Medical University for fine-tuning, right? Which one is my first choice?

08:07

At that time, I didn't want to go to the Northeast because of all the results, because if I went to Shenyang or Harbin Medical University at that time, I could go directly to the clinic, but I didn't want to buy more, so I chose Tianjin may be a medical university in the Beijing-Tianjin-Hebei area, and I may be able to attend, but I can't get a good major. Because at that time, I wanted to rush into the clinical practice of Southern Medicine. clear. So it doesn't seem to have any effect on your opinion after discussing it with your family, no. Then after you come in later, you can tell us if there was anything that attracted you at that time, because after all, the National Class is not a clinical major, why do you say you must leave nursing and come to basic medicine, I Let me tell you about my mental journey when I changed my major at that time. I wanted to switch to clinical practice at that time, and then the summer vacation should be the review stage. During the summer vacation, I went to participate in a volunteer teaching club activity during the summer vacation. Then it took up a large shift for a month, and then I suddenly fell ill after returning home, and then a period of time passed, I felt that my review time was not enough, and then I was very early during that time. I couldn't read the book, and before that, my grades were a little overhang, because if I was transferring to clinical, I needed to be in the top 21%. When I first went to the counselor to check my grades, the counselor said that I was a little overhang, and then I I have been wandering in the clinical and basic areas, because at the beginning, it had to fall back to when I applied for volunteering in the past year. At first, it was a feeling of rushing because of the male first.

10:01

My friend told me at the time, he told me because his father is a doctor, and then he analyzed these for me, and then he said that you can also learn the basics, because the future development of basic medicine is still very good. Yes, at that time, I went to learn about basic medicine, and in the end, although I also went ashore here, it was also nursing, and then at that time, because the counselor told me that your grades were not so good, then I considered basic medicine. I broke down with the clinical medicine, and after the final result came out, I was in the top 20%, and then I finally rushed to the clinic.

10:40

Then later , because I couldn’t watch it anymore during that time, it was very bad. At that time, I felt that I couldn’t get into clinical practice, and I couldn’t transfer, and then I just wanted to change majors again. I don't want to study nursing anymore, and then I actually transferred to a high school in a foreign country because I wanted to change my major. Can you understand what I mean?

11:01

I understand. You said that you had a chat with your classmate's father at that time. They are majoring in medicine, and they said that the development of basic medicine is also relatively good. Good development means which aspect has better prospects in the future, because the foundation is still relatively promising. , but the prospect is good, which means that the society may recognize and demand the profession in the future.

11:29

There is also a need for recognition , which may be mainly due to its relatively high recognition. I was interrupted by something just now and forgot to ask. Do you think those who study nursing need to ask Erya what is going on to make you feel this way, or is this judgment subjective.

11:54

Yes, I subjectively think this is the judgment. In fact, you think it is like this since the first year of college. It is because of all my friends around me, my former teachers, and then my friend's father. Mom or something, they always asked me what did you study?

12:10

I said that I studied nursing, he said that you are still going to study nursing because of your personality. The unanimous opinions of the people around me are all you said. A little bit influenced by society, or did you actually think so before? Then the opinions of the people around you deepen your opinion, and the impression confirms your subjective judgment. I think it is correct to say so.

12:37

You said that the future development prospects of basic medicine are better and more recognized. Can you say that I am just a guess of my own? You can also deny whether it is possible to do this thing, and it can bring a kind of experience to human society. Something, or its social status is relatively high, so you think its prospects are better, and you are more able to accept it. Well, it's okay to say that you think it's hard to say it yourself. As for the basics, I will talk about my views on basic medicine. I think because of the clinical, it is from the person, you are only to treat a disease, you can only guarantee that the disease is optimistic on the surface, and basic medicine If so, you can go further and more slowly to dig its roots.

13:34

I may be a science student. I prefer to dig its roots and find its roots. I think basic medicine can allow me to find the roots. It is simply that you prefer to explore the essence of a thing. Or do you think that a subject like basic medicine may have a greater influence than clinical medicine. Which one do you think I prefer to dig for its roots, or it is more related to yourself. But when you were making something, have you ever considered that it might be something that can really explore its origin, but in fact it is also very difficult. Did you think about it at the time? Actually, you did, but you can say it casually now. It's easy to say that there is nothing to do. If you think he can't do it, you haven't done it, so you signed up for a temporary transfer to a clinical major, including going to the national affairs class. In fact, can I say that the conversation with the student's father has strengthened you. The idea of is to let you say more that I think I think it is possible to switch to technical medicine, or that there may be little clinical hope, so I think I need to think more about the technical basis, and which ingredient will be heavier.

15:10

I think these two ingredients are different and they are not too heavy, because the main thing is to follow me, so I may find it after a period of precipitation, because I have a little bit of it. Very lively, but in fact, I still have a feeling of crowd communication phobia in my heart , so I feel that I have too many clinical dealings with people, and I may not be able to rush down. After we've just gone to the junior high school, you can tell me something that has a more profound impact.

15:54

Can you start by saying when you quit? I was introduced during the summer vacation of last year in my senior year. After two years of sophomore and junior high school, I actually wanted to quit when I was a junior, but for some reason I didn't quit. The epidemic is not in the second semester, not because of the epidemic, because of some other reasons, you can tell me about it first, that is, the second and third years of your sophomore year are the period when you are in the junior high school, which will affect you.

16:24

The happiest thing, the happiest thing, if you don't have it, you can leave it out. The happy thing may be summed up by Director Dong.

16:35

Before I knew basic medicine from the face, then it was because my nursing had never been exposed to basic medicine at that time, and then when I was a sophomore, I might actually go to the laboratory to study and really go to the majors, from face to face. Going inside to understand a little something, I think let me say let me change from ignorance to a feeling of understanding a little bit, I think this makes me quite a bit.

17:12

In fact, let me put it another way. If you don’t change your major, go to study nursing. In fact, you can continue to study nursing, and then continue to deepen your knowledge of nursing.

17:25

Do you think that if you failed to transfer to another country at that time, you can feel the same way in nursing, and can the study of nursing also bring you this feeling? It is also possible, because it is also possible that it is not necessary to say it because it is difficult to say later. It does not matter. Just want to say your own opinion. You can talk about the middle school class now. Things that made you unhappy at that time, I may think that there is no unified standard in the middle school class. What standard are you talking about?

18:08

The assessment requirements he gave us are the same, but it is for each tutor. He asks each tutor to meet our requirements. Different tutors have different requirements for students, but he The final assessment standard is the same , so there will be conflicts here. You are like the laboratory I have been in before, the teacher will not let you go to the cell, and will not let you go to the cell room, but our final assessment will require you to go to the cell. Experiments and related knowledge of cells, and then you will be at a disadvantage, and you will be at a disadvantage, and then there is this, because different tutors are stocking to some tutors, and some tutors will be strict with you, you are like some experiments. The lab is rich, let’s just say, some labs have money and you can recruit others, and it doesn’t matter, while some other labs are more ordinary kind of labs. It might make a bad impression on you or something.

19:14

So can I say that I go to those labs that are well funded, er, they are more willing to be strict with you and give you a lot of requirements, not the kind of well-funded labs, it is just what you want to do. Do, that's the kind of stocking. For those who are stocked, then if you want to learn something, you want to learn experiments or other things, which is very convenient. The more ordinary laboratory will bring you a lot of pressure, and it may be possible to do it poorly. The teacher said it was right, and then there was something in my heart.

19:47

Did you say this situation was your own experience, or did you hear other students have legends about it?

19:53

is actually almost an experience. It is also an experience because, classmates, have you actually discussed your experiences with your classmates? Because my roommates are all middle school students, they actually recognize your experience. Have they experienced a similar situation? Almost, that is, because some people I chose at the time were both the basic and the prevention side, and then I just had such a gap, and if some people stayed in the same laboratory at the same time and didn't leave, there might not be this gap. , you should be referring to the research rotation of the sophomore year.

20:27

right. Doesn't everyone have to transfer to 4 laboratories for the sophomore scientific research rotation? It is not mandatory, you can stay in a lab for a year, so some of your roommates may stay in a lab for the entire sophomore year. I pressed the round for 4 rounds at the time, so he does not actually require you to rotate, you can also choose. At the beginning of you, for example, like your roommate, he only stayed in the laboratory. Did you choose things in a two-way process? Or at that time, he told us that we had to have 4 rounds, but some people would tell the teacher privately, after that, because we were a tutor at the time, we would take two or three students at most, if he stayed here forever Then the teacher's wish is that if I only take him with me, other people may not be able to get in.

21:19

Although it is something else, the classmates will be right, I just assume, because I am not sure whether the real situation will have an opinion on this, for example, you are staying at this time, maybe I want to go, I will go No, because I will not report this situation to the management teacher of the middle school class, because after all, the management teacher asked each of us to have 4 labs, but he said this at the beginning, you know someone actually The final choice is still in the two-way choice and the teacher's choice. Yes, if the teacher is not willing to bring one more, you are useless.

21:54

is actually the teacher's final explanation . Sometimes it's because there are 2~3. Some teachers think that it is enough for me to bring two. I don't want the third one. Sometimes this may be the case, so I can say On the surface of management, it requires you to have 33~4 rounds, and we are required to have 4 rounds of 4, but in reality, when we are changing, maybe the instructor just doesn't want to exchange, so I will take him one to the other. I don't want people to come in. Then, in this situation, the teacher in management may not interfere with the choice of this mentor. You can discuss with the teacher, and you can report to the teacher in management. If you coordinate with another teacher, you can, but it seems that in the end, everyone did not coordinate much, and there seems to be no such coordination successful case. Maybe you can coordinate with the teacher, and then go in. I did not coordinate. However, I don't know.

22:48

Like me, there is another one who is more curious. I don’t know if you know it. For example, your classmate or friend stays in that laboratory and stays there forever. .

23:03

Well, from your personal subjective feelings, what do you think is the main reason for him to stay? It is because the mental pressure is relatively small, the funds may be relatively abundant, and the stocking state. He means that the situation you mentioned has strong funds. This is because you think the biggest is the biggest situation because during the rotation, the teacher will only show you. If you are there for a long time, you can really do it. To do some experiments, you can follow some of the subjects of some senior sisters and seniors, and do the subjects together with them.

23:35

Because for our basic medicine, scientific research is still very important. I can't understand that he spent a whole year in this laboratory. In fact, after getting familiar with it, he can follow the brothers and sisters to do it. Experiment, you can learn something. But if another classmate of mine, my 4 labs, this lab just came out after getting familiar with it, maybe they won't take you to do anything.

23:56

You are talking about me. That's how I am, that's how it is for me, it's really been 4 rounds. After 4 rounds, there are not many times that I have actually started to do experiments. Did you think about it when you were taking the rotation, because you should be in the second laboratory, for example, you have found that some classmates have been staying in one laboratory, have you thought that I will also be here Continue to stay in the laboratory, talk to the teacher, etc. Have you ever tried this?

24:23

I told the teacher before, but the teacher said that you still have to rotate, because you are in the second year of the sophomore year. When you really do things, you can't do anything. If you really study experiments, you may not be able to do anything. , How did you feel at the time?

24:39

Actually, it's not very frustrating, it's just okay, because after all, you didn't learn all kinds of things in your second year. In fact, you did something with your mother, that is to say something too straightforward, there is a photo call , you can add anything according to that thing, but if you are not studying basic medicine, you are studying this major, you are not doing a skill, you say that you are not a machine, so you are not Can you say that you still want to learn some scientific research thinking or something?

25:12

I still want to learn some thinking, but for example, when you are discussing with your classmates, you will find that a certain classmate has been in the laboratory, and they spend a lot of time in such a situation. Will not many classmates express to you a necessary positive emotion, such as what have I learned in this laboratory, etc. You have heard them talk about such similar things, according to me I understand that there was a person who stayed in the same laboratory at that time. In fact, there was no boss in that laboratory. He asked him to do a lot of things. He thought that because he stayed there for a long time, he might have more opportunities to do it. , So saying that I don't rotate is actually just saying that the students themselves think that I may have a lot of the above opportunities. In fact, there is no actual operation. It is also divided into laboratories, and some pairs are also divided into laboratories, so it does not necessarily depend on the situation. Everyone thinks that some classmates may have stayed for a long time, similar to you, and have not learned much, while some may have learned a lot.

26:17 Got

it. So this is during your entire sophomore year, and you went to 4 labs. You feel that during the rotation, I still have a question to ask you, and you feel that you are personally right. Is there a lot of interest in the subject of basic medicine itself? Actually, I think I am still very interested.

26:39

So when you are in the sophomore rotation, you will not feel that you want to explore, such as whether I am interested in the topics done in the laboratory, you think you can achieve this goal, for example, after I am in this laboratory, If I go to a general understanding, I can know whether I am interested in what this laboratory does, or whether it can be done, or whether it is suitable for doing. During the cycle, that is, during the cycle, everyone is actually looking to see if you are interested. That's the purpose of the cycle. In fact, the most important purpose of monopoly is to see that you are interested in him, yes.

27:13

Do you think that the rotation has achieved the purpose he originally set? In fact, it can be achieved because when you want to do something in the future, you are interested in it, and when you want to do something here , The understanding of the laboratory is actually quite useful in the process of rotation. Did you have a special interest in a particular field of basic medicine before switching to activities? Or are you talking about immunity and cancer? Because my mother is immune, and my mother had an immune disease before, I was very interested in immunization before.

27:50

When you were later in the Cold War Lab, was it these two directions? There are no two directions in the country. Then the laboratory I am currently working in is the tumor immunology laboratory. Guozhong seems to have visited other classmates before, and it seems that some classmates have been in the immunology laboratory, but he may have immunity, and he may not have much to do with all kinds of things. He doesn't do this lab, they obviously do B cells.

28:13

I know the person you are talking about, the two of us are on the same floor, they actually do B cells, and we actually do tumor immunity.

28:21

Okay. You said and then I will go back to what you just said at the beginning. That thing is the assessment requirements. The assessment requirements for your rotation are unified, but the things you learned during the Cold War may not be the same, so I bring you this at this time. Something that makes you less happy. Is it possible to say that, right? The grades on the rotating assessment are counted as your core courses.

28:51

It is calculated according to the middle school side. In fact, when you are in rotation, you should not have that kind of theoretical study. In life, theoretical study is actually study in the laboratory. There is a period of time to study some Operation, etc. If you operate, you don't look at the results brought by each laboratory in a unified way. What is your assessment? Unified assessment, unified assessment of a certain operation pair.

29:17

, we agreed to test a p3r and then wanted to test the western blot, but there was no test that day, no test behavior or anything, that is, only P plus r was tested in the end. What do you mean by the second one? Y three Bot proteome protein hardware, why did you want to take the test at that time?

29:37

Because in fact, many people have not done western b. If we were to do western blot at the time, I only did one Vicente blot by myself, and it would take at least a whole day on the west floor. , under normal circumstances, it is two days, and our assessment was only half a day at that time, so I can say that it was because most people reported that I had not done website block, so he decided not to test this, and some people had not done PCI at that time, Because some laboratories are doing cells, the content of this test is determined by the teacher who manages the middle school class and the teacher who manages the middle school class.

30:19

Have you reported to the teacher about me or other classmates or have any of your classmates reported this situation, that is, we haven’t learned this thing at all, and some classmates have reported how he responded, But I don't remember much, it's not what you reflected, not me.

30:36

Because I have done both of them, although I have not done much, so in fact, the rotation of the 44 laboratories in the sophomore year, whether these 4 laboratories have a good impression on you or not, the experience is different. ? Aside from the assessment, it's just a simple rotation. If there is no such thing as a whole process, you can ignore the process. pretty good. There is nothing that impresses you deeply, happy or unhappy, sophomore year not for the time being. By junior year you will have it, right? In fact, the assessment of the sophomore year is to bring you a little dissatisfaction, but it is actually okay, because in the end, everyone has passed this contract for me . , including the things submitted by yourself, as well as the evaluation given by the teacher, the evaluation of the general teacher, after all, the evaluation given by the teacher is actually acceptable.

31:41

But very subjective. Yes, what the teacher gives is really subjective. Therefore, some students may have high scores, and you or other students around you may not recognize this high score.

31:55

Yes, because some of the evaluations you give by the teacher will have a lot to do with the laboratory. If you have little relationship with the members of the laboratory, if you don't have a good relationship or feel very unfamiliar, because they will ask you about the inside of the laboratory. People, some labs will grade not only the teachers themselves, but also the teaching situation of their graduate students, or the impression that the graduate students have on the students. In this case, the grades will be based on these. , I brought a graduate student, you are here for rotation, I will ask the graduate student how this person is, and then based on his influence on me, I may judge based on the scores of the surrounding classmates, you think this may be An influencing factor, because this grade is very subjective and is only given by the teacher.

32:44

And in general, are you taught by graduate students in the lab? Sometimes it is taught by graduate students. Usually, if you go to the teacher to sign a letter or chat about some situations a week, the teacher will know very little about you, and they are all taught by postgraduates. Can I say that you have very limited opportunities to get in touch with teachers in the laboratory rotation, and you can almost get in touch, but I just say that there are not so many, and the main thing is to get along.

33:12

Yes, it is mainly because there are more seniors and seniors than those graduate students.

33:16

Do you think that from your perception, it is your personal situation, or do you think that the students around you are in this situation? In fact, everyone has a lot of contact with senior brothers and sisters, and not much contact with teachers, so in the end The situation may be that the teacher may ask about the influence of the graduate students on you when grading, because the teacher does not know you very well. Tell me about your junior year.

33:40 In

my junior year, I will first talk about why I wanted to retire or not when I was a junior, because after my sophomore year, the middle school suddenly started an innovative vision plan similar to basic medicine, and asked us to do that. This kind of small project research, after we formed a team, they asked me to be the team leader, because the team had already been formed at that time, and then the project had been selected.

34:07

Why did they ask you to be the team leader so you can learn about the recommendation, because I was in the right place at the time, and I was looking for my lab teacher, and then they went to my place together. Uh oh. So if you choose this head, you can't back out at that time. Then I wanted to make a little bit of this thing in my junior year, and then let them do it later, and I retired. you just quit. Yes, because I wanted to retire when I was a junior. Why do you want to quit? Is it because of various laboratories, and another reason is that after the rotation of the sophomore year, I found that I am not very interested in this area, and I still want to do tumor and immunization.

34:58

At that time, you said that you had gone to the middle school to find out. You thought his development situation was also very good. You didn't realize that tumors and immunity had little to do with reproduction? You can put it another way, because I didn’t switch to the clinic at the time, and wanted to use the springboard to switch to the basics, because this was an opportunity to switch majors for the second time.

35:21

You can understand why I was thinking about leaving. Vitality right. Just the basics. Would it be possible to say that technology might bring you closer to the tumor or yes. Because many of us had two choices at that time, one for prevention and the other for basics. If you quit prevention, if I quit from here and I do prevention, I can still rely on my practicing certificate or what to do in the future, but in the end I still Choose the foundation, the foundation can't consider these things, the foundation can't.

36:01 The

basics are mainly still doing scientific research, and the basics are still doing scientific research. When you were a junior, you said that you made an innovative plan. You became a team leader and formed a team. Did they choose you because they thought you were in the same direction as their team, so they chose you to be a team? long.

36:26

Yes, we didn't disclose to you what we wanted to do at the time. Yes, I didn't disclose it to you. After all, I didn't understand some mentors. In fact, can we say that it actually affects your withdrawal? The biggest one is the sophomore year. The experience of the scientific research rotation in these research laboratories is not very good, so to speak, but the junior year actually did not bring you some bad experience, that is, from the last semester of the junior year, I think In fact, it was the third year that had a big impact on me.

37:00

What is that? It was because we did the right thing at the time, and we had already opened the questions before we started, and then we really started to do research on a small topic in this area.

37:11

But at that time, the teacher I was instructing never let us carry out the work, and even though he gave us guidance in name, he always let us go on our own after every time we went to talk to him. I read the literature and found some things myself, but there is no substantive guidance, which makes me think that I still have the cell experiments or some experiments I mentioned at the time. Our experiments need to use these technologies, but he told us Say, after you really start, the time to actually do these experimental studies will be very short.

37:47

The time it takes for you to learn technology is very short, so you are not in a hurry now, but it has been dragged on until the epidemic, and we have not really started experiments. Then we have one year, it will take two years to complete it, and then we need to conduct a mid-term assessment in July last year. After the end of the year, we will have a mid-term assessment. Think about what we did in February last year. Have not developed, still in a state of 0 .

38:18

So it had a big impact on me. I felt that during the mid-term assessment, in fact, you still had nothing, nothing at all.

38:31

So it has a great influence on you, but for me, I feel that when I study in the laboratory, the things I actually learn are always the things I have learned before, and then after the innovation plan is launched, I will go back to the teacher. There are some things we need to learn, he has been telling us about what you really started to learn later, and what you can learn can actually be mastered very quickly, but he has been dragging us back like this, not allowing us to learn.

39:01

Then I was in the same lab as me, a team member was in the same lab as me, and the two of us were in the same lab, and then because of this situation, the two of us quit in the middle. Yes, because it never goes on. Then the teacher has not let us continue, so I want to know about your innovation plan, is it all the students in the middle school class to participate? Or self-organized?

39:29

At that time, it was said that everyone should try to form a team to participate, try to form a team, but I do not force you, but in fact everyone left. In fact, there were actually a few people who didn't form a team at the beginning, but when the second middle school class started to form this group, almost all of the 2017 class had formed a team, um, almost all of them.

39:49 If

there is no team, the teacher will give them a small task and let them do it by themselves. Because some lab teachers won't give them their own small projects for you to do, you have to fight for a small project. So can I say that those who didn't form a team are still because the teacher gave a small topic, not the teacher gave a small topic to the teacher.

40:09

Okay, for example, your innovation plan, you formed a team, where is the source of funding for the project? 30,000 yuan for fruit seeding, 30,000 yuan for each subject, yes, 30,000 yuan for each subject in the middle school, and then an additional 30,000 yuan after passing the mid-term assessment, and an additional 30,000 yuan for excellent mid-term assessment. Did you communicate with this teacher yourself? Yes, he did not assign it after the communication at that time, and then asked us to form a team as much as possible, and then the teacher needs to contact by ourselves. In fact, many teachers here are reluctant to take this kind of college student to start a business, this kind of entrepreneurial innovation, because why do you feel?

40:51

Because I think some teachers may be new, they don't have such experience, and some think that you may not be able to publish any articles with these, and they are not very useful to them. Therefore, from the perspective of the feelings of the classmates around you, most teachers and your innovative plans may all be vigorous and vigorous, but if you are looking for a teacher, many teachers may not be very right, and they are not very willing to think about a very common one. Is there any kind of team that can't find a teacher?

41:27

That's not true. If you can't find it, go directly to the person in charge, and then which teacher you want to find, the person in charge may help you coordinate it, but it's really impossible for you to change the teacher. So there are quite a few. The classmates formed a team and asked the person in charge to help you coordinate it. Yes, if you want to ask the teacher to help you coordinate, did you find the teacher yourself or coordinate it?

41:48

Did he find it himself? I asked at that time, agreed with us and said our thoughts, and asked the teacher if you would like it? The teacher said yes. Then you can. Then I started to prepare a series of materials. You said that after the question is opened, do you want to answer the question? Yes, the question must be defended, that is, in the middle school class. At that time, you said that the teacher would not allow the question to be opened. You are not the teacher today, but it is because the teacher did not allow you to do it later based on what you asked. The question is just a theoretical thing, ah, the question does not need the teacher's approval. You yourself are in charge of the activity class and they will find a teacher to examine it for you.

42:29

We will answer. We will answer and then answer, and then we can open. After opening, if we get to the teacher's side, there may be no progress. There is no progress. Every time you look for him, what's the reason he won't let him go? Is it that your literature research is not enough? still? Yes, he thinks that we don't have what we have done and what we have recently read. He does not support what we can do and cannot support what we can do. Is this the reason every time? So he refused you to continue to go down. This is, um, a few times, we usually look for him once every two weeks or so, once every two weeks, and it's like this for a year.

43:26

In fact, it almost disappeared during the epidemic. In the first semester, December of a semester is not counted, because the exam is in December.

43:34

It doesn't count during the exam, and then from September to November around the end of November, every time he tells you this question, right? Every time he says that his various reasons prevent us from developing, the reason that impresses you the most is the problem of the literature, which impresses me the most. Actually, he said that the literature we have read does not support it. He wants to give you some knowledge, that is, the guidance you should give us, let us read this article, then read an article, and finally come and share our thoughts with him after reading, he still thinks that we Not feasible.

44:15

You are alone or privately or other team members communicate with the teacher privately. After he refuses to communicate, the teacher will give you substantive guidance, and we did not have any substantive theory at that time. It's equivalent to what you said about substantively knowing that it's another group, right? What other groups do you think are some of the knowledgeable guidance that might be considered substantive guidance? What are they going to do, yes. Shows you exactly what you're going to do.

44:46 In the

first step, what article do you want to read? In the second step, you have to write something or do something. Teachers in other groups can do this. When you communicate with your classmates, you are actually like me. Can you say that your group is a very special group, because other students may not have encountered a situation like yours. In fact, after another group, we have been opening the question for almost a year, and there is no result, no Too much progress, but they still have a little progress, as long as he doesn't have pre-experiment results , we don't even have pre-experiment results.

45:25

They're as slow as you are, they have team members drop out of the situation. Your teacher was taking you into an innovation group at that time, because you can only take one teacher if you have a teacher, and a good teacher can only take one, right. So from the opening of the question to the later epidemic, and then including to June, it is to return to school, and return to school in May and June. Have you looked for him when you return to school in five or six years? It's the same after looking for it. After looking for it, he will go to us to change the subject. Just change the direction, yes. What is the reason? The reason is that he felt that what we had thought before would not help him in any way. I don't feel that way about him.

46:11 It

didn't help his lab in any way , and then let us do a lab that was somewhat similar to his lab. Although what we did at the time was related to his lab, he said it didn't help the client, and then he Let's make one that is actually related to him, just to build two intelligences, and then he wants to use the two of us as a graduation project for me and another classmate, so he actually wants to give you a direction. , follow his direction.

46:41

Then the final purpose is to let us stay with him. If you think you will stay for three years from the third, fourth, and fifth years, you will only do one graduation project on his side. He finally pointed out this to us. look like.

46:55

Have any of you talked to him privately or later, on WeChat or by phone, you were not satisfied with the direction we mentioned at the beginning, why didn't you talk about it at the beginning? Has anyone raised this point?

47:08

No one has really asked him this question, yes, I really don't dare. Then it is really equivalent to being there for about a year, so it is equivalent to when the mid-term assessment is about to take place in the third year of junior high school, and the instructor proposes to change your direction, and then in fact, this point makes you unacceptable, yes . While everyone else was moving forward, the other team member and I stayed where we were.

47:39

So after the epidemic, for example, there will be only one other member of your group left in the semester. Yes, it was originally 4 people who knew about it.

47:50 Did

the classmates who launched like the other two join other groups? Or is there one you've already interviewed? I interviewed a lot of people, and I didn't know I was immune. Did you quit? Immunity to quitting quitting when I wasn't there. After he also quit that group, he quit the middle school directly. You think he also thinks the same as you, isn't it, he mainly wants to do public opinion. In fact, I actually have something in common with him.

48:21

Maybe Jelly or even this direction may not be too big for him. right. But I remember that because after the interview, I asked that the interview was another interview with a girl just now. She said that she prefers to go to another school for postgraduate research, but the postgraduate postgraduate in middle school must be in the school, so he wants to pass the ordinary postgraduate postgraduate method. , took the post of guarantee research to export, but now he still wants to guarantee the school, and also wants to guarantee the proofreading.

48:47

The middle school promised us all the research at the beginning, but now there is no result, and it has been changing. So this also makes a lot of people unhappy. Actually, I am also a little unhappy, because the guarantee given to everyone at the beginning is that as long as you don't fail the course, you will receive a full scholarship of 10,000 yuan each year, and then all 30 of the class will be guaranteed. Research, and then you will not be restricted from bail. The initial preaching was like this. You are very impressed by this, right?

49:20

Because I have a deep impression of Baoyan, can I say that the policy of Baoyan has a lot of confidence in you, yes. The same can be said.

49:30

Do you think the basic major has a good prospect, and the former and the latter do not have much impact on me, or the prospect will be more important to your psychology. Then, when I was preaching at the beginning, I said that it is possible to change the extroversion gradually into a joke. Has anyone around you ever thought about quitting because this is something of Baoyan? Have. Did anyone actually quit? It's just that some ideas are not there yet, isn't it to be immune to that? Isn't it really going back? In fact, it was launched mainly because of the policy of guaranteeing research and development. In fact, he was not very interested in promotion.

50:15

Another student in your group withdrew from your group and pushed out another classmate who had not pushed out. He never quit, he went to another laboratory, and he was in another laboratory. I can think of your innovative team, most of the plans are relatively smooth, or it is still quite a lot like yours who stopped in the mid-term.

50:39

In fact, the rest of the labs are going smoothly. If the teacher has been guiding you and letting you advance, it will be quite smooth. You have communicated with other students in other groups. What is the status of an innovation program? My roommates have shared their feelings with them. What is your deepest impression of their feelings? Although they have difficulties, they can still move forward. Although they have difficulties, they can still move forward. What is the difficulty?

51:10

I can't go on, or it's not that I can't go on. At this point, it's a little bit impossible to go down, but with some guidance or some change of direction, or some way of changing, they can still go down, maybe they themselves do it. The experiment didn't go well, but the teacher's guidance was still right, and they could still go down.

51:31

Maybe their difficulties are not the same as yours. Your teacher may not know much about the guidance, and they may not be able to do experiments on their own, which has nothing to do with the teacher. Have you communicated with several groups of students? It is this innovation that actually communicates most in one group, because it is convenient. Therefore, the innovation plan did not continue to advance, which brought you a relatively large sense of frustration. It can be said that. So you are just starting to doubt, I am here, I am not suitable for me and I am not suitable to stay here, that is to say, if I continue to stay in China, I may continue to work here in this laboratory. , he also gave me the direction maybe what I learned or what I can get, there are too few things, or I can't get what I want, yes.

52:32

You can understand that. I'm just making a hypothesis. After you enter the laboratory, is it possible to quit this experimental project and join another group. Have you ever thought about it? I have thought about it, but because I was a group at the time Long, I can't, I can't quit when my team members have not quit, but when they quit completely.

52:54

Did you discuss this idea with the team at the time? Maybe the two of us will go to another group together. We can't push the two of them at all, so now we and I have withdrawn Mr. Guo, and he has withdrawn from the laboratory. In the end, there are only two people left. The team members will go after the laboratory is pushed. If he went to another laboratory, he went to another laboratory, which is equivalent to going to another group.

53:20 Right

. When you quit at the time, you wanted to talk to the team members. You said that the two of us had negotiated something. I mean that because the two of us may have said a little too much, it has already been delayed for a year, so he does not want to continue to spend time here. go down. After discussing it for a while, he said he was still in the pot, and I said I just quit. Did he stay in the circle? You are not with me. Not so. Because we were all rotating at the time, he went back to the lab he had rotated before, how many times did he rotate? three.

At 54:01

, he went back to the original man's laboratory. There was an innovation plan, so he didn't continue to do the project. What is he doing now? There was no subject given to him at that time. He followed his brothers and sisters and they asked him to do what he did, so he actually did not participate in any project plans now. Can I say that he doesn't have any plans at level 7 now? That's not necessarily true. In fact, after I quit there, I didn't know much about it, so you don't know much about what happened next to him. I understand, I don't understand the latter. OK You have thought about transferring to another group, but you may have considered that the team members should not transfer directly, and then you want to quit directly.

54:47

I don't. I've been thinking that it's a big deal and just retire. In fact, my struggle is during the epidemic. I have been struggling for half a year. During the epidemic, I began to struggle. Did your parents discuss this matter when you were struggling? discussed. They say what they say, just be happy. They've been as long as I don't do anything morally wrong, and I'm sure I'll be fine. Good friends and things like that have been discussed. You are happy, yes, I am really happy.

55:17

So can it actually be said that it makes you unhappy that the junior year was delayed for a year? It can be said that in the second year, the assessment requirements are not very clear and transparent, and the subjectivity is too strong, which may make you a little unhappy, but it is not enough to talk about what to think about it, but after the third year, you feel very unhappy to know.

55:42

When you were struggling, did you have any discussions with your classmates who are better roommates in the current active class? They said that you wanted to discuss it with me. They also said that if you really can’t believe it, you If you still want to do cancer or want to do immunization, you can still try it. Although it is very late, after you quit, the requirements for undergraduates are not too high. After you quit, although you have nothing, but you have learning experience, you can go to a new laboratory to study.

56:09

I would like to ask if your team innovation plan had anything to do with tumor immunity? It doesn't matter, because Guozhong has nothing to do with tumors and nothing to do immunization, and the teacher is not very willing to wear a big bed. You just said that you are actually more interested and have made an innovative plan. I would like to mention a hypothesis and mention the entrepreneurial plan. You said that your interests are not very coincident.

56:35

Yes, because but there is also overlap, because the tumor also has a little bit, but if it can progress smoothly, if this teacher gives you a lot of substantive guidance like the teachers in other groups, in fact, you You can also continue to do it. You are still willing to stay in the country to do this thing. If you are willing to stay in the country, you may not be willing. Why is it because you may have overlooked a point before, I said at the time, in fact When I was in my junior year, I wanted to retire, but because of the formation of a team, I thought that if this matter can really be pushed forward, I would retire myself at that time, and there would be room for me to retire, because You can recommend everything, and it doesn't matter if you change the team leader in the end, because you can continue to go down on this matter, but when you can't go down, it's not appropriate for me to retire.

57:31

Therefore, the idea of retire in your junior year mainly comes from your sophomore year. It was an unfair assessment method at that time. Can you say that? It’s not just, it’s not just an unfair assessment method, that is, something will suddenly come up in the event’s total result to make you stand up, and it’s not counted as immediate implementation, that is, within a very short time limit, you will It suddenly feels like you are caught off guard, just like when you suddenly set up this topic, you are suddenly caught off guard, you will be notified in June, and you will have to defend in July.

58:11

Of course, there is such a thing that the tasks assigned by you suddenly have to be completed in a short period of time. In fact, the requirements for the initial assessment were written for you suddenly at that time. The class informs you that you are going to do a small project, and then suddenly informs you. After the notification, before the first exam, if you are suddenly notified of this assessment before the first defense, all kinds of notifications will catch you by surprise. The notice is mainly related to teaching, whether there are other such as practice-related, it will also make you feel less happy.

58:51 No

package? Mainly the exam.

58:53 The

teacher suddenly assigns you a task, or suddenly wants to say something to test, right? Suddenly you are caught off guard. In fact, at the beginning of the school year, it is better to make it clear to you, what should be tested and what should not be tested.

59:09

Yes, it suddenly caught you off guard, like this experience made you unhappy. Do you feel the same way as your classmates? Have you ever complained to them? We've all complained to each other about this. They also feel comparative. So can I say that these two points constitute that you are earning a living, and the idea of quitting can be a sudden surprise assessment work and not-so-transparent assessment requirements. It can be said that there is no other. Whoever said there is no more, there is no more now. Not for the time being, not for the time being.

59:52

Can I talk about the basics, that is, it brings you closer to your point of interest. But in fact, after you came in, your interest in this class was just a little bit bordered by you, not completely overlapping, right? Then there are some things that let you, including assessment, make you feel a lot of unhappy emotions, so you are more determined to launch an idea to make a living.

01:00:26

Yes, how are you feeling in basic medicine now? I think I am very happy. Although I am a little tired sometimes, I think I am very happy. What do you mean by tired? Tired?

01:00:36 It’s

not that I’m tired. Sometimes I can’t say I’m tired. Sometimes I want to learn a lot, and I have a tight schedule. Then I feel a little tired, but I think I’m quite happy, and I have a tight schedule. , indicating that you have arranged some innovative plans of your own arrangements that you have arranged yourself? Or is it an experiment? No, it’s just that some nature is related to nature. Think about learning some things related to nature, and then shoot yourself. Do you go to the teacher to learn from him? Are there any online classes or online classes or something else?

01:01:23

Another one, I would like to ask you, do you feel that not only the junior high school class, but also the one thing you feel most proud of doing since you went to college? Just the best thing to do. Um, the most sense of accomplishment, you can say anything if you can’t say it, and you can leave it if you don’t think there is anything. The final sense of accomplishment is that I went to volunteer teaching in the summer vacation of my freshman year, and I went to complete one of my high school medicines. When I was in high school, I wanted to volunteer, yes.

01:02:03

Where is it? I am in Xuzhou, Xuzhou. Is this called teaching science in rural areas? What I gave them at that time was the kind of medical knowledge related to medicine. Some small cities did not have summer vacations, but they were still okay. It's still possible. Equivalent at the end of the semester or at the beginning of the semester. Not summer vacation is summer vacation. Did you have any influence on your desire to be a volunteer teacher at the time? I still think about a wish of mine back then, and I have always wanted to have a wish of mine back then. I didn’t say anything, there was nothing, it was my wish back then.

01:02:49

After entering the middle school class after the sophomore year, did any junior or senior come to ask you about Wang Yuguo? Have. Do you remember what questions they asked? They asked the most impressed or asked the most. Can you do research? Can you do research? Can you bail out? Can you do research? Can you bail out? At that time, the policy at that time should be that it is possible to guarantee research and guarantee. You said so, and you also recommended them to come. I didn't recommend it at the time. I just gave him a rational opinion at the time, that is, what to let them decide for themselves, because at that time, I actually asked me after I quit, and someone asked me. , why did you quit?

01:03:42

Some people came to ask me, and some students from the 20th grade also came to ask me how to take the exam, you will tell them, yes, I just tell them what you mean by rational view , you can tell me roughly what you can learn when you come to China, but what restrictions will you have?

01:04:05

In the middle school, there are actually quite a lot of restrictions. When we entered the middle school, we were asked not to be members of the student union of any other school. Internal positions were not allowed. Yes, then the school capital is it not OK? All kinds of positions in the student union should be avoided as much as possible, including clubs, but I still did it secretly back then.

01:04:31 As

the president of a club, theoretically he doesn't allow you to do it. In theory, it doesn't allow us to have any knowledge. Then what is his reason? Does he have a reason, does he still concentrate on scientific research, and then theoretically requires us to other colleges, you say that we don't have to participate, or we can try our best, and then we participate in our own projects, right Participate in programs within your own middle school, don't participate in those things, right?

01:05:02

Do you have any projects for your sophomore year? to allow you to participate. Well, what else is there in the travel plan? Didn't he have a plan for not letting you participate? At that time, I was talking about the middle school class. Since he didn't let you participate in the outside world, he would have it himself, right? No. Only that subject is only released at the end of the second semester of the sophomore year, so it is equivalent to the sophomore year that you may want to participate in those physical programs, but Daiso cannot. At that time, I still participated in a training activity in a winter vacation of our college at that time, which was a training activity of the basic medical school.

01:05:37

In fact, what kind of training and what kind of training in the winter vacation is a winter vacation class in the tourism plan. Those students who participate in the training will learn how to participate in the city plan. The training of this kind of thing is not the kind of listening to lectures and then doing experiments. It's like planning a guided lecture or something.

01:05:55 Right

. Because it was the summer vacation at that time, he couldn't restrict me and the winter vacation when I was a sophomore. After he issued these regulations, did you complain about these things to the classmates in the middle school? I can’t join the student union, clubs, city plans or big innovations. Actually, I’m not complaining, because I’ve secretly started clubs, and I’ve never been there for myself. I’m in front of you. It can be considered a rant. They complain more about the student union, or are they unable to participate in other college programs?

01:06:28

Some people have done it, Minister, and then you asked him to retire directly. He was actually very unwilling, not very unwilling.

01:06:35

You are like if now he says that he can't guarantee the postgraduate study, you need the top 40% to be able to post the postgraduate study. If you are like that, if you take the postgraduate entrance examination, these are also a bright spot for you to add points, and then you suddenly quit , and then prove that there are no proofs of any kind.

01:06:50

Some people really retire just after giving birth to the minister. You will tell them about these things. I will tell them that it is very restrictive. You will tell them what you have learned. . I will tell them, because I can actually learn something. What do you think is your biggest takeaway? The biggest gain of the second and third year in the past two years is really because the nursing has learned about basic medicine before, but only got some etiquette on the surface. Does the area mean the theory? Do you really know from the beginning?

01:07:32 In

the end, it's not even a real understanding, even more, if you understand, you will understand. Therefore, it is generally like when they ask you, er, junior and senior students, you mainly talk about these three aspects, analyze them rationally, and cannot bring my own subjectivity to them, that is, you rationally analyze the advantages and disadvantages of it, You decide for yourself whether you want to come or not. Yes, so you talk to them about these advantages and disadvantages, mainly these three points, right? One advantage is that you can really learn some internal logic knowledge, and there is a more practical thing, a scholarship of 10,000 yuan a year.

01:08:07

Well, yes, there are scholarships that we won't let us judge. Those who judge the college can only get his scholarship. Will the college scholarship be more than the 10,000 yuan? Not much, but you can't comment on your academy, and you can't comment on your other Yang Zijiang stuff. You can't judge the principal either, the principal or something, and you can only take his bonus. So in fact, for some students, it is possible for him to get more scholarships than you.

01:08:40

So it actually looks good on the surface, but in fact there is a little bit of beauty on the surface. You will also tell the news to the juniors and juniors. Pay attention to this. Would I tell them they would care? It is a scholarship, and some people will come for a scholarship.

01:08:54

Some people are like you because when you transferred this, it seemed that you were required to be in the top 50%. If you are in the top 50%, many people are in the top 50%. In some colleges, you can only The top 50% of some colleges get a scholarship of 300 yuan. For example, I am more curious. Like they come for those scholarships, maybe 50% can only get a scholarship of a few hundred yuan. This time I get 10,000 yuan. Did they waver after you told him about the restrictions? Or?

01:09:25

I don't know this, you don't know I don't know me. You only told them, the judgment is up to them, I just told them the fact. If you have an education, you said that you just said you have an education, did you ask you why you quit? You have. Tell him truthfully, just say what I told him is that I'm not interested, and I think I'm staying here, maybe I can't adapt to him, I can't let him adapt, I can't adapt to him , and then I'm not happy, I'm not happy here.

01:09:55

Don't they ask you what's unhappy about or what's not interesting to you? Or are they not asking? They are closed. You also didn't tell them that this teacher won't let you advance. The topic of Cca has actually been said sweetly. I said it once, but I didn't tell them in depth. Maybe when you advance your entrepreneurial plan, it may not be as smooth as you think, and you will also talk to your juniors. The schoolgirl talks about this.

01:10:26

All the level 20 people came to you for whatever you said, didn't they? Because you are the first to quit? It's not the first one to quit, because my grades were in the second level of the written test , and then when I launched it back then, many people were surprised, but not a lot of people were surprised, many people actually thought it was normal, I quit the country The students in the middle class can already feel that you are a lot unhappy with their classmates . At least the friend around me is my roommate, and because he is the foundation and prevention, many basics can also feel my inconvenience. Happy, because we are not familiar with the other side, although we are in a virtual class and not at the same level, we will only be together during meetings, so they can actually feel that you are already a lot unhappy very early, so in the end The moment you quit, they weren't actually very surprised or very clear.

01:11:34

Do you think there is anything in the middle school class that makes you feel very stressed? Or in the process of studying, or in the process of scientific research, in fact, when you go to the junior high school, you will draw the drawings for you, the kind of scholarship he gave you, and the scholarship that he gave you at the beginning. Putting you in a cage makes you feel that you are not under any pressure, but when his policies keep changing later, it will put you under a lot of pressure.

01:12:03

Because what you said is very realistic, all kinds of research and research things, you have been here for a long time, you have nothing, you have no capital to use to compete with others, because each Like restrictions, I don’t have any capital to take out. Maybe my student activities, my other research projects or teaching support projects, because I came to your project, I have no way to participate. You promised that I could keep it. Research, and then not necessarily when.

01:12:31

In fact, I have lost a lot. Yes, if you want to study in the main school of Southern Medical University, these are not really things, but in fact, many people want to bail or have another request. At that time, we were on summer vacation. Because for undergraduates, how many activities are required for you to graduate, what activities should you have in winter and summer vacations, activities of three trips to the countryside or other activities can graduate to the second class.

01:12:55 Regarding

the second class, he would not let us go to another second class, but let us go to the middle school class. What is the second class in your middle school? The laboratory is the laboratory study in the summer vacation. The summer vacation of the sophomore year is the same as the laboratory study of the summer vacation of the second year. As long as you study in the laboratory during the summer vacation of the sophomore year, can you complete the second class? No, the second class is 4 or 3, so I have to do it every summer vacation.

01:13:20

Actually, that's right, and that is to say, can I say that you can't practice those other second class points of the Youth League Committee? He is asking you to cultivate yourself, and that is not a requirement that we must cultivate yourself. Try to ask not to go out to participate in activities or something. He will often ask you, but he will not say to stop you, but if you don’t go to the laboratory, you will definitely have a bad impression with the teacher, and the teacher will feel that the impression is not good. If you have a bad impression, you are subjective. Score is not low.

01:13:46

But it will indirectly affect your final exam scores, but when I was with you, did you actually have an argument for the second class during the winter vacation in the middle of the sophomore year? My sophomore summer vacation was too heavy. , I left to teach in the summer vacation of my freshman year, so I only have it in the summer vacation, not in the winter vacation.

01:14:06

Yes, there is no winter vacation, because the winter vacation time is too short. Then there is the second class. In fact, it is not very willing to let us participate in the usual activities, but I was a sneaky person. If you don't let me do it, I may still do it. I just do it sneakily. . Because after all, I was the president of a certain club back then, and you think it would be a pity to quit this club. He didn't quit either. In fact, it would be a pity if I asked him to quit.

01:14:40

You said it will affect the subjective score. I have a point that I don't quite understand. At that time, the sophomore year had two semesters to take exams. You said that the score assessment in those two semesters was more subjective, but the support teaching is like the second class, which is the support teaching. It's only available during the summer vacation, so it doesn't actually have a big impact on your sophomore grades.

01:15:04

Another point is that our score is based on your attendance. At the beginning, we used 5 times a week. Later, because it was really difficult to meet the standard 5 times, we changed it to three times a week.

01:15:19

Is the rotation of the sophomore year? Yes, in the lab. Your boss will see you at least three times a week. You're not saying that the boss usually asks graduate students to take you, yes, but the boss may be going around, yes. We had strict attendance at that time, who would take the test? The boss deducts it himself. The boss comes around and tick it in a circle. You write on it, and then you go to the boss to sign it. The boss's attendance actually accounts for the majority, right? So like the second class, if you don't want to participate in his summer experiment, then he is not very willing to let you participate in the second class project outside the middle school, so will it affect your graduation?

01:16:13

is not. If it is, it is impossible, because he will arrange a laboratory for you if you can't pass. You can see that if you have a laboratory, this is not a project. If you have a project, you can graduate. The main reason is that we will require you to stay in the laboratory during the summer vacation in the laboratory. If you stay in the laboratory, you will not have time to do other places, and there may be a conflict in terms of time, so most shareholders Do the students in the class stay in the summer vacation?

01:16:41

Yes, I stayed until August 20th that year. What do the classmates around you and yourself think? What idea? Don't do this summer software, go out to do it quietly, and there are people who are interested in doing it.

01:17:00

He didn't participate in the summer vacation, and he also participated in the summer vacation. He didn't need to teach the total number. He took it because some of them only took three, three or four days. Are you done? You didn't come for three or four days, and the school didn't have a full vacation at that time. You could say that you didn't have a full vacation, and you went to do that stuff. You can do it for a short period of time, but you can’t do it for a long time, but not for seven or eight days. You can do whatever you want for three or four days. So in fact, can I say that I actually want to do takeout, there are still quite a lot of students who are not related to scientific research.

01:17:38 It

can be said because, but it also requires you to do the second class in scientific research. In fact, there are quite a lot of restrictions on you. In fact, some people did not do that, nor the second one. The second is because at that time, I directly told the teacher that I would not participate in the familiar conclusion, and it was not because we were required to stay in the fourth middle school for at least the summer vacation, and we were required to have at least 4 weeks, and some people might not bring these 4 Week or take 3 weeks, and then do something else, something like this can be understood.

01:18:12 Got

it. Mmmmmmm. I don't really have anything else. Thank you very much for your cooperation. OK Let's see if I missed something. OK Nothing else. Just wait for me for a while. It's alright. Let it go, I'll come and say goodbye. Bye-Bye.
