# Supplementary material for: Why do you choose this program?—A decision-making model of medical students based on grounded theory
Source: PLoS One. 2023 Sep 15;18(9):e0291634. doi: 10.1371/journal.pone.0291634 (PMC10503722; doi:10.1371/journal.pone.0291634)
Supplement: S1 File — (ZIP) [file pone.0291634.s001.zip › RAW DATA/P11 CHINESE.docx]

05月21日_1.wav

00:01

我要签道德伦理需求，本次访谈中受访者是在平等自愿的原则上参与的，受访者必须真实表达自我的、想法和认知，确认自己符合受访条件，访谈的过程会被录音，录音资料将以匿名的形式用于科研，不会泄露给任何第三方，在访谈的过程中和访谈结束后，你都有权取消录呃研究人员录音资料使用权，你是否知晓并同意？同意。

00:32

好的，然后可以先问一下你的年级和专业看一下。大四了有信息基础医学专业的，2017级基础医学基础医学嗯。我们问的第一个问题，想问一问你在大一的那个时候，在报名国中班之前，你是读了国中班又退出了，是的是的，你在报名国中班之前，对于国中班的了解有哪些以及怎么了解的？大一现在是这样的，因为大一之前的时候那个时候确实有消息，包括我们的，辅导员，包括全国办他放出消息是国甚至于国家重点实验室要开一个股东班，而且当时跟我们的宣传是主要针对于基础医学和公共卫生医学，就是预防医学，是主要针对两这两个专业的这两个方向的同学去，去在实验室里面做基础科研，因为大一刚进去，其实对我们自己的专业基础医学也不是那么的了解。

01:31

然后包括大一下的时候，大一下到后面就是三小好老师后面开了一个讲座，专门来介绍，包括他们的国家重点城市和包括开创的创新班就是活动班。我们根据这样的一些消息渠道，然后去了解他们，然后后面然后当然宣传的也非常的好，待遇非常的好，当时就跟我们说，进去了之后大家都可以保研，你知道吗？大家比还是比较关心以后的就是去向问题，包括保研，包括他奖学金的待遇也蛮不错的。

02:06

然后而且他对于这种入围的这种要求其实不是那么的高，所以说后面然后就去报名了过后，你当时成绩的是大概什么位置？当时成绩是年薪是35%左右，因为当时这个名字比较尴尬。

02:26

这就还涉及到一个当时为什么选择股东班，轻微大一下的时候，其实技术学校同学当年我们刚建的时候，我们随着学习，然后和我们去学工办的了解，我们发现技术学在我认为包括现在的同学是一样的，其实在医学医学院校是感觉不是那么受重视，因为大家刚进来的时候，大家都本身都是去当临床医生，我们要想上临床去做手术去治病救人的。

02:53

但是后面然后被迫可能大部分人都是因为调剂来了基础医学，但然后带一下学校不有什么专业，然后大部分同学都是大一的时候拼命的学习，就是为了转专业，从技术学离开再去往临床医学，这是我们本就是我们的目的。

03:08

但是因为后面转专业是有是有成绩限制的，前30% 20%是临床，30%可以去儿科或者是去精神类的。

03:20

当时当年我也是想的想好好学习，然后去到临床也想治病救人他一个医生，然后后面这不是考试成绩不是太理想，就30%我记得很清楚，离入围离去，参加这种临床转专业的入围还差个一米还是两米。

03:37

当时当年国中班的他这种报名的要求是前50%，在我实际上很清楚了，我正好就是处于这两个的中间，所以去报名的过程。我比较想知道一下，你高中时候报志愿的时候，那个时候你是学理科的对吧对？我是学理科的，你当时报报专业报学校的时候，你是理科、工科、医科都报了医科。都报了的，因为当时很尴尬，我高中的时候其实没有考虑到太多高中。

04:08

结束了之后，因为考的也不是那么明显，我本身的就是成绩发挥要稍微是长一点，然后填志愿就是不知道该怎么填，然后就问了谁，我们自己和或者自己我就自己和父亲商量的，因为我是四川的考生嗯，大概当时高考成绩大概在四川排7000名左右，就是四川省7000名。

04:31

然后当时我爸问我的意向首先要去哪个城市，我们首先是先选城市，然后我想的是当时不留在四川，确实当时是可以去川大的成绩。我说我不想去穿了，我说我不想留在市场上。觉得川大很一般，然后后面我想去想买东西，觉得川大一般还是觉得我觉得成都很觉得川大很遗憾，四川人可能当时因为平时成绩也蛮好的，觉得看不太看得上川大，然后是因为很多人都可以上到成对，因为我当时读的是比较好的高中，而且是高高中里面最好的，我们平均水平基本上都是在传达出来。

05:06

后面然后我就想着来东南沿海这边的城市看一下，然后南京上海或者北京，后面发现当时高考确实考得不是很理想，没有原来我能看得上的那几个学校我都考不上，包括南大包括统计上交这种都考不上，感觉差很远。

05:23

然后后面，然后我把我你喜欢什么，根据你喜欢的这种意向我们再去填，不好意思打断一下，假如说你能上同济交大什么南大之类，你会报什么专业考虑过，我就是从来没有考虑过考虑，对现在考虑学校对。

05:38

然后后面确实是因为学校不太容易选，就不太好选了，然后我们再去考虑，然后我爸说我们再考虑一下专业你喜欢什么，但是我高中时候我从小就我感觉我什么都可以学我我，其实没有特别的针对性的想去学习一个东西，然后自己学什么，后面我们就我和我的爸妈，然后就去问了一下，包括他的同事，包括我们的一些亲属，有其中也有包括我的一些老师，也有老师，然后也有律师，也有医生，各行各业的我们都问了一下，当然这是最奇怪的结论。

06:09

我的小学老师、初中老师、告诉我、高中老师都告诉我不要去当老师，当老师很辛苦，我一个当医生的叔叔告诉千万不要去临床非常的累，我当律师的叔叔也是这么跟我说的，他们都不支持我他们这个职业去发展，我也不知道为什么。但当时确实因为没有一个很很能，很确定横向很喜欢的要读的专业，我每个都报了，我报了一也报了功课，包括南航，其实南航我是可以读的。

06:37

就这几个我报的学校，因为家还是比较喜欢求稳，因为高总那时候报志愿，他们还有那种搏一搏，每次可能再往前去一些更好的学校。我一般都是我根据那种报高考的报考指南，我看这些学校招收的在四川省，我当时是7千米，我都报他们的入党线必须要低于7000的，我都不会从那种招6000名招6500的这种，我都是我的分数100%可以进去的这种学校。

07:02

当时报了华东政法，然后南航难以大，后面我爸说这些，好像这个学校都不是一个985，那时候大家还觉得985 211很厉害，他说你怎么怎么也得报一个985学校，我说反正四川我不用传达我不报，然后北京上海的985那都太厉害了，我都报不上，那时候成绩不太够，然后报了个山东大学，然后学了个上大学，选了个什么专业，好像是人家随便选的，反正那时候也对，就是随便学生政法不是文科学校。

07:32

我们也可以报的。

07:32

我们这种理科也可以报，理科好像理科可以报文科，对，但是文科不限，但是文科要受限制的。然后等到后面是因为然后后面那段时间这个学校选好了，但是后面那段时间就是假期的那段时间，然后加上我家里面一些情况，外公病重了，然后后面我们虽然有一些亲戚在医院当医生，但是始终不是很仔细的清楚，可能是很远房的那种情形有一点点关系的。

08:01

那后面我爸说要不我们主要还是考虑一下多个医生，他说我们整个家族包括我爸这方的亲戚，包括我妈妈这方的亲戚都是没有一个直系的亲属是当医生的。

08:13

所以说最后我是把医科排在了第一位，所以说选择了南京科大学，因为最开始考虑的是天津科大学，因为好像天津医科大学是医科院校里面唯一个211，是评了211的，但是后面我看天津医科大学每年的招生在四川都是5千米6千米左右，我想都报不上。

08:31

他说而且那种现那时候就知道学医肯定是一个比较辛苦的，起码要读个硕士，那时候当然只知道读了硕士，然后后面看一哪一大在四川基本上招9000名到1万名，然后我不是7千米，我感觉我还高了蛮多的。然后又在南京，我觉得这个学校还不错，我就填了南南京大学填到了第一志愿，那就被这一段直接录掉了。

08:53

所以其实还是家里有发生了一些事情，让你直接促使你去学，对于你本人来说的话，其实学理科学工科是医科，对我不用那么强烈的。

09:06

后来来了基础医学，当时是什么感觉一定要转到临床去，从入学那时候当时其实当时填出来的时候，我就发了这个专业目录，我就不知道这个基础也是什么东西，我们家族这种也没有去问过的，我们最开始认为我们全家认为的一直认为的，基础学，可能就是那种全科医学现在认为的全科他什么都做，应该是内外妇儿科都做，然后临床医学可能就是专职去搞手术什么的，反正我们认为T恤可能全是去当临床医生，然后就先报名了，然后就发现自己被调剂到了基础医学。

09:39

因为刚开始我确实很失落，因为那一年我那一年南极大在四川的提到钱是580分，我考了620分，我考了40分。然后还有的同学还有一个同同班，同学他是贵州的，他跟我考同样的全国卷三，他就考了580分，我就被他搞了40万。

09:56

然后我们还是在艺术学院当同样的同学，所以你会发现高中学了这么多，最后结果和别人差不多，经历一种很大的失误，对当时确实然后我我爸妈后面也知道这些，他们后面都知道大家都知道，而且后面说就进来之后了解到出血，他以后包括硕士阶段也不能转临床，我们也就是没有资格去考职业，这个很受限制。

10:22

然后我爸就说，然后后面又了解到学校确实有转专业这个政策，在大一下的时候评估成绩，然后转专业，然后我爸就说你就好好用工，再用工一年，然后转个专业。就这样，当时确实他一定的目标是转专业到临床，所以上了基础医学这一年，其实你主要目标就是在努力学习去努力转专业。当然也不是这一年也发生了，其实也发生蛮多事情了，因为那时候我还进了学生会，然后进了学生会接触了辅导员，辅导员的关系还蛮不错的。

10:56

然后那时候就有很多的比赛就面向我们开始展开，面对基础医学的，比如说大跳小跳，直接面向于基础医学的。当时因为大部分其实还是那时候临床做的是比我们好的，因为那时候技术学院还没有划分出去，临床医学的大一到大三还是属于基础医学院管的，原来就是个很大的院征信，生物征信有没有分出去？

11:18

那时候我们辅导员认为但是这个赛事主要是技术学院来承办，甚至主要其实是面对技术员，他认为我们要有一个自己的队伍去参加这种大条萧条，去参加这种科研类的项目。然后当时我跟辅导员关系比较好，他而且那时候我也担任班长，也是一个学生干部，他就让我去组一个团队，然后去参加这种比赛，然后然后我就去报了名。

11:41

然后那时候跟着葛英斌老师生理学系的一个老师，那时候也确实已经开始进实验室开始做科研了。

11:49

然后那时候就开始接触你们组团，肯定要定一个方向对吧？对，方向是你自己选的还是老师这个方向是老师给的。当时就给你们的，对，因为当时确认那时候什么都不知道，因为那时候没有学多少的，就是相关的知识。进去以后有觉得和自己设想吗？会困难一点吗？还是？但现在进去之后？

12:10

比如这样的还有有趣的，我觉得做实验还蛮有趣的，那个时候觉得那时候觉得做实验还蛮有趣的，然后包括这些做一些动物实验，然后造模，我那时候感觉这个不跟临床医生在临床上给病人说怎么样，这是我们在给小组做这些手术，然后我们去研究这些药物或者是它的机制。

12:27

我感觉这样可能他更有更具有深深远的意义，而且然后还可以到一点，因为那个时候很多人跟我说，包括社会上有很多负面的消息，现在医患纠纷他也恶劣了，建议医生待遇特别的不好。

12:44

包括平时我有的时候去医院看病，因为我是一个排满键盘的人，我就跟医生聊天，然后包括我叔叔，也就是我一个当医生叔叔也这么做，他说现在这些医患关系真的还是最好不要去当临床医生。

12:56

他们都说你做基础其实蛮好的，比当临床更好，你做基础永远你是科研工作者，都是在幕后默默无闻的奉献，你永远接触不到这样的东西，你就永远不会有危险。

13:07

所以他说的好是你不会经历那些可能会引发尖锐矛盾的一些，而且他们说很累那个时候，但是那个时候因为我觉得做做科研还没有那么那时候还没有没有体会到科研累，可能那时候老师布置任务比较少，每天做一些实验，然后每天去然后观察一下小组做完实验之后给小组换药，换龙那时候觉得还蛮轻松的。

13:28

所以那时候像你的医生的亲戚跟你说了这些事以后，你算是认同他的说法吗？还是说是一个想法？还是蛮认同的，那个时候其实心里的天平就稍微倾斜了一点点，就算可能技术艺术也没有那么对，也没有那么差，最后没能转成专业，留在技术学院可能也是蛮不错的。

13:49

但这种其实那种心理这是大一时候的事情，对这是大一下学期。但是这种想法其实我一直没有跟我爸爸妈妈提过，因为我认为他们没有接触到这种这种环境，他们的认知是不会有这样的改变了，他们还是认为始终认为临床医生是最好的，就应该做临床。

14:05

你就应该然后包括以后我们家的人，就亲戚和他们的朋友，以后要看病直接找我，就这种医生是最好的。

14:13

其实对于你当时是报了转专业，报了应该不成绩不够，成绩不够，所以当时其实在意识到没有办法转专业以后，你父亲母亲其实还是会比较失落的，对是比较失落的，但是大一下的时候其实最后就是期末考完试，那时候还没有出成绩的时候，然后我说这个暑假可能要统计成绩，然后可能就涉及到转专业这个问题了，我就跟他们说了这个问题，我说可能因为每年每个学期都有成绩单，因为我还可以看我说排名我感觉可能够呛比较悬了。

14:47

当然后面我就跟他们说了我的想法，我说真正的以后不做临床医生，然后其实做科研也蛮好的，后面我后面就转专业没有成功，不能转业之后我就跟他们谈了，而且那个时候其实国重的这种介绍是在期末考试，在大一的期末考试之前的一段时间，就那个时候我们还不知道自己能不能转学的时候，就有活动来给我们宣传，那个时候我就把国中当成第二条路给我的爸妈去介绍，我说你看我们学校唯一一个就是国家重点实验室升级学，他开了一个股东创新班，我感觉这个活动上一般其实还蛮不错，可能符合我以后要走的路，如果以后我不能去当临床医生的话，我进入活动创新班，那时候活动他也跟我们说，本硕博连读给我们这样的机会。

15:29

我说到时候本硕博连读然后读个博士出来，我感觉这样的以后的可能就业前景也不会比临床差。他们认可你的说法吗，我不知道他们认不认可，他们不得不认可，我认为最后他们也因为我感觉不是太懂，我爸爸后面他不得不能，他没办法，因为我确实没有办法去参加这里，那就由不得他们了，但现在蛮认可。

15:50

你刚刚你刚刚说说什么科研可能是幕后会也会避免这种医患矛盾的这些事情，其实肯定有一些很好的事情，比如说不太敢跟父母讲，是不是因为是怕他们不太能够接受，还是怕他们会干扰你的选择了。

16:09

我感觉他们不太能够接受，我感觉因为他们其实对技术学院一点都不了解，他们只认为这些都是没什么用的，对人生对？

16:19

他认为这些都是相当于在给医生打杂辅助医生的是一种起辅助作用的，他认为最主要的就是去能拿七、手、猪刀去做手术的才是真正的临床医生。

16:30

你当时做挑战杯，结果当时其实不是那么的理想，因为后面就没这么做了，因为好像有几个原因，一个是导师的，原因，后面我们怎么都没有做出来，这个也不知道能不能讲，因为当时我们做实验动物模型，因为当时大家都是大一大二的同学，我们其实那时候还没有去考洞房的证，我们做销售模型，那时候在康大楼有一个销售方式给我们做销售模型的，当然后面防备胡笑查封了吗？

17:02

后面查封了，然后因为他这个是我们老师跟我讲的，但是后面我进入国中班，然后我跟胡佳也有交流，他也是这么跟我们提的，他说我觉得他有一次就是散步，他从看大楼那边上过去，就走路他从就走到工位，从学校走到工位，他就走到那条路，他就稳到这，有小鼠的味道。

17:20

他认为看到楼市做实验不应该有小组的味道，很浓的那种小组生存的味道。

17:25

然后他就找人去查了一下，然后发现那里面确实有一个养小狗的房间，但这是不符合伦理的小组，都应该在动物房动物中心实验动物中心去饲养，所以说私自有人在私自养这个东西，对。

17:37

这是几个老师私下里干的一些事情，然后后面就查封掉了，咱们就把所有的小组全部处死了，然后送走了之后，我们辛苦招了半年的目标，然后就没有了。

17:46

后面老师让我们重新做，在洞房里面让我们然后那时候去考东正，然后进东房，在东房里面又从头开始做，就花费了大量的时间物理，然后后面好像也没有做出什么就是真正的成果，老师这方面也不想再去做了，包括后面我们这些学生，有些其实团队除了我之外除了，我之外，其实大部分还是临床的同学，我找他去找的，临床中学，然后那些临床同学他们后面又考虑到要去参加临床的课程，要去医院实习，这个团队所以那个时候就已经大三了是吗？

18:21

他们大三我大二。我比你大一些，对，我找的是学长学姐。然后包括那个时候，其实大二那一年我就已经在国中里面了，然后国通还涉及到这些实验室的文章，还要证明当时说要考证证伦理这个证一般是什么时候考？一般是硕士才考。硕士才考，对，一般是研究生和博士才进步方向。

18:45

我其实大一就已经考了，我已经算是非常提前开始可以，为什么？像比如说像国中班，其实很多人都会做实验，他们不应该都要对国中班是进去的时候，国中班才开的时候，进去的时候，胡笑也考虑到这一点，国中班想让国中接受最提前的这种科研的训练对，因为正常来说，包括我们技术学和预防学以后也干，但是我们一般要到大四或大五，我们的这种学工办就是我们的培养方式才会向科研转变，前面几年我们还是学基础知识，然后去医院实习，实习完了最后做毕设的时候，会让我们跟着我们的本科生导师在实验室做一些科研训练，那个时候才真正进展。

19:22

然后但是国家想到国中班就是要提前一点，就这样打下的基础也比别人更老。就是说国中班就是会统一的去考这个证，你临床的那些血小学，其实他们也是没有这个证的，他们也考的。临床的也可以考这个证的。你不是说要硕士才能考上。理论上就是说对，但是本科生可以报，老师可以帮你报名，你就去考试，然后就可以过。我是因为我还没有进股东班，我就把这个证给考完了，因为那时候在做大调的原因。

19:51

所以其实你有分析过失败可能的原因，可能愿意很多，对，包括一些方向也可能有问题，因为当时可能是我们几个我们也不是因为当时都没有没有人带我们，因为我们都是真正的本科生，没有从来没有接触过科研训练。

20:12

后面我进实验室，包括在国中进实验室，那时候进实验室都是有一些已经熟悉了这样方面的，包括一些博士，就是经验很丰富的博士生的师兄或者是硕士的使用时点来带我们。

20:25

当时刚我们去做那个项目的时候都没有，没有师兄师姐带我们，不是我们自己就只给我们介绍一下，模型该怎么做，就师兄演示了一遍，当你把这个小组这样给他做外科手术做罪犯去除，然后他演示一遍我们做，然后老师从从别的地方拿了一管药，我们就是想研究这个药，这个药里面的其中一种成分，它对于瘢痕修复的影响，他先用这个药，他说你们先吐一吐，涂一涂，然后我们就先做手术，然后给这小组做完手术之后把它放起来，然后就每天给他涂药。

20:58

所以你说这段话说的意思，可能你们手术做的对实验方法可能有问题，实验设计也有一部分问题，因为这个药这个药是给人用的，给小鼠用会不会不能模拟这种真实的情况，包括药的有效成分，能不能真正进入这个细胞里面，有效成分能不能真正到达伤口都是不好说的，而且我们每个人做每个人做手术因为多模型我们要做很多做几十只，我们一般你不是一个人做的，就是要大家一起来做，每个人做的可能也不太一样，对就有误差，这种差别可能都会导致实验失败。包括后期老师老师他自己的精力也不在这上面，后期我们后面要做什么，做细胞方面的研究，要去做什么切片，然后要去做一些免疫组化免疫光这样的实验。

21:42

然后后面后面老师就让他的硕士去做了，就没有让我们再继续跟进这个项目，然后最后告诉我们这个项目停了就不再做了。

21:53

后面因为然后还有一个原因是给了硕士做，也可能是给了硕士做，也可能做出来，后面有具体有没有做出来，我就没有再去问了，这个项目就算是失败了，那就没有再继续跟进。

22:06

你刚说转你，基础医学不太受重视，是基础医学，我觉得就是我自己认为在我们学校技术也是在我们学院基本上我觉得有些事情或者有这种感觉，感觉学校不太重视，技术不是学我不是说学校不太重要，是没事你讲自己感觉。

22:26

我的感觉社会上来说，首先第一个是对社会是不太重视的，因为大家其实根本都不了解技术学院在做什么，然后去医院的人是不太重视的，医院的这些临床医生他们，我见过的大部分的临床医生特别强的可能会重一点，其他他们认为还是他们认为做手术是最重要的，你手术做成功了，才是最重要的，你这些搞基础科研的，很一般感觉比他们还是要差一些。

22:54

嗯包括我跟我临床的同学，我去临床也认识一部分的人，平时打球或者是什么交流的时候，我也感觉感觉他们对基础医学这个也不是那么的重视，他们我感觉他们还是认为临床就是最好的。

23:09

我们学校临床最好的基础医学搞基础科研的确实要比银行的差一些。当然我自己本身我也是这么认为，我自己自己本身也是这么可能，所以说可能我看他们也是这么看我们。所以在那个时候你可能是想报临床，但是还是没有报上，然后最后选择报了国重。果肉比较新颖的可能主要还是保研。

23:34

本硕博培养模式比较新，对是吧对。因为而且他比较新颖嘛，因为那个时候而且他名头很大，他是我们学校唯一一个国家重点实验室。那个时候其实我还不知道实验室这种划分这种我们国家重点城市到底是什么意思，应该说都还是不太清楚的。

23:55

直到后面我进去的时候，我才知道过去虽然是真的很厉害，所以其实你在当时了解过中班的时候，主要还是通过宣讲会。

24:05

嗯对，对通过宣讲会，那么三小老师给我们做了宣传，然后我们的辅导员也是大力雄霸那边也是大力建议我们去的，就是说甚至主要就是面向于基础和预防的，所以你周围同学当时我们周围同学基本上都是17对17级的，一对第一届都比较踊跃。

24:28

除了转专业的同学出来。啊他们转专业就是我的室友就是转专业到临床的，他们就不会去报。然后其他的人都基本上满箱包符合他们能够报名的条件，我们都报了名，技术数学基本上都报了名。

24:45

所以后来进了国中班以后，大二的时候应该是科研轮转，对初级科研轮转轮，1个1个学期轮2个，一年轮了4个实验室，我想对一下时间线，你这个大创当时可能从头做，然后做失败那个点是什么时候失败的点，卖一点就是在大二大二结束的时候18点大一点。

25:08

不是在大二中旬，应该是二中大二上学期的寒假的东西。寒假我也在留校做，然后就基本上就你还是留校在做，对基本上我一从大一之后反正到假期回家了，基本都留在学校，大一的暑假也是在备考活动。什么对，然后大一的暑假除了备课活动，那时候就已经开始在做大创，对。

25:36

当时大创的叫这个老师是你们辅导员给你介绍的吗？还是你们自己找的还是怎么认识的？

25:44

那时候大创他们有一个他们会发一个就是哪些老师愿意带大窗，然后他们有自己的项目对名单，然后我们根据名单一个去联系，因为有些老师他有自己的团队，他有自己的学生，我们然后这个也是我们自己去联系的，然后他的当时的方向可能你也比较感兴趣。

26:03

对，因为我当时看对他确实有，他会说我们要做什么，他已经把这些方向都列好了，然后我们去看感兴趣的。因为疤痕修复当时也不太懂得，当时觉得当时就太厉害了。可以让皮肤回到这种光滑的时候挺好，我倒觉得挺好。

26:21

所以科研轮转你可以跟我讲讲，啊有什么让你印象比较深的事情，科研轮转。

26:26

大二的科研论坛其实印象蛮深的，因为那时候那时候跟我真的接触到的科研还不太一样嗯，因为我原来接触到的就是大创，我其实没这么去实验室，我们大创每次去实验室做动物模型，重做销售动物，我以为大家每个实验室可能都是这样，一直做销售，做销售，跟小动物打交道。

26:51

然后后面我当时第一个去的第一个选择，杨洋老师应该是杨老师做干细胞的，因为当时我对干细胞也特别感兴趣，因为干细胞当时在社会也是个热点话题，杨老师也是刚转过来的，pi比较年轻，后面我就去了他的实验室，我发现基本我从来没见过小说，他们主要还是做细胞方面的研究，而且那个时候可能认为我们大二上刚进去，然后我没有什么基础，学的基础课程也没有学到位，然后也没有实验的基础。

27:20

那个时候其实没怎么让我们去动手操作实验，大部分时间还在给我们介绍，包括杨老师第一个实验室，杨老师他本来就是一个刚来刚，刚来南医大的pi他是一个很年轻的，他实验室也是很新的，他很多系统都还没建起来。他们实验室自己首先第一个人很少，时间是很空，第二个他们自己的时间就很少，所以我就根本都没有看到几个实验做几个实验，包括那时候还要上课，所以当时可能他们实验室没有正在进行中的课题。

27:49

对。或者是有刚才进入的问题，但是没有让我参与。有点像旁观者，甚至旁观的内容都没有那么多，因为我大部分时间我大部分时间，因为白天那时候基础医学的课程非常的满，白天时间都在上课，上课晚上6:00，马上股东那时候是要求我们每每每天6点，然后要去2~3个小时，我基本上每天去两个菜对。

28:11

你6点下课不相当于每天都要八九点才能结束，我基本都留到10:00，但其实6:00结束的时候，他们大白天已经把时间都做完了，6:00我去我也只能坐在实验台，就实验室的实验台，休息区，自己看自己的书，你当时不会觉得这样比较就我觉得很没有用，我觉得科研论坛也没有让我们学到这么多，但杨老师有一个很好的点，那是我记忆犹新的，我们有两个同学一起玩，他会每周给我们布置一个任务，他给我们一些专有的他干细胞领域的专有词汇给大概20~30个，然后我们去查这些意思，给他做一个民警。

28:47

列一个就是word，每每周发给他相当于每周的一个作业。我觉得这样是一个这是比较好的，我真正去深入的了解大细胞领域，然后去了解科研，包括也能提升我查资料的能力的这样一个途径，我觉得这个是唯一有用的，当时这样讲，除了其他可能我都没有学到什么时间技术，我连那时候用一夜枪，就是用那些仪器我都用的很少，可能三个月我就用了不超过三次。

29:14

所以大部分时间是在实验室自习，对其实没什么意义。你有跟同学讨论过这个事情，我讨论过我讨论过，因为这就涉及到我们下一次，因为下一次还要选我那时候我就问他，我说你们平时你们选的那几个怎么样？

29:28

然后有同学就跟我说，他说有些实验室就特别好，实验特别多，每天都做实验，每天而且是不是也带着你就手把手教你怎么做，对，他们晚上去了都没有时间，对，而且晚上还做得蛮晚，的嘛蛮多的，而且舒适也那时候也也很人也挺非常好，很热情的那种，这就涉及到这就会影响我下一个的轮转的判断。

29:51

所以说我第二个时间，第二个轮转的时间是我去了王老师的实验室，王晓明老师免疫他是免疫学的pi但是也在国中的pi的名单里面，他有做一部分升值的内容。嗯那当时去了他的实验室，确实时间非常多，是不是也非常好，是也非常的好，去做很多的事情，而且他会特地安排好。

30:13

比如说如果他有机会的话，他就会特地把他的心安排在晚上，为了给我们展示，为了给我们看，比如说他做的好的一些事情，然后在我们看够一定的数量的时候，比如说你看了个两三次之后，他认为你可能可以操作到哪一些废弃的，样品像我们练手，然后练完手之后，甚至可以真正的帮他做一些工作。

30:33

首先先给你看一看，然后给你那个时候后面再给你安排，就是真正相信你真正觉得你可以做出来之后，然后才什么时候才会相信你。你有他自己的考核，对他感觉你过他应该是有自己的考虑，比如说偶尔安排你去去做一些实验，做一些首先肯定是他后面跟我说的，他自己已经做过两三次的实验，就是已经有结果了。你只是让你去重复一下这个结果。

30:59

这是第一个，你首先要重复出来它的结果，这证明你的实验基础没有问题。

31:03

然后还有一些基本的就是看你这个人，比如说周末没时间的时候，他是要求9点早上9:00要到实验室的，看你有没有准时到，你们不要上课吗？就周末的时候早早周末早上，然后每课的时候也是没课的时候，比如说星期星期二星期四这种没课的时候，也是早上9点去，实验室管理是比较严格的，那时候当然他要求是反正你早上早早上来了之后，然后就跟着，然后没有时间的时候你就可以走。

31:31

然后那时候我还对蛮感兴趣的，然后比较积极，我一般都是早上9点去，如果是周末星期六星期天，我早上9点去到晚上11点我和世界一起一起结束他什么时候做，我什么时候他坐在座位上看文献的时候，我就跟师姐说，我说你要发几篇我先给我看看，就那些英文文献我也跟他一起阅读，我看他11点或者有的时候12点自动实验室离开，我也跟他一起离开。

31:52

你师姐是研究生的师姐是博士的事情，现在是博后那时候是博士。他也蛮努力的。那就是因为这样，然后逐渐可能是逐渐博取了他的信任，后面开始逐渐让我帮他做一些东西。是你师姐让你帮他做，对我师姐因为玩的时候把我交给我这个世界，实际上这个世界全权负责，往事后面就都没有基本没有照顾我，我有什么困惑，有什么聊天，我就直接跟我这个世界谈。

32:18

所以很厉害，能基本上能解决我学术上所有的问题。

32:24

然后这是讲第二个选择，后面第三个本来我想一直一直连着的，后面当时一直连着是这就是对不是选4次，我想第3次第4次还是在选择时间，就第一次留在，但后面说这样不好，后面国中的主任郭亚云老师跟我们说的，你最好是分开选，你不要老在这个轮转就是为了让你们多去看，郭小云是你们的顾顾雅云对是国中的班主任。

32:47

现在应该还是班主任。对我说，我想他说的也有道理。然后我就再去看了一下，然后第三个实验室选择刘明奇老师的实验室，有些老师实际上是后面结果去了之后还是跟第一个实验室差不太多。

33:04

先看你会不会跟郭老师反映。比如说某某老师我去了以后其实也没有时间，我觉得因为那个时候对那时候国中不仅是国中本身很关心我们，包括学工办他这种因为是第一届，第一届总会是各种各样的问题要遇到的，都要去解决就是，所以说偶尔还会给我们开会，就是问我们遇到了哪些问题太少了，给我们安排时间太少，每次都要我们谈话，每次都要我们看太久了，应该让我们一做我们每次都会反映这个问题，但就是不解决。

33:34

他们有的时候其实没办法解决，因为毕竟是人家我后面的理解他毕竟是别的人家研究硕士或者是博士在做的课题，万一你把别人的样品弄坏了或者污染了，比如说他们两三个月进行才有的一次样品如果被你搞掉了或者污染了，这样也很耽误人家的进度的。

33:53

这是确实是要足够信任你，我还认为他要足够信任你了之后，你要体现出能够帮他解决问题的能力，他才会选择去到你帮他做一件事情，就像第二个实验室的世界，他已经很信任你了，所以他愿意把自己一段时间让你来对他相信你，所以说他愿意承担，你如果没有做出来把他的结果弄错了，这样的后果。

34:13

这是后面世界他主动跟我说的他说，他愿意来承担这样的后果。

34:17

就是因为足够钱，你觉得你的能力应该可以能够完成这个了。

34:22

我认为每个实验室可能考量都是这样的。第一个养老实际上是因为他们确实新开刚开始很多真正的实验室，他们刚开始的时候他们要建一些系统，一些成熟的实验方法都还没有，他们他们可能本身，而且那些硕士都也他们也是新招的，我去了我了解他们的研究生的世界也是今年才来的，他原来也不懂这些的，所以他们还要摸一些条件，可能他们自己都做不太出来，还更别替代我了。

34:48

然后第三个明星老师那边，明明老师那边还比较成熟了，但是明天老师而且刘强老师性格特别好，这是我同学也是我同学给我推荐的，主要是特别还可以，甚至还可以跟大家一起吃饭。我现在很少看见实验室的这种导师能够跟学生一起吃饭的还有说有效的，包括一起打游戏，这种都很少见了。

35:08

所以刘老师还是为人是特别好心，特别容易相处的。他那些兄弟也挺好的。

35:14

但那段时间还是和第一个差不多，没有什么事，而且是那段时间可能而且因为他们要放假，我第二个实验室是不放假的，一直要干活的，然后第三个时间它是国中真正的做男性的发生的工作时间，他们是每个星期天放假的，然后星期六偶尔也没有什么事情，但是我主要是星期六和星期天才有时间，星期天星期五还是上课的，而且那个时候还在其实银行经理也还是在去跟进大创的。

35:45

就一星期偶尔还要去做大创，也可能是有一些时间，但是我错过了。但是总体来说第三个实际上是比第一个多一些，但是比第二个要少。所以你到第4个时候有没有想过要回到第2个？

35:59

没错，我第4个就是回到第2个，我的真正的路线就是第4个，然后又回到了第2个实验室。然后就继续待了有半个学期的样子，对大三还有轮装大三就没了，大三我就退出国中了。为什么突然就退出来了？因为因为我现在还留在就是，实验室里第2个和第4个轮赚的钱，我现在还在这个实验室，现在到我时间还在这个时间。

36:25

然后因为那时候王老师，因为我前面两个杨老师，第一个和第三个实验室，他们其实都是真正本质上是做生殖相关的工作的，包括一些干生殖干细胞，第一个是做生殖干细胞，第三个是做精子发生的吗？到王老师那边，因为对他是兼职国中的偏，所以说他大部分跟升职关系不大，只做一小部分，生殖免疫的内容，就生殖和免疫结合起来，大部分还是做真正的免疫的单纯的免疫的方向。

36:51

而他让我跟的那个世界因为我最开始听的时候，他就说我找我最好的学生应该也是他的第一个。

36:59

第一个学生开门大弟子们大世界，他说我找我的就是最好的学生，那也是他最好的学生，找大师姐带你，那时候还没有他不涉及升值的那种，我跟着他做的，所以说做的大部分东西全是面衣的，根本不涉及，甚至然后那个时候然后后面然后后面不是两轮两次轮转，后面选了两次，他们后面然后我就慢慢再做一些免疫的东西，后面我也去了解了一下。

37:24

有王叔叔，王老师后面说你这样，因为毕竟是国中班的学生，那你还是可以再去再跟另一个师兄，你稍微去见识一下生殖免疫是怎么个做法的。

37:35

然后我也去看了一下，然后师兄教了一下我，后面然后在这种做的过程中还是王老师的实验室的，对另外一个就是做升值的，师兄也是一个博士师兄，然后我也跟着他反正两个都去看一看，看看，了解了之后，我确实发现虽然在实验室里甚至免疫，因为也不是那么成熟，就相当于第一个时间段，因为这个也是我们也是王石新开辟的一个方向，所以说有些很多实验技术也不是很成熟，实验结果也不是那么的好。

38:05

然后包括你师兄是硕士还是博士硕士博士，他但是成果并不是让王老师和你们满意，没有做出特别好的结果。

38:16

包括我在干细胞里，然后我去刘江老师我也读了一些文献，因为每每到一个实验室，他们的方向不一样，你就要去读一些文献，了解他们是做什么的。我也读了一些文献，我也看到他们做一些东西，我感觉可能做甚至然后甚至于国创也蛮照顾我们的，让我们去外面看一下，带我们去了。是妇幼的附属的生殖中心，我们去看也去参观了一下。

38:42

然后后面我感觉然后后面因为我做大创其实还认识了不少的老师导师，我就跟这些导师也他们不是跟一个老师交流吗？但是那时候要选的。

38:55

我今天打电话对，然后我就而且我那时候不是还在学生会有工作，我学生会然后那时候我们策划一个节目，是技术学院的学生会技术学院策划一个活动，他为了让医学不是过程中，基础医学的同学更了解基础医学，去找了各种各样的PA，就学海楼的这些各种方向，包括生理的，包括病理的这些大导师介绍一下自己，不管是实验室还是他们的求学经历，然后让这些同学对科研更感兴趣，我作为采访者去的。

39:24

所以跟他们都对，然后采访完了之后，我就顺便问了一下这个问题，我说我就问他，因为当时我也不是太懂问他们，我说老师他说你这应该是你大一的时候的事情，一二二大二我不仅在国中也在兼职那边学生会干部，我说你们觉得就是股东怎么样？生殖医学，他们说生殖器学挺好，的呀，特别是我们国家重点实验室，他有这个名头，它就会有很多的经费，后面我就意识到做科研经费是一个很大的问题，经费组才能做出一些好的东西。

40:00

但是他们甚至有些老师跟我说，甚至做的太还是太偏基础，一些了，太偏真正的其实我读文献我也自己思考这个问题，大部分的那些我看做的科研就是可能当然我读的不是很多，有我自己的理解，他们大部分研究基因或者是分子，它对于我们疾病的影响，比如说他发现这个基因突变了，我们就不能产生精子了，或者是不孕不育了，他就研究他为什么这样造成的，但是很难去做到。

40:30

转化医学就是治疗，治疗医学就只能通过辅助生殖技术，辅助性技术也带我们去生殖中心看过了。辅助生殖技术，但是后面好像说辅助生殖技术好像也不是我们技术学的同学，因为我这要考虑谁先说，是那种别的学校来做还是好像是那种也临床的可以来说，他们也要临床技术还是什么样的。

40:54

所以我还可以说你其实对辅助生殖会更感兴趣一点。

40:57

因为那时候大一大二其实那时候不知道为什么很成熟，就考虑到就业的问题了。

41:03

因为一直说作为通过手机出去，其实很容易找工作，我就一直在思考这个问题，谁一直在说周围的人包括我们的学工办的老师都说你如果不读个硕士，读读过博士是很难找到工作的。

41:17

我进这个实验室第二个就是王老师现在免疫的实验室，中介也跟我说技术学难道不就是留校当老师吗？但是也起码要读到个博士，求学的时间应该蛮漫长的，你不像临床，你临床如果实在不行，你本科读完你也可以考职业啊，你考了职业自己回去开个小诊所也蛮不错的。然后后面我就在思考这些就业的问题，整整个以后的走向，其实我确实对辅助生的技术挺安全的，后面好像感觉辅助升级技术更愿意要一些临床的他们手比较稳定的这种去做。

41:48

然后我们平时就是在国中待的，当然我因为火种它很复杂，它还分为基础果种和预防果种，它分为两个部分，但是我们都可以选股东平台，也有一部分是预防的，但是我看周围的就是我们第一季我们接触的同学，基本上只选基础医学院的老师，他们工位的同学会选基础的，也会最后还是会回到工位去去工会的实验室去学习。所以这就涉及到一个问题，基础的这边的老师，他研究大部分都是很你有没有想过为什么工位的都要选工位老师，其实都要选技术老师？我从来没想过，我想去想，我真的是我跟我同学也讨论过这个问题很奇怪，因为工会的因为我们其实表面上大家最后可能做的都是差不多的东西，最后都是搞科研，但是我们其实学习的本质是不太一样的。

42:36

我们学的东西不太一样，工会他们可能有的时候还更注重一些数据方面的分析，其实我们生物信息学的蛮少的，包括他们搞的一些这些整合的大样本流行病学，就是搞队列方向的研究，我们就是做小样本，我感觉他们我们不太懂，但是但是他们工位的就没有这样的烦恼，因为他们后来我们基础他们就是学实验技术，因为他们也要做实验的，他们就是过来，所以说我看大部分工人都有4次轮转，有个一两次来技术这边选导师，然后做学一些实验技术，反正最后定我看大部分人还会有对，最后基本都会没有工位的同学是定基础的老师，所以这是一个问题，然后我就发现技术的老师做的太基础了，全是这些机制方面的研究。

43:20

我们他也不不那我们就学不了，辅助生殖技术我们没有学，那我以后怎么去生殖中心就业？我就感觉我应该是就不了业了。然后基础这样我感觉然后后面就感觉就不太感兴趣了。

43:36

可不可以说你其实对科研兴趣不是非常的大？

43:41

可以这样说吗？

43:42

其实可以这样说，的因为还是我想因为还是比较想找点工作，也不是想朝这个目标，是想找一个好一点的工作，包括我现在读不可能以后读硕士读博士，我也只是为了拿到硕士或者博士学历，或者是有发一篇发几篇自己属于自己的就是期刊，再出去找一个好朋友，这些都是，所以其实你读博，就算你要读博，其实也是为了找一个好人。

44:12

是的。

44:12

好，工作定义是什么？工资分要高一点吗？

44:16

还是说社会地位要高一点，还是说这些都要考虑的，包括工资，包括社会地位，包括这种轻松的程度，我就是感觉基础干做技术，我原来觉得做技术的很清楚，这个是我大创始的想法，后面我真正深入的实验室了，后面我现在真正深入实验室，王老师已经帮我当成一个硕士或者一个博士在培养的时候，我觉得真的好累，真的很累。

44:41

这个时候我真正退出过主动，当然后面推我，我发现我才发现了活动活动还是蛮轻松的，他们还是有假期的。他们国中好像是要求大部分省市还是每周星期天还是放假的，他们也有寒暑假。我们在王石的实验室，就我现在这个实验室，我们每年只放3~7天，博士生只放三天假，大年初一到初三，然后硕士脂肪大年初一到初七，其他任何的节假日我们不休息的，所有的节假日我你就知道我就感觉好辛苦，我感觉出去当然也这只是个例，这是个例。

45:16

后面我了解到我的同学没有在国中有没有在这些实验室，在别的实验室？别的戏他们也蛮轻松的。所以你意识到有没有意识到这些，还是看老师对这是看老师的，但是但是我后面一直到，但是后面我甚至问过师姐，我说为什么我这么辛苦？所以说我们做的不好啊，但是其实我同向的对比了解，其实王老师现在这个时间已经蛮不错了，他每年可以发还是能发布。

45:40

不知道好文章的。其实跟别的实验室比，包括免疫学习，它现在其实是属于免疫学，我认为在语言学习已经属于比较不错的老师了。

45:49

明确有很多老师老板，确实他们那样的，同学很轻松，我有同学就是在别的。

45:55

不好意思我打断一下，你说只放一年只放三天，周末你其实也没有我们没有做不来，那要有什么事情怎么办？请假。

46:05

有这个那么也不是这么不人性化的，是可以请假的，你不能请太多。钱太多了，老师肯定是对你有意见，是反正原则是紧张不紧张，除非有特别困难的事情。比如说你有病吗？你身体不舒服，你可以休息个半天，也不是说绝对不允许的，是可以的。

46:24

但大部分时间还是尽量是在实验室的，你没有什么事儿的时候，你今年还是在实验室干活，所以你在实验室，其实你去实验室，现在王老师实验室也是在干比较重要的活了，也反正现在基本上是把我当一个硕士在培养，并且然后王石也跟我谈过这个以后，也留在这个实验室，就是我读研究生，也在这个实验室继续读。

46:50

但是太累了，你会觉得会让你有点刚开始其实这个也分，其实前几年的时候，因为我大二下就定了在那边推出国从，大三一年四现在大四下了，大三一年的时候觉得特别累的时候，而且很多课甚至为了做实验，很多课都不去上了，就直接去实验室开始做实验，做一天一一整天的实验。

47:12

现在然后后面那个时候可能是纯粹的思想，相当于像王老师或者像我的世界证明我自己的能力就跟同样的本科生比，或者跟一些刚进来的硕士比，我是有这样的能力，我是有这样的做实验的机会，从而让他们更重视我，更给我一些好的资源。

47:30

到后面大四的时候，我们觉得可能有点太累了，我还是要学会适当的放松自己，后面现在还是需要有一个调整的过程，就是慢慢调整。

47:42

所以你现在还在那个实验室，一直在可能以后也会一直在实验室，而且还把我的室友把我的室友都改成从此改变了我的室友对于这个技术学的看法。

47:52

因为我每天早上我那时候做实验的时候，两三个月，我早上8:00去实验室，他们还没有起床，然后我晚上十一二点回去，他们已经睡觉了，他可能两三个月连我人都看不见。我所以就说他们但是我们真正技术学是要求大四才去实验室的。不会。实际上是都这样，这太累了，做技术就太累了。

48:13

所以他现在从来没有考虑过在技术学院读研究生的打算，他准备直接去考公务员，就跳出这个专业，你那个是转临床的同学没有就是基础医学，他就不愿意再读技术去了，他都是以后不想再从事跟相关的东西，所以说只能去靠公务员。

48:31

因为现在他们也涉及到考研就是考工的问题，所以其实虽然很累，但你还是决定留在王老师的。所以我可不可以说是因为也不是，因为你要这么说的话，其实国中的就业应该我认为股东的就业可能还更好一些，当时是纯粹是因为感兴趣。

48:49

我现在也不是不感兴趣了。

48:51

有比重感兴趣和找工作都是有比重的，也不是哪一个占第一位的，你在王老师时间做多了，发现其实对这些干细胞这些它不是不叫干细胞，应该叫免疫这方面的东西还是比较感兴趣的，对，而且那个时候这个也是个热点嘛，肿瘤大家谁不关心肿瘤或者癌症的治疗？

49:12

好多诺贝尔生理学家不就pd1pd01都颁给这些地方，甚至我感觉虽然确实现在甚至很重要，我们当年去的时候每三不管上升值相关的课程，第一句话包括老师第一句话都跟我们说，现在是不孕不育的发病率，已经占了全球所有疾病的地第三位第一位是心血管疾病，第二位就是肿瘤，第三位就是不孕不育，说甚至也是很重要的。

49:38

这是我可能个人对于升值这一块不是那么的感兴趣，还是对肿瘤更感兴趣的，还是想去研究一下癌症的这些方向。

49:47

所以其实刚刚有说到你去一个师兄他那边去看生殖民对，对。你去了解了以后发现其实可能不是很感兴趣，而且那时候做的不是很好，我做不出什么成果来。你其实你也做了一段时间，我没有做这他对师兄他都没有做出什么，他毕竟也是个博士，呀我还是个本科生，他都没有做出什么成果，能要求我去做出什么成果，我认为没有做出什么成果，你科研就相当于是所以其实他这一点是有点劝退你的，可能我去了生殖免疫这一块，在国人办继续待下去去生殖免疫这一块，可能出来的结果可能并不会出来什么好的，对，而且后面最开始而且国中班它的制度是一直在变化的，我们第一届就会承受这样的问题，我们刚进的时候跟我们说的是招30个人，30个人所有的全部保研全部直接拿到研，你直接拿到就是保研名额，没有任何的硬性指标的要求。

50:44

后面然后就说这样后面可能是无效去跟这些讨论，发现要不到这么多名额，后面就变了。

50:52

他们之间我跟我后面股东同学说他们也买什么，后面变成了英语要过6级，这是最基本的，英语过6级，计算机过2过2级，然后成绩有排名，成绩你必须要保证在，年纪前百分之多少来着？

51:05

那时候40%40%对，然后保研然后就改了，不是所有人都能保研挂科，也不能再抱怨了，包括后面其实我专门去问了的，这些其实要求我都能达到，我曾经也想过是不是如果我还是可以借着国中班的平台去拿到这样的保研制的，因为这些我都没有任何问题，我都符合。

51:27

但是因为王老师我现在这个形式他也是属于普通品牌，我就一直选他不就好了。后面我去问了，互相这个问题。

51:36

国中班毕业必须要以做升值的课题去毕业，必须要升值相关的课题，你本科要做这样的方向才能够毕业，本科才能给毕业。因为后面要涉及到他们的就是毕业涉及到答辩了，答辩的内容必须要说清楚相关的。这一点在招生的时候有提过吗？这两个t加上什么问题？

52:01

所以你觉得可能你要做毕设的话，肯定是做免疫学上面相关的，对，因为这样我如果做升值方向，不一定能做得出成果，如果没做出成果，我怎么去毕业毕设你总也要做点东西出来。本来我们就不是那么成熟，这也是一个考虑因素。

52:18

然后后面还有一个考虑因素，就是后面股东说他说保研名额他给了，然后甚至让我们本硕博连读5+1+3，在一年级的时候甚至然后再考核一次，在期中答辩变成，然后如果你合格的话就读读博士来1+3。然后后面我想呢我们这身体免疫越足，就是哪怕认为我如果说毕业本科毕业了做一点点东西说不定还能做出来。

52:45

但是我不能保证我后面能够5+1+3的顺利的，如果不能5+1+3，纯粹的55+3+3就是正常的这种读书的话，那么他和别的相比的竞争力就不是那么大。因为我本身那时候我在技术学院的本身的排名也蛮不错的，第二名还是第三名那个时候我如果退出国中，我可以去拿技术学院的保健品，我不需要过重的保健品，他对我的心理一下子就没有那么大，所以说是不是说5+1+3，其实人家是必须要做升值方向，你要走这个路的话是的，你要走5+1+3，你这一点特地问了。

53:23

对我专门跟他们有建议沟通过。

53:26

当时是我吸引我的后面，但是后面我发现我没有办法达到这样的条件，所以你觉得可能我去做了5+1+3可能就要做生殖免疫，做生殖免疫可能做的结果并不会那么好，我们没有办法5+1+3这样毕业。

53:40

对，现在因为我后面还是发现做基础，你就是要读到，博士的话我还是希望越早越好，读的越短越好，对，谁如果能够少读两年，谁不愿意，对，但是做身子发现好像不太能够，当然还有一个前提可能是换一个实验室，可能王老师事情是不行，但是别的真正他们只做升职的，他们应该肯定能做出一些东西，因为他们都很成熟这些技术。

54:05

但是那个时候就涉及到大二4次轮转，我已经第4次那时候第4次就在考虑这个问题了。

54:12

已经第4次轮转了，我已经花了40多年时间，那不相当于没因为我的这些技术大部分技术还是在免疫学这个领域去打造，当然实验可能实验技术是相通的，但我大部分了解的包括我的一些科研思维的形成，可能还是在免疫学这个方面，而且我再去大三重新再去轮转，再去别的新的老师那里，我要重新开始熟悉环境，要重新那时候也带我做新的实验，然后就要重新去然后看看他们对于升值这一块是怎么做的。

54:40

他们的套路又是怎么样的，而且包括一些甚至要竞争别的同学，有的同学我就专门过来，有的技术同学他们就这样，他们也是像我一样，他们也有大二的4次，选了2~3次，同一个老师的。他们那种也跟老师谈好了，肯定以后要留在他们大三也直接留在那里。所以说其实我认为可能大部分比较好，一点的老师都已经他们那学生都已经提前去过了，我再去的话也没有什么优势，就跟我同级的竞争也竞争不太过了。

55:09

如果再继续做升值的话，我都考虑过了。

55:13

所以考虑的还蛮多的，所以其实因为很慎重，我考虑了好久了，非常慎重这些这些打算不是随随便便可以做得到，你考虑这个时候有没有跟同学老师或者是家里面人讨论过都讨论过，我所有的都讨论过，所以我们每个都讨论过，你把你的想法跟他们讲，他们其实也是比较认可你的想法，有没有人提出过不同的意见？

55:37

好像也没有。

55:39

我的有些同学是提出过这样不同的理解，他们是这么理解的。

55:44

你也可以他们觉得拿技术院的保研名额可能还会有风险，因为开了大涉及到现在大三大四大家还在卷，还在重修刷分，当时拿国中的保研名额是100%，而且股东每年还发1万块奖学金，那也是一笔不小的钱了。

56:02

然后他们甚至建议我你没关系，你就待在谷中，你就保证你本科毕业，然后你拿他们的保研名额去别的学校去更好的学校去去比如说去上交去复旦，这是不限制的。

56:17

我也专门问了我的辅导员，保研名额是不允许限制，只要别的学校的老师愿意收你就可以去。但是后面其实我琢磨一下，我觉得这样不好，因为你用了国中这么多资源，你最后你不仅没有留在国中，你反而连南大都没有留。其实我个人的就是道德是不太能够接受这一点，而且白白拿别人的奖学金就也没有去做跟甚至相关的东西，我觉得这样不太好。所以说我就没有这样，我说那干脆直接退掉了，不要再拿这些把这些，待遇留给别的同学。

56:51

你有没有考虑过，假如回到基础医学，其实你们的课大二的课是有一些删减，是的，你要重新再补。

57:00

对我也考虑过这个问题我考虑过的。

57:02

幸好因为这个时候就考虑过大儿子大二的时候，大家上的还是比较基础的，生理生化病理病人他活动都没有办法删除的，他只删除了一些无关紧要的一些课程，比如说神经生物基本上只上了大概两三门，这种我还容易补一些。然后如果我到大三结束，因为后面告诉我们每每年结束才能去推，可能如果我到大三结束再去给我大三那三次课就太多了，我就可能要补个七八门八九门，可能就再补都来不及了。

57:32

所以与其这样还不如早点对早点退，因为反正我已经决定了，可能以后不能再甚至这边也不再拿他们一年奖学金了。

57:41

嗯所以其实你现在相当于要去拿技术学院的保研名额去做免疫。

57:50

现在是打算你拿了保研名额，其实你也可以去别的学校，不一定要去王老师实验啊，对，但是我跟王老师已经说好了，我们当时已经谈好了，你现在其实比较确定要去他是读研，我这个人就这样，我觉得一旦谈好了，你们虽然只是一个口头协定，但是你这样再去别学校不好，我认为这个不太好。

58:13

所以说确实现在有很多同学，我去为什么去刚好学校发展一下，去什么去更好的学校发展，因为他们因为毕竟其实我的技术还是蛮不错的，跟这些统计的学生比，以前我确实接触的太早了，他们现在还有很多同学是，我的同班同学是没有进实验室的，从来没有接触过这些，你现在大四比如说你刚说大四你们都要进实验室，对，但是他们但是要看老师的制度，你你，我们他们进了实验室，对老师也不管他们的，甚至也杨老师还给我们，但是我老师从来不管他们，也说你们想来就来，不想，所以说他们可能刚开始去的样子，后面他也不去了，直到最后王老师有这样的同学，就是我们上一级的基础医学的大五了，他们要毕业答辩了，已经要毕业答辩了，已经要写毕业论文的时候，这个时候再来实验室，所以我认为其实主要约束的还是你自己，对，你看你对没有其实完全不约束的。

59:11

你看你自己对自己要求你想干的好一点，你想以后未来的路走得长一点，你就去努力一点，你想这样混着也可以没问题，也可以很轻松的，每天休闲的也可以过。

59:22

所以其实可不可以说王老师他做的可能肿瘤免疫这一块你比较感兴趣，而且其实对你来说可能找工作也比较好找一点，所以你比较确定的就是要在他那个实验室里面，这个也在整个具体流实验室当然有很多因素了，这是比较主要的因素。

59:44

对，应该是。

59:46

虽然累一点，但是其实也可以接受，对，因为累一点，但是只要能做得出东西累一点就累一点。

59:52

可以做出一些成果还是可以的。

59:55

你有对你未来职业做什么规划吗？有想过这个问题吗？后面？

01:00:00

一直在想，我从大一一直想到四大五，因为我感觉技术研究这个东西真的很难找工作，他真的很难。所以我一直在考虑这个问题。

01:00:09

其实这一条路最主要的方向就是留校当老师。我们开创专业的本质就是为了招一些大学的老师，但现在高校这个老师太难当了，就进入高校的要求太高了，但以后也还当然以后还是要看自己做的，如果就自己做的自己的科研做得还不错的话，还是去读博读博士应该要读的，读博不管做的好不好，如果没有博士学历，没有博士学位，我认为硕士毕业和本科毕业都没有什么竞争力了。

01:00:39

然后如果以后再好的话，或者做不好，因为带我的时间，我跟你说从大二就一直带着我嘛，他就一直到现在还在黄石实验室，我就很佩服他，因为他他确实能支付。他很厉害，他不管是学术还是为人处事方面都让我觉得很敬佩，我一直跟着他，我们的关系也特别好，所有事情都跟他说，我们就一起在讨论。

01:01:01

他现在也在王安石先做博后，我们就一直在讨论未来怎么走，他告诉我哪一大就是做博后的要求以后怎么在哪一大留校当老师，这当然是一条路，还有别的比如说去公司就业，如果做的不是那么好的话，博士毕业然后去生物公司，也有人说我们可以去医院，但是我现在不就在医院实习，大四是实习。

01:01:25

我在医院实习，我发现跟这些临床医生聊天，他们其实也不怎么做技术科研，他们对技术科研的热情度也没有那么的高，我们还是想着做手术，就跟我当年任职是一样的，他们还是不太认同科研。可能美国这方面就是国外这方面风气稍微好，一点点。你说的和医生聊天是不是说以后就业去医院，然后和临床医生一起去开展实验研究，对啊对？我以为这就是一种模式。

01:01:51

因为当年大一大二的时候，我们的叫什么导师？

01:01:55

是书成长导师苏川院长，他就跟我们这么说的，他说美国是一个路径，他说美国这样的一个临床医生，然后后面加几个做基础医学的，这样形成一个team，一个团队，临床医生在临床找问题，然后我们基础的就负责在科研上去解决这样的问题，然后从而把它从小小个体推广到整个群体，大的群体，我原来以为还蛮好的。

01:02:18

你在哪个医院实习，是跟哪个院医生做医院衣服是吧？我看他们不怎么做科研，他而且我有时候跟他们我就听他们开会的时候，我就听他们听他们自己聊天，感觉他们对科研不是那么重视，纯粹可能是为了晋升他们要有晋升的要求或者有奖金的要求，才会勉勉强强接触一点点。

01:02:43

就对他们来说，更重要的是做手术，对手术做的多，奖金就高，对对我觉得但可能是比较局限，可能是一附，院可能是接触的医生没有那么多。

01:02:55

其实我这个是跟访谈没关系。

01:02:58

我了我了解的其实有一些医院他会比较愿意招那种做科研的文章的，因为现在医院的评价体系非常的看重科研，但是临床医生他不是说我们人人都能发那么多高分文章的，比如说你像看复旦的医院的排行榜，它有一块是声誉对吧？还有一块是科研，科研其实看的就是你的文章，所以会有很多的医院它会专门招那种科研人员，你进来不是做临床的，就是你进来的任务就是给我发高分，文章就是以这个为目标来找。

01:03:32

因为很多医生他很很多医院在接受评价的时候，他会看你医院发的文章的营养因子，而且其实肿瘤其实还是蛮好发的，对高分子文章算一科里面比较好吧。一一对对，所以我觉得可以算。你可以稍微关注一下，对其实我也是这么想，你知道这个东西我知道的，因为知道肯定我我我原来只是以为是你去医院了，不是医院可能是当时还没有真正好的医生，我的真的好的医院他们还是很注重非常注重科研。

01:04:10

一个医院其实是大概我应该是我进来两年两三年以后，18年的话差不多起来了。

01:04:17

那个时候招的医生很多都是硕士生，对我感觉好像感觉就是很多可能比较热门的科室骨科他们都是说是对，但是到现在可能不太好进了，因为现在是在博士了，我当时现在体系已经充足了，所以他就没有那么强的需求，所以现在没有以前那么好，刚建的时候其实还是很好进的。

01:04:37

像那时候像我那些学弟学妹，他们有点看不上你们医院，现在进都进不去了，因为一附院起点还是蛮高的，这毕竟是学校直属直管的，所以它的平台还是不错的。

01:04:53

但是刚开始其实没有那么好，所以里面的医生可能可能鱼龙混杂，我也感觉刚进去的可能数据还不错，但是那种第一批进去的可能就没有那么的但是相对于像省医这些比较大的医院，包括像鼓楼他们其实都很看重科研这一块，确实甚至会招那种全职的科研人员，为了高发的高分文章，你看排行榜的话，你会发现沈毅其实生育没有那么的高，但他的科研分数很高，曾经超过了很多前10的医院。

01:05:27

嗯所以他们其实还是蛮看重。对，但是我觉得我们的访谈没什么关系，但是你这个是跟从哪了解到的？我说的信息。是跟你学姐讨论的吗？我跟我学姐讨论，跟那些老师也讨论。

01:05:41

导师对，因为我们有一些导师他是会跟医院有合作的，他们说其实医院还有些慢，而且那时候我们实验室而且一般都是好医院，对，而且我们实验室还有临床医生来学习了跟别的导师合作呢，他把他的硕士生介绍过来，在我们这边是做实验做科研，我也会去参加他们临床的硕士，说他们其实做科研做的也蛮不错的，好像就是人一个这种我也考虑，但是本质上来说未来只有这三条路可以走。

01:06:13

对，其实学校做老师可能还是反正也不简单，去南医大也不简单吃饭博后期间，所以其实比较你也是会愿意去读博后的。你和师姐交流了那么多，交流了这么多，后面我发现要看看能力，我认为不管你是读硕士读博士、读博后，你是要看自己的能力的，如果我的能力能够够得上去读的话，还是愿意去读，我发现博后其实还蛮不错的。

01:06:47

但是如果你的能力确实不够，没办法，如果只是进去，你勉勉强强进去了，然后在里面能力就是指能不能出，他需要你出的成果就是读博读博后他对你的要求能不能达到？对吧嗯？

01:07:03

我感觉包括很多，因为我师姐她做博后感觉可能她我说跟别的不一样，她他是我们实验室唯一的博后，既发生博后他又担任大师级的职务，他还要带新来的同学，然后给这些同学指点迷津，包括我这样的本科生，包括研一研二的这些同学，包括很多博士也在他的指导下做实验，这样才能让整个实验室一起他说，他跟我说这样才能推动整个实验室的往前进。

01:07:28

他说他以后他就一直灌输我这样的道理，他说以后你做到这个位置的时候，你也应该要像我这样子去就是这样。所以说我认为我如果以后没有这样的能力，我去做博后，我没有那样能力去解决我的师弟师妹的一些学术上的问题，我没有办法指导他们前进。不管是自己除了自己的问题之外，你还要帮助整个形势整个团队一起进步，我认为这样才是一个比较优秀的博后。

01:07:54

你不能纯粹就为了发科研，就是纯粹就为了发文章那样就显得太可以，所以可不可以说你包括从大二到王老师实验室到现在，其实跟你的师姐交流比跟王老师要多一点。

01:08:11

嗯多很多，我认为这个也是我的科研的领路的很多，我从来没有接触过科研，就是一点想法没有，到现在就你受他影响还是蛮非常大，非常大我感觉我的甚至科研的风格可能就是跟他都可能有七八成相似。

01:08:25

以后你可能本来对可能对于免疫兴趣，你觉得跟它相关还是可能就是因为跟它相关的，因为它很那时候我什么都不懂，他就是深入浅出的去跟我们去跟我讲这样的一些问题，并且他他他认为那时候他觉得我还有一些能力，觉得我的思思维跟得上思考的速度跟得上，他就跟我说要站在他的角度是怎么去思考的，就是去做科研怎么样去去切入，这个可以，我当时觉得确实还蛮不错的。

01:08:58

可能也就是因为第一个不错是指这个方向蛮不错的，包括这样去做科研，我认为跟我的本质上的初心就是比做临床更有意义，更有意义我认为。

01:09:13

更能够推动当然说的很大很空啊，更能够推动医学的发展，但是最后怎么具体做到转化上，我们也做不到转化现在可能这也是一个原因，可能最后就是退出活动，因为可能我去的第一个实验室和第三个实验室可能阴差阳错没有这种手把手带我的师兄师姐，包括我去第三个人民教师，实验室大家也是他也没有给我安排专门的师兄带我，他就让我去，因为他们实验室很小，他们其实是比比普通的实验室要小，他们那时候刘刘，老师是副教授时候拿的实验室，所以就只有一个小房间，大家都坐在里面，大家都做实验，他让我随便去看随便去问，但那个时候不知道为什么那个时候还比较羞涩的人还比较羞涩，就是这些他不主动给你发话的话，他们在那做实验，我还蛮羞涩的，不敢上去跟他们搭话。

01:10:02

你怎么又突然那么艰难，我不知道可能跟师姐聊多了，对可能跟师姐聊多了，那个时候就不太感恩。因为我刚，所以其实在大二大一包括高中，其实你整个人还是不太不太敢和别人很多交流那种。就直接改了。其实我大家都你觉得做到学生会应该还是算我那时候都差点做到学生会主席的位置了。时候我。对我辅导员让我当学生会主席，我后面直接跟他说我拒绝我要推出去这会了，因为那时候就是要去一般这种人都是比较能讲的。

01:10:35

确实你为什么当时比较不知道那个时候感觉，但是平时的自己比较羞涩，不是能讲你要懂一些东西你才能讲的出口。但是如果你什么都不懂，不太好意思说，对不太好意思说，我就感觉有时候可能我的问题会会不会比较智能或者幼稚，或者会引起西红柿这样的不满意。

01:10:56

就说那时候在你们渠道实验室确实也跟过几个，也跟他们聊聊过，但是聊得没有那么多，也可能是时间也有时间的原因，那个时候。

01:11:08

那个时候确实去刘旭老师也是没有那么的多，刘旭老师还对学生比较轻松，他也不怎么管。时间可能休息的时间比较多一些。所以其实其实那两个实验室可能对兴趣的发掘也没有什么太大的错误，对，说不定去了就是逛一逛。好像也没有什么实质性的对作用。假如说他们真的有一个别的师兄师姐去带你做的话，你可能会发现有精子发生还蛮对，但是你当时去的时间是对镜子发生这些东西，其实并没有很深入的了解。

01:11:43

对。所以说这就倒是阴差阳错，你觉得这也是一个有可能是的也说不定对，因为你你我认为你没有在真正深入的去了解了一个领域之后，你就谈不上兴趣，你这些都浮于表面的东西你就谈不上，我跟你说其实大一那个时候你去报国重，其实主要还是为了就业，就是5+1+3，对，那时候为了5+1+3，真的对升职有什么机会好像或者对科研有什么好像没有谈不上的，因为那时候你都没有接触科研，你都没有接触过孙子，你都不知道国家重点实验室你什么都不知道。

01:12:17

那时候就知道每年给1万的奖学金，保研不错，然后还有5+1+3这个制度不错，然后就选择进起码比留在自己的本班级。基础学院基础医学班级要好一点，基础医学没有什么好的待遇吗？其实对于这一点来说，其实大部分同学会不会你自己感觉，比如你说要准确，就你自己的感觉，周围同学是不是主要还是看中保研和奖学金这两天才来的？

01:12:42

是的，大概我的感觉就是这样，真正的对科研感兴趣，其实可能同学有也有吧，但是肯定没有那么多，因为我认为他们接触的甚至没有我接触的那么的多，他们存的可能大部分都是为了第一听到能保研，大家谁也不兴奋就赶快去报，而且还有奖学金，而且额度还这么高。

01:13:02

包括我确实了解到有一部分同学国中的我说国中的有一部分同学，他们可能真正抱着当时我跟你说的想法，他们就是为了去国中拿一个保研的名头，然后本科结束之后再去别的学校去更好的学校，也不是为了做k他们那时候他们那种我认为可能就成为了就是学历。你听听上去上交的上交的博士听起来很厉害，他们可能就是这样想法。

01:13:31

你觉得这种想法学生会比较多吗？

01:13:33

我认为很蛮多，感觉周围的很多，包括我现在这样，其实我现在这种我周围都有好多同学就是熟悉我这样一种情况，他们都劝我，包括现在因为马上就要保研，马上9月份是10月份就涉及到我的就保研了。

01:13:47

他们就说你要不再考虑一下，因为我跟周围很多人说，我说我以后要留在哪里，大要留在网上，他说你要不再考虑一下，他们都让我以我这样的现在这种科研的质量对区块上交应该是没有什么问题的，甚至可以冲一下北大清华的免疫还是非常厉害的，说明是清华的免疫学是非常强的。

01:14:06

补充老师那边有没有心动过吗？我其实有行动，但是后面我后面我仔细思考一下，这就很复杂了，这有很多的问题了。总结起来就古人那句话，宁当鸡头不当凤尾，我去了，哪怕我能去到清华的一个很好的老师的实验室，我能够保证我在里面托养出我的能力，能够他们清华本科生自主培养的，他们也就像我一样大一大二就进实验室，然后一直带到大五，在留在实验室读硕士。

01:14:39

然后还有我这种出版一大读了5年，在保研区的那边读硕士，我根本竞争不了他们老师师兄师姐对他们都太了解了，他们是实验室最了解的了。所以我可不可以把它解读为你可能害怕，我们的前提是你可以去，你比较害怕可能去的，比如清华的免疫，可能他们对你不是很重视，因为他们对你不了解对，因为我认为科研这个东西看老老老师对你重不重视，这是一个很大的，你做一个你去做自己的感受，就很大的一个组促进力或者是主力，如果老板对你不重视，你就是助理，如果老板对你很重视，就可以促进你更成功或者更直白一点啊发更好的文章，这是一个很大的因素我认为。

01:15:27

后面我都跟周围有很多同学解释，我说上交的你包括清华的博士你没什么了不起的。

01:15:32

我认为做我们科研的，你就看你发了多少份的文章，看你是哪个学校毕业的。你如果南大毕业的博士，我假设发一篇nature和你清华毕业的，你就发一篇五六分的文章，大家谁强谁弱，一目了然，不是后面就不看学校了，你就跟他们讲过，就是说你刚刚跟我说的导师对你的重视程度可能会影响你对我跟周国很多人讲过的，因为他们他们是因为他们并没有很认可。

01:16:00

对，因为他们还没有做过科研。我还是认为他们就是纯纯的，还没有他们都不知道什么是基础学，我认为他们以后包括他们现在考试，我见很多同学，他们现在就涉及到考研，考那些劝你的是基础医学的还是活动的？全部吃吗？都是区外校的都有，对。其实按理说国中的那些学生，他们应该还是算比较了解科研的，他们当时有一部分可能没有那么深。

01:16:29

对，有可能是因为我跟师姐聊的太多，那时候熟悉起来，我跟师姐一直很熟，他们可能没有这样的机会。还有一个就是他们可能本身也不是，因为我本身也有一些兴趣，其实也不是说完全也想做一些自己的属于自己的东西出来，我认为这样是比较令人当然这也是师姐跟我说的，所以我也是按他想的，我觉得这样是一个比较令人敬佩的东西。

01:16:50

你还是要想能够做出一点属于自己的东西，创新性的东西。

01:16:54

在科研领域还是要有一定的接触，我认为才是比较好的。但是可能他们因为每个实验室的风格不一样，我不知道这些同学在别的实验室他们是经受怎么样的科研的训练，他们对于这种的认知是怎么样的，我就不太清楚，我跟他们平时聊天的时候，他们有的时候会说可能要去上交负担。

01:17:15

你刚才想说基础医学的同学考试都怎么样？他们比较关注考试成绩，对他们就是纯粹的就是为了读研究生去考研，就为了读研而读研，对，我说你们从来没有考虑过你们喜欢什么方向，你们现在都还没有定方向，你们去做过这些吗？

01:17:31

去做过肿瘤去做过，就是升值，你们就往这方面考，也不去了解这个实验室怎么样，这个老板会不会言必同学会不会对同学很严格啊，从来不去考虑，到时候我们先考虑先去把上交考上，先过了初试过了复试再去考虑这些。

01:17:44

我说到时候你再去考虑这些也太晚，了吧也太晚了。我有时候都替他们感到就是因为，我我相当于大一就在接触，我都觉得要了解很多的东西，才能够有资格去读个研究生。唉当然这是可能了解太多了也不好。像我们首先是对每个实验室新招的部分压力很大，噢你想做的更好，你的压力就很大，你就会很辛苦。你看新来的研究，他们其实也没有什么技术，他们肯定当年也不了解这些，就随便报一报名。

01:18:13

报进来，对考进来的好多还是调剂进来的，都是这样。还有所以其实这种压力其实是自己给自己，你要是真的是对懵懵懵懂懂的进来，其实也还好，你也可以甚至还可以懵懵懂懂的出去。

01:18:29

我后面就认为是这样的，就是你每个人对自己的要求是不一样，你对自己是什么样的要求，你自己有什么样的有些人他甚至说他就是纯粹为了混一个文凭，他又不要求发文章或者不要求做一些课题，他或者比那是用自己发文章带他毕业就好，他只要顺利毕业要有一个学位的话，我认为可能应该在实验室过得也很轻松，所以你觉得现在比较累，比较压力比较大，其实来源于你希望自己能够做出一个对做出一个创新的东西，或者换句话说我就是以这个世界为目标，我希望能够达到这样的水平，因为他也希望我能够达到他这么全部身心，我认为是全心全意的在带我做。

01:19:06

他很多硕士很多博士也没有像我这么大家这么全心全意很多问题给我解答。所以我以他为目标，我就希望能够坐在他家，所以要求自己也很累，我就跟他同进同出他什么时候到实验室，我什么时候他什么时候走，我什么时候走。他这样全心全意带也会是给你带来更多感动，还是你觉得更有自豪感多一点。

01:19:28

这种我感觉都有，但是这么多很感动，因为我觉得没有人会去当然他也跟我说过这个事情，他这么全全带我，可能一方面还是想让，我当他的接班人，他觉得我这个能力还不错，以后因为他毕竟博后做完，他也是要毕业，他也不可能有他跟我说他也不可能永远留在王石这个实验室。

01:19:51

然后他说然后我不是要留在王石实验室，可能以后就希望我能够承担起他现在这样的地位，如果以后后面的同学有什么不懂的问题，希望我想像现在他教我一样去教他们去把这些东西传承下去，按现在你的成绩，你可以进保研的名额没问题。

01:20:07

没问题，是吧。你也不需要再去重修刷分之类，重新说你没用有用的有用的，我们自己是有179 18级之后就再没有从18级开始不用我们是有用的。所以我还真的要去做那个，因为我大三整个一年我我不跟你说，我为了去做实验，我其实很多课都没有上，那年成绩不是很理想。

01:20:27

然后包括他们现在这些我们寄出去的同学，他们有时候要去保研的就在一直刷疯狂刷，噢他们也不去实验室，因为他们从来也不做科研，他们就只是为了成绩好看一点，就这样竞争，其实跟他们还是有跟他们竞争还是有一定的压力的，但目前来说你成绩应该没有问题，只是现在就一个学期了对目前还好。

01:20:48

目前还可以目前但是当年第3名现在已调到第6名还是第7名了。但还是在前班，你们是前面20名，对10个人报10个人有10个名额，我们有10个保研名额。然后当然我这个学期你们30个人保10个，我是不知道20%我不是，50个人啊，我们20%跟预防医学是一样的。所以目前来说你还是可以保研了，你还是因为这个师姐对你比较关照，所以你还是比较想去现在这个实验室，包括当然我也除了观察之外，也要考虑一些现实的东西，实验室方式，王石的实力还是挺强的，能发一些高分的文章。

01:21:29

难道我们去实验室就是看能不能发一些高分文章吗？你现在这个实验室能够达到我的这样的要求，我就觉得挺好的。就留在这里，王老师也对我，王老师也对我挺重视的，也挺喜欢我的嗯。

01:21:45

所以其实更多的还是因为想要去做免疫这个方向去检查，然后之后去退出的。对退出了，我知道对，我刚刚当年退出的时候跟胡笑确实说的是对生殖不感兴趣，到后面我又做了一年多，那时候就已经退了好久了，我后面思考。其实我也没有深入了解谈什么兴趣呢，这个可能也是一样的，但当时可能确实是因为对免疫更感兴趣。

01:22:09

你当时退出还要跟校长聊天吗？也申请了。

01:22:13

要申请申请给他发了邮件，然后后面又让我去亲自跟胡校发邮件，然后后面要去找黄晓燕老师跟她说，然后他就马上把我说的转发给股票，所以你们因为一个退出的都要跟胡笑聊天，因为第一几届总是要受众生他要考，因为这就是无效办的，他要知道我们为什么退出，下一节可能就是后面几页就会发生反应，他反正后面王老师跟我说的，他专门又给王老师打电话的，因为后面说我要留在网上实验室，我要做免疫，我不做甚至反正我是会做互相给王思打电话了，是打电话是出于什么目的？

01:22:52

是说你我好培养大家以后不要再从我家挖人了，还是我具体的不敢，我这个都是大佬之间的话我就不太敢问了。你觉得他跟你讲这话的时候是开心的还是什么情绪对跟你讲不太开心的，但是我应该理由应该走得再充分一点。

01:23:17

他当时好像明年话里的意思是让我理由再充分一点，这样可能互相会怪他，然后他肯定觉得他觉得胡潇在怪他，他可能有点这么觉得，因为他确实也是升值的兼职平台，他理论上来说升职也应该做一部分的，只做不好，我们做的不好我们要承认我王是主动过程，你也有跟胡笑讲过，甚至免疫我从来没有讲过，我只是讲的那时候就是说我的身体不是很感兴趣，对我对美丽，然后那时候我我还讲了一个理由是那时候我家不是四川的，我说有可能也是一个运输，那时候其实那时候王石还没跟我谈刘备这个事情，那时候我还是想的，然后回家在成都这边或者是回川大或者是再找一个高校，因为这边我还是感觉离家太远了，多少还觉得不方便。

01:24:09

那时候还没考虑过留这里的打算，我跟胡耀说，可能我没办法再留在国中去读5+1+3，有可能硕士博士阶段我要回家在家乡那边读。不在南大继续留了。我如果注定要本科毕业就离开的话，我就不用再用保研名额把保险名额留给更有需要的同学。后面这不应该想做一些现在没想到一开始说还是留在那一大堆，晚了，我觉得不知道怎么面对，回去可能已经忘记了，他都要做校长，都不要做校长由你说的。

01:24:40

不会，因为我需要可能会关心我这种小门，我就读研究生读博士，应该接触都接触不到他了。

01:24:48

这个学生好像跟他谈过话，你们别的同时退出的，另外两个同学你有跟他们聊过吗？我可以答应他们聊过了。你跟他们聊过了吗？我跟其中一个聊过了。

01:25:01

我跟我第一个，因为我是第二个，我跟一个女生聊聊过戴月，他跟我说过了，我们第一个推出的都不是我网页，是我们专业的第一名，那也是一个传奇人物。你没有跟他聊过吗？你没有是刚刚进来那个女生跟他聊，但是他的聊天资料我还没有整理，他表达的跟我表达的主要思想好像是第一啊。他是想把保研留给其他的学生，对因为他太强了，他第一名他永远的第一名。

01:25:29

噢他转专业，他转专业也是第一名，他是第一名，他一直都是第一名，他只转专业他没有去转专业，但他一直是第一，他不转专业，他对技术比较感兴趣，我感觉他当年真正他都比我还热，我都认为我已经算还算比较对技术比较感兴趣，比较热爱，当然也有可能是我没有转到临床队，它是真正可以转临床，还是对技术他放弃了转专业，然后去了国中股东考试面试还是第一名。

01:25:55

所以我发现他其实跟你也差不多，对甚至不感兴趣做。是吧？我不知道你跟他聊天感觉他就是这么感觉，甚至他当年跟我说可能他确实很厉害，那时候我们都还就觉得股东说的对一个每个人都保研，没有每个人都有1万块挺好的。他就说不可能的。就是我感觉特别低，他说怎么可能每个人都不一样，这肯定是有问题的，以后因为我们第一届可能给我们这样一个后面说明制度就会改变了。

01:26:23

你先不要相信这个，你想好了就关了，他就进去一两个月就退了。我还坚持了一年，他没有轮转完，他就轮转了一两个实验室。但那时候他也已经早就加入了，我跟他聊过，我退之前就跟他聊过，我说怎么选择退？

01:26:37

他说他嗯那时候在那时候在郭旭江老师的实验室，郭江老师古总已经我认为可能算比较厉害的老师，就是跟我们上主备课，那时候跟我们上主备课，然后我们认识他的，他就在挂失的实验室，他觉得他可能我认为是真正深入了解到深圳，然后觉得不是那么感兴趣。

01:27:00

因为我刚才不是说技术学院，其实很关注我们这一批在国中的学生动起来，偶尔还找我们去交流，所以说我们有什么难处，我们大部分人招了8个，有8个还是9个同学，我们七八个人都是说我们真的没有时间做，我们就是太无聊了，就像自习室一样，我们就去这件事，他就说我时间真的太多我好累，我好辛苦，他就已经提前已经很深入了，他因为比我们我现在认为他都还蛮厉害的，他可能就真的深入去了解的时候，发现不是那么的感兴趣，他会发现甚至比较局限，因为后面还是我后面找好像有一个老师忘记是谁了，他们跟我聊天的时候他说他说后面我发现他说后面他说说不定基础医学的同学可能比国中创新班的同学要更有要更具备一点优势。

01:27:54

他就说深就是过甚至过中的同学，因为他后面的课程设置也是偏升值的，删掉了大部分的课程，然后当时一起出去，课程很慢就，所有的课都上。

01:28:06

但他们有一个优势，就是他们范围很广泛，他们哪怕每一个学科他们只学很前线的部分，他以后他们的选择就很广。

01:28:14

他们如果以后读研究生，他们可以选择去读，甚至他们也可以选择独生子，也可以选择去读病理、读生理、读免疫，他们什么都懂一些。但是如果像我们他当时说那时候我还在国中，他说如果像你们股东，你们大部分学生什么内容，如果以后你们再想从甚至那个时候就读到研究生阶段，你们感觉可能不是很感兴趣，在想，这也是我推出一个原因。

01:28:35

你再想改变就很难了，因为你接受的所有的训练，就是所有的教育全是甚至相关的，然后你再去研究生阶段再想去做免疫，别人为什么要收？你为什么不收一个他对免疫还懂一些的同学，所以说有些这个话是他对你说的，对一个老师都说后面我觉得他讲话还没有道理，这可能也是我就是推出国中的一个道理，那个时候还在国中。

01:29:00

对，那时候采访的时候老师跟我说，所以其实去了甚至可能对你以后未来的可能性会限制，对限制一些可能性，但是这条路好走一些，这就是看个人怎么看嗯。

01:29:14

对我还有一个同学就是一直坚定的留在国中的同学，是我隔壁的室友，关系特别好，他一直留在刘明希老师实验室，他就是说他就要一直做身子，为什么？因为他就觉得我也不是感觉他就觉得这条路好走，因为他他说他家就是这边江苏人的，他觉得留在南医大也挺不错的，南医大可能在江苏这边的知名度还蛮不错的。

01:29:35

他觉得与其去上交复旦这样在也是相当于赌博选导师，他不如现在就把一些就是导师选好你你们，导师他觉得选导师像赌博这种观念是你跟他传导的吗？还是他自己本来就他有这种观念？啊我们俩观念是一致的，我说我也是这么认为的。

01:29:53

我在网上看有什么问题。这确实有点像赌博，因为你不太了解就是外销的导师。所以对于他来说可能说对升职谈上兴趣好像也不大，只是说5+1+3这个模式非常吸引，他们认为这种做了两年对安安心心的安安心心的读到博士结束，他说以后要么他说，而且国中当年也这么跟我说，甚至要招收老师，也是优先从我们国中毕业班的同学找，但是他说他想一条路走到通以后直接流到南京，他当老师，他这样就不用没有考虑任何的东西，但这个也路也好走，他不出意外可能就是这样，你就业的话可能也不一定在南医大。

01:30:30

可能你现在还会想回四川吗？也会封闭可能性。也不方便，但是没有原来这么的想，对，原来我其实是这么想的，因为大一大二的时候，那时候我真的感觉太远了。

01:30:41

而且当年确实填专业真的填错了，我其实分真的可以去读川的，我当年读川大好了也不读一，那时候觉得太辛苦了，读医学太辛苦了，天天上课，然后去实验室太辛苦了，现在怎么又觉得无所谓呢可能现在有一部分兴趣，还有一部分当然了没办法了，你既然没有办法改变，那么就只有把在做，而且也了解了一部分，包括川大的基础医学，他们做的没有那么好，我也跟很多遍，我真的我也聊过了，我问他们，我说你们觉得四川大学怎么样？

01:31:14

他说华西好华西是不错的，但是只是针对他们的临床和口腔，他们基础其实做得非常一般，待遇也不是那么的好。

01:31:23

其实属于你说老师的待遇，如果我以后就业想回到四川的话，老师待遇不都一样，学校里。不是相当于你说是平台，他拿到课题的这些不是是待遇。我的四川大学里面老师不一样，待遇还不一样。不难道不是看你就是引进人才的力度不一样，引进人才，比如说王石跟我说他的师弟，他师弟发了一篇sale，就是震撼的一座，然后想那时候在国外想回国，在国外的博后发的想回国当老师，第一个选择四川大学的技术学院，然后先说回来就给他什么20万还是30万一年，再加一点点几十万的启动资金，他说还比不上南医大的力度，这也是我考虑的一个因素。

01:32:09

现在没有那么强烈，也没有那么看以后的怎么发展，再说对留留回不回家乡都可以。想的比较远，想的太远了，每天想太多了。

01:32:21

我那些同学大部分同学可能只想明天吃什么，我已经看到10年后怎么找工作了。你个性是从高中时候就会有的？好像也没有没有，我高中就从来没有，我高中我随便学，对管学习。上大学开始我，高中也不太学习，是因为高中也不太学，高中我就纯玩。玩玩。那就是天生的聪明。

01:32:44

好像都是所有的这些人都是这么评价我的这些老师，他觉得我这个人还是有一些冲击，不是很努力。然后高中的时候，所以然后最后高考快要高考了，还有几个月来复习一下，单考的也不是很好，我平时不复习，我认为都考都要考，就是南大的水平，后面是考了个川大的水平，就随便选了，而且选了一个南医，大进来进来之后很用功非常用功。

01:33:08

因为可能是大环境大家都在用过，我也不知道为什么。基础医学都想转专业，对那时候大一的时候大家都蛮用功的，那时候考试月的时候就是第二天要考试了，大家基本上没有在3点之前睡觉的，很多女生都是通宵的，我也是受这样的影响，后面我很努力，但是一般说明后面一半我又对一些我认为可能那时候太膨胀了，我觉得物理啊化学这些都太简单了，我就随便看看就去考试，所以考得不好，这也是我没有成长专业的一个点，我的主干课特别好，但是我的一些物理化学数学这些我就随便听一听，随便去考也没怎么重视，所以就考得不好。

01:33:44

因为但是他转专业是要求所有的科目一起算综合的，所以就导致我的综合排名比较低。如果只算主干课的这种平均学分绩点的话，我还是在百分之前20%是绰绰有余的。有后悔过，但是转专业发现就差那么一两个，这就肯定后悔，但是我想我物理化学稍微好好看一看，那几门课稍微多考一点分数，可能现在又是不一样的，但是后面这个人还是比较少，但都既然改变不了，然后继续往前走，所以就导致我现在继续没有在接触这样一种情况。

01:34:22

总体上来说还是你觉得还算满意吗？现在一路走过来。我觉得还算不错，虽然有一些小的看比如说没转成专业，比如说你现在回想还是会觉得有点遗憾，对都有遗憾的，其实其他的事会不会来源于我猜了才说了就来源于父母。

01:34:44

对，主要是没有完成爸爸妈妈对我的这种要求，他们还是可能更希望一个临床医生，这可能是我一个我自己本人。

01:34:54

其实不是好，都还好，不当成其实对我来说并没有太大的影响，但是我感觉总体还是蛮不错的，因为很幸运遇到了很多很好的老师，我觉得这老师我去轮转的这些老师都挺好的。然后去的这几个实验室氛围都很好的，我也遇到了非常负责任的世界，一直带着我，也有机会进入国中，我都觉得已经很不错了。

01:35:20

国中的平台确实我认为国中平台确实比技术学院的平台可能还要高一点。高在哪？实验室更转，接触实验室更多接触显示更多技术与学院，他们甚至可以接触更多的事情，你除了生他们也有生存的便宜也可以，有没有？你觉得他高是高在哪？他们有各种各样的机会，比如说可以带我们去参观生殖中心，然后会有上次还有什么院士来做报告，可以让我们去听他给国中很多的机会，然后甚至包括帮我们报名去考动物证这些。

01:35:51

他们都考虑的其实蛮好，的啊，所以那个基础医学他们基本上没有工程，不管你的如果你的导师要求，那就让你的导师给你报名，你自己去考，基础学不会规定。

01:36:02

所以觉得其实班里对学生的还是挺重视，对学生非常我认为非常重视，科研训练也已经做得很好了。你有没有学弟学妹来问过你？一个无人班的事情有的，他们问什么比较多，他们就这么考什么科目，然后面试的时候有什么问题，他们这个都是已经夏天的，原因就是要考的才问。

01:36:27

面试的题目，对考试什么考考考什么东西，其他的不怎么。因为我感觉他们甚至大部分而且不是基础的同学来问我，还是可能是临床的要检验，好像有几个护理的，还有几个没有临床医学，没有临床的，没有临床检验的检验的，有一个检验的，好像还有一个护理的，他们都是来问你考什么是什么题目，他们我认为可能大一的时候也从来没有接触过科研，他们甚至不知道实验室是什么样子的，他们就纯粹想进来考进来，进来了之后，然后再去接触科研，后面好像搞起来。

01:37:03

你要是说你要退出的时候和另外一个退出的聊了一下，你觉得和他聊了一下，对你思想有什么改变吗？还是说不变不变，后面我一直到后期的时候，我其实已经做好了这样的准备。

01:37:18

嗯就是退出。其实当时我们有两个同学，因为我们每次轮转其实基本上都是两个同学一起轮转到一个实验室，我那个同学是国中的也是国中的轮转，他跟我产生了同样的想法，也是想退出国度，也是我也觉得对深圳不感兴趣。可能是我具体不知道，也是想留在王老师这里，也是想留啊，可能可能这次后来胡笑可能骂了王思远，可能就这边在挖他的人，他最后没退，他退了吗？没退。

01:37:50

最后没退，因为可能考虑到，因为首先他成绩可能不一定拿得到技术学院的保研名额，他在国用更有可能冒烟。对。所以基于这一点，他可能最后就没有对。然后他好像是他去帮我问的，能不能不做生殖免疫的方向？本科毕业，他可能不行就不行，然后他就很苦恼，他那时候很苦恼。其实他可能也不是很想做生产，对可能是，但是他还是想他可能还但是还是保研占大头吧，因为这种他觉得可能太难了，再去基础医院再争一个保健品可能太累了。

01:38:33

然后他就然后就考虑了一下，然后他选择你看我们实验室去别的地方做，甚至我最后就选择退出过程，再留在这个实验室，成绩比较好，可以比较任性一点，跟着自己的兴趣但是也不能叫这样，但其实我是真正考虑了很多，我认为考虑的还蛮多，我说的任性是打引号的，我知道你这个意思。

01:38:56

他可能要首先要保证自己的基本需求，就是要保留神，你其实你的成绩，你的基本需求你都能达到了，我就可以去追求更多的东西，因为这样才能保证自己做得更好。可能兴趣因为你有这样兴趣才会往这方面做得更努力。如果没有兴趣的话，我不会一天十二三个小时都在待在实验室里的。你现在还会有这种累的感觉吗？就是待了这么久，还有十几个小时在实验室，现在感觉还好，感觉习惯了，他也就过去可能就是习惯。

01:39:31

当然如果当你实验做得很成功的时候，你觉得时间过得很快，一点东西你就带到凌晨两天，你都觉得没有什么问题你，现在其实已经有在独立的做某个课题之类的，已经开始对的，就是很早就开始做，这个是相当于世界分给你可能是网上通过网络上网，上是通过这就是在农村的时候，在后面其实也没有在做大创，在做王老师，对，我还专门问了王老师，我说你要不要参加一下大创，我可以帮你去整个团队。

01:39:58

我妈说没有不用了没必要了，后面想想因为独立负责一个课题还是蛮辛苦的，甚至是独立到连连这个小组都要我自己亲自去动物房，自己去配房，自己去和龙分龙。这蛮辛苦的，后面我觉得我自己也没有精力再去参加，别人他就是说如果你想参加可以去参加别的别的实验室的大窗，就是一个组员的身份去参与对对对？觉得我精力跟不上了。这个不太好。

01:40:24

我记得在和别的同学聊天的时候，他们有提到过进了国中班以后，其实他们是要求学生去退出各种各样的学生团体的，你有接触过你有听过这个政策吗？

01:40:39

好像是的，确实他们在大二的时候其实还是在学生会，那里面啊对对我认为，但他们这种退出我觉得没有必要，他们确实是这么跟我们说，但是我们觉得没有必要，因为他们认为那时候可能就是我们的精力如果放在学生工作上，很少能够放在在科研技术这块，但那时候我感觉我的经历还蛮不错的，而且那个时候我已经做到了。

01:41:00

其实学生会那时候我进了主席团了，我觉得我其实没有那么多的事情，大二你接没关系，我同学怎么接他？所以你大二的时候，我主要是让下面的部长去负责一些事情，所以说那时候我倾向于甩手掌柜，其实但是偶尔会做一些事情，但我认为这不影响我主要的精力。我没有退出，我推了一个我推了班长，我办的大一是班长在学生会，对。

01:41:25

所以其实你觉得对你经历其实也没有什么影响，对，所以说我没有退出一些老师也不会去查你，老师怎么会查你，还不当你才在学生会不当主席，我觉得没有，他只是说提了这么一个要求，对，我觉得提这个要求总还是想主要还是想大家除了今天可以啊都放在科研上，可能他想我们接受这些科研训练，正统的科研训练还是需要时间的，要花时间在科研上。

01:41:47

嗯那我能够推出很多，我推出了我们又当了班长，我从田径队推了出来，那是晚上学校田径队的，然后篮球队我也退了，应该叫社团篮球社团，天津社团。基础学院院长就对所以其实你进入国中班以后，你的生活的影响其实对我很多业余时间其实都已经没有了。包括你看我们也要求时候也要求我们每天去，然后我其实都是真正的上课，就除了必要的学生会开会的期间之外，必要的会议之外，除了上课之外，我基本上时间都在实验室。

01:42:27

我说我待的时间我待的时间已经比很多国中的同学都还要多一些的。他们有些还有自己的娱乐的时间，我都甚至连娱乐的时间我都把它取消掉了。

01:42:37

我就觉得很很会有这种负面情绪，但是没有时间娱乐，但是没有，因为那个时候我觉得科研还蛮满心满，蛮有趣的，除了那时候刚才第一个实验室，那不是去自习的时候稍微有一点负面性，我觉得去自习我在哪不能自习，我要坐在实验室自习，嗯实验室的环境也不是那么的好了，我在自习教室不是自习的更清楚一些，稍微有一点负面情绪，其他的包括像现在其实也没有那么多时间要在实验室。

01:43:06

对现在觉得这些这些还是兴趣支撑着你兴趣，还是我真的觉得搞科研没有兴趣，真的太痛苦了。科研如果没有一点一点没有兴趣，真的很痛苦的。其实最大头的就是感兴趣才能把科研抓下去，其他时间其实都是蛮虚的，是最重要的还是能支撑你走下来的，对，因为确实要花费大量的精力和时间的，如果你没有兴趣，你花费这么多时间和精力会给你带来很多负面积蓄，而这些负面情绪又会影响进一步的影响你去做实验的效率或者各个方面。

01:43:45

你看买确实我就是一个比较现在没关系，有的时候问的有一些学生可能会稍微内向一点，问着就因为我也当过访谈的，我去访谈pi啊噢，其实你很多的问题我都不用问，你自己就全讲出来，我就感觉你要问我，我先讲了，我都不用问你自己先讲出来。

01:44:09

其实我觉得也没有什么，我以为我觉得整个成长路径我已经全部都清楚了，对我的三项路径还很简单，我觉得没有什么复杂，就是思考的稍微多了一些，我就把我这些思考的东西说出来。对，其实我就是需要这些东西。O那就到这边，好的，你稍微等我一下，我给你拿一个什么？三天。还有餐券有消费你两个小时。55块钱。谢谢。现在什么时候？好，谢谢老师嗯。
