# Supplementary material for: Why do you choose this program?—A decision-making model of medical students based on grounded theory
Source: PLoS One. 2023 Sep 15;18(9):e0291634. doi: 10.1371/journal.pone.0291634 (PMC10503722; doi:10.1371/journal.pone.0291634)
Supplement: S1 File — (ZIP) [file pone.0291634.s001.zip › RAW DATA/P11.docx]

00:01

I want to sign the moral and ethical requirements. In this interview, the interviewees participated on the principle of equality and voluntariness. The process will be recorded, and the recorded materials will be used for scientific research in an anonymous form, and will not be disclosed to any third party. During the interview and after the interview, you have the right to cancel the right to use the recorded researcher’s recording materials. Are you Know and agree? agree.

00:32

Okay, then you can ask your grade and major first. In the senior year, there is a major in information basic medicine, and the 2017 basic medical basic medicine is ah. The first question we ask, I want to ask you when you were in your freshman year, before you signed up for the junior high school, did you go to the junior high school and then dropped out? Yes, before you signed up for the junior high school , What do you know about middle school classes and how do you know them? The freshman year is like this now, because before the freshman year, there was indeed news at that time, including ours, the counselors, including the national office. He released news that the country and even the state key laboratory would open a shareholder class, and he told us at that time. The publicity is mainly aimed at basic medicine and public health medicine, that is, preventive medicine. It is mainly aimed at students in these two directions of these two majors to go to do basic scientific research in the laboratory, because the freshman just entered, in fact Not so knowledgeable about our own specialty basic medicine either.

01:31

Then including the time of the first year, the third year to the back, the teacher of the three small schools gave a lecture to introduce them, including their national key cities and the innovation class they created is the activity class. We got to know them based on some news channels like this, and then of course, the publicity was very good, and the treatment was very good. At that time, we told us that after entering, everyone can guarantee research, you know? Everyone is still more concerned about the future issue, including the insurance research, including his scholarship treatment is also quite good.

02:06

And his requirements for this kind of entry are actually not that high, so after going to sign up later, what position did you get at that time? At that time, the annual salary was about 35%, because the name was embarrassing at that time.

02:26

This also involves why we chose the shareholder class at that time. When we were a little older, in fact, when we first established the technical school students, we followed the study, and then went to the school and went to the school to understand, we found that the technology I think it’s the same for my current classmates. In fact, in medical schools, I don’t feel that they are valued that much, because when everyone first came in, everyone was going to be a clinician, and we wanted to go to the clinic for surgery. To heal and save lives.

02:53

But later, most of the people were forced to come to basic medicine because of the adjustment, but then the school did not have any majors, and most of the students studied hard in their freshman year, just to change majors, From technology to go to clinical medicine, this is our purpose.

03:08

However, because there are performance restrictions when changing majors later, the first 30% and 20% are clinical, and 30% can go to pediatrics or psychiatry.

03:20

At that time, I also wanted to study hard, and then I went to the clinic and wanted to treat and save a doctor. Then, it was not because the test results were not too ideal, but I remembered 30% very clearly. The finalists for clinical transfer are still one meter or two meters away.

03:37

At that time, he was in the top 50% of the junior high school class. In fact, I knew very well that I happened to be in the middle of the two, so I went to the registration process. I would like to know more, when you applied for volunteering in high school, you were a science major, right? I majored in science, and when you applied to major in school, you applied for medicine in science, engineering, and medicine. I signed up for it all, because it was embarrassing at the time, and I didn't really think about high school too much when I was in high school.

04:08

After the end, because the test is not so obvious, my own performance is a little longer, and then I don't know how to fill in the volunteer, and then I ask who, we or ourselves or me. I discussed it with my father, because I am a candidate from Sichuan, um, the college entrance examination results were probably ranked around 7,000 in Sichuan, which is the 7,000th in Sichuan Province.

04:31

Then my dad asked me which city I would like to go to first. We first chose the city first, and then I thought that I would not stay in Sichuan at that time. It was true that I could go to Sichuan University at that time. I said I didn't want to wear it, I said I didn't want to stay in the market. I felt that Sichuan University was very ordinary, and then I wanted to buy things later. I felt that Sichuan University was generally still felt that I felt that Chengdu was very sorry for Sichuan University. Sichuan people may have been doing pretty well at that time because of their usual grades. Big, and then because many people can go to pairs, because I was in a relatively good high school at the time, and it was the best in the high school, and our average level was basically conveyed.

After 05:06

, I thought about visiting the cities on the southeast coast, then Nanjing, Shanghai, or Beijing. Later, I found out that the college entrance examination was really not ideal at that time. I didn't have the schools that I could get into. I can't pass the test, including NTU, including the statistics submitted.

05:23

Then later, then I will tell you what you like. We will fill in according to the intention you like. Sorry to interrupt, if you can go to Tongji Jiaotong University or NTU, what major will you apply for? However, I just never thought about thinking about it, right now thinking about school right.

05:38

Then it was really because the school was not easy to choose, it was not very easy to choose, and then we would think about it, and then my dad said that we would think about what major you like, but when I was in high school, I felt that I was a child. You can learn from me and me. In fact, I don’t have a specific goal to learn a thing, and then learn something by myself. Later, we just asked my parents and me, including his colleagues, including ours. Some relatives, including some of my teachers, some teachers, lawyers, and doctors, we asked about it from all walks of life. Of course, this is the strangest conclusion.

06:09

My elementary school teachers, junior high school teachers, told me, and high school teachers all told me not to be a teacher. Being a teacher is very hard. My uncle who is a doctor told me not to go to the clinic. It is very tiring. My uncle who is a lawyer That's what they told me, they don't support me and their careers to develop, and I don't know why. But at that time, because there was no one very capable, I was sure that I wanted to study a major that I liked very much. I applied for each one. I applied for one and also applied for my homework, including China Southern Airlines. In fact, I can read it at China Southern Airlines.

06:37

These are the schools that I applied to, because my family still prefers to seek stability, because when General Manager Gao applied for volunteers, they still have the kind of gamble, and they may go to some better schools every time. I usually follow the application guide for the college entrance examination. I see that these schools are recruited in Sichuan Province. I was 7 kilometers away at the time. I reported that their party entry line must be lower than 7000. That kind of school that recruits 6,000 students and recruits 6,500 students, I am a school that I can get into with 100% of my scores.

07:02

At that time, I applied for East China Politics and Law, and then it was difficult for China Southern Airlines to get big. Later, my dad said this, it seems that this school is not a 985 school. At that time, everyone thought that 985 211 was very powerful. He said that you have to apply for a 985 school no matter what. I said that anyway, I don’t need to communicate that I don’t apply for Sichuan, and then the 985 in Beijing and Shanghai were too good, and I couldn’t even apply. At that time, my grades were not good enough. Then I applied for Shandong University, and then I studied for a university. What kind of major is it? It seems that people choose it at random. Anyway, it was right at that time, that is, a random student of politics and law is not a liberal arts school.

07:32

We can also report.

07:32

We can also apply for science subjects like ours. Science subjects like science subjects can apply for liberal arts subjects, yes, but liberal arts subjects are not limited, but liberal arts subjects are subject to restrictions. Then I waited until later because the school was chosen during the later period, but the latter period was during the holiday, and then I added some circumstances in my family, my grandfather was seriously ill, and then we had some relatives in the hospital later. Being a doctor, but not very careful and clear, it may be a little bit related to the situation in the distant room.

08:01 After

that, my dad said that we should consider multiple doctors. He said that our entire family, including my dad's relatives, including my mother's relatives, did not have a single immediate relative who was a doctor. .

08:13

So in the end, I put the medical department in the first place, so I chose Nanjing University of Science and Technology, because the first consideration was Tianjin University of Science and Technology, because it seems that Tianjin Medical University is the only 211 in medical colleges, yes It was rated 211, but later I saw that the annual enrollment of Tianjin Medical University in Sichuan is about 5 kilometers and 6 kilometers. I don't think they can report.

08:31

He said that at that time, he knew that studying medicine was definitely a tough job. At least he had to study for a master's degree. At that time, of course, he only knew that he had studied for a master's degree, and then he would see which major would recruit 9,000 people in Sichuan. My name is 10,000, and then I am not 7 kilometers. I feel that I am a lot taller. Then I was in Nanjing again. I thought this school was not bad, so I filled in the first choice of South Nanjing University, and it was directly recorded by this paragraph.

08:53

So in fact, something happened at home that made you directly urge you to study. For you, in fact, studying science and engineering is medicine, and it doesn’t need to be so intense for me.

09:06

Later, I came to basic medicine. At that time, I felt that I had to transfer to clinical practice. When I actually filled it out at the time of admission, I sent this professional catalog. I don’t know what this foundation is. Our family has never asked about it. At first, we thought that what our family thought all along, basic science, may be the kind of general practice that general medicine now thinks. He does everything, it should be both internal and external gynecology and pediatrics. , and then clinical medicine may be a full-time operation or something. Anyway, we think that T-shirts may all be clinicians, and then we sign up first, and then find ourselves transferred to basic medicine.

09:39

Because I was really disappointed at the beginning, because that year my Antarctic University in Sichuan got 580 points, I got 620 points, and I got 40 points. Then there is another classmate who is in the same class. He is from Guizhou. He took the same national paper three as me. He scored 580 points in the exam, and I was paid 400,000 by him.

09:56

Then we were still the same classmates in the art academy, so you will find that after so much in high school, the final result is similar to others, and I experienced a big mistake. It was true at the time, and then my parents also knew about this later. , they all know that everyone knows it later, and later said that they learned about bleeding after coming in. He can't transfer to clinical in the future, including the master's level. We are not qualified to take the vocational exam, which is very restricted.

10:22

Then my dad said, and then later learned that the school does have a policy of changing majors. When you are a junior, you will evaluate your grades, and then change your major. Then my dad said that you should work hard for another year, and then Change majors. In this way, it was true that his goal at that time was to change his major to clinical practice, so in the year of basic medicine, in fact, your main goal is to study hard to change majors. Of course, it didn't happen this year. In fact, a lot of things happened, because I joined the student union at that time, and then I joined the student union and contacted the counselors. The relationship between the counselors was pretty good.

10:56

At that time, there will be a lot of competitions for us, and those facing basic medicine, such as big jumps and small jumps, are directly oriented to basic medicine. At that time, because most of the clinical work was better than ours at that time, because the technical colleges had not been divided out at that time, the freshman to junior year of clinical medicine was still managed by the basic medical school, which turned out to be a very large hospital. Has the credit report and biological credit report been separated?

11:18

At that time, our counselor thought that this event was mainly hosted by technical colleges, and even mainly for technicians. He thought that we should have a team of our own to participate in the Great Depression and participate in this kind of scientific research. s project. Then I had a good relationship with the counselor at that time, and he and I also served as the monitor and a student leader at that time. He asked me to form a team and participate in this kind of competition, and then I signed up.

11:41

At that time, I followed Mr. Ge Yingbin, a teacher from the Physiology Department. At that time, he had indeed begun to enter the laboratory and start scientific research.

11:49

Then I started to contact you to form a group. It must be a direction, right? Yes, the direction is chosen by yourself or by the teacher. The direction is given by the teacher. It was given to you at that time, right, because it was confirmed at the time that you didn’t know anything at that time, because you didn’t learn much at that time, that is, related knowledge. After entering, do you feel and imagine yourself? Will it be a little more difficult? still? But now after going in?

12:10

For example, things like this are still interesting. I thought it was quite interesting to do experiments. At that time, I thought it was quite interesting to do experiments. Then I did some animal experiments including these, and then modeled. At that time, I felt this Instead of telling the clinician how to clinically tell the patient, we are doing these operations for the group, and then we go to study these drugs or its mechanism.

12:27

I feel that this may have more profound and far-reaching significance, and then it can reach a point, because at that time, many people told me, including a lot of negative news in society, and now he is also bad for doctor-patient disputes. , It is recommended that the treatment of the doctor is particularly bad.

12:44

Including I sometimes go to the hospital to see a doctor, because I am a person full of keyboards, I chat with the doctor, and then include my uncle, that is, my uncle who is a doctor also does the same, he said that these doctors now It's really best not to be a clinician in a patient relationship.

12:56

They all say that your foundation is actually pretty good, better than being a clinician. If you do foundation, you will always be a scientific researcher, and you will be an obscure dedication behind the scenes. If you can never touch such things, you will never be There will be no danger.

13:07

So what he said was that you wouldn't go through some of those things that might lead to sharp contradictions, and they said it was very tiring at that time, but at that time, because I didn't think doing research was that much, I didn't experience research at that time. I was tired. Maybe the teacher assigned less tasks at that time. He did some experiments every day, and then went there every day to observe the group and change the medicine after the experiment. It was quite easy to change the dragon.

13:28

So after relatives like your doctor told you these things at that time, did you agree with what he said? Or is it an idea? I still agree. At that time, the balance in my heart was actually tilted a little bit. Even if the technical art was not so right, it was not so bad. In the end, I failed to convert into a major. It may be quite good to stay in the technical college.

13:49

But this kind of mentality is something that happened during the freshman year, yes, this is the second semester of college. But I have never mentioned this idea to my parents, because I think they have not been exposed to this kind of environment, and their cognition will not have such a change. They still think that clinicians are the most important OK, it should be done clinically.

14:05

You should then include people in our family in the future, including relatives and their friends. In the future, if you need to see a doctor, you should directly come to me. This kind of doctor is the best.

14:13 As a matter

of fact, you applied to change majors at the time, and you should not have enough grades, so when you realized that there was no way to change majors, your father and mother would still be quite disappointed, yes, they were quite disappointed. , but when I was a senior, it was actually the end of the final exam. At that time, when the grades were not released, I said that this summer vacation might have to count the grades, and then it might involve the issue of changing majors, so I told them For this question, I said that it may be because there are transcripts for every semester every year, because I can also see that I said that the rankings may be more hanging.

14:47

Of course, I told them about my thoughts later. I said that I would not be a clinician in the future, but actually doing scientific research is also quite good. Later, I changed majors without success. After I can’t change careers, I will talk to them. We talked about it, and at that time, the introduction of Guozhong was actually during the final exam, a period of time before the final exam of the freshman year. At that time, when we didn’t know whether we could transfer schools, there were activities to promote us. , at that time, I took the middle school as the second way to introduce it to my parents. I said, you see, the only one in our school is the upgrading of the State Key Laboratory. He opened a shareholder innovation class. I feel that this activity is very important. Generally speaking, it’s actually pretty good, and it may be in line with the path I want to take in the future. If I can’t become a clinician in the future, I will enter the activity innovation class. At that time, he also told us that the undergraduate, master, and doctoral program will give us such an opportunity. .

15:29

I mentioned that when I read a bachelor’s, master’s and doctoral degree, and then I read a doctorate, I feel that the possible employment prospects in the future will not be worse than clinical. Do they agree with what you said? I don't know if they agree or not. They have to agree. I think in the end they also because I don't feel very understanding. After my father, he had to do it. He couldn't, because I really couldn't participate. Here, it's up to them, but now it's quite recognized.

15:50

You just said that scientific research may be behind the scenes and will avoid such conflicts between doctors and patients. In fact, there must be some good things. For example, I don’t dare to tell my parents. Is it because I am afraid They are not very accepting, or they are afraid that they will interfere with your choice.

16:09

I feel that they are not very accepting. I feel that because they don’t know anything about technical colleges, they just think that these are useless, right for life?

16:19

He thinks that these are equivalent to assisting doctors in doing miscellaneous tasks. He thinks that the most important thing is to be able to use seven, hands, and pig knives to perform surgery. The real clinician .

16:30

You did the Challenge Cup at that time, but it turned out to be not so ideal at the time, because it didn't do it later, because there seem to be several reasons, one is the tutor, the reason is that we didn't make it later, and this one is not. I know if I can talk about it, because we were doing experimental animal models at that time, because everyone was a freshman and a sophomore at that time, we actually didn't have the certificate to go to the cave at that time, we made sales models, and there was a salesman in Kang Building at that time. The way to make a sales model for us, of course, have you guarded against Hu Xiao's seizure?

After 17:02

, it was sealed up, and then because his teacher told me this, but later I entered the middle school class, and then I also communicated with Hu Jia, he also mentioned it to us, he said that I think he once was For a walk, he went up from the side of the building, he walked, he walked to the work station, from the school to the work station, he went to that road, he was steady here, it smelled like mice.

17:20

He believed that seeing the property market for experiments should not have the taste of a group, a very strong taste of group survival.

17:25

Then he asked someone to check, and found that there is indeed a room for raising dogs, but this is an unethical group, and they should be raised in the animal room animal center laboratory animal center, so say Privately someone is supporting this thing privately, yes.

17:37

These are some things that several teachers did in private, and then they were closed down. We executed all the groups and sent them away. We worked hard to recruit the target for half a year, and then there was no more. .

17:46

, the teacher asked us to do it again. In the bridal room, we were asked to take the Dongzheng test at that time. Then we entered the eastern room, and started from scratch in the eastern room. It took a lot of time for physics, and then there seemed to be nothing in the back. What you do is the real result, and the teacher doesn't want to do it any more, including us students, some of the team is actually a clinical classmate besides me, and I look for him. Yes, clinical middle school, and then those clinical classmates later considered going to participate in clinical courses and going to the hospital for an internship, so this team was already a junior at that time, right?

18:21

They are juniors and I am sophomores. I'm older than you, yes, I'm looking for a senior and a senior. Then including that time, in fact, I was already in the middle school in the second year of sophomore year, and then Guotong also involved the articles of these laboratories, and it was necessary to prove that when it was said that the certificate of ethics should be verified at that time? Usually a master's degree. The master's degree test, yes, is generally the direction of progress for graduate students and doctoral candidates.

18:45

In fact, I have already taken the test in my freshman year. I can start very early. Why? For example, like the middle school class, in fact, many people will do experiments. They shouldn't have to tell the middle school class when they go in, when the middle school class opens, and when they go in, Hu Xiao also takes this into consideration. The middle class wants the middle school to receive the most advanced training in this kind of scientific research, because normally, including our technical studies and preventive studies, we can also do it later, but we usually have to go to the fourth or fifth year. It is our training method that will change to scientific research. In the past few years, we still learned basic knowledge, and then went to the hospital for an internship. After the internship, when the final project was completed, we would be asked to do some scientific research in the laboratory with our undergraduate tutors. Training, that's when the real progress is made.

19:22

Then, the country thought that the middle school class should be a little earlier, and the foundation laid in this way is older than others. That is to say, the middle school classes will take the test for this certificate in a unified way. In fact, those primary schools with blood in your clinic do not have this certificate, and they also take the test. This certificate can also be tested clinically. You are not saying that you need a master's degree to be admitted. In theory, it is right, but undergraduates can apply, teachers can help you sign up, you can go to the exam, and then you can pass. I just finished the exam because I hadn't entered the shareholder class yet, because I was doing major tunes at that time.

19:51

So in fact, you have analyzed the possible reasons for failure, and you may be willing to be a lot, yes, including some directions may also have problems, because at that time it may be just a few of us, and it is not because there was no one to take us at that time, because we all Be a real undergraduate student who has never been exposed to scientific research training.

20:12

, I entered the laboratory, including entering the laboratory in the middle school. At that time, some people who entered the laboratory were already familiar with this aspect, including some doctors, who were the senior brothers of experienced doctoral students or master's degree holders. Use the time point to take us.

20:25

At that time, when we were working on that project, we didn't have it. There were no brothers and sisters to take us. We just introduced to us, how to do the model, and the brother demonstrated it. When you show the group to him like this Perform surgery to remove criminals, and then he demonstrated us to do it again, and then the teacher took a tube of medicine from another place, we just wanted to study this medicine, one of the ingredients in this medicine, its effect on scar repair, He used this medicine first. He said you spit it out and apply it first, and then we will do the surgery first, and then put it up after the operation for this group, and then apply the medicine to him every day.

20:58

So what you mean by this passage, maybe your surgery may have problems with the experimental method, and there may be some problems with the experimental design, because this drug is for human use, can it simulate whether it is used in mice? This real situation, including whether the active ingredients of the medicine can really enter the cells, and whether the active ingredients can actually reach the wound is hard to say, and each of us does surgery for each person because of multiple models, we have to do a lot of work Dozens of them. Generally, you don't do it alone, or you want everyone to do it together. Everyone may do it differently, and there will be errors. This difference may lead to the failure of the experiment. Including the late teacher, his own energy is not on this, what we will do later, do cell research, what to do slices, and then do some experiments such as immunohistochemistry and immunolight.

At 21:42

, the teacher behind him asked his master to do it, so he didn't let us continue to follow up on this project, and finally told us that the project was stopped and we would not do it again.

21:53

Later, because there is another reason, it was done for the master, or it may be done for the master, or it may be done. If there is any specific work later, I will not ask again. This project is a failure. , then there is no further follow-up.

22:06

You just said to transfer you, basic medicine is not paid much attention, it is basic medicine, I think it is my own belief that technology in our school is also in our college. Basically, I feel that there are some things or this feeling, I feel that the school is not very good. Pay attention, technology is not learning, I am not saying that school is not important, it is nothing to tell you how you feel.

22:26

In my opinion, the first thing is that the society does not pay much attention to the society, because people don’t know what the technical college is doing at all, and then people who go to the hospital do not pay much attention to it. The clinicians, most of the clinicians I have seen are very strong and may be a bit heavier. Others think that the operation is the most important thing. You have successfully performed the operation. Scientific research, it is generally felt that they are still worse than them.

22:54

Well, including me and my clinical classmates, I also know some people when I go to the clinic. When I usually play football or communicate, I also feel that they don't pay so much attention to basic medicine. I feel that they are still Think clinical is the best.

23:09

Our school's clinical best basic medicine is indeed worse than the bank's basic scientific research. Of course, I myself think the same way, and I myself think so too, so maybe I think they look at us the same way. So at that time, you may have wanted to apply for clinical, but still did not apply, and finally chose to apply for Guozhong. The more novel pulp may mainly be Baoyan.

23:34

The training mode for undergraduates, masters and PhDs is relatively new, right? Because he was relatively new, because at that time and he was very famous, he was the only state key laboratory in our school. At that time, I actually didn't know what it meant by the division of the laboratory as a key city in our country. It should be said that it was still not very clear.

23:55

It was not until I went in later that I realized that although it was really powerful in the past, in fact, when you learned about the middle class at that time, it was mainly through the lecture.

24:05

Well, yes, through the briefing session, then the third primary teacher gave us a publicity, and then our counselor also strongly recommended us to go there, that is to say, it was even mainly for the basics and prevention. Therefore, the classmates around you were basically 17-to-17 classmates at that time, and the first-year class was more enthusiastic.

24:28

In addition to the students who changed majors came out. Ah, if they changed majors, my roommate changed majors to clinical, so they wouldn't apply for it. Then everyone else is basically full of bags and meets the conditions for them to sign up. We all signed up, and technical mathematics basically signed up.

24:45

So after entering the junior high school class, the sophomore year should be a scientific research rotation. For the junior scientific research rotation, there are 2 rotations per semester, and 4 laboratories per year. I want to correct it. Timeline, you, a big founder, may have started from scratch at that time, and then failed. The point of failure is the point of failure. Selling a point is at 18:00 at the end of the sophomore year.

25:08

is not in the middle of the sophomore year, it should be the winter vacation of the second semester of the second high school. During the winter vacation, I also stayed in school to do it, and then basically you still stayed in school to do it. Basically, I went home for the holidays after my freshman year, and I basically stayed in school. The summer vacation of my freshman year was also a test preparation activity. What's right, then in addition to the lesson preparation activities during the summer vacation of the freshman year, I already started to make big innovations, yes.

25:36

At that time, Daiso called this teacher, was your counselor introduced to you? Or did you find it yourself or how did you know it?

25:44

At that time, Daiso had one and they would send one about which teachers would like to have a big window, and then they had their own list of project pairs, and then we contacted according to one of the lists, because some teachers had their own teams, and he had Our own students, we then contacted us ourselves, and his direction at that time may also be of interest to you.

26:03

Yes, because I did see him at the time, he would say what we are going to do, he has listed all these directions, and then we go to see what we are interested in. Because I didn’t know much about scar repair at the time, I thought it was too powerful at the time. It's nice to be able to get the skin back to this smooth time, I think it's nice.

26:21

So you can tell me about the scientific research rotation, ah, is there something that impresses you more deeply, the scientific research rotation.

26:26

The scientific research forum in the second year of my sophomore year was actually quite impressive, because at that time, the scientific research I really came into contact with was not quite the same. Well, because I originally came into contact with Daiso, I didn’t actually go to the laboratory like this. , Every time we go to the laboratory to make animal models and redo the animals for sale, I think everyone in every laboratory may be like this, always doing sales, doing sales, and dealing with small animals.

26:51

Then I was the first to go to the first choice, Mr. Yang Yang should be Mr. Yang to do stem cells, because I was also very interested in stem cells at that time, because stem cells were also a hot topic in the society at that time, so was Mr. Yang I just turned over, pi was relatively young, and I went to his laboratory later, and I found that I had never seen novels, and they were mainly doing cell research, and at that time, they probably thought that we had just entered in the second year of sophomore year. Then I didn't have any foundation, and I didn't get a degree in the basic courses I took, and then I didn't have the foundation for experiments.

27:20

At that time, we didn't really let us do hands-on experiments. Most of the time, we were still introduced to us, including Mr. Yang's first laboratory. Mr. Yang was originally a pi who just came to Southern Medical University. It is a very young man, his laboratory is also very new, and many of his systems have not yet been built. First of all, they have very few people in their labs, and the time is very empty. Second, they have very little time, so I didn't see a few experiments at all, including a few classes at that time. , so maybe there were no ongoing projects in their lab at that time.

27:49 Right

. Or there are questions that just came in, but didn't involve me. It's a bit like a bystander, and even the content of the bystander is not that much, because I spend most of my time, because the basic medicine courses are very full during the day, and the class is in the daytime, and the class is at 6:00 in the evening, immediately shareholders At that time, we were required to go every day at 6 o'clock and then go for 2 to 3 hours. I basically went to two dishes every day.

28:11

When you finish class at 6:00, it doesn’t mean that you have to finish at 8:00 or 9:00 every day. I basically stayed until 10:00, but in fact, when it ended at 6:00, they had already finished all the time during the day. At 6:00, I I can only sit on the test bench, in the laboratory test bench, in the rest area, and read my own books. You would not think that comparison was useless at the time. I think the scientific research forum did not let us learn. There are so many, but Mr. Yang has a very good point, which is still fresh in my memory. We have two classmates to play together, he will assign us a task every week, and he will give us some proprietary stem cell fields. There are about 20 to 30 proprietary words, and then we will look up these meanings and make him a policeman.

At 28:47

, one of the columns is word, which is equivalent to one homework per week. I think this is a better way. I really want to have a deep understanding of the field of large cells, and then learn about scientific research, including such a way that can also improve my ability to check data. I think this is the only useful thing. At that time, it was like this Speaking of which, apart from other possibilities, I didn't learn any timing techniques. I even used a gun overnight, even with those instruments, I used very little, maybe I used it no more than three times in three months.

29:14

So most of the time is self-study in the laboratory, which is actually meaningless. You have discussed this matter with your classmates, I have discussed it, because it involves our next time, because the next time you want to choose me, I will ask him, I said how about the ones you usually choose? Sample?

29:28

Then a classmate told me, he said that some laboratories are very good, there are many experiments, they do experiments every day, and every day they take you and teach you how to do it, yes, they go at night I didn't have time, yes, and I did it quite late at night, there were quite a lot of them, and I was comfortable and very friendly at that time. They were very kind and enthusiastic. This would affect me in the future. A round-robin judgment.

29:51

So my second time, the second rotation time, was when I went to Mr. Wang's laboratory. Mr. Wang Xiaoming immunized him. He is a pi in immunology, but he is also on the list of pi in the middle school. He has done Part of the appreciation of the content. Well, I went to his laboratory at that time. I really had a lot of time. Is it very good? Yes, it is very good. I did a lot of things, and he will arrange it specially.

30:13

For example, if he has the opportunity, he will specially arrange his heart at night, in order to show us, in order to show us, for example, he has done some good things, and then when we see enough When it comes to quantity, for example, after you have watched it two or three times, he thinks that you may be able to operate some discarded samples, like our hands-on practice, and then after the hands-on practice, he can even really help him to do some work.

30:33

I will show you first, and then I will give you an arrangement at that time, that is, after I truly believe that you truly feel that you can make it, and then will I believe you. You have his own assessment, and he should have his own considerations when he feels that you have passed. For example, he occasionally arranges you to do some experiments, and he must have told me later that he has done it himself two or three times. The experiment has already yielded results. You're just asking yourself to repeat the result.

30:59

This is the first one, you have to repeat its results first, which proves that there is no problem with the basis of your experiment.

31:03

Then there are some basic things to look at as a person. For example, when there is no time on weekends, he asks to be in the laboratory at 9:00 am and 9:00 am to see if you are there on time. Do you not want to go to class? On the weekends, it is early on the weekends and in the morning, and then every class is also when there is no class. For example, when there is no class on Tuesday, Tuesday, and Thursday, it is also at 9:00 in the morning. The management of the laboratory is relatively strict. Anyway, after you come early in the morning, then follow, and then you can go when you have no time.

31:31

Then at that time I was still very interested, and then more active, I usually go at 9 am, if it is a weekend, Saturday or Sunday, I go from 9 am to 11 pm, and I will end him with the world. I do it at any time. Whenever he sits in the seat and reads the literature, I tell my senior sister, I said you want to post a few articles and I will show them to me first. I will also read the English literature with him, and I will see him. The automatic lab leaves at 11 or sometimes 12, and I leave with him.

31:52

Your senior sister is a graduate student, and your senior sister is a doctor. Now you are a postdoc and then you were a doctor. He also worked hard. That's because of this, and then I gradually gained his trust, and then I gradually began to let me do something for him. It was your sister who asked you to do it for him. My sister gave me to my world because I was playing. In fact, this world is fully responsible. After the past, there was basically no care for me. I am confused, what is there to chat, I speak directly to my world.

32:18

So it's very powerful and can basically solve all my academic problems.

32:24

Then this is about the second choice. I originally wanted to keep the third one connected all the time, but it was always connected at the time. This is right or not 4 times. I think the 3rd and 4th times are still choosing the time. , I stayed for the first time, but later said it was not good. The director of the middle school, Mr. Guo Yayun, told us that you had better choose separately. You should not stay in this rotation all the time just to let you see more. Guo Xiaoyun is Your Gu Gu Yayun is the head teacher of the middle school.

32:47

should still be the head teacher now. To me, I think what he said makes sense. Then I went to see it again, and then the third laboratory chose Mr. Liu Mingqi's laboratory. Some teachers actually went to the laboratory later and the results were still similar to the first laboratory.

33:04 Let's

see if you will report to Mr. Guo. For example, after I went to Teacher XX, I actually didn't have time. I think because at that time, I was very concerned about the junior high school at that time, not only the junior high school itself, but also because it was the first class and the first general manager. There will be all kinds of problems to be encountered, and they have to be solved, so occasionally they will hold meetings for us, that is, it is too little to ask us what problems we have encountered, and too little time for us, every time Every time we talk, we look at it for too long, and we should let us do it every time. We will reflect this problem every time, but we will not solve it.

33:34

Sometimes they actually can't solve it, because after all, it is the understanding behind me that he is someone else's research master or doctoral subject after all, in case you damage or contaminate other people's samples, For example, if you remove or contaminate a sample that only takes place in two or three months, it will also delay other people's progress.

33:53

This is really about trusting you enough. I also think that after he trusts you enough, you have to show the ability to help him solve problems before he chooses to go to you to do one thing for him, just Like the world of the second laboratory, he already trusts you very much, so he is willing to let you trust you for a while, so he is willing to assume that if you don't make it and get his results wrong, such consequences.

34:13

This is what he took the initiative to tell me in the later world. He said that he was willing to bear such consequences.

34:17

Just because of enough money, you feel that your ability should be able to complete this.

34:22

I think every lab probably thinks that way. The first pension is actually because they did start a lot of real laboratories. When they first started, they had to build some systems, and some mature experimental methods were not yet available. They may themselves, and those masters are also They are also new recruits. I went to the world where I learned about their graduate students. They only came this year. replaced me.

34:48

Then there is the third star teacher, who is still relatively mature, but the teacher tomorrow and Liu Qiang have a very good personality. This is recommended by my classmates and my classmates, mainly because it is very good, even You can also eat with everyone. I rarely see such instructors in the laboratory who can eat with students and say that it is effective, including playing games together. This is rare.

35:08

So Mr. Liu is still very kind and easy to get along with. His brothers are pretty good too.

35:14

But that time was similar to the first one, nothing happened, and it was possible during that time and because they were going to have a holiday, my second lab didn't have a holiday, and had to work all the time, and then the third one. This time is the real working time for men in the country. They have holidays every Sunday, and then occasionally there is nothing on Saturday, but I only have time on Saturday and Sunday, and I still have classes on Sunday and Friday, and At that time, the bank manager was still following up with Daiso.

35:45 I

have to do Daiso once a week, maybe some time, but I missed it. But overall the third is actually a little more than the first, but less than the second. So when you got to the 4th, did you ever think about going back to the 2nd?

35:59

That's right, my 4th is going back to the 2nd, my real route is the 4th, and then back to the 2nd lab. Then I continued to stay for half a semester, and the junior year and the rotation of the junior year were gone, and I quit the junior high school in the third year. Why did you suddenly quit? Because because I'm still in the lab, the 2nd and 4th rounds earned in the lab, I'm still in this lab, and I'm still at this time.

36:25

Then because at that time Teacher Wang, because of the two teachers Yang in front of me, the first and third labs, they were all actually doing reproductive-related work in essence, including some stem cells. One is for reproductive stem cells, and the third is for spermatogenesis? As for Mr. Wang, because he is a part-time junior high school student, most of him have little to do with promotion, and only do a small part of it. The content of reproductive immunity is a combination of reproduction and immunity. The direction of pure immunity of true immunity.

36:51

And the world he asked me to follow because when I first listened to it, he said that I should be the first to find my best student.

36:59

The first student opened the door to the big disciples. He said that I was looking for me to be the best student, and that was also his best student. I asked the senior sister to lead you. At that time, there was no such thing that he did not involve in appreciation. Kind of, I followed him to do it, so most of the things I did were made of veils, not involved at all, even then at that time, then back and then back instead of two rounds and two rotations, the back was chosen twice, they were behind and then I I will slowly do some immune things, and I will learn about it later.

37:24

There is Uncle Wang, and Teacher Wang later said that you are like this, because after all, you are a student in the middle school class, so you can still go to another senior brother, and you can learn a little about how reproductive immunity works.

37:35

Then I also went to see it, and then my brother taught me, and then in the process of doing this, it was still in Teacher Wang's laboratory, and the other one was to do appreciation. The brother is also a doctoral brother, and then I also followed him. Anyway, both of them went to see and see. After understanding, I did find that although I was immune in the laboratory, because it was not so mature, it was equivalent to the first period of time, because this was also us. It is also a direction opened up by Wang Shixin, so some experimental techniques are not very mature, and the experimental results are not so good.

38:05

Then it includes whether your brother is a master or a doctor, master and doctor, but his results are not satisfactory to Mr. Wang and you, and he has not made particularly good results.

38:16

Including me in stem cells, and then I went to Mr. Liu Jiang and I also read some literature, because every time you go to a laboratory, their direction is different, you have to read some literature to understand what they do. I also read some literature, and I also saw them do some things. I feel that they may do and even then even Guochuang took care of us. Let's take a look outside and take us there. It is an affiliated reproductive center for women and children. We went to see it and visited it.

38:42

Later, I felt that because I was a DAISO, I actually met a lot of teachers and mentors. I just communicated with these mentors and didn’t they communicate with a teacher? But then you have to choose.

38:55

I called right today, and then I just and I was not still working in the Student Union at that time, I was in the Student Union and then we planned a program, it was the Student Union of the Technical College. The Technical College planned an event, and he wanted to make medicine not a process Among them, the students of basic medicine know more about basic medicine, go to various PAs, and study these various directions of Hailou, including physiology, including pathology. I went there as an interviewer to make these students more interested in scientific research.

39:24

So I was right with them, and after the interview, I asked this question by the way, I said I would ask him, because I didn’t know how to ask them at the time, I said the teacher, he said you, this should be you In my freshman year, I was not only in middle school, but also part-time as a student union cadre. I said, what do you think about being a shareholder? Reproductive medicine, they say that genitalia is very good, yes, especially our State Key Laboratory. He has this name, and it will have a lot of funding. Later, I realized that funding for scientific research is a big problem. Only the funding group can do something good.

40:00

But some of their teachers even told me that they were too basic, some were too real. In fact, when I read the literature, I also thought about this problem myself. Most of the scientific research I think is possible. Of course, I haven't read a lot. I have my own understanding. Most of them study genes or molecules, and their impact on our diseases. For example, if he finds that this gene is mutated, we can't produce sperm or are infertile. After nurturing, he will study why he caused it, but it is difficult to do it.

40:30

Translational medicine is treatment, and treatment medicine can only be done through assisted reproductive technology. Assisted sexual technology also took us to the reproductive center. Assisted reproductive technology, but it seems that assisted reproductive technology is not our classmates in technology, because I have to consider who will say it first, whether it is done by other schools or it seems to be the kind that is also clinical. It can be said that they Also what kind of clinical technology.

40:54

So I can also say that you are actually a little more interested in assisted reproduction.

40:57

Because I didn’t know why I was very mature in my freshman year and sophomore year at that time, I considered the issue of employment.

41:03

Because I have always said that it is easy to find a job by going out through a mobile phone, I have been thinking about this issue. People who have been saying that people around me, including the teachers of our school office, say that if you don’t study for a master’s degree, you should study it. It's hard to get a job with a PhD.

41:17 When

I entered this laboratory, the second one is the laboratory where Mr. Wang is currently immunized. The intermediary also told me that technical studies mean staying in school to be a teacher? But at least you have to get a doctorate. The study time should be quite long. You are not like a clinical clinic. If you are really not good at clinical practice, you can also take the vocational exam after completing the undergraduate degree. Pretty good. Later, I was thinking about these employment issues and the whole future trend. In fact, I am indeed quite safe with the technology of auxiliary students. Later, I seem to feel that the auxiliary upgrading technology is more willing to have some clinical students with relatively stable hands. .

41:48

Then we usually stay in China. Of course, because of Tinder, it is very complicated. It is also divided into basic fruit seeds and preventive fruit seeds. It is divided into two parts, but we can all choose shareholder platforms, and there are also some It is preventive, but I see that the students around us are the classmates we contacted in the first season. Basically, only teachers from the basic medical school are selected. The classmates in their work stations will choose the basics, and they will eventually return to the work station. Union's laboratory to learn. So this involves a question. Most of the teachers on the basics side of his research are very good. Have you ever thought about why you need to choose a position teacher, but in fact, you must choose a technical teacher? I never thought about it, I want to think about it, I really have discussed this issue with my classmates. It is very strange, because the union is because in fact, on the surface, everyone may end up doing similar things, and in the end they are all engaged in Scientific research, but the nature of our learning is not the same.

42:36

What we learn is different. The trade unions may sometimes pay more attention to some data analysis. In fact, we have very little bioinformatics, including some of these integrated large-sample epidemiology. It is to conduct research in the direction of queues. We are doing small samples. I feel that we don’t understand them very well, but they don’t have such troubles because they later learned experimental techniques based on our foundation, because they also want to do experiments. Yes, they are here, so I think most workers have 4 rotations, and one or two come to the technical side to choose a mentor, and then do some experimental techniques. Anyway, I think most of them will be right. , In the end, students who basically do not have a work position are teachers with a certain foundation, so this is a problem, and then I found that the teachers of technology are too basic, and they are all research on these mechanisms.

43:20 If

we don’t know him, then we can’t learn it. We haven’t learned assisted reproductive technology, so how can I go to the reproductive center for employment in the future? I felt like I should be out of business. Then the basics are like this, and then I feel less interested later.

43:36

Can you say that you are not very interested in scientific research?

43:41

Can this be said?

43:42

In fact, I can say this because I think because I still want to find a job, not for this goal, but to find a better job, including the fact that it is impossible for me to study for a master’s degree and a doctorate. It's just to get a master's degree or a doctorate degree, or to publish a few articles that belong to your own journal, and then go out to find a good friend, these are all, so in fact, you are studying for a Ph.D., even if you want to do a Ph.D., in fact Also to find a good person.

44:12

Yes.

44:12

OK, what's the job definition? Are wages higher?

44:16

Still say that the social status is higher, or that all these things have to be considered, including salary, including social status, including this level of ease, I just feel that basic skills do skills, I originally thought that skills are very clear, This is the idea of my great founding. Later, I really went deep into the laboratory, and now I really go deep into the laboratory. Teacher Wang has helped me to train me as a master or a doctor. When I was training, I felt really tired, really very tired.

44:41 At

this time, I really quit the initiative. Of course, I pushed me back. I found that I found out that the activities are still quite easy, and they still have holidays. In their country, it seems that most provinces and cities still have holidays every Sunday, and they also have winter and summer vacations. We are in Wang Shi's laboratory, in my current laboratory, we only have 3 to 7 days a year, doctoral students only have three days off, from the first to the third day of the new year, and then the master's degree from the first to the seventh day of the new year, We don't rest on any other holidays. You know that I feel very hard during all the holidays. Of course, I feel that going out is just an example, this is an example.

After 45:16

, I learned that my classmates are not in the country. Are they in these laboratories or in other laboratories? In other games, they are pretty easy too. So you realize whether you realize this or not, it's still up to the teacher to see it, but I went all the way back, but I even asked my sister, why am I working so hard? So we say that we are not doing well, but in fact, I have compared and understood in the same direction. In fact, Mr. Wang is already quite good at this time. He can publish or publish every year.

45:40

Do not know good articles. In fact, compared with other laboratories, including immunology learning, it actually belongs to immunology now. I think it is already a relatively good teacher in language learning.

45:49 It

is clear that there are many teachers and bosses, and indeed they are like that. The classmates are very relaxed. I have classmates who are elsewhere.

45:55

I'm sorry to interrupt, you said that it will only be released for three days a year. In fact, you don't have it on weekends and we can't do it. So what should we do? Ask for leave.

46:05

With this, it's not so inhumane, you can ask for leave, you can't ask for too much. There is too much money, and the teacher must have an opinion on you. Anyway, the principle is not to be nervous unless there is something particularly difficult. Like are you sick? If you are not feeling well, you can rest for half a day, but it is not absolutely forbidden, it is possible.

46:24

But most of the time you try to be in the laboratory. When you have nothing to do, you are still working in the laboratory this year, so you are in the laboratory. In fact, you go to the laboratory, and now Mr. Wang's laboratory is also I'm doing more important work. Anyway, I'm basically training me as a master's degree now, and after Wang Shi also talked to me about this, he also stayed in this laboratory. Read on.

46:50

But it's too tiring, you will think it will make you a little bit at the beginning. In fact, this is also divided. In fact, a few years ago, because I was a sophomore, I decided to launch Guosong there. When I was in my senior year, I felt very tired in my junior year, and I didn't go to many classes even for experiments, so I went directly to the laboratory to start experiments, doing experiments all day long. .

47:12

Now and then at that time, it may be pure thinking, which is equivalent to proving my own ability like Mr. Wang or Minecraft, and comparing myself to the same undergraduates, or to some new masters, I have this The ability, I have the opportunity to do experiments, so that they pay more attention to me, and give me some good resources.

From 47:30

to my senior year, we felt that we might be a little too tired. I still have to learn to relax myself properly. Now there is still a process of adjustment, which is to adjust slowly.

47:42

So you're still in that lab, and you've probably always been in the lab, and you changed my roommate to my roommate and changed my roommate's view of this technology.

47:52

Because every morning when I was doing experiments at that time, for two or three months, I went to the lab at 8:00 in the morning, and they hadn't woken up yet, and then I went back at eleven or twelve in the evening, they were already asleep, he might For two or three months, I couldn't even see anyone. That's why I say them, but our real technology requires seniors to go to the lab. Won't. In fact, it's all like this. It's too tiring, and it's too tiring to do technology.

48:13

So now he has never considered the plan to pursue a postgraduate study in a technical college. He is going to go directly to the civil service exam, so he jumps out of this major. Your classmate who is a clinical transfer student is not basic medicine, so he is unwilling to study technology again. Now, he doesn't want to be engaged in related things in the future, so he can only rely on civil servants.

48:31

Because now they are also involved in the issue of postgraduate entrance exams, so you are actually very tired, but you still decided to stay with Mr. Wang. So can I say it is because it is not, because if you want to say so, in fact, the employment in China should be better. I think the employment of shareholders may be better. At that time, it was purely because of interest.

48:49

I'm not disinterested now either.

48:51

There is a certain proportion of interest and job hunting, and neither one takes the first place. You have spent a lot of time with Mr. Wang, and you find that these stem cells are not not called stem cells, but should be called immunity. I'm still quite interested in this aspect, yes, and it was also a hot spot at that time. Who doesn't care about tumors or cancer treatment?

49:12 A

lot of Nobel physiologists don't award pd1pd01 to these places, even I feel that although it is really important now, when we went there, we didn't care about the courses related to the rise in value every three years, the first sentence included the teacher's first sentence Tell us everything, now is the incidence of infertility, which has accounted for the third place in all diseases in the world. The first is cardiovascular disease, the second is tumor, and the third is infertility. Saying even is important.

49:38

This is because I may not be so interested in appreciation, or I am more interested in tumors, or I want to study these directions of cancer.

49:47

So in fact, it was just mentioned that you go to a senior brother to see the colonization, yes. After you get to know it, you may find that you may not be very interested in it, and what you did at that time was not very good, and I couldn't produce any results. You actually did it for a while. I didn't do it. He didn't do anything to his senior brother. After all, he is also a doctor. Yeah, I'm still an undergraduate student. What results, I don't think there are any results, your scientific research is equivalent to that. So in fact, he is a little bit discouraging you. Maybe I went to the reproductive immunization section, and I continued to stay in the Chinese People's Office to go to the reproductive immunization section. Maybe The results that come out may not be good, yes, and the system of the middle school class has been changing since the beginning. We will suffer from such problems in the first class. When we first entered, we told us The main thing is to recruit 30 people, and all of the 30 people's guaranteed research will be directly obtained for research. If you get it directly, you will be the guaranteed research quota, and there are no hard indicators.

After 50:44

, he said that it may be invalid to discuss with these later, and found that if there are not so many places, it will change later.

50:52 Between

them, I told the shareholders and classmates behind them that they also bought something. Later, it changed to English level 6, which is the most basic. English level 6, computer level 2 level 2, and then the grades are ranked. , You must guarantee that the grades are in the previous age.

51:05

At that time, 40% and 40% were right, and then the postgraduate guarantee was changed. Not everyone can pass the postgraduate examination, and they can no longer complain, including the ones that I actually asked for later, which actually requires me to do everything. If it can be achieved, I once wondered if I could still use the platform of the middle school class to get such a guarantee, because I don’t have any problems with these, and I meet all of them.

51:27

But because Mr. Wang, in my current form, he is also a common brand, so I will always choose him. Later I went to ask each other this question.

51:36

After graduating from the junior high school class, you must graduate with a subject of appreciation, and you must graduate with a subject related to appreciation. You can graduate in this direction as an undergraduate, and you can graduate with an undergraduate degree. Because what will involve them later is that graduation involves the defense, and the content of the defense must be clearly related. Did you mention this when recruiting? What's wrong with these two t's plus?

52:01

So you think that if you want to complete the project, it must be related to immunology, yes, because if I do the appreciation direction, I may not be able to achieve results. If I don't achieve results, how can I go about it? After graduation , you always have to do something. We were not that mature in the first place, which was also a consideration.

52:18

Then there is another factor to consider, that is, the latter shareholder said that he said he had given the postgraduate quota, and then even let us read 5+1+3 consecutive undergraduate, master and doctoral studies, and even re-assessed it again in the first grade. In the mid-term defense becomes, and then if you pass, go to PhD to 1+3. Then I thought later that our bodies are more immune, even if I think that if I graduate from an undergraduate degree and do a little something, maybe I can make it.

52:45

But I can't guarantee that I will be able to 5+1+3 smoothly later, if I can't 5+1+3, pure 55+3+3 is the normal kind of reading, then he is compared with others Competitiveness is not that great. Because my own ranking in the technical college was pretty good at that time. If I quit middle school in the second or third place, I could get the health care products from the technical college. I don't need excessive health care. I don’t think he has such a big psyche for me all of a sudden, so if you say 5+1+3, in fact, people have to do the direction of appreciation. If you want to take this road, yes, you have to take 5+1+ 3. You specifically asked this point.

53:23

I have communicated with them specifically for advice.

53:26

At that time, I was attracted to me, but later I found that I could not achieve such a condition, so you think that maybe if I did 5+1+3, I might have to do reproductive immunity, the result of reproductive immunity. It won't be so good, we can't graduate like 5+1+3.

53:40

Yes, now because I still find the foundation later, you just have to read it. For the doctoral degree, I still hope that the sooner the better, the shorter the reading, the better. Yes, if anyone can read two years less, who doesn't want to. , Yes, but it doesn't seem to be able to do physical discovery. Of course, there is a premise that it may be to change to another laboratory. Maybe Mr. Wang can't do anything, but in other words, they only do promotions, and they should definitely be able to do something. , because they are very mature in these technologies.

54:05

But at that time, it involved 4 rotations in the sophomore year, and I was thinking about it for the 4th time.

54:12 This

is the 4th rotation. I have spent more than 40 years. It does not mean that most of my technologies are still developed in the field of immunology. Of course, the experimental technology may be the same, but Most of what I know includes the formation of some of my scientific thinking, which may still be in the field of immunology, and I will go to the third year to go to the rotation again, and then go to other new teachers. I will start to familiarize myself with the environment again. At that time, I also took me to do new experiments, and then I had to go back and see what they did with the appreciation.

54:40 What

are their routines, including some students who even have to compete with other students, some students I come here specially, some technical students they are like this, they are also like me, they also have 4 sophomores. time, I chose 2~3 times, the same teacher. They have also discussed with the teacher, and they will definitely stay in their junior year and stay there in the future. So in fact, I think most of them are better. Some of the teachers have already gone to their students in advance. If I go again, I will not have any advantages, and I will not be able to compete very well with my peers.

55:09

If I continue to do appreciation, I have considered it.

55:13

So there is still a lot to consider, so in fact, because I am very cautious, I have been thinking about it for a long time, and I am very cautious. These plans cannot be made casually. Do you think about whether you have talked to your classmates, teachers or family members at this time? I have discussed everything, I have discussed everything, so we have discussed each of them. You tell them your thoughts, and they actually agree with your thoughts. Has anyone put forward different opinions?

55:37

doesn't seem to have either.

55:39

Some of my classmates have put forward such a different understanding, and they understand it this way.

55:44

You can also think that it may be risky to take the postgraduate quota of the technical institute, because the opening of the university involves the third, third, and fourth students who are still taking the exam and are still rebuilding their scores. The quota is 100%, and the shareholders also give out 10,000 yuan in scholarships every year, which is also a lot of money.

56:02

Then they even suggested to me that you are okay, you stay in the valley, you guarantee that you will graduate with an undergraduate degree, and then you take their postgraduate places to go to other schools to go to better schools, for example, to submit to Fudan. , which is unlimited.

56:17

I also specifically asked my counselor, there is no limit on the number of guaranteed graduate students, as long as teachers from other schools are willing to accept you, you can go. But I actually thought about it later, I think it's not good, because you used so many resources in the country, you not only didn't stay in the country in the end, but you didn't even stay in NTU. In fact, my personal moral is that I can't accept this, and I don't do anything related to taking other people's scholarships for nothing. I don't think it's good. So I didn't do this. I said that I would simply withdraw it, don't take these and leave these to other students.

56:51

Have you ever considered that if you go back to basic medicine, there are actually some deletions in your sophomore class. Yes, you have to make up for it again.

57:00

I have also considered this issue I have considered.

57:02

Fortunately, at this time, I thought about my eldest son when he was a sophomore, and everyone took a relatively basic course. There is no way for him to delete his activities in a patient with physiological, biochemical and pathological pathology. He only deleted some irrelevant courses, such as Neurobiology is basically only about two or three subjects, and I can easily make up for this. Then if I finish my junior year, because we are told that we can only push at the end of each year, maybe if I go to the end of my junior year and then give me three classes for my junior year, it will be too much, and I may have to make up seven or eight courses. Nine doors, it may be too late to make up.

57:32

So it's better to retire early than this, because I have already decided anyway, maybe I won't be able to get their one-year scholarship again in the future.

57:41

Well, in fact, you are actually going to take the guaranteed research quota of the technical college for immunization.

57:50

Now it is planned that you have obtained a postgraduate quota. In fact, you can also go to other schools. You don’t have to go to Mr. Wang for the experiment. Yes, but I have already agreed with Mr. Wang, and we have already discussed it at the time. You are actually quite certain now that you want to go to another school for graduate school. I am like this. I think once the negotiation is done, although you are just a verbal agreement, it is not good for you to go to another school like this. I don't think it is very good.

58:13

So there are indeed many classmates now, why should I go to a good school to develop, and what to go to a better school to develop, because after all, my skills are actually quite good. Compared with these statistics students, before. I did get in touch too early. They still have a lot of classmates. My classmates have never entered the laboratory and have never been exposed to these. , Yes, but they have to look at the teacher's system, you, you, we, they entered the laboratory, they don't care about their teachers, and even Teacher Yang gave them back to us, but my teacher never cares about them, and said that you can come whenever you want. , I don't want to, so they may have gone at the beginning, and he will not go after that. Until finally, Mr. Wang has such a classmate, who is our senior in basic medicine at the upper level. They are going to graduate and defend, and have already After the graduation defense, when it is time to write the graduation thesis, come back to the laboratory at this time, so I think that the main constraint is actually you.

59:11

You see that you are asking yourself to do better. If you want to go a little longer in the future, you should work hard. If you want to mix things up like this, it can be no problem, or it can be very easy. You can also spend leisure time every day.

59:22

So can you actually say that Mr. Wang's work on tumor immunity may be of interest to you, and in fact, it may be easier for you to find a job, so you are more certain that you want to work in his laboratory? Inside, of course, there are many factors in the entire specific flow laboratory, and this is the main factor.

59:44

Yes, it should be.

59:46

Although a little tired, it is actually acceptable, yes, because it is a little tired, but as long as you can do something, you are a little tired.

59:52 It's okay

to have some results.

59:55

Do you have any plans for your future career? Have you thought about this question? Behind?

01:00:00

I’ve been thinking about it since I was a freshman, because I think it’s really hard to find a job in technical research, it’s really hard. So I've been thinking about this.

01:00:09

In fact, the main direction of this road is to stay in school and become a teacher. The essence of our major is to recruit some university teachers, but now the university is too difficult to be a teacher, and the requirements for entering a university are too high, but in the future, of course, it depends on what you do in the future, if you do it yourself If you have done a good job in scientific research, you should go to a Ph.D. or Ph.D. degree. No matter how well you do a Ph.D. degree, if you don't have a Ph.D. degree or a Ph.D. degree, I don't think a master's degree or a bachelor's degree is competitive.

01:00:39

And if it's better in the future, or if it doesn't work well, because of the time with me, I told you that he has been with me since the second year of sophomore year. I admire him because he can really pay. He is very good. He makes me admire him both academically and as a human being. I have followed him all the time, and our relationship is also very good. I tell him everything, and we discuss it together.

01:01:01

He is also working as a post-doctoral fellow in Wang Anshi now, and we have been discussing how to go in the future. He told me which major is the requirement of being a post-doctoral fellow and how can I stay at the university to become a teacher in the future, which is of course One way, there are others, such as working in a company. If you do not do well, you will graduate with a doctorate and then go to a biological company. Some people say that we can go to the hospital, but I am not doing an internship in a hospital right now. My senior year is an internship.

01:01:25

I was an intern in a hospital. I found out that I chatted with these clinicians, but they don't actually do much technical research, and their enthusiasm for technical research is not that high. We still want to do surgery, just follow me. The jobs were the same back then, and they still didn’t quite agree with scientific research. Maybe the United States has a slightly better ethos in foreign countries, a little bit. When you talk with doctors, do you mean that you will go to the hospital for employment and then carry out experimental research with clinicians, right? I thought it was a pattern.

01:01:51

Because when I was a freshman and sophomore, what was our mentor called?

01:01:55

Dean Su Chuan, the mentor of book growth, he told us that, he said that the United States is a path, he said that there is a clinician like the United States, and then add a few more basic medicine, so that A team, a team, clinicians are looking for problems in the clinic, and then we are basically responsible for solving such problems in scientific research, and then extending it from small individuals to the entire group, large groups, I thought it was pretty good. .

01:02:18

Which hospital did you practice in and which hospital doctor did you make hospital clothes with? I think they don't do scientific research very much. He and I sometimes listen to them when they have a meeting. I listen to them and listen to their own chat. I feel that they don't pay much attention to scientific research. It may be purely for promotion. They have to be promoted. If there is a requirement or a request for a bonus, it will barely touch a little bit.

01:02:43

For them, the more important thing is to do surgery. The more surgery you do, the higher the bonus. For me, it may be limited, it may be attached to the hospital, and the doctors you contact may not have it. so many.

01:02:55

Actually, my question has nothing to do with the interview.

01:02:58

I understand that there are actually some hospitals that are more willing to recruit scientific research articles, because the evaluation system of the hospital now attaches great importance to scientific research, but the clinician does not say that we can all publish articles. There are so many high-scoring articles, for example, if you look at the rankings of Fudan hospitals, one of them is reputation, right? Another part is scientific research. In fact, scientific research is based on your articles. Therefore, there will be many hospitals that will specially recruit that kind of scientific research personnel. You come in not to do clinical work, but your task is to give me high scores. Articles Just looking for this.

01:03:32

Because many doctors and many hospitals are receiving evaluations, they will look at the nutritional factors of the articles published by your hospital, and in fact, tumors are actually quite easy to publish, and macromolecules articles are considered a good one. Bar. One to one pair, so I think it can be counted. You can pay attention a little bit, I actually think the same way, you know this thing, I know it, because I know for sure, I just thought it was you who went to the hospital, not the hospital, maybe there was no real good doctor at that time, mine Really good hospitals, they still pay great attention to scientific research.

01:04:10

In fact, a hospital is probably about two or three years after I came in, and I almost got up in 18 years.

01:04:17 At

that time, many of the doctors recruited were master's students. To me, it seemed that many departments of orthopedics, which may be popular, were right, but it may not be easy to get into now, because it is now in the Doctor, I had enough system at the time, so he didn't have such strong demand, so it is not as good as before, but it was still very good when it was first established.

01:04:37

Like my younger brothers and sisters at that time, they looked down on your hospital a little, and now they can't even enter, because the starting point of the first affiliated hospital is still quite high, after all, it is directly under the management of the school , so its platform is still good.

01:04:53

But it was not that good at the beginning, so the doctors inside may be mixed, and I also feel that the data of the first batch may be good, but the first batch may not be so good, but compared to the provincial These relatively large hospitals, including those in Gulou, actually attach great importance to scientific research, and indeed even recruit full-time scientific researchers. In order to publish high-scoring articles, if you look at the rankings, you will find that Shen Yi actually has no fertility. So high, but his research scores are very high, once surpassing many top 10 hospitals.

01:05:27

Well, so they actually value it. Yes, but I don't think our interview is relevant, but where did you learn this from? Information I said. Did you discuss it with your sister-in-law? I discussed it with my senior and also with those teachers.

01:05:41 The

tutor is right, because we have some tutors who will cooperate with the hospital. They say that the hospital is still a bit slow, and at that time our laboratory was generally a good hospital, yes, and our laboratory was still A clinician came to study and cooperated with other tutors. He introduced his master students. On our side, they are doing experiments and scientific research. I will also participate in their clinical master's degree, saying that they are actually doing scientific research. It's pretty good. It seems that I would also consider it if it is a person, but in essence, there are only three paths that can be taken in the future.

01:06:13

Yes, in fact, being a teacher at school may not be easy anyway, and going to Southern Medical University is not easy to eat during the post-doctoral period, so in fact, you will be willing to go to the post-doctoral period. You have communicated so much with your senior sister, and I have exchanged so much. Later, I found that you need to look at your ability. I think whether you are a master, doctorate, or postdoctoral student, you should look at your own ability. If my ability is good enough If I have to read it, I am willing to read it. I found that the postdoc is actually quite good.

01:06:47

But if your ability is really not enough, there is no way. If you just go in, you can barely get in, and then the ability in it means whether you can get out. The result he needs from you is the post-doctoral student. Can your requirements be met? right eh?

01:07:03

I think it includes a lot, because my sister is a postdoctoral fellow, and I think she may be different from others. She is the only postdoctoral fellow in our laboratory. , he will also bring new classmates, and then give them guidance, including undergraduates like me, including those students in the first and second studies, including many doctors, who also do experiments under his guidance, so that the whole laboratory can be together. He said that he told me that this is the only way to move the whole laboratory forward.

01:07:28

He said that he will always instill such a principle in me. He said that when you reach this position in the future, you should also be like me. That's it. So I think that if I don't have this ability in the future, I will be a postdoctoral fellow, I will not have the ability to solve some academic problems of my juniors and juniors, and I will have no way to guide them forward. In addition to your own problems, you have to help the whole situation and the whole team to improve together. I think this is a relatively good postdoc.

01:07:54

You can't just publish scientific research, it's just too good to publish articles, so can you say that you include from sophomore year to teacher Wang's laboratory to now, in fact, communication with your senior sister is better than with Teacher Wang wants more.

01:08:11

Well, a lot, I think this is also a lot of my scientific research. I have never been in contact with scientific research, but I have no idea at all. Up to now, you are still very influenced by him, very big, I feel that I Even the style of scientific research may be 70% to 80% similar to him.

01:08:25

, you may have been interested in immunity. Do you think it is related to it or it may be related to it, because I didn't know anything about it at that time, and he just explained the profound things in simple terms and followed us to me. Talking about some such questions, and he, he, he thought that at that time he thought I still had some abilities, and felt that my thinking could keep up with the speed of thinking, so he told me how to stand from his point of view What I was thinking about was how to do scientific research. This can be done. I thought it was quite good.

01:08:58

Maybe it’s because the first one is good means that this direction is pretty good, including doing scientific research in this way. I think my original intention is more meaningful and meaningful than clinical practice. I think.

01:09:13

It can promote the development of medicine. Of course, it is very big and empty, and it can promote the development of medicine, but in the end, we can't achieve the transformation. Maybe this is also a reason, and it may be quitting in the end. Activities, because maybe the first laboratory and the third laboratory I went to may not have the kind of senior brothers and sisters who took me hand in hand, including me going to the third people's teacher, and everyone in the laboratory did not give I arranged for a special senior to take me, and he let me go. Because their labs are very small, they are actually smaller than ordinary labs. At that time, Liu Liu, the teacher took the lab when he was an associate professor, so only In a small room, everyone was sitting in it, and everyone was doing experiments. He asked me to look at it and ask questions, but at that time, I don’t know why people who were more shy at that time were more shy, that is, he didn’t take the initiative to speak to you. If they were doing experiments there, I was quite shy and didn't dare to go up and talk to them.

01:10:02

Why is it so difficult for you all of a sudden, I don't know, maybe I talked to my senior sister a lot, maybe I talked too much with my senior sister, and I wasn't very grateful at that time. Because I'm a freshman, in fact, in the second year and freshman year, including high school, in fact, you are not too afraid to communicate with others a lot. Just changed it directly. In fact, everyone of me thinks that I should be the student council president. I almost got the position of the student council president at that time. time me. My counselor asked me to be the president of the student union, and I told him directly later that I refused and that I should leave the meeting, because at that time, I wanted to go to ordinary people who are more able to talk.

01:10:35 It's

true why you didn't know how you felt at that time, but you are usually shy, and you can't say something that you need to understand. But if you don't understand anything, you're too embarrassed to say it, and you're embarrassed to say it, I feel that sometimes my question may be more intelligent or naive, or it will cause dissatisfaction like tomatoes.

01:10:56 He

said that at that time, I did talk to a few of them in your channel lab and chatted with them, but not so much. Maybe it was because of time and time. At that time.

01:11:08 At

that time, it was true that there were not so many visits to Mr. Liu Xu. Mr. Liu Xu was more relaxed with the students, and he didn't care much. There may be more time to rest. So in fact, the two labs may not have any big mistakes in the discovery of interest, yes, maybe they just go for a stroll. It doesn't seem to have any real effect. If they really had another senior brother and senior sister to take you to do it, you may find that it is quite right to have spermatogenesis, but you went there at the time to see these things happen in the mirror, and actually did not have a deep understanding of it.

01:11:43 Right

. So this is a mistake. You think this is also a possibility and maybe it is right, because you, you and I think that after you have not really understood a field in depth, you will not be interested. You You can’t talk about these superficial things. I told you that when you were in your freshman year, you went to serve the country. In fact, it was mainly for employment, that is, 5+1+3. Yes, at that time, it was for 5+1+3 , It seems that there is no chance for promotion or scientific research, because at that time you have no contact with scientific research, you have no contact with grandchildren, you don't know the State Key Laboratory, you don't know anything Know.

01:12:17

At that time, I knew that the annual scholarship of 10,000 yuan was good, and the 5+1+3 system was good, and then I chose to at least stay in my own class. The basic medicine class in the basic college is better. Isn't there any good treatment for basic medicine? In fact, for this point, in fact, most of the classmates will feel it yourself. For example, if you say that it is accurate, it is your own feeling. Are the students around you mainly interested in the two days of postgraduate research and scholarship?

01:12:42

Yes, that's how I feel about it. I'm really interested in scientific research. In fact, there may be some of my classmates, but there are definitely not so many, because I think they don't even have as much contact with me. Most of the money they saved was probably for the first time they heard that they could guarantee the research, and if no one was excited, they should apply as soon as possible, and there were also scholarships, and the amount was still so high.

01:13:02

Including I did know that there are some classmates in the middle school. I said that there are some classmates in the middle school. They may really hold the idea that I told you at that time. They just went to the middle school to get a postgraduate name. First, and then go to another school to go to a better school after the undergraduate degree, it is not for the sake of being k The doc you turn in sounds awesome, and that's probably what they think.

01:13:31

Do you think there will be more students with this idea?

01:13:33

I think a lot, and I feel a lot around me, including me now. In fact, there are many classmates around me who are familiar with my situation. If I want to guarantee research, I will be guaranteed research if it involves me in September or October.

01:13:47

They said you should stop thinking about it, because I told a lot of people around me, I said where I want to stay in the future, I want to stay on the Internet, he said you should stop thinking about it, they all let me With the quality of scientific research like me, there should be no problem with submitting the block, and even the immunity of Peking University and Tsinghua University is still very strong, which means that the immunology of Tsinghua University is very strong.

01:14:06

Supplementary teacher, have you ever been moved? I actually have actions, but I will think about it carefully later, this is very complicated, and there are many problems. To sum up, as the ancients said, it is better to be the head of a chicken than the tail of a phoenix. I will go. Even if I can go to the laboratory of a very good teacher in Tsinghua University, I can guarantee that I will nurture my ability in it and be able to They were self-trained by Tsinghua undergraduates, and like me, they entered the laboratory in their freshman and sophomore years, and then continued to their senior year, where they stayed in the laboratory to study for a master's degree.

01:14:39

And I have studied publishing for 5 years. I studied for a master's degree in the insurance research area. I can't compete with their teachers, brothers and sisters. They all know them too well. They are the best in the laboratory. . So can I interpret it as you may be afraid, our premise is that you can go, you are more afraid of possible going, such as the immunization of Tsinghua University, maybe they don't pay much attention to you, because they don't understand you right, because I I think that scientific research depends on whether the old teacher pays attention to you or not. This is a big deal. If you do what you feel like doing yourself, it will be a big boost or main force for the group. If the boss doesn't pay attention to you, you It is an assistant. If the boss attaches great importance to you, it can promote you to be more successful or to be more straightforward and to publish better articles. This is a big factor in my opinion.

01:15:27

Later, I explained to many students around me that I said that you, including the PhD from Tsinghua University, are nothing special.

01:15:32

I think that for our scientific research, you should see how many articles you have posted and which school you graduated from. If you graduated from NTU with a Ph.D., I assume that you will post an article in Nature and you graduated from Tsinghua University, and you will post an article with five or six points. You can see at a glance who is stronger and who is weaker. I've said it, that is to say, the importance that the mentor you just told me about you may affect what you have told me and many people in Zhou Guo, because they are because they are not very recognized.

01:16:00 Right

, because they haven't done research yet. I still think they are pure, and they don't know what basic science is. I think they will include their current exams in the future. I see many classmates, and they are involved in postgraduate entrance exams now. Those who advise you to take the exam are basic medicine. Still active? Eat all? There are all schools outside the district, yes. In fact, it stands to reason that those students in middle schools should have a relatively good understanding of scientific research, and some of them may not have that deep knowledge at that time.

01:16:29

Yes, it may be because I talked too much with my senior sister. At that time, I became familiar with my senior sister. I have always been familiar with my senior sister, and they may not have such an opportunity. Another is that they may not be themselves, because I have some interests myself. In fact, it does not mean that I also want to make some of my own things. I think this is more impressive. Of course, this is what my senior sister told me, So I also think according to him, I think this is a more admirable thing.

01:16:50

You still want to be able to make something of your own, something innovative.

01:16:54

There is still a certain amount of contact in the field of scientific research, which I think is better. But maybe they have different styles in each lab. I don't know what kind of scientific research training these students have undergone in other labs. I don't know what their cognition is. , When I chat with them, they sometimes say that they may have to hand in the burden.

01:17:15 What did

you just want to say about the basic medical exams? They pay more attention to the test results. For them, they are simply going to postgraduate entrance examination for the sake of graduate school, and they are studying for postgraduate study for the sake of graduate school. Yes, I said that you have never considered which direction you like. You have not yet set a direction. Have you done any of these?

01:17:31 I've

done cancer treatment, and it's just an appreciation. You should take the test in this area, and you don't want to know how the laboratory is. Will the boss be strict with his classmates? We never think about it. When the time comes, we will first think about handing in the exam first, and then we will consider these after the first trial and the re-examination.

01:17:44

I said it's too late for you to think about it, it's too late. Sometimes I feel for them because I have been in contact with them as a freshman, and I feel that I have to know a lot of things before I can be qualified to go to graduate school. Alas, of course, it is possible to know too much and it is not good. For example, we have a lot of pressure on the new recruiting part of each laboratory. Oh, if you want to do better, you will be under a lot of pressure, and you will be very hard. If you look at the new research, they actually don't have any technology. They must not have understood these things back then, so they just signed up.

01:18:13

Reported in, and many of those who were admitted in the test were still transferred in, and it was all like this. And so in fact, this kind of pressure is actually given to yourself. If you really come in ignorantly, it's actually fine, and you can even go out ignorantly.

01:18:29

I thought it was like this later, that is, each of you has different requirements for yourself, what kind of requirements do you have for yourself, what kind of yourself do you have? For a diploma, he doesn’t ask to publish articles or do some topics. He may be better than that he can graduate with his own articles. As long as he successfully graduates and has a degree, I think he should probably live in the laboratory. It is also very relaxed, so you feel more tired and more stressed now, which actually comes from your hope that you can make a pair of innovative things, or in other words, I am aiming at this world, and I hope to achieve This level, because he also wants me to be able to reach his full body and mind, I think he is leading me to do it wholeheartedly.

01:19:06

Many of his masters and doctors did not give me answers to many questions as wholeheartedly as me. So I took him as my goal, I wanted to be able to sit at his house, so I was very tired, so I would go in and out with him when he arrived at the lab, when he left, and when I left . He will bring you more moving, or you will feel more proud.

01:19:28

I have all these feelings, but it is very touching because I think no one will go. Of course, he also told me about this. He took me all the way. When he was his successor, he felt that my ability was not bad. In the future, because he had finished his postdoc, he would also graduate, and he would not have told me that he would not stay in Wang Shi's laboratory forever.

01:19:51

Then he said that I don't want to stay in Wang Shi's laboratory. Maybe I hope I can take on his current position in the future. If there are any problems that the students in the future don't understand, I hope I can imagine that he teaches now. I'm going to teach them to pass on these things. According to your current grades, you can be admitted to the postgraduate research. There is no problem.

01:20:07

No problem, right. You also don't need to re-repair and brush points, etc., to say that you are useless and useful, we ourselves have 179 after level 18, and it is useful to not start from level 18 without us. So I really want to do that, because I won't tell you for the whole year of my junior year. In order to do the experiment, I actually didn't take many classes, and my grades that year were not very satisfactory.

01:20:27

Then including the classmates who are now sent by us. Sometimes they go to the research institute and they just keep swiping like crazy. Oh, they don't go to the laboratory, because they never do scientific research, they just In order to make your grades better, just compete like this. In fact, there is still a certain pressure to compete with them. But for now, your grades should be fine. It’s just that it’s only a semester away, so it’s fine for now.

01:20:48

It's still okay at the moment, but the 3rd place in that year has now been transferred to the 6th place or the 7th place. But you are still in the front class. You are in the top 20. There are 10 places for 10 people to apply for 10 people, and we have 10 places for guaranteed research. Then of course, I will guarantee 10 of your 30 people this semester. I don’t know if I am not 20%. For 50 people, our 20% is the same as preventive medicine. So for now, you can still guarantee the research. You still want to go to the current laboratory because this senior sister is more concerned about you. Of course, in addition to observation, you should also consider some realistic things, experiments. In the room method, Wang Shi's strength is still quite strong, and he can post some high-scoring articles.

01:21:29

Do we go to the lab just to see if we can post some high-scoring articles? Now that your laboratory can meet my requirements, I feel very good. Just stay here, Mr. Wang also treats me, Mr. Wang also attaches great importance to me, and likes me a lot.

01:21:45

So in fact, it is more because I want to go to the direction of immunization to check, and then I quit. Yes, I quit, I know, yes, when I quit back then, I did tell Hu Xiao that I was not interested in reproduction. Later, I did it for more than a year, and it was already a long time since I quit, and I thought about it later. In fact, I didn't have a deep understanding of what interest in talking about. This may be the same, but it may have been because I was more interested in immunity at the time.

01:22:09

Did you still want to chat with the principal when you quit? also applied.

01:22:13

I sent him an e-mail to apply, and then asked me to send an e-mail to Hu Xiaoyan in person, and then I went to Mr. Huang Xiaoyan to tell her, and he immediately forwarded what I said to the stock, So you all have to chat with Hu Xiao because of one who quit, because the first few sessions always require students to take the test, because this is invalid, he needs to know why we quit, the next section may be the next few pages. There will be a reaction. He told me later, Mr. Wang, and he specifically called Mr. Wang again, because later he said that I would stay in the online laboratory, I would have to be immunized, but I would not even do it anyway. I called Wang Si. What was the purpose of the call?

01:22:52 It

means that you and I are good at cultivating everyone and don't poach people from my house again, or I don't dare to be specific. If I am a big boss, I don't dare to ask. Do you think he was happy when he said this to you or what kind of emotion was not very happy for you to talk about it, but I should have a good reason to go.

01:23:17

At that time, he seemed to be saying next year's words to give me more reasons, so that they might blame each other, and then he must think that he thinks Hu Xiao is blaming him, he may feel that way, because he does too. A part-time platform for appreciation. In theory, he should also do part of the promotion, but if we don't do well, we must admit that my king is an active process. You have also told Hu Xiao, and even immunity I have never talked about However, I just said at that time that I was not very interested in my body, and I was interested in beauty, and then I also said that at that time my family was not from Sichuan, and I said that it may also be a transportation, then At that time, in fact, Wang Shi didn't talk to me about Liu Bei at that time. At that time, I still thought about it, and then go home in Chengdu or go back to Sichuan University or find another college, because I still feel too far from home here. Now, how inconvenient it is.

01:24:09

At that time, I didn't think about staying here. I told Hu Yao, maybe I can't stay in the middle school to study 5+1+3, maybe I have to go home in my hometown during the master's and doctoral stage. Read over there. No longer at NTU. If I was destined to leave after graduation, I would not have to use the postgraduate quota to save the postgraduate quota for the more needy classmates. I shouldn't want to do some things later. I didn't expect that at the beginning, I would still stay there. It's too late. I don't know how to deal with it. When I go back, I may have forgotten. He has to be the principal, not the principal. said.

01:24:40

No, because I need to be concerned about my kind of door. I am a graduate student and I should not be able to get in touch with him.

01:24:48

This student seems to have talked to him. The other two of you quit at the same time. Have you talked to the other two students? I can promise they talked. Did you talk to them? I talked to one of them.

01:25:01

I was the first with me, because I was the second. I talked to a girl about Dai Yue, and he told me that the first one we launched was not my website, but our professional The first place, that is also a legend. Haven't you talked to him? You are not the girl who just came in to chat with him, but I haven't sorted out his chat information. The main idea he expressed to me seems to be the first. He wants to leave the research to other students, right because he is too strong, he will always be the first.

01:25:29

Oh, he changed majors, he was also the first to change majors, he was the first, he was always the first, he only changed majors, he did not change majors, but he was always the first, he Instead of changing majors, he is more interested in technology. I feel that he was really hotter than me back then. I think I am fairly interested in technology and love it. Of course, it may be that I did not transfer to clinical. Team, it can really be transferred to clinical, or he gave up on technology and transferred to major, and then went to the middle school shareholder examination for interview and still ranked first.

01:25:55

So I found out that he's actually similar to you, not even interested in doing it. right? I don't know how you feel when you chat with him, even when he told me that maybe he is really powerful, at that time we all felt that the shareholders said that everyone should be guaranteed research, not everyone has 10,000 Blocks are fine. He said it was impossible. It’s just that I feel very low. He said how could everyone be different? This is definitely a problem. In the future, because we may give us such a post-declaration system in the first session, the system will change.

01:26:23

Don't believe this for now. If you think about it, close it, and he will leave after a month or two. I persisted for a year, and he did not finish the rotation, so he rotated one or two labs. But he had already joined at that time. I talked to him, and I talked to him before I quit. How do I choose to quit?

01:26:37

He said he was in Mr. Guo Xujiang's laboratory at that time, Mr. Guo Jiang, Mr. Gu, who I thought might be a relatively good teacher, was to prepare lessons with us, and then prepare lessons with us. , and then we got to know him. He was in the laboratory where he reported the loss. He thought that he might, I think, really understand Shenzhen in depth, and then he was not so interested.

01:27:00

Because I didn't say technical colleges just now, I was actually very concerned about the movement of our group of middle school students, and occasionally asked us to communicate, so what difficulties do we have? Most of us recruited 8 students. , there are 8 or 9 classmates, seven or eight of us all say that we really don't have time to do it, we are just too boring, just like the study room, we just go to this thing, he says I really have too much time Too much of me makes me tired, I work so hard, he has already gone deep in advance, because he is more powerful than us, I think he is quite powerful, he may find that he is not so interested when he really goes to understand, He will find it even more limited, because it is still after me. It seems that there is a teacher who has forgotten who it is. When they chatted with me, he said that he said that later I found that he said that he said that the students in basic medicine may be better than middle school students. Students in the innovation class should have more advantages.

01:27:54

He said that Shen is a classmate who has passed or even passed the middle school, because his later course settings are also appreciating, he deleted most of the courses, and then went out together at that time, the course was very slow, and all the courses were superior.

01:28:06

But they have an advantage, that is, they have a wide range. Even if they only learn a very front-line part of each subject, they will have a wide range of choices in the future.

01:28:14

If they go to graduate school in the future, they can choose to go to study, even they can choose to study the only child, they can choose to study pathology, physiology, and immunization, they know a little bit about everything. But if he said at the time that I was still in middle school like us, and he said that if he was like your shareholder, what about most of your students, if you want to go to graduate school from that time, you may not feel very interested , thinking, this is also one of the reasons I launched.

01:28:35

If you want to change, it will be very difficult, because all the training you have received, that is, all the education is all related, and then you go to graduate school and then want to do immunization, why should others accept it? Why don't you accept a classmate who knows a little bit about immunization, so he said some of these things to you, and even to a teacher, I don't think his speech is reasonable. This may be because I am one of the high school students. It's true, I was still in the country at that time.

01:29:00

Yes, the teacher told me during the interview at that time, so actually going there may even limit your future possibilities in the future, and limit some possibilities, but this road is easier to walk. What do you think personally.

01:29:14

To me, there is another classmate who has been staunchly staying in high school. He is my roommate next door. He has a very good relationship. He has been staying in Mr. Liu Mingxi's laboratory. body, why? Because he thinks that I don't think he thinks this road is easy to go, because he said that his family is from Jiangsu here, and he thinks it's good to stay in Nanjing Medical University, which may be well-known in Jiangsu. Not bad.

01:29:35

He thinks that instead of going to Fudan, it is equivalent to gambling to choose a mentor. He might as well choose some mentors for you and you. The mentor thinks that choosing a mentor is like gambling. You taught him the concept ? Or did he just have this idea himself? Ah, we both have the same concept, and I said that I also think so.

01:29:53

I look online to see what is wrong. It's really a bit of a gamble because you don't know much about being an export mentor. So for him, he may say that he is not very interested in promotion. He just said that the 5+1+3 model is very attractive. They think that this kind of reading is good for An Anxin after two years of work. At the end, he said either in the future or he said that, and the middle school told me that back then, even if we wanted to recruit teachers, we would give priority to the students in the graduating class of our middle school, but he said that he wanted to find a way to go directly to the school. Nanjing, he is a teacher, so he doesn't have to think about anything, but this is also a good way to go. If there is no accident, he may be like this. If you are employed, you may not necessarily be in Nanjing Medical University.

01:30:30

Maybe you still want to go back to Sichuan now? also closes the possibility. It’s not convenient, but I didn’t think so. Yes, I actually thought so, because when I was a freshman and a sophomore, I really felt too far away.

01:30:41

And I did fill in the wrong majors back then. In fact, I can really go to Sichuan. I didn't take the first one when I was good at Sichuan University. At that time, I thought it was too hard, and it was too hard to study medicine. , it's too hard to go to class every day and then go to the lab, why do you feel that it doesn't matter now? Maybe some of you are interested now, and some of you can't do anything about it. Since you can't change it, you can only do it and understand it. Some of them, including the basic medicine of Sichuan University, are not that good. I have talked to them many times, and I have actually talked to them. I asked them, and I said, what do you think of Sichuan University?

01:31:14

He said that it is good for Huaxi to be good to Huaxi, but it is only for their clinical and oral practice. In fact, their basics are very general, and the treatment is not so good.

01:31:23

In fact, it belongs to the treatment you mentioned as a teacher. If I want to return to Sichuan for employment in the future, the treatment of teachers is not the same, in the school. It's not the same as you say it's a platform, these things he got the subject are not treatment. The teachers in my Sichuan University are different, and the treatment is different. Isn't it because you have different strengths in introducing talents. For example, Wang Shi told me about his junior and his junior, and his junior and senior posted a sale, which was a shocking one, and then I thought that I wanted to go back to China when I was abroad, and I was abroad. The postdoctoral fellow wanted to go back to China to become a teacher, and the first choice was the Technical College of Sichuan University, and then he would give him 200,000 or 300,000 a year when he said it back, plus a little hundreds of thousands of start-up capital, he said. It is not as powerful as the Southern Medical University, which is also a factor I consider.

01:32:09

It's not so strong now, and I don't care so much about how it will develop in the future. Besides, it's okay to stay or not to go back to my hometown. Think far, think too far, think too much every day.

01:32:21

Most of my classmates may only want to eat tomorrow. I have seen how to find a job in 10 years. Has your personality been there since high school? I don't seem to have any, I have never been in high school, I learned casually in high school, and learned in person. When I started college, I didn’t study much in high school, because I didn’t study very much in high school, and in high school I just played. have fun. That is innate intelligence.

01:32:44 It

seems that all these people commented on my teachers like this. He felt that I still had some impact and I didn't work very hard. Then I was in high school, so the final college entrance examination was about to take the college entrance examination. I still have a few months to review. The single test is not very good. I don’t usually review. After taking the level of Sichuan University, I chose it at random, and I chose a southern medical doctor. After I came in, I worked very hard.

01:33:08

Because maybe everyone has used it in the big environment, and I don't know why. I wanted to change majors in basic medicine. Everyone worked very hard in the freshman year. At that time, the exam was the next day during the exam month. Basically, everyone didn't go to bed before 3 o'clock. Many girls stayed all night. Yes, I was also influenced by this. I worked very hard later, but in the second half of the general description, I was concerned about some things that I thought might be too expansive at that time. I thought physics and chemistry were too simple, so I just looked at it and went. Exams, so I didn't do well in the exams. This is also a point of my not growing major. My main course is very good, but I just listen to some of my physics, chemistry and mathematics. did not do well.

01:33:44

However, when he changed his major, he required all subjects to be integrated together, so my overall ranking was relatively low. If I only count the grade point average of the main course, I am still in the top 20%, which is more than enough. I regretted it, but when I changed my major, I found out that it was only one or two short of that. I definitely regret it, but I think I took a good look at physical chemistry and took a little more scores in those courses, which may be different now. But there are still relatively few people in the back, but since they can't change, they continue to move forward, so I continue to not be in contact with such a situation.

01:34:22

Generally speaking, do you still feel satisfied? Go all the way now. I think it's not bad, although there are some minor things, such as not turning into a major, for example, if you think about it now, you will still feel a little regretful, and you will regret it. In fact, other things will come from my guess. It comes from parents.

01:34:44

Yes, the main reason is that my parents have not fulfilled this requirement for me. They may still prefer a clinician, which may be me or myself.

01:34:54 It's

not really good, it's all good, it doesn't really affect me much, but I feel that it's pretty good overall, because I'm lucky to have met many good teachers, I think this Teachers, the teachers I went to rotate were all very good. The atmosphere of the labs I went to was very good, and I also encountered a very responsible world that has always taken me with me, and I also had the opportunity to enter the middle school. I feel that it is already very good.

01:35:20

The platform of the middle school is indeed I think the platform of the middle school is indeed a little higher than the platform of the technical college. Where is Gao? The labs are changed, and the labs are more exposed to more technologies and colleges. They can even be exposed to more things. In addition to giving birth to them, you can also have a cheap living, is there? Where do you think he is tall? They have all kinds of opportunities, for example, they can take us to visit the reproductive center, and then there will be some academicians who will give a report last time, so that we can listen to the many opportunities he gave to the middle school, and even help us Sign up for the animal certification test.

01:35:51

They all think it's pretty good, yes, so basically they don't have engineering in basic medicine, no matter what your instructor asks, then let your instructor sign up for you, and you can take the test yourself. Fundamentals are not required.

01:36:02

So I think that the class still attaches great importance to the students, I think they attach great importance to the students, and the scientific research training has been done very well. Have you ever had any of your schoolmates come to ask you? There is an unmanned class. They ask a lot about what subjects they take, and then they have any questions during the interview. They are already in the summer, and the reason is that they only ask when they have to take the exam.

01:36:27

The questions of the interview are about what to test and what to test, but not much else. Because I feel that most of them are not even basic classmates to ask me, it may still be a clinical test. It seems that there are a few nurses, and a few who have no clinical medicine, no clinical, and no clinical testing. There is a tester, and there seems to be a nursing one. They all come to ask you what the test is and what the questions are. I think they may have never been in contact with scientific research during their freshman year. They don’t even know what the laboratory is like. They just want to come in and take the exam, and after they come in, they go to contact with scientific research, and it seems that they will do it later.

01:37:03

If you said that you wanted to quit, you chatted with another quitter. Do you think chatting with him changed your thinking? Or to say that it remains the same, I have actually made such preparations when I go to the later stage.

01:37:18

Well, it means quitting. In fact, we had two classmates at that time, because every time we rotated, basically two classmates were rotated to a laboratory together. My classmate was a junior high school student and also a junior high school student. He had the same idea as me, I also want to quit the country, and I also feel that I am not interested in Shenzhen. Maybe I don't know the specifics, but I also want to stay with Teacher Wang, and I also want to stay. Maybe Hu Xiao later scolded Wang Siyuan this time, maybe it's the person who was poaching him here. He didn't quit in the end, he quit. ? did not return.

01:37:50 In

the end, he did not withdraw, because he may have considered that, because first of all, his grades may not necessarily get a guaranteed place in the technical college, and he is more likely to smoke in the country. right. So based on that, he might not be right in the end. Then he seemed to be the one who asked me to help me, can I not do the direction of reproductive immunity? After graduating from a bachelor's degree, he may or may not be able to, and then he is very distressed, he was very distressed at that time. In fact, he may not really want to do production, yes, maybe, but he still thinks that he may still be responsible for research, because he thinks it may be too difficult, and it may be too difficult to go to the basic hospital to fight for another health care product. tired.

01:38:33

He then thought about it, and then he chose you to see our lab go elsewhere, and even I finally opted out of the process and stayed in this lab, the results were better, and it could be more willful One point, follow your own interests but you can't call it that, but in fact, I really thought about it a lot, and I think it's quite a lot. I said willfulness in quotation marks, I know what you mean.

01:38:56

He may have to first ensure his basic needs, that is, to retain God. In fact, you can achieve your grades and your basic needs, and I can pursue more things, because in this way Promise yourself to do better. It may be that you are interested in doing more in this area because you have such an interest. I wouldn't be in the lab 12 or 3 hours a day if I wasn't interested. Do you still feel this tired now? Even after being in the lab for so long, there are still more than ten hours in the laboratory, and now he feels okay, and he feels used to it, so he may have been used to it in the past.

01:39:31

Of course, if when your experiment is very successful, you feel that time flies quickly, and you bring a little thing to the early morning for two days, and you feel that there is no problem with you, now you have actually been working independently. Doing a certain topic or the like, you have already started right, that is, you started doing it very early. This is equivalent to the world's distribution to you. Maybe you can surf the Internet through the Internet, and the top is through the Internet. This is when you were in the countryside, and there was actually no later. I am doing Daiso, and I am doing Teacher Wang. Yes, I also specifically asked Teacher Wang. I said if you want to participate in Daiso, I can help you go to the whole team.

01:39:58

My mother said that there is no need for it and it is unnecessary. After thinking about it, it is quite hard to be responsible for a subject independently, even so independent that even this group requires me to go to the animal room by myself, to arrange the room by myself, Go and divide the dragon with the dragon. It was quite hard work. Later, I felt that I didn't have the energy to participate. Others said that if you want to participate, you can participate in the big window of other laboratories, and you can participate as a team member, right? I feel like I can't keep up. This is not very good.

01:40:24

I remember when I was chatting with other classmates, they mentioned that after entering the junior high school, they actually asked the students to withdraw from various student groups. Have you heard of this policy?

01:40:39

Seems to be true, indeed they were still in the student union during their sophomore year, right there, I think, but I don't think it is necessary for them to withdraw like this. They did tell us that, but we feel It was not necessary, because they thought that at that time, if our energy was focused on student work, we could seldom focus on scientific research and technology, but at that time I felt that my experience was quite good, and at that time I had already done arrive.

01:41:00

In fact, I joined the presidium at the time of the student union. I don't think I have so many things. It doesn't matter if you pick him up in the second year. How can my classmate pick him up? So when you were a sophomore, I mainly let the ministers below take charge of some things, so at that time I tended to leave the hands of the boss. In fact, I occasionally did some things, but I don't think it affected my main energy. I didn't quit, I pushed one, I pushed the monitor, the monitor I run for the freshman year is the monitor in the student union, yes.

01:41:25

So in fact, you don't think it has any effect on your experience, yes, so if I didn't quit some teachers, I wouldn't check you. How could the teachers check you? I don’t think so. He just said that he made such a request. Yes, I think that the main thing to ask is that everyone should focus on scientific research except for today. Maybe he wants us to receive these scientific research trainings. Orthodox scientific research training is still It takes time to spend time on research.

01:41:47

Well, then I can introduce a lot. I introduced and we became the squad leader. I was promoted from the track and field team. It was the school track and field team at night, and then I also retired from the basketball team. It should be called the club basketball club. , Tianjin Society. The dean of the School of Basic Studies was right. So in fact, after you entered the junior high school class, the impact of your life on my spare time has actually disappeared. Including when you see that we also ask us to go every day, and then I actually have real classes, except during the necessary student union meetings, except for necessary meetings, except for classes, I basically spend all my time there. laboratory.

01:42:27

I said that I have spent more time than my classmates in many middle schools. Some of them have their own entertainment time, and I even cancel the entertainment time.

01:42:37

I felt very negative emotions, but I didn't have time for entertainment, but I didn't, because at that time I thought scientific research was quite full and interesting, except for the first laboratory at that time, It's not that when I go to self-study, there is a little bit of negativity. I feel that I can't study by myself when I go to self-study. I have to sit in the laboratory for self-study. Well, the environment of the laboratory is not so good. I am not a self-study in the self-study classroom. Be clear, a little bit of negativity, others include not actually spending as much time in the lab as now.

01:43:06

For now, I think these are still interests that support your interest, or I really feel that I have no interest in doing scientific research. It is really painful. If there is no interest in scientific research, it is really painful. In fact, the most important thing is to be interested in scientific research. The other time is actually quite empty. The most important thing is to support you. Yes, because it does take a lot of energy and time. If you do not have Interest, you spend so much time and energy will bring you a lot of negative accumulation, and these negative emotions will further affect the efficiency or various aspects of your experiment.

01:43:45

You can see that I am a comparison person. It doesn’t matter now. Sometimes some students may be a little introverted. I am asking because I have also been an interviewer. I will interview pi. Oh, in fact, you I don’t need to ask many questions, you just tell them all by yourself. I feel like you want to ask me, so I’ll tell you first, and I don’t need to ask you to tell me first.

01:44:09

Actually, I don't think it's anything. I think I think I have already understood the whole growth path. My three paths are still very simple. I don't think it's complicated, but I think a little more. I'll tell you what I've been thinking about. Yes, I actually need these things. O then come over here, okay, you wait for me a moment, what shall I get you? three days. There are also meal coupons that cost you two hours. 55 dollars. thanks. When is it now? Okay, thank you teacher.
