# Supplementary material for: Why do you choose this program?—A decision-making model of medical students based on grounded theory
Source: PLoS One. 2023 Sep 15;18(9):e0291634. doi: 10.1371/journal.pone.0291634 (PMC10503722; doi:10.1371/journal.pone.0291634)
Supplement: S1 File — (ZIP) [file pone.0291634.s001.zip › RAW DATA/P12 CHINESE.docx]

00:00

这个这个过程和理性的一个选择的情况。但是然后在这个访谈的之前，我先要读一下道德伦理须知，本次访谈中受访者是在平等自愿的原则上参与的，受访者必须真实表达自我想法和认知，确认自己符合社保条件。访谈过程或者录音，其录音资料将以匿名形式用于科研，不会泄露给任何第三方，在访谈过程中和结束以后，你都有权取消研究人员录音资料使用权，你是否知晓并同意嗯？

00:34

我知道了一下。

00:37

你先说一下你的年级和专业，我是大一现在，然后我的专业是护理，你的成绩大概是在什么？位置啊？年级排名是110米。你们总共多少人？总共有我们这个专业有250多个人，250多个人。好。你们现在有有在宣传国中办这个事情吗？之前班委有发过那些相关的文件，关于活动班的招收什么的那些，你有看吗我有看这个文件发了一个文件，其他没有。

01:19

有宣讲会之类的，东西吗还没有是吧？这个文件也是上个月才发的。是什么时候发的？好像有一段时间了，是上个月好像发的是19级的转国中班的一些事情嗯。你看完你看完以后有跟同学讨论吗？有。因为我还蛮感兴趣的，对。你是个人比较感兴趣，还是你觉得周围同学都很感兴趣？我个人比较感兴趣。

01:52

可以说一下为什么吗，因为首先我是因为调剂进的护理，当时所以我其实对这个专业没有那么的感兴趣。然后我以后从事的职业的话，我感觉我对科研的话会更加有兴趣一点，所以我本身就比较想转专业。然后我看到国中班它是可以转专业的，所以我就还蛮感兴趣的。

02:18

你当时高中的时候报名是报的什么学科？我报的是医学检验。然后我第一志愿是医学检验，然后后来调剂调剂到了护理。你们这边应该是可以报6个的，对。你就报了一个。我检验检验，然后后面报的是报后面报的我也不记得了，然后护理是排在第5个的还是第几个，然后反正就不是反正这不是你一开始想进的能力。对。除了检验你有没有其他比较想进的专业现在我们，现在目前是特别想进基础，基础对，基础和检验目前是自己比较想去的两个专业对。

03:11

我可不可以问一下你在高中的时候，报名啊报考大学的这个时候，你是理科生还是文科生是理科生，你当时报的时候是报了医科还是说我就只报了一科，我第一专业是南京医科大，第二专业是南京中医药。

03:32

可以说一下原因吗？因为我当时就说一下这个过程，也不叫原因，对就是大概是怎么抉择的一个过程。首先是我的家长，我爸爸妈妈都比较希望我从事医学相关的工作，当时他们是觉得比较稳定工作。然后然后觉得比较实在，可能就是护理，还有一个师范，但是我自己的话我不是很喜欢当老师，然后我就比较对医学比较感兴趣，而且当时我高中的选科是无声，所以我就报考了医学类的专业。

04:11

所以可不可以说是家长先帮你确定了一个范围，医科或者是师范类。

04:16

对，然后你选了一个一科，大概过程是这个样子是吗？对我自己本身也对，我跟我的爸爸妈妈也一直就在我选高二的时候选课的时候，我就已经表达我比较喜欢医学类的，所以我才选的50所以其实当时也没有考虑工科或者理科这些专业的东西没有考虑，然后你就报进来到了医科这一块，进了检验，奖金检验，最后进了护理对吧？

04:53

对。你目前的成绩应该是符合进国中的成绩的条件，对吧？对。嗯你跟你周围同学讨论的时候，没事，你坐这儿还是坐那儿？我就在这，你在这等吗？你在这里没有椅子，对他这没有我的意思了。我可不可以问一下嗯，您有没有考虑过转专业这个事情？我有考虑转专业的事情。你们有收到这个通知吗目前？有。然后你有考虑过要转哪个专业吗我要考虑我想转基础也还是基础，对基础。

05:43

基础和预防，因为预防它转可以转进的人数比较多，所以我感觉预防转的话可能就是能进的概率大一点。你一开始高中的时候报的是检验，为什么现在突然又要报基础和或者预防这两个专业因为，我这两个专业我在上高中的时候，我其实并没有就是说了解过，是我上了这个大学之后，我才了解这两个专业的，然后我就身边也有一些预防，还有基础的同学，然后我就感觉我还对还是比较感兴趣的。

06:17

可以说你是从哪些途径了解到的专业吗？就提出把这些东西首先是我跟预防班的同学是一起上英语课的，然后有的时候就会聊一些关于他们专业是干什么的这种事情。然后还有的是从我的一些其他的朋友那里，就是同学那边知道的他们到底是做什么同学，是高中同学还是没有是大学同学？

06:45

你怎么认识这些大学？一些部门里的同学，有的是部门里的，有的是社团里的那些同学，你参加了哪些社团啊，或者说就可以申请外院的人？天文协会，然后就会认识很多外援的，然后有的时候做志愿者活动，我也会遇到一些外援，团委的，志愿者评优那些东西，对。他是护理的是吗？你是护理管理的。现在大一对吗？对你在考虑转专业是吗对，考虑转基础或者预防对。

07:22

你刚刚说上英语课可以和别的同学一起讨论他们专业的东西，比如说讨论他们的专业是干什么的，你可以说一下你对基础预防的理解，他们以后可以干什么，预防的话主要是在疾控中心工作，然后也可以进科研室，就是去搞科研进实验室，然后基础的话好像就是进实验室。

07:45

然后我听一个学姐说说基础的话，他如果是本科毕业的话，他没有那个工作等于本科毕业等于失业，他有这样的一个说法。然后主要是可以跟着一些做实验发文章，然后以后工作的话就没有劲，进医院那么累，好像可以自己把控时间，经营基础或者预防。

08:14

你刚刚感觉好像漏问了一个东西，你在高中时候报检验的时候有考虑过是哪些因素吸引到你了吗？还是说只是随便报了或者说专业和你的分数比较匹配才报的？首先是专业跟我的分数比较匹配，然后我想进医院工作，我觉得好像工作的话比较就是说比较稳定。

08:37

然后也好找工作，我感觉还是比较好找工作的，像护理这些专业其实也挺稳定的，进医院当护士或者进医院的检验科其实都是一个性质，但是我感觉护理的话就比较累，他很辛苦，然后有的时候我感觉我上一些护理的实验课的时候，我就感觉我对这个专业并没有那么的感兴趣，就是说以后如果让我就一直从事护士这一行的话，我感觉会有一点不太舒服，而且他要三班倒，然后我就这点我也有点接受不了，护士比较辛苦，然后不感兴趣，我感兴趣可以。

09:19

聊出来具体的东西吗？还是说整体上的一个感觉？他一些工作我觉得很繁琐，太繁琐，然后又没有什么创造性，我感觉大多数时候都是听医生指挥，然后就没有什么创造性，所以就不太喜欢。

09:41

你报的是检验，然后去护理，对条件对。然后他现在比较想去基础和预防，你觉得像现在有一个转变，从检验然后到现在想去基础预防，你觉得基础预防和检验这些最大的区别是什么？或者说和护理之间？我觉得他的你可以发挥的就是创造性更多，我觉得可以自己思考的东西更多。怎么说你可以做更多的事情，也也可以做更多的事情指的是哪方面？比如说你科研，然后发表文章什么的，我觉得还有一些你做出来一些成果的话，我觉得他自己带来的那种满足感也比较大，就成就感也比较多。

10:33

在护理没有让你感觉到这种成就感是吗？我觉得护士的工作感觉更像是一个工具，有的时候就是执行别人的命运，对我我不需要有什么自己的思考，我只要做别人让我做了什么事情就可以了，这是你的一个感觉，对不对？我行动准确吗？差不多这种感觉，有点没有什么创造力我感觉。你说你其实比较喜欢有创造性的工作，你自己在这一年就是大一你也快结束了。

11:09

这一年有探索过科研方面的什么实践吗或者之类的东西并没有，因为我也没有接触到这个机会，但是我有参加一个什么就是双创训练营，但是我发现我虽然参加了之后，感觉并不能学到特别多的东西，感觉还是有点模糊，然后也没有什么机会。

11:31

平常双创训练营是11万的，不知道好像是应该是我们学校的办的，因为那是一个也有其他学院的同学，我看到里面没什么收获，是指哪方面？学不到你想要学的东西，对，我感觉学的都是比较表层的东西，就没有说表层是指什么东西。我感觉他只是说给你一个框架那种感觉，然后并没有说是把里面的内容告诉你。你比较想希望从训练营里面得到什么东西？我就是我希望能够说说是能够实际的干一些什么，就是东西能够嗯不仅是停留在理论层面，就行了。

12:19

目前你说你想做比较创造一些东西，然后也尝试着去做了一个参加了一个双创的训练营，你自己有思考过自己。对对什么东西感兴趣吗？或者说对哪方面感兴趣，或者自己去探索这方面？不一定要参加别人组织的活动，就是你自己的课余时间有没有做过类似的事情？是关于学术的吗？医学的学术上面并没有，因为我感觉对大一的话，我觉得还是要把成绩给提高，是因为我觉得现在我的成绩就是说还是不是那么的。

12:57

现在其实你目前来说是有两个机会，一个是转专业，一个还有国重。其实你转专业也想去基础或者是预防，目前还没有想好对吧？但是就除了基础和预防，其他的基本上目前就已经不考虑了，是这个意思吗？其实技术和预防包括国重其实是进了同一个专业，所以你相当于要用这两次机会。假如说我举举个例子，你第一次转专业的时候，你已经成功的进了技术和预防，你后面还会再选择再去报国重。对会是吧？对。为什么？

13:36

因为国重的话，它里面我觉得它有很多更多的东西，它可以就是说会他说大学他会给什么导师，就是你可以选择导师，如果你是单什么进基础和预防的话，导师也是要自己找的，但是国重的话好像会有给就是说你选择不用自己再去找老师这样子。你是从什么途径了解到国中班的信息，就是我们班委发的一些文件上面看到的，就是关于国中班的一个介绍，这个文件是学校官网发布的，然后他下载下来发到你们群里的。

14:17

应该是的应该是辅导员发的。通知类似这样的文件，你有没有在自己再去找我们关于我们的信息没有看过。没有，我就直接在那上面看。

14:33

所以目前对你来说，活动班最吸引你的还是说他会给每个学生配相应的导师？这是比较新颖的一点是吗？其他的你可能就没有觉得对你来说可能没有什么吸引。力大的地方。或者买其他还有他的她好像对保研的话，还有转博士的话，他好像也有一些优先的东西，我看当时看的不是很仔细，但是我记得是有的。然后我觉得这点也特别的吸引我。

15:05

像导师和保研这两块东西比较新颖，你有和同学讨论过这些东西吗？他们也想去吗？我没有，我跟他们说过，但是我的舍友他们有文科生，文科生他们就是护理就是护理他们不能转，所以他们也没有太大的兴趣，对。文科也可以去活动。我们科可以治国，中国中对学科没有要求，所以你舍友其实都是文科生，所以他们其实本身就是一个性格的。对。

15:35

所以你其实和同学讨论了，可能就没讲几句就没了。你在未来的工作，你说护士工作比较的辛苦，你是不是对于呃，你对于未来的找工作有什么要求吗？找工作的话，他首先是经济上面，我觉得一定要就是说能够让我就做到经济独立，然后一方面还可以说对父母的话还是可以有些余额可以给父母这样子。

16:11

然后其外的话，我希望工作是我感兴趣的，不能够就是说是没有什么就一直重复的做同一件事情，我不喜欢就是说有一些创造性，还有说这个工作我不是很喜欢三班倒，像护士一样，需要不停的就是熬夜什么，就是说我可以自己主动性的熬夜，但是我不能够就是说被动的就是说让我去强制我。

16:44

刚刚你比如说你说你和同学之间有讨论过基础和预防他们的特性关系，其实基础和预防还是有一点点小区别的，对吧？嗯你你觉得预防的话好像是主要特点，它是可以跟从政比较有关系，然后基础的话好像是变更加的偏科研一点。你目前有倾向吗？还是觉得都可以？我就是还是都可以。因为预防也是可以做科研的，主要还是看到时候转的时候，在因为预防它可以转进的人数更多，但是但是基础的话就只能转3个人，预防的话可以转10个人，所以在考虑中还在所以是不是说基于这一点考虑，可能就是说可能预防的可能性稍微大一点，你会更倾向于报预防。

17:35

对对于这两个专业他们的不同来对你来说可能没有那么的大，就是说虽然预防可能从政多一点，基础从跟科研相关多一点，但是这个东西对你的影响不是很大，你还是比较关心能不能转出去。对。你想想转专业是刚进入到护理学院就有在想。对刚进护理就有在想，虽然我父母是觉得都可以，他觉得护士的话也是一个很不错的工作，对女孩子来说。

18:12

但是我自己的话首先本身就有一点不喜欢，然后查了一些护士的就是那些工作那些资料吗，然后我就更加的不太喜欢，然后上了大一上学期的课，然后有一些护理实践的课程，然后我也没有说特别感兴趣那种。

18:33

你想转专业有跟你父母讨论过，我有跟我父母讨论过，然后我的爸爸妈妈都是说他们也不是特别懂，然后他们就觉得根据我自己的喜好来，嗯所以他们也没有说一定要转或者不转，就是随你的听你的。

18:51

你有没有跟他们讲过你的职业规划这方面？

18:55

我有跟他们讲过这件事情，然后他们的话他们其实觉得我之前也跟他们说我想转技术的事情，然后我说我可能还要再读几年，就是说不可能可能要读比护理的话，我可能本科毕业我就可以去工作了，但是基础的话我可能要多读几年，然后我的父母就觉得女孩子不需要那么辛苦，他们觉得读读很多年的话可能太辛苦了，但是我自己的话我还是更加喜欢基础做科研这样的事情。

19:27

其实就是做科研的话，这个的话可能有一点可能和你未来的职业规划里面有一点点相违背的东西。举个例子，比如说你像做一个实验，可能我为了验证某一个东西存在或者不存在我，可能要做很多组的对照实验，这个其实要做非常多的重复性的工作，这个东西对你来说会是会是一个一个负面的影响作用。

19:57

我觉得不会，因为我平常做生理生化实验的时候也会有这样的情况吗？但是我觉得做实验的时候感觉自己是自己设计的实验，然后感觉自己在有自己的思考，在里面但是做起来会更有动力我觉得。

20:17

所以其实和护士工作性质重复的工作性质还是有一点不一样的。我明白了你还是比较喜欢嗯有自己思想参与的一个重复性的劳动在里面，你还是可以接受这个东西。基础它的时间比较长，你对于来说也没有什么太大的担心。对。然后好像国重的话，它是会给就是说每年它会发1万的对对补助，我觉得这点也是我比较详尽的点，因为毕竟如果我念很多年的话，对父母来说也是一个压力，毕竟其他的都已经开始在已经在工作了，但是我还在没有任何的收入的情况下，我觉得这点也是可以说对我比较吸引我的店。

21:17

但是你的父母不是说这就是说可能他们比较关心的，是你辛不辛苦的问题，可能对你经济方面好像也没有什么。是我自己是我自己对。你自己的想法觉得可以缓解一下他们的经济压力嗯。你有了解过保保研的政策吗？目前对我看到那上面说是首先是成绩，还有一个是成通过成绩，成绩特别好的话是可以保研，还有一个是发essay，是叫SCI，然后也是可以保研的。

21:51

然后有这两种途径你知道目前不是国中班的普通专业的保研的途径有哪些你知道吗？你有了解过吗？我听一个临床的时候好像也是发文章，如果你发的文章比较多的话，好像也是可以保研的。所以目前来说，其实你没有很仔细的去了解有哪些跟同学聊天这样子认识。

22:19

你获取信息的途径我可以说很多，其实是从和同学之间互相交流的比较多，你自己可能自己去搜的那那些，信息内容占的比较少的比例，你自己感觉一下，主要还是和别人聊天，然后来获取这些信息，像保研或者是国政班或者转专业这些东西，你都比较愿意和他们聊天来获取这些信息。

22:41

也有在一些社交平台上，也会有看到一些社交平台是指什么？像知乎知乎这类，我就有的时候会在上面看到一些关于就是说里面会有一些已经在工作的一些护士，他们会发帖子什么的，然后我会根据他们看到他们一些帖子，然后了解一些情况，就了解一下以后他们以后工作大概是什么样的状态，就和你自己想的是不是一样的，就类似这样来判断一下自己想不想学会。

23:13

从大一进刚进护理的时候，其实你就已经比较像转出去了。

23:19

当时其实可能还是想转回检验。对，当时还是说是转医学检验，当时我是想赶，然后后来是我通过就是说接触了一些更多东西，然后自己也在网上查了一下。然后我感觉可能预防和基础也是也不错。你们要转进基础和预防的话，成绩有要求吗？就是普通专业转专业的过程，只要不挂科就可以了。就可以申请，然后考试面试做检验的话，其实也可以保证经济独立，然后不用熬夜三班倒这种工作模式。

24:16

但是检验的话检验他最后给的是理学，好像他给的学位证书是不一样的，只有预防和基础他会给医学的学位证书，然后检验是给了理学的，你觉得医学学位学士学位和理学学位有什么区别吗？能不能做医生的区别？觉得是能不能做医生，然后你觉得拿了一个理学学士，本质上来说还不是医学生对，而且你不是说本身主要是他做的话可能是偏技术性的工作，但是如果是医学学位的话，做的应该是偏医科类的工作。

24:59

护理也是理学学士对吗？拿了医学学士学位，这个东西可以让你觉得更有认同感吗？还是对会有认同感。然后同时他以后做的工作也是不一样的，检验的话他出来做的还是偏技术性的一点。我觉得然后也我觉得偏技术性的都工作有的时候都像是工具，没有就是说思考性没有特别多。

25:46

我们举一个例子，就是几个月以后，9月份进入了国中班以后，已经开始分配导师了，你觉得你在留学导师的时候会有什么标准吗？还是说看安排。首先我是会看导师研究的东西，它是不是我比较感兴趣的，我可能会优先考虑我比较感兴趣的课题这样子就是选导师的时候，然后其他的话我倒没有那么多。

26:16

生殖医学重点实验室里面可能主要跟生殖相关，的你有了解过生殖相关的东西吗？我看了，他主要是说胚胎什么的，大概是那些东西。然后我还没有太了解，因为我现在学的都是我现在想按部就班的把所有的课程都给及格，然后进入所能考的分数稍微高一点，因为我觉得他如果你成绩更好的话，到时候转专业应该也是有优势的。

26:49

所以我现在更加是倾向于把专业知识都学好嗯，所以说你其实现在还是先考试，然后到暑假的时候再去了解这些东西，然后再进行转专业。到时候转专业的话要考两门。课里面学过吗？然后学过一个是牺牲，还有一个是细节。这两个你都学过。这个是上个学期考的还是学期考的？细细节是上学期的课，然后牺牲是这学期的课。当时你师姐考的考的还行，我考了80分不是很好，不是特别好，但也不是特别差，就属于那种到时候，你暑假重新看对我暑假因为我们护理学的细节，我们是护理学的叫人体解剖，还是反正跟临床他们学的是不一样的，然后我们到时候考的是更厚的一本，所以当时还是要重新学的。

27:55

像你们周围你有个同学讨论过转专业这个事情，像你们舍友他们有想转或者周围的人有他们都是想一般是想去哪里比较多，他们比较想去临床的比较多。想做医生对对临床有成绩的要求，他们他们自己目前成绩有达到那个要求吗？有达到的也没有达到。很多就是说没有达到的话，也有特别想转临床。

28:28

举个例子，比如说你进入了基础了，不是国中。其实你这样就像刚刚你同学说的一样，进入了专业，其实就要面临一个考研的问题，你有没有觉得这对你来说是一种负担呢？嗯是确实也是一种负担，但是我觉得他考研的话是必须的，如果我不能我会可能我，是肯定也是负担，但是我觉得也是必须要有的一个条件，毕竟如果不考研的话，就很难找到比较合适的工作嗯，但是相对于这种专业来说，像护理其实你本科毕业就可以找到工作，其实它还是有一定自己的优势的。

29:27

我我的个人感觉对。但是我觉得我父母他也是支持我，可以他首先经济基础也是有的，可以支持我继续念下去，然后我父母也支持我，遵从自己喜欢的，所以我觉得也不是特别大的负担。然后我也自己也有意愿想要去继续念下去，假如父母对我说假如父母对这一方面比较不支持，他们比较希望你去年护理下面找点工作，你会考虑继续待在护理，或者说那种本科毕业就能找到工作的专业，我可能会更加如果我的父母非常的说不支持的话，就觉得希望我本科就能找到工作的话，我可能也会我会比较考虑本科就医学检验这样的专业。

30:19

还是不会考虑护理，护理也会考虑，但是我还是会尝试着去转一转，如果转不成功的话，我就会好好的就是待在护理。

30:30

所以相对于护理来相对于护理来说，你觉得检验更大的优势是哪？一方面啊。他其实也是在做重复性，没有什么创造力的工作，他不用扎针什么的，这是最重要，不用扎针是什么意思？就是会被，人打针对不用打针，什么的为什么你会这么讨厌就是给人打针？不太喜欢，我自己本身也怕被别人打着，然后就对。不知道是心里还是什么，不太喜欢打针这件事，我可不可以理解为你选择就是科研方向的工作，有一部分原因也是你对临床操作这方面的一些排斥。

31:19

对，有一点对。像检验可能就不涉及临床上面的操作，还有医患关系这方面，自己也特别害怕，害怕和人起冲突。对因为我不是特别会交流那种，然后可能对就会有一点点担心。所以其实像这种科研环境不需要和太多人打交道。你有了解过这个信息吗？对不不需要说是其实也是蛮吸引你的一个。对。没有什么人际之间的矛盾。总结一下你们选择的就是转专业去从事科研方面有关的工作，我给你排了几个影响因素，啊嗯你之前有说到一个你提到的比较多的是有一个经济上的因素，包括奖学金，还有将来好就业之后，在工资上面的一些要求，然后你又提到了你自己的一些兴趣上的影响，对，然后还有一个工作环境的影响，大概就这三个对吧？

32:37

对。这三个如果让你排个序的话，你会怎么排呢？我觉得应该工作环境应该是第一个应该是兴趣，然后是工作环境，然后是工资，因为现在其实护士的话，她拿到的工资也是说也是非常可观的。然后主要还是兴趣还有是，工作环境，然后是自己你比较向往一个什么样的工作环境？我希望就是说首先可以自己安排，就是安排时间时间自由，对然后，其次是我觉得工作环境的话，我是希望不要就是说说有太多就是说像那种遇到那种什么就是人际的那种圆寂，然后其他的话也还好。

33:27

就一个就是工作时间，还有一个工作当中需要处理的一些人际关系，对就这两个事情考虑比较多的。

33:37

你说兴趣是你找工作比较主要的一个点，其实影响着你转专业的选择，包括选择技术预防和选择国重这一块。可以这么说，我假如说你以后再进入了果冻和技术预防以后，发现可能自己对我们就举个例子，你进了升值甚至过重，你进去以后发现其实对生殖这个东西并不是很感兴趣，你会选择退出。

34:09

应该还是说想想目前讲不出来，感觉不出来。对，我现在目前还没有想那么远。对，但是我觉得我应该不会退出。因为我觉得过重是一个很好的机会，我觉得还是值得去牢牢把握的机会，所以我不太是什么样的机会。他是你首先可以接触导师，然后你保研的话会有更多的有可能，所以我觉得这个是可能，如果我实在不太喜欢，我可能也会选择忍一忍，就是为了这两个条件一定会留在沟通的，这两个是非常吸引力进取和留下的一个条件，对。

34:51

其实接触导师的话，比如说和导师做实验或者和导师交流，导师的话一般是不太会改变自己的研究方向的，你和他去交流的话，可能他的他的兴趣点和你的兴趣点不是很重合的话，其实也是会有矛盾存在，我可不可以理解为你和更多的导师接触以后，其实对于你搞研来说是更有利一点的，因为他们会认识你这个人，会会会了解你这个人的科研素养和科研能力，对你这方面比较有帮助。

35:23

他就说你觉得和导师接触会更有利于发掘自己的兴趣点，你这两点跟哪个贴哪一个更贴近你的想法呢？觉得都有都有，二的话可能是二后者就是说就是你跟更多的导师去去了解以后，去去找自己的兴趣点到底在哪里。对，然后我也可以就是说见识的更多，我觉得我现在还是挺狭隘的。说有的时候你想见识更多哪方面东西，就是科研方面的东西是吧对，包括从做实验的步骤，基于实验操作的这些流程这些东西，你比较想自己去动手去做一下。

36:15

像之前你说的双创训练营就给你做，应该是内容形式应该是讲座。我猜的是对。除了讲座有其他的形式内容吗？没有，而且我们因为不是我们后来改成了是网上网课的形式，疫情期间是吧？其实到最后还是上课。你会觉得这种东西对你来说没有什么你有什么收获。对吧？对于保研的话，我不知道你知不知道在国中班保研的话是要保升值方向的。

36:59

研究生上面有写他是可能好像是本硕博好像都是要在升职这方面，对升职这方面。但你发现其实可能甚至不是你的兴趣点以后，你可能还会继续去做这方面的研究，继续去升职的研究生，继续做这方面的研究。虽然他可能跟你的兴趣不是很重合，所以可不可以说一旦出现了这种比较重大的矛盾以后，对于就业还是会给兴趣让步的，会优先考虑就业这方面，考研考博或者工作这方面。

37:44

嗯我并不是说一定要做我最感兴趣的那方面的事情对，但是在同等的条件下，我还是会愿意更选择自己。所以我可不可以再问一个问题，就是你目前有对自己兴趣点有明确的方向，应该也没有对吧没有目前也没有你还有问题吗。你目前在护理学院学习以来，有没有虽然你很想转专业，但有没有哪些瞬间是让你认可或许想要留下来的？还是说没有产生？有过，有的时候我上次就有一次我们做了一个国旗下的思政课，然后当时我就听陶连山老师，然后他讲的时候，我就感觉其实护士她是一个很有成就感的工作，然后他那一刻我就感觉还是他也是非常有意义的。

38:56

你们是你第一次对护士这个专业就是护理专业产生一些认同感是吗？对。但是怎么又磨灭掉了？我觉得一直我对护士都是有认同感的，他只是不适合我。他这个专业是好的，专业护士也是很好的一个职业，但是只是不适合，我觉得他不适合你，主要就是你刚刚说到的客户环境对还有很关注，而且我感觉我在这里可能做不好，能够成为一个特别好的护士，我只能成为一个合格的护士我感觉。

39:32

你刚刚说听陶连山讲了以后，觉得做故事比较有成就感，成就感是体现在哪方面？是他们在疫情期间的时候，有的时候别有一有病人可以认出他来，我觉得这一点病人认可你，我觉得这点就是成就感，获得病人的认可，你之前不是还讲完了医患沟通对啊对吧？其实比较恐惧，就是跟医患发生矛盾，但其实如果处理好这个关系，会给你带来一些成就感对，对吧？

40:07

对。陶老师讲的经历里面主要是他成功处理的一个人际关系，然后他工作中带来的一个成就感是他获得病人的认可，那么你其实也是希望说能够处理好这关，只是你觉得自己没有能力，很好处理这个关系，是有发生过什么样的事情让你觉得你处理不好？

40:35

这种情况首先我本身就是有一点点的涉恐的那种感觉，我不是很喜欢说就是说跟陌生人接触的那种，然后有的时候说话就不是很利索，就是感觉自己然后就感觉自己可能处理不好。

40:53

你有这种感觉是有没有具体发生过？比如举个例子发生过什么事情？

40:59

没有，我有的时候在一些聚会的时候，我就不是很喜欢，我喜欢坐边上，不是很喜欢就是说跟别人说话什么的，就是那种想，而且我平常也不怎么交友，不是很喜欢跟别人打交道。只是觉得自己是单纯的不喜欢还是？首先是不喜欢，因为我觉得很累，有的时候其实有的时候我就觉得聊天的时候我就觉得很累，有的说我们就可以去聊天，你们来报名。

41:35

这个是我感觉我可以了解一些国中班的事情，我来的时候我就觉得他这是一博中班的，所以我就过来了，而且他们聊的不一样，他们聊的大多数时候我觉得都是在抱怨什么的，我就听抱怨啊或者是讲一些说什么八卦，然后我听了就会很对，因为有的时候我也不了解，然后我就听见我就觉得不懂他们对不懂在讲什么，有的时候不懂他们在，讲什么就会有一点点的难受，我听你这样说，你是很想了解一下补充班的具体的一些信息是吗对，你有没有自己去通过找一些途径，我只看了那个文件，然后我听学姐说说我们上学期有就上一年有一个就是人他转到了国中，因为我刚开始看的时候看那个文件，我刚开始还有只有基础和预防的人能转，然后后来说那个学说之后又再去看了一下那个问题，然后发现护理也是可以转国中的。学姐是你的上一级护理专业，对。他现在还在护理。对他没有转成功。噢他是想去，但是没去成。对他是转临床没有转成功，但是转完临床其实你还可以再选择要不要去国中，他有去吗？

42:51

当时他好像没有噢对，你跟他聊了一下，就是没有去找他，跟你推荐说转成功的，没有互动就是不好意思，然后我也没有去找，然后我自己就在自己又看了一遍，然后我觉得还有还蛮吸引我的，社交场合的边缘化是你自己选择的还是？我自己的想法吧，我有的时候我觉得就不太好意思，有的时候也然后也不是很说话说的他们我说有的时候我说话有时候说不太清楚，不会，所以我自己也不怎么。

43:35

你有没有想到改变这种感觉？也有想改变，有的时候别人说说说你说话说不清楚，你还是会想改变。但是我说就比较难。而且我觉得现在也挺好的，说少就是说虽然我因为不太喜欢社交，所以我参加的学生工作什么的也比较少，然后我觉得这样子的话我也可以做自己更加感兴趣的事情。

44:06

觉得你目前比较感兴趣是哪些事情？

44:10

可以实现，首先是我喜欢看电影什么的，但是我上了大学之后看的真的比较少，然后还有说天文协会就是这样子。你说其实你有一点涉恐，但是你有没有想过在大一那个时候加入那么多学生社团，其实就是涉及到和很多人交流这种。

44:34

我本来一个都不想加入，然后后来是因为我在路上的时候遇到了一堆学学长，有一群学长学姐在搬器材，然后我当时看到他们在搬天文望远镜，然后我就特别的感兴趣，然后我就很那个时候主动的去找他们搭讪的时候，我说我可以帮你们搬，然后我就帮他们搬了，搬到那边，然后他们就让我用天文望远镜看大的那个，星星。

45:03

然后当时我就特别的开心，然后这个事情的时候都变得开心，因为我一直都很想要有这个机会，但是我之前从来没有过就是说亲眼的看到天文望远镜，然后亲所以我当时就很激动，然后我就过去找他们，我说我可以帮你们搬这样子。

45:23

你其实加入这个社团也是因为自己感兴趣，对。

45:27

但是其实也是蛮偶然的，你要是没有看到他们在搬东西，你可能都不会加入到这些手段，对，因为我可能都不知道，因为当时他们就是社团摆摆就是出来摆摊，就是那种的时候我都没有去看，我说后来那天晚上我去跑步的时候，我看到他们在那搬东西搬到操场去，然后我就看到了，然后我就去帮他们搬了，你一直想看这些心境是有什么事情或者什么东西，让你其他的兴趣还是说只是比较好奇而已？

45:54

是从小就有了这个东西，还是说在那个时候看到这个东西就想去用一下试一下这个事情？很久之前也不是很小的时候，可能是初高中的时候，我感觉比较对，很喜欢这些东西，然后我当时看到他拿着天文望远镜，然后我也有一点好奇，因为我之前也没从来没试过，然后想看看到底是什么感觉那样看。

46:19

所以你像你平时参加天文社团的也蛮活动也蛮多的，也不是特别多，我他们有啥活动，他们有的时候会组织一起看电影，然后或者一起组织去看星星，然后我也就那一次用过天文望远镜，后来我也就不太敢了，后来来就因为他们有的时候说是要一起去什么的时候，我就有点不敢感觉，因为我就觉得我一个他们好像都认识，然后我一个人就不太认识的，过去就感觉有点点的。

46:53

对，尴尬。你觉得不太认识？他们在发动你们应该是有一种自己的群之类的，发动自己去活动，然后你基本上都不太会去。对，但是有一次有的时候他们看电影就那种很多人的时候，我又又会去不需要和大家都安静的坐在那里，没有人际交流的时候你还是愿意去。如果是178个人那种的话，我就不太愿意去，如果是很多人的话，我就会愿意去。

47:26

其实这里其实会不会也影响到你工作上的选择，你比较不愿意选择一个人的那种工作，不太愿意选择需要人际太多人交流的工作，这其实跟你的性格比较有关。知道了。其实没有考虑过这一点是吧？他们都说我是那种就是说如果那个人就不太熟悉，我的人可能会觉得我还蛮嗯就是，不爱讲话，但是如果熟的话就会比较爱讲话。

47:58

你说之前和周围同学在一起，老师听他们抱怨，他们抱怨的是什么东西，是学习吗还是？对，主要还是学习，他们觉得医学生特别苦特别累这样子，大概也就是这些。

48:16

你觉得给你带来太多负能量了吗？也还好，我觉得。也没有说会带来太多负能量这样子，我觉得有的时候自己也会觉得太累，但是也没有说是负能量。这种没有。所以我大概总结一下，其实你现在目前还没有明确的说自己喜欢哪一个科研方向或者是哪一个学科，你像你现在进国中主要是还想进去，通过学校给你分配导师来探索自己的科研兴趣，目前是这么一个想法。当然保研也是很重要的一个目标，这样可以促使你进一步实现自己这个方面的理想。

49:00

假如说转失败，你有考虑过这个事情吗？还是说没有考虑？

49:06

转失败了，其实就是因为要在护理，我就好好的就是对接受，或者说你有没有想过另外一种方案就是稳妥一点就是，你转专业的时候进行检验，国中的时候就冲一冲这种类似就是我一定不能留在湖里这种，有想过吗？还没有一心要进行护理基础和预防。

49:33

对，而且我觉得预防的话算是比较好进的，因为它可以转10个人。我觉得这块比较好，你自己想出来还是跟学姐她问？他告诉你的我自己想的。你目前还没有了解过以前转专业的情况，我了解过。我知道，但是但是我觉得他们不能说是怎么作为参考什么的，而且我们之前上一年他也是预防也是10个人，然后里面有7个是我们护理的，所以我觉得进的概率还是比较大的。

50:11

你目前就对于这个事情转专业和果冻这两个事情其实就跟学姐讨论过，比较多一点。跟同学其实讨论的他们都是文科生讲着讲着也讲不下去了。对我还跟一个朋友也讨论过，这个朋友是社团里别的专业的朋友吗？

50:28

对，然后他跟我说说国中班是就是说很厉害什么的，说里面的人就是说说如果他说让我可以试试看，他说如果他觉得这个还是很厉害的什么的，国中班很厉害，你有会一想过自己进了博士班，可能会成绩排名比较靠后一件事情，对我想过，因为我觉得我还是不是那么的那个我，觉得有点有的时候还比较懒惰的样子，然后我就想可能会会排名靠后，但是我想着如果我一直在一个都是分数都比较低的一个团队里，我的分数可能也不是那么高，但是如果再一个就是竞争性比较大，然后大家分数都很好的地方的话，我觉得我也是督促我可以我自己可能也会去努力一下，所以其实你还是可以接受这一点。

51:20

就成绩排名可能会往后靠，但是会会更加激励你去努力的学习，你还是可以接受自己排名，可能会稍微考核一点现实。

51:33

你说护理工作可能会比较辛苦，其实你有没有考虑过，其实科研工作其实也挺辛苦的。对，但是这两个辛苦不是同一种辛苦，他可以自己控制时间的，一种是自发性的，一种就是说感觉是被迫的一样。他说我可以儿子，但我不可以被迫你可以自己去熬夜。对。

52:14

我还想问一个点，你有没有想过，其实科研它方面，你的压力是精神上面的压力，护理他可能就是说你必须要在半夜去工作这方面生理上的压力，但其实他做的是重复的简单的工作，他在精神上没有很多的压力，精神上也有有什么样的压力？

52:36

首先有一个叫做专业名词我有点忘了，反正如果产品的操作，职业暴露对职业暴露，还有一个是医患关系，我觉得这都是，这对你来说都是经济上的压力。

52:53

所以其实你觉得做科研，比如说也可能会面临比如说做实验失败，或者说整个课题进行不下去这种情况，这种竞争下的压力，其实你觉得相对于护理来说，你还是更能接受这方面的压力是吗？

53:10

你有考虑过我刚刚讲的这方面的东西吗？因为我就是认识一个临床的朋友，他就现在在写文章，他怎么样也写不出来，他觉得然后我就感觉我看到，然后我就觉得确实还蛮辛苦的，但其实这个也没有让你改变你的想法，对没有？我没有要跟他。好。我们就到这。你稍微等一下。多久。
