# Supplementary material for: Why do you choose this program?—A decision-making model of medical students based on grounded theory
Source: PLoS One. 2023 Sep 15;18(9):e0291634. doi: 10.1371/journal.pone.0291634 (PMC10503722; doi:10.1371/journal.pone.0291634)
Supplement: S1 File — (ZIP) [file pone.0291634.s001.zip › RAW DATA/P12.docx]

00:00
This process and rationality of a choice situation. But then before this interview, I have to read the moral and ethical guidelines. In this interview, the interviewees participated on the principle of equality and voluntariness. The interviewees must truthfully express their thoughts and cognitions, and confirm that they meet the social security requirements. condition. During the interview or recording, the recording will be anonymously used for scientific research and will not be disclosed to any third party. During and after the interview, you have the right to cancel the right to use the researcher's recording. Do you know and agree? ?

00:34
I figured it out.

00:37
Tell me about your grade and major first. I am a freshman, and my major is nursing. What is your grade? location? The grade rank is 110 meters. How many of you are there in total? In total, there are more than 250 people in our profession, and more than 250 people. it is good. Are you currently doing this in the propaganda country? The class committee has issued those related documents before, about the recruitment of activity classes, have you read them?

01:19
There are lectures and things like that, aren't they? This document was also issued last month. When was it posted? It seems to have been a while, and it seems that last month, it was about the transfer of the 19th grade high school class. Did you discuss it with your classmates after reading it? Have. Because I'm quite interested, yes. Are you more interested personally, or do you think the students around you are very interested? I am personally more interested.

01:52
Can you tell me why, because first of all, I was in nursing because of the transfer, so I was not so interested in this major at that time. Then, in my future career, I feel that I will be more interested in scientific research, so I would like to change my major. Then I saw that it is possible to change majors in the middle school class, so I was quite interested.

02:18
What subject did you apply for when you were in high school? I applied for a medical test. Then my first choice was medical testing, and then I was transferred to nursing. You should be able to apply for 6 here, yes. You get one. I checked the test, and then the report was reported later, I don't remember, and then the nursing is ranked fifth or the first, and then it's not anyway. Anyway, this is not the ability you wanted to enter in the first place. right. In addition to testing whether you have any other majors that you would like to enter, now we are particularly looking to enter the basics. The basics are right, and the basics and testing are currently the two majors that you want to go to.

03:11
Can I ask you when you were in high school, when you signed up for college, were you a science student or a liberal arts student or a science student, did you apply for medicine or I just applied for one? Department, my first major is Nanjing Medical University, and my second major is Nanjing Chinese Medicine.

03:32
Can you tell me why? Because I talked about the process at the time, not the reason, but the process of making a decision. First of all, my parents. My parents **wanted** me to work in medicine. At that time, they thought it was a more stable job. Then I thought it was more realistic, maybe it was nursing, and there was a normal school, but I didn't like being a teacher very much, and then I was more **interested in medicine** , and my high school selection was silent, so I applied for the exam Specialized in medicine.

04:11
So can it be said that the parents first helped you determine a scope, medical or normal.

04:16
Yes, and then you chose a subject, probably the process is like this, right? It’s also true for myself. My parents and I have always expressed my preference for medicine when I was in my second year of high school, so I only chose 50, so I didn’t consider engineering at that time. Or you didn't consider things like science, and then you signed up for the medical department, got an examination, a bonus examination, and finally got into nursing, right?

04:53 Right
. Your current grades should meet the conditions for entering high school grades, right? right. Well, when you discuss with your classmates around you, it's alright, do you sit here or there? I'm here, are you waiting here? You don't have a chair here, I don't mean it to him. May I ask, um, have you considered changing majors? I'm thinking about changing majors. Have you received this notification so far? Have. Then have you considered which major to transfer to? I have to consider whether I want to transfer to the foundation or the foundation, right.

05:43
Basics and prevention, because the number of people who can be transferred to prevent it from being transferred is relatively large, so I feel that preventing transfer may mean a higher probability of being able to transfer. When you started high school, you applied for inspection. Why do you suddenly have to apply for the two majors of basic and or prevention? Because, when I was in high school, I didn't mean to understand, it was me After I went to this university, I only learned about these two majors, and then I also had some preventive and basic classmates around me, and then I felt that I was still more interested in it.

06:17
Can you tell me where you learned about your major? I just proposed to put these things first because I took English classes with my classmates in the prevention class, and then sometimes we would talk about things like what their majors were doing. Then there are some of my other friends, that is, classmates who know what kind of classmates they are, whether they are high school classmates or not college classmates?

06:45
How do you know these universities? Some of the classmates in some departments, some from the department, and some from the clubs, which clubs have you joined, or people who can apply for the outer court? Astronomical Association, then I will know a lot of foreign aid, and sometimes I do volunteer activities, and I will also meet some foreign aid, youth league committees, and volunteer evaluations, yes. Is he nursing? You are in care management. Are you a big couple now? Is it right that you are considering changing majors? Consider changing basics or prevention.

07:22
You just said that in English class, you can discuss with other students about their majors, such as discussing what their majors do. You can talk about your understanding of basic prevention and what they can do in the future. The main thing is prevention. It is working in the CDC, and then you can also enter the research room, that is, to do scientific research and enter the laboratory, and then the basics seem to be entering the laboratory.

07:45
Then I listened to a senior student talking about the basics. If he graduated from an undergraduate degree, if he did not have that job, he would be unemployed after graduation. He had such a statement. Then I can mainly follow some experiments and publish articles, and then I will not be motivated to work in the future, and I will be so tired when I enter the hospital. It seems that I can control the time, operate the foundation or prevent it myself.

08:14
You just felt like you missed something. Did you consider what attracted you when you applied for the test in high school? Or do you just apply casually or if the major matches your score? The first is that the major matches my score, and then I want to work in the hospital. I feel as if the job is relatively stable.

08:37
Then it is easy to find a job. I think it is easier to find a job. Majors such as nursing are actually quite stable. Being a nurse in a hospital or entering the laboratory department of a hospital is actually a nature, but if I feel that nursing is It’s quite tiring, he is very hard, and sometimes I feel that when I take some nursing experimental courses, I feel that I am not so interested in this major, that is, if I am allowed to continue to work in the field of nursing in the future , I feel a little uncomfortable, and he has to work three shifts, and then I can't accept this. The nurse is more difficult, and then I am not interested, I am interested.

09:19
Talk about something specific? Or a general feeling? I think some of his work is very cumbersome, too cumbersome, and not very creative. I feel that most of the time I follow the doctor's instructions, and then there is no creativity, so I don't like it very much.

09:41
You reported for an inspection, and then went to nursing, the conditions were right. Then he wants to go to basics and prevention now. You think there is a change now. From inspection to now, he wants to go to basic prevention. What do you think is the biggest difference between basic prevention and inspection? Or between nursing and nursing? I think what you can do with him is to be more creative, and I think you can think more about yourself. How do you say you can do more, and you can do more? What does it mean? For example, if you do scientific research and then publish articles or something, I think that if you produce some results, I think the sense of satisfaction brought by him is also greater, and the sense of accomplishment is also greater.

10:33 Being
in nursing doesn't give you that sense of accomplishment, does it? I think the job of a nurse feels more like a tool. Sometimes it is to carry out other people's destiny. I don't need to think about me. I just need to do what others ask me to do. It's yours A feeling, right? Am I acting correctly? Almost this feeling, a little bit uncreative I feel. You said that you actually prefer creative work. You are a freshman in this year and you are almost finished.

11:09
Have you explored any practice in scientific research this year or something like that, because I have not been exposed to this opportunity, but I have participated in a training camp for entrepreneurship and innovation, but I found that although I participated in After that, I didn't feel like I could learn much, I still felt a little vague, and then I didn't have much chance.

11:31
The usual mass entrepreneurship and innovation training camp is 110,000. I don’t know if it should be run by our school, because it was a student from another college. I didn’t see anything in it. What do you mean? If you can’t learn what you want to learn, yes, I feel that what you learn is all superficial, so I don’t say what superficial means. I feel that he just said that he gave you the feeling of a frame, and then he didn't say that he told you the content inside. What do you want to get out of the training camp? I just want to be able to say that it can actually do something, that is, things can be, not only at the theoretical level, that's fine.

12:19
At present, you said that you want to compare and create something, and then you also tried to do a training camp to participate in a dual-creation training camp. You have thought about yourself. Interested in something? Or what areas are you interested in, or explore this area yourself? You don't necessarily have to participate in activities organized by others, that is, have you done similar things in your spare time? Is it about academics? There is no academic aspect of medicine, because I feel that for the freshman year, I think I still need to improve my grades, because I think my grades are still not so good.

12:57
Now you actually have two opportunities, one is to change majors, and the other is to have a national weight. In fact, you also want to go to the basics or prevention when you change your major, but you haven't thought about it yet, right? But apart from the basics and prevention, the others are basically not considered at present. Is this what you mean? In fact, technology and prevention, including Guozhong, are actually entering the same major, so you are equivalent to using these two opportunities. If I give an example, when you changed your major for the first time, you have successfully entered technology and prevention, and you will choose to serve the country again later. right? right. Why?

13:36
Because of the national weight, I think there are many more things in it. It can be said that he will say what kind of tutors he will give to the university, that is, you can choose tutors, if you are only in the basics and prevention , the tutor also has to be found by yourself, but Guozhong's words seem to be given, that is, if you choose not to find the teacher yourself. How did you learn about the information about the middle school class? It is what we saw on some documents issued by our class committee, which is an introduction to the middle school class. This document was published on the school's official website, and then he downloaded it and sent it to you. in the group.

14:17
should be, should be sent by the counselor. Notice similar to this document, have you ever looked for us on your own and haven't read the information about us. No, I'll just watch it right there.

14:33
So for you, what attracts you most about the activity class is that he will assign each student a corresponding tutor? This is relatively new, isn't it? Others you may not feel may not be attractive to you. Powerful place. Or if she buys other things with him, she seems to be interested in Baoyan, and if she is transferred to a Ph.D., he also seems to have some priorities. I didn't read it very carefully at the time, but I remember that there were. And then I think that's what particularly appeals to me.

15:05
The two things like mentors and Baoyan are relatively new. Have you discussed these things with your classmates? Do they want to go too? I didn't, I told them, but my roommates have liberal arts students, and the liberal arts students are nursing or nursing, and they can't transfer, so they don't have much interest, yes. Liberal arts can also go to activities. Our subjects can govern the country. There are no requirements for subjects in China, so your roommates are actually liberal arts students, so they are actually a character in themselves. right.

15:35
So you actually discussed it with your classmates, maybe you didn't say a few words. In your future job, you said that the nurse's job is relatively hard. Do you have any requirements for, er, your job search in the future? If he is looking for a job, he must first be financially. I think that it must be able to make me financially independent, and then on the one hand, I can say that I can still have some balance for my parents.

16:11
In addition, I hope that the work is what I am interested in. If I can't, it means doing the same thing over and over again without anything. If I don't like it, it means there is some creativity, and I am not very interested in this work. I like to work in three shifts, like a nurse, all I need to keep up is staying up late, which means I can take the initiative to stay up late, but I can’t just say passive means let me force me.

16:44
Just now, for example, you said that you and your classmates have discussed the relationship between foundation and prevention. In fact, there is a little difference between foundation and prevention, right? Well, you think that prevention seems to be the main feature. It can be related to politics, and then the basics seem to be more scientific. Are you currently inclined? Still think it's okay? I just can. Because prevention can also be used for scientific research, the main thing is to transfer when you see it. The number of people who can be transferred because of prevention is more, but for the basics, only 3 people can be transferred, and 10 people can be transferred for prevention, so I am considering So, based on this consideration, it may mean that the possibility of prevention is slightly higher, and you will be more inclined to report prevention.

17:35
Yes, the differences between these two majors may not be that big for you. That is to say, although the prevention may be a little more political and the foundation is more related to scientific research, this thing does not have a great impact on you. , you are still more concerned about whether you can transfer out. right. When you think about changing majors, you are thinking about it as soon as you enter the nursing school. When I first entered nursing, I was thinking about it. Although my parents thought it was all right, he thought that being a nurse would be a very good job for girls.

18:12
But I didn't like it at first, and then I checked some nurses' information about those jobs, and then I didn't like it even more. Then I took a class in the first semester of my freshman year, and then there were some Nursing practice courses, and then I didn't say I was particularly interested in that kind.

18:33
You have discussed with your parents if you want to change majors, I have discussed with my parents, and then my parents said that they don’t know much either, and then they thought it was based on my own preferences, um, so they It doesn't say that you have to turn or not, it just follows you.

18:51
Have you talked to them about this aspect of your career plan?

18:55
I have told them about this, and then they said that they actually felt that I had told them before that I wanted to transfer technology, and then I said that I may have to study for a few more years, which means that it is impossible to If I study more than nursing, I may be able to go to work after graduation, but I may have to study for a few more years for the basics, and then my parents think that girls do not need to work so hard, they think that studying for many years may be too hard Yes, but in my own words, I still prefer things like basic scientific research.

19:27
In fact, if you are doing scientific research, this may be a little bit contrary to your future career plan. For example, for example, if you are doing an experiment, maybe in order to verify the existence or non-existence of a certain thing, I may do many groups of controlled experiments, which actually requires a lot of repetitive work. It will be a negative impact.

19:57
I don't think so, because I usually do the same thing when I do physiological and biochemical experiments? But I feel that when I do the experiment, I feel that I am an experiment designed by myself, and then I feel that I have my own thinking, but it will be more motivated when I do it.

20:17
So in fact, the nature of the work that repeats the nature of the work of nurses is a little different. I understand that you still prefer a repetitive labor with your own thoughts involved, and you can still accept this. It takes a long time to base it, and you don't have to worry too much about it. right. Then it seems that if the country is heavy, it will give 10,000 subsidy every year. I think this is also my more detailed point, because after all, if I study for many years, it will also be a pressure for my parents. , after all, the others have already started to work, but I still don’t have any income. I think this is also a store that is more attractive to me.

21:17
But your parents don't say that it means that maybe they are more concerned about your hard work, maybe it doesn't seem to matter to you financially. It's me, it's me. Your own thoughts feel that you can relieve their financial pressure. Do you know the policy of Baobaoyan? At present, I have seen that the first is the grade, and the other is the passing grade. If the grade is particularly good, it can be guaranteed for research, and the other is to send an essay, called SCI, and then it can also be guaranteed.

21:51
Then there are these two ways. Do you know what are the ways of postgraduate research for ordinary majors who are not in middle school classes? Did you know? When I listen to a clinic, it seems that I also publish articles. If you publish a lot of articles, it seems that you can also protect the research. Therefore, at present, in fact, you have not been very careful to understand which ones are chatting with classmates like this.

22:19
I can say a lot about the ways you get information. In fact, you communicate more with your classmates. For those you may search for yourself, the information content accounts for a relatively small proportion. You can feel it yourself. The main thing is to chat with others, and then come to get this information, such as Baoyan or national affairs class or change majors, you are more willing to chat with them to get this information.

22:41
There are also some social platforms, and there are also some social platforms. What does it mean? Like Zhihu Zhihu, sometimes I will see some information on it, that is, there will be some nurses who are already working, they will post something, and then I will see some of their posts based on them , and then understand some situations, and then understand what kind of state they will be working in in the future, whether it is the same as what you think, and like this to judge whether you want to learn or not.

23:13
When you entered nursing from freshman year, it was actually more like you were transferred out.

23:19
At that time, he might actually want to switch back to testing. Yes, it was still said to be transferred to a medical examination at the time. At that time, I wanted to catch up, and then I passed it, which means I had access to some more things, and then I also checked it online. Then I feel that maybe prevention and foundation are also good. If you want to transfer to basic and prevention, are there any grade requirements? It is the process of transferring from an ordinary major to a major, as long as you don't fail the course. If you can apply, and then take the test and interview, you can actually ensure financial independence, and then you don’t have to stay up late and work three shifts.

24:16
But in the test, he finally gave science, as if the degree certificate he gave was different, only prevention and basic he would give a medical degree, and then the test was for science, do you think the medical degree is bachelor Is there any difference between a degree and a science degree? Can it be the difference between being a doctor? Think about whether you can be a doctor, and then you think that if you get a bachelor of science, you are not a medical student in essence, and you are not saying that it may be technical work if you are mainly doing it, but if it is a medical degree, do should be part of medical work.

24:59
Nursing is also a BSc right? With a bachelor of medicine degree, does this thing make you feel more identified? Still have a sense of identity. At the same time, the work he will do in the future will also be different. If he is tested, what he does is still a bit technical. I think and then I also think that all work that is more technical is sometimes like a tool. If it is not, it means that there is not much thinking.

25:46
Let's take an example, that is, after a few months, after entering the junior high school class in September, the assignment of tutors has already begun. Do you think you will have any standards when studying abroad as tutors? Or look at the arrangement. First of all, I will look at the research of my supervisor, whether it is more interesting to me, I may give priority to the topics that I am more interested in. This is when choosing a supervisor, and then I don’t have that many other things.

26:16 The
key laboratory of reproductive medicine may be mainly related to reproduction. Have you ever learned anything related to reproduction? I saw that he was mainly talking about embryos or something, probably those things. Then I don't know much about it, because what I'm studying now is that I want to pass all the courses step by step, and then get a slightly higher score in the entrance exam, because I think if you get better grades, you can go to It may also be an advantage to switch majors.

26:49
So I am more inclined to learn all the professional knowledge, so you should take the exam first, and then learn about these things during the summer vacation, and then change the major. At that time, if you change majors, you will have to take two exams. Did you study in class? Then I learned that one is sacrifice, and the other is details. You have learned both. Was this taken last semester or last semester? Details are last semester's class, and sacrifice is this semester's class. At that time, your sister did well in the exam. I got 80 points in the exam, not very good, not particularly good, but not particularly bad. It is the kind of time when you re-look at me during the summer vacation because of the details of our nursing study. We study nursing called human anatomy, or is it different from what they study in clinical practice, and then we took a thicker book, so we still had to study it again.

27:55
Like you have a classmate around you who discussed the matter of changing majors, like your roommates, they want to change, or people around them all want to go to where they want to go, and they want to go to clinical clinics more. . Want to be a doctor's requirements for clinical performance, do they have their current performance to meet that requirement? What has been achieved has not been achieved. Many say that if they have not achieved it, there are also those who especially want to transfer to the clinic.

28:28
For example, for example, you have entered the foundation, not the middle school. In fact, just like what your classmates said just now, when you enter the major, you will actually face a problem of postgraduate entrance examination. Do you think this is a burden for you? Well, it is indeed a burden, but I think it is necessary for him to take the postgraduate entrance examination. If I can't, I will be possible. It must be a burden, but I think it is also a necessary condition. After all, if you do not take the postgraduate entrance examination, it will be very difficult It is difficult to find a more suitable job, but compared with this kind of major, like nursing, you can actually find a job after graduating from an undergraduate degree. In fact, it still has its own advantages.

29:27
I personally feel right. But I think my parents also support me. First of all, they have the financial foundation and can support me to continue reading. Then my parents also support me and follow what they like, so I don't think it is a particularly big burden. Then I also have the will to continue reading. If my parents told me that if my parents were less supportive in this aspect, they would prefer that you find a job in nursing last year, and you would consider staying in nursing, or that This is a major that can find a job after graduation. I may even think that if my parents are very unsupportive, I think I hope that I can find a job after graduation. I may also consider medical examination as an undergraduate. specialized.

30:19 I
still don’t think about nursing, and I will consider nursing, but I will still try to take a turn. If the transfer is unsuccessful, I will just stay in nursing.

30:30
So compared to nursing, what do you think is the bigger advantage of testing? On the one hand. In fact, he is also doing repetitive, non-creative work. He does not need to stick needles or anything. This is the most important thing. What does it mean to not need needles? It's just that you'll be targeted. People don't need injections. Why do you hate giving people injections so much? I don't like it very much, and I myself am afraid of being beaten by others, so that's right. I don’t know if it’s in my heart or what. I don’t like injections very much. Can I understand that your choice is scientific research, and part of the reason is your rejection of clinical operations.

31:19 Right
, a little bit right. For example, the test may not involve clinical operations, as well as the doctor-patient relationship. I am also particularly afraid of conflicts with others. Yes, because I am not particularly good at communicating, and then maybe I will be a little worried. So in fact, such a scientific research environment does not need to deal with too many people. Did you know this information? Needless to say, yes, it is actually quite attractive to you. right. There is no interpersonal conflict. To sum up, what you have chosen is to change your major to engage in scientific research. I have listed several influencing factors for you. Ah, you mentioned before that there is an economic factor that you have mentioned more often, including Scholarships, and some requirements on salary after a good job in the future, and then you mentioned some of the influence of your own interests, yes, and then there is the influence of the working environment, about these three, right?

32:37 Right
. If you were asked to rank these three, how would you rank them? I think that the work environment should be the first interest, then the work environment, and then the salary, because now in fact, the salary she gets is also very impressive. Then the main interest is still, the work environment, and then what kind of work environment do you yearn for? I hope that it means that you can arrange yourself first, that is to arrange time and time freely, and then, secondly, if I think about the working environment, I hope not to say that there are too many, that is to say that it is like the kind of encounter that is interpersonal. Kind of passed away, and then everything else is fine.

33:27
One is working time, and another is some interpersonal relationships that need to be dealt with during work, and I think more about these two things.

33:37
You said that interest is one of the main points of your job search, but it actually affects your choice of majors, including the choice of technical prevention and the choice of national weight. It can be said that if I say that after you enter jelly and technical prevention in the future, you may find that you may give us an example. You enter the appreciation or even overweight, and after you enter, you find that you are actually not very interested in reproduction. , you will opt out.

34:09 I
should still say that I can't talk about it, I can't feel it. Yes, I haven't thought that far right now. Yes, but I don't think I should quit. Because I think being overweight is a good opportunity, and I think it's still an opportunity worth taking firmly, so I'm not quite an opportunity. He is the first for you to contact the mentor, and then you will have more possibilities if you are in the research, so I think this is possible, if I really don't like it, I may choose to endure it, just for these two conditions Will definitely stay in communication, these two are very attractive and enterprising and stay a condition, yes.

34:51
In fact, if you talk to your supervisor, for example, do experiments with your supervisor or communicate with your supervisor, your supervisor will generally not change your research direction. If you communicate with him, his interests may be the same as yours. If the points of interest are not very overlapping, there will actually be contradictions. Can I understand that after you get in touch with more mentors, it will actually be more beneficial for you to do research, because they will know you as a person and will It will be helpful for you to understand your scientific research literacy and scientific research ability.

35:23
He said that you think contacting with your mentor will be more conducive to discovering your own interests. Which of these two points is closer to your thoughts? I think there are all of them. The second may be the second. The latter means that after you get to know more about the mentors, go to find out where your interests are. Yes, and then I can also say that I have more knowledge, I think I am still quite narrow. It is said that sometimes you want to see more things, that is, scientific research, right, including the steps of doing experiments, and these processes based on experimental operations, you prefer to do it yourself.

36:15
Like you said before, the dual-creation training camp will do it for you. It should be a lecture in the form of content. I guessed right. Are there any other forms of content besides lectures? No, and because we were not, we later changed to the form of online courses, during the epidemic, right? In fact, at the end of the class. You will feel that this kind of thing has nothing to you and you have nothing to gain. Right? As for the postgraduate research, I don't know if you know that if you are in the middle school class, the postgraduate research is to ensure the appreciation direction.

36:59 The
postgraduate wrote that he might be a bachelor, master or doctor. It seems that they are all about promotion and promotion. But after you find that it may not even be your interest, you may continue to do research in this area, continue to go to graduate students who are promoted, and continue to do research in this area. Although he may not have your interests very much, can it be said that once such a relatively major conflict arises, he will still give in to his interest in employment, and will give priority to employment, postgraduate entrance examinations, or work.

37:44
Well, I'm not saying that I have to do what I am most interested in, but under the same conditions, I would still be more willing to choose myself. So can I ask another question, that is, you currently have a clear direction for your interests, so there shouldn't be any problems, right? Since you are currently studying in the School of Nursing, are there any moments that make you recognize or want to stay even though you really want to change your major? Or did it not happen? Yes, sometimes I did an ideological and political class under the national flag last time, and then I listened to Teacher Tao Lianshan, and when he talked, I felt that the nurse was actually a very accomplished nurse. Work, and then I felt at that moment that he was also very meaningful.

38:56
This is the first time you feel that the profession of nursing is the nursing profession, isn't it? right. But how did it get wiped out again? I think I've always identified with the nurse, he just wasn't right for me. His profession is good, and professional nurse is also a good profession, but it is just not suitable. I think he is not suitable for you. The main reason is that the customer environment you just mentioned is still very concerned, and I feel that I may be here. If I can't do well, I can become a particularly good nurse, I can only become a qualified nurse I feel.

39:32
You just said that after listening to Tao Lianshan’s talk, you feel that making a story is more fulfilling. In what way is the sense of accomplishment reflected? When they were in the epidemic, sometimes there was no patient who could recognize him. I think that the patient recognizes you. I think this is a sense of achievement, to gain the recognition of the patient. Did you finish talking about the doctor and patient before? Communication is right, right? In fact, it is more fearful, that is, conflicts with doctors and patients, but in fact, if you handle this relationship well, it will bring you some sense of accomplishment, right?

40:07 Right
. The experience Mr. Tao talks about is mainly about a relationship he successfully handled, and then a sense of achievement brought about by his work is that he is recognized by the patient, so you actually hope that you can handle this well, but you feel that you have not. Ability to handle this relationship very well, is there anything that has happened that makes you think you can't handle it well?

40:35
In this situation, first of all, I have a little bit of a feeling of being involved in fear. I don’t like to say it very much, but it is the kind of contact with strangers. Then sometimes I don’t speak very neatly, I just feel myself and then I feel like I might not be able to handle it.

40:53
Do you feel this way has it happened specifically? For example, what happened?

40:59
No, sometimes I don't like it very much at some gatherings. I like to sit on the side. If I don't like it very much, I just talk to others, that's what I think, and I don't usually make friends. , I don't like dealing with other people very much. I just think I just don't like it or not? First of all, I don't like it, because I feel very tired. Sometimes I actually feel very tired when chatting, and some say we can chat and you can sign up.

41:35
This is because I feel that I can understand some things about the junior high school class. When I came, I thought he was a junior high school student from Yibo, so I came over, and they talked differently. They talked a lot. Most of the time I feel like I'm complaining or something, I just listen to the complaints or talk about some gossip, and then I hear it and it's right, because sometimes I don't understand it, and then when I hear it, I don't think so. If you know what they are talking about, you don’t know what they are talking about. Sometimes you will feel a little uncomfortable when you don’t know what they are talking about. I heard you say this. You really want to know some specific information about the supplementary class, right? Did you go through some ways by yourself? I only read that document, and then I heard from my senior sister that we had one last semester, and last year, there was one person who was transferred to a middle school, because I read it when I first started reading it. That document, at first, there were only basic and preventive people who could transfer it. Later, after I said that theory, I went to look at the question again, and found that nursing can also be transferred to middle school. Senior Sister is your upper-level nursing major, yes. He is still nursing. It didn't turn out to be successful for him. Oh he wanted to go, but couldn't. For him, the clinical transfer was unsuccessful, but after the clinical transfer, you can actually choose whether or not to go to the middle school. Has he gone?

42:51
At that time, he didn't seem to be there. Yes, you talked to him, but you didn't go to him. You recommended that the transfer was successful. If there was no interaction, I was sorry. Then I didn't go to find him, and then I was on my own. I read it again, and then I think it's still quite attractive to me, is the marginalization of social situations your own choice? My own thoughts, I sometimes feel embarrassed, and sometimes I don’t speak very much. not very.

43:35
Have you thought about changing that feeling? There are also people who want to change. Sometimes when others say that you can’t speak clearly, you still want to change. But I say it's more difficult. And I think it's pretty good now. To say less means that although I don't like socializing very much, I have less student work and so on. Then I think that I can do things that I am more interested in.

44:06 What
do you think you are more interested in at the moment?

44:10
can be achieved. First of all, I like to watch movies or something, but after I went to college, I really watched less, and then I said that the Astronomical Association is like this. You said that you are actually a little involved in fear, but have you ever thought about joining so many student clubs in your freshman year, which actually involves communicating with many people.

44:34
I didn't want to join any of them at first, and then it was because I met a group of seniors on the road, and a group of seniors and sisters were moving equipment, and then I saw them moving astronomical telescopes, and then I was very special. They were interested, and then when I took the initiative to approach them at that time, I said I could help you move, and then I helped them move, moved there, and then they asked me to use an astronomical telescope to look at the big The one, the stars.

45:03
Then I was very happy at that time, and then I became happy when this matter happened, because I always wanted to have this opportunity, but I have never seen the astronomical telescope with my own eyes, and then kissed so I was very excited at the time, and then I went to find them, I said I can help you move like this.

45:23
You actually joined this club because you were interested, yes.

45:27
But it's actually quite accidental. If you didn't see them moving things, you probably wouldn't have joined these methods, yes, because I probably didn't know, because at the time they were just clubs or stalls. , I didn't even look at those times, I said that when I went for a run that night, I saw them moving things to the playground, and I saw it, and then I went to help them move , Have you always wanted to see what or something these moods are about, which makes you have other interests or are you just curious?

45:54 Did
you have this thing since you were a child, or did you want to use it when you saw it at that time? A long time ago, not when I was very young, maybe when I was in middle school and high school, I felt more right and liked these things, and then I saw him holding an astronomical telescope, and then I was also a little curious, because I had never seen Tried it, and then wanted to see what it was like to see it that way.

46:19
So like you usually participate in astronomy clubs, there are quite a lot of activities, not too many. What activities do they have? Sometimes they will organize to watch movies together, and then organize to watch the stars together I also used the astronomical telescope that time, and then I didn't dare. Later, because they sometimes said that they were going to go together, I was a little afraid, because I felt that I was one of them. It seems that they all know each other, and then I don't know very well alone, and I feel a little bit in the past.

46:53 Right
, embarrassing. Do you think you don't know well? They are mobilizing you to have a kind of group of your own, mobilizing yourself to do activities, and then you are basically not very good at going. Yes, but there was a time when there were a lot of people watching a movie, and I would go there again when I didn’t need to sit there quietly with everyone, and you were willing to go when there was no interpersonal communication. If it was 178 people, I would be reluctant to go, but if it was a lot of people, I would be willing to go.

47:26
In fact, will this actually affect your choice of work? You are more reluctant to choose a job of one person, and you are less willing to choose a job that requires too many people to communicate with each other. This is actually compared with your personality. related. understood. Haven't really thought about that, have you? They all say that I am the kind of person who means that if the person is not very familiar with me, my people may think that I am alright, that is, I don't like to talk, but if I am familiar with it, I will be more talkative.

47:58
You said that when you were with your classmates before, the teacher listened to them complaining, what were they complaining about, was it study or? Yes, the main thing is to study. They think that medical students are very hard and tired. That's probably what they are.

48:16
Do you think it brings you too much negative energy? It's okay, I think. It didn't say that it would bring too much negative energy. I think sometimes I feel too tired, but it didn't say it was negative energy. No such. So let me summarize, in fact, you have not yet clearly stated which scientific research direction or subject you like. Like you are now in China, you mainly want to go in, and you can explore your own research by assigning tutors to you. Research interest is such an idea at present. Of course, research is also a very important goal, which can motivate you to further realize your ideals in this area.

49:00
If the transfer fails, have you considered this? Or not thinking about it?

49:06
The transfer failed. In fact, it was because I needed to be in nursing. I just accepted it, or have you ever thought about another plan, which is to be more secure. When you change majors, you can test it, and when you are in middle school. Just rush to rush this kind of thing is that I must not stay in the lake, have you ever thought about it? Nursing basics and prevention have not been focused on.

49:33
Yes, and I think prevention is better because it can transfer 10 people. I think this one is better, do you think it out by yourself or ask the senior sister? He told you what I thought myself. You haven't learned about the situation of changing majors before, I have. I know, but I don't think they can say how to use it as a reference, and we also had 10 people for prevention in the previous year, and 7 of them were nursed by us, so I think the probability of entering is still relatively high. .

50:11
At present, you have discussed with my senior sister about changing majors and jelly, a little more. In fact, I was discussing with my classmates, and they were all liberal arts students, and they couldn't go on talking. I also discussed it with a friend. Is this friend a professional friend in the club?

50:28
Yes, and then he told me that the middle school class is very powerful or something, and the person inside said that if he said let me try it, he said that if he thinks this is still very powerful or something , the middle school class is very good, you might have thought that if you entered the doctoral class, you might be ranked lower in grades. You thought about me, because I don’t think I’m that kind of me, I feel a little bit. I was lazy at the time, and then I thought that I might be ranked lower, but I thought that if I was always on a team with low scores, my score might not be that high, but if One is that the competition is relatively high, and if everyone scores very well, I think I also urge me to do my best and maybe I will work hard, so in fact, you can still accept this.

At 51:20
, the performance ranking may be backward, but it will motivate you to study hard. You can still accept your own ranking, and it may be a little more realistic.

51:33
You said that nursing work may be more difficult. In fact, have you considered it? In fact, scientific research work is actually quite hard. Yes, but these two hardships are not the same hardship. He can control the time by himself. One is spontaneous, and the other feels like forced. He said I can have a son, but I can't force you to stay up all night by yourself. right.

52:14
I would like to ask another point, have you ever thought about it, in fact, in terms of scientific research, your pressure is mental pressure, and nursing him may mean that you have to go to work in the middle of the night. In fact, what he does is simple and repetitive work. He does not have a lot of mental pressure, but what kind of mental pressure does he have?

52:36
First of all, there is a term called a professional term that I have forgotten a bit. Anyway, if the operation of the product, occupational exposure to occupational exposure, and the doctor-patient relationship, I think this is all, it is economical for you. pressure.

52:53
So in fact, you think that doing scientific research, for example, may also face such a situation that the experiment fails, or that the entire project cannot be carried out, and the pressure under such competition, in fact, you feel that compared with nursing, you Or is it more acceptable to accept the pressure in this area?

53:10
Have you considered this aspect of what I just said? Because I just knew a clinical friend, he was writing an article right now, and he couldn't write it no matter what, he felt and then I felt that I saw it, and then I felt that it was really hard, but in fact, this did not make You change your mind, right? I didn't want to talk to him. it is good. Here we are. You wait a moment. how long.
