# Supplementary material for: Why do you choose this program?—A decision-making model of medical students based on grounded theory
Source: PLoS One. 2023 Sep 15;18(9):e0291634. doi: 10.1371/journal.pone.0291634 (PMC10503722; doi:10.1371/journal.pone.0291634)
Supplement: S1 File — (ZIP) [file pone.0291634.s001.zip › RAW DATA/P2 CHINESE.docx]

00:00

已经开始了。同学你好，这个是我们这边是医学教育研究所，然后是想做一个有关于国中单的调研，然后我先给你说一下实验道德的伦理须知。这次访谈我们是采取自愿报名的，原则上的基础上开展的，所以我们希望受访者就是你能够真实的表达自我的想法和认知。其次你要确认自己符合我们的受访条件，就是你的成绩什么的都要符合。

00:37

在我们整个所有的访谈过程中，我们有录音笔会进行录音，录音资料是它的使用形式是科研，就是我们最后要形成一个科研文章要出来，但是呃除了科研这个目的不会泄露给任何的第三方，在访谈的过程中，假如你觉得有任何的问题觉得不舒服，你可以拒绝回答。在访谈结束以后，可能过了一段时间，你觉得呃这一段录音资料，你不想被我们用于科研，你也可以告知我们，让我们不要使用录音资料，这个就是我们的实验道路的须知，你是否知晓并同意？

01:17

好的。接下来我们就直接开始访谈了，现在10:30，这个访谈时间大概是45~910，就是看我们整个过程开展的怎么样。你先简单的介绍一下你自己。好，我是17级基础医学国中班的，然后原来的专业它也是基础医学，基础医学5年制，一期期的基础。

01:44

另外我还漏讲了是一个点，我们这次访谈的目的主要是想要还原就是你从大一开始到报名到比如说宣讲到报名到选拔过程，到这个，最后整个学习的过程的还原，在这个呃在我们访谈的过程中，我们希望你可以讲出，你可以讲你当时发生的事情，你也可以讲你你当时的感受就是你的情绪是怎么样的，你也可以去提你以往的人生经验，或者说认清力，是不是影响了你某某一次，比如说某一个学习的上面的选择都可以讲，我们是一个开放式的问答，我们的问题也很少，主要是呃通过你的对整个过程的一个还原，我们来从中抽取信息和资料是这样一个目的。

02:43

所以呢希望可能在整个过程中我们俩可能讲的会稍微少一点，我们可能会稍微引导的问一问，你在整个过程当中发生的一些事情或者是之类的东西好吧？在访谈的开始的时候，我们想因为我们今天访谈的是关于国中班的，所以我们想访谈的第一个的那个问题，我们想知道你在一大一的时候在报名国中班之前，你对古筝班有哪些的了解，以及是怎么了解的，你可以把整个过程给我们讲述一下。

03:25

因为我们应该是第一届国中版，在我们之前是没有关于国中班这个概念的，但是知道国家重点实验室这个实验室大一的时候，因为我们是基础医学这个专业，所以它本身的话对科研的教学或者说你自己对科研就是要有一些限制，然后当时是报了一个跟学姐组一个大创，然后当时是在遗传实验室，然后遗传或者是跟生日是有一些关系的。

03:55

然后后来有听说我们深圳实验室其他的一些导师或者是方向，然后因为当时大创开展的不是很顺利，然后因为本身是新生，他自己学习的东西也不是很多，然后再加上因为做大创的话，它可能一个团队啊或者什么我们都不是特别的有经验，然后当时想的是如果你想做科研的话，可能是需要一个更好，或者是说能够管理你的一个平台，然后后来有了国中班，你就报我就去报名了。

04:29

所以对于你来说，可能你觉得参加果冻班对你来说，你觉得最重要的一个收获可能是是不是就是说在科研上面会对你有所帮助，这对于你来说是最吸引你的一个点。对，就是因为这一点，所以才会想去抱他的。我比较好奇。你刚刚说你参加大创，你也非常对科研非常感兴趣，你当时选择基础医学专业是你自己选择的吗？还是说你家长选择这个过程是是什么样？

05:04

高中的时候这是我自己选的其实比较奇妙，因为我对临床是有一些恐惧的，就是我家里是独一是很支持的，然后本身有一点晕血，然后再加上因为小时候对生物很感兴趣，所以当时考了医学院，这种时候我是填的是基础医学，包括其他一些志愿，就是其他大学的志愿填的也是基础医学。

05:29

所以我来到这个学校之后才发现原来大家挺基础医学都不是自愿的，我反而是很特别的然后对，所以大一的时候应该是已经很早的很主动的去想去接触一下实验室。你刚说到你有大创的经历，大创是你在大一的时候就报名参加了是吗？嗯对。大创的项目是就是说你自己想好的一个题目，还是说你跟导师讨论了以后，或者说你跟学长学姐讨论以后得来的这么一个项目，就是大创你想选择这个项目的话是先选择实验室，然后我以前感兴趣的方向就是癌症的party治疗，然后所以说看了一下我们目前我们学校有哪些实验室或者什么是研究过方向的，然后看了一下是遗传实验室，他有一个老师是研究肿瘤的，他不是说研究 Party治疗的，他就是说曾经做过一次讲座是关于的，然后我就可能自己就以为他们实验室会做这个方向的，然后后来和学姐一起认识了之后，就在实验室组建了大肠，对，因为他们实验室是做胰腺癌的。

06:44

李刚说了一个非常专业的名词，就是癌症的颗粒治疗，我想知道你选择这个方向的时候，有没有跟你家里人或者说跟你的同学讨论过这些东西？其实是有的，因为其实开始招人很早的，当时在高中的时候就有，然后我们高中当时生物老师是因为我高中时候参加生物竞赛，然后老师跟我说就说 car t应该是很有效，但目前进展不是很好的一个。

07:10

然后后来家里是因为也有长辈生病，靶向治疗的话，癌症的话培养治疗是很热门，但是他问题是效果并不是很好我看了一下，毕竟就是这些长辈他们是因为这个也没有得到特别有效的治疗，所以说既然后来又选择了这个专业，就想自己去看一看目前我们国内它进展到底是怎么样子，以及自己能不能做些什么事情，所以你就选择了大创的方向，然后再你和学长学姐组了一个大创的组，在实验室里的学长学姐，在实验室的学长学姐啊，然后他们就建议你去多了解一下实验室嗯。

07:59

对在跟他们在实验室里。因为后来他们说因为实验室并不做那个方向的，然后他说肿瘤治疗的话，其实归根到最后都会是靶向治疗，但是你要了解很多的机制，然后你就会觉得自己以前只懂了一个点，然后你也是听他们建议去深入了解，然后大家就开始做大创，当时报名的时候你有参加他们的宣讲会吗？国政的对参加了的。你有没有通过其他的渠渠道或者说有去再多的了解，还是说你的所有的对于班级的信息来源就来源于那一次的宣讲会？

08:43

你有跟辅导员或者说学长学姐或者说你周围同学或者家里人有讨论过这个事情吗？其实都有就都有，对。我想听听你跟家里人讨论的过程是怎么样的，他们怎么想，他们对这个班级怎么想？因为我父母他家里的比较年纪比我大的一些哥哥姐姐就知道我读了专业之后，建议家里面就是说可以考虑以后出国读做科研方向，所以他们觉得当时报这个班，他们就想的是会不会影响，也会会有限制，可能你要一直读下去，然后不能出国或者什么的。

09:25

然后后来在宣讲会上的时候有说就是说可能会有联合培养 CC或者是其他的方式，然后后来我跟我爸妈就讲，我爸说那也行，可能对于你你的父母来说，他们比较关心你以后未来发展可能会跟出国这些东西会不会影响对就，是因为是哥哥姐都这么建议的。

09:51

你自己接受他们的建议吗当时？当时的确有接受因为，对，因为像我大一后不是有一个什么访学计划，访学出国访学的一个项目，自己也是报名参加了的。访学计划是你基础医学里面还是全校的？全校的是吧？访学计划是跟你专业相关的吗？当时访学去的是威斯康，星威斯康星它是威斯康美国的威斯康星大学，它是做干细胞治疗的，是比较出名的一个，所以当时我也就想去看一下。

10:27

所以这是对于当时来说，你对火中班的所有了解就是来源于那一次的宣讲会，毕竟也是第一届对吧？也没有其他的渠道，然后和呃主要是和家里人讨论一下。有跟辅导员或者同学讨论吗？他们有没有听过他们的想法是什么样的？有。辅导员其实是当时去问了，他当时也是觉得说这个班很好，然后班里的同学当时是在转专业和考活动，同学们都在忙这两件事情，然后当时其实我这两个也都报了的，然后后来的话就是转专业，你刚刚说你报了转专业，对。

11:10

转专业当时你报的是哪里？我报的是口腔。口腔是吧？是他这也是父母推荐的，一个是太阳还是有很多担忧的，就担心就是说可能你做基础的以后要走很长很长的路，或者是担心有的工作，所以他们就建议说有转专业的机会去考一个口腔试试看，走很长的路是不是指的？

11:38

可能以后走科研这条路可能读书的时间比较长，他们的意思是这个意思，对，他们一般就觉得无论是在国内还是出国读，你都要走了很久，就是说你读到可能以后一直往下读，说不定你在国内读到博士之后，你还要出国做博士后，这种他就觉得这是一个非常长。

12:01

但是你刚刚说就是说你自己是对科研这些比较感兴趣的，你父母我觉得你也是比较愿意和你的父母去做交流的一个同学啊。

12:13

你的父母在提到以后，比如说学技术医学，可能可能要花费个人比较长的时间，走路比较长，你知道心里是怎么想的，但是我觉得自己的时间花费还可以，其实我担心的更多的是觉得你听了父母对你的这些建议以后，你对科研的态度是什么想法，或者你的情绪是怎么样的。

12:37

怎么说读专业之前他也自己有一个个有一点点了解，然后父母说或者是朋友，就因为我有还有读其他专业或者不在本校的朋友，他们也跟我说，但是怎么说，我们都觉得读书可能比工作或者什么的要嗯更像一个乌托邦或者说是金字塔那种，所以我父母也我觉得读书可能还好，但是后来就发生了变化，等到了大三的时候就开始有一种感觉，就好像是有一种你一直在围城里的感觉就是做科研就是什么的那种，好像看不到就是一个头的感觉，这种看不到头的感觉说或者说在围城里的这种感觉是某一件事情影响到你，是突然让你有这种感觉，还是说是慢慢的一个过程？

13:45

嗯我觉得是慢慢积累的，他跟你想象的不一样，就跟你一开始自己对科研或者是说你想象的理想有一点开始慢慢出现偏移了，当然这种偏移，但是你越慢慢积累下来，感觉这种偏移就越来越大，就脱离你自己一开始所构想的东西了。

14:08

你有没有想过当时现在所做了这么多时间做那么多科研，和你当时高中时候理想的偏移，你有没有总结过它来源于哪里呢，或者说会不会有可能是比如说我随便说了，有没有可能是实验做的失败的次数太多了，或者说他没有达到你当时刚刚想的是做科研，改变人类社会这种愿景可能觉得太难了。

14:40

是难度太高，还是说生活中就是科研中遇到的挫折太多，导致你有没有总结过？

14:46

我总结过，但是您说的这些它其实也真正的发生了，但是它却不是让我就觉得很难以接受或者是难过的事情。

14:59

我反而是觉得让我慢慢开始对科研觉得很无奈的一种情况，说发现是一个就是自己他知识断层的，它是一个你也不是知识，是你了解段子，你做这件事情和你想这件事情是完全不一样的两个概念。

15:22

你原来可能是以为是自己一个人在做，当你真正去干一件事情的时候，它是有一个周围的一个环境的，所以你当时从来没有设想过那样子的环境，就等你真正进去了之后，你发现你的环境是一个封闭的实验室，是一个封闭的细胞方，或者是一个封闭的实验台的时候，他这些具体化的想象，具体的实际跟你当时对于科研的想象是完全不一样的。

15:51

还有一点的话就是说人没办法永远活在象牙塔里面或者是乌托邦里面，你必须是跟生活实际是有很多接触的。你以前想可能或者是跟朋友就天真的想就这样一直读下去也没有关系，但是其实你长大之后就会发现自己慢慢在承担很多的责任。就有就是当然这是我个人的感觉到了周围身边亲人的他生老病死就会可能自己是因为还比较感性的人，你会发现有些无能为力，就有的时候我也会责怪自己为什么去不去选临床，尤其是在医学院面临这种事情的时候就会很很自责，因为当时的分数不是不可以的，就有一段时间就会很焦虑，就很责怪自己，就如果能后悔的话，自己一定不会选这条路。

16:48

有一段时间就是沉浸在这样的一个情绪里，我从这里面补到两个星期，我有你第一个信息我想问一下，你说的第一点是当时觉得自己的，知识断层可能就是没有办法做到自己从当初幻想的科研环境，我想问一问你还记得当时高中对于科研的幻想是什么？是科研是哪一个点或者哪一些方面是吸引到你，让你当时高中的你想要去做做这个事情。

17:20

因为从小其实对科学新闻社可能是爸爸的一个诱导，就是我们他会带我看很多的科教片，然后包括一些很有趣的动物的纪录片，然后当时你对科研的想法是很天真，是觉得你自己是一个野外记录，然后包括就是观察，然后相当于这种动物动物行为学的一个方面的。

17:47

然后后来高中接触了医学，然后像刚刚提到的car t治疗，然后你觉得是临床上面的一个应用转化或者是这种但是后来你到了大学之后，你接触到的科研，你就会发现它是你必须就相当于把你关在一个屋子里面去做事情。

18:11

一步一步的就脱离了你原来的想象，是不是会觉得有点枯燥无聊？

18:21

也没有你其实也没有也没有，只是觉得自己的知识还没有支撑，没有办法支撑自己做完科研的这些一些步骤也没有。

18:33

他说的我刚刚其实说知识断层不太对，信息面的一个断层，他真实的世界跟你了解的跟你自己想象的是完全不一样，和小时候看的那些记录，对，包括你高中或者是什么，你参观的无论是科技馆还是什么，它都是不一样的，对。

18:53

科研就是整个过程和它和你了解你其实高中当时可能了解的是一些科研的成果，结果一些东西我觉得很有意思，但是你在实际去探索的时候，发现很难去探索一起就立马去探索到一些有效的成果。

19:14

对，后来不开心了，你一开始就可以很开心，当你接触这个课题的时候是开心的，你了解这个课题的时候，好像开始变得沉重，你要想很多的事情，然后你再慢慢去做的时候，又是不一样的感觉了。

19:37

做做的时候和了解的时候，是两种感觉，你刚说的第二点，家里可能发生了一些事情，让你感觉到焦虑，就是你可能感觉到自己是帮不上忙，所以才有一点点是后悔，报了技术医学是吧？

20:01

对。

20:06

家里这个事情方面讲，不方便讲就跳过。

20:10

可以跳过的。

20:14

你刚刚我们问了就是你和家里人讨论国中班，还有辅导员讨论国中班，你有没有和你同学讨论过当时报名的那会儿，因为和同学讨论过这个事情，你有没有和他们交流过你们之间的想法？

20:31

有，你是说指同班的同学，还是说都可以宿舍的或者是同班的，或者说路上走路碰到的之类的，都可以让你印象比较深的事情，或者说影响到你的事情之类的。

20:45

因为我有朋友是接触社会医学的，然后他跟我这个朋友是你的同学吗？还是你学校别的学校就一直对。

20:54

所以说他当时我跟他其实是在高中之前，我们就可以一起做一些比较有意思的一些嗯，不能算是社会调查，一些话题的探讨，然后当然我一直是很偏理科的那种，所以他当时我跟他说，我可能可以从肿瘤转去做生殖方面的科研的时候，他其实是说就是说很有意思，因为我们当时都在讨论很多关于同性，然后性教育，然后艾滋这些很多的一些话题，因为我们去关注了很多一些社会上很当然说lgbt这种群体当然是他研究的他关注的一些方向，我们都一直在探讨，所以说当生殖医学就是出现在我跟他探讨的时候，我们是其实很兴奋的，因为我们当时知道关注一些报道，就是关于很前沿的一些了，就是说同性生子这样的一个环节，就是说你可以诱导一个精子它去变成卵子或者说一个卵子它就变成一个精子，这样子就是同性它也可以孕育后代这样子的一个东西。

22:05

所以说这个话题是很让人兴奋的，这也是为什么我说你一开始了解生殖医学是让你非常开心的一件事情，因为它的确就跟你之前接触的一些兴趣点，他相撞就会让你很开心，你好像就觉得自己进入科研之后，以后就是可以往这个方向去做的，所以这个是他很鼓励我去读，所以和同学的讨论更加激发了你在和同学同学讨论之前，你是说自己本身就很想参加，还是说自己还不太确定想跟他在所以这也是想参加，我在想参加的原因是因为我自己觉得大一的时候自己科研能力不行，就觉得自己关于实验或者是什么能力不够嗯，然后和同学讨论以后。

23:00

对，就会发现更有兴趣的地方，感觉就好像在鼓励你一个问题。

23:09

你都可以问到，我觉得是我一个人在那问，我们这是一个聊天的过程，你现在是17级对吧？

23:23

所以是五中班已经上了有三年了，大4我在之前的国中班的宣讲的PPT里面，有看到过一些他的对他一个改革教改模式一个特色的介绍，里面有一个有几点我跟你说一下，一个是奖金科研导师制，还有一个特色的建实习，免疫优先录取，还有5+1+3的一个博士的模式，还有一个出国深造，这里面你觉得哪个是当时最吸引你的一个点？

23:58

我觉得是科研导师制。

24:00

嗯对当时。

24:02

科研导师制的话，你们现在是大四对吧？

24:07

大四的时候应该已经选了导师了。

24:10

选的是大三的时候选的研究方向是，按计划来说的话，呃对，但是其实大二的时候，我大二大三就已经在负责一个课题了。

24:20

嗯大二大三当时选这个课题的时候，是你自己选的吗？

24:27

你？

24:28

刚刚说的你和社会医学的同学讨论了一个关于精子转转换成卵子，这种这种对它是一个遗传应急消除，然后再转换细胞的一个方法。当然了我看到我们实验室是有做的，我以为进去了之后可以选这个方向进行科研，但是其实没有。所以我大二大三做的课题不是我自己选的，是导师提供的，所以当时没有选上这个原因的能说吗，他不是没有选上，我去的时候没有人在做这个了，然后实验室又有其他的课题，他没有人来做。

25:09

所以说导师是说既然你想做一个课题的话，就是说你从中选一个就好了，当时你比如说我只是提了一个假设，当时你本来是想选这个方向，就会发现这个班级没有导师在做这个方向，你有想过要比如说退出吗之类的？

25:33

当时没有想退出，因为当时两两因为是两个原因促使我来这个过程的，有一个感觉自己科研的确不行，所以说他必须自己要提升，所以说当然是要留下来，毕竟大二我也没有学什么，就跟要学学他，说看看自己以后，或许就是装备好这些能力之后，才能去做自己感兴趣的东西。虽然课题啊和自己当时题目和自己想的不一样，但是还是可以训练一下自己的科研能力。

26:09

所以这一点还是让你坚持下来去继续学习，还有吗？

26:14

两你当时说有两个原因，嗯就是，一个是科研能力，还有一个就是自己幸福。我是想是自己以后对就想自己以后可以做。有了这个能力以后，我就可以自己去主动的去开展，我感兴趣，对。

26:31

你现在对这个还感兴趣吗？

26:33

我现在已经换了导师换了课题了，因为在这两年的过程当中就慢慢的发现，可能自己想的太天真了，他你做课题并不是仅局限于自己的兴趣的，就是还有很多你的资源，包括你导师，包括实验室的它整体的方向，然后甚至还有一个整个的一个实验室环境，然后你能学会的就是时间进来，甚至包括一个你实验动物的选择，它都是很困难的一件事情，就像是如果我们学校你想用猕猴做实验的话，其实是很难的事情，所以说那么他科研做科研做到你想自己随心所欲的时候，那是一个非常非常难的地步。你在这个过程当中你说的的确也是事实，科研很多时候选择并不是基于自己的兴趣选择，你在了解到慢慢的经过很多事情，比如说弥合的这些事情，还有整个课题选题的这些事情以后，你当时有什么想法吗，是不是觉得会不会有一种挫败感，有没有？

27:49

有这就是我前面感觉自己信息断层了，真实的不是你就是那样美好的想法，可能你自己就觉得科研就是想做自己感兴趣的事情，去研究一些人类未知的事情，但是你现在直接操作的时候，发现很多很多环节都会受到不同程度的阻拦，或者说就是说没有办法去在你的想法之内进行操控，比如说人力或者资源的调用这些嗯对，然后就会有一种有一种可能叫挫败感较合适这种感觉。

28:31

落差对和自己当时高中时候想的那种感觉不太一样。对。落差太大。了嗯。当你一旦知道没有办法去弥补这个落差和什么的时候，你就会发现这太太让我直丧了。

28:54

你可以谈一谈某一件事，某一件事情，你在比如说你想做的一个科研课题，但是发现资源并不是你想用就能用的，比如说猕猴的事情，我想听一听你有的事情，因为这事情其实是一个他不是主要的事情，他只是我用来举例的，就是当时他我当时去美国，然后后来导师问我美国的实验室和中国有什么不一样，然后因为不是那个实验室是挺出名的，他都作为了一个可以参观的地方了。

29:30

所以当时我们是看到了像一个动物园非常多的小猕猴，还有其他种类的什么实现猴一些常用的实验。但是我们实验室我们有动物中心，其实我们动物中心是不饲养猴子的，然后他问我什么，我当时只提到了我说这个给我带来的影响还蛮大的，我以为其实国内外做科研其实是差距没有那么大了，然后我导师说是的确存在这样的一个差距。

30:06

然后当时他跟我说，我们现在也没有要需要做到猴子的这样子的一个实验或者是课题，然后我说也是但是后来你自己一想嗯，如果就是说哪怕你现在到了我们学校这样一个做已经成为了一个很优秀的教授或者是什么，当你想就是说可能我的实验或者课题需要使用猴子或者什么，其实你是没有办法用到的。

30:35

你之前说就有一段时间你非常后悔，继续作为我们班大概持续了多长时间，现在还有这种感觉吗，就大概持续了三四个月或者更长一点，我自己没有感觉。是大三的时候。

30:58

你后来是怎么解决这种负面情绪？

31:05

我不知道就继续读书继续搞，就感觉自我调节我一直自我调节能力还蛮强的。

31:13

因为出现这种情绪是因为家里出现一些变故，感觉无能为力，为什么不学临床这种这种后悔，就是人一旦陷入这个后悔，他就反反复复后悔，然后因为家里会不断的跟家里可能跟你的爸妈去因为他们也指责我为什么不选择临床，所以我就会因为这些情况就会更后悔，他就会陷入了一个圈子，当时一度想退学去，要跟爸妈说我重新高考好了，没有开到跟你说的继续活动呢，有什么好有给你提供这种想法的？

31:59

是朋友或者亲人。

32:01

没有，所以整个过程就是你自我消化，对，所以其实也没有，我觉得应该也不会跟同学说说这个事情，有跟朋友说，然后我朋友说如果你真的想退学的话，我也支持你。

32:17

然后到了那个地步，然后其实就花了很长一段时间才平静下来，平静下来之后就觉得自己还是要做一些让自己感受到被肯定或是有价值的一些东西，就开始产生想要跟他们证明我们选择什么样的错误什么的，也没有产生这样的一个改变，当时是不是当时想了第一个想法是我要换一个环境，然后不过当时已经错过国重，退出国重的这样的一个。

32:55

你们不能随时退出吗？他是要在学习初的时候，对，要不然教务那边很难对接。对每一年的对。然后然后当时最想的是要换一个环境，所以后来的话嗯后来又遇上了疫情，对我本来就很焦虑，有一点抑郁，然后在疫情的那种阶段的时候就更更难受了。因为我好之前就很朋友很喜欢探讨社会话题，所以说当武汉分成的一段，时间也是我很难熬的一段时间。

33:31

然后后来当然也不知道自己后来又返校了，然后当时开始接触我们学校，开始做一个叫疫情的应急管理系统，然后我是想瞬间改变一下环境，所以后来去参加了，这是一个大创项目吗？还是什么东西，这是一个大创项目。你是怎么知道这个东西的？是因为国中班的同学对在做，他邀请你加入他的组。

34:02

对他跟我说了之后，因为我当时是说想自己换一个环境，我就问一下就是说你这个是不是比较偏社科一类的，然后就觉得然后又在疫情期间，当时自己的想法就是说又想换环境，又想自己想自己被自己肯定就没有那么糟糕其实。所以说当时就是换了导师和项目，做的你的导师也换了。对。在这个时候和他换了一个统一的导师是这样子吗？就换了差不多是同一个方向的，但是跟他导师不一样，但是这几个导师都在做这个事情，你不懂事还可以，就这样换了，你觉得老师没有想法。

34:47

有，因为其实这个时候因为之前在大二大三的时候，其实跟导师交流很多，我们就是因为我当时带了很大的热情来到这个实验室，所以说做课题的时候也一直跟他交流然后什么的，所以后来出现了一些包括自己认知的一些落差，然后后面带再带自己心里的一个变化，你都跟导师交流这些东西。

35:14

没有跟他太交流，但是他有时候会问我，然后我有提出来，然后因为我当时一开始去联系这个导师，因为很优秀，他就是我说的他之前做过一个精子的印记消除的，他是做过的，所以说当时我也是因为这个社会话题去问他的，然后后来他问我，就是说感觉最近的状态，或者是做实验，他问我进度满不满意或者是什么的。

35:46

然后后来在这种交流过程当中，我提出来一点就是感觉自己很迷惑，或者是什么，然后他说然后就告诉我我还小，还有很多的选择就是路还很长啊，所以你换导师这个事情是跟对，是跟原来的导师探讨过的，但是我没有跟他说。

36:10

对我没有跟他说换去哪里也没有跟他说，只是我当时跟他说，嗯就是自己感觉是需要更换的，因为是感觉自己原来带着极大的热情和兴趣来到这个实验室的，但是不知道这两年是因为外界或者是因为自己他的兴趣就开始慢慢消失了，你跟第一个导师讨论研究方向，感觉你们交流了很多，当他跟你提出来，可能你没有办法做到当时高中时候或者说跟社会学的同学探讨这个方向的时候，你心里的感觉你还记得吗？

36:44

印象里会很深吗？没有很熟，因为当时我迅速的接受了，因为我想的是我来的是来提升自己的科研能力的。好的，然后换了导师换了环境之后，还有去了新的项目之后，就是你情绪就转换过来了。对，因为这个事情发生的很后悔，那些情绪就带走了。我的确是这样子，他又慢慢就变好了。

37:15

所以其实你是换到了一个比较感兴趣的项目去，对的就是说可能自己又产生了新的兴趣点，就是觉得兴趣点能让自己更肯定自己。

37:28

在这里面你主要考虑的就是自己的兴趣，而不是说就朋友父母的建议或者就业能力。这么想好像是的，要感觉做让自己开心满足的事，如果做不到的话，自己就会陷入很长久的怀疑。牙一种好。我们想听一听你从读国中班以就是这么应该叫两两有三年了嗯，对。大二大三大四。这三年来你觉得印象最深的一件就一件一件事情，或者说最大的一个感受是什么？最快乐的事情或者说最不满的事情，都可以说印象最深的一件事情。

38:22

影响最深的一件事情就是有一天那天应该是情绪不好吧，可能是跟妈妈交流之后，然后家里的事情不好，然后宿舍我又觉得很烦，跟室友说了一下，室友就安慰了我，但是我不想听，因为我觉得我在听我会哭，然后我就了出去的时候，我就去了学海楼就是科研楼，然后去学校了去了我们实验室，然后走了一个过道的时候，然后已经憋不住了，就太难受了。

38:53

我就当时我就觉得自己一定下一秒就会落泪，然后我的师兄跟我说，你的课题汇报准备好了吗？他就拦住了我，然后当时我一个就硬生生的，他所有的情绪都被憋回去的，感觉那一个瞬间我是记忆最深刻的。企业的工作对我但也当时我写的是我写日记，我说我真的成年人的崩溃，就是我当时感觉第一次成人的世界就不需要眼泪。

39:26

你那么当时是在过道里是吗很黑，没有那么明白他，没有感受到你很难过，没有，因为我走得很快，因为我其实不想自己哭，我想的我本来想的是回实验室，可能看看文献看什么应该自己能平静下来，但是他那么觉得问了我一句说你的师兄是知道你有这个情绪，还是就是非常直白的方式安慰他不知道。

39:55

他真实的问题，对，他就真实的问你课题汇报主要是内部会就有那种聚餐或者之类的交流，就会有关系还是？关系还是可以的。

40:07

对，但是这种事也不太好，不太想说，所以他也不太了解。所以这种其实是比较私密的事情，可能也不会跟实实验室还没有有那种要好到跟因为他们毕竟是师兄师姐，就是你要尊敬当时你说要回实验室，看看文献一，当时是晚上是吧？不是，就下午我要办完快快到晚上了。其实当时你主要是想一个人待一待，不想和别人有交流，就想自己内部消化一下，对。

40:47

这个有当时影响到你对会不会觉得是不是什么做实验的，都是这个环境怎么这么不近人情，怎么这么实在，其实没想那么多，但是自己想的当时是什么样，当时就真的觉得是有一点太难受了，太不会赢，你之前有讲过人因为你说人不可能永远待在象牙塔里面，还是要和人和人有什么互动之类的，做科研要沉下心来静下心来一个人去做这个事情。这个事情是不是会不会对你有这种改变的是一个诱发的一个人？导向，因为你讲这个事情突然让我想到你之前讲了一个我觉得他们是有联系的，你自己在成长，你就开始慢慢发现，你不能天真的想你自己想的事情，你必须要跟很多东西都要有沟通。

41:52

嗯就是你这个时候开始认真的去想父母的说的工作，然后或者说以后的他们是工作是什么样子？他们在问就是说你的就业或者是说你打算是什么，还要不要出国？然后或者是说你，如果出国的话花费会很多。当时真的会有考虑一些，以前是那种想的，可能是热情能当兴趣能当饭饭吃，但是后来你就开始慢慢发现不是的，你真的要考虑很多。

42:25

尤其是当一切都还好的时候，你自己也不用考虑那么多，但是一旦有一个平衡点你消失了，比如可能家庭或者是亲人有一些事情发生了平衡点就没有了，你那个时候就开始考虑就业怎么样，你如果出国读书花销又会怎样，然后你能不能真的为为、家庭或者是说家人做些什么，这个时候会真实的考虑，感觉自己是有一点长大了。

42:59

你现在还会考虑出国吗？现在也会考，现在是想的是两布线同时走，也不是同时作为现在做的方向的话，其实因为是自己是走出来的，就不会陷入之前那种否定怀疑，然后再后悔那样一个恶性循环里面，当你慢慢走出来的时候，你再看其实在做这个事情还是只要是一直往下去做，无论是工作还是什么的，你是可以慢慢得到的，不要那么的就是不相信自己。

43:39

现在有这么想，就是说如果到了那个时候读书读到的，需要你出国或者是什么时候是可以考虑的。

43:48

因为后来也跟父母讲，他们其实后来我妈妈说要不要去买抑郁抗抑郁症的药，因为当时已经那个样子了，后头还跟我妈妈说，我有一个点也买了，也蛮难过的，就是我那个时候是在陪在医院的顶楼吗？当时我很大的一个想法就是我可不可以从这里跳下去，然后就很难过。

44:16

那个时候后来我回了家跟我妈妈说，我说妈妈我以前没有这样的想法，他为什么整个访谈会把我整？然后说了我们还有一个然后我一直很久没有说没有对别人说这种话，就我跟妈妈说过什么。

44:34

你妈妈当时有很自责吗？

44:38

那我，我妈妈说因为她也那段时间也好，也很忙，他自己也不过过不了自己的情绪里，对，然后也没有对，我说我们那时间太难过了，我就说如果可以的话，妈妈我昨天我想让自己不会有那么多事情缠绕着，然后当时后来我妈妈也说了一句，我可能那时候我后来我觉得让我很开始慢慢我其实可能一开始都没有想我要走出去，正好有一个想法产生，因为已经困在那个里了，后来我妈妈跟我说，就是说他跟我说就是说人生每个时候都有很多的阶段，他说这个时候很多事情都是你不需要想的这是妈妈来想的事情。

45:31

那个时候我妈说的那样子的话之后，我就开始他就感觉是困境就撬开了一个口子，我就想自己可能想的不对，也想的太多了，就一直在绕。当那个时候停下来就行了，停下来就不要想了很多，停下来就可以让自己看看能不能走出去，所以说那也是一个改变。

45:57

你的妈妈一般就是在或者说你的父母对你的就是这些选择，我感觉应该一一般都是比较支持的。对吧你当时想报基础医学，没有报临床医学，你的父母应该也是同意的，对吧？他们还是比较愿意让你追随自己的理想的。对，他们是不同也没有办法，因为我可能会所以其实他们还是内心里还是有点不愿意，但是说既然你要选择，那你就学。

46:29

对，因为我感觉自己从小特别的我妈说特别的有错误的途径，这种感觉，但是我妈会反反复复跟我说，就形成了一种我自己怕自己，就你我妈说你可以选，但你一定要负责你，一定要对自己负责。

46:47

所以时候我就一直很后悔，我就感觉好像自己负不了责，对自己负不了责，所以这种情绪可能也来源于你你你，妈妈就是一种传递，就是你的，理念其实没有错，就是自己选择自己会自己的选择去承担这个后果。现在应该不是每个人都会像我这样的这样有这样子的一个英语的情况，我觉得应该是对，我应该是个例。

47:24

以后在你们现在也大四了，马上就五大五，你有讲过研究生这方面是怎么考虑的吗？是要继续学校还是出国？因为我现在其实基本上就已经更换了方向，以前可能是纯升值的。对。对，如果读纯生殖的话就应该是留在本校，然后读因为现在做的是疫情的一个应急管理体系，它已经比较偏社科和卫管方向了，所以我是在考虑我们考虑以后读研还是读应急体系，对，会考虑未管的方向。

48:06

所以说到底是继续基础医学还是管理学，比自己现在也还没不是很清楚，还想继续看一看。其实感觉自己心里已经有一个很偏向的了，你觉得你现在对这个体系现在这个研究项目还是很感兴趣，感觉感受怎么样？

48:30

感触就是他让我觉得我自己新的兴趣点，我觉得他从某一种程度上他在帮助我，让我觉得自己不那么不那么糟糕，是因为我们这个项目它是需要很多的访谈的某一个发现，还是说某一件事情让你觉得你参加了这个项目有个成就感，或者说让你就是说也是我觉得肯定是有一件事情或者有一个感受在那，才会让你觉得你让你觉得不会沉浸在自我否定的循环里面。

49:10

有，对，因为我刚刚其实就想讲，因为我们这个项目它要做，其实还需要很多的访谈，就像我们现在所以我就为什么做访谈，会把我把我给弄哭了我就感觉我也做过很多的访谈，我们有一个访谈，他是要去武汉的，关于这个的时候，然后那天其实我其实是非常晕车的一个人，然后呃在疫情期间，我当时看了一个别人拍的片子，就是说我的武汉病了，他现在一辆车都没有在路上，然后那天我也是坐在武汉的一个出租车上，武汉都是车水马路，他那条路上都在堵车，然后车非常多，那天我头一次感觉自己去年下半年还是对去年下半年，对那次我头一次感觉到我自己不晕车了，我自己内心非常的开心，是真实的开心，感觉就是武汉他这场病它就又好了，你因为课题它其实说是它是卫生事业管理，它也偏社会医学，它跟医学有关系，因为毕竟是公卫的一个人群的一个东西，疫情管理这方面，所以当时我真的有一种虽然他不是我让他变好的，但是我在经历这样一个变好的一个感觉，他给了我很大的一个安慰和信心，就是你在经历一个美好的事情，因为他让我会想到以前这边会发生了其实非常不美好的事情，但是无论是时间还是观念的讲，就是我们这种疫情已经就卫生的一个应急体系它建立的他让这一切都变好了，他会让我实际的感受到，我想了解一下你现在在做的项目，因为你只是讲了一个题目，应急管理体系，为什么会涉及到访谈你们主要的访谈的大概的一个访谈的话是你要关注很多东西，你访谈对象，然后问题就像我们现在这样的，你的目的是什么？

51:19

他不同种类，其实我们当时最主要的就是说，因为疫情期间我们也是比较一个最初的出发，在疫情之前我们其实有很多的恐慌的，然后也有很多的一些可能不太对的漏洞出现，中国有很多包括当时的口罩或者是其他的一些物资，很多你这些事情不是新闻是你正式发生的。

51:42

所以从这些漏洞出发，你其实是想做一个很全面的访谈，就是真实的去揭示一下，就是说你认为在疫情期间初期，最基本的是比如说社区的那些你采访社区的人，他就问的是说你在一层初期你遇到了一些困难还有问题，然后再往上你可能是一些市级的疾控，他问的可能是一些疾控或者是医院的领导，然后他从他们脚面角度，因为他们是真正的前线，他们面对患者或者是说一个疫情防控的时候，他们真正出现的问题还有可以改进，或者说他希望得到一个改变是什么？

52:25

这是一个访谈，当然访谈都是我们的初期工作，你后面还有一些因为我分为很多板块，后面你会负责吗？还是说也会，但是一直跟进的？初期我们是想积累很多的材料，为你后面的数据分析，包括其他的一些东西建立提供一些数据。

52:45

然后目前的话，你们现在应该资料收集已经结束了，还是在这个过程中？资料设计已经结束了，因为现在它已经到了一个省赛的一个阶段了，所以说对一个这是个大创项目。

53:01

对他是一个大型项目，然后他参加的是一个挑战杯的比赛，还有第一次你们约我那时候正好在弄挑战杯的材料，然后没有来参加。这样的。明白。我想知道一下，你们你这个其实是相当于参加了一个大创的项目那。大创的项目的导师和就是你你们自己研究方向，因为我当时看国中班的宣讲PPT，我的理解是你们在上课之余还要再选导师，然后在业余时间跟着导师去做这些研究，所以这些都是可以重合的。

53:45

对对对其实还是蛮蛮可能还算一个蛮便利的事情，因为可能不参加国中班的话，他们要参加大中的话，其实也是用自己的课余时间来做这些。

53:59

对你如果不参加过程中的话，我如果不参加过程中，我不清楚我会不会经历自己大三的一个一个一段就是不太好的就是时期，但是你经历，但是你参加了国寿你也经历了这个时期，但是你也有契机可以去认识很多的，比如像现在大创像现在这个项目其实算是一个蛮典型的人文社科类的项目，应该和你大二接触的升职的就是纯升职的科研方法应该是完全不一样的，对。

54:29

不一样的嗯。有感觉到困难吗？

54:32

接触人文社科的这种研究方法的时候，比如说从访谈的，比如说你在访谈的时候，你们应该是做半结构化的反弹吧，还是做完全的有那种就是问答式的方法。半结构的，对。但是这些困难我觉得还好，因为它相对于实验室的歌封闭的一个环境，它是很开放的，所以这个也就是和你之前讲的也相互印证了。因为人文社科的研究是人的研究，人和人文研究，它并不是像理工科类那种研究，是自己窝在实验室里去做实验，需要静下心来你一个人去做，然后去分析我们这种可能需要人和人之间互动来采集信息，采集信息的途径不一样，所以可能会更会不会是因为更贴近你原本的一个想法，还是说因为你这个想法促使你去转投到人文社科方向的项目里面来的？

55:40

都有我觉得相互影响，所以说你接触了之后，他更能让你感到舒适就是一个很契合你的一个兴趣点。

55:53

我想问一问你的个人性格，你觉得你是一个外向还是内向的人，你喜欢和人打交道，感觉自己这一点好像挺外向的，但他分很多时期，我感觉如果我自己不是不是在一个心情很平和的时期，其实自己一般都是封闭起来的，会自己调节自己的心情，不会说想要因为有的人像遇到的是比较烦心的，或者说伤心的事情，喜欢跟人聊天，然后来来排解苦闷，我感觉你应该不会，你就是自己自我消化。

56:35

一个愿景，对。对是。国中班来最大的感受，我印象比较深的就是科研方向的一个转变，从一个自然科学转变到一个人文社科的转变，对于未来的规划，其实你现在还是比较偏向于，人文社科的嗯那会不会感觉自己5年有点浪费。

57:08

有这种感觉吗？有点可惜，没有最近在准备。

57:14

一些材料的时候，就是会看到自己历年来不是是保研的材料，对，因为他不是还提供一个保研的都一个便利，所以自己在准备保研的材料的时候，然后看到这一路过来还是挺开心的，但明显我觉得自己大大三是自己收获最少的时候。大三这也是一个转折期，对，应该相差也可以这么说，花了很多的时间去自我调节自己的心情，自我排解那些负面情绪，花了很多时间。

57:56

保研。

57:57

保研的话，其实你假如拿到了保研这个资格，你你会考虑人文社科的方向，相当于最后还是说没想好，想好了就会去我我现在已经在对。

58:13

这边好像是国中，那边他老师也收集过这样子的。

58:20

收集过就是问大家的意向，你可能是留在国中，还是说去读其他的学校其他专业？不是其他专业会对你们有一些负面影响吗？

58:31

在这个时候，比如说你说我不想再继续再补充读下去了，我觉得一定不能是到了最后这个时候，你可能对他如果到了大五的时候，你对说语言专业好像不喜欢要选择的话其实还挺难的。如果你可能大三大四就对新的方向或者是有了一些想法的话，是可以考虑试一试的，我觉得应该不会有特别大的阻碍。

59:04

我还有一个想问的问题，就是我看到他的宣讲PPT他的它的专业基础是基础医学或预防医学，然后它增开了生殖生物学、发育生物学、体验论坛、科研实践，这些课程应该是基础医学没有的课程，对吧？你对这些课程你有什么看法或者感受？

59:26

这些其实是一个有挺有用的一些设计，对你科研学习是很有用的，尤其是大二的时候，你在做课题的时候，毕竟我像我今天提到你自己的科研能力是不是很充足的，所以你是需要希望自己能够你自己学的时候，他是没有系统，甚至说你可能资质或者是一些选择的科目或者是知识是不对的。

59:53

但如果他给你提供这种课程学习的话，他是非常给了你一个系统学习快速提升的一个机会嗯。所以它其实是满足了你的需求，这些课程，因为你本来来就是想想想提升一下自己的科研能力，这些也能提升点自己的，科研能力。

01:00:12

你觉得假如你没有报国中班，你自己去上基础医学的那些课，你觉得可以通过那些课里面提高自己的科研能力或者科研方法，他没有这个来的直接，他这个的话是我觉得要比原始的要更有效一些。

01:00:30

你原来的基础学它的课程设置是跟临床是很相似的，然后他这边的话是删减了一些临床的，然后多了一些直接跟科研相关的。

01:00:42

你现在就是做了人文社科这个项目以后，其实科研方法不太一样，你是怎么去习得这个方法是导师教你，还是说跟同学一起探讨，对，这都有对。他是教会了你一个学习的能力，你包括大二他可能是说生命科学和不一样，但是同样的都是你就保持学习这样你这样这个能力他是无论你换到任何的一个其实都是差不多的。

01:01:13

学习能力是你觉得你是从哪里吸得的？

01:01:17

首先你要学会看文献，你要学会基础的就是检索文献，然后去看文献，然后这个课我会感觉有点像。

01:01:27

图书馆检索课你们有吗？

01:01:31

我们没有这个课，因为我们是直接去因为，你必须做文献汇报然后什么的，逼着你必须是彻底高效，然后掌握这个东西，你文献检索还有文献，所以你跟师兄师姐他们讨论学习的，对这些东西是他所以这些还是和就是说实验室里的得到的一些东西，可能跟同学还不太能获得这些信息对。

01:01:56

实验室里他们师兄师姐也很愿意和你们分享。

01:02:06

信息也愿意带你，对。你还有问题吗？那是最后一个问题。

01:02:18

最后一个问题，国中班的学习是否达到了，你的预期就是和你当初报名的时候所想象的能得到的东西是不是一致的？

01:02:34

有没有达到或者超过没有达到。

01:02:38

对他肯定是打到了，我当时是带着要想提高自己科研能力和满足自己的兴趣探索这种，虽然他否定了我的兴趣探索，但是他也是某种印象对我的兴趣给了一个回复，然后同时他也是应该是付赠了一个这样子让我看到其他方向的一个机会。

01:03:01

所以他应该是超过了对他的其实，你这个项目组这个同学是你国中班的同学对吧？它也是基础医学吗？别的还是预防医学转到基础这种。

01:03:14

不是因为他国中班基础和预防是放一起的，你们在上什么生殖生物学这些课的时候，你们是拼在一起上的，对拼在一起上课的，或者是开会或者说一些集体活动的时候是在一起的，所以你就会有交流的机会。

01:03:35

你们什么时候会分开来呢，因为我看就是运行课程运行里面是基础医学括号活动班，还有预防医学括号活动班，就是上一些你本专业特有的课，比如说我们这种病理病生，可能预防那边学的课时学可能跟我们但是对少一点。这种套衣风不他们可能对。但是这种像什么国中论坛或者是什么，包括我们实习也是在一起的。

01:04:10

所以交流的机会还是有的。

01:04:12

你们实习也是在实验室里实习，实习对它是分两步，一部分在实验室学习，还有一部分是在医院在市妇幼。是不要做科研吗？还是临床实习？为什么会在医院做临床实习啊？这个可能我觉得叫你们每个人都要去吗？还是说每个人都要去，一部分人在这一个人在然后对调，不大家一起去那边。像我们会馆都会去实习，因为目前实习跟着医生去轮转那种实习见习查房去，你们也是。

01:04:53

对已经经历过了吗？对已经结束了。行为管权对想来5人问一些吧，然后你你是大几呀研二研二研究生，对。出国深造你有，所以说你现在有在申请出国没有？暂时没有了，暂时没有是吧？你的，父母有在有接受你以后可能会转投人文社科这个决定吗？他们现在感觉有什么说法或者想法，或者有他们现在是接受我做基础医学了。

01:05:34

对，他就想接受你不做临床去做基础，对，现在要接受你不做基础你做什么？

01:05:42

对，我觉得他们可能我跟他们讲，但他们每次讲虽然他们像失忆了一样，我每次回去了，然后他们就说你好好读你的接触一些什么的，我说我说妈妈不是跟你讲了吗？我在考虑其他的我妈就不说话了，他说你吃饭就这样子一个过度了，所以你自己其实心里也清楚他们也是有想法的。

01:06:08

对，他们可能花了几年或者是怎么样，已经接受了这样的一个事情。但我现在想的是他们既然能接受这个，也不是不能接受，当时出国深造没有对你造成冲击吗？觉得国外的科研，因为你是大几出国的？二大二。当然其实当时还是处于在基础医学那个阶段，当时还是想主要还是提高自己科研能力，什么的，你会发现国内国外可能还是有差距，以后没有觉得出国会得到更好的资源和条件，可以调用更多的东西来满足自己。

01:06:46

因为当时还远才大二，我们当时出国去看一看是吗？

01:06:51

对，去看看在大学里项目。没有在大学里就是上几堂公开课那种去了多久？月一个月。所以你觉得出国深造对你来说，我刚刚说的民和的事情是吧？你会发现还是要有很多资源，对你对见识见实习有什么看法吗？或者想感受吗？没有可以不说。印象最深的事情，或者说你觉得没什么影响，那就不说。

01:07:30

嗯我觉得如果是可能我还没有有其他兴趣的时候，如果是在生殖医学这边做的话，可能还是会有印象的。

01:07:39

的确他有一些甚至那些一些点都很触动，无论你是在胚胎室，还是说在生殖中心那边轮转的时候，他因为跟你所做的之前的科研是有相关的，所以他还是会让你真实地感受到一些应用价值，应用价值以及它出现了一些很多暂时还没有办法解决的病例或者是什么的。

01:08:09

因为老师他也会跟你讲，所以说你这个时候会想一下，就是说也许可以临床合作，然后解决这种问题。所以你的意思是对于那些还在探索自己兴趣的同学来说，其实还是很有用的，但是你已经找到自己的心意点了。

01:08:26

对。

01:08:27

国中班还有一个特色5+1+3，我觉得对你来说也没有什么用，因为你以后也不会想再去参加他们的硕博的项目，或者说博士后的项目，对暂时没有，但也不能这么绝对就是解释从自己的兴趣出发可能不会去选择的，但是以后从父母或者是说还有一年多，如果有什么可能这个课题如果他暂停了或者什么的，感觉有可能，但是目前它已经不是我的主流选择了，但是我觉得父母可能会影响你，但主要还是你的选择主要还是基于基于，你自己的一个兴趣或者一个想法来出发的，对吧？你的父母也不会过多的应该说干涉阻挠你的选择，基本上还是尊重你的，对吧？你的感受，你再给你一次机会，你会选择来国中班吗？

01:09:29

再给我一次机会。

01:09:32

是如果要复回到大一回到大一只，嗯不好说，如果回到当时那个情景，你自己还是会选择的，因为当时你有那个需求，所以你还是会选择的。

01:09:49

如果现在再给你一次机会选择121，或者说我们换一个说法，有一个我是一个学弟，我来问学姐国中班怎么样？我比较我想读研，我比较想考研，你觉得我要报各种办法，我会想问他他自己想读什么样的语言，如果他真的就是说对是那些有兴趣，而且是说可能之前有接触过实验室，然后也想一直读下去的话，我就觉得这是一个很好的推荐，对。

01:10:23

还有什么问题吗？

01:10:27

没有。

01:10:29

我们就差不多到这。

01:10:31

好的。

01:10:32

很感谢你你把录音其实我们把很感谢你配合我们做调查，我浪费了你一点时间，我们把我把餐票拿一下。
