# Supplementary material for: Why do you choose this program?—A decision-making model of medical students based on grounded theory
Source: PLoS One. 2023 Sep 15;18(9):e0291634. doi: 10.1371/journal.pone.0291634 (PMC10503722; doi:10.1371/journal.pone.0291634)
Supplement: S1 File — (ZIP) [file pone.0291634.s001.zip › RAW DATA/P2.docx]

Female

00:00

Hello students, our side is the institute of medical education, and then we want to first give you the ethical instructions of experimental ethics. This interview was conducted on a voluntary basis, so we want the interviewees to be able to truly express your own thoughts and perceptions. Secondly, you need to confirm that you meet the conditions for our interview, that is, your grades should meet everything.

00:37

In the process of all our interview, we have a recording, recording data is the use of its form is scientific research, is that we finally want to form a scientific research article, but in addition to scientific research this purpose will not leak to any third party, in the process of the interview, if you feel any questions feel uncomfortable, you can refuse to answer. After the interview, maybe after a period of time, you think, well, you don't want to be used for scientific research, you can also tell us not to use the recording data, this is the instructions of our experimental path, do you know and agree?

01:17

fine. Next, we just started the interview directly. At 10:30, the interview time is about 45~90, just to see how the whole process goes on. You first briefly introduce yourself. Ok, I am in the national middle class of basic medicine of grade 17, and then the original major is also the basic period of basic medicine.

01:44

And I missed a point, The main purpose of this interview is to restore the process that you go from freshman year to registration to presentation, to the selection process, To this point, Finally, the restoration of the whole learning process, In this er, in the course of our interview, We hope that you can speak out, You can tell what happened to you, You can also tell you how you felt about your mood, You can also mention your past life experience, Or the cognitive understanding, Did it affect you once once, For example, the above choice of a certain study can be said, We are an open-ended Q & A, Our problems are also very few, Mainly through a reduction of the whole process, We will extract information and information from it.

02:43

So hopefully we might talk a little less in the whole process, we might guide us a little bit and ask, something of you happen or something in the whole process, right? At the beginning of the interview, we want to because we interview today is about the middle class, so we want to interview the first of the question, we want to know when you in a big one in the middle class, what do you know of guzheng class, and how to understand, you can tell the whole process to us.

03:25

Because we should be the first Chinese edition, before we are not about the concept, but know the state key laboratory this laboratory freshman, because we are basic medicine this professional, so it itself to scientific research teaching or your scientific research is to have some restrictions, and then was a group with a big gen, and then was in the genetic laboratory, then genetic or have some relationship with the birthday.

03:55

Then I have heard that our Shenzhen laboratory other mentor or direction, and then because the big gen is not very well, then because itself is new, he own learning is not a lot, and then add because do big, it may be a team or what we are not particularly experienced, then thought is if you want to do scientific research, may need a better, or said to manage you a platform, and then have the middle class, you will I go to sign up.

04:29

So for you, maybe you think that one of the most important things for you to attend the jelly class is whether it will help you in scientific research, which is one of the most attractive points for you.Yes, it's because of that that I want to hug him. I'm curious. You just said that you participated in daInnovation and you were very interested in scientific research. Did you choose basic medicine major? Or what is it like for your parents to choose this process?

05:04

High school when this is my choice is more wonderful, because I have some fear of clinical, is my home is unique is very support, and then itself has a little dizzy blood, and then plus because I was interested in biology, so when the medical school, this time I fill is basic medicine, including some other volunteer, is other universities volunteer fill is also basic medicine.

05:29

Therefore, after I came to this school, I found that the basic medicine was not voluntary, but I was very special and right, so I should be very active to contact the laboratory. You just said that you have the experience of big innovation, when you signed up in your freshman year, did you? Well, right. The big creative project is a topic that you think about for yourself, Or do you discuss it with your tutor, Or if you discuss such a project with your senior, If you want to choose this project, you should choose the lab first, Then the direction I was previously interested in was the party treatment for cancer, So here's a look at what laboratories or directions we have in our school, Then I took a look at the genetic lab, He has a teacher who works on tumors, He is not saying that studying Party treatment, He means he once gave a lecture about, And then I probably thought their lab would go in that direction, Then I met with my senior, Big the was intestine in the lab, Yes, because their lab is in pancreatic cancer.

06:44

Li Gang said a very professional term, which is the treatment of cancer particles, and I want to know if when you choose this direction, have you ever discussed these things with your family or with your classmates? In fact, there is some, because actually started very early, when there was in high school, and then our high school biology teacher because I participated in a biology competition in high school, and then the teacher told me that car t should be very effective, but the current progress is not very good one.

07:10

And then the family was because the elders got sick, For the targeted therapy, Culture therapy for cancer is very popular, But his problem is that the effect is not very good, and I read it, After all, it's these elders who don't get particularly effective treatment either, So since I chose this major later, I want to see what it's going at China, And if you can do anything for yourself, So you chose the direction of big innovation, Then you and a senior group of a big creative group, Senior senior, senior in the lab, Senior senior, senior in the lab, Then they advised you to learn more about the lab.

07:59

Right is staying with them in the lab. Because later they said that because the laboratory does not do that direction, and then he said tumor treatment, actually in the end will be targeted treatment, but you want to know a lot of mechanism, and then you will feel you only understand a point, and then you also listen to their advice to understand, and then you began to do big, when you have to attend their lecture? The government of the state attended. Do you know more about it through other channels or channels, or do all of your information sources about the class come from that lecture?

08:43

Have you ever discussed this with the counselor or the seniors or your classmates or family around you? In fact, there have, yes. I want to hear what you did with your family, what do they think, what do they think about the class? Because my parents of his home older than my some brother and sister know I read the professional, advice home is to consider going abroad to do scientific research direction, so they think that the class, they think will affect, there will be restrictions, may you have to always read on, and then can't go abroad or something.

09:25

Then later in the briefing has said that there may be joint training CC or other ways, and then I tell my parents, my dad said that also, maybe for you your parents, they are more concerned about your future development may be with go abroad these things will affect to, because is brother and sister are so suggested.

09:51

Did you accept their advice yourself then? At that time, I did accept because, yes, because after I was a freshman, I also signed up for a visiting project. Is your visit plan in basic medicine or the whole school? Is it in the whole school? Is the visit plan related to your major? At that time, I visited Wisconsin, and Wisconsin was the University of Wisconsin. It was a stem cell therapy, and it was one of the most famous ones, so I wanted to see it.

10:27

So for that time, everything you know about the fire middle class came from that lecture, after all, it was the first one, right? There is no other channel, and then and er is mainly with the family. Did you discuss it with your counselor or your classmates? Have they ever heard what they think like?have. In fact, the counselor went to ask at that time, he also felt that the class was very good, and then the students in the class were changing major and examination activities, the students were busy with these two things, and then in fact, I both reported, and then later words changed major, you just said you changed major, right.

11:10

Where did you apply for your major transfer? I reported was my mouth. Is the mouth? Is he this is also recommended by his parents, one is the sun or have a lot of worry, worry that maybe you may do the foundation to go a long and long way, or worry about some work, so they suggest that there is the opportunity to change the major to take an oral test, take a long way to mean?

11:38

May go after scientific research this road may read a long time is longer, they mean this meaning, yes, they generally feel both at home or read abroad, you have to go for a long time, that is to say you may read after reading down, maybe you read in the domestic doctor, you also go abroad to do postdoctoral, this he thinks this is a very long.

12:01

But you just said that you are more interested in scientific research. Your parents, I think you are also a classmate who is more willing to communicate with your parents.

12:13

Your parents in mentioned later, such as technical medicine, may take personal long time, walk longer, you know what to think in the mind, but I think your time can spend, actually I worry more is think you listen to the parents after these advice to you, what is your attitude towards scientific research, or what is your mood.

12:37

How to say before he also have a little understanding, Then the parents say or are friends, Just because I have friends who are studying in other majors or not in my school, They also said to me, But how, We all think that reading may be more like a utopian or a pyramid than being at work or something, So my parents and I think reading might be fine, But then it has changed, By the junior year began to have a feeling, It's like the feeling that you've been in a siege is that doing research is what, It seems that not seeing it is just like a head, The feeling of not seeing a head or the feeling in a siege is something that affects you, It suddenly makes you feel that way, Or is it a slow process?

13:45

Well, I think is a slowly accumulated, he is different from you imagine, with you first own to scientific research or say you imagine the ideal of a little began to slowly offset, of course, the offset, but you more slowly accumulated, feel the offset is bigger and bigger, away from your conceived at the beginning.

14:08

Have you ever thought that now did so much time to do so much scientific research, and you were in high school ideal offset, have you summarize where it comes from, or can it be like I casually say, can it be the experiment failed too many times, or he didn't reach you was just want to do scientific research, change the human society this vision may feel too difficult.

14:40

Is it too difficult, or that there are too many setbacks in life, have you ever summed it up?

14:46

I summed it up, but what you said was actually really happened, but it was not something that was very difficult for me to accept or sad.

14:59

On the contrary, I felt that I gradually began to feel very helpless about scientific research. I said that discovery is a fault of knowledge, you is not knowledge, and you understand the joke, and you do this thing is completely different from what you think.

15:22

You may be thought is a person doing, when you really do one thing, it is a surrounding environment, so you have never imagined that environment, after you really go in, you found that your environment is a closed laboratory, is a closed cell, or is a closed experimental bench, he these concrete imagination, concrete actual with you for the imagination of scientific research is completely different.

15:51

Another thing is that people can't live in the ivory tower or in the utopia forever, and you have to have a lot of contact with life actually. It doesn't matter if you thought before, maybe or if you were naive to your friends, but when you grow up, you will find yourself slowly taking on a lot of responsibility. There is, of course, this is my personal feeling relatives around relatives he may be because also more emotional people, you will find some powerless, sometimes I will blame yourself why not to choose clinical, especially in medical school facing this kind of thing will be very very remorse, because the score is not not, there is a period of time will be very anxious, blame yourself, if you can regret, you will not choose this way.

16:48

There is a period of time is immersed in such a mood, I from it to two weeks, I have your first information I want to ask, you said the first point is that feel yourself, knowledge fault may be no way to do yourself from the original fantasy of scientific research environment, I want to ask you remember when high school for the fantasy of scientific research? It was the one point or some aspect of scientific research that made you want to do it in high school.

17:20

Because actually to science news agency may be an induction of dad, is we he will take me to see a lot of science and education, and then include some very interesting animal documentary, then your idea of scientific research is very naive, is to think you are a field record, and then include is observation, and then equivalent to an aspect of the animal behavior.

17:47

And then you get to medicine in high school, and then car t treatment like that just mentioned, and then you think it's a clinical application or this, but when you get to college, you get to research, you find that it means you have to shut you in a room to do things.

18:11

Step by step out of your original imagination, will not feel a little boring?

18:21

In fact, you have no, just feel that their knowledge is not supported, there is no way to support these steps of scientific research.

18:33

He said I just said that knowledge fault is not right, a fault of information, his real world with you imagine is completely different, and when I was a child, right, including your high school or what, whether you visit the science and technology museum or what, it is not the same, right.

18:53

Scientific research is the whole process and your understanding that you might know something in high school, and I think something is very interesting, but when you actually explore, you find it difficult to explore together and immediately explore some effective results.

19:14

Yes, then unhappy, you can be very happy at the beginning, when you are happy when you contact this topic, when you understand this topic, it seems to start to think about a lot of things, and then you slowly do it, it is a different feeling.

19:37

When doing and understanding, there are two feelings, the second point you just said, something may happen at home, let you feel anxious, is that you may feel that you can not help, so only a little bit is regret, reported to the technical medicine, right?

20:01

To.

20:06

Home this matter, not convenient to skip.

20:10

Can skip.

20:14

You just asked if you discussed the national middle class with your family and the national middle class. Have you discussed with your classmates, because you have discussed this matter with your classmates, have you communicated your ideas with them?

20:31

Yes, whether you refer to the classmate, or say that can dormitory or the same class, or walk on the road encounter and so on, can make you a deep impression of things, or affect your things and so on.

20:45

Because I have a friend who contacts social medicine, and then are he and my friend your classmates? Or that the rest of your school is always right.

20:54

So he and I were actually with him before high school, We can do some interesting things together, Not considered as a social survey, A discussion of some topics, And then of course I've always been very science-oriented, So he was, and I told him at the time, When I might be able to switch from tumors to reproductive research, He actually means that it's very interesting, Because we were all talking a lot about the same sex, Then the sex education, Then some of these topics, Because we focus a lot of society and certainly say that lgbt is of course some of his focus, We've all been exploring it, So when reproductive medicine is when I talk to him, We are actually really excited, Because we knew to follow some reports, It's just something about the cutting-edge, That is s same-sex birth, That means you can induce a sperm and it goes to become an egg or an egg and it turns into a sperm, It's the same sex that can breed offspring.

22:05

So it's an exciting topic, That's why I said you first understanding reproductive medicine was a thing that made you very happy, Because it does have some of your previous interests, He collided and will make you happy, You feel like you're in the research, That's what you can do in this direction, So this is something he really encouraged me to read, So the discussion with your classmates inspires you even more, before you discuss with your classmates, You mean you really want to participate, Or is I not sure I want to be with him, so I want to participate, The reason I wanted to participate was because I thought I didn't do scientific research when I was a freshman, I don't feel like enough about my experiments or my abilities, Then you will discuss it with your classmates later.

23:00

Yes, you will find more interesting places and feel like encouraging a question.

23:09

You can ask, I think I'm alone there, we are in a chat process, you're at level 17, right?

23:23

So is five middle class has for three years, big 4 I before the middle class preach PPT, have seen some of his reform model a characteristic introduction, there is a few points I tell you, one is a bonus research system, and a characteristic building internship, immune priority, and 5 + 1 + 3 a doctor model, and a go abroad for further study, which do you think which is the most attract you a point?

23:58

I think it's a scientific research tutorial system.

24:00

Well, at the time.

24:02

For research mentors, you are senior, right?

24:07

I should have already chosen a mentor by my senior year.

24:10

In my junior year, the research direction was, as planned, well, yes, but in my sophomore year, I was already in charge of a topic in my sophomore year.

24:20

Well, did you choose this topic in your sophomore year and junior year?

24:27

you?

24:28

You just talked about a social medicine student about changing a sperm turn to an egg, which is a genetic emergency elimination and then converting cells. Of course, I saw that our laboratory did something. I thought I could choose this direction for scientific research after I went in, but it did not. Therefore, the topic I did in my sophomore and junior year was not selected by myself, but provided by my tutor, so I could not choose this reason at that time, but he was not selected. No one was doing this when I went, and then there were other topics in the laboratory, and there was no one for him to do it.

25:09

So the tutor means that since you want to do a topic, if you just choose one from the middle, for example, I just made a hypothesis, when you originally wanted to choose this direction, you will find that there is no tutor in this class to do this direction, have you ever thought about something like quitting?

25:33

Didn't want to quit, because at the time because is two reasons prompted me to this process, there is a feeling their research is not, so he has to improve, so of course is to stay, after all, sophomore I didn't learn anything, just to learn him, said to see yourself, perhaps is equipped with these abilities, to do something interested in. Although the topic and their topic at the time, but can still train their scientific research ability.

26:09

So that keeps you going to continue your study, and still again?

26:14

You said that there were two reasons, well, one is scientific research ability, and the other is their own happiness. I think I just think I can do it in the future. With this ability, I can take the initiative to carry out, I am interested in, right.

26:31

Are you still interested in this right now?

26:33

I've changed my tutor and changed my topic right now, Because in the course of the past two years, I slowly found that, Maybe I was too naive, You don't do confined to your own interests, Just if there are a lot of your resources, Including your mentor, Including its overall orientation of the laboratory, Then there is even a whole one-lab environment, Then all you can learn is the time coming in, Including even a choice of your experimental animals, It is all a very difficult thing, Like if you want to experiment with macaques in our school, It's actually a very difficult thing, So when he studies and you do what you want, That was a very, very difficult point. You you said in the process is also a fact, many scientific research choice is not based on their interest choice, you learned slowly after a lot of things, such as bridging these things, and the whole topic topic after these things, did you have any idea, think there will be a sense of frustration, have?

27:49

Have this is before I feel their information fault, real is not you are so good idea, may you think scientific research is want to do something interested in, to study some human unknown things, but you now direct directly, found a lot of many links will be blocked by different degrees, or say there is no way to control within your ideas, such as human or resources call these well, then there will be a possible call frustration more appropriate this kind of feeling.

28:31

The gap is not quite the same as when I was in high school. To. The gap is too big. Well. When you know that there is no way to make up for the gap and what, you will find that this wife has lost me straight.

28:54

You can talk about one thing, one thing, you are in, for example, you want to do a scientific research subject, but found that resources is not what you want to use can use, such as macaques, I want to listen to what you have, because it is a he is not the main thing, he just I used, for example, is he was I went to the United States, and then tutor asked me American laboratory and China, and then because is not the laboratory is quite famous, he is as a place to visit.

29:30

So we saw a lot of little macaques like a zoo, and what else there were other kinds of monkeys to implement some common experiments. But our laboratory we have an animal center, actually our animal center is not raising monkeys, and then he asked me what, I only mentioned I said the impact to me is quite big, I think domestic research is not so big, and then my mentor said that there is such a gap.

30:06

Then he told me at that time, we now don't need to do the monkey of such an experiment or subject, then I say is but then you think well, if even if you are now to our school such a do has become a very good professor or what, when you want to say maybe my experiment or subject need to use a monkey or what, actually you have no way to use.

30:35

You said that there was a period of time when you were very regretful. I continued as our class. Do you still feel this feeling now, but it lasted about three or four months or longer. I don't feel it myself. It was my junior year.

30:58

How did you deal with this negative mood later?

31:05

I don't know to continue to read to do, I feel I have been self-regulation ability is quite strong.

31:13

Because this mood because of some changes in the home, feel helpless, why not learn clinical this regret, is once into the regret, he will repeatedly regret, and then because the home with home may go with your parents because they also accused me why not choose clinical, so I will be more regret, he will be into a circle, once wanted to quit school, to tell my parents I to the college entrance examination, not to say you to continue activity, what is good have to give you this idea?

31:59

A friend or a relative.

32:01

No, so the whole process is your self-digestion, yes, so actually no, I think I should not tell my classmates about this thing, I will tell my friends, and then my friend said that if you really want to quit school, I will also support you.

32:17

Then to that point, and then it took a long time to calm down, calm down after feel still want to do some feel sure or valuable things, began to want to prove that we choose what kind of wrong, also did not produce such a change, was not thought of the first idea is I want to change an environment, but then has missed the heavy, out of such a.

32:55

Can't you quit at any time? He is going to study at the beginning of the time, right, otherwise it is very difficult to connect. For every year of the right. Then what I wanted to do was to change the environment, so later, and then I encountered the epidemic, I was very anxious, a little depressed, and then I was even more uncomfortable at the stage of the epidemic. Because my friends liked to discuss social topics before I was good, so it was also a very difficult time for me to share with Wuhan.

33:31

Then, of course, I didn't know that I returned to school later, and then I began to contact our school and began to build an emergency management system called the epidemic. Then I wanted to change the environment instantly, so I went to participate in it later. Is this a big innovation project? Or something, it's a big creative project. How did you know anything about this thing? Because the national middle class students are doing, he invited you to join his group.

34:02

After he said to me, because I said I want to change the environment, I will ask if you this is more partial social science kind, and then feel and then during the outbreak, my idea is that I want to change the environment, and want to be their own must is not so bad. So you changed your mentor and your project, and you changed your mentor. To. Is this like a unified mentor with him at this time? Change is almost the same direction, but with his mentor is not the same, but these several mentors are doing this thing, you are not sensible can also, so change, you think the teacher has no idea.

34:47

Yes, because actually this time because before sophomore junior, actually communicate a lot of mentors, we are because I brought a lot of enthusiasm came to the laboratory, so do subject has always communicate with him and then what, so then appeared some including some of their cognitive gap, and then back with a change in his heart, you communicate with teachers these things.

35:14

No communicate with him too, but he sometimes asked me, and then I have, and then because I started to contact the mentor, because it is good, he is I said he did a sperm mark before, he was done, so I also because of the social topic to ask him, and then he asked me, that is to feel recent state, or do experiments, he asked me the progress full not satisfied or what.

35:46

Then then in the process of this communication, I put forward a point is to feel very confused, or what, and then he said and told me that I am still small, there are a lot of choice is still a long way, so you change the mentor this thing is right, is discussed with the original mentor, but I didn't tell him.

36:10

I didn't tell him where also didn't tell him, just I told him, well is his feeling is need to change, because feel with great enthusiasm and interest came to the laboratory, but don't know the two years because the outside world or because of his interest began to disappear, you discuss with the first mentor, feel you communicate a lot, when he proposed with you, may you have no way to do when high school or with sociology classmates discuss this direction, you feel you remember the heart?

36:44

Will I be very impressed? I didn't know it very well, because I quickly accepted it, because I thought that I was here to improve my scientific research ability. Ok, and then a mentor, an environment, and a new project, and your mood changes. Yes, because this incident happened very regret, those emotions were taken away. I did, and he slowly got better again.

37:15

So in fact, you are changing to a project that you are more interested in, that is, you may have a new interest point, that is, you feel that the interest point can make you more sure of yourself.

37:28

Your main consideration is your interests, not your parents' advice or employability. Think so like yes, to feel happy to do things, if you can't do it, you will fall into a very long period of doubt. A good tooth. We want to hear you from the middle class should be called for three years, yes.sophomore, junior and senior. In the past three years, you feel the most one impression is one thing, or what is the biggest feeling? The happiest thing, or the most dissatisfied thing, can be said to be the most impressive thing.

38:22

Impact the deepest thing is one day should be bad mood, may be after communicating with mom, and then things at home, then the dormitory I feel very tired, with my roommate said, roommate comfort me, but I don't want to listen to, because I think I listen to I will cry, then I went out, I went to the sea building is scientific research building, and then went to school to our laboratory, then walked an aisle, then have to hold back, is too uncomfortable.

38:53

I was at that time that I thought I would shed tears the next second, and then my elder brother told me, is your project report ready? He stopped me, and then I was born alone, all his emotions were held back, feel that a moment I was the most memorable. Corporate work for me but also then I was writing my diary, I said I really adult collapse, was that I felt that the first adult world didn't need tears.

39:26

So you were in the corridor is very dark, not so understand him, didn't feel you very sad, no, because I go quickly, because I don't want to cry, I think I wanted to back to the laboratory, may see the literature what should calm down, but he felt asked me said your brother is to know you have this mood, or is a very straightforward way to comfort he don't know.

39:55

His real questions, yes, he will really ask you if the topic report is mainly internal, there is that kind of dinner or other communication, will it matter or? The relationship is still OK.

40:07

Yes, but it's not very good, I don't want to say it, so he doesn't know much. So this kind of is actually more private things, may not be with the real laboratory has not have that kind of good to follow because they are, after all, elder brothers and sisters, is you want to respect at that time you said to return to the laboratory, read the literature one, it was night, right? No, I'll finish in the afternoon and finish the evening. In fact, at that time, you mainly wanted to stay alone, don't want to communicate with others, just want to own internal digestion, right.

40:47

This has then affect you will not feel what do experiments, are how so inhuman environment, how so really, actually didn't think so much, but oneself think what is at that time, was really feel a little too uncomfortable, too won't win, before you have said because you said people can't stay in the ivory tower forever, or and people have what interaction and so on, do scientific research to sink to calm down to a person to do this thing. Will this thing have such a change to you is to induce a person? Guide, because you talk about this thing suddenly let me think of you said something before, I think they are connected, you are growing up, you slowly begin to find that you can't really think about what you think about yourself, you have to communicate with a lot of things.

41:52

Well, this is when you start to think seriously about what your parents say about work, or then what will they work look like? They are asking what is your employment or whether you want to go abroad? Then, or say you, if you go abroad. At that time, there will really be some consideration, before is that kind of thinking, may be enthusiastic can be interested to eat, but later you began to slowly find that it is not, you really have to think a lot.

42:25

Especially when everything is good, you don't have to consider so much, but once there is a balance point you disappeared, such as possible family or relatives have something balance is gone, then you start to consider employment, if you study abroad spending, and then you can you really for, family or family do something, this time will be real consideration, feel he is a little grow up.

42:59

Will you still consider going abroad now? Now also will test, now is to two wiring go at the same time, also not as the direction of do now, actually because he is out, will not fall into the negative doubt, and then regret that a vicious circle, when you slowly come out, you see actually doing this thing or as long as it is to do, whether work or what, you can get slowly, don't so is don't believe in yourself.

43:39

Now I think so, that is to say, if you can read it by then, you need to go abroad or when you can consider it.

43:48

Because later also told their parents, they actually later my mother said whether to buy depression and anti-depression drugs, because at that time was already like that, then also told my mother, I have a point to buy, also quite sad, is I was at that time to accompany in the top floor of the hospital? One of my big thoughts was if I could jump from here and feel sad.

44:16

At that time, then I went home and told my mother, I said, mom, I didn't have such an idea before, why did he finish me up in the whole interview? Then I said we had one and then I haven't had a long time to say that to others, just what I said to my mother.

44:34

Did your mother blame herself?

44:38

Then me, My mom said because she was that time, Very busy, too, He himself could not outlive his own emotions, Right, and then again, not right, I said our time was too sad, I would say that, if I could, Mom, I thought yesterday that I wouldn't have so many things hanging around me, And then my mother said, I was maybe I was then I thought it made me start slowly I probably didn't think I'm going out at first, An idea happened to come, Because already I was stuck in there, Then my mother told me, That means he told me that there are so many stages in every time of his life, He said that at this time a lot of things are you don't need to think about this is mom to think about things.

45:31

At that time, after my mother said that words, I began to feel that he is a dilemma to pry open a hole, I think I may think wrong, also think too much, has been around. When you stop, stop, don't think a lot, stop, and you can see if you can go out, so that's a change.

45:57

Your mother is usually in the process or your parents to you is these choices, I think one should be generally more supportive. Right, you wanted to apply for basic medicine, you didn't apply for clinical medicine, and your parents should have agreed, right? They still prefer you to follow your ideals. Yes, they are different and have no way, because I may be so actually they are still a little reluctant in the heart, but said that since you want to choose, then you learn.

46:29

Yes, because I feel that since I was a special child, my mother said that there are special wrong ways, this feeling, but my mother will tell me repeatedly, which has formed a kind of my own fear of myself, and your mother said that you can choose, but you must be responsible for you, must be responsible for yourself.

46:47

So when I have always been very regretful, I feel like I can not take responsibility, can not take responsibility for themselves, so this mood may also come from you, you, you, your mother is a kind of transmission, is your, the idea is not wrong, is to choose their own choice to bear the consequences. Now should not everyone will have like me so have such an English situation, I think it should be right, I should be an exception.

47:24

In the future in you now also senior, immediately five five, have you talked about graduate student this aspect is how to consider? Should you continue your school or go abroad? Because I have actually basically changed the direction now, before it may be pure appreciation. To. Yes, if I read pure reproduction, I should stay in our school, and then read it because what I is doing is an emergency management system of the epidemic, which is already in the direction of social science and health management, so I am considering whether we consider the future graduate school or emergency system, yes, I will consider the direction of neglect.

48:06

So in the final analysis, whether to continue basic medicine or management is not very clear than myself, and I want to have a look. In fact, I feel that I already have a very much bias in my heart. Do you think you are still very interested in this system and this research project. How do you feel about it?

48:30

Feeling is he let me feel my new interest, I think he from a certain extent he helped me, let me feel not so not so bad, because our project it need a lot of interview found, or something makes you think you participated in the project has a sense of achievement, or let you say I think there is one thing or a feeling in that, will make you think you let you feel not immersed in the cycle of self-denial.

49:10

have, Yeah, because I just actually wanted to say it, Because we have this project and it will do, There are actually a lot of interviews still needed, Like we now so I do interviews, I will make me cry and I feel like I've done a lot of interviews, We have an interview with this one, He's going to Wuhan, About this time, And then I was very motion sick, Then, uh, during the outbreak, I watched a film from someone else, That means I am sick in Wuhan, He is now without a car on the road, Then I was also sitting in a taxi in Wuhan that day, Wuhan is a busy road, His road was all stuck in traffic, Then there are so many cars, The first time I felt like the second half of last year was like the second half of last year, For the first time I felt I was not motion sick, My own heart is very happy, Is the real happy, Feel that Wuhan he this disease it is good again, You because of the subject it actually says it's health cause management, It also favors social medicine, It has something to do with medicine, For, after all, a thing of a crowd of the public guard, In terms of epidemic management, So at the time I really had a way that though he wasn't what I made him better, But I'm experiencing such a better feeling, He gave me a great comfort and confidence, Is you go through a good thing, Because he made me think of something very bad happening here before, But both in terms of time and the idea, It is our epidemic that has established an emergency system that has made it all better, He will make me actually feel it, I want to see something about the projects that you are working on right now, Because you just covered a topic, The Emergency Management System, Why the general part of your main interview is that you have a lot to focus on, Your interviewees, Then the problem is just like what we have right now, What is your purpose?

51:19

He is different kinds, in fact, we were the main thing is to say, because during the outbreak we are also compare a beginning, before the outbreak we actually have a lot of panic, and then there are a lot of some may not be loopholes, China has a lot of including masks or some other supplies, a lot of you these things are not the news is you officially happened.

51:42

So starting from these vulnerabilities, You actually want to do a very comprehensive interview, It's the truth to reveal it, That means you think that in the early days of the outbreak, The basics are people like those in the community where you interview the community, What he asked was that you had some difficulties and problems in the early days of the first floor, Then go up you might be some municipal CDC, He may be asking about some disease control, or some hospital leaders, Then he angles it from their feet, Because they are the real front lines, When they face patients, or when they control an epidemic, The real problems they have can be improved, Or what does he want a change?

52:25

This is an interview, of course, the interview is all our initial work, there are some behind you because I am divided into many sections, will you be responsible for the back? Or will it be, too, but keep following up? Initially, we wanted to accumulate a lot of material to provide some data for your subsequent data analysis, including some other things built.

52:45

And then for now, should you have the data collection now over, or in the process? The data design has been over, because now it has reached a stage of a provincial competition, so it is a big innovation project.

53:01

It was a big event for him, and then he was in a Challenge Cup match, and the first time you asked me to get the challenge Cup material, and then you didn't come in.such.clear. I want to know, you you are actually equivalent to participating in a big innovation project that. The mentor of the project is your own research direction, because I watched the PPT of the Chinese Middle class at that time, my understanding is that you should choose a tutor after class, and then follow the tutor to do these research in your spare time, so these can overlap.

53:45

In fact, it is quite likely to be a quite convenient thing, because they may not attend the national middle class, if they want to attend the big middle class, in fact, they also use their spare time to do these.

53:59

For you if you don't participate in the process, if I don't participate in the process, I don't know if I will experience their junior a period is not very good is period, but you experience, but you attended the life you also experienced this period, but you also have an opportunity to know a lot of, such as big gen like now like now this project is actually a typical humanities social science project, should contact with your sophomore promotion is pure promotion research method should be completely different, right.

54:29

Different huh. Do you feel that it is difficult?

54:32

When contacting this research method of humanities and social sciences, for example, when you are in the interview, should you do a semi-structured rebound, or do a complete question and answer method. Semi-structural, vs. But I think these difficulties are ok, because it is very open relative to the closed environment of the laboratory song, so this is exactly what you said before. Because the humanities and social science research is a human research, people and humanities research, it is not like that kind of research, is himself in the laboratory to do experiments, need to calm down to you alone to do, and then to analyze our may need to interact between people to collect information, collect information way, so may be more because closer to your original an idea, or because you this idea prompted you to turn to the direction of the humanities and social science to project?

55:40

I feel that they influence each other, so after you contact, he can make you feel more comfortable is a very fit for your interest point.

55:53

I want to ask your personal character, do you think you are an outgoing or introverted person, you like to deal with people, feel this seems quite outgoing, but he points many periods, I feel if I am not in a period of mood is very peaceful, actually he is generally closed, will adjust their mood, don't say want to because some people like is upset, or sad things, like to chat with people, and then to relieve depression, I feel you should not, you are your self digestion.

56:35

A vision, yes. Yes is. The biggest impression of the Chinese middle class is the change in the direction of scientific research, from a natural science to a humanities and social sciences. In fact, you still prefer the future planning. For the humanities and social sciences, will you feel that your five years are a little wasteful.

57:08

Does it feel like that way? A pity, not preparing recently.

57:14

Some materials, is to see yourself over the years is not research material, yes, because he is not to provide a research is a convenient, so preparing preparing grind material, and then see this all the way is very happy, but obviously I feel big three is his harvest at least. The junior year is also a turning point, yes, should be different, spent a lot of time to self-adjust their mood, self-solve those negative emotions, spent a lot of time.

57:56

postgraduate recommendation.

57:57

Of protect grind, in fact if you got protect grind this qualification, you you will consider the direction of humanities and social sciences, equivalent to finally still say did not think well, think good will go to me I have been in now.

58:13

This side seems to be the country, there his teacher also collected this.

58:20

Collection is to ask your intention, may you stay in the country, or go to other schools and other majors? Don't other majors have some negative impact on you?

58:31

At this time, for example, you say that I don't want to continue to read again, I think it must not be at the last time, if you may be to the fifth year, you seem not to like the language major choice is actually very difficult. If you may have a junior year or senior year of the new direction or have some ideas, you can consider giving it a try, I don't think there should be a particularly big obstacle.

59:04

Another question I also want to ask, is that I saw his preaching PPT his professional foundation is basic medicine or preventive medicine, and then it added reproductive biology, developmental biology, experience forum, research practice, these courses that basic medicine does not have, right? What do you think of or feel about these courses?

59:26

These are actually a very useful some design, scientific research is very useful to you, especially in the sophomore year, when you are doing subject, after all, I like I mentioned your own research ability is enough, so you need to want yourself to learn, he is no system, even said you may qualification or some choice of subjects or knowledge is not right.

59:53

But if he offers you this kind of course, he really gives you a systematic chance to improve quickly. So it's all about meeting your needs, these courses, because you just think about improving your own scientific research skills, which can also improve your own scientific research skills.

01:00:12

You think if you do not serve the country middle class, you go to those classes of basic medicine, you think you can improve their scientific research ability or scientific research methods through those classes, he does not have this to directly, his words are I think to be more effective than the original.

01:00:30

Your original basic learning and its curriculum is very similar to the clinical setting, and then his words are deleted from some clinical ones, and then some more directly related to scientific research.

01:00:42

Now that you are doing the humanities and social science project, in fact, the scientific research methods are quite different. How do you learn this method is whether the tutor will teach you, or say to discuss with your classmates, yes, this is all right. He is taught you the ability to learn, you including sophomore year he may say that life science and different, but the same is you keep learning so that you such this ability he is whether you change to any one is actually the same.

01:01:13

Where do you think you suck from?

01:01:17

First of all, you have to learn to read the literature, and you have to learn to search the literature based on it, and then go to read the literature, and then this class I will feel a little like.

01:01:27

Do you have a library retrieval class?

01:01:31

We don't have this class, because we are directly to because, you must do literature report and then what, forcing you must be thoroughly efficient, and then master the thing, your literature search and literature, so you discuss with senior they learning, so these things and he is to get something in the laboratory, may with classmates will not be able to get the information.

01:01:56

In the lab, they are also willing to share it with you.

01:02:06

The information is willing to take it with you, too, yes. Do you have any more problems? That is the last question.

01:02:18

The last question is, whether the study of the national middle class has been reached, and whether your expectations are the same as what you had imagined when you signed up?

01:02:34

Is it reached or exceeded.

01:02:38

To him must be hit, I was with want to improve their scientific research ability and meet their interest in exploring this, although he denied my interest in exploring, but he is also some impression of my interest to give a reply, and then at the same time he should be paid a such let me see the other direction of an opportunity.

01:03:01

So he should be more than to him, in fact, your project team is your national middle class classmate, right? Is it also basic medicine? The else is preventive medicine to basic.

01:03:14

It is not because the foundation and prevention in other countries are put together. When you take any reproductive biology classes, you work together, for classes together, or for meetings or some group activities, so you will have the opportunity to communicate.

01:03:35

When will you come separately, because I think it is running the course operation inside is the basic medicine bracket activity class, and the preventive medicine bracket activity class, is to take some classes special to your major, for example, our pathological students, may prevent the class hours learned there may learn with us but less right. This jacket style is not what they may be right about. But this kind of national forum or what, including our internship is also together.

01:04:10

So there are still opportunities to communicate.

01:04:12

Your internship is also in the laboratory internship, the internship for it is divided into two steps, part in the laboratory study, and part is in the hospital in the city of women and children. Is it necessary not to do scientific research? Or is it a clinical practice? Why can you do a clinical practice in the hospital? Maybe I think each of you is going to go? Or everyone will go, some people in this person and then tune, not everyone go there. For example, we will go to practice, because the current internship follows the doctor to rotate, so are you.

01:04:53

Have you already experienced it? The pair is over. Behavior management right to want 5 people ask some, then you you are big ah grind two graduate student, right. Do you go abroad for further study, so are you applying for going abroad now? No, is it? Are your parents likely to switch to the humanities and social sciences after accepting you? They now feel like they have any ideas or ideas, or that they are now accepting me in basic medicine.

01:05:34

Yes, he just wants to accept that you don't do clinical practice to do the foundation, yes, now you have to accept that you don't do the foundation, what do you do?

01:05:42

Yes, I think they may I tell them, but every time they say, although they are like amnesia, every time I go back, and then they say you read your contact with something, I said I said my mother didn't tell you? I'm thinking about the other people, my mother doesn't talk, he said that you eat too much, so you actually know that they have ideas, too.

01:06:08

Yes, they may have taken several years or how to have accepted such a thing. But what I think now is that since they can accept this, it is not unacceptable, then going abroad had no impact on you? Think foreign scientific research, because you are how to go abroad? Second sophomore. Of course, I was still in the stage of basic medicine at that time. I still wanted to improve my scientific research ability, and you will find that there may still be a gap at home and abroad. In the future, I did not feel that the congress can get better resources and conditions, so I can call more things to satisfy myself.

01:06:46

Because it was far away and only a sophomore year, we went abroad to have a look, right?

01:06:51

Yeah, go see the projects in college. How long did I not take a few open classes in college? Month a month. So you think going abroad for you, I just said Minhe, right? Will you find that there are still a lot of resources, what do you think about the internship? Or do you want to feel it? There is nothing to say about it. The most impressive thing, or if you think nothing affects it, then don't say it.

01:07:30

Well, I think if it is possible that I have no other interest yet, and if I do it here in reproductive medicine, it may still be impressive.

01:07:39

Indeed he has some even some points are touched, whether you are in the embryo room, or said in the reproductive center there rotation, because he with what you do before scientific research is related, so he will let you really feel some application value, application value and it appeared a lot of temporarily not a way to solve the case or what.

01:08:09

Because the teacher will also tell you, so you will think about it at this time, that is, maybe you can cooperate clinically, and then solve this problem. So what you mean is actually very useful for those students who are still exploring their interests, but you've already found your own heart point.

01:08:26

To.

01:08:27

The national middle class also has a feature of 5 + 1 + 3, I don't feel very useful for you, either, Because you won't want to go to their master and doctoral program again, Or a postdoctoral program, For the moment, if not, But you can't definitely explain that you may not choose from your own interests, But more than a year from parents or speaking, If there is anything possible for the subject if he suspended or something, Feeling that it is possible, But right now it's no longer my mainstream choice, But I think that your parents might influence you, But the main is that your choice is based on, An interest or an idea of your own starts out, Is that right? Your parents won't interfere too much against your choice, and basically respect you, right? Your feelings, you give you another chance, you will choose to visit the national middle class?

01:09:29

Give me another chance.

01:09:32

If you want to go back to the freshman year and return to the freshman one, it's hard to say, if you go back to that scene, you will still choose, because you have that need, so you will still choose.

01:09:49

If now give you a chance to choose 121, or let's say it another way, one I am a junior, let me ask the senior national middle class how? I compare I want to read graduate school, I want to one's deceased father grind, you think I want to report all kinds of ways, I will want to ask him what kind of language he wants to read, if he really is to say to those who are interested in, and said may have contact with the lab before the time, and then also want to always read it, I think this is a very good recommendation, right.

01:10:23

Any more problems?

01:10:27

not have.

01:10:29

We are almost about that.

01:10:31

fine.

01:10:32

Thank you for recording, actually we really thank you for cooperating with our research, I wasted you a little time, we took me with the meal ticket.
