# Supplementary material for: Why do you choose this program?—A decision-making model of medical students based on grounded theory
Source: PLoS One. 2023 Sep 15;18(9):e0291634. doi: 10.1371/journal.pone.0291634 (PMC10503722; doi:10.1371/journal.pone.0291634)
Supplement: S1 File — (ZIP) [file pone.0291634.s001.zip › RAW DATA/P3 CHINESE.docx]

00:00

好，可以开始。了，开始了。同学你好，我们是医学教育研究所所的研究人员，在访谈开始之前，我想先跟你说一下实验道德的论语须知。本次访谈中受访者是在平等自愿的原则上参与的受访者必须真实的表达自我想法和认知，确认自己符合社保条件。访谈的过程，会被、录音、录音的资料将以匿名的形式用于科研，不会泄露给任何的第三方。

00:32

在访谈的过程中和访谈结束后，你都有权取消研究人员录音、资料的、使用权，你是否知晓并同意？知晓同意。好的，请问一下你现在是一级哪个专业一级出去？在访谈开始之前我我们先说一下我们访谈的目的是什么？访谈的目的就是还原你从国中班宣传到报名到录取，到学习整个过程。所以在整个访谈的过程当中，我们主要是想要知道你你在这个过程中发生的主要的事情，你可以讲你发生的主要事情，也可以讲你的你的情绪，你的感觉或者说思想转变的方方面面都可以聊，尽可能的聊出来比较对你来说比较重大的一些事情或者是想法，主要是好像一个过程大概是这样的。

01:36

轻松一点就是聊天。对我们纯聊天。首先是因为你知道我们这个是关于国中班的一个访谈，所以我们想问的第一个问题就是想问一下中班在宣传那个时候，你对国中班的了解有哪些以及当时是怎么去了解他的？

02:00

那时候是专门搞了一个我不知道是不是只针对技术医学，那时候是相当于开了一个会，专门宣传口中班这个事情，然后那时候可能是在大一七大一要结束的时候，然后我那时候是其实因为之前也学过细节，这些相对临床的课那些有些临床是临床老师来上的，给我们讲过一些临床的事情，本来是想说转专业的，后来上了一年之后感觉其实可以也没有那么强烈的意愿想要去转临床或者转其他的，方向就说还是可能继续留在技术比较好，然后留技术最起码要进实验室。

02:38

我其实是有点这种选择困难症。然后我不？你让我选方向选具体哪个方向我可能不太好选，然后正好国中班出来了。然后甚至这方向我因为都没有了解，也不知道自己喜欢什么，我想先去试试。主要是想说进实验室学习一段时间，如果说对这个方向不感兴趣，我可以再换。然后那时候就听了古筝班会，后来他们创立古筝班的时候，主要是为了培养生殖方向的一些，就像提早给我们定方向。

03:06

我就一我一开始觉得这个还是可以的，并且他们其实给了很多优惠政策都还可以，包括保研的优惠以及每年奖学金这个都是还是可以的，然后基于以上这些情况，然后包括我那时候成绩还可以，我直接报了国中班。

03:23

当时你说你没有很强烈的要转专业的欲望，所以当时教务处当时又出了转专业的规定，政策出来以后你没有报是吗？没你没有报就只报了一个五中班对，只报了一个过程。除了你刚才提到了就是说开了一个会，除了这个会你有没有其他途径了解信息，比如说你有没有和你的同学朋友或者是老师或者是家里面的人去讨论过这些事情呢，或者从网上去看一些相关的信息。

03:58

具体到没有，那时候是倒是在网上查了一下关于果种一一些情况，但是其实查到的也都是比较不是那么具体，对表面的就是他放出来的东西，然后我们也跟同学商量过这个事情。

04:16

然后我那时候是觉得我总归是要选一个方向的，不如就直接趁此机会先进选甚至方向学习一段时间，因为我不对任何方向就是讨厌，也没有那么喜欢。

04:27

这样说先选一个方向，先进一个实验室学习一段时间，因为总归是要进实验室的。当时高中的时候你选是选择的基础医学，还是说调剂的基础医学？我我技术研究院应该是那时候不是好像是我记得是可以填6个志愿，就哪年大后面可以填6个就专业。我前面几个专业都是临床，因为那时候可能还是想去临床技术医学。那时候是真的不知道技术学是干什么。我那时候是真的以为基础医学可能跟临床差不多，可能就什么都要学那种，所以我把技术学院可能排到了第五还是第六，然后慢慢就减分减到就减到技术医学。

05:04

但其实包括说大一前一段时间学习的时候，我还是比较想去转临床的，然后后来慢慢对专业也了解了一部分，了解一份我觉得我可能比较更适合做技术，我就想想说留下来了。

05:17

当时在高中的时候，我想了解先了解一下高中的时候的事情。高中时候你是说填志愿那会儿是一定要确定学医科这个类吗？还是？基本是想要学习课，因为我第一志愿是南大，第二志愿就是苏大的医学院。你当时确定选医科是因为什么事情，或者说自己对什么专业的了解，或者说自己擅长什么之类的，也不是说特别了解，因为一方面高中选修选的是生物可能对生活这方面稍微感兴趣一点，然后再加上那时候包括从初中到高中的时候，初中升高中的时候，就家里人其实也蛮希望我学医的，然后我自己也上网查了一些资料，其实可能也感觉我对医学也蛮感兴趣的，所以就想说就读医科类，大学。

06:06

你父母是比较支持你，对他们是希望我读一个大，因为他们有个朋友，他朋友的儿子就是南大的7年制临床，然后他们也希望我像这样过南医大读过读过医学类，因为我姐姐是老师，我姐姐是老师，然后她就希望我读个一群人，你可以讲再仔细一点，就是你具体你知道你父母为什么特别希望你，也不是特别希望你知道你父母为什么支持你学习，其实主要可能还是因为有同时，对可能还有方面，后来可能医生之后的工作比较稳定，可能一方面想说工作可能不是好找，就稳定一点。

06:45

比较喜欢医生这个工作，你认可他们那个想法吗？我是蛮认可的。也不全全，也不全是因为工作稳定这方面，他们因为他不是朋友，他儿子就是南京大的，其实他们也蛮聊，平时聊天他们聊天也经常聊的是这个事，就觉得哪一大也挺好，就像我也读。

07:08

你觉得像你当时选择一颗是自己喜欢生物原因多一点，还是说家里的支持对你的影响会更大一点？或者说差不多。我的话可能是家里支持多一点，因为我其实比较无所谓。对因为我没有对某个方向特别感兴趣，觉得可能可能他差不多，就可能有些专业我可能确实是不喜欢，我不会填，我跟他们讲过。然后我就说然后正好对医学类我也不讨厌，其实还蛮感兴趣的，我就这样填了。

07:41

好的。

07:42

然后我还有一个第二个比较好奇的一个问题当时你也说了当时高考的时候填志愿填临床比较多嗯，后来慢慢转变了，又决定不转的人也到临床了是发生了什么事情，或者是是大一下学细节的时候，有个临床有个老师他以前是在临床工作当医生的，经常跟我们上课就会讲那些临床的事情，然后讲着可能我就觉得可能我自己不适合去就读当医生做临床这方面的，可能就觉得因为那时候也是逐渐了解了，其实是你讲你觉得不适合还是老师说我自己觉得你听老师讲的一些经历之后，你就觉得自己可能不太适合离开他们对。

08:27

正好又对技术慢慢了解下来，就也大家都知道技术是具体是做什么的，然后我结合自己的性格，然后怎么样就觉得可能我还是更偏向留下来，然后转专业的时候我也跟我爸妈商量过，跟我爸爸妈妈主要还是看我，因为上大学之后，他们主要都是一般在学校这方面还是比较听我的，然后我就跟他们说我的想法，然后他们就说你就不转专业，可以报那个活动办试试看。

08:53

那个老师对你产生的一些影响，你能举一些具体的你觉得或者换一句话，你觉得你不适合医生是哪方面你觉得不适合，或者说你觉得不喜欢结合老师的案例或者也没有不喜欢，我那时候是大一期末的时候不是说不喜欢，可能更偏向于是想要是留着做基础，因为临床一方面压力太大了，包括像我们那一届可能武林那边就可能有14个班级，因为压力太大了，技术这边压力环境相对小一点，然后包括说有他们之前老师给我们讲的一方式，其实我们平时他们讲讲的时候，我们当做笑谈，就觉得很好玩，觉得怎么样，但是我觉得可能可能对于我来说可能不是特别适合具体举个例子我可能也举不出来，就是一种感觉。

09:51

比如说是什么工作时间或者是都有他讲的各方面老师其实上课挺好玩的，就各种讲他领导的事情，那就是让你产生了你不适合这个想法，是他说了什么事情，也没有说具体哪件事，就是一整个学期学下来就给我这种感觉就是这样。压力大对吧？可能压力是一方面，但主要还是可能最，主要是是我觉得这可能我不适合是临床的一个环境。

10:21

因为大一课程可能还比较模糊，然后包括大四之间去实习去医院实习，在医院待了一段时间，可能就觉得可能因为我们接触的比较少，我们科室轮转比较多，我就觉得每天如果说让我做这种重重复性劳动重复性天天敲兵役，天天天天干就重复，一样的事情我可能一方面不喜欢，然后医院的话其实医院其实也蛮复杂的，可能我不是不是特别适合。

10:51

就给我的感觉就是这样，你在考试月的时候你会感到那种比较焦虑的情况，一般就在这种比较高压的环境，就是大一可能会现在不会打一回事，因为现在老老油条了，就。已经熟悉了，对已经习惯了。因为大一那时候不管是主干科还是非主干科，那时候主干科你都会很不是焦虑，也不是压力大，就是会复习就复习。

11:20

总会要有流程，你也不能说一点心理波动都没有。其实那时候是这样，我那时候不能转专业的，不转专业的原因，还有一个是我成绩是在20%~30%之间，我是可以找儿科，这种是转儿科的，但是我对儿科不行，我不喜欢儿科，我就没转临床我是转不了。一方面转不了然后我确实也不感兴趣，大一可能还稍微好一点，然后反正后来越来越了解基础专业，我可能越来越更喜喜欢，基础这方面更多一点。你觉得它吸引你的点在于哪里？

11:52

是说人际关系比较简单，是说我比较喜欢科研这些，我可能比较喜欢科研这方面，因为我进实验室之后也自己拿了课题，我可能个人更偏向喜欢这种探索性的东西，你刚说你拿到了一个课题，拿到课题是什么时候的？事情是大几？大二升大三暑假，因为大二的时候我还在轮转，国中有一个轮转制度，就在国中现在是轮转。然后我大二其实就已经定下来了，大二结束之后我就定在那个了定在我现在老师的实验室了，我跟他说我定下来我要走了。

12:29

我觉得因为城市的氛围我很喜欢，是不是这些人对我都挺好的。然后那个时候正好李老师就给了我一个课题，我就一直在做。所以做下之后我给我的感觉对，刚开始可能是师兄带着我，因为有些时间我不会做，然后带着我，然后后面自己会做的时候都是我一个人在辅导。我可能就更喜欢这种。这个课题刚刚拿到手，一遍空白，我自己在上面涂鸦，想怎么涂怎么涂，当然也是有条理的涂，然后就这样一步一步探索下去，我觉得蛮喜欢这种感觉的，所以有在基础最主要的原因是你的兴趣，对可以这么说。

13:09

你可能大一不是很了解，大一因为也没有进实验室，也没做实验做的也不多，后来是慢慢喜欢技术的。了解深入之后，对大一当时除了家里人是比较支持你自己做决定的对吧？你有没有跟老师讨论过，还是说没有？包括辅导员，包括你的专业课老师，大一没怎么讨论过。没有，大一是没有讨论过的。同学因为那时候国中班刚创业的时候，因为第一页什么都不懂，其实也没有说特地找那个老师问我。

13:44

你觉得之间讨论有什么让你印象比较深刻的事情吗？

13:49

讨论的话好像也没有讨论的不多，因为那时候可能考试月都在复习。我跟我是有讨论过，他们他其实也是想报的，然后但是他后来因为成绩成绩不是不够，就不能报成绩要前50%，对，因为他好像大一有两门课考的比较低的比较低，然后他不能报。

14:14

我大概总结了一下国中班的就几个优势，一个是奖学金，第二个是科研导师。科研导师制就是有比较多和科研导师老师接触的机会。第三个就是特色的教学安排，就是加了一些科研课程，还有特色的建实习。第4个是免研，第5个是5+1+3的本硕博的一个模式。第6个是出国深造，你觉得这6点哪一个对你来说吸引会更大一点？

14:46

你说对我现在吸引最大，还是说当时大一的年。说的话你也记不清了，记不清可以不说。当时信其实是有过进实验室的途径，进行有个进行直接进行时的途径。因为我这样我就只能说自己官网上再搜第一个挑，我觉得挑的话可能我又要纠结半天，就这半天，然后还要去联系导师，不纠结对对对，老师就可以帮你定下来。

15:26

就像不是定下来，他进去之后会给一个导师的名单，导致名单一个双向选择嗯，然后现在我觉得这样可能直接一点，因为我可能那时候觉得可能不同时间是对我影响可能不是对我的感觉可能都差不多，我只想想进实验室学习一下，因为那时候大一的时候辅导员就已经相当于建议我们就提早进行实验室提早进行实验室感受学习。

15:50

但大一没有好的途径，那时候也主要在学习就搞成绩，然后可能就没进实验室。后来就想说有个这么好直接进实验室的机会，我就直接进去了。

16:01

当时大二是科研轮转，然后要4个导师对吧？4个导师轮轮转一下当时选导师的时候你是怎么想的？还是说你觉得导师的课题跟你情绪比较相合，或者说这是一方面，因为他不是有个导师简介，有个导师的研究方向相关，因为我是问这个问题是为什么？因为你一直在说就是说不想去纠结，但是我想你大二到了4个老师那轮转了一下，你还是要从4个老师里面选1个，或者说我说就不纠结，可能稍微不那么麻烦，我其实挺怕麻烦的，但是有些事情再麻烦也要去做。

16:43

然后他毕竟给你相当于给你一个导师名单，总归不会说像技术学院那么大一样，整个选了那么多个老师让我一个跳，不怎么跳不好。

16:54

我技术学院里面挑的话要方向要调，那个方向里面哪些老师厉不厉害，然后实验室氛围好不好，又要调，我觉得这个可能就我自己其实要挑也能挑，就可能比较痛苦。当时你现在导师是当时轮转的4个导师之一，我其实只能转了三个老师我。

17:14

三个老师对我第一轮在那个老师，我最后一轮又回去了。当时为啥要回去了？因为我赚了我前面转了三个老师，我觉得可能都差不多，然后正好第一个老师的氛围，我真的我很喜欢老师人也很好，实验室师兄师姐人也很好，就实验室氛围简要的说一下轮状的内容是什么吗？为什么会让你感觉这三个轮状都差不多？因为其实我们进行那时候进实验室可能主要还是说学实验技术为主，但其实基础实验像pcrs什么这些可能都差不多，所以应该基础的实验方法都差不多，可能不同的是实验室的氛围。

17:50

然后导师的性格，导师的实力其实我们那时候太没有感觉太多，因为那时候可能我们是那时候可以说跟导师接触不是特别多，主要还是以自己一个学习的为主。

18:02

然后导师实力怎么样我不清楚，然后主要给我感觉最深的就是我觉得我现在在实验室氛围特别好，很其乐融融啊。

18:16

所以你在学习的过程当中非常关心就是和别人的互动，就是在自我科研的时候还是差不多，因为我觉得好不好，哪怕说之后读研究生总归要苦三年，为什么不能快乐的苦三年呢？你在我在一个实验室氛围不好的，就天天勾心斗角的，我觉得我自己工作也是工作不好。

18:38

你和实验室的师兄、师姐、或者是、师弟、师妹也会一起出去会餐什么之类的，不会是吧？你进活动班有没有跟父母讨论？讨论过，但是他们也不太懂。他们其实不太懂，还是主要是以我为主，就是开始看我的。所以现在想回到一个刚刚你提到的一个点，你当时说拿到课题这个事情你印象好像还蛮深的，直接说出来了。可以跟我们详细的讲一讲这件事情吗？大二结束。

19:14

那会儿是吗？

19:15

对，当时那会儿我就跟我导师说就提了一句，我就说我就不能这样问一下，就我问他能不能说之后我一直在待在这，然后他其实也同意了，也同意了之后，正好我师兄有一个他手里销售比较多，就课题比较多，就意思是相当于氛围把它这就是还没有还没有开始的课题，相当于让他带着我做先做，因为这个课题其实是怎么讲，这个课题其实之前是有人做，是在我前一个16级的临床学生在他那做的，但那时候做的时候什么都没有，销售都没够，销售都还没构建出来。

19:51

然后他那时候就大三了，他要走了，要去医院实习了，然后就课题就放了。所以我进去之后怎么学生做不出来，不是做不出来，因为还没开始。

20:00

小鼠还没反应出来是是，学生来的时间太短了，就没有足够长的时间去做销售，时间点可能太不太好，因为他来的时候他那时候准备做课题的时候可能已经大三了，因为临床不是要先下点了，对下点他可能就后面也做不了了，那时候小鼠都没出来所以说，他来的时间不对。

20:17

对，然后我去的时候正好小鼠出来了，正好小鼠出来有小鼠可以做了，然后正好把这个课题给我，正好就把这个课题给你，然后你的师兄就带着你去做这个课题，对。当时你拿到这个课题是感觉很兴奋是吧？所以你是蛮兴奋的。因为第一次相当于自己负责一个课题嘛嗯，课题相当于是像那种研究生模式的老师生生下来的课题，然后发给你们。

20:48

可以大概的说一下课题是什么方向的。关于也是生殖医学的对。 Catip基因敲除之后可以发现它金子是不动的，主要是探究一个为什么会导致金子不动。当时你在做的时候有没有遇到什么困难，或者说还是说兴奋大于所面面临的刚开始肯定是兴奋比较多，然后做着肯定不可能一帆风顺，肯定会有。

21:18

我记得最清楚的是免疫工程电实验实验，因为一方面小组刚出来，小组不是很多，可能做的如果能一次成功最好，如果就一次失败的话，可能后面还要再继续等小鼠反应。

21:33

我那时候可能做了一个学期才做出来。

21:35

各种原因，因为那时候刚开始是相当于是我自己做的时候试剂盒前两次是因为自己操作失误了，然后没做出来。不是没做出来，就效果不是特别好，做事做出来效果不是特别好，然后第三后面两三次是后面两三次就莫名其妙做不出来了。

22:00

因为当时试剂盒可能就要的条件比较严谨，可能那时候条件不太对，然后后面又做了两次是实际上我做了这么这么多次不大好，然后他直接自己做，最多用的是敲出书，因为之前是没有用敲出书的，他用敲出书之后还是做不出来。

22:15

因为发现裂解液的问题，各种问题，然后反正后来可能做了一个多星期，我后来又做了换了个方法，就不用自己和换了个方法，然后做出来了做出来之后才继续往后推进。

22:26

其实那时候蛮痛苦的，因为做一次可能要蛮久，这次做一次可能要将近四五天，因为那时候做实验我要挑时间，那时候课又多，我有时候我其实那时候还不是说翘课去做实验，20应该是已经大三了对吧？

22:41

我想。想对。应该是已经大三了。大三那时候因为主要有课，那时候可能大三刚开学课又比较多，会比较多，我必须得挑，比如哪天半天是没课的，然后那天去做实验，然后把时间点安排，然后合理安排下，所以可能我只能一一周只能做一次。所以他一直就后来可能做了将近一个学一个学期。当时做出来以后，还记得是什么心情吗？肯定很开心，总归。

23:14

做出来了，总算做出来，能往后推进。我刚刚是想问哪个写的有点乱。有的同学啊会觉得读了活动班毕竟是走走科研的道路，肯定会遇到像你刚刚说的这种困难，然后像你是做出来了，成功了，但是也有很多可能是做到最后还是依然是失败的。

23:49

你对所以他们有的同学可能会觉得科研失败对他们来说可能代价很大，所以就会有一种畏惧的情绪，你对这种观点怎么看？有的因为确实可能有的是时间做不出来或者课题课题不是特别好，就走到后面走死了，走死路了就走不出来，我觉得。

24:12

你在报名之前的时候有没有想过以后做了一个课题做时代了，那时候没想那么多，那时候没想那么多。

24:25

我觉得现在影响想不用到处纠结选什么方向，我起码可以确定我选什么方向。当时主要的想法是对于以后可能会产生的困难或者是这些其实也没有想到太多。当时还是现在当时没有。现在是有什么转变吗？现在转变肯定是有。

24:54

那时候其实刚进国中班的时候想说，那时候想比较远，最后工作做，什么做？因为基础技术专业还是可能最大的问题说怎么就业？嗯那时候刚进工程班的时候，就觉得老师感觉好厉害，就想说奔一本像学校那些导师一样留校做教授，做教授，然后一样做相当于有人做教授之类的，就教授上跑，然后现在其实觉得感觉他们这种因为自己也了解蛮多，在实验室也跟师兄师姐聊过天，就觉得可能像如果说像老师那样做教授的话，其实感觉不是不能做很困难。

25:40

我觉得是我给我的感觉是现在很对我，现在我来说肯定很困难，因为我现在还在学，因为可能要处理很多方面，一方面基金的事，基金然后课题申报就各种乱七八糟的事情。我觉得可能对于我现在是觉得可能有点应付，如果应付的话可能会很困难，可能如果说之后等我读研究生读博士可能会稍微好一点。所以目前的来目前你来说，你以后是想做现在还没想清楚。现在其实有点纠结，我之前是想说留任做教授做副教授做教授的，所以我觉得这个问题可能还是要等我研究生或者博士生读完之后才会探索，对，还没有完全的想清楚。

26:25

你觉得国中班读下来跟你预期的差不多吗？

26:29

对我来说差不多，因为我本身可能我没有说想着，研究生去外校去或者去青霉素胶，我也想谁谁也不想去好好学校，但我觉得可能我今天跟老师也聊过，他就说那些好学校大牛实验室好的显示可能它自己本身实验室自己学校的本身就已经挑满了，然后我不一定能去，我们不一定能去的话，去了有些不好的实验室或者实验室氛围不好，或者老师比较老师比较不好的，我还不如有哪一大。

26:58

这样因为主要是我最后也就是想留下来，最大一部分原因就是我老师的实验室就对我吸引力特别大。

27:09

给你吸引力大的主要是因为它的范围是对。我觉得导师实验室强不强，可能没有太大影响，还是想待在一个更好的实验室呃。不是就是氛围更好的实验室。其实我觉得你好像对于你来说，实验室的氛围还蛮重要的，包括不仅仅是实验室，你以后假如说在选择工作的时候，可能对于工作氛围这一个观点这个点工作的话没有想那么多，我其实包括氛围不仅仅是就是轻松愉快的环境，包括和人际之间的处理这些。

27:52

你会觉得自己比较看重吗？可能会吧因为还是想达到一个稍微愉悦一点的氛围里面工作，包括你刚刚说做教授可能会有点困难，但是你说的困难的点是这个就是硬实力困难了，所以就涉及到我想再多了解一下你个人刚实验室氛围的问题，我觉得是不是这个问题，我想换换一个问题来问了。

28:26

你觉得对于你来说，大学的这几年，包括本科硕士、博士，你整个学习高等教育的学习生涯的规划来说，你觉得对你来说目前或者说在这几年来，对你来说最重要的一件事情是什么？最重要你这几年来最重大的一个目标是什么？目标是没有那么是可能有的人会觉得成为成为学生会主席比较重要，有的人可能觉得找个女朋友男朋友比较重要，有的人觉得考题比较重要，有的人觉得保研比较重要，每个人觉得事情觉得比较重要的事情不一样。

29:11

你有你有思考过这个问题吗？你觉得你这几年干的这些事情是围绕哪个目标进行的？对你来说最重要的我觉得可能有点虚。主要还是说我本科这段时间想提升自己的相当于还是说你没有考虑过这个问题？是考虑过，因为大一大一肯定是想说大一是想说奔着转专业去，然后后来因为不想专业了嘛，然后那时候就在说要想看能不能保研，保研我不太想考研。

29:40

保研奔着保研这个目标去，但这样工作班之后他就不需要我就不需要那么像平常一样，一定要把绩点刷多少才能保险，就国中的保研环境保研的条件比较松，比较小。

29:57

对压力比较小，然后也没有就保研方面的事也没有想那么多，再加上我自己成绩也其实蛮好的，保研基本没什么问题。我就可能没有太太考虑这个事情，然后可能想的最多的主要是想说在实验室能多学点东西，多提升一下自己的实力。

30:16

你说就是保研成绩积点比较松，你现在成绩大概在什么位置？你说国中班吗？还是在原技术？你们保研是按照基础专业，是因为我们不是有的课，我不是还在基础吗？但是技术有些课是删掉了，就我们跟技术课只算重合的算重合的排名要在基础原专业的前40%，我觉得这个其实已经满足了，我成绩是肯定够的，然后就没有太担心这个事情。

30:45

所以你们现在只要在成绩在前40%就可以保研对。他说是这么说，但是因为第一届不好讲，我们第一届具体怎么搞，那就等等就是今年保研出来之后，对他不是报出去很多了，口头承诺你也就是长个心眼。

31:03

有没有纸质的那种书面的承诺宣传单之类的宣传单这个有。这个应该是应该差不多，虽然是是是这么讲，对。因为毕竟是第一届，他们像因为我为什么问这个问题，因为昨天有个学生其实对他来说，他是比较想去就拿到博士学位的，所以他在刷刷他的课程，他在重修他的课程。

31:30

对，说它的基点。所以我不知道为什么他要刷几点，他的成绩好像也跟你在差不多一个段位上。你们保研是有什么其他政策吗？他是国中班的学生吗？还是是国中班？只要到前40%就可以保研是吗？对。只看成绩不看其他的原本是两条路，你要么再减40%，要么就是你发篇文章发一篇SCI，那你就可以直接报验。但是现在好像不是说国家规定发文章不能保研了吗？然后就把取消了，就看看成绩我觉得其实蛮松的。

32:08

活动其实可能是主要想让其他专业会更严是吗？基础其实是什么？基础保研是百20%。我觉得其实已经蛮松的了，然后我们可能比技术还要重40%。所以其实对于当一当时大一的你来说可能保研的政策还是蛮吸引你的。应该可以这么说。吗嗯。

32:33

因为他也想过这个事，但没有那么形容。因为有可能主要还是想去，因为我自己那时候觉得我大一成绩其实还可以在20%~30%之间。嗯但是相当于把所有的课全部算进去，算的20%~3%的时间算，如果只算主要课的话，我应该还能再靠前。所以我不担心自己承接，所以保研有吸引，但没有那么大。对你没有信任，主要是实力足够，所以不需要太多考虑这些，说狂一点就是这样。

33:09

你你刚刚前面有一个点我没有抓着问，现在我就比较好奇了。你当时说在大一的时候可能会不是说可能不能没有焦虑这么严重，可能会比较紧张一个状态，到了大二大三，现在不会那么紧张了是。

33:32

你觉得最主要的原因是什么？找到了主要是因为那时候刚进大学，对于相当于第一次考试第一次参加期末考总归会有点紧张，反正后面考试也多了，包括期中考期末考都有，其实就慢慢就自己相当于也适应这个环境了。那时候从高中升大学的时候可能还是有点不适应。你已经知道了怎么去学习。对。学习方法的习得，你觉得是你自我习得的比较多，还是老师教出来的比较多？

34:08

因为其实我觉得大学学习可能还是以自己为主，也不像高中老师盯着老师追着你学，所以大学就出来自己学，你想学总会能找到自己的方法，国中班在你刚刚说您有一个印象非常深的事情，拿课题那个事情中间遇到一些困难，在遇到困难的一个学期，结果都出不来的时候，你当时有改变过想法吗？有没有想过不打算再做科研之类的？

34:48

没有，因为其实这个困难我觉得是一个小困难。没有改变是因为这种困难我觉得你当时其实觉得蛮小的，因为这个东西是可以做出来的。只是因为可能自己的原因或者客观原因是没有做出来就能做，但是如果说真的是到那种这个东西做不出来了，这个课题做死了就或者说做不下去了。

35:09

如果说这个课题我刚开始做，做下去了，我觉得可能还好，毕竟没有花那么大时间，如果说可能我做了可能两三年三四年，然后发现做不下去了，可能就确实有点痛苦。我觉得痛苦还是有但是所以，当时你还是觉得这是一个它造成现象的原因还是你可以去改变的，对，就我知道自己哪一步错了，或者说具体因为什么原因他没有做出来，我大致是能够给你带来很强的挫败感嗯。挫败感倒没有，倒是有失落，倒是感觉不强。

35:45

对，因为那时候刚开始做实验，总做失败了，总归会经历有点不是特别好，包括说因为是集合稍微有点贵。我是因为我纠结的有件事，所以管子是配套的，可能用完之后，管子用完之后，整个设计是要重新买了。然后那时候一做了可能不是大概可能有四五次还是五六次，可能管的话比较多，反正我都有点心疼了，然后然后我就说你放心大胆做随便做，就不要说因为这个事情就不敢做之类的。有没有其他让你影响比较深的事情，就整个学习过程中大二开始就这么办，或者说让你比较失落的事情，没有的话可以跳过。

36:28

除了做实验一下子做了一个学期最后做出来了。我没有觉得过的，我也没有说特别兴奋，也没有特别失望那种。

36:40

所以你你觉得你我可不可以就说，你的就是个人的情绪起伏不会有那么大，可以这么说吗？我想问的另外一个问题就是你的还是一个关于你个人的问题，有有两件事情，你觉得哪件事情会给你带来更大的自豪感或者成就感。

37:08

一件事情是你在学习的过程中发现掌握了一项技能，比如说一项科研技术手段，或者说一个实验方法，或者说一一项新的理论知识。另外一个呢是，班级里考了前10%，或者举个例子随便说，就是考到考到了前列突破了，你以往的成绩排名，你觉得这两件事情哪件事情会让你感到更兴奋？或者说第一个对我现在来说是第一个，对你现在来说是第一个，但对于当时的大一可能是第二个你，比方说一一对大一的话刚进大学，可能还是第二个比较。

37:44

第一个因为说实话我现在其实成绩算是蛮靠前，的了蛮靠前，然后因为我平时是不怎么学的，我平时可能学习没有那么没有让他们真的有人去图书馆天天学习，我其实不喜欢学习，我只是纯粹是为了应付考试，所以可能成绩好当然是也蛮开心的，但没有那么没有没有那么让我兴奋，因为毕竟我这几年成绩其实也蛮靠前的。

38:11

你的个人时间的分配是用来干什么？你假如说不是说去看书的话，大二吗？还是大一？你刚刚说你不怎么去图书馆去学习，是从大一到现在都是样子的，还是从某一个时间段开始？好像我们去图案没有花很多的时间去用在理论学习上，因为我发现可能我从大一开始就是这样的，大一可能稍微好一点，有到大二大二之后，可能就考试前我自己定个时间，比如说还下个月就要考试了，我花这一个月时间去学习，我会挑一个时间段集中复习。

38:58

你就是在这一个月之外的话就是去实验室去实验室，就在身上或者自己在宿舍玩或者去运动啊之类的。因为其实那时候我也经常跟他们一起打球跑步之类的，涉及到刚刚你的导师，你和你导师讨论过去什么清北复交之类外校的问题。对。假如说你真的有机会去，你看当然我也不会说说否定为什么你肯定也会去的对吧？大家正常的一个学生肯定都愿意去更好的一个环境，但是假如说我只是提一个假设提你有机会去了清北复交这种比较顶尖的学府，但是我们都知道这种学校周围的强人会更多，你的可能你的成绩可能就是不会像在南医大这么靠前。

39:54

如果说你觉得所有环境都一样的话，包括说如果我举个例子，如果说刘老师实验室现在在北大实验室氛围都是一样，我可能会去北大，你还是会去是吧？因为我其实研究生现在留在哪一大，主要是想留在哪里，刘老师那个实验室不是说想留在哪一大，所以像成绩排名这些事情会给给你带来挫败感，其实没有那么强对。因为只要说不影响我，就如那么就只，如果说不影响我之后保研，我考100和我考60对我来说没区别。

40:25

结果对你来说是对，只要不影响结果。在读国中班之前你有没有让你觉得最自豪或最有成就感的事情，就是刚刚你提到的做了一个学期的实验做成功，那个算是你从大二开始到现在你觉得最有成就感的一件事情吗？实际上但是是有件事，其实前段时间但是不是活动班，就是公办。也可以，你说吗？数据计划大创。不是有个答辩下台答辩，然后我是用我就之前做的就就这个课题去答辩的。

41:13

我们刚刚对和答辩自己做的PPT，我相当于说这个东西从头到尾都是我一个人在做，虽然说我们组有其他几个人，因为其他几个人也是我们显示就就、本科生，但他们因为要么刚来，要么就是在外面实习就没有做什么事，但我还是把他们拉一起，因为这个课题可能之后想要说一起负责。

41:34

这些本科生是你国中班的同学还是原来技术的同学，有一个是公证班同学，有的是原基础的，然后那时候就从到位我一个人做完之后去那边答辩之后，那时候拿的是第一全部都是你一个人做吗？

41:48

可以。这么说。因为之前前段时间他们因为各有各的事情还没开始做，然后这个课题一直都是我一个人在负责。然后那时候打第一之后其实还是蛮高兴的。高兴在哪？因为通过了是高兴还是拿第一？拿了第一，就是一辩拿了第一，所以你会选择经常来参与这种课余的科研的兴趣活动是吗？没有参加过就是类似这种比赛，对比赛参加了很多是吗？

42:25

也不是很多，我其实参加的不是很多，有些比赛我其实都没有什么会突然想到参加大创的。我是大二的时候参加过挑战杯，就类似的挑战杯，然后我也跟着我导师聊过这个事，他问我今天要不要参加。

42:40

然后我说如果想参加的话，我可以去参加。是因为对于我来说，在他们参加这个比赛没有那么大的吸引力，对我来说可能参加也好，不参加也好，没有太大区别。但如果参加的话，我肯定会想好好搞，拿个第一就是拿个往后的名字奔。然后正好那时候刘老师就说今天去参加，然后用我就之前做的课题，就相当于不用再重新开一个课题了。就一方面不耽不耽误我自己的时间，一方面也能相当于让我把整个课题理一遍。

43:11

然后还有一个就是答辩的，相当于让我多一次答辩的机会。因为之后如果读研究生读，我是答辩总会少不了，然后让我提前体验一下，所以其实还是导师促使你参加了这一次有一部分对有一部分还有一部分是什么？今天我听你的讲话，感觉你好像个人对于参加这个好像是可有可无的一个，其实我就是觉得可有可无，但是如果说参加的话，我肯定会想好好搞。

43:36

因为我大二参加我不是团队，主要是负责人，我相当于只是一个参与者，但是这个是相当于第一次。你第一次这种我团队负责人所有事情我当然那那个那个项目你参加了，中间的环节参加了，但是对因为我只是一个参与者，不是团队负责人。你有参加过学生会或者是社团之类的。参加过。是我参加了哪些社团？社团有学生会，其实教学不算学生会算学生组织。

44:14

今天俊豪我之前参加的是是大一时候参加的吗？大原来就是我对，那时候初中班的时候，国中班的时候，因为那时候是这样，我大一不是没有参加学生会，没有参加学生组织，然后大家经常说我那时候觉得上过大学，一个学生组织不参加有点不太好，一方面我想体验一下学生组织的生活，就做事情那种氛围。

44:41

然后我就好像是具体哪个阶段我记不清了，反正进了就进了群行。

44:48

可能有一个学期，因为一个学期感受一下，我就纯粹感受一下我就出来了，感觉怎么样？挺好的，其实那个挺好的，如果说我从大一开始说过吗？有总归是有，我如果从大一进去的话，我觉得应该能做得更好，但是大二我可能主要重心是平台，好像是那种类似于可能跟普通人差不多活动之类的，是这个东西吗？可能跟波音差不多。福音就跟学生的服务社对。大二可能我主要就是去体验一下，体验完了出来也没有再去做。

45:25

所以我觉得我可不可以说你是比较喜欢去给自己创造一个挑战的或者接受挑战的一个人，你觉得你是这种人吗？我觉得应该是。我不太想那种一成不变的生活就稍微有点挑战性挺好的。但是科研在整个过程当中，啊是其实是要耐得住寂寞，要一个人去做，你不觉得和你的个性会有一点点不符吗？你觉得这一点你怎么看？我觉得不会，不服啊不会。因为其实我听你的讲话的感觉是讲的事情的感觉，你还是蛮喜欢做不不一样的事情，去体验不一样的事情，也喜欢给自己带来一些挑战。

46:09

但是你在整个做科研的过程当中，我们不谈结果你也说了，做出来会有好感情感，但整个过程其实很枯燥，对包括说一个可能要做两三次做三四次，你不会觉得这会给你带来很大的挑战吗？就是给你的个性不是对是有一点。

46:26

我天天做p加，我肯定是不喜欢的，但是我觉得如果说我天天比如我我这两三天天天做，能给我后面带来带来好处，或者说后面的时间带来推进的作用，我觉得这个是可以的，只要虽然这个过程你可能不是很喜欢，但只要能达到你最后的目标，还是会去愿意不愿意去做。因为但是你如果说单纯的让我说我整个研究生我只做PC，我肯定我肯定不喜欢。

46:59

明白。

47:07

了，所以当时就参加这些俊航这些东西。也就是想体验一下不同的生活，对，因为大二的时候特别的对，大二可能主要侧重还是放在国中放科研这方面。你觉得你参加的这些活动，像大树青年这种给你带来最大的就是收获和感受是什么？

47:30

如果你觉得没有，你说没有还是有，因为包括说这次大创的话，我相当于第一次上台，也不是第一次，在那么多人面前上的去汇报去汇报去答辩，也经历其实还是蛮好的，然后包括锻炼我胆量，包括答辩技巧这方面其实还都挺好的，包括俊航的话其实多一些东西，咱们把工作分布下来，然后自己做东西这种。然后最后如果说自己能看到你做的东西能有作用，其实也还是蛮好的。

48:04

你挂过科吗？没有。挂科不就不能报过重了吗？这样对。二三大二大三挂科了就要退出吗？那不是退出就是不能保研。学校都是这样子，所有的领导很喜欢给自己给自己加上就是给自己很多挑战，这样说给自己带来很多挑战，你会害怕挑战后，假如说失败了，你有想过事情吗？

48:37

比如说啊大创，你自己主动负责的一个大创项目，有没有想过大创项目后面失败了怎么办？你有考虑过这个问题？我想过我那时候想到可能是就像如果说我上去打那边可能拿的名次不是特别好，失落总归会有，但我觉得我觉得拿我我那时候觉得能拿多少名字不重要，我主要是想说自己上来去落一下，锻炼一下自己。

49:03

可以跟我们说说你当时准备你负责大创的项目的一个大致的过程。答辩前一个星期我天天在那改PPT，天天在那念稿子，因为很紧张，我其实很紧张。是蛮紧张，因为第一次相当于上台汇报吗？可能有点不习惯，原先你们之前做科研做组，比如说组开组会的时候不都是要给我们小汇报，我们实验室可能不太一样，我们实验室之前组会是相当于就工作汇报的话跟组会是我们是分开的。

49:35

工作汇报我们相当于自己跟老师聊天，就相当于我跟你现在这样就把我汇报了，我做了什么东西我跟你讲一遍，然后把图给你看一遍，然后老师跟我讲之后怎么做，然后包括怎么改进之类的组会。

49:46

只会主要是研究生去讲讲文献之类的，就不会说让我不是说让我去讲你们去听你参加吗？我参加，但是主要是听所以他之前像其实你没有什么参加过这种公开演讲的这种对少了。

50:01

其实你觉得你当时紧张的来源是来源哪里？还没有公开演讲过？对。其实并不是我我可不可以说并不是来源于对于失败的恐惧，还是不能上台演讲，主要是第一次这个事情比较紧张，因为那时候其实我拿多少名次对我不重要。因为我也不指望说靠一就进行审菜，进行消菜对我来说其实无所谓。所以主要是去想锻炼一下，对。但是能拿好名次最好，拿不了好名次其实也还好，就没有太多功利的心态。

50:47

下面一个问题是关于可能还是对于大二的时候，我估计是大二的时候就是关于你已经进了博士班以后，我当时有没有到了第二年的时候啊？有没有什么学弟学妹来问你？

51:04

关于国内办的事情有人来咨询过你，有。你当时他们问的是什么问题，我们了解其实主要也有好几届问过，我觉得直接都问过，对，包括18级19级可能都有人问过我。可能对他们找到你各种方法，因为他们有时候在学生会里，因为之前有一个学生说他大二的时候学弟学妹都会来找我，毕竟是下一届的，到大三了就没有人来找我了。

51:31

大三其实是有一个，因为大三可能就去找19对找找18级的了，所以我很好奇怎么会找到你的。是他们有的在学生会有，然后有他们学生会，因为我们我们大三的时候，我们这一届的人还在学生会里面吗？有人还在学生会里面，然后我正好跟他们几个人有的人玩得好，然后他们就会你是跟学生会那些人玩的好。对，因为平时可能我人际关系其实还可以，可能跟谁都玩的差不多，玩的挺好的，他们就推荐你，对，因为他们可能也问我，就说我想包括公众班，有没有人有就想找人问一下，然后可能就把我推过去了。

52:06

他没来问什么类型的问题？其实主要还是我觉得最多的他们觉得进国资办会不会说6走窄了，就觉得我这么早定一个方向，我最后就不能搞别的了。

52:17

你觉得会有这种我，其实是觉得真不至于考虑这个问题，因为其实说实话，你最后读研究生读博士，你总归是研究一个方向的点，你也不可能说你把整个方向研究透彻你不现实，都是研究一个点，我觉得不存在说方向这个方向窄不窄，这个方向小不小，我觉得其实没多少影响，纯粹是一个方向问题，你对深圳感兴趣了，你怎么做都能做，你对深圳不感兴趣了，你肯定不想来。

52:45

你觉得在来之前要确定一下自己的兴趣的一个范围？

52:50

也不是说兴趣，因为我其实觉得大一升大二的时候，就那时候报课程班的时候，应该没有多少人对科研感兴趣，甚至没有人多少人对升值感兴趣，可能主要是先进来体验，如果我个人觉得你进来之后你觉得自己不讨厌，你就可以继续做下去。因为其实对我现在来说就是这样，我我不敢说我喜欢升级这个方向，但我知道我能做下去，我不讨厌这个方向，我就可以继续做下去。

53:17

所以像学基础医学学了这么久，我可以说是你其实对于任何一个研究方向都没有到讨厌或者说无法接受的程度，但是也没有对任何一个方向有一个这是浓烈的一个兴趣的地步嗯，对，其实对于我来说是这样，我可能你给我啥我都能做。

53:36

我觉得是这样，我接触的多了，我自然也不是说喜欢，就相当于可能会更偏向，因为我懂得比较多了，我更加了解，我对这方面更加了解，我可能就觉得我能做下去，只要我不讨厌，在我不讨厌的前提下我就能继续做下去，但如果说有的方向可能我做如果做了一会之后，我觉得我确实不喜欢，那我就不是特别想做了，不喜欢的点会来源于哪里？

54:00

是实验方法太复杂了，还是说你们研究？当然现在我也不太清楚，因为我没有碰到，我没有去各个方向我这样过，对。那么就好比跟高考填志愿一样，有的专业我确实不喜欢。对，所以我刚刚就想问你，你现在就是方向你讲出来，讲不出来讨厌在哪里，高中那个时候跟你说你父母讲了，有一些你不喜欢的是哪一些是本科，一方面是本科的，一方面可能有些像时候不想报哪个学校来着。

54:39

不是学校主要是大方向。大方向可能我不是特别喜欢像我同学去的，因为我们之前报报志愿的时候经常会一起商量，有的同学去报食品类，但我可能我对视频也不是特别感兴趣，因为可能是因为我了解的比较少，所以不感兴趣。

55:00

有可能是因为我本来就比较不感兴趣，这个我也不知道。听你讲了这么多，我感觉你你这个人是一个能力很强，啥都能接的下来，也很喜欢给自己带来挑战的一个人。有没有什么有没有什么事情？所以我听你讲的时候就比较好奇，有没有什么事情是可以给你引起来，给你引起你极大兴趣的？你最大的一个爱好，我想听一听。兴趣你除了做实验，这是或者说学习也不是说那么的波澜不惊，有些事情确实会让我很高兴，但也不是说高兴的那种，要发疯的那种就也少。

55:39

我想想或者说换一种说法，就是你平时课余时间分配时间最多，如果说真的要感兴趣的话，我就想说5+1+3能真正读下去，能真正读5+1+3，就按照这条路走下去。

55:54

因为现在之后能不能走还不好说，如果能走的话当然挺相当于挺让我很兴奋的，对，因为毕竟能少读好几年了。主要是因为它可以让你少读好几个人，主要是吧？因为你正常读的话6年，然后如果5+1+3的话可能就直接减少两年。因为相当于我其实本科精神性的蛮多，相当于把本科当做研究生来用了。减少学习的年限是为了什么呢。我中午怎么安排的我也不太清楚，我觉得他们可能就是想说本科阶段就让我们相当于当研究生来用，相当于然后最后研究生可能给我们减少两年，但是学生肯定是都乐意往5+1+3上面走，但老师可能不太乐意老师可能不是特别喜欢，为什么老师不喜欢？

56:43

因为你想再多锻炼几年，不是都锻炼起来，就相当于少人没确认给你干活了，你自己培养一个学生5+1+3，是有限额的吗？还是说，我看他的表述好像是对他的，成绩达标都可以。我感觉不好处理，审计达标都可以，应该是曾经达20%还是多少，我有点忘了，40%百分之那个是保研可以，保研他好像是研究生。

57:15

对16年研一的时候，他有个类似于答辩。你答辩过了你就能5+1+3。答辩不过你就正常3加三答辩是啥？内容啊？可能类似于开题报告，开题就相当于自己做的东西。所以当时学弟学妹问你，会不会方向变窄？你说你你你的个人观点还是会觉得你来了可以试一下，你要真的不喜欢，你也没必要来报。主要还是看自己的兴趣方向是否符合。但我估计不一定也有可能大家觉得问这种问题的学生一般情况下来说，可能他也不知道自己的心理，对，主要可能因为大一可能都不知道自己心里有问题，所以我跟他说是因为反正国中是允许退的，我说你知道平球先进来报，所以给这些人的建议还是说给他们让他们来试试看。

58:14

对，你可以先进来看看。先试试看。包括说你学学了一年，学了一年之后你再退出去，因为没有什么影响，退回一年你到时候就算补的和补的也不多。还有什么其他的人印象比较深的问题，就是李雪梅来问你来咨询你。

58:34

就一个方向面展示，其实主要还有差不多也是相当于国中不是会上课吗？像神经生物像什么卫生学就给生物学，还有生物学，这个是加进去的课，然后就删了几门课，然后他们可能就会说我上了这几门课相当于会不会亏了。但是亏了是什么意思？就是说。

58:56

对差不多是这一条，对差不多是这意思，但是我个人觉得国中山的那几门课，你说句不好听的，你最后到大四了，没多少人会上课认真听，你最后是到大三大四都是老油条，上课基本都不行，就大三的课时，大三大四的那那，大二其实也大二上的不多，大二没怎么上过。主要是大三大四的课。

59:18

现在因为改了，现在因为18级跟我们不太一样，18级其实课改改了蛮多的，反正都差不多可能上了我们上的课他们基本可能也上了，像什么神奇生物，像卫生学这种，他上的这些课其实你都没上过对吧？我对我是没上过，但你还是了解。因为我舍友时尚的呀啊，所以他们上课都不听，他们就觉得没什么用，就觉得纯粹是为了应付考试。所以我觉得应该不存在这个担忧，但是他们可能还是觉得说算了对我自己有点影响。没什么用，是指对以后工作没什么用，所以以后的科研没什么用。

59:55

其实说句不好听的，选内外妇儿也没太大用。对我们来说我们这个专业因为我们毕竟不上临床，但是学总还是说你学总可能学到东西，像卫生学生他上那节课我因为没上，但其实我是觉得那些课你再有用，你过个两年你也会忘，你一直不用的话，你过两年你也会忘。

01:00:14

你要是在这个方向里面，你天天用到，比如我在生理期，我天天用到生理学这本书，我可能几十年都不会忘，所以我觉得这我个人觉得这个问题就不存在什么，所以学弟学妹提出来这个质疑，你也觉得里面不存在对，反正至少我也跟他们讲过，然后他们自己怎么想我就不知道。

01:00:35

那么可能还是会有些有担心说上课会不会有什么影响？你你你觉得从你的角度来出发，你或者说从你的感受来出发，他们觉得影响会影响是哪一方面？是觉得找工作不好找，还是因为是大一的时候？大一的时候刚进大学。

01:00:54

对都不懂就觉得我少上了课感觉亏了，对，因为我其实那时候刚进国中大二刚开始的时候，我们不是说要上课，刚开始是想说把那样话全删掉。我其实那时候我也觉得我也不乐意，我说我那边妇儿全上了，我不是少学好多东西吗？但其实后来那块听可能有时候也听，但是没有那么夸张，我上学期考上学期考的第二我可能就已经不记得了。

01:01:18

自己当时觉得为什么要把那块儿全删掉，就大一大二的时候可能会觉得你觉得对于你就谈你自己对于你来说，你觉得上课给你带来的这种焦虑或者说不乐意是来源于哪里？

01:01:33

那个时候焦虑是因为不是焦虑，就是或者说不高兴就觉得我少见很多东西，少学很多东西，因为我不想说变成一个纯科研的机器人，我还是想想去接受新东西的，但是从我现在看的话，没太大区别。因为反正我上学期考的我这一期我也忘了。

01:01:56

当时你不想成为一个纯科研的机器人，就是不想学的全部都是科研的这些也不是这么说。大一大二的时候可能还想更偏向于想多学点东西，不管说可能多见到一些知识，你说的纯科研的机器人是你当时你，这话其实可能说的不太对。

01:02:17

没关系，这是你当时真实的想法吗？我想问的是你当时觉得去过中班之后，做科研这方面是纯做科研的技术也不是这么讲，因为那时候上的确实有点多了。因为那时候上了之后觉得是不是太少，我觉得有点不大好，就是说能不能再加回来。然后后来确实加回来了，现在其实我觉得就做科研，我现在到不了，他把课全删了，我这样的时间还能多一天。

01:02:45

你发生这样的转变是怎么发生这样的转变？就是一老一个挑战，没有什么特别的事，就是上课上多了总归对吧？我我不是那种天天上课理解，就是你对这方面的认知。增加了对活动方案各方面的，对，因为毕竟大四了，了解东西也多了。然后你比较明确就是自己想要学什么。对，就我知道自己就这个阶段该学什么哪些东西确实学了没什么用，我自己心里也有数。

01:03:17

然后你就觉得对自己的时间分配规划，对我觉得时间分配其实蛮好的。这个事情假如说和你的长远目标没有太大的关系的话，你就是会选择不去选择尽量的不去做这件事情，也不是不去做，可能没有不不会花那么多时间在这上面。

01:03:38

因为包括说因为有考试嘛你学总归还是要学的，但是你就像假如说我们说的绝对一点，某一门课一点都没有用，假如说把它删掉的话，你还是会比较开心的。你说什么时候是现在你都要-23了，我都无所谓，你要-23了我都挺乐意的，不是说一点用都没有，可能。

01:04:05

对于现阶段他来说可能是就做实验，举个例子，比如说临床，他觉得那幅画肯定是有用的，他最后上临床那幅画可能都要用到，但是可能我之后我不去医院，我另外付二工撑死就是我家里人生病了问我我哪里不舒服怎么用我翻翻掉，或者说我可能能回答他一下，有可能作用不是那么大，没有什么实际性的作用嗯。

01:04:28

对。但学总还是要学的，你毕竟毕竟有这门课这个作用主要是为你你是觉得是为你的工作将来的工作服务服务，就这个作用你做客吗？你觉得没有必要。嗯唉其实我现在觉得可能除了生理生活这些比较基础的课，可能稍微因为确实之后科研部相当于科研方面会用到，包括说生活中也基本可能会用到，但是其实相对来说肯定要发生生意，不是生活中其实也能用到就我。

01:05:06

爸妈我亲戚朋友哪里不是问问我，我发完书也能给他答案，他就感觉没有那么就相当于对临床的需求跟临床相比的话，需求度没有他们那么高。

01:05:18

你觉得读国中班来说，国中班这段期间最大的收获是什么呢？没有特别的感觉的话也可以不说，没有，因为其实没有特别大的。举个例子，如果说我没进过这班，我只是单纯的基础医学的，我也同样选择刘老师的实验室，我就没太大区别。但是你不是像一开始说的，一开始说你说你不太喜欢，就是纯粹跳过来那个过程，嘛就跳过来对。我这就不用再说。

01:05:51

可能国中班让你选择上更方便了一点，就是包括说后面论收获的话好像也没有。你在哪都能，因为我其实觉得你你如果想学的话，你在哪都能学到，不会说因为这一个班级你就多学东西，少学东西，你想学总归能学。

01:06:09

果树班期间有没有让你不高兴或者不满的事情啊？对我来说感压力的事情，对我来说没有。有实习的问题。实习因为我们去师傅又实习太远了，太远了就天天来回跑。你们现在应该也在实习结束，五一前结束了。因为今天来回跑又没有校车，又不让我们坐在舞台，可能比较痛苦，因为早上6点多就得起来，在医院干活。

01:06:36

也看科室了，有的科室放的早，有的科室放的晚的话一般5点。周一到周六吗？周一到周五。到师傅要不要实习？多久我们三个半月。三个半月每天到6点起来，对差不多就从江陵这边坐地铁过去，对新街口那边。确实蛮痛苦的。因为是因为基因为基础的实习，基本专业他们在哪实习？江宁或义父。江宁医院是吧？对，就算江宁医院其实也不远，那为啥非要让你们去吃vivo？

01:07:12

我们也想问这个问题，他可能他觉得更贴近升值，但其实对我们来说没太大，不是作用肯定还是有了解医院的情况，我觉得这个可能还是有，但只是我不用不用说别人，就说你自己个人的感觉作用肯定还是有，但主要是真的被以前上课的时候天天想着实习，我去医院玩多好，就不用上课多舒服，现在是实习的时候天天想着上课，上课多少，主要还是来源于通勤的路程，这在整个上班的过程基本上还是不算累。

01:07:51

因为我们实习主要以看为主。看对，因为我看我们毕竟不是临床，他不可能说像临床一样，让我们去这样去看病人之类的主要还是一个观摩。所以其实算我觉得不能算叫倍感压力，应该叫让你不太满意的一件事情。对，通行时间太长了，对。因为那时候他们是这么想的，火中是这么想的。不然我们班宿舍就是想着晚上回来也能就对去实验室，我最开始也是这么想的，说我天天可能6:00到学校认可这个数，我最开始是认可，但是后来发现太累了，太累了，我天天6点回来跟死要死了一样，直接回宿舍睡觉去了。

01:08:40

因为那段时期那段时间基本来说不会去，实验室基本上都没去，那你不会觉得这样对你来说代价太大了，占用了太多时间。

01:08:50

不会，因为实习总归总归因为总归要实习的，可能我只是唯一的一个部分。对总只是说可能路上时间有点久而已。你说今天下班去新街口那边这些东西也还可以。倍感压力的事情好像对你来说应该也不存在压力。可能压力最大的就是大创答辩准备那个时候。可以这么说，要么就是最后的5+155+1+3名额，就是六年级的可能会对。

01:09:28

还有刚刚可能我问着就问问走掉了，学弟学妹除了就那两个问题，应该没有了。一个是关于方向，研究方向主要的课程。主要就这两个问题是吗？对，主要就是这些，其他的就很少。因为其他的1/3也不至于说一定要问我就其实自己也大致都能了解。对，他们可以通过别的途径来了解。其他的我感觉国中班的学习肯定也是达到你预期的。毕竟你觉得和在不在国中班对你来说是差不多的，对，所以你还是比较认可，包括说后面的5+13+3，其实对我的优对我的优惠优惠力度还是蛮大的。

01:10:25

所以其实我可不可以说像我刚刚列了这6点，你其实刚一开始的时候没有没有很明确的选出来，我可不可以说5+1+3对你来说吸引力是算最大的一个，你现在是对，因为当时没有了解，没有很多的了解，可能也是一片空白只是说。来了以后比较方便嗯，对。所以你是比较强的一个读博的动机，的对我肯定要读博。读博的话，你有跟家里人同学或者老师商量过这个事情，我爸妈他们也知道，他们也了解过这个专业，说这个专业必须得读博，如果说之后要就业的话，最后就是赌博。

01:11:02

你妈妈说这个专业必须要读博，所以你觉得你读博士因为专业的必须性要多，还是说你觉得我想要科研才赌博，你觉得哪一个占的比重更大一点？

01:11:17

或者说都一样的一半。

01:11:37

你跟你父母讨论的时候，就是讨论读博的这个事情的时候，或者说换一个说法吧，就是我想知道你父母会不会给你从高考填志愿开始，包括后面的每一步走路，他们会给你建议或者是意见吗？

01:11:54

不会，除了高考填志愿之后，他们基本上还是听你的。

01:11:58

对，因为他们其实也不太懂，因为我其实到学校之后，很多事情他们其实不太懂，可能还是听我，因为我这可能有些包括像你也比较愿意跟他们讲。

01:12:09

对就讲刚开始会讲就让他们了解一下之后，决定权还是在我。但是像我爸妈他们可能有时候平时上网看看资料，上网查查点东西也会讲跟我商量，但主要还是看我的想法。

01:12:30

有一些人的读博的一个观点或者说一种想法，就是觉得读博会给他们带来一种社会地位的上升或者是一种荣耀感，你觉得对于你来说会有这种感觉存在？

01:12:54

对于你来说可能更多的还是一个科研方法的细则，包括以后一个工作的一个这两个角度的考虑。其他应该也没有了。

01:13:05

然后我现在想不到别的。

01:13:07

没有，那就没有啊还有问题，我没有什么问题。

01:13:12

好，很感谢你配合我们做访谈。

01:13:16

我把你把停一下。

01:13:19

我去拿一下这个材料。

01:13:22

谢谢你。

01:13:37

这个是你们相当于搞的一个活动吗？还是什么？

01:13:43

不是活动，是我们的一个质性研究，主要是研究国中班同学们对专业选择这方面的一些影响因素。
