# Supplementary material for: Why do you choose this program?—A decision-making model of medical students based on grounded theory
Source: PLoS One. 2023 Sep 15;18(9):e0291634. doi: 10.1371/journal.pone.0291634 (PMC10503722; doi:10.1371/journal.pone.0291634)
Supplement: S1 File — (ZIP) [file pone.0291634.s001.zip › RAW DATA/P3.docx]

male

00:00

Okay, you can get started. Well, it's the beginning. Hello, we are researchers at the Institute of Medical Education. Before the interview begins, I would like to tell you about the Analects of Confucius on experimental ethics. In this interview, the respondents who participated in the principle of equal and voluntary respondents must truly express their own ideas and cognition, and confirm that they meet the social security conditions. The interview process, recorded, recorded and recorded data will be used in anonymous form for scientific research and will not be disclosed to any third party.

00:32

During and after the interview, you have the right to cancel the recording, the right to use the data, and by the researchers. Do you know and agree? Know the consent. Ok, excuse me, you are now a level which major a level to go out? Before the interview begins, let's talk about the purpose of our interview? The purpose of the interview is to restore the whole process from national middle class publicity to registration to admission, to learning. So in the process of the whole interview, we mainly want to know you you happen in the process, you can tell the main things you happened, also can talk about your emotions, your feelings or all aspects of thought change can chat, as much as possible chat out more important to you some things or ideas, it is mainly like a process is probably like this.

01:36

A little easier is to chat. Just chat to us. First of all, because you know that this is an interview about the Chinese middle class, so the first question we want to ask is ask what did you know about the Chinese middle class at that time and how did you know him at that time?

02:00

It was one, and I don't know if it was just about technical medicine, It was the equivalent of a meeting, To promote the middle-class story, Then it might be at the end of the year, And then I was actually because I had learned the details before, These relatively clinical classes Some clinical classes are attended by clinical teachers, Tell us something about the clinical stuff, I wanted to change my major, Later, after a year, I felt that I didn't have a strong desire to go to the clinic or another, Direction is still likely to stay in the technology is better, And then leave the technology or at least go into the lab.

02:38

I'm actually a bit of this choice problem. Then I don't? You asked me to choose the direction to choose which direction I may not choose, and then the middle class came out. Then even because I didn't know this direction, I didn't know what I like, I want to try first.I mainly want to study in the laboratory for a period of time. If I am not interested in this direction, I can change it again. Then at that time, I listened to the guzheng class meeting. Later, when they founded the guzheng class, it was mainly to cultivate some reproductive direction, just like setting the direction for us in advance.

03:06

At the beginning, I thought this was ok, and they actually gave a lot of preferential policies, including the preferential treatment of postgraduate insurance and the annual scholarship. Then, based on the above conditions, including my grades at that time, I directly applied for the national middle class.

03:23

At that time, you said that you did not have a strong desire to change your major, so the Academic Affairs Office then issued a major change regulation. Did you not report after the policy came out? No you did not report, only reported a five middle class right, only reported a process. In addition to what you mentioned just now is having a meeting, do you have other ways to learn about this meeting, such as whether you have ever discussed these things with your classmates, friends or teachers or people at home, or read some relevant information from the Internet.

03:58

Specific to no, at that time is on the Internet to check about the fruit of some situation, but in fact, found are more not so specific, the surface is he put out of the things, and then we also discussed this matter with the students.

04:16

Then I thought at that time that I always wanted to choose a direction, so it was better to directly take this opportunity to choose an advanced direction or even study for a period of time, because I just hate any direction, and I do not like it so much.

04:27

So first choose a direction, advanced a laboratory to study for a period of time, because it is always to enter the laboratory. When you were in high school, did you choose basic medicine or dispensing basic medicine? I my technology research institute should be at that time is not as if I remember is to be to fill in 6 volunteers, after which year big can fill in 6 major. My previous majors are clinical, because at that time may still want to go to clinical technical medicine. At that time, I really didn't know what technology was about. At that time, I really thought that basic medicine might be similar to the clinical practice, and I might want to learn anything, so I probably ranked the technical college in the fifth or sixth place, and then slowly reduced the points to the technical medicine.

05:04

But in fact, when I studied in my freshman year, I still wanted to go to clinical practice. Later, I gradually learned some parts of my major. I thought I might be more suitable for technology, so I just think about staying.

05:17

When I was in high school, I wanted to know about things in high school first. When you were in high school, did you mean you were sure to study medicine?still? Basically, I want to learn, because my first choice is NTU University, and my second choice is the medical school of Jiangsu University. You were sure to choose medical because of what things, or their understanding of what professional, or what they are good at, also not to understand, because on the one hand, high school elective choose biology may be a little interested in life, and then then including from junior high school to high school, junior high school, the family actually pretty hope I study medicine, and then I also checked some information, actually may also feel I am interested in medicine, so I want to say that medical class, university.

06:06

Your parents are more supportive of you, To them is to wish I read a big, Because they have a friend, His friend's son is a seven-year NTU clinic, And then they wish I went to the University and read medicine, Because my sister is a teacher, My sister is a teacher, Then she wanted me to read a bunch of people, You can speak a little more carefully, You know, you know why your parents really want you, Nor do you particularly wish you to know why your parents support your studies, In fact, mainly because of the same time, For the other possible aspects, Later maybe the doctor was more stable, Maybe on the one hand, you want to say that a job may not be easy to find, Just be stable.

06:45

I like the job of a doctor. Do you recognize their idea? I quite agree with it.It is not complete, not all because of the stable job, they because he is not a friend, his son is Nanjing big, in fact, they also pretty chat, usually chat they often chat about this matter, they think which big is also very good, just like I also read.

07:08

Do you think you chose one for more biological reasons, or do your family support have a little more impact on you? Or something that is similar. My words may be a little more family support, because I actually don't really care. Because I wasn't particularly interested in a certain direction, I think maybe he may be about the same, maybe I may really don't like some majors, I won't fill in, I told them. Then I said and then just to the medical class I also don't hate, actually quite interested in, I just filled in.

07:41

fine.

07:42

Then I have a second more curious question when you also said that when the college entrance examination, fill in the clinical more um, Then it slowly changed, The person who decided not to turn around is what happened to the clinic, Or when it's a big time of detail, There was a clinical teacher who used to work as a clinical doctor, Always tell us the lessons about those clinical things, And then maybe I think maybe I'm not fit to be a doctor, Maybe I feel that because at that time is also gradually to understand, In fact, after you said you think it was inappropriate or the teacher said I think you listened to the teacher, You just feel like you might not be very fit to leave them right.

08:27

Just slowly understand the technology slowly, also everyone know that technology is specific is what to do, and then I combined with their own character, and then how think maybe I still more inclined to stay, and then turn professional I also discussed with my parents, with my parents mainly or see me, because after college, they are mainly generally in school or more listen to me, and then I tell them my idea, and then they said you don't turn professional, can report the activity to try.

08:53

Some influence that that teacher has had on you, Can you cite something specific that you think, or in another sentence, What do you think you are unsuitable for a doctor do you feel unsuitable, Or if you don't like the teacher's case or don't, I was late in my freshman year and I never said I didn't like it, Maybe more inclined, so you want to keep the foundation, Because the clinical one hand is too stressful, There may be 14 classes like our class, Because the pressure is far too high, The pressure environment on the technology side is relatively small, And then including saying a way their teacher told us before, In fact, when we usually say what they talk about it, We think as jokes, Just think it's really fun, How do you think of that? But I think it may not be particularly suitable for me and I may not give it an example, It's just a feeling.

09:51

Such as what working time or have he said all aspects of the teacher actually class is fun, about all kinds of things, that is to let you have you are not suitable for the idea, is what he said, also did not say which specific thing, is a whole semester to learn to give me this feeling is like this. The pressure is right, right? Maybe stress is one thing, but mainly or probably the most, mainly is that I think maybe I am not suitable for a clinical environment.

10:21

Because freshman course may also be more fuzzy, and then include senior between internship to hospital internship, stayed for a period of time in the hospital, may feel may because we contact less, our department rotation is more, I feel every day if let me do this repetitive labor repetitive military service every day, repeat every day, the same thing I may on the one hand don't like, then the hospital hospital actually is quite complex, maybe I am not not particularly suitable for.

10:51

Give me the feeling is such, you in the exam month when you will feel that more anxious situation, generally in this more high-pressure environment, is a freshman may not play one thing, because now the old man, just. Already familiar with, already used to. Because at the freshman year, whether it is backbone department or non-backbone department, then backbone department you will be not anxious, is not under pressure, is will review and review.

11:20

There's always a process, and you can't say that there's no psychological fluctuation. In fact, at that time, I could not change my major at that time and I did not change my major. Another reason was that my grades was between 20% and 30%. I could turn to pediatrics. On the one hand, I can't transfer it and then I am really not interested in it. I may be a little better in a freshman year, and then anyway, I know more and more about the basic major. I may like it more and more in the basic aspect. What do you think about what it appeals to you?

11:52

It means that interpersonal relationship is relatively simple, and I like scientific research more. I may like scientific research more, because after I entered the laboratory, I also took the topic by myself, and I may like this exploratory thing more. When you just said that you got a topic, when did you get the topic? How many big things? Sophomore year promoted in the summer vacation of the junior year, because the sophomore year when I was still in rotation, there is a rotation system, in the country is now rotation. Then I actually decided down my sophomore year, after the sophomore year, I was decided in my current teacher's laboratory, I told him I decided down I want to go.

12:29

I think I like the atmosphere because of the city. Are these people very good to me. Then at that time, Miss Li just gave me a topic, and I have been doing it. So after doing it, I gave me a feel right. At first, my elder brother probably took me, because I can't do it in some time, and then I took me, and then I will do it alone. I probably like this one even more. This topic just got hands, a blank, I myself graffiti on it, how to paint how to paint, of course, is also an organized coating, and then so step by step to explore, I think quite like this feeling, so the main reason in the foundation is your interest, you can say so.

13:09

You may not know much about it in your freshman year, because you didn't go to the lab and didn't do much experiments, and you gradually liked technology. If you know more, in addition to your family, right? Have you ever discussed it with the teacher, or said no? Including counselors, including your professional teacher, who didn't discuss it much in your freshman year. No, the big one has not been discussed. Students because at that time when the national middle class just started a business, because the first page did not understand anything, in fact, also did not say specially to ask the teacher to ask me.

13:44

Do you think there is anything more impressive about the discussion?

13:49

The discussion did not seem to be much discussion, because at that time the exam month review. I have discussed with me, they actually wanted to apply, but then, because the grades were not enough, he couldn't apply for the top 50%, yes, because he seemed to have two lower courses, and then he couldn't apply.

14:14

I have roughly summed up the advantages of the national middle class, one is scholarship, the second is scientific research tutor. Scientific research tutorial system is to have more opportunities to contact with scientific research mentor teachers. The third is the characteristic teaching arrangement, which is to add some scientific research courses, and the characteristic construction practice. The fourth is the exemption from research, and the fifth is a 5 + 1 + 3 undergraduate and doctoral model. The sixth one is to go abroad for further study. Which one do you think will be more attractive to you?

14:46

You said that the most attractive to me now, or said that when the freshman year. You can't remember what you say, and you can't say it. At that time, the letter was actually a way to enter the laboratory, and a way to go directly. Because I so I can only say that my official website to search the first pick, I think maybe I will struggle for a long time, on this half a day, and then go to contact the mentor, not tangled right to right, the teacher can help you decide down.

15:26

Like not down, after he went in will give a mentor list, lead to the list of a two-way choice, and now I think this may be a little directly, because I may feel different time is to me may not be to my feeling may be similar, I just think into the laboratory, because when the freshman counselor is equivalent to suggest we will be early laboratory laboratory experience learning.

15:50

But there is no good way to freshman, at that time also mainly in learning to do grades, and then may not enter the laboratory. Then I wanted to say that there was such a good chance to go directly into the laboratory, so I went directly in.

16:01

It was a sophomore research rotation, and then four mentors, right? What did you think when you were choosing a mentor? Or do you think the tutor's topic is more compatible with your emotions, or this is one thing, because he doesn't have a mentor profile, he has a mentor's research direction is related, because I am asking this question why? Because you have been saying that you don't want to struggle, but I think you sophomore to 4 teachers that turn around, you still have to choose 1 from the 4 teachers, or I said not tangled, may not be a little so troublesome, I am actually quite afraid of trouble, but some things again trouble to do.

16:43

Then, after all, he gave you the equivalent of a list of mentors, always said not as big as the technical college, the whole selected so many teachers to let me a jump, not very jump bad.

16:54

My technical school inside to choose the direction to adjust, the direction inside which teachers are not fierce, and then the laboratory atmosphere is good, and to adjust, I think this may be my own actually want to pick also can pick, may be more painful. At that time, your mentor was one of the four mentors, and I could only transfer three teachers to me.

17:14

Three teachers to me the first round in that teacher, I went back to the last round. Why did you go back? Because I earned me in front of the three teachers, I think it may be about the same, and then just the first teacher's atmosphere, I really I like the teacher is also very good, the laboratory brothers and sisters are also very good, on the laboratory atmosphere briefly say what is the content of the wheel? Why do you feel that all three wheels are similar? In fact, when we entered the laboratory, we may mainly learn the experimental technology, but in fact, the basic experiments like pcrs may all be similar, so the basic experimental methods should be the same, and what may be different is the atmosphere of the laboratory.

17:50

Then the character of the tutor, the strength of the tutor, in fact, we did not feel too much at that time, because at that time, maybe we can say that we did not have much contact with the tutor at that time, mainly based on our own study.

18:02

Then I don't know about the strength of the tutor, and then what mainly feels me the most is that I think I think the atmosphere in the laboratory is very good and happy.

18:16

So in the process of learning, you are very concerned about the interaction with others, is in the time of self-scientific research or similar, because I think it is good, even if you said that after the graduate school always has to work for three years, why can't you be happy and bitter for three years? You in me in a laboratory atmosphere is not good, every day intrigue, I think my own work is not good work.

18:38

You and the lab elder brothers, sister, or yes, younger brother, sister will also go out for dinner together what, won't it? Have you ever discussed it with your parents in the activity class? Discussed, but they don't understand much either. They actually don't understand, or mainly to me, is starting to see me. So now I want to go back to a point that you just mentioned, and you said that you got the topic, and you just say it. Can you tell us about this in detail? End of sophomore year.

19:14

Was it then?

19:15

"Yes", I told my tutor at that time, I said I couldn't ask that, When I asked him if he could say that I had been there ever since, And then he actually agreed, After having also agreed to it, Just as my elder brother has a man in his hand sales more, There are so many topics as there are, It means it's an atmosphere and it's a subject that hasn't started yet, It's like asking him to take me to do it first, Because what is it really said about this subject, This topic was actually being done before, When my former grade 16 clinical student did it in him, But nothing was done when it was done, Sales are not enough, Sales haven't been built yet.

19:51

Then he was a junior year, he was going to leave, going to go to the hospital for an internship, and then put the subject. So after I go in how students do, not do, because haven't started.

20:00

Mice haven't react is, the students to time is too short, there is not long enough time to do sales, time point may be too not too good, because he came when he was ready to do subject may have been junior, because the clinical is not to first, to the next point he may also can not do behind, then mice didn't come out, so he to time is wrong.

20:17

Yes, and then when I went there, just the mouse came out, just when the mouse came out and there was a mouse to do, and then just give me this subject, just give this subject to you, and then your senior brother will take you to do this subject, right. Was you so excited to get this topic, were you? So you are pretty excited. Because the first time is equivalent to a subject, well, the subject is equivalent to a teacher born like the graduate model, and then send it to you.

20:48

You can roughly say what the direction of the topic is. About reproductive medicine, too. The Catip gene knockout can be found that the gold is motionless, mainly to explore why one causes the gold to remain motionless. At that time, when you have encountered any difficulties, or or to say that the excitement is greater than the face of the beginning must be more excited, and then do it must not be smooth sailing, there will be.

21:18

What I remember most clearly is the immune engineering electrical experiment, because on the one hand, the group just came out, there are not many groups, may do the best if a success, if a failure, may continue to wait for the mouse to react.

21:33

I probably would have done a semester to do it.

21:35

For various reasons, because at the beginning, it was like I made the kit for the first two times because I did it wrong, and then I didn't make it. Is not not done, the effect is not particularly good, work to do the effect is not particularly good, and then the third after two or three times is the last two or three times on the inexplicable can not do out.

22:00

Because the kit may be more rigorous conditions, may then conditions is not quite right, and then do two twice is actually I did so many times is not good, then he do it directly, the most use is knock book, because before is no knock book, after he use knock book or do not.

22:15

Because of the lysate problems, various problems, and then I may do it for more than a week, and then I did a different method, so I don't have to change the method, and then made it before continuing to push forward.

22:26

In fact, at that time is quite painful, because it may be quite long, this time to do one may be nearly four or five days, because at that time to do experiments I have to choose the time, at that time, there are more classes, I sometimes I actually at that time is not to say to skip class to do the experiment, 20 should be already a junior, right?

22:41

methinks. Want to right. It should be a junior year. In the junior year, because there were mainly classes, at that time, there might be more classes in the junior year, so I would have to choose, such as which day there is no class, and then to do the experiment that day, and then arrange the time point, and then reasonable arrangement, so maybe I can only do it once a week. So he probably studied for nearly a semester. After making it at that time, do you still remember what the mood when it is? I must be very happy, always return.

23:14

It's done, it's done, it can go back. I just wanted to ask which one wrote was a little messy. Some students will feel that after all, reading the activity class is to walk the road of scientific research, will certainly encounter such difficulties as you just said, and then like you did, successful, but there are many may be the last or still a failure.

23:49

Some of their classmates may think that scientific research failure may be costly to them, so there is a sense of fear. What do you think of this view? Some because it may indeed have plenty of time to do or the topic is not particularly good, they go to the back of the dead, go dead road can not go out, I think.

24:12

Before you sign up, have you ever thought about doing a topic later, then didn't think that much at that time, didn't think that much at that time.

24:25

I think now influence to do not have to go around in what direction, I can at least be sure what direction I choose. The main idea at the time was about the difficulties that might arise in the future. It was still not there right now. Is there a change now? There must be a change right now.

24:54

At that time, when I just entered the national middle class, I wanted to say, at that time, I want to go far away, and the final work to do, what to do? Because basic technology major or may the biggest problem say how to obtain employment? Well then just into engineering class, feel the teacher feel good, just want to say a book like the school those tutor to do a professor, professor, do and then do equivalent to a professor, professor, just run, and now actually feel this because they also know pretty much, also chat in the laboratory with elder sister, may feel like if like a teacher do a professor, actually feel not can't do very difficult.

25:40

I think I give me the feeling is now very right to me, now I must be very difficult, because I am still learning, because it may have to deal with many aspects, on the one hand, the fund matter, the fund and then the project declaration on all kinds of messy things. I think it may be a bit to cope for me now, if it might be difficult, maybe it may be a little better if I go to graduate school and PhD later. So for now, you want to do it and you haven't figured yet. In fact, I am a little confused. I wanted to stay to be a professor and an associate professor, so I think this problem may not be explored until my graduate or doctoral students have finished reading. Yes, I haven't completely thought it clearly.

26:25

Do you think the Chinese middle class is similar to what you expected?

26:29

For me, because I myself may I did not say think, graduate students to foreign school or to penicillin glue, I also think who also don't want to go to a good school, but I think maybe I also talked with the teacher today, he said those good school good cow laboratory may it itself laboratory school itself has been full, and then I can not go, we may not be able to go, went to some bad laboratory or laboratory atmosphere is bad, or the teacher is the teacher is bad, I also have a big.

26:58

Mainly because I just want to stay, and the most part of it is because my teacher's lab is very attractive to me.

27:09

What makes you very attractive is mainly because its scope is right. I think the tutor's lab is strong and may not have much impact, but I still want to stay in a better lab. Not just a lab with a better atmosphere. Actually I think you seem to for you, the laboratory atmosphere is quite important, including not only the laboratory, if you say in the choice of work, may for working atmosphere this point work did not think so much, I actually include atmosphere is not just a relaxed and happy environment, including and interpersonal processing between these.

27:52

Do you think you value it more often? May be because still want to achieve a slightly pleasant atmosphere inside work, including you just said as a professor may be a little difficult, but you said the difficult point is this is hard power difficult, so involves I want to know more about your personal just laboratory atmosphere, I think is this question, I want to change a question to ask.

28:26

What do you think for you in the recent years of college, including undergraduate, master, PhD, what do you think is the most important thing for you at present or in these years? Most Important: What is one of your biggest goals in recent years? Some people may think it is more important to become the president of the student Union, some people may think it is more important to find a girlfriend and a boyfriend, some people think the exam questions are more important, some people think the postgraduate guarantee is more important, and everyone thinks things are more important are different.

29:11

Have you ever thought about this question? What purpose do you think you've done in the years? The most important thing to you, I think it may be a little empty. Is it basically that I want to improve myself during my undergraduate period or that you haven't considered it? Is to consider, because the freshman certainly want to want to say freshman is want to say to change major, then later because do not want to professional, then at that time said to want to see can protect grind, protect graduate school I do not want to take an entrance examination too much.

29:40

Insurance research rushed to protect the goal of this research, but after this work class he does not need me do not need to be so as usual, must brush the GPA to insurance, on the national research environment protection research conditions are relatively loose, relatively small.

29:57

The pressure is relatively small, and then I did not think so much about the postgraduate research, plus my own results are actually quite good, and the postgraduate research is basically no problem. I may not have a wife to think about this matter, and then probably think about the most mainly is to say that I can learn more things in the laboratory, and improve my own strength.

30:16

You say is to protect graduate result product point is relatively loose, you now result probably in what position? Do you say China middle class? Or in the original technology? You protect graduate school is according to the basic major, because we do not have classes, I am not still in the foundation? However, some technical courses have been deleted, and the overlapping ranking should be in the top 40% of the original basic major. I think this has actually been satisfied, and my grades are definitely enough, and then I do not worry too much about this matter.

30:45

So you can now in the top 40%. He said so, but because the first session is not easy to speak, our first specific how to do, that wait is this year after the guarantee research out, he is not reported out a lot, oral commitment you are a long mind.

31:03

Is there any paper written commitment flyer or so on. This should be about the same, though that's true, right. Because after all, it was the first time, they like why I asked this question, because yesterday there was a student who actually for him, he wanted to get a PhD, so he was brushing his course, he was taking his course.

31:30

Yes, say its base point. So I don't know why he wants to brush when, his grades seem to be at about the same level as you. Are there any other policies for your postgraduate insurance? Is he a student in the Chinese middle class? Or is it the national middle class? As long as the top 40% can be guaranteed for graduate research, right? To. Just look at the results of the other is originally two ways, you either cut another 40%, or you post an SCI, then you can directly report to the test. But now does not seem to say that the national provisions of the article can not protect the research? Then cancelled, just look at the results I think actually pretty loose.

32:08

The activity may actually be the main way to make other majors more strict, right? What, really, is it, is the foundation? Basic insurance research rate is 20%. I think it's actually pretty loose, and then we may be 40% heavier than the technology. So in fact, for a freshman, the policy may be quite attractive to you. That should be said. Well,.

32:33

Because he thought about it, too, but it didn't mean that.Because I may mainly want to go, because I thought that my freshman score could actually be between 20% and 30%. Well, but it's equivalent to counting all the lessons, 20% to 3% of the time, and if only the main lessons, I should be at the top again. So I don't worry about undertaking it by myself, so the research guarantee is attractive, but not that big. Don't trust you, mainly strength enough, so don't need to consider these too much, say crazy is so.

33:09

You you just have a point in front of you I did not grasp to ask, now I am more curious. You said that in your freshman year, you might not say that you might not be so serious without anxiety, and you might be more nervous. In your sophomore and junior years, you won't be so nervous now.

33:32

What do you think is the main reason? Found mainly because at that time just entered the university, for the first time to take the final exam will be a little nervous, anyway, the back of the exam is more, including the final exam have, in fact, slowly equivalent to themselves also adapt to this environment. It was probably a little uncomfortable going from high school to college. You already know how to learn about it. To. Do you think you learned more about yourself, or the teacher taught more?

34:08

Because actually I think the university study may be given priority to with yourself, not like a high school teacher staring at the teacher after you learn, so the university is out to learn, you want to learn will always can find their own way, the middle class in you just said you have a very deep thing, take the subject that thing encountered some difficulties, in the difficulties of a semester, the results are not come out, did you change your mind at that time? Have you ever thought about not going to do scientific research again?

34:48

No, because in fact, I think this difficulty is a small difficulty. It didn't change because of the difficulty, and I thought you thought it was pretty small, because it could be made. Just because of their own reasons or objective reasons is not done can do it, but if it is really to that kind of thing can not be done, this topic is dead or do can not go on.

35:09

If I just started doing this topic and went it on, I think it might be fine. After all, it didn't take that much time. If I probably did it for two or three or three years and then found that I couldn't do it, it might be a little painful. I think the pain is still there, but so, at that time, you still think it was a cause of the phenomenon or you can change it, right, I know what step was wrong, or specifically why he didn't do it, I can roughly bring you a strong sense of frustration. There is no frustration, but there is loss, but it is not strong.

35:45

Yes, because at the first beginning of the experiment, always failed, always will experience a little not particularly good, including said because the collection is a little more expensive. I have something I struggled with, so the pipe is matching, maybe after use, after the pipe is out, the whole design is to be rebought. Then at that time, it may not be about four or five times or five or six times, may be more tube words, anyway, I have a little distressed, and then I said you rest assured to do casually, don't say because of this thing dare not do and so on. Is there any other things that make you influence more deeply, just start with the whole sophomore year, or make you lose more things, if not, you can skip it.

36:28

Except for doing the experiments for a semester and finally doing them. I didn't feel it, I didn't say very excited, I wasn't particularly disappointed.

36:40

So do you, you think you and I can just say, your thing is a personal emotional ups and downs will not be so big, can you say so? Another question I want to ask is yours or a personal question about you. There are two things, which thing do you think will give you a greater sense of pride or accomplishment.

37:08

One thing is that you learn a skill, such as a scientific technology, or an experimental method, or a new theoretical knowledge. The other is that you took the top 10% of the class exam, or for example, if you reached the top of the exam. Your previous ranking, do you think which of these two things will make you feel more excited? Or the first is the first for me now, the first for you now, but for the freshman may be the second you, such as the freshman just into college, may be the second comparison.

37:44

First because I to tell the truth now result is pretty top, pretty top, then because I do not learn at ordinary times, at ordinary times I may learn not let them really go to the library to study every day, I don't like to learn, I just purely to deal with the exam, so may be good grades, of course, is pretty happy, but not so not so let me excited, because after all, I these years actually pretty top.

38:11

What is your personal time allocated for? If you say not to read, sophomore? Or freshman? You just said you don't go to the library to study, is it looks like now, or from a certain period of time? As if we didn't spend a lot of time to use in theoretical study, because I found that maybe I started from freshman, freshman may be a little better, after a sophomore year, may I set a time before the exam, such as also the exam next month, I spend this month time to study, I will pick a time concentrated review.

38:58

It's just a month before you go to the lab and go to the lab, and you just play on your body, or by yourself in the dormitory, or when you do exercise. Because in fact, I often played and ran with them and so on, involving your mentor, you discussed you and your tutor in the past. To. If you really have a chance to go, you see of course I won't say negative why you will certainly go, right? Everyone normal a student is willing to go to a better environment, but if I just mention a hypothesis you have the opportunity to go to the north resume this more top universities, but we all know that the strong people around the school will be more, you may be your grades may not be like in the south.

39:54

If you think all the environments are the same, including if I give you an example, if the atmosphere of Teacher Liu's laboratory is all the same in the Peking University laboratory now, I may go to Peking University, but you will still go, right? Because I actually graduate students now, mainly where I want to stay, Mr.Liu's laboratory is not that I want to stay, so like the ranking of these things will bring you frustration, in fact, not so strong. Because as long as say do not affect me, just like so, only if do not affect my later graduate school, I test 100 and I test 60 there is no difference for me.

40:25

The results are right for you, as long as it does not affect the results. Before you read the Chinese middle class, did you feel the most proud of the most accomplished or fulfilling thing, is what you just mentioned as doing a semester of experiment success, is that the thing you feel the most accomplished from your sophomore year? In fact, but there is a thing, in fact, some time ago but is not the activity class, is the public. Yes, do you say that? Data plan big innovation. It's not a defense to step down, and then I use what I did before to defend this subject.

41:13

We just to and defense yourself PPT, I am equivalent to say this thing from beginning to end is I a person doing, although said our group has several other people, because several other people are also we show, undergraduates, but they either just come, or in the outside internship did not do nothing, but I still pull them together, because the topic may want to say together.

41:34

These undergraduate students are your country middle class students or the original technical students, there is a notary class students, some of the original foundation, and then from that time in place I a person to go there after the defense, at that time is the first all is you a person to do it?

41:48

can. So say. Because some time ago, they did not start to do it because of their own things, and then I was always in charge of this topic alone. And then it was actually pretty happy after playing in the first place. Where is the joy? Because pass is happy or take the first? Take the first place, is to take the first place by a debate, so you will choose to often participate in this kind of extracurricular scientific research interest activities, right? I didn't participate in this kind of competition, I participated in a lot of games, right?

42:25

Not a lot, I actually do not participate in a lot, some competitions I actually have nothing will suddenly think of to participate in the big creation. I was in the Challenge Cup as a sophomore, similar to it, and then I talked to my tutor, and he asked me if I wanted to participate today.

42:40

Then I said I could go there if I wanted to attend. Because for me, it's not that attractive to them in this competition, and for me, it's not much difference whether it is or not. But if I participated, I would certainly want to do it well, take the first one is to take the back name to run. Then just at that time, Miss Liu said to attend today, and then use the topic I did before, it is equivalent to do not have to open a new topic. On the one hand, I can not delay my own time, on the other hand, I can let me manage the whole topic.

43:11

Then there is a defense, equivalent to let me one more chance to defend. Because if I go to graduate school, I will always have a defense, and then let me experience it in advance, so in fact, the tutor prompted you to attend this time, part, part, part, part? Today I listen to your speech, I feel like you seem to attend this is an optional one, in fact, I just feel dispensable, but if participating, I will definitely want to do it well.

43:36

Because I was a sophomore, I was not a team, I was mainly in charge, I was just a participant, but this was the first time. The first time I was my team leader, I certainly did that project, you participated in the middle part, but because I was just a participant, not a team leader. You have attended a student union or a club. Have participated in. What clubs did I join in? The club has a student union, in fact, teaching is not a student union as a student organization.

44:14

Was I a freshman here today? Big original is I right, then junior high school class, the middle class, because at that time is so, I freshman not did not attend the student union, did not attend the student organization, and then people often say I feel at that time to university, a student organization don't attend a little not very good, on the one hand, I want to experience the life of student organizations, just do things that kind of atmosphere.

44:41

Then I was like the specific stage I can't remember, anyway, I went into the group.

44:48

There may be a semester, because a semester to feel, I purely feel I came out, feeling? It's good, it's good, if I said it from the freshman year? There is always some. If I went in from the freshman year, I think I could do better, but my sophomore year may mainly focus on the platform, as if something similar to activities like ordinary people. Is this? Probably something like a Boeing thing. The Gospel is just about the student service club. In my sophomore year, I probably mainly went to experience it, and I did not do it again.

45:25

So I think I can say that you are a person who likes to create yourself a challenge or accept a challenge, do you think you are this kind of person? And I think it should be. I don't really think that that unchanging life is a little more challenging. But in the whole process of scientific research, ah, is actually to endure loneliness, to do it alone, do you not think that you and your personality will be a little inconsistent? What do you think of that? I don't think so, I don't agree. Because in fact, what I listen to your speech is like what you talk about things. You still like to do different things, to experience different things, and you also like to bring some challenges to yourself.

46:09

But you in the whole process of doing scientific research, we don't talk about the results, you also said, make it will have good feelings, but the whole process is actually very boring, including saying that one may do two or three times, three or four times, don't you think it will bring you a big challenge? Just give your personality is not right or right.

46:26

I do p every day, I certainly don't like, but I think if I every day I do this two or three days every day, can bring benefits to me behind, or behind the role of the time, I think this is ok, as long as although this process you may not like, but as long as you can achieve your final goal, will go to be willing not to do. Because but if you simply let me say I whole graduate student I only do PC, I am sure I certainly don't like it.

46:59

clear.

47:07

Well, so I participated in these handsome things. That is to want to experience a different life, right, because the sophomore year is special right, sophomore year may mainly focus on scientific research in the country. What do you think the biggest activities you participate in, like the tree Youth, is the harvest and feeling?

47:30

If you think no, you say no or have, because including the big gen, I equivalent to the first time, is not the first time, in front of so many people to report to report to reply, also experience is pretty good, and then including exercise my courage, including defense skills actually is very good, including handsome navigation actually more things, let's work distribution down, and then do things like this. And then it's good to say if you can see what you do work.

48:04

Have you ever been involved?not have. Can't you report it too much after failing it? That's right. Two, three, sophomore, three, fail will quit? That is either exit or can not protect graduate research. The school is like this, all the leaders like to add to themselves is to give yourself a lot of challenges, so said to bring yourself a lot of challenges, you will be afraid of the challenge, if said failure, have you ever thought about things?

48:37

For example, for a major innovation project, have you ever thought about what to do if the project fails? Have you ever thought about this question? I thought when I thought may be like if I went up to play there may not be particularly good, lost will always be, but I think I think I can take how many name is not important, I mainly want to say that they come up to fall, exercise themselves.

49:03

I can tell us about the general process when you prepared you to take charge of the big creative project. A week before the defense, I changed PPT every day, read manuscripts every day, because I was very nervous, I was actually very nervous. Is it quite nervous, because the first time is equivalent to reporting on the stage? You may be not used to it. When you did scientific research work before, for example, our laboratory may be different. Before our laboratory, the group is equivalent to the work report.

49:35

Work report we are equivalent to talking to the teacher, it is equivalent to my report to you now, I told what I did, and then show you the picture, and then the teacher told me how to do, and then including how to improve and so on the group meeting.

49:46

Will only be mainly graduate students to talk about literature and so on, won't say to let me not to say to let me go to talk about you to listen to you to participate in it? I attended, but mainly listened, so he's done less like you haven't actually attended this public speech before.

50:01

Where do you think your nervousness came from? Haven't spoken in public yet? To. In fact, it is not me, I can say that it is not from the fear of failure, or can not give a speech on stage, mainly for the first time, this thing was relatively nervous, because at that time, how many ranking I took is not important to me. Because I don't expect to say that it doesn't matter to me. So mainly to want to exercise, right. But can get a good ranking is the best, can not get a good ranking is actually also good, there is not too much utilitarian mentality.

50:47

The following question is about maybe or for sophomore time, I estimate it is sophomore time is about you have entered the doctoral class, did I have the second year ah? Have any junior students to ask you?

51:04

Someone has come to consult you about the domestic affairs, yes. What did you asked, we know that we have asked for several times, I think they have asked directly, yes, including level 18, level 19 may have asked me. Maybe find you all kinds of ways for them, because they are sometimes in the student union, because before a student said that he would learn to come to me in the sophomore year, after all, it is the next class, to the junior year, no one came to find me.

51:31

In fact, there is a junior year, because the junior year may go to find 19 pairs to find the 18 grade, so I am very curious about how to find you. Is some of them in the student union, and then have their student union, because we were in our junior year, our class of people are still in the student union? Someone is still in the student union, and then I happen to play well with some people of them, and then they will see that you play well with those people in the student union. Yes, because usually maybe my interpersonal relationship is actually ok, may play almost with everyone, play very well, they recommend you, yes, because they may also ask me, said I want to include the public class, have anyone just want to find someone to ask, and then may push me over.

52:06

What kind of question did he not ask? In fact, the main thing is that I think the most of them, they think they will not say 6 go narrow, so they think I set a direction so early, I finally can't do anything else.

52:17

You think there will be this kind of me, actually feel really not consider this problem, because in fact, to tell the truth, you finally read graduate student PhD, you are always is a research direction, you can't say you put the whole direction research thoroughly you are not realistic, is a point, I think there is no narrow direction, this direction is small, I think not much influence, is purely a direction problem, you are interested in Shenzhen, how you can do it, you are not interested in Shenzhen, you certainly don't want to come.

52:45

Do you think you have to determine a range of your interests before you come here?

52:50

Also is not to say interest, because I actually feel freshman sophomore, at the time of classes, there should not be how many people interested in scientific research, not even how many people interested in appreciation, may be mainly advanced to experience, if I personally think you come in after you don't hate, you can continue to do it. Because it's true for me right now, I can't say I like to upgrade this direction, but I know I can do it, I don't hate the direction, I can do it.

53:17

So like learning basic medicine for so long, I can say that you actually for any research direction is not to hate or unacceptable degree, but there is no to either direction this is a strong point of interest, yes, actually so for me, I may you give me what I can do.

53:36

I think so, I contact more, I naturally also not say like, is equivalent to may be more biased, because I know more, I know more, I know more, I may think I can do it, as long as I don't hate, under the premise that I can continue to do, but if some direction may I do if done after a while, I think I really don't like it, then I am not particularly want to do, don't like the point will come from where?

54:00

Is the experimental method too complicated, or do you study it? Of course I don't know that now, because I didn't meet, I didn't go in all directions, right. So just like filling in the college entrance examination, I really don't like some majors. Yes, so I just want to ask you, you are now the direction of you, can not tell where you hate, high school at that time told you that your parents said, some of you don't like which is undergraduate, on the one hand is undergraduate, on the other hand may be some like time do not want to apply to which school.

54:39

Not the school is mainly the general direction. In the general direction, I don't particularly like my classmates, because we often discuss it together before. Some students go to apply for food, but I think I am not particularly interested in video, because I may know less, so I am not interested in it.

55:00

Maybe it's because I'm not quite interested in it, and I don't know that. Listen to you say so much, I feel that you, you are a very strong ability, what can take up down, but also very like to bring challenges to their own a person. Is there anything, is there anything, or anything else? So when I listen to you, I was curious. Is there anything that can cause you and give you great interest? One of your biggest hobbies, I want to hear it. Interest you in addition to doing experiments, this is or study is not said that so calm, some things do make me very happy, but it is not to say that the kind of happy, to go crazy of the kind of less.

55:39

I think or in another way, is that you usually spare time allocation of the most time, if you really want to be interested in it, I just want to say that 5 + 1 + 3 can really read on, can really read 5 + 1 + 3, just go on according to this road.

55:54

Because it's hard to say if I can go right now, and if I can go, I'm pretty excited, yes, because I've been reading less for several years. Mainly because it lets you read fewer people, mainly right? Because you read it for six years, then if 5 + 1 + 3 reduce it for two years. Because equivalent to me actually undergraduate course spirit of quite much, equivalent to the undergraduate course as a graduate student to use. What is reducing the number of study for. I noon I also don't know how much, I think they may just want to say undergraduate stage let us when a graduate student to use, equivalent to the last graduate student may give us for two years, but students are certainly happy to 5 + 1 + 3 above, but the teacher may not be happy to the teacher may not be particularly like, why don't the teacher like it?

56:43

Because you want to exercise more for a few more years, not all exercise up, it is equivalent to a few people did not confirm to work for you, you are by yourself to cultivate a student 5 + 1 + 3, is there a limit? Or, I think his expression seems to be for him, the results can meet the standard. I feel hard to deal with, the audit standard can be, should be once reached 20% or how much, I forgot a little, that 40%% is a graduate student can, he seems to be a graduate student.

57:15

When he studied for 16 years, he had a similar defense. You reply and you can be 5 + 1 + 3. Defense but you are normal 3 plus 3 defense is what? What? It may be similar to the opening report, which is like what you do yourself. So at that time junior junior asked you, will the direction become narrow? You say you, your personal opinion or you will think that if you have come and you can try it, if you really don't like it, you don't need to report it. Basically still see whether their interest direction is in line. But I estimate not necessarily may you think to ask this question of students generally said, maybe he doesn't know his psychological, yes, mainly because freshman may not know their heart problems, so I told him because anyway is allowed to retreat, I said you know flat ball advanced to report, so give these people advice or give them to give them a try.

58:14

Yes, you can take a look ahead. Try it first. Including said you learn for a year, learn after a year you quit, because there is no impact, return a year when the time comes even if you fill and fill is not much. What other people impression more deep question, is Li Xuemei to ask you to consult you.

58:34

On a direction of display, in fact, the main there is almost equivalent to the country is not the class? Like neurobiology like what hygiene to biology, and biology, this is an added class, and then delete a few courses, and then they may say that I took these a few courses are the equivalent of a loss. But what does it mean by losing money?that is to say.

58:56

To almost is this one, is almost this meaning, but I personally think the Zhongshan that several courses, you say not nice, you last to senior, not many people will listen carefully in class, you finally to junior senior are veteran, basic not class, junior class, junior senior that, sophomore actually little sophomore, sophomore not how on. Mainly in the junior and senior years.

59:18

Now because of the change, now because grade 18 is not quite the same as us, 18 level class actually changed a lot, anyway, almost probably on the class we have they are basically probably on, like what magic biology, like hygiene, he actually you have not taken these classes, right? I haven't been to me, but you still know it. Because my roommates are fashionable, ah, so they don't listen to the class, they think it is useless, they think it is purely to deal with the exam. So I don't think there should be this concern, but they probably think it makes a little difference for myself. It doesn't use, it doesn't use for future work, so future research doesn't use.

59:55

In fact, it is not good, choose inside and outside women and children is not too useful. For us, our major because we are not clinical, but learning always said that you may always learn things, like the health student he took that class I did not on, but in fact, I think those classes you are useful, you have two years you will forget, you have not used, you have two years you will forget.

01:00:14

If you are in this direction, you use every day, such as I in physiology, I use physiology every day, I may not forget for decades, so I think I personally think this is there is no problem, so junior to the question, you also think there is no right, anyway, at least I also told them, and then how they themselves think I don't know.

01:00:35

So there may still be some concerns about whether the class will have any impact? You, you, what do you think, from your perspective, from you, or from your feelings, and what do they think the impact affects? Do you think it's hard to find a job, or because it's a freshman? I first entered college in my freshman year.

01:00:54

If I don't understand, I feel lost, yes, because I just entered the country at the beginning, we are not to say to have a class, the first is to say to delete all that words. I actually at that time I also think I am not happy, I said my side there all the women and children on, I did not learn a lot of things less? But in fact, later that piece may also listen to sometimes, but not so exaggerated, I took the last semester on the second semester exam I may not remember.

01:01:18

Why did you think you had to cut it all? When you were a sophomore year, you might think you just talked about yourself. For you, where did you think the anxiety or unhappiness that class brought you came from?

01:01:33

At that time, anxiety was either anxiety, or if I was unhappy or not, I thought I saw a few things and learned a lot less, because I didn't want to become a purely scientific robot, I still think about accepting new things, but from what I see now, it's not very different. Because anyway, I forgot this issue of me last semester.

01:01:56

It didn't mean you didn't want to be a pure research robot, and you just didn't want to learn all about research. When I was a sophomore year, I might want to learn more things. No matter I said you might see more knowledge, you said that the pure scientific research robot was you at that time, which may not be quite right.

01:02:17

Never mind, is that what you really thought then? What I want to ask is that you think that after going to the middle class, doing scientific research is not so much, because it was a little bit more at that time. Because at that time after the feeling that it is too little, I think a little not good, that is to say can add back. Then I did add it back, but now I think I just do scientific research, I can't arrive now, he deleted all the class, I can spend another day.

01:02:45

How did you make such a change? Is an old a challenge, there is nothing special, is on the class more always return, right? I'm not the kind of everyday class understanding, is your understanding of this aspect. Added to the activity program in all aspects, right, because after all, senior, know more things. And then you are more clear about what you want to learn. Yes, I know what I should learn and what I should learn at this stage, and I know it myself.

01:03:17

Then you think your time allocation plan is really good for my time allocation. If it doesn't have much to do with your long-term goals, you will choose not to choose not to do it as much as possible, or not to do it, and you may not spend so much time on it.

01:03:38

Because include to say because have an exam you learn always return to learn or want to learn, but you are like if say we say absolutely a bit, a certain course is useless, if say to delete it, you still can be more happy. You say when is now you all want-23, I don't care, you want-23 I am very happy, not to say a little useless, possible.

01:04:05

For this stage he may be an experiment, for example, such as clinical, he think the painting is certainly useful, he finally clinical that painting may be used, but maybe I after I do not go to the hospital, I also pay two support dead is my family was ill asked me where I uncomfortable how to use me, or I may be able to answer him, may not so big, no practical effect.

01:04:28

To. But learning always still want to learn, after all, you have this course this role is mainly for you, you think it is for your work in the future work service service, this role you guest? You don't feel that it is necessary. In fact, I now think that in addition to the more basic courses of physiological life, it may be a little bit because the scientific research department will be used in scientific research, including in life, but in fact, relatively speaking, business must happen, not what I can also use in life.

01:05:06

My parents, my relatives and friends do not ask me, I can also give him the answer after sending the book, he felt that it is not equivalent to the clinical demand compared with the clinical words, the demand is not as high as them.

01:05:18

What do you think is the biggest harvest of the Chinese middle class during this period? No special feeling can not say, no, because there is no special big. For example, if I did not enter this class, I was just a simple basic medicine, I also choose Mr.Liu's laboratory, I did not make much difference. But you don't say, like at the beginning, that you said you don't like it, just jump over that process, just jump over, right. I don't have to say that again.

01:05:51

Maybe the country middle class for you to choose a little more convenient, including the rest of the harvest seems to be no. You can learn anywhere, because I actually think that if you want to learn anywhere, you can learn anywhere, you will not say that because of this class, you will learn more things, learn less things, you want to learn always can learn.

01:06:09

Are you anything unhappy or unhappy during the fruit tree class? Things that are stressful for me, and not for me. Have internship problems. Internship because we go to the master and practice is too far away, too far away every day back and forth. You should also be over in the internship now, it is over before May Day. Because today to run back and forth and have no school bus, and do not let us sit on the stage, may be more painful, because we have to get up at about 6 o'clock in the morning, to work in the hospital.

01:06:36

Also look at the department, some departments put the early, some departments put the late words general 5 o'clock. From Monday to Saturday? Monday through Friday. Want to practice to the master? How long are we for three and a half months. From three and a half months to 6 o'clock every day, I will almost take the subway from the Jiangling side to the Xinjiekou side. It's really pretty painful. Because because of gene-based internships, where do they practice in basic majors? Jiangning or righteous father. Jiangning Hospital, right? Yes, even if Jiangning Hospital is not far away, then why ask you to eat vivo?

01:07:12

We also want to ask this question, he may he feel closer to the appreciation, but actually not too big for us, not certainly must still understand the hospital, I think this may have, but just I don't have to say others, say your personal feeling must still have, but mainly is really was before class thinking about practice, how good I go to the hospital, no class more comfortable, now is the internship thinking about class every day, how much class, mainly from the commute, this in the whole process of work is basically not tired.

01:07:51

Because our internship is mainly to see. Right, because I see that we are not clinical after all, he can not say that like clinical, let us go to see the patient is mainly a view. So in fact, I think I can't call it much pressure, should call you not very satisfied with a thing. Yes, the passage time is too long, yes. Because that was what they thought, so in the fire. Otherwise our class dormitory is thinking to come back in the evening can also go to the laboratory, I also think so at the beginning, said I may every day 6:00 to the school to recognize this number, I was at the beginning is recognition, but later found too tired, too tired, I come back at 6 o'clock every day to die, directly back to the dormitory to sleep.

01:08:40

Because you basically didn't go during that time, and the lab basically didn't go, then you didn't think it was too expensive for you, and it took up too much time.

01:08:50

No, because the internship always belongs to the internship, and maybe I'm just the only part. It's just that it road be a little long. You said that today after work to go to Xinjiekou over these things are also ok. Feeling stressful things should not be stressful for you. Perhaps the biggest pressure is the big innovation defense preparation for that time. So to speak, either the last 5 + 155 + 1 + 3 quota, is the sixth grade may be right.

01:09:28

And just maybe I asked to ask away, junior junior in addition to the two questions, should not have. One is about the direction and research direction. Are there the only two main questions, right? Yes, that's the main thing, but very few others. Because the other 1 / 3 of the also does not say to ask I actually can roughly understand. Yes, they can know about it in other ways. Other, I feel that the study of the national middle class must also meet your expectations. After all, you think and is not in the national middle class for you is about the same, right, so you are still more recognized, including said the latter 5 + 13 + 3, in fact, to my excellent to my preferential strength is quite big.

01:10:25

So in fact, can I say that like I just listed these 6 points, you actually did not have a very clear choice at the beginning, I can I say that 5 + 1 + 3 is the most attractive to you, you are right now, because you did not understand at that time, not a lot of understanding, may also be a blank just say. It's more convenient to come here, yes. So you are a strong motivation to read a blog, and I must read a blog. If you read a PhD, you have discussed this matter with my family, classmates or teachers. My parents also know that they also know this major and say that it must be read for a PhD. If you want to find a job later, it is gambling.

01:11:02

Your mother said that this major must read a PhD, so do you think you read a doctor because of the necessity of more, or do you think I want scientific research to gamble, which one do you think accounts for a larger proportion?

01:11:17

Or all about half the same way.

01:11:37

When you discuss with your parents, it is talking about reading a blog, or in another way, I want to know if your parents will give you to start from the college entrance examination, including every step of the walk, will they give you suggestions or opinions?

01:11:54

No, they basically listen to you except after they fill in the college entrance exam.

01:11:58

Yes, because they actually don't understand very much, because after I actually go to school, they actually don't understand a lot of things, may still listen to me, because I may include you are more willing to tell them.

01:12:09

At first, after let them know, it's up to me. But like my parents, they may sometimes usually surf the Internet to read information, look up some things online will also talk to discuss with me, but mainly to look at my ideas.

01:12:30

One of some people's views or ideas is that it will bring them a rise in their social status or a sense of honor, which do you think will exist for you?

01:12:54

For you, it may be more of a detail of the scientific research method, including a consideration of the future work. Nothing else should be there, either.

01:13:05

Then I can't think of anything else right now.

01:13:07

No, then there's no problem. I don't have any problem.

01:13:12

Ok, thank you very much for cooperating with us for the interview.

01:13:16

I will stop you up.

01:13:19

I will go and get this material.

01:13:22

thank you.

01:13:37

Is this the equivalent of your activity? What's it?

01:13:43

Not an activity, is our qualitative research, mainly to study the national middle class students on the choice of major in some influencing factors.
