# Supplementary material for: Why do you choose this program?—A decision-making model of medical students based on grounded theory
Source: PLoS One. 2023 Sep 15;18(9):e0291634. doi: 10.1371/journal.pone.0291634 (PMC10503722; doi:10.1371/journal.pone.0291634)
Supplement: S1 File — (ZIP) [file pone.0291634.s001.zip › RAW DATA/P4 CHINESE.docx]

00:00

但这个开始之前我要先在本次访谈中，受访者是在平等自愿的原则上参与的，受访者必须真实的表达自我想法和认知，确认自己符合受访条件，访谈的过程会被录音，其录音资料将以匿名的形式用于科研，不会泄露给任何第三方，在访谈的过程中和访谈结束后，你都有权取消呃研究人员录音资料的使用权，你是否知晓并同意？

00:30

我同意好。也就是现在开始想先问一下你的年级，我是18级的。18级的。预防过重是吗？对。你在进预防活动之前是什么专业？就是预防。我想问的第一个问题就是你先说一说，在大一的时候，你对国中班的了解有哪些？就是了解的渠道是哪些？我其实了解宣传的整个过程，其实我是从大一下学期才开始知道有国中班。的嗯。当时是大家在组织那种大创嘛，然后我就听一个同学说，其实想做科研不一定要通过大创这个途径，也许可以考虑一下报考国中班，从那个时候我是第一次听到有国中班这个名字，后来那个学期快结束了，会有老师过来宣讲回来。

01:41

照着急，大家稍微等一下，是你刚刚说同同学是你预防医学班的同学是吗？所以我们就那就继续吧然后他就会来宣讲，当时是我们18级的国政班的班主任，是顾老师来宣讲，然后他就给我们讲解了一些国政班的基本情况，我们对果冻班就开始产生兴趣了，就开始去了解果冻班了。

02:11

基本上了解就是一个这样的过程。你有没有跟你的家里面的人，包括你的同学、老师、辅导员，这4类人我就讨论过这个事情，我跟家长肯定是讨论过的。然后我跟他们也说了一下那些老师跟我们宣讲的内容和我自己的看法，然后我家长他说支持我的决定，然后我就决定报考国中班。

02:39

你和同学老师他们都没有怎么讨论，同学的话跟室友讨论过。室友他们怎么看，他们觉得这是个很好的途径，因为他们都知道我是对科研比较感兴趣，所以他们就也支持我，他们帮忙啊他们没有他们是不是条件不符合不相同条件不符合我觉得。

03:01

在高中的时候啊你是直接报的预防还是是被调剂到预防的？我们湖我是湖南的，所以我们报考是有6个平行志愿的预防，是在直接填的志愿中间，一个并不是调剂的。预防是你第几志愿？这个记不清楚，好像是3或者4。你在沟通的时候，一心要学医医学，文科学类，他就说你的当时理科工科你都报了，高中的时候还记得吗？学肯定是想学医学，但是报肯定都报过。我爸他也是一个医学相关的专业嘛，所以他对我的影响比较深，我一直都特别想从事医学相关的工作。

03:47

所以你当时报的话是除了医学还报了哪些？还报了比如说哈工大这种工程类的学校和理科就没有搞。理科的话，这个应该比较少嗯，主要是报了、工科和医科。当时报工科有什么考量吗？是自己感兴趣吗？还是也算感兴趣？当时我高中对整个理科就是理物理化学生物都很感兴趣，所以这三方面我都会去考虑。但是生物跟医学这方面是主要考虑的，可能对于工科来说，医科还是你主要考虑的一个方向之一。

04:35

当时你的同学也都知道你比较喜欢科研，你在知道了国中班以后你有参加大专吗？当时还是有的。比那个是在大一下学期就开始报名了。国中应该是考转专业考试之后才有才考的，所以大一下的大创是继续做下去了，一直做到大二的时候进入文革以后还在做。后来就没有做下去了。

05:03

当时是是什么原因？就是我们组员好像内部应该是没有沟通好，然后大家都比较分散，就最后就没有聚在一块做。没有功能好，是因为可以具体一点是可能是其他专业不一样，不好聚在一起是也不是专业，专业是差不多的，有未检的也是有预防的，大家都是一块的。

05:26

但是他们可能忙其他的事情比较多，在这方面付出太少。导致嗯就是我跟另外一个负责人两个人支持不下去，一共是5个人，所以就没有做下去当时。大创是一个创新的项目还是创业的项目？应该是创新的创新的项目。你们两个你和另外一个同学是相当于项目的主要的负责人，嗯然后，但是可能组员对你的支持，没有办法对你们两个人可能就没有办法把这个项目做下去，做不下去是因为可能任务太多了，还是说可能当时选题就有些问题。

06:11

当时我们虽然有指导老师，但是其实老师也比较忙，我们其实见面的次数并不多，大多数需要靠我们自己去摸索。

06:20

然后只有我们两个的话，我们就有很多方面就考虑不到，实验我们也去做过，尝试了大概将近一个月，然后发现都尝试不出来，然后其他人又给予的支持不是很够，所以你们当时尝试是在实验室里尝试那个实验室你们是怎么联系的呢？当时是班主任，他在我们班群里问有没有同学对这个感兴趣？

06:44

兼职班主任，对是兼职班导师，兼职班主任职，班主任对。兼职班主任就是在他那个方向里面，他有个课题。有没有同学感兴趣，对你和另外一个同学说你们俩感兴趣，然后就用这个课题设立一个大创项目对，这个项目大概什么内容可以简单的说一下。

07:09

是什么方向我想了解一下，我觉得它偏化学材料方向，材料是工信院他们那边的不是也是工位的是未检，他们是做一个纳米分子材料。检验那方面的材料对开发，但其实和和医科差的蛮远的，它算一个工科，其实那个材料它是可以再要的。它可以把我们需要的药物包进去，然后递送给就是你需要的患者。

07:42

你们相当于研究材料对。你不你你当时看到这个项目以后，你是很感兴趣吗？还是说我只是想试一试科研？因为我看题目可能跟医科的关系不是非常的大对，他还是算一个材料学，因为当时我对其他的领域并不是很了解，我只有这一个方向可以尝试一下，我就选择了尝试了，大一上的时候你有尝试过类似的科研室倒没有，正好有这么一个机会，大家一下的时候，然后同学跟你说有果冻这么一个途径可以去做科研。

08:25

对做科研。这个东西是你觉得你对科研的热情是来源于哪里是，来于家里熏陶，还是小时候看的什么东西这都有吧，因为我爸他他科学就特别感兴趣，虽然他自己不能成为科学家，但是他就是会给我买一些科普类的书，经常让我看为什么你爸爸成为不了科学家，因为他可能当时他没有上大学，他是读中专直接就出来工作，所以他的工作可能跟科研没有什么关系对，但是他对科学特别感兴趣。

09:01

嗯然后我自己也会买一些那种科学方面的书去看。比如说我高中的时候就特别喜欢看那种免疫学的书，后来我还会有一些奇奇怪怪的想法，后来就进入国中班之后，他就会去跟那些老师讨论，因为也有免疫的老师是国政老师，然后他们有些时候也会给我做一些指点。

09:26

他们对你的想法有什么评价吗？就是这些科研想法，他们就是觉得本科生刚刚开始接触，肯定天马行空的想法会很多。他说这种想法越多越好，但是虽然很多都是不切实际的，但是总会有一两个会议，有些符合那种可能可以研究的方向。他说反正还是挺鼓励我们去思考的，你在思考这么多以后，有没有在大一那个阶段，你现在应该是大三是吧？在大一的那个时候有没有在看了那么多书，也做了一次大创的科研项目。

10:06

在那个阶段情况下，你有没有发觉出或者感觉出自己特别的对某一个领域有特别的兴趣？他就说当时应该还没有有发掘出来，是在了解到在做完大创之后，还是对大一的暑假，大概那个时候国中班报名之前，就是说我倒应该还没有。

10:29

应该还没有，只是说对科研比较感兴趣，但是具体对哪个领域比较感兴趣，还没有没有特别的当时大一结束的时候应该你还有一个转专业的机会对吧？你有报吗你？报的哪里？我转的是儿科，儿科是吧对。我说假如你儿科你转进去了吗？应该是没有。假如你儿科成功转进去，你会报公众吗？如果转进去估计不会包括这种，因为当时对科研其实也没有太具体的看法，只是感兴趣对，但是相对于从预防和临床两个专业来选的话，你还是会选择临床。

11:16

你当时高考报志愿的话，其实还是主要呃偏向于临床。那些专业你会更感兴趣一点是吧对，没有报大临床报儿科是喜欢儿科吗？还是因为成绩还是因为什么别的原因？成绩成绩我当时儿科的要求是前30%，但是我是没有达到20%的，所以说报不了临床只能报个课。

11:46

所以当时转专业结束了以后，然后又报了国中班对对，谷中班你是参加了一个宣讲会。国中班其实它里面改的东西挺多的，你觉得当时对你来说冲击最大的，或者你印象最深的是有还是说没有特别的只是想想做科研，所以来工作。嗯当时是我总结了一下，我看了他宣讲的p第一个是奖学金很高，第二个是可以直接的就是从大二开始接触科研导师。

12:30

第三个就是课程的改革，就是它删掉一些课，又加了一些特色课，还有特色的建实习。第4个可能他有免研的资格会比普通的预防要高一点。第5个是5+1+3的，本硕博的模式你觉得这几个特点哪一个是对你冲击最大的或者最吸引你的。

12:50

可以了。可以接触到科研导师的那个点是吗？除了这一点，你还有什么其他印象比较深吗？就是参加宣讲会的那个时候。还记得吗？没有的话你可以说没有，我想一想。我应该没有特别深的印象。主要是科研导师可能相比较起来的话，还是稍微吸引你一点，对。但是你要是我让你直接讲哪个最吸引你可能也也不太好讲出来。

13:32

可以这么说吗？我觉得还是会讲可以，我觉得我的第一个想法就是你说家里也是比较支持你的想法的，他们就是你从小的家里的环境，就是说包括你报高考志愿，或者你比如说考高中的时候，嗯他们你家长会会是怎么讲会干涉你的自己那个想法吗？或者说会劝阻你，或者说建议你某些想法吗？还是说完全就听你的？

14:15

有有就是你人生中那么多的抉择，我觉得都会有看出什么方面的抉择。像填志愿这种的话，他们就会半代劝阻一半的建议，然后首先是我兴趣中间选一些出来，然后他们再从中挑，然后再给我建议，然后如果我选的不对的话，他会劝阻我。这样的。

14:43

劝阻是指什么时候是报高中的时候，还是报高考志愿，还是比如填志愿？假如我填了一个他们觉得不太好的学校，他们就会跟我说学校的专业可能不行，不符合你以后的兴趣，比如说我跟他们说我想做科研，然后他们说比如专业不能做科研或者奖励，就是什么专业他们不想让你报。

15:07

我记得这个好久了，但当时确实有讨论嗯。

15:17

所以当时其实一你报的是医科和工科，也是在你框了一个范围，他们给你框了一个更小的范围，之后做出来的一个选择，可以这么说吗可以这么说。

15:31

所以你的父母肯定是在你的意愿的基础之上，然后再对你可能有一些引导的这么一个家庭环境，你现在是大三了对吧？

15:52

大二的时候你们经历的是一个叫科研轮转，是吗？

15:58

对。

15:59

你可以跟我讲讲当时科研轮转。

16:02

4你是4个导师吗？

16:04

当时好像是由于疫情，最后第二学期只选了一个导师。按理来说他是一学期要选两个导师的，对，然后但是第二学期他因为只选了一个对，只选了一个导师，所以就是大二的时候你是赚了三个导师。

16:24

对。

16:25

三个实验室。

16:27

你现在选的导师你现在已经确定研究方向了，对吧？基本上确定了你的研究方向和你的导师是当时轮转的导师里面的吗？

16:36

不是。可以跟我讲一讲这个过程当时是怎么选的导师。

16:43

就说现在这个导师对我之前轮转过三个实验室在分别是在基础这边和工人那边都有。然后我觉得其实他们研究的方向我都不是特别感兴趣。然后后来我就跟我的进就是国中班的班主任讨论过这件事情，我希望他能给我一个指点。

17:08

然后他就跟我说了一下，我现在这个导师他说这个导师是刚刚来的，他有些什么研究方向，他也比较年轻，可能跟我们这种学生没有代沟。然后就跟我介绍了一下他我，就说那好我可以尝试一下，我就选择了这个导师，然后跟了他之后，我就发现他其实真的很不错的方向，我也挺感兴趣的。

17:32

你说前面的三个导师的方向你感兴趣可以跟我大概讲一下是什么方向，第一个导师也不能说不感兴趣，他们是做免疫的，就是我之前跟你说我，高中对免疫特别感兴趣，所以我第一个选择就是他，但是我发现他做生殖方面其实做的并不是主攻的方向，所以我觉得如果带着他们实验室做升值方向的课题，我可能并不能做出一些什么东西或者学到一些什么东西，我就决定还是专找那些做专门做升职的导师，是这样考虑的，然后后面的导师的话，我觉得他们对于我们国中班的本科生去他们实验室进行轮转，他们态度看法也不一样。

18:14

有些像在工会那边的话，他们对本科生就特别重视，他们就觉得你我我们国中班的同学来了，我们就一定要好好的培养他们，然后他们就会给让专门的师兄师姐来带我们做实验。

18:28

然后像在基础那边的话，有某一些导师他们可能会比较倾向于指导研究生跟博士生，可能对本科生并没有那么多的精力，所以我们在那边待的时候就可能会有一些他要求自己去看一看，其实并没有对我们有太大的益处，我觉得是这样的，你说在免疫实验室的时候，你可能不太能做到升值这个方向，对吧？

18:58

所以其实你不太倾向于在这个实验室里待。

19:00

你在当时大二应该是对大二轮转的时候，你在大二的时候就已经确定了，就要想做这个升值是吗？他就说进了这个班是要求你们一定要做深的这个方向的，这应该是两方面考虑，因为既然进了升值方向的过程中，肯定是要跟升值相关的内容。然后第二个的话我也觉得其实升值那边我还没有去看过，因为第一个选的是免疫，然后我就想去看一下升值到底是个什么样子。

19:30

升职还没有看过是，就是没有去他们实验室看过到底是他们在做些什么，没有去轮转过这个意思。

19:43

你觉得当时工会对俄生比较重视，结论是来自于他们可能会找一些师兄师姐来带你们，从手把手的教你们科研方法是这个样子。

19:56

他们不仅仅教的是科研的方法，他们还会教你一些思路。

20:00

比如说我遇到这个问题，我应该怎么去思考它，怎么去解决这个问题？

20:05

是你上课的问题，还是说在做实验的问题？

20:08

实验的问题对，那就是说明你在轮转的时候，你就已经可以在他们的工位实验室里做实验了。

20:15

你的实验室你自己是他们给你分配的一个时间任务吗？

20:19

还是说老师分配的一个时间任务，还是是他们分配的时间，然后师兄师姐让你来做，他会先教我们，然后我们都会要求要不让我们自己上手试一试这种提这种想法，然后他们基本上都会满足你们，然后会看着你们做，然后对你们做熟练之后，你们可以自己大家可以自己承担实验任务的时候，他就会让你们做，在基础的时候可能就不太能做得到是吗？

20:48

至少我大二的时候是没有能做到的。

20:55

但是可能你后面轮转的两个工位的实验室方向，你还是不是很感兴趣。

21:01

是这样的。

21:02

后来你才选择年轻的老大，其实在工会他只那一学期，当然下学期其实只轮转了一次，就待在工位了，然后我是大三的时候换到换到新导师这边来，大二那个实验室就是大二下学期实验室的方向你也不感兴趣是吗？也不是。因为我想我觉得我做了一定的实验了，我想想承担自己独立承担一一小段的科研的过程。

21:31

然后但是在那边的话，可能他是始终在师兄师姐的就是说他带着指导你带着你做的情况，下你单单只做了实验，但是你没有一个完整的那种过程，就是科研的过程，我想去尝试一下整个过程。

21:48

所以我就换了这个显示。

21:51

我比较好奇，你有想过为什么在大二下学期实验室没有办法独立承担一个项目，而是到了新老师以后，你就可以去承担这个项目，是因为竞争太激烈还是这个东西其实我后来我当时考虑的是我以为我能力是够的嗯，但后来我那是大二下学期，现在是大三下学期，我是大三下学期选的老师，中间还经历一个大三上学期，那也是在其他的导师那儿的。

22:25

然后你大三上学期也在轮转，大三有两次轮转，后来我经历了就看的多了之后，我才发现其实我的能力并没有达到。

22:36

血拼。

22:39

并不是因为我嗯觉得我可以，然后就去那样去做，其实他们我不给我们承担，而暂时不让我们去做一个阶段性的工作，是因为我们觉得能力还没有达到。

22:55

你在大三上的时候又轮转了两个实验室，大三整个大三轮转两个小时，整个大三相当于你大二下大三，你轮了5个实验室。

23:07

对。

23:11

所以说你现在选的实验室是大三轮转的实验室对。

23:17

明白了其实排除排除掉。因为你现在也说了，可能在当时那个情况下，你还没有能力去独立承担一个实验项目或者科技项目，小孩排除掉这个因素，你还愿意回到大二。

23:38

当时那个实验室我现在怎么说，我现在这个导师其实跟我之前实验室是有关联的，所以我经常要在这两边同时要在这两边做实验。我现在记在我现在这个导师这里做，也会跑到大二下学期导师那边去做。噢所以是他们俩其实是有联系的。你们做你做的东西是他们俩共同生生的一个课题，是这样的。

24:06

其实也不能算是课题，我只是承担了一一小个阶段性的工作。当时所以说你刚刚说的说你和你的班主任讨论了一下，他给你推荐了一个刚来的年轻的导师，这个事情是发生在大三的下学期这个学期的开始就是大三上学期的末尾，上学期的末尾上学期那次轮转了，有寒假前。

24:34

对。

24:37

给你推荐了这个导师，然后你觉得没有代沟，是他说的还是你说的？

24:44

这个是他大概的意思，我理解是理解是这个样子，因为他说比较年轻之类的。

24:54

你也觉得这个倒是很不错具体。

24:57

可以具体一点。

24:59

他比较他跟我们坐在同一个实验室，我跟他基本上坐的很近，我有什么问题我都可以直接问他，那些就算是非常小的实验方面的问题，他也会非常细心的给我解答，所以老师可以给你带来很多的指导，所以你觉得这方面很不错，你也会比较关心导师对你带来的帮助，或者说你也更希望通过国中班从导师那里获得很多学习到很多东西，应该是可以这么说的。

25:41

对。

25:43

所以我可不可以说你其实对于保研或者说本硕博这个事情，是一个不是那么重要的位置。相对于科研来说的话，它应该算是第二应该它并不能占据地位嗯，所以你现在是相当于承担新导师的课题中的部分的内容的工作，对吧？

26:08

应该是这样，你的研究方向也就相当于是和他保持一致了。其实对他的研究方向我也不是特别了解，这些方面我真的没有沟通，我只知道我自己这个方向是。

26:21

你现在在做的大概是什么内容啊？

26:23

做一个跟肿瘤相关的基因，然后要看一下它的作用。

26:29

肿瘤相关的基因好像和深圳好像基因在它比较特殊，它只在生殖系统跟肿瘤中间有表达，所以我们要研究一下他们之间的关联，你做的感觉怎么样？你现在还在收集资料和做准备，阶段还没有完全的开始，因为这个时间我不会做，我所以我现在在工位就上一个就是大二下学期实验室，在学习这个技术，我等我学完之后就可以开始做自己的噢所以，你现在是正在大二下学期的实验室学技术手段是吗？对。让学习学校事业来带你是吧？对。公司。

27:30

你现在做的现在其实我可不可以说大二的时候，其实你在去相当于体验实验室的一个氛围叫你学习的话，主要还是可能就是大二下学期的时候，学长学姐教了你一些科研的手段，技术到了上学期的时候，可能开始真正的学学到了更多的科研技术，但是到目前为止还没有独立的承担过一个一个课题或者说一个项目，或者说一一组实验，可以这么理解。

28:10

你的进度是在你同学当中是算普遍的吗？你周围的同学基本上都是进度。不是，也有一部分人他能独立承担工阶段性的工作也，而且有人已经做出一小段的工作那种都有，在大三的时候就已经做。

28:31

出来了你有跟他们讨论过吗？我们会开那种类似于研讨会，然后他们会讲一下在他们实验室做了一些什么研讨会，是实验室的研讨会还是你们班级的研讨会？就是班级的。然后他们会说一下他们的进展，他们也有人就是独立承担老师的课题，或者两个人承担同一个课题。比如说那个课题有我有很多阶段他们已经完成了一二阶段的工作了，这个也有。

28:59

我也不是说会羡慕他们，我就觉得做好自己就行了。所以你也没有其他的任何的情绪。听他们讲一讲，也不会影响到你自己对我。

29:15

听他们讲主要是也看一下他，学一下他们在学什么东西。就了解一下。但是项目也谈不上，这种同学会比较多吗？还是比较少的一部分人？大三就已经开始做出了一个实验结果。他们肯定也就是顶尖的那一小部分人吧，我觉得大部分人还是跟你的进度差不多的。我觉得是这样的。

29:40

下面一个问题是你有了解过，我想问一下，国中班的确是做了一些课程的改革，然后有一些甚至生物学发育生发育生物学你们都学过吗？生殖生物学是学过了，发育还没有学过。

30:05

你有了解过预防医学被删掉，就你们不用上那些课程，这些课程你有了解过吗？有一部分了解过吧？你对这些被删掉的课程有想法，或者是有了解吗？嗯没有可以说没有。想法倒应该是没有的，没有是吧？你觉得他们加的这些生殖生物学这些课对你来说有有有帮助吗？或者说有什么收获吗？

30:33

有肯定是有收获的，他们选的这些课程其实我觉得对于我们生殖活动来说肯定用处还是比较大的，比如说生殖生物学，它是以生殖方面的一些很多的基础知识，长期积累来的研究，嗯我们要做升职方向的课题科研，肯定是要了解这些基础知识的，所以上这节课我觉得还是蛮有必要的。

30:58

你上这节课的时候你会感觉还蛮有趣的，还是说？我的意思就是想问你，你在上节课的时候会会有迸发出浓烈的兴趣？还是说只要上好课就可以，只要为我后面做科研不跑路就可以。

31:18

这个应该是只为科研跑的就行了，因为这些课都是非常基础的课，并不能带来一些前沿的知识，你从上了大二以后，在各个实验室轮转，会不会有没有做过一些尝试，有没有尝试过了解预防医学一些其他甚至以外的一些一些学科的内容？

31:37

也有人群之类的东西。流行病学、流行病、统计学家。类似这些你有了解过是吧？有了解过是基于什么样的目的，就是感兴趣了解一下，还是随便翻一翻了解一下？我觉得我毕竟还是预防医学这些也属于专业内的东西，我觉得还是有必要去认识一下这些讲了些什么东西。你在做科研的时候，或者说我我换一个问题，你可以跟我说一件你印象最深的事情。从读国中班以来，在这么多实验室轮转，印象最深的事情，或者说假如你一下子可能想不起来的话，有没有让你最特别高兴的一个事情，或者让你特别不满的一个什么事情？

32:31

不满倒没有。特别印象最深的，其实最主要我想知道一个印象最深的一件事情，印象最深的一件事情还是我就是来国中班之后，我接触的第一个导师的免疫学的，其实我高中的时候看免疫的书，因为我了解不不了前沿的知识，所以我只能产生一些天马行空的想法。

32:55

当时我有一个想法就一直留到了就是第一次见那个导师，后来我跟他聊了一下我这个想法，他跟我说我这个想法是这几年研究的特别火的一个方向，然后我就特别高兴，我的想法也不是完全的不切实际，偶尔也有可以简单的讲一讲我免免疫系，人体免疫细胞有一个细胞叫t细胞，他是参与人体的体液免疫跟细胞免疫的可以说是人的免疫系统中也离不开细胞，然后我想当时想法是我们的肿瘤，因为它肿瘤它是会有免疫逃逸的，就是说我们免疫系统先清除不了他，有些时候是识别不了他，这个时候就需要t细胞去工作。

33:49

然后我们怎么去工作？就是我们把嗯肿瘤的表面的一些分子就直接在体外让它的t细胞给识别了，然后再给它回收到，体内，这样的话它就会自动识别那些肿瘤细胞把它杀伤。

34:03

我这个想法是我一直看免疫书一直都有，然后跟老师讨论了一下，老师说这个叫做car t。然后现在研究特别火，我当时是很高兴，就这件事应该是最高兴的一件事情，很高兴。但是我看你刚刚说了是在免疫是免疫你实验室里面，可能他做的跟生殖的关系不是很大，所以你还是决定就是退出免疫的对实验室。你不会觉得很可惜吗？你高中想到现在这个想法已经快要接近了，也有一个因为来了国中班要叫做升职，我觉得这个因素还蛮复杂的。

34:47

一方面就是生殖，毕竟是国中班要做升职方向，第二个我也觉得免疫其实有点难度，虽然我的想法有时候那个想法是对的，但是并不代表我擅长做。我看了一下他们做的实验，其实有很多我觉得还是很有难度。

35:07

你觉得你下了一个结论说你不一定擅长，这个结论是来源于哪里？是你看某些东西你觉得很难还是还是一种自我安慰，当时我尝试过做实验，因为当时也是进实验室轮转，有时候会要尝试做一下实验。我发现我实际上当时做免疫的实验做的并不好，然后就没有什么自信心是指哪里不好，结果出现的不太对，嗯有些操作也不对。然后虽然师兄师姐她跟我说没有关系，下次再继续学，但是我可能当时建没有建立起来，自信心可能是这样。

35:50

所以我可不可以说你可能你在平时可能会是一种比较悲观主义的人就是，喜欢把事情往坏的方向想是那种偏一点。

36:02

对，因为有的人很乐观，有的人很悲观，或者有的人喜欢把事情往好的方向想，有的人喜欢把事情往坏的方向想，你觉得你属于哪一种？大多数情况还是偏悲观一点，悲观。一点这样会。你觉得会给你带来一种不自信的感觉吗？就是你的这种应该叫价值观。有时候会有会有影响，有时候会所以你在当时在免疫实验室里面做可能做了一个因为不太恰当的操作带来了没有带来很让人满意的结果。

36:43

是可不可以说是给你带来一点挫败感嗯？对，有挫败感。很强吗？反正偶尔会想到那件事情，然后最后就换了实验室有没有？想过可能你有没有曾经想过，可能我把那个方法学得稍微好一点，所以结果可能还不错。我后来是这样想的，见识的多了之后，我才觉得这就是大二下大二下或者说是大二的第二次文章开始，我就觉得其实这种事情很常见，只要去把它多学习多多做多做就会可以做好。

37:22

所以你再见了，多了以后，发现其实很多科研时候失败是很正常的。对，你在遇到这种情况也得到这种结论以后，有没有想到要再回到免疫再继续做 Par？有想到过有想过也尝试联系过老师。后来是联系免疫的老师是吗？对。后来他其实跟我说，我们国中班也有一部分要求，就是说要类似于假如是研究生阶段，可能是要做生殖相关的，但是它并不是擅长生殖跟免疫的联合领域交叉领域，所以他说这样可能对我的发展并不是特别好。

38:06

他又要我仔细考虑一下，然后我又跟父母沟通了一下，觉得可能还是在升值方面做一种所以是不是因为我政策其实我也是看了一下PPT而已，我想我想问一下是不是你你保研了以后必须要做升值方向的研究，是这样子吗？

38:26

是的。假如说你是考研的话，应该不影响是。不影响。然后我一旦进入了成绩的前40%拿到了保研资格，我就一定要进国重。现在其实我们大家对这个也不是特别清楚，按他们之前宣讲的来说的话，应该是要进国重的。你有没有跟周围同学聊就是聊过保研的东西？他们都愿意保。研直接进国中吗？有聊过这个事情，他们的想法各各种各样的都有吧就。

39:15

很多人比如说他们保研，你觉得最主流的想法是什么？最多的呢是，有的想法，他们可能会想拿着保研资格去更高的学校去进行科研工作，他们拿了保研资格，理论上应该是可以的，对吧？但是好像我听你说，好像国中的老师要求你们必须要在国中也没有要求，这个是没有硬性要求的。你拿了国中的保研资格，我可以去复旦大学继续做升职，是这个意思吗？你也可以不做升职，也可以做其他方向。我有点迷糊了。你刚刚说保研了必须要做升职是什么意思？

39:58

就是我保研如果是按照正常的流程，就是我们保研保升值方向的话是肯定要做升值的，是这个意思，基本上都是要保升值方向，如果在本校读的话，你们假如说进了成绩的前40%，然后是有可能会有保研资格，我拿到保研资格，要是保本校的话就必须要走升值。之前我是这样理解，后来好像又听其他同学说，也不一定就是说你可能晋升值它还有另外一个条，附加的条件不是5+1+3，这个是升值系统里的我们要整个流程是5+1+3，然后如果你要去，你想你突然你不想读生殖啊，你去假如你想读流行病了，你可能就不能按照5+1+3走，但5+1+3是博士，所以其实进入其实除非你要是非常想走博士那条道路的话，所以你可能必须要走升值，但是假如说我真的不想不想读，甚至我想读个研读了研以后，我自己再去考一个别的博士什么之类的，其实他就没有必要再去读，甚至是这个意思。

41:18

所以说对于那些想走5+1+3这条路的学生来说，他可能必须要留本校，还必须要读深圳，是这个意思吧嗯，但是对于其他来说，其他人其他的同学来说，他拿了保研资格，我可以留本校读别的，也可以去外校读别的，对，所以就无所谓了对。所以其实我听你的意思就是说你可能更想去走5+1+3这条路，是是这个意思吧？

41:44

因为我觉得我肯定是要读博士的，我觉得既然要做科研，肯定是要读博士了，所以5+1+3可能会更好一点。好在哪？它的时间更短，能更快的让我往更高更高层次的研究。

42:02

你有没有想过5+1+3，它虽然它的确是少了两年时间应该叫但是可能也会很难有有想过就会阻碍你继续走这条路的信心吗？就会挫败你继续是什么意思？你就是因为它很难，所以可能我还不如直接考考个研很很难就是我想知道我觉得我这个想法比较难表达你对生殖的热情，会不会会不会概括5+1+3它的优势其实我还在我这个还是比较混乱的，因为我刚承担一个阶段性的工作，还没有开始嘛，所以我并不知道其实如果我真正自己开始做升职了，会是一个什么样子。

42:57

所以其实你现在对于你自己本人对于升职的热情来说，你也很难去评估它。对，你不太清楚你到时候会不会对他非常感兴趣，或者怀着极大的热情去做。可以实现现在，只是说5+1+3路，摆在我面前我可以去走，那我就去走一下。虽然它可能会限制了我的方向，但是我还是会愿意去牺牲一下我的方向，就是牺牲一下我选方向的权利，去走5+1+3模式的路。

43:33

可以这么说吗。

43:34

其实我觉得现在还比较早，我还在大三嗯就是5+1+3，至少也得在推免资格出来之后才能知道走不走这个东西。然后我们现在讲的这些都是假设你已经获得了资格，你有一个选择的权利或者有选择的一个方案摆在你面前的时候，对于对于将来的你来说，我觉得还挺难去评估的。但我更倾向于我现在目前来说更倾向于留在本校继续读下去嗯，继续走5+1+3的。

44:14

虽然可能以后得做升职，我可不可以说就是，其实你现在对于自己科研的可能有极大的兴趣点，可能还没有发掘出来。

44:31

就是说其实导师让你做什么方向你都可以做。我现在只是想要习得他的一个研究方法而已，你要让我做什么方向，其实我都是愿意接受的，可以这么说吗？也不一定现在让我再去做，我之前大创那个方向我就不会愿意了大创是。化学材料是吧？还是想做医学类的？对生物医学类的对啊。

44:56

所以当时免疫的老师跟你讲了以后，就是说你继续做免疫这个方向可能会阻碍你去读5+1+3，这条路阻碍你去走这条路，所以你就思考了一下，你决定还是不要冒险，还是去保研。对于你周围的同学来说，其实大部分学生还是想走5+1+3这条路的。

45:21

我其实你的感觉了解的不多，我觉得他们对开对半开就另外一半是啥？相当于可能想去其他地方都想保研去其他地方，可能有一半的学生是想保研去其他地方，或者读其他方向，或者说另外一半可能想做5+123可能达不到一半，但是就是说想出去的人可能达不到1万，但是也占一定的比例了，想出去的还是少数人。

45:53

我觉得1/3会有应该1/3吧。大部分人是想去5+1+3，对，但是竞争很激烈啊。可能是吧跟他们了解的不是主要还是跟预防的同学了解的多一些。基础那边的同学其实交流不是很多。你们不是会合在一起上某些课吗？但是他还是他们坐在他们那一次我们坐在我这一组是这样的，所以其实你跟预防的同学交流的还是比较多的，预防的高中的班的同学。

46:38

我再提一个假设题，对，假设题你已经博士毕业了，你跟5+1+3这条路已经走完了，你通过研究生殖的某一个方面毕业了，你拿到了教职，就是在可能举个例子，就在我们学校拿到了教学岗，教学研究岗的职位，你可以独立自主地开展研究了，你会考虑再去回到你当时的免疫的方向去研究吗？

47:09

我觉得应该不会考虑了。因为之前已经研究了太多的，基础都在升值。对。你可以跟我讲一讲，你觉得入学入国重班以来最大的收获是什么？最大的收获嗯，我觉得还是学到了一些科研的思维。科研的思维是指什么？对一个科学问题的思考，怎样去应该怎样去研究这个问题？这种思维我觉得还是很重要的，虽然说我现在也学的不是特别好，至少会有一点点。

48:03

你有回想过你就是科研思维的习得是来源于哪里吗？是来源于实验室还是老师的上课，还是说是自己日常的操作还是什么方面，主要是老师的上课和跟老师和师兄师姐的交流。假如说你当时没有进入国中，进行普通的预防医学的那种班级的课程模式走的话，你觉得你还能习得科研思维这个东西吗？我觉得应该很难了，很难说。

48:38

对我因为我接触不到那些科研方面的导师，我不知道怎样去接触他们，我可能就跟其他同学一样，就是把课程学完，然后考研或者保研就这样做下去了嗯。

49:02

所以你其实我可不可以说你其实在平时对和你导师的交流其实还是挺多的，因为我听听你提导师提提的蛮多次的，跟有部分导师交流比较多。部分导师是比如我现在这个导师我会跟他有交流比较多，还有我的国中班的班主任，其实他也算某种意义上也算导师，然后我也会跟他交流比较多。

49:29

班主任有会带你们去做研究吗？他会让师兄师姐就是说他会带师兄师姐，那些博士跟硕士，然后那些人就在我们，某种意义上来说，他们也相当于是我们的导师，师兄师姐就带你们去做研究方法的学习。

49:48

你的班主任的那个是你带的那些博士生，他们带你学学这些他们嗯，带他们带他指导的那些博士生带我们科研方法，还有一些科研思维，这算是一门课吗？

50:04

这这不这就算实践科研轮转中的一部分，科研轮转中的一部分，所以你们的科研轮转都是班主任，来排啊也不是在工位，就是在也不算整个工位，就是说我大二下学期实验室，他那个实验室是体系的，因为我们班主任是那个实验室。

50:30

你跟你的班主任还有你现在导师交流比较多，可不可以？

50:39

换句话说，其实你很多的收获是来源于班主任和你现在导师来了国中班以后，很大程度上来说的话是来源于他们两个人，嗯也当然也有自己去看文献这种，也会获取一些知识。

50:58

自己开文献的话，是不是你提这句话，是不是因为觉得假如说在预防医学班级里去学那些理论课的话，可能就不会说自己去搜那些文献了。

51:11

对就是因为到了现在这个环境，对老师会让我们去了解文献，然后他会给我们一个大方向，然后我们自己去搜相关的文献去看，他并没有强制，但是我觉得这个对我可能有意义，我就去搜，然后去看他给你们一些方向是基于什么？是基于课题。你们要研究的课题吗？还是？大概他们觉得的意思并不是专，也不是一定是为了课题。某种情况下他说就是学的多，有些东西虽然现在没有什么用，但是对我的思维可能会有锻炼。

51:52

这个文件是吧？

51:53

对。然后他就随便提。提几个方向，然后你们自己去搜你们感兴趣的文件对。我对这个方向感觉我就说这个方向的问题。你在进入国中班以后，有没有什么让你比较不满的事情？不满嗯。我可能还得想一想，因为我现在没有什么印象，没有印象可能都挺满意的，对吧？应该是可以这么说。有没有什么让你感觉比较有压力的事情？压力就是我现在还行吗？

52:46

大二的时候压力会比较大，在学习跟科研上找不到一个好的平衡，你你有分析过原因，吗有有分析过原因，现在可以掌握平衡了，现在也没那么平衡，但是比大二要好，很多了。那是怎么改过来的，怎么好很多？我会在考试月来临之前，一小段时间就会跟我的导师说明情况，说我可能要考试要我，这段时间我就不来实验室做实验了。

53:20

然后我之前大二的时候就不是这样的，因为当时我也不知道是个什么情况，然后也是每天都去实验室看，然后我学习的时间，我我我这个人个人就如果我在做做实验，我可能就没有心思学习了，所以就把握不了平衡，现在的话我就会提前跟他说，我这段时间不来做实验了，我这段时间就专门用来学习了。所以这样就稍微好一点，大二的时候为什么不去说呢？是不好意思吗？还是也有不好意思的成分？你觉得最主要的是什么原因？还是说没想到还是也？

53:53

有没想到。也有不好意思，你觉得你你觉得当时为什么没有什么错呢？当时没有这么做，也想看一下时间，也想多学一点。你大二当时有有得不好的吗？大二有。

54:16

考的不好的，比如说像微生物这种课，我考的就没有其他人高。你觉得当时没有想到这个主要是因为什么？是没想到还是主干道还是不好意思，还是想我觉得想多学点东西。但是想多学一点东西，但是没想到可能影响到了自己的考试成绩。然后现在就已经可以去可以更好的平衡了对科研和学习。所以我可不可以说你现在其实压力比大二要小一点，对。

55:17

可以这么说。

55:18

但是课程就是在学习方面的压力，我觉得也不一定说小一点，虽然大二的课程多一些，但是其实我们现在学的这些课程中间有一部分是人文社科类的学科，我并不是很擅长，对。然后这一部分我不是很擅长，所以说我其实也要用相当于大人一样的时间来学的，就是更多的时间来学它。你说人文社科要花更多的时间来学它，我之前有采访别的同学说有听人说过，那个人文诗歌课他们觉得很水，主要是我个人不太擅长这方面的科目。

55:57

我从小就可以举个例子吗？什么你现在觉得哪门课很顺利，比如社会医学，还有这种卫生事业管理在哪里？我抓不抓不住他们想讲的内容的重点，还有一些记忆的东西，我可能并不能那么完好的把它记住，他会要背一些东西。然后那些其实我在背方面并不是很擅长，所以我要花更多时间。

56:26

社会医学好的，然后我现在想问的下面一个问题是可不可以跟我讲一个你觉得从进国中班以来，啊嗯你觉得做的最自豪的，让你最自豪的一件事情，或者让你最有成就感的一件事情，不一定去学习。

56:49

生活上面各方面都可以最有成就感，我不要限制在国中扮演，一从进大学以来，你觉得做了一件一件让你最有成就感或者最自豪的一件事情都可以，不一定是学习，也可以是科研，也可以是学习，也可以是生活，也可以是比如说社团啊什么的都可以。

57:13

没有。我就说一说最近太远了，我也记不清楚了说一下大三以来当时流行病学不是会有翻转课堂这种类型的学习方法，然后当时需要大家去一个小组去搜一些文献，搜一些前沿的进展，然后给他做一个类似于小科普一样给大家进行讲解，就是流行病学方向的。

57:41

然后当时是我们小组大概有十几个人，国政班的也有好几个，但当时大家好像都比较想划水的那种感觉。

57:51

这个时候我觉得这是一个锻炼的机会，然后我就会自己虽然我已经被安排了某一部分工作，但是我会在完成我自己工作的前基础上，我还要去帮别人做一下他们的工资，就是以我在国荣班学到的知识，比如说怎么样去搜一篇好的文献，怎么样把它文献的精华部分提炼出来，然后我就做了这样的工作，然后当时去小组演讲的时候，我们组可能老师特别青睐，然后就表扬了我们组，然后他们就会在群里说我就是辛苦之类的东西。

58:27

这个还是比较有成就感的，我可不可以说我听了这个故事的感受，是不是你觉得怎么讲？就获得了，别人的肯定对你来说是会非常让你有成就感，觉得一件事情我就这么说有时候会有有时候会有嗯嗯嗯，你是不是一个情绪起伏可能没有那么大波动的人，或者说你的情绪可能不会变化的那么距离因为我觉得就是问题，比如说最高兴或者最不满或者最有压力的事情，你好像都不太能说得出来的，感觉就是进国中班之后，可能不一定国中班就是大一以来我。

59:30

我想问一下你这个人是不是情绪起伏可能没有那么的剧烈，就不会有那么没有不会有特别兴奋或者特别高兴或者特别沮丧那种时候。我其实会有。会有对。我性格感觉挺奇怪的，就是有有时候如果天气好，我可能会心情就特别开心，然后做什么事情都感觉很开心。然后如果天气不好，我做什么事情也可能就感觉很沮丧，但有些时候并不会表现出来，只会在自己心里对自己有展呈现，就不会呈现给大家的，就是你不会把你的情绪给外露出来，大多数情况是不会的。

01:00:12

比如说你假如说遇到沮丧的事情，你会选择和别人倾诉吗？还是说自己自我消化掉？如果是程度不是很深的话，我就会自己消化掉，如果确实特别沮丧的话，我可能会跟我的朋友或者跟我的父母会有沟通。

01:00:31

有过吗？有真的有过这种经历吗？有非常沮丧，然后跟别人沟通。有过没有？女朋友。有女朋友。有你不跟女朋友沟通。现在他在学习，我们沟通的就少一点，以前会找他沟通。我说的是现在我跟他说的是现在。所以其实你对自己的情绪的记忆，我可不可以说其实你的情绪其实也也有很多的波动，只是说可能不进入自己记住这些特别开心或者是沮丧，我会很快就忘记他。

01:01:14

但是在好的事情我会记住，就是什么事情会让你印象比较深刻？印象深刻倒不至于就是说假如我对一个人，我看他交往，他可能会做一些我不是很支持，但是他也会做一些我特别让我满意的事情，我可能只会记住他对我的好的方面，在那坏的发票我都会过一段时间就忘记了，所以我相当于只会记住别人的好的方面。

01:01:47

你说的应该叫什么？叫形容是有具体的事例，存在是吗，你真的有人对你做过不好的信仰，最后你只记住它好的那一面。

01:02:00

有基本上我对大多数人都是这样的，比如我室友她大一的时候会打游戏，然后他敲键盘声音特别大，大家睡觉他也会这样子啊，我就特别恼火，就找辅导员，辅导员找他谈话，然后他父母跟他聊天，他都不听，然后这种这个事情大一的时候会特别让我苦恼了，嗯后来我就会苦恼，会让你苦恼到需要跟别人倾诉的地步吗？还是说自我消化？

01:02:33

偶尔跟女朋友提一两句，但是也不会过多的去说。当然后来我就跟我亲自自己跟室友讨论嘛，然后也有对他类似于有一点抱怨或者发火，然后后来到大二这件事情就基本上解决了。后来这之后我基本上好像没有发生过这种事情一样，就该跟他怎么相处就怎么相处了。

01:03:02

解决了是指他也不会再扮演小键盘。我跟我会在睡觉的时候跟他说一声我要睡觉了，然后他自己就知道了，我要学习他就不会这样子，他怎么会突然有这个转变呢？我也不知道可能是我对他可能抱怨发火了，然后他可能就觉得确实影响不好。可能他以前可能觉得我说这些都是一些玩笑。对。类似于这样的话，认真一下，他就觉得意识到问题了，你觉得下一个问题是。

01:03:43

我想问一下你你自己的可能之前也有跟你讨论过了，你觉得大学这几年对你来说，可能从大一开始一直到现在的本科到未来的硕士博士阶段，对你来说最重要的事情是什么呢？就在整个高等教育学习的阶段来说，最重要的事情对可以是生活上的，比如说做学生会主席，或者说拿第一或者说拿什么奖或者发什么文章，或者做什么实验，拿什么课题，你觉得这两个最重要的事情是什么？

01:04:18

仅仅就是在大学是吧？对，从上大学以来，包括到未来，我觉得还是我在我自己的领域有所成就，这才是最重要的事情。有所成就是指就是说他文章吗？还是说拿到学位，还是说你觉得成就的定义是什么？我想研究这个东西，我能把它研究出来，我发现他我就算它不是我预期的结果，但是我至少能把这个东西给研究透彻，我觉得这才是很重要的。

01:04:49

在我的领域中间能攻克一些问题，你觉得攻克问题的标志是什么？对你对你来说是你展现的一个研究成果，是把它发表了，还是说你拿到了这个结果，你就会觉得有有成就感，或者说你的成就感，你有没有想过自己成就感来源是哪里？这个问题肯定首先是我觉得其他的东西都是要在建立在我把结果给弄出来的基础上的。

01:05:26

至于发文章这个东西，我觉得只要我的结果是正确的，然后是对这个领域是有贡献的，我觉得发文章应该是最后大家共享给大家的一个途径，并要我做科研，其实我并不是为了发文章而做科研，我觉得还是可不可以换一个说法，说你做一个实验，跟你感兴趣的一个实验一个问题，你做出来的一个结果也获得了导师和周围你可能你的同事或者说你的博士生同学的同仁同仁的认可，但是可能结果发不出来，但是在这个时候你依然会很有成就感，因为毕竟你做下来的结果也受到周围人的认可，我可以这么说吗？

01:06:10

我现在是这样想的，也许以后会变得更现实一点。但我现在是这样想，我觉得没有关系，就是从你的情绪出发，我觉得比较实际一点，可能现实情况也会影响到你你的你的认知或者说感官，但是我觉得就从现在来说的话，讲讲出来就可以，讲出来你自己现在想法就可以。现在我还有一个还有一个问题，就是你现在是大三对吧？你在上大二进进那个活动班以后，有没有学弟学妹会问你一些关于股东办的问题？有很多嘛嗯也不能算很多，就拿现在19级国政班的有几个同学而言的话，大多数是预防的同学，他们一般都问啥？

01:07:01

啊他们一般都问什么问题就刚开始就会问一些国中班是什么，他跟大家一样不了解国中班，然后给他介绍一些基本情况，然后后来他会深入的问一下我在里面会学一些什么东西，然后我再跟他们说一下，然后我就会说如果你是真的想做科研的话，你这是个很好的选择，我但是这个东西还是看他自己去做决定，我就会这样跟他们说。其实大部分人在跟我，我也不知道是我对他们有没有影响，反正我认识的那几个人大多数还是报考的过程班，而且现在就在过程班里进行学习。所以他们更关心的是可以在国中班里面学到什么，是这个意思吗？也有会关注保研。保研的对。他们也会问这种问题啊，你觉得哪一种学生学弟学妹会多一点？哪一种就我觉得这两个都蛮多的。

01:08:03

而且有一部分人他是这两个问题他会同时都很关注。我既要学到一些东西，同时我也要抱怨对。其实大多数人是这种人嗯，你一定要让它区分哪个更重要，可能也没有太大的必要。

01:08:26

但是其实大家都是带着这种学习性的目的和功利性的目的，这些东西都有。对你来说你应该也是同事都有，我也同事都有，只不过谁更重要，谁次要这个问题。我觉得他们应该也是这样。假如说五中班当时到了你毕业那会儿，发现算了，我不问这个问题了，这个问题可能引导性太强了，你还有没有什么其他印象比较深刻的事情可以跟我讲一讲，没有的话就可以跟我说没有。

01:09:20

没有，我可以了解一下你加入了什么社团或者是学生会之类的组织吗？校内校外都可以。

01:09:34

社团我大一的时候有都是一些体育类的社团，比如网球社，一个网球社。

01:09:45

现在退了是吧现在？退了，因为为什么退了，因为我学不会学不会对我跟他们一起练过，然后也学过尝试过打了一个月，然后发现确实没有天赋。

01:10:00

你没有什么运动天赋是吧？不是我打篮球还行，但是网球是真不行。你有参加过类似学生会之类的组织吗？没有。当过班委算了班。委好像也算院里学生会的干部嘛，对。当过班委对。现在还是吗？现在不是。为什么不做啊？我觉得我当之前当的是文体委员。

01:10:26

这个就是大一的时候对当了一整年，后来我就觉得其实班委我当跟不当，其实文体委员做的东西其实并不多，我就决定反正进国中班学习跟科研都做了，然后就去干，学生干部就不打了。放下。所以你觉得我可不可以说是因为他可能做不做对你来说都没有什么影响？好像也没有这个单位对。对我觉得应该也没什么收获也。反而有可能会占用你时间，对。所以还不如退掉算了，对。

01:11:11

可以说一下你平时除了学习和科研以外有什么爱好吗？打篮球偶尔。如果那段时间学习或者科研压力比较大，我也会打游戏，就放松一下。你觉得在活动班里面有没有思考过可能对于那些预防医学的学生来说，会不会感觉很多时间都在实验室里，可能时间的支配自我可以支配的时间，相对于那些预防医学的学生来说可能没有那么多，有时候有其他的这种可能比较大的牺牲，有想过这些东西吗？

01:12:20

有想过。毕竟每天都要去按国政班的要求来说的话，是每周要去三次实验室。其实实际时间有规定吗？就去这个是根据你的导师来对你的进行要求的。然后这个是国政班自己的最低要求是这样的。然后我们实际上我像我个人而言，我基本上一个星期会去6次，周六会休息。然后你导师对你的要求是几次啊？导师其实没有要求。然后我一看自己，然后我是一个星期去6次，然后最近我跟导师说，我说可能要考事业了，因为我这周六就有考试了，可能要考试，我可能只做一部分时间，就要比以前要减半的时间要减半，所以去的时间不要不会。

01:13:11

那么勤啊相对于其他同学来说的话，就会晚上回宿舍，比如说刷朋友圈，我会看到他们到时候在那边吃玩之类的，就是假期的话，有时候也会想这件事情，就会想你假期也在学校。

01:13:29

一般寒暑假的话回家的时间其实也不多，长假也是这样的，平常周六周日的话，也就基本上周六休息一天，前周日也会工作。这样。这个是老板要求还是你自己要留在这儿？长假。长假是国中也有要求要一留一段时间，然后自己的要求的话是自己想学更多的东西，好的。其他的好像该看的都差不多了，我看一下。你在考试月的时候会有焦虑感吗？会。很强烈吗？还行。也不到不至于到那种要跟人倾诉的那种程度嗯。

01:14:31

你觉得从大一大二到现在这个比较的话，你觉得你的焦虑感是在增强还是在减弱，还是说持平的一种状况，你觉得是有起伏的，要根据当时的学习这也有影响。还有有就是说我那学期学了一些什么课程，这也是有影响。课程是指课程难度吗？对我的难度，比如说你之前采访的那些人，他们说人文社科类的很水，其实对我来说就不水了。我觉得这种我没有天赋的就得死记硬背，然后他们可能学的比较好一点，这种压力就会比较大了。

01:15:06

你不是不太擅长的科目，你可能会感觉交流感会更强一点，对。但是你擅长的话擅长就没有这么大，所以我可以说焦虑感是来源于可能是通过考试带来的，会有这种担心我还有一个假设性的问题想问一下，假设你有两件事情，一件事情是你你学会了你一直很想学习的一个科研的方法，或者说技巧，或者说一套学学习到了看完了一本书，就是学习到了一套，你一直想学习到的一个科研的理论，这件事情和你努力了很久，考到了班级第一这两件事情，哪件事情会让你觉得更有的成就感，或者说更有自豪感，或者让你更兴奋？

01:16:04

第一第一件事，我对成绩其实没有太大要求，我觉得只要满足国中班对我的要求就行，就是满足就能保研的那种，有些保研能到博士那种最低要求就可以。你在遇到一些比较有挑战性的任务的时候，最后做了以后，比如说做实验做失败了，你一般会分析他们的可能带来的原因，吗你会不会分析？一般分析原因的话会有可以给我举个例子吗？你做什么事情不一定。是实验只能做类似于做学生活动什么之类的，其他的任何一件事情，有挑战性的任务做失败了，然后你去分析它的原因。

01:17:13

简单一点，简单一点，影响比较深的。那就是我我还是讲实验，毕竟记得最深的还是实验。我刚学一个蛋，当时是第一次学一个蛋白质方面的实验，我当时连续做了两次都是同样的，结果都是很奇怪，就会多了很多的杂带。我不想要的那些杂志可能说嗯，然后我就一直都不知道是怎么原因，而且自己需要的挑战却没有出来，这样的一个情况。

01:17:50

后来就跟新闻事业他们讨论了，然后后来得出可能要么就是我在最开始的对我我想我想知道的是你在跟你师兄师姐讨论之前，你有自己分析过这个原因吗？就是没有在和外界交流。你自己分析的原因是什么？我可能有些操作不当，就比如操作不当，蛋白质实际上需要做封闭，然后我可能封闭的时间不够，导致其他的杂质很多，这是一个方面。

01:18:23

然后另外但我自己要的条件没有可能是因为我当时对这个细胞进行操作的时候，并没有让他表达出这个担子。O我只是想知道你自己的想法是什么就可以了。另外还有一个比较想知道的是你我想知道你害不害怕。

01:18:44

失败，比如说遇到一件难度非常大的任务摆在你面前，你有没有可能会因为考虑到他以后可能会难，因为难度太大，可能你做不到，就选择不去接这个任务，你觉得你是众人吗？你会害怕失败吗？害怕怎么肯定会有的，但是要看那个任务具体是个什么性质的话，我觉得如果真的他那种是一个可选择的一种可选择的就可做可不做的，如果我做这个任务，他我会得到一些什么呢？

01:19:20

那种比如说你的课题，讲讲讲的详细一点，你自己深深的一个做了一个一个课题的方向，在你选择的时候，这个方向和这个方向可能他它难度很大，但是很有可能会失败。

01:19:40

然后但是他做出来肯定是成就非常大的。这个方向可能它的难度是中等的，也有可能会成功。但他可能就是影响力可能没有第一个那么大，你可能会更倾向于是我目前这个状态，还是说以后以后你工作了，你要开始申请课题了。我是想了解一下你对于任务的选择的一个倾向，我觉得如果是我刚开始准备工作的话，还没有能做到在科研领域游刃有余的话，我可能会选择难度小一点，的那种。

01:20:15

先做出一点。对我觉得这个是要循序渐进的是有什么样的考虑吗？可以说一下吗？

01:20:27

考虑的话一方面说如果我刚开始做，我可能没有那么多的资源去做这个事情，然后我需要先通过一些完成一些小的工作，来把我自己的根基先扎稳，然后再往更深处的方向扎稳是指获得同行的认可吗？获得学校或者用你单位的我我认可。我觉得不是就是说在我、知识体系，还有对于课题的研究，我觉得这个研研究做多了才会有更新的想法。

01:21:02

明白。Ok我其他没有什么问题了。还有一个国中班是否达到你的预期，你觉得基本上能达到。进来以后，其实你刚刚跟我说的主要也是想和老师多接触多交流是吧？其实你到目前为止，我听李老师应该也做到这件做到这件事情，基本上是能达到我的预期了，所以学弟学妹来问你的话，只要他们是想做科研的，其实你也是会推荐的，对，但是不是不想做科研的话，其实就不太建议他们来这儿我个人倾向于不太建议，但是我也会把事实告诉他们，就是说确实会有这些政策。

01:21:50

比如说保研对嗯也是算优势了，可能你不喜欢科研，但是你来了还是有这个优势的，对就是忍着是的。Ok谢谢你啊，我去嗯那还有什么事吗？好，谢谢。请从那边出去一下，谢谢。
