# Supplementary material for: Why do you choose this program?—A decision-making model of medical students based on grounded theory
Source: PLoS One. 2023 Sep 15;18(9):e0291634. doi: 10.1371/journal.pone.0291634 (PMC10503722; doi:10.1371/journal.pone.0291634)
Supplement: S1 File — (ZIP) [file pone.0291634.s001.zip › RAW DATA/P4.docx]

00:00

But before this start I first in this interview, the respondents are in principle of equality and voluntary participation, respondents must truly express self ideas and cognition, confirm oneself meet the interview conditions, the interview process will be recording, the recording will be used in the form of anonymous for scientific research, not leaked to any third party, in the process of interview and interview, you have the right to cancel er researchers recording data, do you know and agree?

00:30

I agree. That is, now I want to ask your grade first, I am grade 18. Class 18. Is it excessive prevention? To. What major was you before you entered the prevention activities? Is to prevent. The first question I want to ask is that you will first talk about it, what did you know about the Chinese middle class in your freshman year? What are the channels to understand? I in fact understand the whole process of publicity, in fact, I began to know the national middle class from the second semester of my freshman year. Well. At that time is everyone in the organization of the big creation, and then I will listen to a classmate said, actually want to do scientific research is not necessarily through this way, maybe can consider to enter oneself for an examination the middle class, from that time I heard the first time have the middle class this name, then the end of the semester, there will be a teacher to come to preach back.

01:41

According to worry, everyone wait a little, is you just said that the classmate is your preventive medicine class classmate? So we will continue that and then he will come to preach, at that time is our level 18 national government class head teacher, is the teacher Gu to preach, and then he explained to us some of the basic situation of the national government class, we began to be interested in the jelly class, began to understand the jelly class.

02:11

Basically, understanding is one such process. Have you ever discussed this matter with the people in your home, including your classmates, teachers, and counselors? I must have discussed it with my parents. Then I told them about what the teachers had told us and my own opinions, and then my parents said he supported my decision, and then I decided to apply for the national middle class.

02:39

You and your classmates, teachers have not discussed much with your roommates. What do their roommates think, they think this is a very good way, because they all know that I am more interested in scientific research, so they also support me, they help ah, they do not meet the conditions of different conditions without them, or I think.

03:01

In high school, were you reported directly for prevention, or were you transferred to prevention? Our lake I am Hunan, so we apply for 6 parallel volunteer prevention, is in the middle of the volunteer, one is not adjusted. What is your choice for prevention? I can't remember that clearly. It seems like a 3 or a 4. When you were communicating, you wanted to study medicine, literature and science, and he said that you had applied for all your science and engineering, did you remember when you were in high school? I must want to learn medicine, but I have all reported. My father is also a medical-related major, so he has a deep influence on me. I have always wanted to engage in medical-related work.

03:47

So what else did you say, besides medicine? Also reported for such as Harbin Institute of Technology such engineering schools and science did not do. In science, this should be less, mainly college, engineering and medical. What considerations you applying for engineering at that time? Are you interested in it for yourself? Or are you also interested too? At that time, I was very interested in the whole science, physics, chemistry and biology, so I would consider these three aspects. But biology and medicine are the main considerations, and for engineering, medicine is probably one of your main directions.

04:35

At that time, your classmates also knew that you like scientific research. Did you attend a junior college after you knew about the Chinese middle class? There was still there. The registration began in the second semester of my freshman year. The country should be after the examination of the professional examination to have the examination, so the big innovation is to continue to do, until the sophomore year into the Cultural Revolution is still doing. Then it didn't work on.

05:03

What was the reason for that time? Is that our team members seem to be not good communication, and then everyone is more scattered, so we did not get together to do. There is no good function, because it can be a specific point is that it may be other professional is different, not good to get together is not professional, professional is about the same, there are not checked is also preventive, everyone is together.

05:26

But they may be too busy with other things and pay too little on it. As a result, I and another person in charge could not support it, with a total of 5 people, so I didn't do it at that time. Is Dachuang an innovative project or an entrepreneurial project? It should be an innovative and innovative project. You two of you and the other student are the main person in charge of the project. Well, then, but maybe the team members can support you before you can do it, because there may be too many tasks, or maybe there may be some problems with the topic at that time.

06:11

At that time, although we had instructors, but in fact, the teacher was also relatively busy, we actually did not meet many times, most of us need to rely on ourselves to explore.

06:20

Then only the two of us, we have a lot of aspects to consider, we also went to do the experiment, tried for about nearly a month, and then find all try not to come out, and then others give support is not very enough, so you try to try in the laboratory that the laboratory how do you contact? Is the head teacher at that time, he asked in our class group to have any students interested in this?

06:44

Part-time class teacher, right is part-time class tutor, part-time head teacher, head teacher right. The part-time head teacher is in his direction, and he has a subject. Have any students interested, to you and another student said you two interested, and then use this topic to set up a big innovation project right, what is the general content of this project can be briefly said.

07:09

I want to know what direction, I think it is more chemical materials, the material is the industry and Information Technology Institute they is not the station is unchecked, they are making a nanomolecular material. Test that aspect of the material to develop, but in fact and the medical poor quite far, it is an engineering, in fact, that material it can want again. It packs in the medicine we need and delivers it to the patient you need.

07:42

You are equivalent to the research material pairs. When you saw this project, were you very interested in it? Or am I just trying to try out scientific research? Because I see the topic may be the relationship with medical is not very big, he is a material, because I was not know about other areas, I only this one direction can try, I chose to try, freshman when you have tried similar research room, just have such a chance, everybody, and then students told you have jelly is such a way to do scientific research.

08:25

To do scientific research. This thing is where you think your passion for scientific research comes from, at home edification, or see what things this has, because my dad he is particularly interested in science, although he can't become a scientist, but he will buy me some science books, often let me see why your father cannot become a scientist, because he may be in college, he is not read technical secondary school to work directly, so his work may have nothing to do with scientific research, but he is particularly interested in science.

09:01

Well, then I'll buy some science books myself. For example, when I was in high school, I especially liked to read the books of immunology. Later, I would have some strange ideas. After I entered the Chinese middle class, he would discuss them to those teachers, because the immune teachers were Chinese teachers, and sometimes they would give me some advice.

09:26

Do they have any comments about your thoughts? Is these scientific research ideas, they just think that undergraduates just began to contact, there must be a lot of unrestrained ideas. He says the more this think, the better, but while many are impractical, there are always one or two meetings, some in that direction that could be studied. He said that he still encouraged us to think anyway, after you are thinking so much, are you in the freshman year, you should be a junior year now, right? In my freshman year, did I read so many books and do a great scientific research project.

10:06

At that stage, did you notice or feel a particular interest in a particular field? He said that at that time, there should not have been discovered, is to understand that after finishing the big innovation, or for the summer vacation of the freshman year, probably before the national middle class registration, that is to say, I should not have.

10:29

Should not be there yet, just said to be more interested in scientific research, but not particularly interested in which field, but at the end of your freshman year, you should have a chance to change your major, right? Have you reported you? Where is the report? I turned to pediatrics, pediatrics, right. I said if you pediatrics you turned in? Should be no. If you successfully go to pediatrics, would you report to the public? If you wouldn't include this, because you didn't have a specific view of scientific research, you were just interested in it, but compared to the prevention and clinical majors, you would still choose clinical practice.

11:16

If you applied for the college entrance examination at that time, it was still mainly clinical. Those professional you will be more interested in a point is right, did not report big clinical report pediatrics is like pediatrics? Or is it for the grades or for something else? At that time, my requirement of pediatrics was in the top 30%, but I did not reach 20%, so I could only sign up for a class if I could not apply for clinical practice.

11:46

So at that time after the end of the transfer of major, and then applied for the national middle class to the right, the valley middle class you are to participate in a lecture. In fact, it changes a lot of things, you think for you at that time, the biggest impact, or you are the most impressive or said that there is no special just think about doing scientific research, so to work. Well, when I summed it up at that time, I read his "p". The first one was that the scholarship was very high, and the second one was that he could directly contact the scientific research tutor from his sophomore year.

12:30

The third is the curriculum reform, that is, it deleted some courses, adds some characteristic courses, and characteristic construction internship. The fourth possibility is that he would be a little higher than ordinary prevention. The fifth is a 5 + 1 + 3 model, which of these features do you think is the most striking or the most attractive to you.

12:50

Can be. Is that the point of access to scientific research mentors? What else do you remember more about this? That was the time for the lecture session. Remember? If not, you can say no, and I think about it. I should not be particularly impressed. It's probably compared to research mentors, or a little more attractive to you, right. But if I ask you to tell which you appeal to.

13:32

Can you say that? I think still can speak, I think my first idea is that you say home is more support your idea, they are you small home environment, that is to say, including you volunteer the college entrance examination, or you say high school, well they you will be how will interfere with your own that idea? Or will it dissuade you, or suggest you some ideas? Or is it all up to you?

14:15

There are so many choices in your life, I think there are be choices. If you fill in a volunteer, they will be half a generation to dissuade half of the suggestions, and then first I choose some of my interests, then they pick from them, then give me advice, and then if I choose the wrong ones, he will dissuade me.such.

14:43

Does dissuasion mean when it is applying for high school, or the college entrance examination, or fill in? If I fill in a school that they don't think is very good, they will tell me that the school major may not work, it does not meet your future interests, for example, I tell them that I want to do scientific research, and then they say that the major can not do scientific research or reward, is what major they don't want you to report.

15:07

I remember this for a long time, but there was a discussion about it.

15:17

So at that time, you actually applied for medical and engineering, but also in you framed a range, they gave you a smaller range, and then made a choice, can you say that.

15:31

So your parents must be based on your will, and then you may have some guidance in such a family environment, you're a junior now, right?

15:52

In your sophomore year, you experienced a rotation called scientific research, did you?

15:58

To.

15:59

You can tell me about the research rotation.

16:02

4 Are you four mentors?

16:04

It seemed to be due to the epidemic, and only one tutor was chosen in the second semester. In theory, he had to choose two mentors in a semester, yes, but in the second semester, he chose only one right one and only one mentor, so you earned three mentors in your sophomore year.

16:24

To.

16:25

Three laboratories.

16:27

The mentor you have chosen and you have decided your research, right? Basically basically determined that your research direction and your mentor are the rotating mentor at the time?

16:36

fault. I can tell me how the mentor.

16:43

Now this tutor has rotated three laboratories on the foundation and the workers. And then I don't think I'm particularly interested in their research direction. Then later I have discussed this matter with me into the class is the national middle class head teacher, I hope he can give me a guide.

17:08

Then he told me, I now this mentor he said that this mentor is just here, he has some research directions, he is also relatively young, may have no generation gap with students like us. Then I introduced him to me, and said that it was good that I could try, so I chose the mentor, and then followed him, I found that he was actually a really good direction, which I was also very interested in.

17:32

If you say the first three mentors and you are interested, you can tell me about what direction it is? Nor can the first tutor say uninterested, They are doing immune, I'm what I told you about me before, High schools have a particular interest in immunization, So my first choice was for him, But I found out that he didn't really focus on reproduction, So I think if I take their lab to the subject of appreciation direction, I may not be able to make something or learn something, I decided to find mentors who specialize in promotion, With this consideration, Then, with the words of the latter tutor, I think they rotate our Chinese undergraduates to their lab, Their attitudes are also different.

18:14

Some like in the labor union side, they pay special attention to undergraduates, they think you and I our country middle class students come, we must well train them, and then they will give the special senior brothers to take us to do experiments.

18:28

Then like in the basic side, there are some mentor they may tend to guide graduate students with doctoral students, for undergraduates may not have so much energy, so when we stay there may have some he asked himself to have a look, is not too much benefit to us, I think, you said in the immune laboratory, you may not be able to appreciate this direction, right?

18:58

So you don't really feel very inclined to stay in this lab.

19:00

You should be on the sophomore round in the sophomore year, and you have been determined in the sophomore year, you should want to do this appreciation, right? He said that into this class is to ask you must do deep in this direction, this should be two aspects to consider, because since into the appreciation of the direction of the process, must be related to the appreciation of the content. Then in the second word, I also think that I haven't seen the appreciation over there yet, because the first choice is immunity, and then I want to see what the appreciation is really like.

19:30

I haven't seen the promotion yet, but I didn't go to their lab to see what they were doing, and I didn't mean to rotate it.

19:43

You think the trade union attached more importance to Russian students, and the conclusion was that they might find some senior brothers and sisters to lead you, and how to teach you such scientific research methods.

19:56

They not only teach scientific research methods, they also teach you some ideas.

20:00

For example, when I encounter this problem, how should I think about it, how to solve this problem?

20:05

Is it your problem in class, or you doing experiments?

20:08

The experiment is right, and that means that when you rotate, you can already do the experiments in their station lab.

20:15

Is your lab yourself a time task they have assigned to you?

20:19

Or the teacher assigned a time task, or they assigned time, and then senior sister let you do, he will teach us first, and then we will ask not to let us try this idea, and then they basically will meet you, and will look at you do, and then skilled to you, you can yourself you can undertake the experimental task, he will let you do, in the basic may not be able to do it?

20:48

At least I didn't do it in my sophomore year.

20:55

But maybe you're not interested in the lab direction of the two stations you rotate behind.

21:01

It is true.

21:02

Later, you chose the young boss, in fact, he was only in one semester in the union, of course, the next semester actually only rotated once, just stayed in the station, and then I changed to my new mentor, the sophomore laboratory is the direction of the second semester laboratory you are not interested in?neither. Because I think I think I have done certain experiments, and I think about undertaking the process of independently undertaking a short period of scientific research independently.

21:31

Then but there, maybe he is always in the senior brothers, that is, he took the guidance of you with you, and you only did the experiment, but you don't have a complete process, it is the process of scientific research, I want to try the whole process.

21:48

So I just changed this display.

21:51

I am curious, you have thought about why in the second semester sophomore laboratory has no way to undertake a project independently of, but after the new teacher, you can go to undertake the project, because the competition is too fierce or this thing actually I later I was considering I think my ability is enough well, but then I was sophomore second semester, now is junior next semester, I am junior second semester selected teacher, also experienced a third year last semester, that is also in the other mentor there.

22:25

Then you were also spinning in the first semester of your junior year. There were two spinning rounds in your junior year. After I saw more than it did, I found that my ability was not actually reached.

22:36

Blood spell.

22:39

It's not because I think I can and just do that. In fact, I don't take them to us, but temporarily let us do a stage job, because we don't feel the ability is achieved yet.

22:55

You had two more labs in your junior year, two hours in the entire third year of your junior year, and the entire third year was equivalent to your sophomore year, and you had five labs.

23:07

To.

23:11

So the lab you choose now is the junior rotation lab right.

23:17

Understand that the exclusion is actually excluded. Because you also say now, maybe in that case, you have not been able to undertake an experimental project or technology project independently, the child eliminated this factor, you are willing to go back to the sophomore year.

23:38

What do I say about that lab now, and my current mentor is actually related to my previous lab, so I often have to do experiments on both sides simultaneously. I remember to do it as my mentor, and I will go to do it in the second semester of my sophomore year. Oh, so they are actually connected. Doing what you do is a subject for both of them, and that's it.

24:06

In fact, it is not a topic, I just undertook a small stage of work. At that time, so you just said that you discussed with your head teacher, he recommended a new young mentor to you, this thing happened at the beginning of the second semester of the third year is the end of the first semester of the junior year, the end of the last semester of the last semester that rotation, before the winter vacation.

24:34

To.

24:37

I recommended this mentor to you, and then you think there is no generation gap, is it what he said or what you said?

24:44

That's what he means, and I understand that's what, because he says he's younger and so on.

24:54

You think it's pretty specific.

24:57

There can be something more specific.

24:59

He compared him sitting in the same laboratory with us, I basically sit close to him, I have any problem I can directly ask him directly, those even very small experimental problems, he will be very careful to answer to me, so the teacher can bring you a lot of guidance, so you think this is very good, you will be more concerned about the mentor help to you, or you also want to through the tutor to learn a lot of things, should be said.

25:41

To.

25:43

So can I say that you are actually not so important position for the postgraduate or postgraduate protection. Compared to research, it should be the second and it shouldn 't hold on, so you're doing the same part of your new mentor's project, right?

26:08

That should be true, and your research direction is just in keeping with him. In fact, I don't know very much about his research direction. I really don't communicate with him about these aspects, but I only know that my own direction is.

26:21

What are you doing right now?

26:23

Make a tumor-related gene, and then look at what it works.

26:29

Tumor-related genes seem to be special to Shenzhen, which are only expressed between the reproductive system and tumors, so we need to study the association between them, how do you feel? You are still collecting data and preparation, stage is not completely started, because this time I won't do the, I so I am now on the station is a second semester laboratory, in the learning the technology, after I wait I finished can start to do their own oh so, you are now sophomore second semester of laboratory technology is? To. Let the school career lead you, right? To.company.

27:30

Do you do now in fact I can I say a sophomore, actually you go to experience the laboratory an atmosphere call you learn, mainly may be sophomore second semester, senior senior taught you some means of scientific research, technology to last semester, may start to really learn more scientific research and technology, but so far has not independent bear a topic or a project, or a group of experiments, you can understand.

28:10

Is your progress common among your classmates? The students around you are basically in progress. No, there are some people who can do periodic work independently, and some people have already done a short period of work, as they had already done in their junior year.

28:31

Have you ever talked about it with them? We will have similar seminars, and then they will talk about what seminars they did in their lab, is it a lab seminar or your class seminar? It's the class. Then they talk about their progress, and some of them take the teacher's project independently, or two people do the same topic. For example, I have many stages of that topic, they have completed one or two stages of work, this also has.

28:59

I don't mean I envy them, I just be yourself. So you don't have any other emotions either. Listen to them talk, it won't affect you to me.

29:15

Listen to them is to look at him and learn what they are learning. Just get to know it. But the project is not mentioned, this kind of students will be more? Or is a relatively small number of people? I have already started producing an experiment in my third year. They must be a small part of the top people, I think most people are similar like you. I think it is.

29:40

The following question is that you have learned, I want to ask, the national middle class is indeed to do some curriculum reform, and then some even biology developmental biology you have learned? Reproductive biology has already been learned, but it has not yet been learned in development.

30:05

Have you ever learned that preventive medicine has been deleted, but you don't have to take those courses, have you ever learned about these courses? Have you ever learned about some of it? Do you have ideas or ideas about these deleted courses? Well, there is nothing to say, no. The idea is not there, is it? Do you think these reproductive biology lessons are helpful to you? Or what to gain?

30:33

There is certainly a harvest, they choose these courses actually I think for our reproductive activities certainly useful, such as reproductive biology, it is a lot of reproductive basic knowledge, long-term accumulation of research, well we will do promotion direction of scientific research, must be to understand the basic knowledge, so this lesson I think it is quite necessary.

30:58

Would it feel pretty interesting when you took this class, or so? I mean I want to ask you, do you have a strong interest in the last class? Or as long as a good class, as long as I do scientific research does not run away.

31:18

This should be just run for scientific research, because these classes are very basic class, does not bring some cutting-edge knowledge, after you from the sophomore year, and in the laboratory rotation, will have done some try, have tried to understand preventive medicine some other even outside the content of some disciplines?

31:37

There are also crowds and other things, too. Epidemiology, epidemiology, and statistician. You know anything about that, do you? Have understood is based on what kind of purpose, is interested to understand, or casually turn over to understand? I think, after all, I am still a professional thing in preventive medicine, and I think it is necessary to know what is said. When you are doing scientific research, or say I will change a question, you can tell me something that impresses you most. Since reading the country middle class, in so many laboratory rotation, the most impressive thing, or if you may not remember suddenly, have make you the most particularly happy one thing, or let you particularly dissatisfied with a what thing?

32:31

No dissatisfaction. Especially the deepest impression, actually the main I want to know a most impressive thing, the most impressive thing or I am to the class, I contact the first tutor of immunology, actually I read immune books in high school, because I know not the forefront of knowledge, so I can only produce some wild and unconstrained style ideas.

32:55

I had an idea until I first met the mentor, Then I talked to him about my idea, He told me that my idea was a particularly popular direction of my research over the years, Then I was particularly happy, My ideas are not completely unrealistic either, Occasionally, I can simply talk about my immune department, Human immune cells have a cell called a t-cell, He is involved in the human humoral immunity and cellular immunity, it can be said that the human immune system is also inseparable from the cells, Then I think the thought was our tumor, Because it tumors and it has an immune escape, That means our immune system can't clear him first, Sometimes you can't identify him, This time requires t cells to work.

33:49

And then how do we go to work? We put some of the molecules on the surface of the tumor and identify its t-cells directly in vitro, and then recycle it in the body, so that it will automatically recognize the tumor cells and kill it.

34:03

My idea is that I have been reading immunization books all the time, and then I discussed it with the teacher, and the teacher said that this is called car t. Then now the study is very hot, I was very happy at that time, on this matter should be the most happy thing, very happy. But I think you just said that immunization is immunization in your lab, and maybe he does not have much to do with reproduction, so you still decided to quit the immunization lab. Don't you think it is a pity? You think of high school and now this idea is almost close to, there is also one because of the national middle class is called promotion, I think this factor is quite complicated.

34:47

On the one hand, reproduction is the cause, after all, the national middle class should do the promotion direction, and on the second hand, I also think it is a little difficult to be immune. Although my idea sometimes that idea is right, it does not mean that I am good at doing it. I looked at the experiments they did, and I thought it was still very difficult.

35:07

Where do you think you have made a conclusion that you are not necessarily good at it to come from? Do you see something that you find difficult or to comfort myself, I tried to experiment, because I went in the lab, and sometimes I had to try to do the experiment. I found that I actually didn't do anything good at the immunization experiment, and then there was no confidence that was bad, the results were not right, and some of the operations were wrong. Then, although she told me that it had nothing to do with and would continue to study next time, I may not have built it up at that time, and my confidence may be like this.

35:50

So can I say that you are probably be a more pessimistic person who likes to take things in the bad direction.

36:02

Yes, because some people are very optimistic, some people are very pessimistic, or some people like to think about things in the good direction, and some people like to think about things in the bad direction, which one do you think you belong to? Most of it is a little more pessimistic. This will happen. Do you think it will give you a feeling of unconfidence? That's what you do and that should be called values. Sometimes it had an impact, and sometimes you probably did it in the immunization lab with an inappropriate operation that didn't work very well.

36:43

Is it a bit of frustration? Yeah, there was a sense of frustration. Is it strong? Anyway, occasionally think of that thing, and then finally change the lab? Have you ever thought, maybe I learned that method a little better, so it probably was good. I thought so later, after more knowledge, I think this is the beginning of the sophomore or sophomore article, I think this kind of thing is very common, as long as it to learn more and do more can do well.

37:22

So you goodbye, more, it is found that a lot of scientific research failure is very normal. Yes, when you get this conclusion, did you expect to return to immunization and continue to do Par? I have thought about and trying to contact the teacher. Then came the teacher who contacted the immunization, right? To. Later, he actually told me that there are also some requirements for our national middle class, which is similar to the graduate stage, which may be done related to reproduction, but it is not good at the joint field of reproduction and immunity, so he said that it may not be particularly good for my development.

38:06

He asked me to consider it carefully, and then I communicated with my parents, thinking that it may still be in appreciation, so is it because of my policy actually I also read PPT, I think I want to ask if you after you must do research on the direction of appreciation, is it like this?

38:26

yes. If say you are take an examination of grind of the word, should not affect is.do not affect. Then once I enter the top 40% of my grades and get the postgraduate qualification, I must enter the country. In fact, we are not particularly clear about this now, according to what they have said before, it should be to enter the country. Have you ever talked to your classmates about protecting graduate school? They are all willing to protect them. Is the research going directly into the country? Having talked about this, they have all kinds of ideas.

39:15

Many people, such as they protect the graduate school, what do you think is the most mainstream idea? At most, some ideas, they may want to go to a higher school for scientific research work, they get the postgraduate qualification, theoretically it should be possible, right? But it seems that I listen to you say, as if the teacher in the country asked you to also must not be in the country, this is no hard and fast requirements. You took the national postgraduate qualification, I can go to Fudan University to continue to do a promotion, is this meaning? You can do without promotion or in other directions. I'm a bit confused. What did you just mean to have a promotion?

39:58

Is I protect grind if it is in accordance with the normal process, is our protect grind protect appreciation direction is sure to do appreciation, this means, is basically is to protect appreciation direction, if read in our school, if you said into the first 40% of the grades, and then there is likely to have protect grind qualification, I get protect grind qualification, if the school will have to go appreciation. I understood that before, Then I seemed to hear the other students say, It doesn't necessarily mean you might be worth it has another bar, The additional condition is not a 5 + 1 + 3, This is the appreciation system, we want the whole process is 5 + 1 + 3, Then if you are going, You think you suddenly you don't want to read reproduction ah, You go, if you want to read an epidemic, You may not be able to follow the 5 + 1 + 3 walk, But the 5 + 1 + 3 is a PhD, So unless you really want to take the doctor's path, So you may have to go up in value, But if I really don't want to read it, I even want to read a graduate school, I'll take another doctor or something, There was no need for him to read it again, Even that, as it means.

41:18

So for those who want to walk 5 + 1 + 3 students, he may have to stay this school, also must read Shenzhen, is this meaning well, but for other, other students, he took the postgraduate qualification, I can stay this school to read other, can also go to read other school, right, so it doesn't matter right. So what I hear you mean, you might want to go 5 + 1 + 3, and that's it?

41:44

Because I think I definitely want to get a PhD, and I think since I want to do scientific research, I definitely have to get a PhD, so the 5 + 1 + 3 may be better. What's good? It takes a shorter time and leads me to a higher level of research faster.

42:02

Have you ever thought about 5 + 1 + 3, although it should be called after two years away, it may be difficult to have the confidence that it will prevent you from going this way? What does it mean to defeat you by continuing? You are because it is very difficult, so maybe I might directly test a grind is hard to know that I think I think this idea is difficult to express your enthusiasm for reproduction, will not summarize 5 + 1 + 3 its advantages actually I still in my this is chaotic, because I just take a stage of work, has not started, so I don't know actually if I really began to do a promotion, what will be a look.

42:57

So it's hard for you to assess for your own enthusiasm for promotion. Yeah, you don't really know if you'll be very interested in him, or do it with great enthusiasm. Can achieve now, just say 5 + 1 + 3 road, put in front of me I can go to go, then I will go to go. Although it may limit my direction, but I will still be willing to sacrifice my direction, is just to sacrifice my right to choose the direction, to go to the 5 + 1 + 3 mode road.

43:33

Can you say that.

43:34

In fact, I think it is still relatively early now, I am still in the junior year is 5 + 1 + 3, at least also have to push after the qualification out to know can not walk this thing. And then what we're talking about now is all about assuming that you're qualified, that you have the right to have a choice or a choice in front of you, and I think it's hard for you to evaluate. But I prefer that I am more at now inclined to stay at our school, well, go on 5 + 1 + 3.

44:14

Although you may have to get promoted in the future, can I say that, in fact, you may have a great interest in your own scientific research right now, which may not have been discovered yet.

44:31

That means you can actually do whatever the mentor asks you to do. Now I just want to learn one of his research methods, you want me to do what direction, in fact, I am willing to accept it, can you say so? Do not necessarily let me do now, I before big that direction I would not be willing to big is. Chemical materials, right? Or do you want to do medicine? For the biomedical class, right.

44:56

So after the immunization teacher told you, that is, you continue to do the direction of immunization may prevent you from reading 5 + 1 + 3, this road prevents you from taking this road, so you thought about it, you decided not to take a risk, or go to graduate school. For the students around you, most students still want to take the 5 + 1 + 3 road.

45:21

I don't know much, I think they are the other half? Equivalent to may want to go to other places all want to go to other places, maybe half of the students are to want to go to other places, or read other directions, or the other half may want to do 5 + 123 may reach less than half, but just want to go out may reach less than 10000, but also accounted for a certain proportion, want to go out or a few people.

45:53

I think 1 / 3 will have what should be 1 / 3. Most people just want to go to 5 + 1 + 3, yes, but the competition is very fierce ah. Maybe it is more about them mainly or the students of prevention. In fact, the basic students over there do not communicate very much. Don't you get together for some classes? But he is still they sitting in them that time we sat in my group is like this, so in fact, you communicate with the students of prevention or more, prevention of the high school class students.

46:38

I mention a hypothesis, yes, assuming you have graduated from Dr, you with 5 + 1 + 3 this road has finished, you graduated through the research of one aspect of reproduction, you got the faculty, is in may, for example, in our school got the teaching, teaching research post, you can be independently research, you will consider to return to the direction of your immunity to study?

47:09

I don't think it does so. Because too much has been studied before, the foundation is appreciating.To. You can tell me, what do you think is the biggest harvest since entering the national school? The biggest harvest is well, I think I have learned some scientific research thinking. What does the thinking of scientific research mean? How to think about a scientific problem, and how to study this problem? This kind of thinking I think is still very important, although I am not learning very well now, at least a little bit.

48:03

Have you ever recalled where your acquisition of scientific research thinking comes from? Whether it comes from the laboratory or from the teacher's class, or from their own daily operation or from what aspects, it is mainly from the teacher's class and the communication with the teacher and the seniors. If you say that you did not enter the country, and conduct the class curriculum mode of ordinary preventive medicine, do you think you can still learn the thing of scientific research thinking? I think it should be hard, it's hard to say.

48:38

Because I can't have access to those scientific research mentors, I don't know how to contact them. I may be just like other students, just finish the course, and then take the postgraduate entrance examination or postgraduate guarantee.

49:02

So you actually I can say that you actually have a lot of communication with your tutor in ordinary times, because I have listened to your tutor mention quite many times, and I communicate with some mentors more. Some mentors are, such as my current mentors, I will communicate with him more, and my national middle class teacher in charge, in fact, he is also a mentor in a sense, and then I will communicate with him more.

49:29

Will the head teacher ever take you to do the research? He would ask the seniors to say that he would take the seniors, the doctors, and the masters, and then the people are in us, in a sense, they are also our mentors, and the seniors would take you to learn the research methods.

49:48

The one in your head teacher is the doctoral students you take, they take you to learn them, the doctoral students they take them to guide our scientific research methods, and some scientific research thinking, is this a course?

50:04

This is not this even if the practice of research rotation, part of the research rotation, so your research rotation is the teacher in charge, to row is not in the station, is not the whole station, that is to say my sophomore second semester laboratory, his laboratory is the system, because our teacher in charge is the laboratory.

50:30

You communicate with your head teacher and your tutor more now, can you?

50:39

In other words, in fact, a lot of your harvest is from the head teacher and your mentor now came to the national middle class, to a large extent, the words are from them, of course, also have their own to read the literature, will also get some knowledge.

50:58

If you open the literature by yourself, do you mention this sentence, whether because do you think that if you say in the preventive medicine class to learn those theoretical classes, you may not say that you will search those literature.

51:11

Is because to the present environment, the teacher will let us to understand the literature, and then he will give us a general direction, and then we go to search the relevant literature to see, he is not forced, but I think this may be meaningful to me, I will go to search, and then to see him give you some direction is based on what? Is based on the subject. Are you going to study the topic?still? Perhaps what they think does not mean is specialized, nor is it necessarily for the subject. In some case, he said that he had learned more. Although some things are not useful now, they may exercise my thinking.

51:52

Is this the file?

51:53

To. Then he mentioned it casually. Take a few directions, and then search the documents you are interested in. I feel about this direction and I just talk about this direction. Do you have anything dissatisfied with after you entered the national middle class? Dissatisfied well. I may have to think about it, because I don't have any impression now, and I may be quite satisfied, right? It should be said. Is there anything that makes you feel more stressful? Is the pressure just that I can do it right now?

52:46

In the sophomore year, the pressure will be relatively great, and I can not find a good balance between study and scientific research. You have ever analyzed the reasons, and now you can master the balance, which is not so balanced now, but it is better than the sophomore year. How did that change it, how much better? I will explain to my tutor for a short time before the exam month comes, saying that I may want me for the exam, and that I will not come to the laboratory to do experiments.

53:20

Then before I was a sophomore is not like this, because I don't know what is a situation, and then go to the laboratory every day, then I study time, I, I personally I this person if I do doing experiments, I may have no idea to learn, so can grasp the balance, now I will tell him in advance, I this time to do experiments, I have this period of time is dedicated to study. So it's a little better, so why not say it in your sophomore year? Are you sorry? Or is there an element of embarrassment? What do you think is the main reason? Or is it either unexpected or too?

53:53

Did not expect. Sorry, too, why do you think you think there was nothing wrong with you? I didn't do this at that time. I wanted to see the time and learn more. Did you have a bad thing in your sophomore year? Sophomore.

54:16

Not doing well, such as microbiology, I was not as high as others. What do you think you didn't think of this as mainly because at the time? Did not think of or the main road or embarrassed, or think I think I want to learn more things. But I want to learn something more, but I did not think it may affect their test results. And now it can be a better balance between research and learning. So can I say that you're actually under a little less pressure than your sophomore year, right.

55:17

So to speak.

55:18

However, the course is the pressure in study, and I don't think it is necessarily smaller. Although there are more courses in my sophomore year, in fact, some of the courses we are taking now are humanities and social sciences, and I am not very good at them. And then I'm not very good at this part, so I actually have to learn it in the same time as adults, just spend more time to learn it. You said that the humanities and social sciences should spend more time to learn it. I have interviewed other students before and said that the humanities and poetry class was very popular, mainly because I am not very good at this subject.

55:57

Can I give me an example since I was a child? What do you think is well, like social medicine, and where is this health management? I can't grasp the focus of what they want to say, and some memory things, I may not be able to remember it so well, he will have to carry something. And then those I'm not really good at, so I spend more time.

56:26

Social medicine is good, and then I now want to ask the following question is whether can you tell me a you feel since the national middle class, ah, you feel the most proud to do, let you the most proud of things, or let you have the most sense of achievement of things, not necessarily to learn.

56:49

Life above all aspects can have the most sense of achievement, I don't limit in the country, a since the university, you feel made a one let you the most fulfilling or the most proud of one thing can, is not necessarily learning, can also be scientific research, can also be learning, but also can be a life, it can also be such as club ah what can be.

57:13

not have. I said too far recently, I also can't remember the said since the junior year epidemiology is not flipped classroom this type of learning method, and then need to go to a group to search some literature, search some frontier progress, and then give him a similar to small science to explain, is the direction of epidemiology.

57:41

Then there were about a dozen people in our group, and several people in the national administration class, but everyone seemed to want to paddle the water.

57:51

This time I think this is an opportunity to exercise, and then I will myself although I have been arranged a part of the work, but I will finish my own work, I have to help others do their salary, is I learned in honor class, such as how to search a good literature, how to extract the essence of the literature, then I did this work, and then went to group speech, our group may teacher special favor, and then praised our group, and then they will be in the group said I am hard.

58:27

This is still a sense of achievement, can I say how I listened to the story, do you think how to say? Get, others must be for you is very let you have a sense of achievement, feel one thing I say sometimes there are sometimes have mum, well, you are a mood ups and downs may not be so big fluctuations, or your mood may not change so distance because I think is a problem, such as the most happy or the most dissatisfied or the most stressful things, you don't seem to say, feeling is into the middle shift, may not necessarily the middle class is a freshman since me.

59:30

I want to ask you if the emotional ups and downs may not be so intense, and there will not be so so excited or so happy or so depressed. I'll actually have one. There will be right. My personality feels very strange, but sometimes if the weather is good, I may be very happy, and then I feel very happy to do everything. Then if the weather is bad, I may feel very depressed about what I do, but sometimes I don't show it, only show myself in my heart, I will not present it to everyone, that is, you won't show your emotions out, most of the case is not.

01:00:12

For example, if you say that you encounter depressed things, will you choose to talk to others? Or do you digest it by yourself? If it's not very deep, I will digest it myself, and if I'm really depressed, I may communicate with my friends or with my parents.

01:00:31

Have you ever? Have you really had this experience? Have been very depressed, and then communicate with others. Have you ever?girlfriend. Have a girlfriend. If you don't communicate with your girlfriend. Now he is studying, we communicate a little less, we used to communicate with him. I was talking about now and I tell him about now. So in fact, your memory of your emotions, I can I say that in fact, your emotions also have a lot of fluctuations, just said that you may not enter yourself to remember these are particularly happy or depressed, I will soon forget him.

01:01:14

But in the good things I will remember, is what things will impress you? Impression is not to say if I to a person, I see his contacts, he may do some I am not very support, but he will do something I particularly make me satisfied, I may only remember his good aspects of me, in the bad invoice I will be after a period of time to forget, so I will only remember the good aspects of others.

01:01:47

What should you call it like? There is a specific example, yes, you really someone did a bad faith in you, and finally you only remember its good side.

01:02:00

Basically I to most people are such, such as my roommate her freshman will play games, then he hit the keyboard sound particularly big, he will sleep like this, I was particularly angry, just find a counselor, counselor to talk to him, and then his parents chat with him, he didn't listen to, then this thing freshman will let me upset, well then I will distress, will let you distress to need to talk to others? Or is it self-digestion?

01:02:33

Occasionally talk to my girlfriend, but also won't say too much. Of course, then I talked to me with my roommate myself, and then I complained a little or got angry about him, and then it was basically solved in my sophomore year. After that, I basically hadn't happened, so I should get along with him.

01:03:02

It means that he won't play a small keyboard anymore. I told him that I had to go to bed, and then he knew it himself, and if I wanted to learn that he would not be like this, how could he suddenly have this change? I don't know if maybe I might get angry about him, and then he might think it didn't work well. Maybe he used to think that I said these were some jokes. To. Things like this, seriously, he feels aware of the problem, you think the next problem is.

01:03:43

I want to ask you if your own possibilities have been discussed with you before. Do you think, what is the most important thing for you from the beginning of the freshman year to the future master's degree? In the whole stage of higher education, the most important thing can be in life, such as being the president of the student Union, or taking the first place, or taking what awards or sending what articles, or doing what experiments, or taking what topics, what do you think are the two most important things?

01:04:18

Just in college, right? Yes, since college, including in the future, I think I've done something in my own field, and that's the most important thing. Does something mean his writing? Or do you get a degree, or what do you think is the definition of achievement? I want to study this thing, I can study it out, I found that he I even if it's not the result I expected, but I can at least study this thing thoroughly, I think that's very important.

01:04:49

To overcome some problems in the middle of my field, what do you think is the sign of overcoming problems? Is it a research result for you to show to you, do you publish it, or do you get this result, and you will feel a sense of achievement, or your sense of achievement, have you ever wondered where your sense of achievement comes from? This question is certainly first and foremost that I think everything else is based on what I get the results out.

01:05:26

As for sending out this article, I feel that as long as my results are right, Then there is contributing to the field, I think sending an article should be a way to share with you at the end, And I asked me to do scientific research, I'm not really doing scientific research to post articles, Do I think I can say another story, Say that you do an experiment, One question, with an experiment that you are interested in, One result you made is also recognized by your supervisor and your possible colleagues or your doctoral students, But the result may not be sent out, But you will still feel very fulfilled at this time, Because after all, you are recognized by the people around you, Can I say that?

01:06:10

I think now, maybe a little more realistic later. But I think so now, I think it is irrelevant, just from your emotions, I think more practical, may also affect your reality of your cognition or senses, but I think from now on the words, speak out, speak out your own thoughts can. Now I still have a question, that is, you are now a junior year, right? After you entered the activity class in your sophomore year, did you ask you some questions about the shareholders? There are a lot of well also can not calculate a lot of, take now the 19 grade national political class has how many students for words, most of the prevention of students, they generally ask what?

01:07:01

Ah they generally ask what questions will ask some what is the middle class, he don't understand the middle class, and then introduce him some basic situation, and then he will deeply ask me will learn something inside, and then I tell them, then I will say that if you are really want to do scientific research, you this is a good choice, I but this still see his thing to make a decision, I will say to them. In fact, most people are working with me, and I do not know whether I have influenced them, anyway, most of the first and a few people I know are still registered for the process class, and now in the process class to study. So they are more concerned about what they can learn in the middle class, Does that mean? There will also pay attention to the insurance research institute. The right to protect research. They also ask these questions, which kind of student do you think will be more junior? I think there are a lot of both.

01:08:03

And there are some people who are very concerned about both issues. I have to learn something, and I also have to complain right. In fact, most people are this kind of people, well, you must make it distinguish which is more important, and may not be too necessary.

01:08:26

But in fact, everyone is with this learning purpose and utilitarian purpose, these things have. For you, you should also have both your colleagues, and I also have all my colleagues, but who is more important, who is secondary to this problem. I think they should be the same. If say five middle class at that time to you graduate at that time, find forget it, I do not ask this question, this question may be guiding too strong, you still have no other more impressive things can tell me about, have no words can say with me to have no.

01:09:20

No, can I know what club you joined or an organization like the student union? Both on and off of school.

01:09:34

Association When I was a freshman, there were some sports associations, such as a tennis club, a tennis club.

01:09:45

Now you back it, right now? Back, because why quit, because I can't learn not to allow me to practice with them, and then also learned to try to play for a month, and then found that there is really no talent.

01:10:00

You don't have any athletic talent, do you? I play basketball, but tennis is not good. Have you ever participated in an organization like the student union?not have. When the class committee to forget the class. The committee also seems to be the cadre of the college student union, right. When the class committee is right. Is it still? Not now. Why not do it? I think I was a commissioner of sports and sports before me.

01:10:26

This is the freshman year when a whole year, then I felt that the class committee I when with improper, in fact, the sports committee member to do things are not much, I decided to anyway into the middle class study and scientific research are done, and then go to do, the student cadres will not play.lay down. So do you think I can say that it is because he probably does nothing to you? It doesn't seem to have this unit, right. I don't think I should have much to gain. It could take up your time, right. So you might as well get back, right.

01:11:11

Can you say what hobbies you usually have except for study and scientific research? Playing basketball occasionally. If there was a lot of study or research during that time, I would play games and relax. Do you think in the activity class may think for those preventive medicine students, will not feel a lot of time in the laboratory, may dominate self time can control time, relative to those preventive medicine students may not be so much, sometimes have other this may be a big sacrifice, have thought about these things?

01:12:20

Have thought about. After all, every day according to the national administration class requirements, is to go to the laboratory three times a week. Is there an actual time requirement? Just go to this is to ask for you according to your mentor. Then this is the minimum requirement of the national administration class itself is such. Then we actually I personally, I basically go 6 times a week and get off on Saturdays. And then how many times did your tutor ask for you? The tutor didn't actually ask for it. Then I look at myself, and then I go six times a week, and then recently I told my tutor, I said I might take a career exam, because I have an exam this Saturday, may have an exam, I may only do part of the time, the time to half, so the time should not go.

01:13:11

Compared to other students, they will go back to the dormitory in the evening, such as checking the circle of friends, I will see them eating and playing there, that is, during the holiday, sometimes they will think about this thing, they will think you are in school during the holiday.

01:13:29

General winter and summer vacation words to go home time is actually not much, long vacation is also like this, usually Saturday and Sunday words, also basically Saturday rest a day, before the Sunday will also work.so. Is this the boss asking for or do you stay here yourself?long leave. Long holiday is the country also has a request to stay for a period of time, and then their own request is their own want to learn more things, good. Everything else seems to be similar, so I'll take a look. Do you feel anxious during the exam month?get together. Is it strong?not too bad. It's not to the extent that you have to talk to people.

01:14:31

If you think from your freshman or sophomore years, do you think your anxiety is increasing or decreasing, or that there are ups and downs, based on your study at the time. There are other courses that I learned that semester, which also has an impact. Does the course mean the difficulty of the course? For my difficulty, such as the people you interviewed before, they said that the humanities and social sciences are very water, but actually it is not water for me. I think I don't have the talent to memorize it by rote, and then they may learn a little better, and the pressure will be greater.

01:15:06

You're not a subject that you are not very good at, and you might feel a little better about communicating, right. But what you are good at is not that big, So I can say that the anxiety comes from probably coming through exams, There will be this concern and I have another hypothetical question to ask, Suppose you have two things, One thing is that you have learned a scientific research method that you have always wanted to learn, Or rather, the technique, Or a set until you read a book, Just learned a set, A theory of scientific research that you have always wanted to learn, This matter and you have tried very hard for a long time, I got the first thing in the class, What makes you feel more fulfilling, Or even more proud, Or to make you even more excited?

01:16:04

First, the first thing, I actually do not have too big requirements for grades, I think as long as to meet the national middle class on my requirements, is to meet the kind of postgraduate protection, some postgraduate research can go to the doctor of the minimum requirements can be.When you encounter some more challenging tasks, and finally do them, such as doing the experiment failure, you will generally analyze their possible reasons. Will you analyze them? Can you give me an example if you generally analyze the reasons? You do not necessarily do what you do. The experiment can only do something similar to doing student activities, but any other thing, and the challenging task fails, and then you will analyze the reason of it.

01:17:13

Simple, simple, the impact is relatively deep. That is, I still talk about the experiment, after all, I remember the most is the experiment. I just learned an egg, and it was the first time to learn a protein experiment. I did the same thing twice in a row, and the results were very strange, so there were a lot of miscellaneous bands. The magazines I didn't want might say well, and then I just didn't even know why, and the challenges I needed didn't come out.

01:17:50

Then I talked about journalism, and then I came out that either I told me at the beginning. I think what I wanted to know was that did you analyze your own reasons before you discussed it with your senior brothers? I just didn't communicate with the outside world. What are the reasons that do you analyze by yourself? I may have some improper operation, such as improper operation, the protein actually needs to be closed, and then I may not close for enough time, leading to a lot of other impurities, which is one aspect.

01:18:23

Then another, but I can't ask for it myself, because when I manipulated the cell, I didn't ask him to express the burden. O I just want to know what your own thoughts is. Another more want to know is that you and I want to know if you are not afraid of harm.

01:18:44

Failure, for example, when you encounter a very difficult task in front of you, do you possibly choose not to take the task because it may be difficult in the future, and because you may not be able to do it. Do you think you are a crowd? Are you afraid of failure? How certainly there will be fear, but it depends on the specific nature of the task, I think if he is really a choice of a choice can do it, if I do this task, what will I get?

01:19:20

For example, your topic, talk about a little more detailed, you yourself deeply do a topic of the direction, when you choose, this direction and this direction, it may be very difficult, but it is likely to fail.

01:19:40

And then, but he certainly did it as a great achievement. This direction may be of in difficulty, or it may be successful. But he may be that the influence may not be as big as the first, you may be more inclined to so my current state, or say that later you work, you will start applying for a project. I want to understand your tendency to choose tasks, and I think if I just started preparing, if I was not yet comfortable in scientific research, I might choose the less difficult one.

01:20:15

Make a little bit first. What consideration do I think this is about going step by step? Can you say that?

01:20:27

On the one hand, if I just started doing it, I might not have so many resources to do it, and then I need to do some small work first, to stabilize my own foundation first, and then go deeper down, is it meant to get the recognition of my peers? I recognize the school or your unit. In my opinion, it is not that in my knowledge, the knowledge system, and the research on the subject, I think the research will have more updated ideas.

01:21:02

clear. Ok. I have no other problems left. There is also a national middle class whether to meet your expectations, you think basically can meet. After coming in, in fact, what you just told me is to contact with the teacher, right? Actually you so far, I listen to teacher li should also do this do this thing, is basically can meet my expectations, so junior junior to ask you, as long as they want to do scientific research, actually you will recommend, yes, but not don't want to do scientific research, but actually don't really suggest that they come here I personally tend not to suggest, but I will tell them the fact that it will indeed have these policies.

01:21:50

For example, the guaranteed graduate school is also an advantage, you may not like scientific research, but you come to still have this advantage, right is to endure yes. Ok thank you, I'm there? Ok, thank you. Please get out over there, thanks.
