# Supplementary material for: Why do you choose this program?—A decision-making model of medical students based on grounded theory
Source: PLoS One. 2023 Sep 15;18(9):e0291634. doi: 10.1371/journal.pone.0291634 (PMC10503722; doi:10.1371/journal.pone.0291634)
Supplement: S1 File — (ZIP) [file pone.0291634.s001.zip › RAW DATA/P5 CHINESE.docx]

00:00

影响比较大的事情，都可以说今天也是一个聊天，大概是这么一个过程，这是我们访谈的一个目的，在访谈之前我先和你唉先确认一下实验道德伦理须知。在本次访谈中受访者是在平等自愿的原则上参与的，售房者必须真实的表达自我想法和认知，确认自己符合社保条件，访谈的过程会被录音，但是其录音资料仅会以匿名的形式用于科研，并不会泄露给任何第三方。

00:37

在访谈的过程中及访谈结束以后，受访者都有权取消研究人员录音资料的使用权，你是否知晓并知晓？同意。你是117级的预防医学是吧？两个。我来补充那么首先想要了解一下你在进入国中班之前，你是通过什么渠道了解到国中班的吗？因为当时他通过微信公众号宣传什么的，招生然后我就看到了。你主要就是通过微信公众号了解到的吗，了解的程度，你现在想一想觉得当时自己了解的。

01:28

当时了解程度只是看到他在公众号上说一些招生的条件，还有以后培养的一些计划，但是当时只是一种提纲式的这种宣传了解程度，并不是非常的但是我记得你们是有开宣讲会的，你们有没有参加的宣讲？

01:52

在宣讲会上，你主要的信息来源居然是来源于公众号，不是来源于因为宣讲会上它的主要内容也是跟公众号上有大部分是重合的，所以你首先是从公众号了解的，你觉得了解到的那些关于国中班的有哪些方面是比较吸引你的，吸引你到多少班？

02:20

第一方面是它生殖医学是国家重点实验室的平台，然后起点就比较起点是比较高的。其次就是它的培养方案可以让本科生像研究生一样接触那些实实在在的接触科研。这一方面这两个方面是主要吸引我的。其次它的质量方案，硕士研究生期间，他对硕博的对优秀学生的硕博是可以压三的模式，然后可以很大缩短我们医学的培养周期，主要就是这几个方面比较吸引。

02:54

我总结了几个就是国中班的一个叫特色或者说优点，一个是还有奖学金，第二个是科研导师，就是可以直接接触科研导师在本科阶段。

03:07

第三个就是特色的教学安排，就是你们删除了一些课对吧？又加了一些别的课，还有一个见实习，这都是教学安排上有和别的不一样的。第4个是免研就是优先录取。第5个就是呃本博方向，第6个是有一个出国的机会。

03:24

你觉得这6点就是我总结这6点，哪一点最吸引你是本博是吗？对。有第二点的其次其次是接触科研导师。科研导师是吧？所以你在当时大一的时候，已经已经决心要走上科研这条道路是吗？可以这么说。可以这么说你是对。科研比较感兴趣是吗？从小就觉得科研比较那种。是比较感兴趣。从小很小的时候就了解到科研是做什么的。每个男孩子小时候不是说当科技家，这种意思。

04:10

你要不要选择就是医学这个专业是为什么会选择学医呢？或者说和，我们说一下你在高中时候填专业报名专业的时候的一个过程。当时报名的时候是只填医学吗？还是理理工？医都填了之类的，第一志愿是医学，然后其次是文学类的文学类的。

04:44

你高中学的是理科是吧？对liqueur。但我觉得我理科不是很突出。第一志愿就写了医学，对南京医科大学本来是想多临床医学的，但是成绩稍微差了一点。没有少，然后被调节到预防医学。你有跟父母沟通过吗？讲。志愿挺好，你父母支持你。你父母主要是让我自己来比较尊重我的意见，然后基本上没怎么看。主要是我自己的他们对以前这边没有产生什么影响是吗？主要是你自己去做决定，因为我也想填医学这个方向，我父母也比较支持医学学习，所以没有。

05:30

什么以前南医大。你是为什么会选择男的呢？是从什么方面了解的啊？因为我家是徐州的，徐州当地也有个徐州医科大学，但是我想读大学，不能在家门口转悠，我就想但是也不想走的太远。然后南京医科大学在全国也是非常出名的，然后也不是很远也不很近，然后就去了南大。

05:54

你是通过什么渠道了解到关于哪一点信息？当时我有一个室友，她爸就是一个医生，有一次对对对，有一次我们他来看他还跟，我们聊天的时候，他说以后报什么，我就说想读医学，还有说南京医科大学医学挺强的，你们可以考虑。

06:18

听到哪一个名字是吗？对。然后后面你有自己去了解过，后面就在网上百度搜了一下，有没有？一个老师就高中老师他们好，之前有没有同学也就是互相聊天那种。去医学院没有跟朋友们聊过普通聊天，也没有太仔细的去深入，没有深入的，你刚刚说你其实来的时候是想学医，然后报临床医学，是为调剂到预防医学，你在大一的时候其实有一个专业的机会，你当时报了吗？

06:56

没有，当时出来股东的创新班，然后我觉得这个可能更适合我一点。事合适吗？我觉得适合。你觉得适合是哪方面适合是和你的兴趣相适合还是什么？还是和你的规划比较相适合，发挥和那种理想更适合一点，因为高高中的时候并不是对医学感觉到很深，我觉得当临床医生治病救人就很很有意义，但是我读了预防医学对那些老师宣传，我发现如果当临床医生的话，只能一个的治病救人，如果你毕生精力可能并不能救得了多少，而且现在医学真正能治的病并没有多少。

07:46

然后预防医学的话，它可以从群体上来，去去从群体的方面去干预。一级预防二级预防是比那种临床的治疗意义会大得多，对于人民的健康也会有很大的促进，但是预防医学现在有一种偏离，从人群然后又到他们那种基础研究，感觉并没有很好的结合。比如说现在的工业学院，虽然说是从人群的方向，但是大多数的导师是做的基础研究，他就没有很好的融合，我就想如果我本科读的预防，然后在很早接触科研以后，是不是能把人群的层面，还有这种基础的研究层面能够很好的结合一下。

08:40

我可不可以说你的意思，现在其实他们做的这些基础研究，对于人群的预防其实没有太多的促进作用。

08:49

对比如说就像抽烟，人家都知道抽烟是怎么会促进肺癌，但是他们的研究不是去研究怎么去阻止人们吸烟，而是吸烟是通过哪种分子来促进这种预防的，就是说对于这种控烟并没有什么值得的帮助，我比较好奇你这个想法是某一次的讲座当年产生的这种想法吗？还是说是大一整个过程逐渐逐渐形成的？是突然一夜之间形成吗？还是逐渐形成的？就逐渐形成啊那么，在这个形成过程当中对你产生影响的主要是哪些？

09:29

比如说你刚刚说的讲座，还有讲课，还有上课的那些我们工业学院的老师都有那种老师、们嗯、老师的宣传，通过老师的影响，然后他自己参与的一些讲座的影响，也就是说你经历的一个过程变化就是从临床被调剂到了预防。然后当时其实有点失落的时候，因为觉得临床医生很有意义，但进入预防之后，觉得预防专业它是针对群体的，甚至比临床医生的一个的去拯救生命更有意义。

10:05

如果做得好的话，然后后来又发现预防的就是整个大方向有一点点偏了，偏离了你的预期，什么跟你想的又不太一样，有一点这种感觉，那会有很大的落差，并没有很大的落差。

10:23

你后来选择中班是因为想要从改变这个基础研究的现状，然后它的应用面更广一点一己之力肯定没法改变，只能说我以后从我自己的研究方向，如果可以的话跟这种工作和基础怎么样合作对。

10:46

去火车班是你是觉得是对这方面是有帮助的是吗？当时报名果重的时候，你有跟你的你说是从微信公众号上了解的，你有跟你周围的人去有聊天或者是，交流，包括老师或者说辅导员或者说同学，或者你高中同学，还有你的朋友，这些渠道你有跟他们交流过吗？有没有让你比较印象深刻的事情？

11:20

主要是跟室友是这个这个，室友交谈过，他们都有条符合这个条件，吗嗯他们都符合他们也都想在家，大多数都报名了，但是考试的时候没有考上来，但是他们感觉他们不是很重视，因为有一部分他是想转专业，有一部分只是想试一下，还有几个就是自己已经参与到了很好的科研项目，我就不想再搞别的科研项目。

11:55

已经参与到很好的科研项目是指什么？大创嘛、大学生挑战杯、挑战杯，他们大一就可以参加到很好的挑战杯的科研项目好是指什么？是指很符合他们兴趣吗？还是？力度很大？支持力度很大，一方面支持力度很大，第二个也是很体系的从一进去，然后开始培训做实验做课题，然后到发文章。挑战杯是吗？你当时选择公众的时候自己有参与到什么项目吗？就是去国中班之前。

12:34

对，你看你室友都参加，你也觉得很好是吧？你当时有想去做这个挑战杯吗？当时陈占斌他是选几个人，并不是真的所有人，然后我当时参加了我们老师的一个大学生大创，但是还没有开始，当时还没有正式开始，然后就出了股东班这个老师说老师建议我说你去帮这个活动，妈妈如果考上你就进去就回来，我这边当时跟我们的本科生导师我跟他说过，他建议你说考上国人班以后，然后什么，然后好好在国中班那边学习就可以不用参加大创了，因为他觉得这边可能不如国中班培养的更好。

13:21

就是你的本科生导师觉得国中班是要给大创给你带来更多的可以科研经历的。如果你当时手上有像你摄影一样比较好的项目在做的话，你还会选择不动它。我可能会犹豫一段时间，到底会不会选？现在不敢说，因为毕竟现在是选过了。你觉得就是你，本科生导师对你产生了去五中班的想法就影响大吗？

13:59

一定程度上的是一种你和他交谈是你单独跟他交谈，还是在你们交流会上交谈？我在学习室里面，然后日常聊天，学习是什么东西，他们地方有自己的学习室，然后他们的本科生导师会去你们学习室吗。就跟我日常聊天的，所以这种聊天是经常会发生的。本科生导师他是也是个新的老师，讲师然后可以就跟我们关系比较多一点，不是特别忙。

14:43

你产生去国中班这个想法是在这个老师跟你提了之后，然后你再去了解的，还是说你自己本来也在了解？正好老师也说了一下，是出来招生，宣传之后然后就跟有一次老师就来了，然后聊天他就说建议我去报，然后我自己也比较想去报，它就相当于一个加速的作用。

15:14

你选择国中班，你觉得你最主要考虑的是什么意思？还是培养方案，就是硕博这种比较大的，最吸引最大的嗯。

15:30

这个是跟你的就是一个职业规划有关吗，也算。然后我觉得医学的周期很长，对于这种家境很普通的人来说不是很友好，然后他能这一下子缩短了两年也也，是非常的你学学医学医，还有包括选国内班爸爸妈妈都很支持。他们有没有跟你讲过跟你讨论过跟？没有关系。没有很深入的讨论过，他们也是看招生的宣传觉得很不错。

16:13

主要是你跟他们聊什么，他们的信息或许主要是通过你，因为他们文化水平不是很高，并不了解很多，然后主要是看你的意思，他们都支持你。你读国中班以来有没有发生过什么让你印象比较深刻的事情？我印象比较深刻就是大。二是进博士班第一年结束的时候，大二的暑假，然后他们开了启动了一个项目，科研训练计划，让我们工程班内部的三五个人组成一个队，然后有导师独立分配一个小的课题，让我们从全程参与，从课题一开始到最后这种逐步独立的完成。

17:06

你一个人负责一个项目吗？还是一群人负责一个项目，小组三五个人三个人。你在这个小组当中你觉得是扮演一个什么样的角色？

17:22

当时负责小组是两个人，当时负责人是另一个，但是我们分工大大55开的那种。后来他觉得这边发文章太慢了，他就跑了他就走了。知道这叫什么？发文章太慢了，基础研究发文章周期很长，不如工位那边很快。他的退出活动不是他就退出我们这个组去别的组，然后现在主要是我一个人。其实就是你一个人在做这个项目，我很辛苦，一个人做这个项目不是很辛苦，这个工作量也不是特别大，小课题。

18:03

然后还有一个师兄们帮忙有师兄们在帮你。对你没有动摇吗？没有跟走掉的同学一样吗？一样的那个想法吗？做技术研究的确要沉下心来做。很久产出成果可能也没有像他们工位或者临床一样，效率那么高，他们的发文章速度应该想要发的话还是蛮快的，基础研究可能比较难一点。我觉得这算是一个蛮满劝退别人的一个点。

18:36

你怎么想的？首先我们开展这个课题的目标并不是让我们去发文章，而是在这个过程中锻炼我们这种科研能力思维之类的东西，对文章没有什么要求。主任就说了，说你们发不发无所谓，主要是这个过程。然后这就给我们很少心理压力，其次国中班定位并不是说弄出这30个人来，然后以后就使劲发文章，并不是说发这种小文章，而是说能够做出一些突破性的进展。

19:10

你现在我对这种发展时间就跟他们说难听点，水文章我并不是很想跟他们一起这样，他说这个课题估计还有个一两年就可以出一些成果。

19:27

你说你比较在意对能力的提升，你觉得经过这段时间的学习，就是从进入国中就不要进入国中班，你们来就从大一到现在，你觉得你你自我评估的话，从你自己的角度出发，你觉得你的能力提升了吗？

19:45

科研这方面自学的能力确实提升，自学的能力，获取信息的渠道那种。你觉得你获取信息渠道的能力是从是通过上课，或者说通过这些训练习得的嘛嗯就是通过做接课题以来，然后会在师兄指导下说去哪个网站去找一些什么样的信息，就怎么样检索文件或者你想要的东西，还有自己在网上搜的一些课程来看，你有提到你师兄们好像对你帮助挺大的。

20:28

你进入股东单之后，他们有没有跟你聊过股东班跟股东班有关系？比如说股东们怎么样，然后前发展前后怎么样怎么讲？有讨论过没有？具体的讨论。现在1+3不太现实，可能有可能实现不了。升值。升值这个东西如果想去医院找工作的话，就业面并不是特别广，但是如果好好干，在生命科学领域分分头办事还是可以的。类似这种正常的现象，是不是有没有对你这些言论有没有对你产生什么影响？

21:14

比如他们说我们家说应该是1+3有没有？我现在也在怀疑什么实现保障是吗？我现在也在怀疑他到时候到底能不能保证培养模式就是5+1+3是为什么有可能？其实我觉得你们现在做的很多事情是啊是，本科里面可能并不会侧重的事情，你们现在做的可能很多是有点像研一研二研三在做的事情嗯。

21:48

你为什么会觉得5加他们可能做不到5+1+3的？那现在的硕博连读的话至少也要6年了。如果我们这儿一下子就为什么至少要6年是你在哪看到一个规定吗？是啊是那边今年新出的，前两年好像连读的话还是2年硕士3年博士5年，现在已经变成了6年。是国中班的规定还是工位的规定？整个学校的大趋势就是这样。全国好像也有。

22:26

你是不是也有提到就跟就业有关的东西，你有没有想到？将来就业没有。要往什么方向走，大概是你去哪儿？

22:41

好的话是想留在本校当个研究员之类的pi之类的，如果不行的话，去一些医院的生殖中心做科研岗，或者是别的学校的教学岗和技师之类的，陪他操作时，有这方面就这些方向有符合你的预期吗？跟你本来进入我们班真有想象的职业规划有达到你的预期吗？大致是一样的。

23:17

进工程班主要想以后从事科学研究，如果在学校的话或者去医院科研岗还是从事的研究工作大致是一样的，进入国中班学习之后，有没有什么让发生过什么让你印象特别深刻的事情？蛮方便的。什么东西没想到。

23:41

印象特别深刻的，刚刚说了一个小课题的事情，其他没有也可以不说，或者让你不高兴的事情也行，高兴或者特别开心的事情不高兴的让你失望的。失望倒是有一些，比如说他们培养的这种并不体系化，并不体系化，我可以说具体一点吗？一开始我们进来了，然后让这30个人在两边选导师，然后分散下去。

24:21

你是可以用瓷砖的时候，感觉很散，然后会浪费很多时间，还有他那个，课程的设置，这时候给我们砍掉了很多课程，然后又补充了一些特色课程，但是这些特色课程他们摆摆的位置并不是很很重视的位置大多数是以选修课的形式，他给我们上了然后，所以就给你们砍掉了很多必修课，然后又给你们加了很多选修课。

24:47

 Java他们说是国重特色课程，但是他并没有体现出多么特色，然后也没有生殖生物学、发育生物学这些，这两个人倒是挺好的，然后其余的前线论坛那些都是选修课。

25:04

你觉得像前期论坛这节课其实没有什么特色，给给给你帮助不是很大。类似于科普那种，如果好好上的话也是很有帮助的，但是以选修课的形式，然后学生就可能会不重视。上课的时候不去，或者说吃的不听的多，你觉得为什么你当时我问你当时去没去，你觉得为什么你那个同学你周围那些同学他们会不去，就是因为是选修课，所以他的学分就没有那么重要是吗？

25:41

对，选修课又不考勤也没有期末考试就算。都没有期末考试。会不会是因为其实同学们觉得上门课也学不到什么，因为而且反正他也不重要，我为啥要来啊？也有这方面原因。那些导师讲的很前沿，然后我们当时上的时候是大二或者大三，所以说在科研实验室轮转过一段时间，但是对这种知识掌握还是有一个很浅薄的层面去了，不一定能听得懂。

26:16

大多数都听不懂这种情况是会大会经常发生吗？还是会偶尔发生？会经常发生一种经常发生。

26:24

可能他们选的内容对你们来说太难了，或者说太前沿了嗯，对于还你们在西德一个基础，科研手的手段方法，还有技术理论知识的时候，给你们直接讲一些前沿的东西，可能你们都接受接受不了太难了，你刚刚说你们在科研轮转的时候，教师这个很散是什么意思？

26:56

是你们当时应该是可以选4个老师去轮转，对吧？大二的时候很散，是因为你觉得老师和你们的方向不符吗？还是？是什么意思？我没有听太懂，就是这一个班级的整体被分散到了好多的实验室嗯，就有的，实验室会教的比较的体系，有的实验室就是在他旁边看着，然后这样就会造成这个这些，人群同学之间有一些差异，是吧？

27:29

有的就在那边学习觉得很好学的操作什么，但是有些是并不需要他们去干什么，也没有他们能干的东西。在实验室可能直接拉出来给你们选，但是并不是说每个实验室真的都需要人，可能就是说让他可能看似给你很多选择，可能真正需要学生去的，或者说真正想去培养学生的就能不是所有的实验室都是这样子。可以这么说。

28:00

还有一点就是我们学习这些实验操作，主要就是在这些实验室里跟那些师兄是这样，但是他们水平也是有差异的，有的人掌握好一点，从原理从操作什么更好，也有的人只会简单的这种操作，然后这也会造成我们这种实验手法操作的一些差距。如果说能够从一开始能体系化的一起集中过来教学的，这样会好一点。从你个人的感受来说，就是你个人的科研冷战的经历来说，你觉得你是属于比较幸运的，那一拨人还是比较惨的一拨，人前期比较惨，后来就是有4个导师，前一半前一半是比较惨的，然后后来到了比较好的实验室，所以技术水平都很高，这会影响到你后面选导师吗？影响了星期大三的时候，你们是确定研究方向的，对。

29:08

基本确定可以确定一个大选的导师。

29:12

是你大轮转的其中一位吗？是的，我大二上半学期上学期是两个，然后下学期是两个，但是下学期我就只选了，然后后来一直跟老师他做的大概是什么方向，可以跟我们说一下吗？精子发生，精子发生，和你，当时预期要做的这种基础性研究符合吗？符合你当时的一个设想说你说前一半比较惨的，我想知道具体让你觉得比较惨的是哪些事情？

29:58

刚进来的时候感觉他们做实验，白了做实验很多。然后我去实验室他们主要是做那种统计分析，数据方面的，然后因为我当时是刚上完大一，然后这些知识还有那些编程的一一窍都不通，然后也没有时间可以教。那边看也看不懂他，他们干脆就不管说你去学习去看书，然后就到那边，并没有收获到什么，然后他们做那些实验都学会了，我们那边就没有什么机会去学。

30:35

那你坐我那，这也跟我自己的那种主动性有关，当时比较刚接触这种实验室比较的腼腆，害羞什么的，可能就那个时候他们在旁边做实验，他们自己自学，我们在他们自己的学习室里面就是工作室里面就那种做电脑上的工作，就是感觉数据什么？处理怎么分析比较他是大。二他刚上大二不懂。所以你觉得在前两个实验室都没有学到东西是吗？因为你自己专业上的一些欠缺，因为也没有教那些东西。

31:32

有没有渠道去学那些有渠道，但是当时不知道也没有人。当时不知道有渠道可以学相关的知识什么渠道，现在知道。知道就是那些网上有好多的课程，在网上自己找课程学还是得咨询，对。

31:54

你刚刚说有一个事情，硕博连读，可能他们可能实现不了5+1+3的承诺嗯，但是你在一开始的时候你也说了，其实比较新颖的一点是它可以缩短你的学制，我说假如假如说他真的可能实现不了这个程度，你可能会想过要退出这个有没有可能会说这是一个劝退你的点？想要。

32:23

都在读生殖这个方面的他当时是这样说的，说先上一年的硕士，然后会对你进行硕士进行考核，如果成绩比较好的话，表现比较好的话，可以直接到三年的博士，如果进不了的话，你可以正常读，三年的硕士硕士之后你继续读博，还是毕业就去找工作，这个就靠自己。我想如果没法1+3的话，读完硕士是可以。

32:58

你也是可以接受。

32:59

的嗯，并不是说一定要退出，你觉得你读了国中班以来，从你个人角度出发，你觉得你最大的收获是什么你最大的收获还是那种学习方法，学习方法、学习方法这个东西是你觉得是从哪里习得是实验室的师兄师姐教教会你，还是你跟室友讨论出来的？

33:41

这个也不叫室友了，叫同学国中班的同学讨论出来的，还是跟老师学到自己？主要是自己在这边摸索，然后师兄指点一下。所以实际上假如说你不参加过这种班的话，其实你假如还在预防医学的话，其实自己也可以写的这些不一定不一定对，因为进来有需求，需求就是迫使我去找就是去学这个东西，学那种东西。

34:10

如果我不进来的话，可能正常上的课，然后课后也不会有这种想法去找需求是什么需求。比如说他给你一个基因，你想获得基因的一些信息什么的，你到哪里面去找？首先到一些数据库还是下来，然后再到发收一些以前发表的时候这些文件，然后还有你需要学会一些做实验的原理操作之类的东西。你要去图书馆查阅那些书，或者到网上去搜这种这种这些需求。

34:54

促使你去就迫使你去学习这些这些知识。

35:02

嗯刚刚你说的大二暑假的时候，有一个科科研小组，当时大二暑假的时候你们已经选了导师了，已经你在做那个课题。当时选导师吗当时？选导师就大二的时候他会跟你444个垄断机会选方向讲错了。

35:30

你们大大三的时候选研究方向，但是水平方向并没有说具体的，你就必须在这个时候写出来，交上去以后只能干什么，并没有这种只不过是大家自己在自己的心里大二暑假这个课题是国中班的每一个学生都要参加的是吗？还是说就是像那种团委的科研训练这种东西一样，是可参加可不参加的。

35:58

其实是可参加可不参加的，但是基本上所有人都参加。

36:07

这个题目是导师给你的嗯，然后你就做了你自己对这个项目感兴趣吗？或者说换一个说法，你你在自己进了补充班以后，因为科研的需要迫使你去查了很多资料，你在看了这么多书以后，你有没有感觉到有让你激发你的兴趣的一个点，或者说一个方向，或者说一个话题，或者说一个其他类似的一个什么现象，你比较感兴趣？

36:48

有因为人对那些未知的东西都是很想去弄，搞明白它到底是什么原因呢？然后正好科研也是它不像我们正常上课了是吧？手上都有所有的对不对？这边科研就是需要你去自己去探索，去做实验去验证。然后你有觉得你最感兴趣的东西吗？你就是经过这些应该说17级是大四了对吧？经过这三年的实践，你有感觉到哪些事情会让你感觉引激发你的兴趣吗？或者能吸引你的注意力，甚至注意力，或者说你非常感兴趣的人类的生殖力。

37:36

生殖医学，对，因为这是生殖医学主要这点事情，就对于这种生殖力的这种保护或者促进比较感兴趣，然后再往上看到一些发表，介绍一些科学研究成果，我会重点去关注一下，你点开点进去看一看。

37:56

升值率是什么样的，这么说过去几十年男性的精子质量下降了50%，如果再不逆转的话，可能再过多少年之后就不会有自然生殖这种过程可能就会要借助辅助生殖。

38:15

但是如果现在有研究发现了一些什么东西可以阻止下降的过程，不是对这种方面比较感兴趣，你有试着往这个方向去做更多的研究嗯，现在基本上在这个方面上研究的新男性新鲜中发现一些东西，但是实在是太复杂了，靠我这一个人是没有办法，你只能在自己的老师给了一个小课题中把自己给做好，这个小课题也算就是你讲的甚至立的一个子课题，可以这么说吗。

39:07

主要是官网中的一些东西都是属于这个方面。你把你想要研究这个方向放到你未来规划当中去，就以后如果有条件会去做这方面的研究，会如果有机会继续做科学研究的话，我还会做真的发生这个方面的课题。

39:41

你可以说说就是这几年来让你最自豪或者最有成就感的一件事情，没有的话可以不说。

39:50

前段时间我们自己从一开始，然后到最后发表一篇中文的小综述。中文的小图书虽然跟别的那些发SCI的没法比，但是这也是我独立完成的一个在师兄老师的帮助下，学习相当于有了一个成果出来了。嗯会让你感觉到比较有成就感，比较自豪，对。

40:19

然后还有当时在大三暑假的时候过了一年有个中期汇报，然后汇报成果的时候可能比较受老师的认可。比较说他的认可是指什么？追加经费。你的课题被追加经费了，所以就得到老师的认可，会让你更有动力去做这些东西。

41:02

我们现在下面想问你一个问题是你在进入博专班以后，会不会有学弟学妹来问你一些什么关于活动班的问题？他们问你大概最主要的是哪一类问题，或者哪哪一种问题，让你印象比较深刻的？

41:39

一般都是已经进来了，他会问我哪个实验室比较好，或者说我是选导师轮转的一种选法啊，还有做科研，还有较多的还是大二结束了一个承担课题的一个项目。嗯这两方面问题比较多。

41:59

大二结束承担课题是你刚刚讲的暑假可选可不选的东西。这个课题来源于是你的导师就是轮转4个导师给你的课题，还是说导师会放一个课题一个列表出来，然后你们学生自己选？这形势是怎么样的？比如说三个人组成一个小组，然后这三个人其中一个导师他手上有一个比较适合我们做的小课题，就给这三个人，给这三个人的标准是什么？

42:32

标准并不是很多，我们这几个人就向他们申请项目一样有个申请表，我把这方面的背景啊规划都写清楚，到时候会做一个类似于开题报告的一个东西上面会有导师会有学生在下面听，然后基本上就会不通过。

42:55

会有竞争吗？会有两个组，同时今天一个项目情况发生，没有没有。

43:00

没有，基本都你们私下里就分配好了，你别来报了，是这样的吗？这是我们分一个小组就我们三我们三个做。对万一另外三有一个小组也对我们报改进一下。那他们三个人不是我导师下面的，他们就没法过了。

43:19

还是导师。导师名下的对学生组成的一个小组。不是自由组的小组，对。肯定有一个是自己导师带领，另外几个人可以不是组个队，所以没有那种大一，不确定要不要来补充班的那种人来问你问题。要不要来我们班？对那种大一在活动办宣传期间，有没有大一的时间来办？

43:53

这个倒没人问我没有是吧？他可能会问别人，如果有人来问你跟他推荐我要看他对于这种科研的兴趣，他如果说只是想把当一个跳板，所以或者说想拿甲状腺或者说看保研比较的，那么我会会很建议他还是把这个机会留给，所以真正喜欢科研，想对人类甚至做一些共同的嗯，但是他们大一的话也不会有这么多的想法，还是要看它的主要目的是什么，所以你觉得主要还是最适合那些可能只是想读个研可能都不太适合可以这么。

44:55

说吗。还是比较适合那些想要一路走走科研道路，要一路读到博士那些适合这种要走科研道路的，就想读研的话，不是很推荐，因为他最后股东班培养这些人，主要是想让我们大多数都选南医大生殖这方面的研究生，但是有好多人会眼界高一点，想到一些更好的学校去读。

45:26

然后所以拿五中班当一个跳板。对。跳板让他跳还不清楚，因为第一届还没有到那个时候。我想问一下你是那种水域而安的人，比如说我举一个例子或者什么假设一种情况，导师可能突然换了个方向，可能你现在做的和你现在真的做的方向不一样了，想希望你跟他跟着他一起做另外一个方向，你这种情况你可以接受吗？

46:01

如果不造成什么很严重后果的话，我会如果说我到后果是指什么？严重后果是指什么是。比如说我到快要毕业的时候，我这个课题再做一点就可以出成果发文章顺利毕业了，但是他突然让换一个没法正常毕业，我可能会比较大影响到你正常毕业。

46:23

对你不影响你想毕业的话，你还是会接受的什么，假如说反正有情况，那也就是说在这些选择当中最能触动到你核心想法核心影响因素，还是你的毕业，还是就对未来规划的一些如果把整个规划都打乱了，那就是时间的问题，甚至就是一年两年的这个，你以后读了博士的话，也是想读升职的是吗。

47:08

刚刚讲了就是你们课程生殖生物学、发育生物学，你觉得他们比较有意义吗？我刚刚好像听你说的是吧，比较你觉得他给你带来的收获是什么？当然收获就是发育生物学还没上，现在只上了生殖生物学，生殖生物学可以从好多个方面就是什么性？磁性。这两方面可以从一个整体的观上面，让我们了解生殖医学整个的方向。

47:45

你有没有了解过你们预防医学删掉的那些课程？你有跟你室友聊过吗？你室友应该还是预防医学对吧？前三年是大一大二大三的时候是有他们还是正常预防医学。但是我们删掉的一些课也是一些没了解可以不说，了解过一些社会心理学什么经济学这种，对于科研这方面的并没有什么实质性的，可能对于生殖的研究来说可能就没有什么太大的帮助，但是假如你还是从事预防医学的话，可能还是要了解一下社会医学上面的东西，对吧？

48:33

所以他们自己也认为删掉那些可是水坑，是吧？水课是指老师的卷子不难，的啊是说老师上课没有什么对他们帮助不是很大，感觉说不考勤那种，可能有点像社会科学的一些东西。删掉了。其实不水的话也就是大四的时候会他会知道我们上妇儿的4门临床课程，还有传染病，然后他们正常预防的那种口腔皮肤病，耳鼻喉神经，这些预防医学课他们都上，这方面这上面的比较多，可能会感觉到比较可惜。

49:19

毕竟是医学的学位。

49:21

之前的话会不会有点我比较好奇你刚刚说像那些社会科学课，你的同学认为是删掉的，水客就是不足为奇，他们认为水是因为这个课给他们带来的，他们就从你室友你跟他聊天，你的感觉出发啊，他们觉得这东西给他们带来的收获不是很大，还是说我随便准备就能过考试了，没有？

49:50

内外妇儿这么难。主要是哪方面的？主要是这种收获不是很大，因为他收获不大。他现在还是在学校里面上课，如果说你学一些，你找医学方面的专业的知识的话，它会跟别的医学科就是融会贯通一下，但是这种社会科学的话他在这边用不到，可能等步入社会了之后想用的话，基本上都忘了。

50:21

其实我觉得你个人来说还是蛮有那种家国情怀的一个学生的，所以你对于科学研究来说还是比较希望你的研究可以有更大的一个应用面和范围面的，其实这个还是蛮偏向于社会科学的，其实。

50:42

所以我比较好奇你这个思想思想来源，你自己有有有思考过方面你有没有觉得你跟你的室友不太一样？他们可能可能会觉得比较想想过一场考试者或者是怎么样的，但是你可能会更清晰。整个整个整类或者之类的。

51:11

你有感觉到你有你有和他们不一样吗？这个想法也没有什么太多的鬼，我。没有是吗？就讲举一个简单的例子来说，当时科技小组的时候，他们觉得发文章他不出来就走了，你还在那个课题小组里面你有过思考过为什么他们会有这种想法吗？或者为什么你还能坚持下来是什么？支持支撑的坚持下来做这个事情。

51:38

不去想发文章还是觉得我们之所以进这个班，并不是想做一些快乐，那些快的一些没有什么用的研究，还是想要在生殖领域做一些突破，突破性的研究，如果说就把精力都放在发一些这些小文章上面，其实技术研究的话，你要想做突破其实很难的，你你有你有一意识到问题是非常难，大家都知道，但是如果不去试的话，那就恐怕只是一个词，但是现在整个环境工作的环境，我不知道你有没有去了解过，像你们导师他们科研环境，包括像国中班他们宣传的PPT里面也会经常说导师发了多少文章，或者影响因子有多少，你有没有有没有有没有想过，我在假设有没有想过，假如以后真的是从事这种最基础的科学研究，可能它的确会影响到更多的人，但是对于你个人来说，可能你的就是这些影响因子或者这些文章可能就没有别人强，可能甚至会影响到你后面的整个科研道路的发展。

52:51

有没有想过他走这条路可能给你带来的负面作用？

52:57

因为现在中国的科研就像像那种高山论道，从一开始就像爬山的过程，想赶紧爬到山顶，然后到山顶之后才能去欣赏那些东西，就做自己真正想要的，但是过程很困难，你在快速爬山的时候会忽略了一些旁边的东西，就就说的直观一点，就是我出来毕业毕业之后去工作，然后我想做一些比较大的，但是周期很长，需要可能8年10年，这个时候没有文章，可能我做一半就被辞退了就这种所以说从一开始先要写文章，把自己抬到一定的高度之后，然后你才能去做一些真正想要的。

53:54

所以你是想想想想想这样的就很纠结。

54:00

很纠结，对，因为我觉得这样的路的话，其实应该是和你的价值观是冲突的。

54:06

是冲突的，因为人精力最旺盛，然后创造力最丰富的时候还是年轻的时候，然后但是年轻的时候，你就追求了些文章，追求什么？追求晋升职位，然后你就会错过了这些机会，等到老了真的想做一些东西的时候，你会发现做不动，没有年轻人那些丰富的想法，你有思考过怎怎么办吗？或者自我消化，或者说自我调节，或者说干脆改变一下自己的想法，有想过吗？有想过，但是我觉得可能太远了，是我自己力量太弱了。

54:46

远远不远也快了，快毕业了还有几年。我觉得可能现在中国科研领域也也会意识到这种问题，到时候看看他们会不会有所改变。我们说一种比较坏的情况，假如没有改变的话，这种现实情况和你价值观的冲突，你你你是打算怎么去综合去调解。

55:10

我也就只能像他们一样，先睡一睡，然后比较好的话，做了一个到一个比较高的位置，然后自己有了一定的影响力之后，后来就提出来从自己的这方面开始改变。因为如果你只想按照自己的路子走，就是把年轻的时候精力旺盛的时候放在做一些非常难的拿出成果的课题上面有可能做一半自己就什么也没有得到，科研大环境真的不是很友好。

55:59

你说你以前小时候想做科学家，当时是因为周围人都在讲，受到周围人影响才会想去做科学家的。也没有当时是在农村周围人的文化可能都不高，但科学家也都是从电视上看到的。

56:25

比较有面子，对，当时社会地位比较高，对当时对于这种方面也没有太没有什么了解，当时很小谁会想这些东西，你有没有我现在想再问另外一个问题了，你觉得国中班和你你和室友聊天感觉到的预防医学最大的不同是什么？不同还是预防医学，它不是你说你觉得没什么不同，也可以说。你觉得没什么。不同有很大的不同。预防医学还是从广度上面是这种人群这种去怎么去干预它？属于一级预防一级，但是国中这边主要是一种深度，从一个很小的东西到分子到蛋白质到 Rna到DNA这种完全是两种不同的方向。

57:30

你有没有想过，假如说你当时没有去过这种话去继续学预防医学，其实你可以探索到更多和人类相关的一些技术的科学研究领域，你进入旅游国中以后，其实你的范围有一点点是被限死到，甚至是这一小块当中的，你在大一的时候可能还没有了解到预防一些很多东西的时候，你的大二直接来到了甚至不中，你的研究领域就已经被限制在这一块，你没有了解到更多可能会激发你兴趣的东西，你有没有想过这个问题？

58:02

你们会觉得可惜我之类的。现在你有没有想过这个问题？

58:06

有，我还跟当时导师说过，我说我们现在就选当时以后会不会就现实在这一个小小的所以这种想法有点多了，因为现在方法是相通的，只不过你的东西是方向是不同，方法是相通的。他说就我们也不希望培养出来，只知道生殖的领域一些东西，但别的东西都不知道，那也不是我们想培养的学生。我们主要是想这是你导师说的什么？他说我们主要是想培养你大三的导师，对，你是现在的导师。他的意思主要还是生殖领域这个领域助手，然后培养一些做科研这方面的方法思想，还有你的能力，到时候你如果这一套你都掌握的话，所以你不想做升职，你想做心血管或者做别的，然后你要做的也就是花一小段一部分时间来了解这个领域的一些知识，然后但是他的方法思维体系还是一样的，所以你也能很快的入手行业，所以你也认可他的说法。

59:29

认可。对。现在发现确实是这样的。我是像之前在实习就是不同的科室，他也会让我去了解一下，这是这科室方面的研究，然后你说是 vivo的事情吗？对。然后发现确实也是这一套读一读他们的他们领域的文件，发现也并没有什么特别大的差异。你们去实习，比如去看看一下，就看看为什么会要求你们去读他们那个，科室的文献。他们知道我们的主要是做充值的，比如说像升值中心的时候，他们会让我们写综述就这种可能他们知道我们这种主要的方向是以后做科研的，或者说自己也想去了解一下他们的一个老，带教老师在做什么研究，你自己想想看看他们在研究啥？

01:00:33

嗯你觉得果种参加果种班有让你失去什么东西吗？没有。的话可以不说，也失去了一些，不能说是失去就很难讲，或者说可能是你曾经想要的就是来了，果肉麦以后可能就没了。不太好说就可以。不说是不是也有一种后我是后来设想的，如果说我的想法一直都在，但是我真的不是国中，而是普通留在异常医学专业会不会更好一点，就是什么医学、临床医学、预防医学、预防医学就留在更好，预防医学会好一点。

01:01:25

大学培养的就是那，如果是知识很专业的话，有可能是一种局限。大学生应该培养自己一些批判性思维，还有人文科学的一些知识，还有一些什么其他的这一类的。你觉得你自己的就是你刚刚提到的批判性思维这些能力，你觉得你还没有很好的习得是吗？因为进来之后大多数的时间在做在在学习在做实验，或者说做做自己的小课题，然后对那些别的东西支持，比如说哲学类的文学类的那些可以拓展自己。

01:02:08

其他方面素养的东西时间就会错过，没有这些时间。后来发现是想是后来是某一件事情让你突然这么想的，还是是有什么那个点，还是说你一直都在思考问题嗯，一开始并没有思考，后来我也不知道为什么觉得觉得大学生活并不能只是抓投入在自己的领域，东西好像是看着是没什么事情你没做到吗？还是看了什么话？好像在哪看到一句谁说的什么话，好像是外国哪个大学的一些什么思想说如果大学生培养出来，他对于自己的专业技能非常的精通的话，大学教育有可能是失败的。

01:03:02

这种他们主要意思就是说大学并不是要专门去配合他这个领域的技能技能，要到研究生的时候再培养，大学生就要培养的就是他们对其他领域就像批判性思维，还有对哲学心理社会那种那种思想意识的培养，其实你也可以，比如说在课余时间去去看看这个社会科学的这些东西。

01:03:30

对，现在就开始。抽时间去看看，对。感觉是你现在除了上课，还有比如见识系，还有一些科研训练，其实平时还会干什么？所以说你的兴趣爱好和乐趣。晚上的时候会运动，中午如果中午会稍微睡一会，然后会看一些课外的书，最近才开始人文社科的因素吗？但是进度很慢，因为时间真的太有限。其他的没有问题。你在读国中班期间有没有让你觉得压力比较大的事情？有。除了果冻班，我对做进实验室做那些实验还是比较感兴趣的，我就想把大部分的时间都就在那个上面，但是当时我女朋友好像非常的不满意，就是你你一有课时间就跑到实验室都不都不用那种。

01:04:59

他会跟我平时吵闹一下，然后我就觉得我去实验室对学习有帮助的，你为什么要跟我当时可能会跟他的关系会比较紧张一点，压力会比较大怎么怎么解决了你？

01:05:17

解决后来各自退一步，各退一步。你稍微多出一点时间，他们也不要那么。他是国中班的吗？不是，他是别的专业。他是别的学校的别的专业。我想问一下，你觉得大学这几年从本科一直到未来的硕博，那你对你来说最重要的事情是什么呢？从你个人的角度出发，你从个人的角度可以是和女朋友好好的走下去，也可以是拿博士学位，也可以是发文章或者之类的。

01:06:12

你觉得整个阶段来说，高等教育的阶段来说，对你来说最重要的事情。这样是是学业上面这是拿博士学位吗？拿到？不一定是非得博士吧。你说学业上我想听的具体一点可能是学业上有什么突破吗？还是说达到最终的目标还是说嗯，学学学到什么方法或者什么之类的，最起码还是说你你你是不是没有仔细想过这个事情，没有特别深的想过，但是最起码要顺利拿到这个相应的学位了，读完硕士就是拿到硕士，读完博士就拿到博士，是先要解决以后的这种生存问题，比较现实一点。

01:07:05

对。好的话就可以做一些比较好的成果以后，更可以走得更顺利一点。在做基础研究的时候，也有很大的可能性会失败，这个会让你就是打退堂鼓嘛，或者说改学改改掉改成别的研究方向，就是以后工作就不是学习了，工作以后的话，比如说你已经可以开始自主的开展课题研究了，可以申报你自己想要研究的东西了，你申报了某一个方向，但是这个方向可能成功的假设成功的可能性比较急，毕竟是基础性研究。

01:08:01

你你你可能就是在这个过程当中发现，未来成功的可能性可能比较低，你还会继续开展项目如果说课题方向就是个错误的，肯定要是及时的停止掉。

01:08:22

如果说它比较难的话，或者说他进展的方向跟自己预期都不一样，还是要做下去，因为科研生命科学领域谁也说不好，只要不是错的，那就只要不是错的，大家可能成功的可能性比较低，但你还是会继续做下去。你在期末考试的时候会感到焦虑吗？会比较焦虑，是比较强的那种，还是说有一点点焦虑？中等程度的。因为平时大多数课余时间还是从大一到到现在都是一直会回家，大一到没有大一的时候那么焦虑了，还是比以前更焦虑。

01:09:11

就进了股东班之后一直到股东班以后会因为平时课余时间会很多一部分放到实验室，然后你平时的复习什么的就会落下，到最后期末的时候就会比较着急，那个时候焦虑，所以说你进了股东班以后，其实到了期末考试那段时间，你会比大一的时候更焦虑。

01:09:32

因为时间少了，但是也没有非常焦虑，平时感觉其实还是很好。

01:09:40

平时你的投入平时你在上课的时候还是会很认真的去高去预习复习这样的学的。对，你就平时上课的时候会专注的听一点，到最后有时候发现焦虑都是瞎焦虑的，考的还是不错的。最后也会实验室也会给两两三周时间去自己去复习。

01:10:05

如果有时候焦虑是自己给自己的，我想问一个假设的问题，假设你发现你回想一下，假如说当时大二进了国中以后，发现研究的方向其实并不会给人类带来或者说整个人类和整个社会带来很大的改变，和你想象的不太一样，不是那种技术性研究的话，对于当时的你来说你会选择退出吗？

01:10:37

你不会选择退出。不会选择退出，因为毕竟是医学的，如果对人类健康资金没有什么，他们也不会开展。嗯再说了，他们不开展以后我也可以自己做，还是要这是一个机会，并不是看他做什么一个方向。

01:10:57

机会就是学习机会。行行。你来那儿学习的话，主要还是想学习那些科研的时间的技能吗？还是想学习什么的？那就是想学习科研这一一套东西，科研的方法思维好，我没有什么问题。没问题，我还想问一下。因为你们有出国证照吗？你有出国深造吗？没有，现在还没开始出国深造。这个是每个人都要去的，还是说选择性的自己报名的？

01:11:55

现在还没开始没有听到这方面的消息吗？没有听到，你看到你觉得有的同学怎么已经出口了？他们是各自学院每年寒假还有一个访学计划，所以这个并不是国中班的，国中班的出国是闹还没有开始。你们是大部分时候去的时候，他们说有跟你说的是什么时候吗？只有开始。刚开始宣讲的时候说了一下，后来就没怎么提。过。对。估计现在这个形势很困难。谢谢。谢谢。
