# Supplementary material for: Why do you choose this program?—A decision-making model of medical students based on grounded theory
Source: PLoS One. 2023 Sep 15;18(9):e0291634. doi: 10.1371/journal.pone.0291634 (PMC10503722; doi:10.1371/journal.pone.0291634)
Supplement: S1 File — (ZIP) [file pone.0291634.s001.zip › RAW DATA/P5.docx]

00:00

It can be said that today is also a chat, probably in such a process, which is a purpose of our interview. Before the interview, I will first confirm the experimental ethics and ethics instructions with you. In this interview, the interviewees participated in the principle of equality and voluntary participation. The sellers must truly express their own ideas and cognition, and confirm that they meet the social security conditions. The interview process will be recorded, but the recording materials will only be used in an anonymous form for scientific research, and will not be leaked to any third party.

00:37

During and after the interview, the respondents have the right to cancel the researchers' audio recording materials. Do you know and know it?agree. You are level 117 in preventive medicine, are you?two. Let me add that the first thing I want to know through what channels you learned before you entered the national middle class? Because at that time he through the wechat public account propaganda what, enrollment and then I saw. You mainly know through the wechat public account, the degree of understanding, you now think that you know at that time.

01:28

At that time, the degree of understanding only saw that he said on the public account of some enrollment conditions, and some training plans in the future, but at that time was just an outline of this kind of publicity understanding, not very, but I remember you have a lecture, have you ever participated in the publicity?

01:52

In the briefing, your main source of information is from the public, not from because the briefing on the main content of it is mostly with the public is overlapping, so you first know from the public, do you think the know about the middle class what is to attract you, attract you to how many class?

02:20

The first aspect is that its reproductive medicine is the platform of the state key laboratory, and then the starting point is relatively high. The second is that its training program allows undergraduates to engage with real contact research like graduate students. These are the two things that mainly attract me. Secondly, its quality plan, during the period of master's degree, his master's degree for excellent students, and then can greatly shorten our medical training cycle, mainly because of these aspects are more attractive.

02:54

I have summed up several of the characteristics or advantages of the Chinese middle class. One is the scholarship, and the second is the scientific research supervisor, who can directly contact the scientific research supervisor in the undergraduate stage.

03:07

The third one is the special teaching arrangement, which is that you have deleted some classes, right? Add some other classes, and a see internship, these are teaching arrangements are different from other. The fourth is exempt from research is the priority of admission. The fifth is benbo, and the sixth is a chance to go abroad.

03:24

Do you think these six points are when I summed up these six points? What point most attracts you is Benbo, do you? To. The second point is followed by contact with scientific research mentors. The research tutor, right? So when you were a freshman, you were already committed to scientific research, were you? So to speak. You can say that you are right. Research is more interested in it, is it? I think scientific research is younger. Is more interested in it. I have learned from an early age what scientific research does. Every boy doesn't mean being a tech, not as a kid.

04:10

Do you want to choose the same medicine major? Why do you choose to study medicine? Or, let's talk about a process when you fill your major in high school. Was it only medicine when I signed up? Or science and technology? When medicine is filled in, the first choice is medicine, and then the second choice is literature.

04:44

You are science in high school, are you? For liqueur. But I don't think I do science very much. The first choice was to write about medicine, for Nanjing Medical University was originally wanted to do more clinical medicine, but the results are a little worse. No less and then regulated to preventive medicine. Have you ever communicated with your parents?speak. Great for volunteering. Your parents support you. Your parents mainly asked me to respect my opinion and basically didn't see it. It's mainly myself and they didn't have much impact on here before, right? It's mainly you who make your own decision, because I also want to fill in the direction of medicine, and my parents also support medical study, so they don't.

05:30

What was it before the Southern Medical University. Why did you choose men? What do you know it? Because my family is from Xuzhou, there is also a local Xuzhou Medical University in Xuzhou, but I want to go to a university, so I can't walk around my door, so I just think, but I don't want to go too far. Then Nanjing Medical University is also very famous in the whole country, and then it is not very far away or not very near, and then it went to NTU.

05:54

What kind of information do you know about through? At that time, I had a roommate, her father was a doctor, once right, once he came to see him, he said what to report, I said he wanted to read medicine.

06:18

Do you hear which name, is it? To. Then you have to their own to understand, behind the Internet Baidu search, have you? A teacher is a high school teacher, who is to chat with each other. When you went to medical school, I did not talk to my friends, and I did not go deep, not deep, you just said that you actually wanted to study medicine, and then apply to clinical medicine, is to adjust to preventive medicine, you actually had a professional opportunity in your freshman year, did you report at that time?

06:56

No, I came out of the shareholder innovation class, and then I thought it might be more suitable for me. Is it appropriate? I think it's right. What aspect do you think is suitable or what is it suitable for your interests? Or and your planning is suitable, play and the ideal is more suitable, because high school is not feel deep to medicine, I think when clinicians cure save is very meaningful, but I read the preventive medicine for those teacher propaganda, I found that if when the clinician, only a cure save, if your life may not save how much, and now medicine can really cure the disease is not much.

07:46

Then in preventive medicine, it can come from the group, to intervene from the group side. Primary prevention Secondary prevention is much more significant than the clinical treatment, and will also have a great promotion to people's health, but preventive medicine is now a deviation from the population and then to their basic research, it does not feel a good combination. For example now the institute of industry, although from the direction of the crowd, but most of the teacher is doing basic research, he is not very good fusion, I want if I read undergraduate course prevention, and then after early contact with scientific research, can put the level of the crowd, and the basic research level can be a good combination.

08:40

Can I say what you mean, but the basic research they do now doesn't contribute much to the prevention of the population.

08:49

Like smoking, such as smoking, somebody else know how can promote lung cancer, but their research is not to study how to stop people smoking, but smoking through what kind of molecules to promote the prevention, that is to say for this kind of tobacco control and nothing worth help, I am curious about you this idea is a lecture when this idea? Or is it that the whole process of the freshman year is gradually formed? Was it suddenly formed overnight? Or is it a gradual formation? So, what are the main effects on you in this formation process?

09:29

For example, the lecture you just said, the lecture, and the teachers in our School of Technology have the publicity of teachers, teachers, through the influence of the teacher, and that is to say, a process that you experience is changed from clinical adjustment to prevention. Then I was a little lost, because I thought clinicians were meaningful, but after entering prevention, prevention specialty was aimed at the group, even more meaningful than clinicians to save lives.

10:05

If you do well, and then then you find that the whole direction is a little biased, deviate from your expectations, what you think is different, there is a bit of this feeling, there will be a big gap, and there is not a big gap.

10:23

You later chose the middle class because you want to change the status quo of this basic research, and then its application is a little wider. I can only say that I will cooperate with this work and foundation if I can.

10:46

Going to the train train is something you think it's helpful, right? When you signed up for Guoguo weight, you told you from the WeChat official account, and you had chatting or communicating with people around you, including teachers or counselors or classmates, or your high school classmates, and your friends. Have you ever communicated with them through these channels? Is there anything that has impressed you more deeply?

11:20

Mainly with my roommate is this roommate, this talk, they all have a conform to this condition, do they all meet they also want at home, most all signed up, but the exam, but they feel they are not very seriously, because some he wants to turn professional, some just want to try, there are several are already involved in the very good scientific research project, I don't want to do other scientific research projects.

11:55

What is a good research project? Big innovation, college students Challenge Cup, Challenge Cup, they can participate in a very good challenge Cup of scientific research project what is good refers to? Does it mean that it fits their interests very well?still? How strong is it? Support is very strong, on the one hand, the support is very strong, the second is also very systematic from one in, and then began to do experiments to do topics, and then to send the article. Challenge Cup is it? Did you participate in any projects when you chose the public? Is before going to the country middle shift.

12:34

Yeah, you see your roommates attending, and you think it's nice? Did you want to make this challenge cup? When Chen Zhanbin he is choose a few people, is not really everyone, then I was in our teacher of a college students, but has not started, has not officially started, and then out of the shareholders class the teacher said the teacher said I said you go to help the activity, mother if you go back, my side with our undergraduate tutor I told him, he suggested you said Chinese class, and then what, then study in the middle class there can not participate in the big gen, because he thought this may be better than the middle class training.

13:21

It is your undergraduate tutor that thinks the middle class is to bring more research experience to DaChuang. If you had a good project on hand as you did, you would choose not to move it. I may hesitate for a while. Will I choose it? Now dare not say, because after all, now is the choice. Do you think it's you, and the undergraduate tutor has a big impact on your idea of going to middle class five?

13:59

In part, do you talk to him, or do you talk to him alone, or at your exchange meeting? I am in the study room, and then have daily chat, learning what is it, and they have their own study room somewhere, and then will their undergraduate tutor go to your study room. I chat with me daily, so this kind of chat happens very often. The undergraduate tutor is also a new teacher, and the lecturer can be a little more connected with us, not very busy.

14:43

Did you have the idea of going to the national middle class after the teacher told you, and then you went to know it again, or did you know it yourself? Just the teacher also said, is to come out to recruit students, after the publicity and then with a teacher came, and then chat he said that I go to report, and then I also want to report, it is equivalent to an accelerated effect.

15:14

What do you think you mean when choosing a national middle class? Or training program, is the master and doctoral this relatively big, the most attractive um.

15:30

Is this related to your is a career plan, also calculate. Then I think the medical cycle is very long, for this kind of ordinary family person is not very friendly, and then he can suddenly shorten the two years, is very you study medicine, and including the selection of domestic class parents are very supportive. Have they ever told you about talking to you? It doesn't matter. Without a very in-depth discussion, they also see the recruitment of students propaganda feel very good.

16:13

Mainly about what you talk to them, their information is probably mainly through you, because they are not very well educated, they don't know a lot, and then they mainly look at what you mean, they all support you. Have you ever had anything happened since you read the Chinese middle class that impressed you? I was very impressed by the big one. Second, into the PhD class at the end of the first year, sophomore summer vacation, and then they started a project, scientific research training plan, let us internal engineering class members of a team, and then have a mentor independently assigned a small subject, let us from the beginning to the end of the gradually independent completion.

17:06

Are you in charge of a project alone? Or a group of people in charge of a project, with three or five people, three people. What role do you think you play in this group?

17:22

At that time, there were two people in charge, and the person in charge was the other, but our division of labor was 55. Then he thought it was too slow to post here, so he ran away and he left.Know what it's called? The article is too slow, the basic research article cycle is very long, not as fast as the station side. His withdrawal activity is not that he will quit our group to go to another group, and then now mainly is me alone. In fact, you are doing this project alone, and I work very hard, and it is not very hard to do this project alone, and the workload is not particularly large, but also a small topic.

18:03

Then there is a brother to help the brothers to help you. Didn't you waver in you? Isn't it like the classmate? The same idea? You really have to settle down to do technical research. For a long time, the output results may not be as efficient as their work station or clinical work, and their publishing speed should be quite fast, and basic research may be more difficult. I think this is a pretty good point to dissuade others.

18:36

What do you think? First of all, our goal of carrying out this topic is not to let us send articles, but to exercise us in the process of our scientific research ability, thinking and other things, there is no requirement for the article. The director said, said you hair hair indifferent, mainly is this process. Then this gives us very little psychological pressure, and then the national middle class positioning is not said to make the 30 people, and then later try to send the article, is not said to send this kind of small article, but said that can make some breakthrough progress.

19:10

Now you I tell them bad about this development time, water article I do not want to work with them, he said that there is be a year or two away to produce some results.

19:27

You said you are more concerned about the improvement of ability, you think after this period of study, you do not enter the middle class from the country, you come from the big to now, you think you you self-evaluation, from your own perspective, do you think your ability has improved?

19:45

The ability of self-study in scientific research does improve, the ability of self-study, the way of access to information. You think you are the ability to obtain information channels from through the class, or through the training acquisition is by doing the subject, and then under the guidance of which website to find what kind of information, how to retrieve files or what you want, and their search some courses on the Internet, you have mentioned your brothers seem to help you quite big.

20:28

After you entered the shareholder list, have they ever talked to you about the shareholder class and the shareholder class? For example, how about the shareholders, and then how about before and after the development? Has there been any discussion? Specific discussion. Now1 + 3 is not very realistic, and it may not be possible.revalue. If you want to go to a hospital to find a job, the employment area is not particularly wide, but if you do it well, it can be done separately in the field of life science. Is this normal phenomenon having any impact on you?

21:14

For example, do they say that our family says it should be 1 + 3? I am now wondering what to ensure, right? I'm wondering whether he can guarantee that the training model is 5 + 1 + 3? In fact, I think a lot of things you do now are ah, yes, the undergraduate course may not focus on the things, you may do a lot of things now is a bit like research, one research, two research and three are doing things.

21:48

Why do you think that with 5 plus they may not be able to do 5 + 1 plus 3? It will be at least six years now now. If we are here, why at least six years is where do you see a rule? Yes, there is a new one this year, the first two years seem to even read or 2 years master 3 years doctor 5 years, now it has become 6 years. Is it the regulation of the national middle class or the regulation of the work station? This is the general trend across the school. It seems to be there across the country.

22:26

Have you also mentioned something related to employment, have you ever thought about it? No future employment is available. What direction are you going? Where are you going?

22:41

Good words is to want to stay in this school when a researcher of pi and so on, if not, go to some hospital reproductive center to do scientific research post, or other school teaching post and technicians and so on, when accompany him to operate, have these direction is in line with your expectations? Do you really have your imaginary career plan in our class to meet your expectations? It's about the same thing.

23:17

Into the engineering class mainly want to engage in scientific research in the future, if in the school or go to the hospital scientific research post or engaged in research work is roughly the same, after entering the national middle class study, is there anything that has impressed you particularly impressive things? Very convenient. What did I not expect.

23:41

Very impressed, just said a small topic of things, other no also can not say, or let you unhappy things are also ok, happy or particularly happy things are not happy to let you down. There is some disappointment, for example, they cultivate this is not systematic, is not systematic, I can say a little more specific? At first we came in and then let the 30 people choose mentors on both sides and spread out.

24:21

You can use ceramic tile, feel very scattered, and then will waste a lot of time, and he, curriculum, this time we cut down a lot of courses, and then added some characteristic courses, but the position of these characteristic courses they are not very much attention to the position of most is in the form of elective courses, he gave us on then, so give you cut down a lot of required courses, and then give you added a lot of elective courses.

24:47

Java said they are national specialty courses, but he didn't show much character, and then no reproductive biology, developmental biology, these two people are pretty good, and then the rest of the front forums are elective courses.

25:04

You think like the early forum this class is not characteristic, give you is not very help. Similar to popular science, if good, it is also very helpful, but in the form of elective courses, and then students may not pay attention to it. When you don't go to class, or you don't listen to eat much, why do you think you asked you if you went, why do you think the classmates around you don't go, just because it is an elective course, so his credits are not so important, right?

25:41

Yes, the elective course is not checked or the final exam. There were no final exams. Is it because the students don't learn anything, because he doesn't matter anyway, why do I come? There is also this reason. Those mentors talked about the cutting edge, and then we were sophomore or junior, so we had a rotation in the research laboratory, but there was still have a very shallow level of mastering this knowledge, not necessarily to understand.

26:16

Most people don't understand this? Or does it happen occasionally? It happens often.

26:24

May they choose the content is too difficult for you, or too cutting-edge well, for you a foundation in west Germany, the means of scientific research method, and technical theoretical knowledge, give you some frontier things directly, may you all accept not too difficult, you just said you in the research rotation, the teacher this is very loose mean?

26:56

Are you supposed to have four teachers to rotate, right? My sophomore year was scattered because you think the teacher doesn't fit your direction?still? What does it mean? I didn't understand too much, but the whole class was scattered to a lot of laboratories, well, some, the laboratory will teach the comparison system, some laboratories are watching next to him, and then this will cause this, there are some differences between the crowd and students, right?

27:29

Some are there to learn to feel very studious what to operate, but some do not need them to do, and do not have they can do. In the lab may be directly pulled out for you to choose, but it is not said that every lab really needs people, it may mean that he may seem to give you a lot of choices, may really need students to go to, or really want to train students can not all laboratories are like this. So to speak.

28:00

Another point is that we learn these experimental operation, mainly in the laboratory with those brothers, but they level is also different, some people master better, from the principle from what better, also some people will only simple this operation, and then it will cause us some of the experimental operation technique gap. It would be better if we could systematically teach together from the very beginning. From your personal feelings, is your personal scientific research experience of the cold war, do you think you are lucky, the group or miserable wave, early miserable, then there are four mentors, the first half is miserable, and then later to the better laboratory, so the technical level is very high, it will affect you choose mentor? In your junior year, you set your research, right.

29:08

Basic certainty can determine a mentor for a general election.

29:12

Is it one of your big wheels? Yes, my sophomore year in the first half semester of the first semester is two, and then the next semester is two, but the next semester I only choose, and then later has been doing with the teacher he is about what direction, can tell us? Spermatogenesis, spermatogenesis, and the basic research that you were expected to do at the time, fit in? In line with one of your assumptions at that time, you said that the first half was miserable, but I want to know what exactly what made you feel miserable?

29:58

When I first came in, I felt like they did experiments, but I did a lot of experiments for vain. Then I went to the lab and they mainly did the statistical analysis, the data aspects, and then because I was just in a freshman year, and then I didn't know anything about it and the programming, and then I didn't have time to teach it. I can't understand him over there, they simply say that you go to learn to read, and then go there, and gain nothing, and then they do those experiments have learned, we have no chance to learn.

30:35

That you sit me that, it is also related to my own initiative, was just contact with the laboratory is shy, shy what, may at that time they do experiments beside, they self-study, we in their own study room studio is the kind of work on the computer, is feel data? How to deal with the analysis to compare him is big. Two he just got a sophomore year don't understand. So you don't think you learned anything in the first two labs, right? Because of your own professional deficiencies, because you do not teach those things.

31:32

There were no channels to learn from those channels, but I didn't know at that time. At that time, I did not know what channels to learn relevant knowledge, but now I know. Know is those who have a lot of courses online, on the Internet to find a course or have to consult, right.

31:54

You just said there is one thing, master, even read, they may they may not achieve 5 + 1 + 3 promise well, but you also said at the beginning of the time, actually more novel is that it can shorten your schooling, I said if said if he really may not achieve this degree, you might want to quit this may say that this is a point to dissuade you?want.

32:23

Are reading in this aspect of reproductive he was said, said a year of master, and then will master examination to you, if better, the performance is better, can directly to three years of doctor, if not, you can read normal, after three years of master's master you continue to read, or graduation to find a job, this is on their own. I think if you can't get 1 + 3, you can finish the master's degree.

32:58

You are also acceptable.

32:59

Well, is not to say that must quit, do you think you read the class, from your personal perspective, what do you think you are the biggest harvest is your biggest harvest or the kind of learning method, learning method, learning method this thing is where do you think is acquisition is laboratory brother sister teach you, or you discuss with your roommate?

33:41

This also is not called the roommate, call the classmate country middle class classmate to discuss out, or with the teacher to learn yourself? Mainly is their own groping here, and then the elder brother to give advice. So actually if say you did not attend this kind of class of words, actually if you are still in preventive medicine, actually oneself also can write these are not necessarily not necessarily right, because come in have demand, demand is to force me to find is to learn this thing, learn that kind of thing.

34:10

If I don't come in, I may have a normal class, and then I won't have this idea to find out what the need is needed. For example, if he gives you a gene, if you want to get some information about the gene, where do you find it? First to some databases, then to the documents that you published before, and then you need to learn how to do experiments. You have to go to the library to look up the books, or to search for them online.

34:54

Push you to go to force you to learn this knowledge.

35:02

Well, when you talked about your sophomore summer, there was a research team, and you had already chosen a mentor in your sophomore summer, and you were already doing that subject. Did you choose a mentor back then? When you choose a mentor as a sophomore, he will tell you wrong about 444 monopoly opportunities.

35:30

You big three when you choose the research direction, but the level direction did not say specific, you must write out at this time, hand in later can only do, and do not have this is just everyone in their own heart sophomore summer vacation this topic is the national middle class every student to participate in it? Or is like the kind of youth League committee scientific research and training this kind of thing, can participate or not participate.

35:58

Either you can attend or not, but basically everyone does.

36:07

The title is what the tutor gave you, and then you did what you are interested in this project yourself? Or in another way, after you are in yourself into the supplementary class, because of the need of scientific research forced you to check a lot of information, after you read so many books, do you feel a point of let you stimulate your interest, or a direction, or a topic, or a other similar a phenomenon, you are more interested in?

36:48

Because people want to get those unknown things, what is it? And then the scientific research is that it's not like our normal class, right? Is it all right in your hands? This research requires you to explore by yourself, to do experiments to verify. Then do you have something that you are most interested in? You just go through this and you should say that grade 17 is a senior, right? After three years of practice, do you feel anything that interests you? Or it can attract your attention, or even your attention, or the fertility of humans that you are very interested in.

37:36

Reproductive medicine, yes, because this is the main thing of reproductive medicine, I am more interested in the protection or promotion of this reproductive power, and then see some publications, introduce some scientific research results, I will focus on it, you click on it to have a look.

37:56

What is the appreciation rate, so men's sperm quality has dropped by 50% over the past few decades, and if not reversed, in how many years there may be no natural reproduction process may rely on assisted reproduction.

38:15

But if there is research found something can stop the process of decline, not more interested in this aspect, you have tried to go in this direction to do more research well, now basically in this respect of new men found something fresh, but it is too complicated, by me this one person is no way, you can only in his teacher gave a small topic to do yourself, this small topic is what you speak even set a subtopic, can say so.

39:07

Mainly because some of the things in the official website belong to this aspect. You put the direction that you want to research in your future planning, and if you have the conditions to do this research in the future, and if I have the opportunity to continue to do scientific research, I will also do the topic that really happens.

39:41

You can say that one of the things you have been most proud of or accomplished in recent years, if you have not.

39:50

Some time ago, we ourselves from the beginning, and then to the end published a small review of Chinese. Although the small Chinese books can not be compared with other people who send SCI, but this is also a one I independently completed with the help of the senior teacher, learning is equivalent to a result out.Well, it will make you feel more fulfilling and proud, right.

40:19

Then, after the summer vacation of my junior year, there was a mid-term report, and then the report results may be more recognized by the teacher. What is his recognition to? Additional funds. Your project is further funded, so being recognized by the teacher will give you more motivated to do these things.

41:02

Now we want to ask you a question is after you enter the doctoral class, will you students to ask you some questions about the activity class? What kind of questions did they asked you, or which one impressed you?

41:39

Generally, he has already come in, he will ask me which laboratory is better, or I am a selection method of choosing the mentor rotation ah, and do scientific research, and more or a sophomore year after a project project. Well, these two aspects are more problems.

41:59

At the end of sophomore year is something you just talked about in summer vacation. Does this topic come from whether your tutor turns the topic that four tutors give you, or does the tutor put a list of topics, and then you students choose it yourself? What is the situation like? For example, three people form a group, and then one of the three people mentors, he has a small topic more suitable for us to do, just give these three people, what is the standard for these three people?

42:32

There are not many standards, we will have an application form to them for a project, I will write down the background and planning, and then I will do something similar to the opening report, there will be mentors and students to listen to the below, and then basically will not pass.

42:55

Will there be competition? There will be two groups, and one project happens today, no no.

43:00

No, you've basically it in private, don't report, is that? This is a group of three of us. For case other three, also report an improvement. Then the three of them are not under my mentor, and they can't pass it.

43:19

Or a mentor. A group of the students under the name of the mentor. Not a group in the free group, right. There must be one who is your mentor, and the other people can not form teams, so there's no freshman, not sure whether to supplement the class to ask you questions. Should you come to our class? Is there any time to be a freshman during the publicity period of the event?

43:53

No one asked me, didn't it? He may ask others, if someone to ask you with him recommend I want to see his interest in the scientific research, he if he just want to put as a springboard, so or want to take thyroid or see grind comparison, so I will suggest that he or the opportunity to, so really like scientific research, even want to do some common well, but their freshman also will not have so many ideas, or to see its main purpose, so you think the main or most suitable for those who may just want to read a grind may not be suitable for this.

44:55

Say?. Or more suitable for those who want to walk all the way, to read the doctor to the research road, just want to graduate school, not very recommended, because he finally shareholders class cultivate these people, mainly want to let most of us choose south university reproductive this graduate students, but there are a lot of people will vision higher, think of some better school to read.

45:26

Then so take the fifth middle class as a springboard. To. The springboard made him jump was still unclear, because the first one was not yet at that time. I want to ask you are the kind of water and Ann, such as I give an example or what assume a situation, mentor may suddenly change a direction, may you do now and you really do different direction, want to hope you follow him to do another direction, you can accept this situation?

46:01

If there are no serious consequences, I will say what are the consequences of me? Serious consequences are what it is. For example, when I am about to graduate, I can do a little more work on this topic and then successfully graduated, but he suddenly let another one can not graduate normally, I may greatly affect your normal graduation.

46:23

For you do not affect you want to graduate, you will accept what, if there is anyway, that is to say in the choices of the most touches to your core idea core factors, or your graduation, or some of the future planning if the whole planning upset, that is the matter of time, even a year or two of this, you read the doctor, is also want to read a promotion.

47:08

Just talked about your courses in reproductive biology and developmental biology. Do you think they are more meaningful? I just seemed to hear what you say is, right, compare what do you think he brings to you? Of course, the harvest is that developmental biology is not yet on, now only on reproductive biology, reproductive biology can be from many aspects is what sex?magnetism. These two aspects can let us understand the whole direction of reproductive medicine.

47:45

Have you learned the courses you deleted from preventive medicine? Have you ever talked to your roommate? Your roommate should still be in preventive medicine, right? In the first three years, first, second, and third, are they there or normal preventive medicine. But we delete some of the class is also some didn't know can not say, understand some social psychology what this kind of economics, for scientific research and there is no substantial, may for reproductive research may not be too much help, but if you are still engaged in preventive medicine, may still want to understand the social medicine above things, right?

48:33

So they think they are waterholes, do they? Water class refers to the teacher's paper is not difficult, ah is said that the teacher class does not help them is not very much, feel that say not attendance that kind, may be a bit like some social science things. Delete. In fact, if there is not water, that is, in the senior year, he will know that we take 4 clinical courses of women and children, and infectious diseases, and then they normally prevent the oral skin diseases, ear, nose and throat nerve, these preventive medicine classes, they are more in this respect, may feel more pity.

49:19

It's a medical degree, after all.

49:21

Before will be a little I am more curious about you just said like those social science class, your classmates think is deleted, water is not surprising, they think water because the class brings them, they are from your roommate you chat with him, you feel, they think this thing brings their harvest is not very big, or say I just ready to pass the exam, no?

49:50

It's so hard for women and children. What is the main aspect? The main thing is that it did not gain very much, because he did not gain very much.He is still in school now. If you learn some, you look for professional knowledge of medicine, it will be integrated with other medical departments, but he can not use this social science here, and he may want to use it after he enters the society, he basically forgets.

50:21

In fact, I think you personally have a student of the national feelings, so you still hope that your research can have a larger application and scope for scientific research, in fact, this is quite biased to social science, in fact.

50:42

So I'm more curious about the source of your ideas. Have you ever thought about it, do you think you are different from your roommate? They may think they have thought about a test or what, but you may be clearer. The whole class or something.

51:11

Do you feel that you have and you and are different from them? Not so much ghost about this idea either, me. No, is it? To give a simple example, when the tech group was there, they thought they thought he left without coming out. Are you still in the subject group and you ever thought about why they had that idea? Or why can you stick to it? Support the support to stick to doing this.

51:38

Don't want to want to send articles or think we are into this class, Not to be some happy, Some quick, useless research, Still want to make some breakthroughs in reproduction, Breakthrough research, If you just focus on sending some of these little articles, In terms of technical research, It's really hard to make a breakthrough, You you have you have a realization that the problem is very difficult, As you all know, But if you don't try it, Then I'm afraid it is just a word, But now where the whole environment works, I don't know if you have ever learned it, Like your mentors, their research environment, Including the PPT they often say how many articles the mentor posted, Or how many influencing factors are there, Have you ever thought about it, I was assuming if I ever thought, If it is really engaged in this most basic scientific research, Maybe it does affect many more people, But for you personally, Maybe you have these impact factors or these articles may not be as strong as others, It may even affect the entire research path behind you.

52:51

Have you ever thought about the negative effects of this path may affect you?

52:57

Because now Chinese research is like a mountain theory, From the beginning, like climbing the mountain, Want to get to the top of the mountain quickly, Then to the top to enjoy those things, Just do what you really want, But the process is very difficult, You will ignore something nearby when you climb the mountains fast, Just to be intuitive, And I came out and went to work after graduation, Then I wanted to do some bigger ones, But the cycle is very long, It takes maybe 8 years and 10 years, No articles were available at this time, Maybe I was fired half way through, so I have to write from the beginning, After lifting themselves to a certain height, Then you can go and do what you really want.

53:54

So you're struggling to think about that.

54:00

It's hard, right, because I think that way, it should be in conflict with your values.

54:06

It's a conflict, because you are the most energetic, and then when you are the most creative or when you are young, and then when you are young, when you pursue some articles, what do you pursue? Seek promotion, and then you will miss these opportunities, when you really want to do something, you will find that you can't do it, without those rich ideas, how have you thought about what? Or self-digestion, or self-regulation, or simply change your mind, have you ever thought about it? I have thought, but I think it may be too far away, because I am too weak.

54:46

It's not far away. It's still a few years left to graduate. I think maybe China's scientific research field will also be aware of this problem now, and I will see if they will change. We say a relatively bad situation, if not changed, this reality situation and the conflict with your values, you, you, you are going to synthesize to mediate.

55:10

I can only like them, the first sleep, and then better words, do a higher position, and then they have a certain influence, and then put forward to change from their own respect. Because if you just want to follow your own path, you are focus on some very difficult projects and you can get nothing, the scientific research environment is really not very friendly.

55:59

You said that you wanted to be a scientist when you were a child, but it was because everyone around you said that it was influenced by the people around you. The culture around the countryside might not be high, but scientists also saw it on TV.

56:25

More face, yes, the social status was relatively high, and I did not have much understanding of this aspect at that time, at that time, who would think of these things, do you have any more? I want to ask another question now, do you think what is the biggest difference in preventive medicine when you chat with your roommate? Different or preventive medicine, it's not that you say you think it's nothing different, you can say it. You don't think it is very much. The differences are very different. Preventive medicine or is it breadth and how to intervene with it? It belongs to the primary level of prevention, but the country is mainly a depth, from a very small thing to a molecule to a protein to a Rna to a DNA.

57:30

Have you ever thought, if you haven't been to continue to learn preventive medicine, actually you can explore more and human related technology scientific research field, you enter the tourism country, actually your scope is a little is limited to death, even the small piece, you may not know to prevent many things, your sophomore directly came to even not, your research has been limited in this area, you don't know more may stimulate your interest, have you ever thought about this problem?

58:02

You guys feel a pity for me or something. Have you ever thought about this question right now?

58:06

Yes, I also told the tutor at that time, I said that we will now choose whether the reality will be in this small future, so this idea is a little much, because now the method is the same, but your things are the direction is different, the method is the same. He said that we don't want to cultivate it, but only we know something in the field of reproduction, but we don't know anything else, and that is not the students we want to cultivate. Most do we think about what did your mentor say? He said we mainly wanted to mentor your junior year, yes, you're your mentor. He means mainly reproductive this field assistant, then cultivate some scientific research method, and your ability, then you if you master, so you don't want to do promotion, you want to do cardiovascular or do something else, and then you do that is to spend a short time to understand some knowledge in the field, and then but also his method of thinking system is the same, so you can also quickly with the industry, so you also recognize his statement.

59:29

approve. To. Now it is found that it really is so. I was like when I practiced before, he will let me know, this is the research of this department, and then you say it is vivo things? To. Then then found that it was indeed this set of documents in their field, and that there was no particularly big difference. You go to the internship, for example, to see why you are asked to read their that, the department literature. They know that we mainly do top-up, such as like appreciation center, they will let us write a review of this possibility they know we this main direction is later do scientific research, or they also want to know one of their old, teach the teacher is doing what research, you think about what they are studying?

01:00:33

Well, do you think attending the fruit seed class makes you lose something?not have. Words can not say, but also lost some, can not be said to lose it is very difficult to say, or it may be that what you once wanted is to come, the pulp of the wheat may be gone later. It's not very easy to say. Not to say whether there is a later I imagined later, if my idea has always been there, but I really am not in the country, but will it be better, but ordinary medicine, clinical medicine, preventive medicine, preventive medicine is better, preventive medicine will be better.

01:01:25

The university training is that, if the knowledge is very professional, it may be a limitation. College students should cultivate their own critical thinking, some knowledge of the humanities, and some other things. You think your own is the critical thinking skills that you just mentioned, and you think you haven't learned it very well yet, have you? Because most of the time after coming in is doing in learning and doing experiments, or doing your own small topics, and then support those other things, such as the philosophy of literature, those that can expand themselves.

01:02:08

Other literacy things time will be missed, without this time. Later found is want to be something later let you suddenly think so, or what is the point, or say you have been thinking, well, initially not thinking, then I don't know why feel that college life is not just caught in their own field, things seem to look at is nothing you didn't do it? Or I read something? It seems to see a sentence who said what, as if some foreign university some thought that if college students cultivate, he is very proficient in their professional skills, university education may be a failure.

01:03:02

The main meaning is the university is not to specifically to cooperate with his skills in this field, to graduate students, college students to cultivate them to other areas like critical thinking, and the philosophy and psychological society of the kind of ideology, actually you can also, such as in their spare time to see the social science of these things.

01:03:30

Yeah, start it right now. Take the time to see it, right. I feel that you now in addition to class, such as knowledge department, and some scientific research training, in fact, what will you do? So say your interests and fun. I will exercise in the evening, and at noon, if I will sleep a little at noon, and then I will read some extracurricular books, which have just recently started the humanities and social science factors? But the progress is very slow, because the time is really too limited. The rest has no problem. Did you feel stressful in the middle class?have. Except for the jelly class, I was interested in doing those experiments in the lab. I wanted to spend most of my time on that one, but at that time, my girlfriend seemed very dissatisfied at that time, but you didn't have to go to the lab whenever you had class time.

01:04:59

He will make a noise with me, and then I think I go to the laboratory is helpful to study. Why do you have a more tense relationship with him, and the pressure will be greater. How to solve you?

01:05:17

Then each step back, each step back. You have a little more time, and they don't want that either. Is he in the national middle class? No, he's another major. He is a different major in another school. I would like to ask, what do you think is the most important thing for you in the past years from undergraduate to future? From your personal perspective, you can go on well with your girlfriend, or get a PhD, or post articles or something.

01:06:12

You think the most important thing for you is the whole stage, the stage of higher education. Is this an academic degree? Is this a PhD?get? It isn't necessarily a doctor. Do you think what I want to hear in school may be any academic breakthrough? Or said to achieve the ultimate goal or say, well, learn to learn what method or what, at least or say you are you don't carefully thought about this thing, no particularly deep thought, but at least to get the corresponding degree, read the master is a master, read the doctor will get the doctor, is to solve the problem of survival later, more realistic.

01:07:05

right. If it is good, you can do some good results, and then you can go a little more smoothly. When doing basic research, there is also a high possibility of failure. This will make you retreat, or change your studies and change to other research directions. In the future, work will not be learning. After work, you will For example, you can start to carry out research projects independently, you can declare what you want to research, you have declared a certain direction, but the possibility of success in this direction is relatively urgent, after all, it is basic research .

01:08:01

You may find in this process that the probability of future success may be relatively low, and you will continue to carry out the project. If the direction of the project is wrong, it must be stopped in time.

01:08:22

If it is more difficult, or the direction of his progress is different from what he expected, he still has to do it, because no one can say that in the field of scientific research and life science, as long as it is not wrong, then as long as it is not wrong, everyone may The chances of success are lower, but you will keep doing it. Do you feel anxious during final exams? Will it be more anxious, the stronger kind, or a little anxious? Moderate. Because most of the spare time is usually from the freshman year to now, I have been going home all the time. I am not as anxious as I was in the freshman year, and I am still more anxious than before.

01:09:11

After entering the class, until after the shareholder class, a lot of time will be put in the laboratory because of the usual spare time, and then your usual review and so on will fall, and you will be more anxious at the end of the final period. At that time, you are anxious, so It is said that after you entered the shareholder class, in fact, during the final exam period, you will be more anxious than when you were a freshman.

01:09:32

Because there is less time, but I am not very anxious, I usually feel very good.

01:09:40

Usually your input Usually when you are in class, you will still seriously go to high school to preview and review such learning. Yes, you usually pay attention to listening in class. In the end, sometimes you find that the anxiety is blind and anxious, and the test is still good. In the end, the laboratory will also give two or three weeks to review by myself.

01:10:05

If you sometimes give yourself anxiety, I would like to ask a hypothetical question. Suppose you find that you think back, if you were a sophomore and entered a middle school, you found that the direction of research did not actually bring human beings or say The whole human being and the whole society have brought about great changes, which are different from what you imagined. If it was not that kind of technical research, would you choose to quit for you at that time?

01:10:37

You will not opt out. Won't opt out because it's medical after all, and if there's nothing to human health funding, they won't carry out. Well, besides, I can do it myself after they don't start, but I still want this as an opportunity, not a direction based on what he does.

01:10:57

Opportunities are learning opportunities. OK. If you come to study there, do you mainly want to learn the skills of those research time? Still want to learn something? That is, I want to learn a set of scientific research. I have no problem with scientific research methods and thinking. No problem, I want to ask again. Because you have a passport to go abroad? Did you study abroad? No, I haven't started studying abroad yet. Does everyone have to go, or do you choose to sign up yourself?

01:11:55

Haven't heard any news about this yet? I didn't hear it, you saw how some of your classmates have already exited? Their respective colleges also have a study visit program every winter vacation, so this is not a middle school class, and the middle school class's going abroad has not started yet. When you go most of the time, do they say they told you when? Only the beginning. I talked about it when I first started preaching, but I didn't mention it much later. Pass. right. It is estimated that the situation is very difficult now. thanks.
