# Supplementary material for: Why do you choose this program?—A decision-making model of medical students based on grounded theory
Source: PLoS One. 2023 Sep 15;18(9):e0291634. doi: 10.1371/journal.pone.0291634 (PMC10503722; doi:10.1371/journal.pone.0291634)
Supplement: S1 File — (ZIP) [file pone.0291634.s001.zip › RAW DATA/P6 CHINESE.docx]

00:00

31的时候到最后录取，包括到后面的学习的整个一个过程，包括中间发生了比较重大的事情，或者你的一个让你有情绪变化的事情等等，这不仅仅可以聊事情，也可以聊你的感受感觉等等，就是什么你印象深刻的人都可以去聊。主要就是想还一个过程，其他的没有什么，就是一个简单的聊天。

00:26

然后在这个访谈之前我先读一下美女，本次访谈中受访者是在平等自愿的原则上参与的，受访者必须真实表达自我想法和认知，确认自己符合社保条件，访谈的过程会被录音，录音资料将以匿名的形式用于科研，但不会泄露给任何第三方。

00:48

在访谈的过程中以及访谈结束以后，你都有权取消研究人员的录音资料使用权，你是否知晓？你同意？同意嗯。你是哪一级的一？9级1。9级的预防吗还是？预防过程。我们问的第一个问题，你在大一你现在是2，你在大一的时候，对于国中班的了解有哪些是什么途径去了解的？回想一下当时是当时宣传的时候的事情。首先我是一开始最初听说的时候是听到别的同学谈论起，然后某某同学是国中班的学生，然后后来自己通过官网上查询搜索国中班，然后就能搜到相关的资料，然后点进去就可以看到国中班的招生简介，然后就自己了解了一下。

01:43

为什么你跟同学讨论会讨论他是国中班的学生，是就听说比如说有一些人一大堆人出来了，然后听可能有认识的，学生就说他是我认识的国中班的一个学长或者学姐之类的，然后就听到了国中班会和你们学普通学生有有什么不一样的吗？可能就是他认识这个人，然后正好就是说他是国中班的，然后他就讲出来了，然后我们就知道他们是有什么不一样的，不是。

02:19

然后你就自己去搜了一下资料，他们好像有什么你搜完资料以后，当时宣传的时候，你有跟你的辅导员或者老师或者家里人或者是同学讨论事情了？有。当时我想报国中班的时候，我跟我原学院的辅导员交流过，然后辅导员当时就是推了一个上一级的一个预防国中的一个学姐的QQ号给我，然后我跟学姐在进行深入的交流。

02:52

你原来是什么专业？原来是医学信息工程的。你是转专业的。对。你在高考的时候报名的是什么专业？医学信息工程对。医学信息工程属于工科的专业对。是为什么想要转到预防区呢？一个是因为我个人在学习一年计算机之后，感觉自己这方面的兴趣不是特别浓，然后还有我们学校预防比较好，所以我就想然后正好有一个国中班，然后我就顺便一起报了一下。

03:37

你说你在大一的学习过程中发现其实对计算机的兴趣不是很大，你当时在高中的时候报名是怎么想的？是你父母让你报名还是你自己自己就是自己搜索信息来报，还是受老师的影响来报的高考的志愿嗯。

03:55

自己和家长商量的，因为当时高考的时候其实并没有发挥得很好，然后我们学校比较分比较高的专业的话可能也上不了，然后相比较之下就自己选了一个所以你现在是认定了南医大，然后再挑专业，对先学校再选择专业，为什么会选择南医大？

04:16

因为我觉得首先南医大我觉得它转专业有一个转专业的途径，然后我就想其实一开始进来的时候想有机会的话可能会转专业，然后如果是自己通过一年的学习喜欢上工科的专业的话，我可能就不会转，但如果自己兴趣不是特别大的话，我可能就想转，然后最后的话是第二种结果。

04:39

来之前是想转哪里就是来学校之前，呃来这个学校之前，我觉得临床和预防我都可以接受，都可以考虑，都在对比之中。

04:50

所以其实选择医学信息工程的人还没有进入学校之前，就已经想好要转正了。

04:57

对，就是想转，然后有这个想法，但是先试试看，喜欢就继续去，不喜欢就要转。

05:04

当时因为在国中国中班之前其实还有一个转专业的机会，你当时有报吗？报了哪里？我当时报的是临床。你也符合转临床的条件啊，对符合。为什么选择了国中？当时转临床的话，我的细节成绩有一点不太好，然后就没有通过笔试，因为我细节大一的时候是没有这门课的，所以我学的比较吃力，所以就没有通过。如果通过的话你会选择临床，如果通过的话，那就直接进入临床学习了，那就没有之后国中班的事了。

05:51

所以其实对于临床和预防来说，你还是更喜欢临床的。不是，我其实两个都能接受，因为我当时想的是如果是进入临床学习的话，预防这方面的知识也能够学到，然后他可能面涉猎的更广一点，预防的话他是没有帮别人治病的这种能力的，所以所以我当时这种考虑，所以选了临床，但其实两种我都挺喜欢的。

06:20

挺喜欢是是什么激发了你的对他的喜好，是他的以后的工作比较稳定吗？还是？喜欢看哪方面，一个？是你本身对他学科又感兴趣，一个是我感觉学医我还挺喜欢的，还有一个感觉治病救人挺有成就感的，包括从事卫生公共卫生领域，我也挺有成就感的，包括工作稳定也算一方面。

06:47

你觉得对你影响最大的是哪一方面是成就感吗？还是稳定的工作还是？嗯别的就是让你喜欢喜欢临床成就感，因为我觉得从小受一些熏陶，我感觉医生然后公共卫生事业之类的都挺伟大的，从小受熏陶是来熏陶来源于哪里？是电视吗？还是家里电视？我比较喜欢看那种呃，医疗的纪录片之类的。

07:19

我们家没有人从事医疗相关的事，你爸爸妈妈对医疗方面医疗工作的看法是怎样的？他们都觉得挺好的，觉得都还不错，前面很聪明。前进，对，其实也不错，就他们比较务实。你当时报名国中的时候，包括报名临床转专业的时候，有跟你父母聊过吗？他们？有没有给你什么意见？他们都比较尊重我自己的想法，基本上就是表示支持我，然后也没有基本上没有提出什么别的意见。

08:03

你会朋友们还有舍友同学们聊过吗。我跟我比较亲近的人说过，然后轻轻的是你那种高中的好朋友吗？还是？对高中的朋友，还有我大一比较亲近的朋友，但是我没跟太多人说他们就对于你加入我中班有什么他们有影响到你吗？没有影响到你就可以不说。你和他们讨论的时候亲近的朋友，基本上就是他们表示很支持我，因为我之前有透露过想转专业的这种意愿，然后他们表示还挺支持我的。

08:43

所以其实你对于国中班的了解，基本上就来源于你自己在网上搜索到的那些互动班的信息，他们有开宣讲会有没有去参加？他的宣讲会是面对护理学院，然后还有预防就是工位，然后之类的学院，然后像我们生物医学工程与信息学院他没有开训练，所以我不太知道。你在了解到跟国中班有关的他们的招生的宣传之后，你觉得哪一点最吸引首先我觉得如果你的成绩达到他的要求的话，可以9年手术博这样子读下去，我觉得蛮辛苦，还有他有奖学金，一年如果达到他的要求的话，可以有1万块钱到最近这都挺喜欢我的。

09:44

你刚刚提了一点啊，就是宣讲会主要是面向护理，你们好像可能没有什么类似的宣讲会。嗯那，你的同学对这个东西的了解多吗？你刚刚说有跟你比较亲近的朋友，聊过他们在你你跟他们聊之前，他们自己有知道吗？知道的不是特别多，人对大部分人其实对这个了解都不是很多，他们是通过哪方面了解的，就道听途说，大家都是道听途说，基本上所以你是抓住了道听途说的机会，然后就报了这个活动。

10:26

对。

10:29

有没有那种可能就是有的学生有的同学根本就不知道你们班要不你或者你们学院有，我当时转走之后，我们班同学有的都不知道我走了，然后后来就突然之间发现我的名单，从那个班班级的名单里名字里就是突然没了，然后问我怎么回事怎么走了。

10:49

然后后来我就跟他们说去国中班了，然后他们就说国中班是什么就问我，根本不知道，其实所以这种同学还挺多的是吗？突然发现你走了不知道五中。我知道的个别，但我不知道其他同学是不是这样。明白了。有没有什么事情是让你坚定的要去播种感呢？有没有发生过这种事情有？有一方面的原因是因为当时有转专业，当时不是因为考试没有通过，然后有一点心理受刺激，然后自己喜欢再加上受刺激，然后就更加发奋的去学习了一下主推，因为其实主推这个课跟转专业考试的要学的内容不一样，然后我当时用了几天时间，大概就是从早学到晚然后这样子，所以就通过通过组培是要进国中班的条件是吧？

11:50

对，牺牲不对。对，所以其实你从大一开始刚进学校开始就已经坚定了，一定要转到预防或者工作呃，预预防或者临床里面去。我当时其实不算特别坚定，因为我觉得如果如果自己特别坚定的，然后最后失败了的话，其实打击挺大的，我当时想的就是试一试，不给自己留遗憾，如果没有成功的话也不要紧这样。

12:17

你当时一开始的时候说学习计算机可能跟你不太适合，是哪方面不太适合？我觉得自己可能感兴趣，还是说学得很吃力，还是哪一方面我是觉得自己可能比不上那种纯工科的，比如说南大东南之类的，出来的专业计算机出身的那些人可能竞争不过他们。

12:43

其实我的成绩在原专业的计算机还可以，但是我觉得可能竞争不过他们，所以我后来就想给自己另外找一个助理，竞争不过他们就是指以后在找工作的时候可能会比较困难。嗯那你觉得来了临床以后，或者说来了预防以后，找工作上可能会比这种工科专业会更优势会更大一点是对对。

13:16

你刚刚说就是去也没有说非常的一定要去专业转成功，假如说我假如当时转失败的话，你也可以接受继续去学公司的专业，你也是可以去胜任学习的，对我可能觉得对你来说也不是很困难，我可能会投入比以前更加的努力这样子去学它，因为其实我虽然的虽然对他不是不算特别的感兴趣，但是如果我好好学一，我坚信我自己还是能学好。你觉得你最感兴趣是什么？学科最最感兴趣的学科是什么？最感兴趣我好像没有，最其实我对像医学法学类都对医学法学之类的都可以接触，法学类是对法律类噢那，你当初选择学医，所以我还想插一句，你当时高考报名的时候有报名法学类的专业吗？我其实没有，因为我基本上报的都是我我我报的就是两类，一个是工科类的，一个是医科类的，就这两个。

14:29

其实你说你对法学挺感兴趣，当时为啥没报呢嗯？挺感兴趣，但是最后反正跟家长商量着也没报，家长不让你报。不是不让我报。分数不太够，我觉得我去的那些法学院校，如果最后去的话不太好，所以开法学的学校挺多的，什么我看审计大学都有。

14:52

可是我觉得可是我觉得不太好，要就要去一个比较牛逼的学校感觉，所以最后还是填的时候还是都填了一合共。对对对。你刚要会查一下。选择学医这条道路是有什么具体的事情，坚定了你要学习的决心，就是高中或者小时候什么时候产生学医这个想法？因为我家有人得尿毒症，糖尿病最后转化为尿毒症，然后就肾衰竭之类的比较多，像我公公然后我阿姨基本上他们都是肾不太好，然后我看到他们有的身体不好，有的已经去世了，然后心里特别难受，特别想尽自己的能力能够去挽救一下他，就算没有办法自己动手去挽救，就懂一些相关的知识，能够帮助家人更好的维持健康，我觉得也挺好的。

16:03

这件事情你嗯有没有？你现在是大二是吧？有没有参加大创或者挑战杯什么的？没有了对还没有。可有接触过科研之类的？国重就是现在在国中班之前没有。就没有从来没有接触过科研，对，你知道股东班主要是进入活动班之后，主要就是要从事科研工作，什么的我知道你是自己有了解过做科研是干嘛的？现在因为国中班都在实验室轮转，所以基本上我目前轮转了三个实验室，知道大概实验室大概是要做些什么，就比较了解，现在进入之后加深了对补充的一些了解。

16:59

你有没有有符合你的预期吗？有没有？进入普通班之后跟你本来想的差不多吗？我觉得感觉科研比我想象的稍微要枯燥那么一点，但是我觉得既然这未来以后是我要从事的工作，还是要自己主动去热爱它，虽然枯燥那么一点，但是我还是多去实验室这样子。有没有什么让你比较失望的事情发生在国中班？在国中班比较失望的是对让你比较失望的点，比如说刚刚比你想象的要枯燥乏味一些，也算有没有发生过具体什么事情。

17:45

之前国中班开过一次，全部国中班就是17 18 19的所有的国中班的学生一起开会，然后当时请了教务教室的人，然后来跟我们讲一下国中班具体未来的保研是什么一个资格，然后之前是他们就说达到一个什么样的要求就能够保研，然后但是后来他们重申了一下，说是达到排名就是预防专业的前40%的话，你就有保研的一个资格，但也不一定保研成功，然后因为这件事情大家当时都挺激愤的，后来和学校也进行了一些沟通，然后反正当时感觉有一点小失望，但是后来想了一想，就觉得自己努努力应该可以达到，所以就没有那么失望。

18:43

我不太明白什么叫到了40%有可能保研成功，要求40%就成绩在百分之千百分之跟预防或者基础的人我，基础那边我不太清楚，我就想预防了在预防专业你排到前40%，就是和所有普通预防专业的学生一起比的话，排到40%你就有一个保研的资格，但是他保研名额就那么多，如果他到时候分配给国中班的名额达不到你们国中班这么多人的话，你国中班可能要内部竞争，但原本我们以为就是你只要达到40%国中班，如果所有都达到了，所有的都可以保研，但最后发现可能还避免不了内部的竞争。

19:23

样子。后来有一些改动。你害怕产生内卷是吗？对。对内部竞争那时候还不是比成绩吗？比如说保研百分之40，你们超过了保研的名额可能能举个例子能保两个，你们有三个进来前40%，最后怎么从这三个人挑这两个人出来，他没有说吗？没有说，应该是以绩点为主，然后看辅助看科研的成果。但是当时产生大家产生比较大反响的原因是因为他进来的文件上，就是说如果达到前40%就可以保研，而没有强调说如果名额不够的话，还需要进行内部竞争这件事情。

20:12

原本我们以为就是广州班的人努力学习，然后不用可以避免那一卷就是这样。你在报名之前有注意到这个条款吗？就是前40%可以直接保研。我当时注意到，但是没有想到这么细，可能只想到前面一层。其实你当时最吸引你的还是你一开始说的可以那个本硕博，对你还是比较想读一个博士的，对我比较想继续往下读下去，所以就这个比较吸引我想读博士的原因是什么？

20:42

因为我觉得现在大家都研读博，然后提升学历也挺重要的，就必不可少，我觉得尤其是学医，你说他本科肯定不够，所以要研读，所以读博还是有点跟风的心态，就是赌博。

21:01

我觉得有一点，但是无可避免毕毕竟，大家现在都在想想往然后继续念，读博士最终还是为了为哪方面服务，是你的职业规划还是兴趣？我可能觉得更多的是职业考虑，考虑到就业的因素，所以想要读博士对。

21:25

所以当时除了这个决定以后，你有跟你的国中班的同学讨论保研的政策吗？他们有没有人说是想要退出了？有过有人说有人说过就是想退出，有考虑过这个问题，但是最后也没有退，有可能也在考虑当中。其实就是对于普通的预防医学的专业人来说，活动班的保研的机会会更大一点，可以这么说吗？对，但是好像国中班，因为他最后保研还是就保到南医大，对于那些成绩优异，然后想更上一层楼的同学来说的话，对，可能就是退出的话，它的出路会更好。

22:14

有一些可能比较优秀的同学他就这么想。你去进入中班，现在轮转是吗？对，轮转是可能要轮转4个科室，4个实验室。对，但是我有一轮的话是第一轮第二轮都是留在同一个老师实验室，所以有这样的想法。因为那个时候要考试月了，所以我不太想重新再去可以花时间再去融入一个新的城市，所以就留在里面了。是考试压力大吗？对医学生压力都挺大的。压力很大的话你会你会比较焦虑吗？我？考试挺焦虑的，我觉得。

23:08

压力我想问一下，你有没有拖延症之类的？或者说到快要到了时间了，然后快马加鞭的啊啊，比如说某个项目某某一个一个叫什么汇报的小组汇报之类的，快要到时间了，然后快马加鞭的去做。我在学校没有拖延症，我在家有在家有时候在生活里面怎么说，在如果寒暑假在家不用上学，我会挺拖延的，然后在学校我一般不会拖延。

23:44

因为事情越多，你会比较害怕，很努力的去学学某一门课或者复习某一门功课，但结果最后又有失败了，你会比较害怕这种情况发生，或者说我换一种说法，这种说法有点我刚刚说的不太好，比如说你很努力去做了某一项实验，但是最后却失败了，你会比较害怕这种情况发生吗？

24:11

这样会是这种情况会让你感到比较郁闷或者说在开始之前，他是很有可能失败的这种情况让你感到比较焦虑吗？

24:25

为什么我在开始之前就会觉得他会失败？你在预判的时候，你在做某件事情的时候，你肯定会去想一下这个实验是否会成功，你会考虑这个问题。我应该会考虑，但是就我比较害怕失败，但是我在做的时候应该不会想到他会失败，我肯定希望他会成功吗？我可能不会一开始就想的那么悲观。但是其实对于从你的个性来出发的话，你还是比较害怕失败的。对，我肯定比较害怕失败。

25:04

这些压力除了学业上考试的压力，有没有其他的压力？考试压力，还有怎么说科研也算是一种压力，因为我看我周围的有的同学有的已经加入课题了，课题组，然后就是在跟进课题了，然后有的导师可能对他们更伤心一点，然后会给他们一些任务做平常，然后你的导师没有让你觉得因为我们那边一个有个比较特殊，有个导师他是从一开始就要求说全国的同学，然后就一直本科阶段都要跟着我就不能换导师了，然后一开始就让他们加入课题组去跟课题了。

25:47

然后像我们一直在轮转的话，是因为半学期就要轮转一次，所以导师不会对你特别伤心，因为你最后不一定会留在他那里。

25:55

所以就可能没有那么深，就个人老师没有对。

25:59

你很伤心是吗？不是对我不上心，这个就是普遍的一个现象。就是因为因为你只是在他这里轮转一下而已，不会留在他那儿，所以老师肯定不会对你特别对你会觉得学到东西，学到了一些基础的实验操作，然后看了一些论文。是谁教你的呢？是师兄教你的，还是你自学的？都是平常在实验室跟师兄师姐这样看实验看，但是自己做实验的机会目前还不是很多。你觉得学到这些东西对你帮助大吗？对于我大三，因为国中大三他有一个国中班内部的一个什么一个比赛，好像要互相组队，然后完成一个课题，有这些基础的实验操作的一些基础，到时候完成肯定会有帮助的，我觉得。

26:50

实验室你是前两个都是在一个实验室是吗？你喜欢那个氛围吗？人文环境什么的。我觉得前两个的实验室其实我我现在有点后悔，我觉得有点选错了，因为那个实验室的导师他是一开始让我就是读文献，然后让我少做实验操作，从文献入手，但是我个人的规划其实是先学一些实验操作，然后再就学习完这些基本操作之后再去读文献。

27:18

然后这样子的一个顺序就是一开始他让我读文献的话，我其实实验操作都是自己去求着师兄教我的，所以就觉得有一点选择其实不太对吧，可能是因为我当时选之前做的了解比较少。你有没有跟老师聊过，就是谈谈你自己的想法？聊的也比较少，是因为老师会定期的发一些文献给我让我看，然后最后他给我的任务把文献就写一篇报告整理给他这样。你没有跟他聊过，二老师其实我更想多学一下，先学操作，在看文献。没有聊过。所以这会让你感觉你的收获感可能会没有那么的高。可以这么说。

28:12

你可以跟我们说一下你读就是从应该可以说从读大学开始，让你感到你觉得最自豪的一件事情，或者说你做了一件让你非常有成就感的事情，有一件事情有吗？必须要跟国中班相关吗？不用。生活的也可以，感情也可以。我觉得学生会什么的都可以印象最深的。没有吗？自己有一次做为国教院的中秋晚会的负责人，然后成功的策划了一场中秋晚会，这个事情让我挺自豪的。

28:58

大一的时候，还是大二的时候，大二刚开始的时候。主策划的。对，包括安排，然后设备之类的都是我负责国教院的中秋，对。因为他跟我们部门，我原来是想你怎么会去国教会帮国教院做中心晚会，因为他当时跟我们部门要接设备，然后跟我们对接。

29:27

我是校团委办公室的，就负责这些灯光音响之类的一些设备。你从大一开始就是进入了这些像类似学生会团委一些组织是吗？你觉得收获大吗？在之前？受团什么的，我觉得收获挺大的，收获最大的是认识了很多别的专业的同学，他们给我一些学习资料，对我学习上有很大帮助。学习上的工作有没有别的？就跟他们关系也很好，有的时候会一起出去玩之类的，生活上也有。

30:04

你可以跟我们说一下，你现在加入了几个组织，一个是团委的学生会还有别的吗？我其实现在都基本上之前参加的都退了，我大一的时候还在医院。团委待过，当时做到了科创部部长，然后后来因为退出了就就转专业了之后就退了。然后现在的话社团就大一就是加入了校校和院，对。社团没有吗？社团有，但比较杂，就什么羽毛球社，然后滑板社，计算机设，然后都退了是吗？没有退，但是参加活动参加的比较少，现在的话主要参加滑板社的活动，有的时候放松一下，周末会去滑板。

30:55

所以说你你当时说大一这些社团主要都退了。其实我说的是大一的组织退的就是医院的，还是因为转专业，所以才退了。假如当时没有转成功的话，还是会继续在里面做的，对。因为毕竟当时其实刚竞选上部长，嗯对就后来因为我退出了，所以部长就换了一个人就换了，对。

31:26

你说中秋晚会是让你最有成就感的一件事情，他最让你自豪的一个点是什么？可以跟我们具体说一下。因为之前我一直都是听这个部门的其他的负责人，然后来说应该怎么做，然后自己去听吩咐去做事情，然后那一次是我给别人下达一下指令，然后让他们去完成，然后包括自己要负责整个会场的统筹，我觉得那一次挺骄傲的这件事情，因为是我第一次尝试自己一个人去统筹这些工作。

32:03

你在校团委嗯没有做过这些统筹的工作吗？我一直都是去帮忙接设备的一个工作。那一次是自己第一次统筹，所以我可以说自豪感来源于你做了一件以前没做过的事情。这样描述准确吗？不是特别准确。你觉得自豪感来源哪里？是来源于呃完成了一件我觉得比我以前做的做过的事情难度更大，然后而且完成的比较好。你可能刚开始接触的，任务可能觉得不太能完成，我觉得对我来说是个挑战，对最后成功的做完了，这是最让你自豪的一个点。

33:06

我现在想问一下，大学这几年，你觉得对你来说最重要的一件事情是什么？是完成学业，还是说比如说团委在做到副主席主席之类的事情，或者说科研上面有什么成就，你觉得对你来说最重要的事情是什么？有想过这个问题吗？想过，但是我觉得首先就是团委那个事情，我可能不打算再继续进行选了，因为我国中班挺忙的。然后但是学业和科研我希望能够达到一个平衡，就是在我学业不影响排名的情况，之下呃科研也尽量能够做到很好，因为我觉得这两个没有办法做一个重要性的权衡。

33:55

所以你觉得团委的事情其实是有干扰到你的学业的。对，其实他那边挺忙的。你打算明年的时候退掉是吗？对应该是大三退嗯，但是你刚刚说你觉得最自豪的一件事情是中秋晚会，你有没有想过可能在团委里面，你才能更多的接触到这些比较大型的会议组织或者说活动组织？可能以后退出了以后就接触不到这种可以这种高挑战高挑战高难度的事情。

34:35

我想过这件事情，但是首先我觉得我一直都把学业和科研这两件事情放在更高的一个位置上，就是在我都很忙的时候我必须要舍弃掉一个的时候，我只能舍弃我其实大一结束的换届，大家都会有那种负责人换届，对校团委的主席之类负责人换届，当时有竞选过，但因为准备的不够充分，其实没有竞选上，这样就其实当时也是想过继续往上竞选，但是后来成功进到国中班就就想以学业为重，对，所以渐渐把这些事情都放下了，其实现在也不是太去忙那边的事情。

35:32

国中班有没有让你感觉压力比较大的事情？

35:37

科研上面有没有？科研有，因为他每周不是要求我们轮转，要他至少要求我们要到实验室去轮转三次，然后做实验的话一般时间比较长，然后学业平常课程也挺多的，比较占用课后的复习一些课程的一些时间，然后其实给我挺大压力的。

35:59

科研占用了太多理论学习的时间。对。你读中班以来就给你们一个最大的感受是什么？或者说比较难回答的话，你可以跟我们讲一讲，你觉得大二的这一年就进国人班以后有什么最大的收获吗？我觉得没有的话，你觉得没有什么区别，也可以说。我觉得有一点区别，就是我觉得我现在认识的人比我以前认识的同学可能学习能力上更强，然后跟他们能够学，就是他们能够更好地带动我。

36:48

因为其实我觉得以前班上的同学他们可能有一些对于学习上面不是特别上心，然后包括整体班级的成绩的水平也没有现在的高，现在同学他们都比较用功比较卷，然后你觉得原来的那种环境会影响到你学习的动力吗？因为周围人都不太学习，所以可能你也你可能也就没有那么大的强的学习的动机。

37:20

是是是，我可以这么说吗？也不算太准确。平常我之前班级他其实学习大家就就有一些人有很多一部分人就包括尤其是一些男生，他其实成绩不是特别好，然后学习欲望也不是特别强烈，但是我其实身边的一些女生还是比较用功的，但因为他们就、班级水平、成绩不是特别好。然后当时维持一个排名也不是特别困难，然后就导致我没有更大的动力去学习这样。

37:55

所以你的意思就是到了一个新的班级，其实班级周围的学生可能更优秀了，你这种环境会促使驱使你去更努力的去学习，对对对现在的话不敢松懈，对。

38:11

他们说同学可以带动你，其实带动的主要是他们给你带来的就是学习可能学习排名上你可能就会嗯和用以往的那种学习的精力的分配的话，可能到了现在新的班级可能会我排到后面去，这种压力会驱动你去学习，所以他们带动你是在这个方面去带动你。

38:40

我就想问一个问题，我还是一个假设题，班级就是班级考第一和学习了一个新的技能，哪一件事情会让你更加兴奋？是什么方面的技能呢？你一直想学到的比如说某一个科研方法，实验方法或者说写文章的一个理论研究的方法，实验方法都可以你想学到的一个东西。你一直都想学到的一个技能，哪一件事情会让你感觉到更兴奋？

39:19

比如说你是想学做某某某一样实验，其实那个时间挺难的，然后最后你学成了和你复习了很久，考到终于考到了班级第一，你觉得哪些事情会让你更兴奋？可能可能还是班级第一班级会让你更新，对，但是我不是其实我没有对自己要求那么高，就希望自己班级低，但如果考到班级第一，我肯定会更兴奋一点。

39:58

所以我觉得你是一个挺，挺追求成功的一个人。你有没有去想过自己，你也在科研轮转，有没有想过对生殖这方面的兴趣有没有？很新颖。你觉得你对他感兴趣吗？目前为止？目前为止还是挺感兴趣的，因为我觉得甚至怎么说，我觉得它意义还挺大的。因为现在好多就是那种不孕不育的问题，然后未来如果能够帮助这些人，感觉意义挺大的。

40:57

你有去了解过预防一些其他的方面的东西吗？有，比如说他们未来可能是从事什么疾控海关之类的，假如了解了这些东西以后，你会感觉到会对比如说疾控这方面的东西会更感兴趣，尤其是发生了疫情以后，我可能目前还是对国重这方面就是升值更感兴趣一点。

41:27

嗯因为就是因为亳州还给我们开了很多的像选修课之类的，就对这方面可能更了解，然后兴趣更浓一些。你说更了解是不是会不会或者说换种说法说因为你学习了很多有关于升职的基础，所以唉你有学习的基础在，所以你会更想要去从事这个东西。有没有这个因素？在有。有你提到选修课。因为上过中班之后，他的课程设置也发生了一些变化，好像有删掉一些课程是吗？对此有什么看法？我觉得他升课程的同时他也加了课程，然后我觉得可以理解，因为毕竟如果我们光加不删的话，是的那那些课程你会觉得就是说有一些其实不应该被删掉。

42:32

我觉得山科你了解那些被删掉的课吗？我知道，就上学期其实他有的是改了一个顺序，比如说他们有的预防的是上一学期学的纲要，我们这是这学期学的纲要，但这学期的话他们有一个寄生虫，我们没有学寄生虫，但是我们多了一个生殖生物学，我觉得反正深圳也没有太大的感觉了感觉。毕竟学本来感觉学也挺重要的，就学那么多挺累的。

43:06

新加入的那些课程，你上课以来感觉怎么样？感觉对你的帮助大吗？不知道怎么去应对考试，因为它其实就给了我们一本生理生物学的书，然后那本书特别的厚，但感觉国中的老师备课的时候跟书的关系不是特别大，就我感觉好像又要以PPT为准，但是感觉 PPT他讲的东西又比较的就跟别的课程感觉不太一样，就比较的只是你找不到知识点的那种感觉，就不知道怎么去应对考试。

43:47

主要是考试困难，如果不适应对考试你有没有觉得学到东西了？有，他介绍了一些比较前沿的技术知识之类的，觉得这些对你的帮助哪对哪方面有帮助，是对你将来就业还是说你感兴趣的方向？比如说他应该是对我们首先就是一个科研思维有了一个启发，就是他给你一些思新的思路多，见见那些新技术对你开拓思维还是有帮助的。

44:21

你刚刚说来做科研以后，科研你想象要枯燥，我也从和你谈话的时候，我也感觉到你还是一个蛮喜欢和别人交流的一个同学，可以这么说吗可以你你觉得这个枯燥其实可能不会和你的个性有一些冲突。

44:40

我觉得会有，因为他们在实验室里有的时候做一个实验，可能要待上好几个小时，然后人是空间比较狭小，就感觉给我感觉比较压力一点。

44:51

你有想过这个问题吗？因为你刚才也说了你想读博对吧？以后做就是读了硕，读博其实还有很多很长的时间，你有没有想过以后可能很长一段时间都是这样，在实验室里面待着怎么去调节它你，有想过这个问题吗？调节你的压抑情绪，可能做实验和你的个性有一点冲突，你有想过问题吗。

45:17

我想过，但是我当时想的感觉可能是因为有可能是因为我现在本科阶段要学的课程还比较多，然后实验这个不算是我的本职工作，然后就有一些别的压力给我强加过来之后，我会感觉比较压抑，但如果到了我读研的时候，我可能做实验就是我本职工作之后，我就不会有那么多的别的压力来干扰我，就可能没有现在这么压抑，我现在有一部分压力可能还是来自于平常的课业比较繁重，然后还要花很多时间在实验室里，然后给我带来了无形的压力。

45:54

所以就是说你觉得你你现在是一个以学习为主的状态，在学校以后做实验了以后可能就没有那么多学习的压力了。

46:06

你有没有？一一我有两个问题，一个问题就是你对于你现在这个状态来说，其实科研你没有做的很好的话，其实对你来说也不会有什么影响，会有影响吗？比如说你可能做了很久时间也没有发出来文章，对你以后升学会有影响吗？

46:29

升学应该不会有影响，因为现在政策改革之后，不会说因为你科研做得特别厉害，成绩不达标就直接让你保研现在的话还是以绩点为主，所以我跟你说科研的压力是你自己给自己，是这样的吗？你有自己上更向上的一个进去的一个动力在，所以你就想给自己施加一下科研的压力，想多学一点科研的方法之类的。

46:58

可以这么说吗？就一部分是我自己的压力，一部分也是因为周围同学他们在科研上也比较用功，然后我也都看在眼里，对这个环境其实对我也产生了影响。

47:12

我想问的第二个问题，你说了以后就到了硕博阶段，可能上课就没有那么多了，可能读书的压力就没有那么大，但是我现在是提出一个假设题，比如说你在做了某一项实验以后，进入到一个瓶颈期，你可能需要通过学习和阅读文献来解决瓶颈，实际上某种意义上程度来说也是相当于像你现在这个阶段的一个理论学习的一个过程你，有没有想过其实像在实验室遇到瓶颈，然后自己再去学理论学习，其实也是会影响到你整个实验的进度，这会不会也是一种压力的存在？

47:57

也算，但是就是现在跟我现在相比，因为我们还有你之前有想过我提的假设吗？之前没有想过这个问题，你刚才想想说什么？以后虽然说做实验的时候遇到这些困难可能要去查文献，但是现在我们不仅需要看文献，然后看实验，平常还要上课，然后课后还要完成作业，就压力可能更多一些。

48:27

到时候看文献我觉得呃，因为我知道以后做实验肯定要看文献的，就已经有了一个心理准备，就可能不会再换一种说法，说会不会我想说问一下我的想法对不对？你的压力会不会是来源于就是硬性的要求，比如说考试，比如说作业，这些东西是你必须要做的。

48:50

这样你可能你做了实验以后，你自己去自主学习，你去看文献，其实你你今天不看明天看都一样，对你来说只是对你来说你的实验周期变得更长而已。

49:02

但是并没有人去要求你一定要在今天把把这个理论综述给写出来，是挺准的我觉得是吧？嗯嗯嗯。下面一个问题是嗯，你现在已经进入大二了对吧？其实马上可能应该快了。还没有快，可能到下个月才会开始果冻班的宣讲。

49:34

现在有学弟学妹来问你这个问题吗？关于狗中班的事情有两个，之前他问了啥呀，就问国中进国中有哪些要求，对成绩方面，然后国中班怎么样，就跟他们介绍一下主要是怎么样，这个问题好难回答，你怎么回答？我比较怎么说就跟他们你是官方的介绍了一下。

50:02

对比较官方的介绍一下，没有太带个人感情色彩这样，因为我觉得就每个人感觉不一样，不能太误导他们。噢你你害怕误导他们是害怕哪一方面？他们被你的信息给误导，万一我觉得国中班，如果我就是觉得国中班特别好，我跟他们说之后，他们进来之后，如果觉得不好，可能会怪我误导他们。

50:23

是这样，你会比较推荐他们进入这种班吗？我比较推荐，因为我其实问的都是我之前专业的直系学弟学妹，我还比较希望能多几个之前他们是怎么知道的。他们通过我知道的，因为他们知道上一届有个学姐转走了，你在你之前就是18 17级有人转走吗？18级转了一个，然后19级就转了我一个，一共就两个人，我们当时有问你18级的转走的同学我问了，我就是跟他深入交流了，具体什么问题我有什么问题都问他，然后他都跟我回答了。

51:08

你当时问他最多的是哪方面你还记得？我记得，因为当时印象特别深刻，就是当时转专业考试结束之后，然后听说不久之后会有一个国中班的招生，国中班的一个笔试通知，然后笔试通知的时候转专业结果出来了吗？出来了噢就出来了，然后当时没有过，然后我就已经开始想这件事情了，但是通知迟迟不下，大概过了10天左右才下，然后那个期间我一直特别的焦虑，我一边在准备一边在想他会不会今年不招了，然后就一直在问学姐怎么回事，问学姐就安抚我说没有那么快，可能要再过一段时间才出来。

51:51

所以你当时是非常害怕，他直接不招了。

51:56

不招的话，你的焦虑是来源于可能还要继续学工科这个专业。我的焦虑当时是来自于可能十几天，因为当时快开学了，这个时间我玩不好，为什么要在这边苦苦的学习？然后假如说他不招了，你学了不是白学了，是是是这个吗？明白。高中班期间就是或者说从可以说大一的事情吗？就是说国中班期间有什么让你印象最深的事情？有吗？可以说？一件吗。没有就算了好像。没有什么特别印象深刻。

52:41

我中班跟你就进入工程班之后，你觉得跟原来的预防有没有什么特别不同的地方？没有什么特别不同，因为其实目前大部分的课还是跟同学一起上课，但可能到大三之后，大家要分开的时候，目前为止还没有什么不同。我还想问你跟当时学姐问的问题，你就一直在问他怎么还没有开始报名，没有问过他别的东西，我问了。

53:18

但这个问题你刚问我是不是印象最深，然后我就说了就我还问了他，他当时的排名是怎么样？考试难不难，考试考哪些题型，然后你说入学的考试吗？国中的入学入学考试，期初中的期末考试，国中的就是选拔的笔试选拔考试。你以后是想读博士吗？你？觉得博士班的我是不是问过这个问题了，中班的学习有没有达到你的预期效果？

54:08

问过了，我想起来了你有没有之前有出国过吗？应该没有。没有说19级，因为有些刚进来，然后就我提醒他，但我上次参加了一个线上的交流，就是他原本是如果是不是疫情的话，应该是要出国交流，但是上一次是因为疫情，然后只有一个线上的学习。

54:34

学啥？是国中班还是不是国中班，就是学校申请的国教院那边组织的。是学什么内容？我当时报的是新官疫情的房屋防治政策，反正就跟公共卫生有关系，跟跟谁一起做现场交流？就跟美国威斯康星大学请了一个教授过来教我们，你是通过什么渠道了解到可以参加现场的辅导员发群里，然后报名的宣传是吗？对。你觉得交流就是有什么收获吗？我觉得当时跟我们上课教授他其实不是预防，然后公共卫生专业的，但是另外一个专业别的什么专业我忘了。

55:31

然后他当时讲的内容其实跟公共卫生相处的比较少，大部分他请了一些他的朋友，然后来介绍一些中国和美国的一些特色。还有我印象特别深的是他有一次让我们介绍自己的红场，然后其实更多的是锻炼的英语能力，然后感觉专业知识方面没有太大收获。又有什么兴趣爱好吗？兴趣爱好。什么都得说，啥啥都能说，不一定要学习。跑步，滑滑板，然后追剧。我刚想问啥跟科研导师接触的时候，跟科研导师接触，你目前是接触了一个导师对吗？上个学期上轮转。

56:35

我现在是到第三个到已经到第三个了。所以你就是相当于你这一个学年就是三个导师，因为你有一个导师是连续转了两次，转了一次多转了一次。是这样的，就是三个老师你在后面选导师的时候，你会从这三个导师里面选，还是从别的导师里面选？我应该就会从这三个导师里选，因为到时候大三国中班的要求是你尽量要稳定下来，稳定在一个实验室了。然后如果你没有去过那个老师的实验室，那个老师不一定会收你，因为他根本不了解你。

57:11

你现在有想过选谁了吗？是基于什么样的标准，有想过选谁，但是目前还没有确定，因为其实第三轮轮状也是这个星期才刚开始我还没有见过第三轮的导师，然后我目前比较倾向于第二轮的导师，因为实验室的师兄师姐人比较好，经常都会带着我做实验比较多，然后也教了我一些怎么样做文献汇报，制作PPT，然后读文献上方面的一些技巧，还借书给我看，然后我觉得很温暖，所以我比较喜欢实验室的氛围，目前比较偏向于第二个，但是可能具体最后的确定要等我轮转完第三轮之后才能确定。

57:52

你在选导师的时候会考虑他们研究的方向这种问题吗？比较其实比较少，是因为我觉得目前没有哪个方向说特别吸引我，也没有哪个方向我特别讨厌。所以我目前选的原则是我觉得哪个实验室的氛围我更喜欢。

58:11

你说他们学长学姐会给你借书，你看是借什么类型的书啊？分子生物学借了一本这么厚的书，你分子生物学应该是你后面要学的课吗？还是为什么要建这个书？是师姐推荐给我的以后做实验要用到这个知识，他觉得这个知识比较基础，然后我先从知识先分子生物学不应该是是是需要学的一门课，我们已经学完了大二上学的生物化学与分子生物学，但是我觉得里头的不够教材里面的，东西不够，介绍了很多，比如说基因敲除之类的一些技术比较基础的东西，就是他借给你的书里面。

59:05

对。

59:07

但这个东西可能教材里不会讲基因敲出这些东西，比应试更多，然后这个的话就课外科普的东西更多，就是操作性更强一点的东西讲得更详细。所以目前为止你在轮转的时候也应该也没有说就动手做实验有吗有过，但是比较少，只是说基础的一些操作，你还没有独立自主的开展一个项目，目前还没有。

59:55

你们读博这种班是不是假如说保研了就必须要保本项？对。是他要求你们这样做的吗？保研不是说拿了一个保研名额，你就可以去自主的去报别的学校吗。不可以，国中班就只能报南医大，然后生殖医学这个方向的。这么我报别的学校会有别的后果吗？这个没试过，因为还没有毕业生，我不清楚，所以好多觉得就不想继续读这个方向的人他都退了。

01:00:27

这也算一种限制，毕竟他对你就有了优惠嘛。退了是就是说你的同学吗？还是你的上面上面上面，我们这一届还没有退，我们之前群厅那么积分为什么还不退？是想再学学看什么？这个消息是在你是之前你学长学姐就告诉过你，还是说今年或者去年下半年才告诉你们？当时刚进国中班的过了一个月左右开了一个会，然后是请了呃，好多学校领导也过来就听。

01:01:06

这样当时你的学长学姐就你进来以后，学长学姐有跟你说过他们之前有听过这个消息吗？就是前40%不一定能导演。当时没有人提过，但是当时会上后面坐了一一排学长学姐，他们就说嗯什么他们也是第一次听到这个消息，不是他们好像是说这个跟他们上一次听到的又不一样了，然后说什么又改了，可能之前已经变过了，然后现在又在变。

01:01:33

然后上一次我们就要求当时是请了哪个副校长不记得了，让他做一个承诺，然后当时副校长当时在外地出差，然后后来又特地飞回来，然后给我们国中班的同学做了一个承诺，就是说规则就是这样。

01:01:52

就应该不会变了承诺。是他承诺了什么呢？但是没有承诺说就是按照前40%有保研的资格，但是不一定保研的上，但是他会尽量为我们争取名额，因为所以他也没有把握。把那个话给说对。他没有一个纸质的，但是当时好多同学应该录音了，我觉得但是他说了这个话，其实也没有把自己限死，就是你们进了前40%，他还是可以让你们不保研。

01:02:24

他说的那些话。对，他也没有说你们进了前面人其实就一定能保研。所以录音也没啥用，他其实也把这个话留了一点空间给自己对。

01:02:40

所以你们其实我可不可以说你周围同学还蛮重视保研的资格的事情挺重视的，你觉得你周围同学他们有像你这样想读博的多，还是说他们只是想读个研究生多一点？我没跟他们细细讨论过，但反正起码我知道大家肯定都想读研究生是肯定的。

01:03:06

你觉得你读博的东西大吗？比如说我换一个说法，可能这个是5+1+3，你到了第6年的时候，可能就是假设答辩失败了，然后可能要再读个三年的硕士，再加三年的博士，你会继续选择这样读下去吗？还是读一个三年的硕士就结束了？还没想过。为什么失败了之后要再读三年的硕士，是因为本来是5+1+3，第6年你答辩成功了，是直接进入博士阶段的学习吗？假如说答辩失败了，可能就是要延期延到第7年或者延到第8年就还在硕士阶段。

01:03:43

其实在这种情况下来说，你和那些考研考进来的，或者说其他保研保进来的，普通的比如说研究生和他们的时间时长不是一样的吗？你会继续再去赌博吗？如果时间太长我可能不会读了，但是如果时间就是一年一年还能接受，我觉得因为因为因为以前要读很多年，其实我说的是什么意思，可能我表达不清，不是很清楚啊。

01:04:16

比如说你到第5年结束了，到了后面的研究生了，你的硕士又延了两年，其实你六在第8年的时候，你是有一个选择的是继续读博还是去工作，因为对你来说你选择两样对你来说学制时长都是一样的，都没有什么区别，你会更倾向于选择读博还是去工作？研究生毕业的时候，这个问题我好像还没有想过这个问题。

01:04:50

以后工作你有什么规划吗？在大学做老师做实验员还是可能会去说是什么都会试一试。生殖中心可能目前是我比较理想的一个规划，进了国中班有改变的，到你的职业规划嗯有因为之前我们宿舍他有一个同学他特别关注像做叫什么国考，神口。还有什么叫什么生来着？什么叫什么对选调生。就这种事情，然后你的舍友对舍友，然后她跟我们宿舍介绍过，然后大家都觉得好像挺不错的，然后都有把列入自己未来可能想做的一个想走的一条路这个方向，然后有也之前读工科的时候也想过未来可能会去一些像阿里腾讯之类的大公司，就比较想去这些大公司去，然后目前但是现在读了生殖医学这条路的话，就是方向其实发生了很大的改变，就目前比较想去生殖中心，你在大一的时候也考虑过要走去想考公，也想过那你现在完全没有这种想法，还是说也不排斥，但是不是你的主要选择。

01:06:25

了对。

01:06:26

因为我现在还是想把硕士博士都念完这样，或者说我换一个说法，你毕业了？

01:06:35

本科毕业了，你考工考上了，然后保研也保上了，你会选择哪条路？这好难写。我可能暂时不我还是会继续念书，我感觉。原因。为什么？因为他不是每次毕业都可以考，但是我还是想提升学历为主，因为我觉得现在就考公务员，可能以后你工作之后发现自己学历不够，还要再继续念书，所以其实最后还是为就业服务，提高学历是不是更好的机会，对。其他没有了有。我觉得该按照我这里你可以稍等一下好的。
