# Supplementary material for: Why do you choose this program?—A decision-making model of medical students based on grounded theory
Source: PLoS One. 2023 Sep 15;18(9):e0291634. doi: 10.1371/journal.pone.0291634 (PMC10503722; doi:10.1371/journal.pone.0291634)
Supplement: S1 File — (ZIP) [file pone.0291634.s001.zip › RAW DATA/P6.docx]

00:00

31 hours to the final admission, including the whole process of learning later, including a relatively important thing happened in the middle, or one of your things that made you emotionally changed, etc. This is not only for talking about things, You can also talk about your feelings, etc., that is, you can talk to anyone who is impressed by you. The main thing is to return a process, nothing else, just a simple chat.

00:26

Then, before this interview, I will read about the beauties. In this interview, the interviewees participated on the principle of equality and voluntariness. The interviewees must truthfully express their thoughts and cognitions, and confirm that they meet the social security conditions. The process will be recorded, and the recorded data will be used for scientific research in an anonymous form, but will not be disclosed to any third party.

00:48

During the interview and after the interview, you have the right to cancel the researcher's right to use the audio recordings, did you know that? do you agree? Agree um. Which level are you at? Level 9 1. Level 9 prevention still? prevention process. The first question we asked, you were in your freshman year and you are now 2. When you were in your freshman year, how did you learn about the middle school classes? Think back to when it was a time of propaganda. First of all, when I first heard about it, I heard other students talking about it, and then a certain classmate was a student of the middle school class, and then I searched for the middle school class through the official website, and then I could find relevant information , and then click in to see the introduction to the admissions of the junior high school class, and then understand it for yourself.

01:43

Why do you discuss with your classmates that he is a student in the junior high school class? You just heard that there are a lot of people coming out, and then you may know someone, and the student says that he is a student from the country I know. A senior or senior sister of the middle school class, and then I heard that there is any difference between the middle school class and your ordinary students? Maybe he knew this person, and he just said that he was in the middle school class, and then he said it, and then we knew what was the difference between them, no.

02:19

Then you searched for the information yourself. They seemed to have something. After you searched the information, did you discuss things with your counselor or teacher or family or classmates during the promotion? Have. At that time, when I wanted to enroll in the middle school class, I communicated with the counselor of my former college, and then the counselor just pushed the QQ number of a senior from a senior prevention middle school to me, and then I followed the school. Sister is having an in-depth exchange.

02:52

What was your major? It turned out to be medical information engineering. You are changing majors. right. What major did you apply for when you entered college? Medical Informatics Engineering. Medical information engineering belongs to the professional pair of engineering. Why did you want to move to the prevention zone? One is that after learning computer for a year, I feel that my interest in this area is not particularly strong, and our school is better for prevention, so I thought that there happened to be a junior high school class, and then I signed up by the way. a bit.

03:37

You said that during your freshman year, you discovered that you were not very interested in computers. What did you think about signing up when you were in high school? Did your parents ask you to sign up, or did you just search for information to sign up, or did you apply for the college entrance examination under the influence of your teacher?

03:55

I discussed it with my parents, because I didn’t perform well in the college entrance examination at that time, and if our school has a higher score, we may not be able to get into the major, and then I chose one by myself, so you are now It is the identification of Southern Medical University, and then the choice of majors, and the choice of majors after the school, why did you choose Southern Medical University?

04:16

Because I think first of all, I think there is a way to change majors in Southern Medical University, and then I thought that when I first came in, if I wanted a chance, I might change majors, and then if I liked it through a year of study If I major in engineering, I may not transfer, but if I am not particularly interested, I may want to transfer, and then the final result is the second result.

04:39

Before I came, I wanted to transfer where I wanted to be. Before coming to this school, er, before I came to this school, I felt that both clinical and preventive measures were acceptable to me, and they could be considered, and they were all being compared.

04:50

So in fact, people who choose medical information engineering have already decided to become a regular before they enter the school.

04:57

Yes, I just want to switch, and then I have this idea, but try it first, keep going if you like it, or switch if you don't like it.

05:04

At that time, because there was actually an opportunity to change majors before the middle school class in China, did you report it at that time? Where did you report it? I reported it was clinical. You also meet the conditions for clinical transfer, yes. Why did you choose middle school? At that time, when I transferred to clinical practice, my grades in details were not very good, and then I failed the written test, because I didn't have this course in details when I was a freshman, so I had a hard time studying, so I failed. If you pass, you will choose clinical. If you pass, you will go directly to clinical study, and then there will be no middle school classes in the future.

05:51

So actually for clinical and preventive, you still prefer clinical. No, I can actually accept both, because what I thought at the time was that if I entered clinical studies, I could also learn about prevention, and then he might cover a wider range of topics, and he didn’t help others in prevention. The ability to cure diseases, so I thought about it at the time, so I chose clinical, but in fact, I like both.

06:20

I like him. What inspired you to like him? Is his future work relatively stable? still? Which aspect do you like to see, one? It is because you are interested in other disciplines, one is that I feel that I like studying medicine, and the other is that I feel a sense of accomplishment in treating diseases and saving people, including in the field of health and public health, and I also have a sense of accomplishment, including work. Stability is also one aspect.

06:47

What aspect do you think has influenced you the most is the sense of accomplishment? Still a stable job? Well, the other thing is to make you like the sense of clinical achievement, because I feel that I have been nurtured since I was a child. I feel that doctors and public health are all great. Where does the nurture come from? Is it TV? Or the TV at home? I prefer to watch those kind of uh, medical documentaries or something.

07:19

No one in our family is engaged in medical work. What are your parents' views on medical work in the medical field? They all feel pretty good, they all feel pretty good, and they are very smart ahead. Going forward, yes, it's actually not bad, as far as they are more pragmatic. Did you talk to your parents when you signed up for high school, including when you signed up for a clinical transfer? them? Did you give any advice? They all respected my own ideas, basically expressed their support for me, and then basically did not put forward any other opinions.

08:03

Have you chatted with your friends, roommates and classmates? I told someone close to me, and then lightly is your kind of high school friend? still? For my high school friends, and my close friends in my freshman year, but I didn't tell too many people what they did for you to join my middle class. Did they affect you? You can say nothing if it doesn't affect you. When you discuss with them, your close friends are basically the ones who expressed their support for me, because I had previously disclosed my desire to change majors, and then they expressed their support for me.

08:43

So in fact, what you know about the junior high school classes basically comes from the information about those interactive classes that you have searched on the Internet. Will they participate in the lectures? His presentations were for the School of Nursing, and then there is the Prevention is the Workplace, and then the schools like that, and then like our School of Biomedical Engineering and Information, he didn't have training , so I don't know much about it. After you learned about their admissions promotion related to the middle school class, which point do you think is the most attractive? First of all, I think if your grades meet his requirements, you can continue to study like a 9-year surgery doctorate. I think it is quite hard. , and he has a scholarship. If he meets his requirements, he can have 10,000 yuan a year. I like it recently.

09:44

You just mentioned that the seminars are mainly for nursing, and it seems that you may not have any similar seminars. Well, do your classmates know much about this thing? You just said that you have friends who are close to you, and you talked to them before you and you talked to them, did they know about it themselves? They don’t know much, and most people don’t actually know much about it. What way did they learn about it? It’s just hearsay. Everyone is hearsay. Basically, you seize the opportunity of hearsay and report it. this activity.

10:26 Right

.

10:29

Is there such a possibility that some students and some classmates don't know whether your class or your college has you? Suddenly I found my list, the name from the class list of that class suddenly disappeared, and then asked me what happened and what happened.

10:49

Then I told them that I was going to a junior high school, and they asked me what the junior high school was. I didn't know. In fact, there are quite a lot of students like this, right? I suddenly found out that you are gone and I don't know No. 5 Middle School. I know some individuals, but I don't know if other students are like this. Understood. Is there anything that makes you determined to plant a feeling? Has this ever happened? On the one hand, it was because I changed my major at that time, not because I failed the exam, but I was a little psychologically stimulated, and then I liked it and got stimulated, and then I worked harder to learn the main push, because in fact, the main push is this. The content of the class is different from the content of the major transfer exam. Then I spent a few days at that time, probably from early to late and then like this, so passing the group training is a condition for entering the middle school class, right?

11:50 Right

, sacrifice is wrong. Yes, so in fact, you have been firm since the beginning of your freshman year, and you must switch to prevention or work, er, prevention or clinical practice. I was not particularly determined at the time, because I felt that if I was very determined and failed in the end, it would actually be a big blow. What I thought at the time was to give it a try and leave no regrets for myself. If I didn’t succeed, I would It doesn't matter so.

12:17

At the beginning, you said that studying computer may not be suitable for you. Which aspect is not suitable for you? I think I may be interested, or it is very difficult to learn, or in which aspect I feel that I may not be able to compete with those who are purely engineering, such as those in the southeast of NTU, and those who come out with professional computer backgrounds may compete But they.

12:43

In fact, my grades in my former major in computer science were okay, but I thought I might not be able to compete with them, so I wanted to find another assistant for myself, but to compete with them means that it may be difficult to find a job in the future. Well, then you think that after you come to the clinic, or after you come to the prevention, you may have more advantages and greater advantages in finding a job than this kind of engineering major. You are right.

13:16

You just said that you would go, but you didn't say that you must go to a major to succeed. If you say that if I fail at that time, you can also accept to continue to study the major of the company, and you can also study competently. For me I may think it is not very difficult for you, I may put in more effort than before to learn it this way, because in fact, although I am not particularly interested in him, if I study hard, I will I believe that I can still learn by myself. What do you think you are most interested in? What subjects are you most interested in? I don't seem to be the most interested. Actually, I have access to medical and legal studies, such as medical and legal studies. Law studies are for law studies. Oh, you chose to study medicine at the beginning, so I would like to add that you were Did you apply for a major in law when you applied for the college entrance examination? I don't actually have it, because basically what I report is I, I, and I. There are two categories, one is engineering, the other is medical, that's all.

14:29

Actually, you said that you were very interested in law, why didn't you apply at that time? I was very interested, but in the end I didn’t report it after discussing it with my parents, and my parents wouldn’t let you report. It's not that I'm not allowed to report. The score is not enough. I think the law schools I went to are not very good if I go to the last, so there are quite a lot of schools that offer law, and I think there are audit universities.

14:52

But I don't think it's very good. I have to go to a better school, so in the end I still filled out a total of them. exactly. You were just about to check it out. What specific things did you choose to study medicine, which strengthened your determination to study, that is, when did you have the idea of studying medicine in high school or when you were a child? Because someone in my family got uremia, diabetes eventually turned into uremia, and then there were more kidney failures, like my father-in-law and my aunt basically all of them had kidneys that were not very good, and then I saw that some of them were not in good health. , Some have already passed away, and then I feel very uncomfortable. I especially want to do my best to save him. Even if there is no way to save him by myself, knowing some relevant knowledge can help the family to maintain better health. I think Also good.

16:03

Do you have any experience with this matter? Are you a sophomore now? Have you participated in Daiso or Challenge Cup or something? No, not yet. Have you ever been in contact with research or something? Guozhong just didn't have it before he was in the middle school class now. I have never been in contact with scientific research. Yes, you know that the shareholder class is mainly engaged in scientific research after entering the activity class. I know you have an understanding of what scientific research is for? Now, because the middle school classes are all rotated in the laboratory, basically I have rotated three laboratories at present. I know what the laboratory is about to do, and I have a better understanding. After entering, I have deepened some understanding of the supplements.

16:59

Did you meet your expectations? Do you have? After entering the regular class, is it similar to what you originally thought? I feel that scientific research is a little more boring than I thought, but I think that since this is the work I want to do in the future, I still have to take the initiative to love it. Although it is a bit boring, I still go to the laboratory more like this. . Is there anything that disappoints you in the middle school class? What disappoints you more in the middle school class is the point that disappoints you. For example, it was more boring than you imagined , and it can be considered whether or not anything specific happened.

17:45

, the junior high school class was held once. All the junior high school classes were all students from the junior high school class on 17, 18, 19. They had a meeting together, and then they invited people from the academic affairs classroom, and then came to tell us about the specific future of the junior high school class. What kind of qualification is the insurance research, and then they said that if you meet a certain requirement, you can be guaranteed the research, and then they reiterated that if you reach the top 40% of the prevention major, you will be guaranteed. A qualification for research, but it does not necessarily guarantee the success of research, and everyone was very angry at the time because of this matter. Later, I also had some communication with the school, and then I felt a little disappointed at the time anyway, but after thinking about it, I felt that I should be able to achieve it with hard work, so I was not so disappointed.

18:43

I don't quite understand what it means to be 40% likely to be successful in the research, and I am a person who requires 40% to score in 1000% and prevention or basics. I don't know the basics, so I just think In prevention, if you rank in the top 40% of the prevention major, that is, if you are compared with all the general prevention majors, if you rank in the 40%, you will be eligible for a postgraduate study, but there are only so many places for him to post a postgraduate study. If the quota allocated to the junior high school class cannot reach as many people in your junior high school class, your junior high school class may have to compete internally, but we originally thought that you only need to reach 40% of the junior high school class. All can be guaranteed research, but in the end, it is found that internal competition may not be avoided.

19:23

look like. There were some changes later. Are you afraid of involution? right. Wasn't it a comparison of results for internal competition at that time? For example, if you are 40% in the guaranteed research, you may be able to guarantee two if you exceed the quota for the guaranteed research. If you have three of you in the top 40%, how did you choose two people from these three people? Didn't he say? ? I didn't say that it should be based on grade points, and then look at the results of scientific research. But the reason why everyone had a relatively large response at that time was because the documents he came in said that if he reached the top 40%, he could guarantee the research, but he did not emphasize that if the number of places was not enough, internal competition was needed.

20:12

Originally, we thought it was the people from the Guangzhou class who worked hard to study, and then it was not necessary to avoid that volume. Did you notice this clause before signing up? That is, the top 40% can be directly guaranteed research. I noticed it at the time, but I didn't expect it to be so detailed, maybe only the front layer. In fact, what attracted you the most at the time was that you said at the beginning that you could get a master's degree and a doctorate. For you, you still want to study for a doctorate, and for me, I want to continue reading, so this is the reason why I want to study for a doctorate. what is it?

20:42

Because I think everyone is studying for a Ph.D., and it is very important to improve their academic qualifications. I think it is necessary to study medicine. You said that his undergraduate degree is definitely not enough, so he needs to study, so the Ph.D. is still a bit of a follower Mindset is gambling.

21:01

I think there is a point, but it is inevitable. After all, everyone is now thinking about where to go and then continuing to study. What is the purpose of pursuing a doctorate? Is it your career plan or interest? I may feel that it is more of a career consideration, considering the factors of employment, so I want to study for a doctorate.

21:25

So apart from this decision at that time, did you discuss the policy of postgraduate research with your classmates in the junior high school? Did any of them say they wanted to quit? Some people have said that they just want to quit, some have considered this issue, but they have not quit in the end, and they may also be considering it. In fact, for ordinary preventive medicine professionals, the chances of the activity class being guaranteed will be greater. Can you say that? Yes, but it seems to be a middle school class, because in the end, he was guaranteed to Nan Medical University. For those students who have excellent grades and want to go to the next level, yes, if they quit, the way out will be even better. it is good.

22:14

He thinks that there are some students who may be better. You go into the middle class, and now it's rotating, right? Yes, it is possible to rotate 4 departments and 4 laboratories. Yes, but I have a round of words that the first and second rounds are all in the same teacher's laboratory, so I have this idea. Because it was a month before the exam, I didn't really want to go again and I could take the time to integrate into a new city, so I stayed in it. Are exams stressful? There is a lot of pressure on medical students. Do you feel more anxious when you are stressed? I? Exams are very anxious, I think.

23:08

Pressure I would like to ask, do you have procrastination or something? Or say that the time is about to come, and then hurry up, for example, a certain project, a group report called a report, etc., it is almost time, and then hurry up and do it. I don't procrastinate at school. I am at home and sometimes what I say in my life. If I don't have to go to school at home during the winter and summer vacations, I will procrastinate a lot, and then I usually don't procrastinate at school.

23:44

Because the more things you have, the more afraid you will be. You try very hard to learn a certain course or review a certain homework, but in the end you fail again. You will be more afraid of this happening, or I In other words, this is a bit of what I just said is not very good. For example, if you try very hard to do a certain experiment, but you fail in the end, are you more afraid of this happening?

24:11

Is this a situation that makes you feel more depressed or that he is more likely to fail before you start making you feel more anxious?

24:25

Why do I feel like he's going to fail before I even start? When you are predicting, when you are doing something, you will definitely think about whether the experiment will be successful, and you will consider this question. I should think about it, but I am more afraid of failure, but when I do it, I should not think that he will fail. Do I definitely hope that he will succeed? I might not be as pessimistic as I thought at first. But in fact, starting from your personality, you are still more afraid of failure. Yes, I am definitely more afraid of failure.

25:04

Apart from the pressure of exams in school, are there other pressures? Exam pressure, and how to say scientific research is also a kind of pressure, because I see that some students around me have already joined the project, the research group, and then they are following up on the project, and then some tutors may be more sad for them A little bit, and then give them some tasks to do as usual, and then your tutor didn't make you feel that because one of ours is quite special, there is a tutor who asked to talk about students from all over the country from the beginning, and then he has been studying at the undergraduate level. If you want to follow me, you can't change tutors, and then let them join the research group to follow the project from the beginning.

25:47

Then, like we have been rotating, it is because the rotation will be done once in half a semester, so the tutor will not be particularly sad for you, because you will not necessarily stay with him in the end.

25:55

So maybe it's not that deep, it's just not right for a personal teacher.

25:59

Are you sad? It's not that I don't care, this is a common phenomenon. It's because you just take a turn with him and won't stay with him, so the teacher will definitely not treat you particularly, and you will feel that you have learned something, learned some basic experimental operations, and then read some papers. Who taught you? Did your brother teach you, or did you teach yourself? I usually watch experiments like this with my brothers and sisters in the laboratory, but there are not many opportunities to do experiments by myself. Do you think learning these things will help you a lot? For my junior year, because the junior high school junior has a competition within the junior high school class, it seems that he will form a team with each other, and then complete a subject. There are some foundations for these basic experimental operations. helpful, I think.

26:50

Lab You are the first two are in one lab right? Do you like that vibe? human environment or something. I think I actually regret the first two laboratories now. I think I chose the wrong one, because the instructor of that laboratory asked me to read the literature at the beginning, and then asked me to do less experimental operations and start with the literature. But my personal plan is actually to learn some experimental operations first, and then read the literature after learning these basic operations.

27:18

Then the sequence of this is that if he asked me to read the literature at the beginning, I actually asked my brother to teach me the experimental operation, so I felt that it was not right to have a choice, maybe it was because of me. At that time, there was little understanding of what was done before the election. Have you ever had a chat with your teacher, just about your own thoughts? There is also less chat, because the teacher will regularly send me some documents for me to read, and then in the end he will write a report on the task he gave me to organize the documents for him. You haven't talked to him. In fact, I want to learn more about the second teacher, first learn the operation, and read the literature. Haven't talked. So this will make you feel that your sense of gain may not be as high. you can say it this way.

28:12

You can tell us that you started from college. It should be said that you are most proud of the one thing you are most proud of, or that you have done something that makes you feel very fulfilled. Is there anything? Does it have to be related to the middle school class? Need not. Life can also be, feelings can also be. I think the student union can be the most impressive. do not have it? I was once the person in charge of the Mid-Autumn Festival Gala of the Orthodox Academy, and then successfully planned a Mid-Autumn Festival Gala, which made me quite proud.

28:58 When I

was a freshman, when I was still a sophomore, when my sophomore year just started. Master planned. Yes, including arrangements, and equipment and so on, I am in charge of the Mid-Autumn Festival of the Orthodox Academy, yes. Because he was with our department, I originally thought how could you go to the Orthodox Church to help the Orthodox Academy hold the central party, because he and our department wanted to pick up the equipment, and then connected with us.

29:27

I am the office of the Youth League Committee of the school, and I am in charge of some equipment such as lighting and sound. You have been involved in some organizations like the Youth League Committee of the Student Union since the beginning of your freshman year, right? Do you think it's a big gain? before? I feel that I have gained a lot from being in a group. The most rewarding thing is that I have met many students from other majors. They gave me some study materials, which were very helpful to my study. Is there any other work in study? Just have a good relationship with them, and sometimes go out to play together and so on, as well as in life.

30:04

Can you tell us, you have joined several organizations now, one is the student union of the Youth League Committee and others? In fact, I have basically withdrawn from the previous participation, and I was still in the hospital when I was a freshman. I served on the Youth League Committee and became the Minister of the Science and Technology Innovation Department at the time, and then I changed majors because I quit. Then now, the club will join the school and college in the freshman year, yes. No society? There are clubs, but they are more complicated, just some badminton clubs, then skateboard clubs, computer design, and then they all retired, right? I didn’t quit, but I took part in activities relatively few. Now, I mainly participate in the activities of the skateboarding club. Sometimes I relax and go skateboarding on weekends.

30:55

So you said at the time that the major clubs in the freshman year have withdrawn. In fact, what I'm talking about is that the freshman organization retired from the hospital, or it was because of changing majors, so it was retired. If the transfer was not successful at that time, I would still continue to do it in it, yes. Because after all, I had just run for minister at the time, um, then because I quit, so the minister was replaced by just one person, yes.

31:26

You said that the Mid-Autumn Festival Gala is one of the things that makes you feel the most fulfilling. What is the one thing he is most proud of? Can you tell us more about it. Because before, I always listened to other heads of this department, and then said what should be done, and then listened to the instructions to do things myself, and then that time, I gave instructions to others, and then asked them to complete, and then included I have to be responsible for the overall planning of the entire venue. I feel very proud of this event, because it is the first time I have tried to coordinate these tasks by myself.

32:03

Haven't you done these coordinating work in the Youth League Committee? I've always been there to help pick up equipment for a job. That was my first time coordinating, so I can say that pride comes from doing something you haven't done before. Is this description accurate? Not particularly accurate. Where do you think your pride comes from? It comes from, er, completing something that I think is more difficult than what I have done before, and then it is done better. You may have just started, and the task may not be able to be completed. I think it is a challenge for me, and the final successful completion is the point that makes you most proud.

33:06

I would like to ask now, what do you think is the most important thing to you in the past few years in college? Whether it is to complete your studies, or to say, for example, that the Youth League Committee is doing things like the vice chairman and chairman, or what achievements have you made in scientific research? What do you think is the most important thing to you? Have you thought about this question? I thought about it, but I think the first thing is the Youth League Committee. I may not plan to continue the election, because our middle class is very busy. However, I hope to achieve a balance between studies and scientific research, that is, if my studies do not affect the ranking, er, scientific research can also be done as well as possible, because I think there is no way to balance the importance of these two.

33:55

So you think the Youth League Committee is actually interfering with your studies. Yes, he's actually quite busy there. Are you planning to quit next year? Yes, it should be the third year of retirement, but you just said that one of the things you are most proud of is the Mid-Autumn Festival party. Have you ever thought that maybe in the Youth League Committee, you can have more access to these relatively large conference organizations or activities? organize? Maybe after I quit in the future, I won't be able to get in touch with this kind of high-challenge, high-challenge and high-difficulty thing.

34:35

I thought about this, but first of all I think I have always put academic and scientific research in a higher position, that is, when I have to give up one when I am very busy , I can only give up the change of term at the end of my freshman year. Everyone will have the kind of change in charge. For the chairman of the Youth League Committee of the school, there was an election campaign at that time, but because the preparation was not sufficient, there was actually no election. In this way, I actually wanted to continue to run for election at the time, but after I successfully entered the junior high school, I wanted to focus on my studies, yes, so I gradually let go of these things. In fact, I am not too busy with things over there. .

35:32

Is there anything in the middle school class that makes you feel more stressed?

35:37

Is there anything in scientific research? There are scientific research, because he does not require us to rotate every week, but at least requires us to go to the laboratory to rotate three times, and then doing experiments usually takes a long time, and then there are a lot of normal academic courses, which take up some of the review after class. Some time in the course, and then it actually put a lot of pressure on me.

35:59

Research takes up too much time for theoretical study . right. What is the biggest feeling you have given you since you started middle school? Or if it is more difficult to answer, you can tell us, what do you think is the biggest gain after entering the Chinese class in the second year of sophomore year? If I don't think so, and you don't think there is any difference, you can say it. I think there is a difference, that is, I think the people I know now may be more capable of learning than the classmates I knew before, and then I can learn from them, that is, they can drive me better.

36:48

Because in fact, I think some of the students in the previous class may not be particularly concerned about learning, and the level of grades including the overall class is not as high as it is now. Now they are all working hard and comparing papers, and then you think Does the original environment affect your motivation to study? Because people around you don't study very much, maybe you and you may not have such a strong motivation to study.

37:20

Yes Yes Yes, may I say that? Not too accurate either. Usually, in my previous class, he actually studied, and there were some people, including some boys in particular. In fact, his grades were not very good, and his desire to learn was not particularly strong, but some girls around me were actually more diligent. , but because they are not particularly good at their class level and grades. Then it wasn't particularly difficult to maintain a rank at the time, which made me less motivated to learn it.

37:55

So what you mean is that you have entered a new class. In fact, the students around the class may be better. Your environment will drive you to study harder. You can't relax your current words, yes.

38:11

They say that your classmates can lead you. In fact, the main reason is that what they bring to you is to study the possible study ranking. If you use the energy distribution of the previous study, it may be new now. The class may put me at the back, and this pressure will drive you to study, so they drive you to drive you in this aspect.

38:40

I just want to ask a question, I am still a hypothetical question, the class is the first in the class test and learning a new skill, which one thing will make you more excited? What kind of skills? For example, a certain scientific research method, an experimental method, or a theoretical research method for writing an article that you have always wanted to learn, the experimental method can all be something you want to learn. A skill you've always wanted to learn, which one thing excites you more?

39:19

For example, you want to learn to do the same experiment of so-and-so. In fact, that time is quite difficult, and then you finally learn and review with you for a long time, and finally you get the first place in the class. What do you think will make you Are you more excited? It may be that the first class in the class will let you update, yes, but I am not. I don't have such high requirements on myself, and I hope that my class is low, but if I get the first class in the exam, I will definitely be more excited.

39:58

So I think you are a very, very successful person. Have you ever thought about yourself, you are also rotating scientific research, have you ever thought about your interest in reproduction? Very novel. Do you think you are interested in him? till this moment? I'm still very interested so far, because I think it's quite meaningful, even if I say it. Because a lot of it is the kind of infertility problem now, and if I can help these people in the future, it feels very meaningful.

40:57

Have you looked into any other aspects of prevention? Yes, for example, they may be engaged in disease control and customs in the future. If you understand these things, you will feel that you will be more interested in things such as disease control, especially after the outbreak. I may still be more interested in the appreciation of Guozhong in this regard.

41:27

Well, because Bozhou also offered us a lot of elective courses, we might have a better understanding of this aspect and be more interested. Would you say it would be better to know better? Or to put it another way, because you have learned a lot about the basics of promotion, so alas, you have the basis for learning, so you will want to engage in this thing more. Is there such a factor? In There. There are electives you mentioned. Because after he went to middle school, some changes have taken place in his curriculum. It seems that some courses have been deleted, right? What are your thoughts on this? I think he also added courses when he promoted the course, and I think it is understandable, because after all, if we just add and not delete, then you will think that some courses should not be deleted.

42:32

I think Shanke, do you understand those deleted lessons? I know, in the last semester, some of them actually changed the order. For example, some of their preventions were the syllabus of last semester. This is the syllabus of this semester. But this semester, they have a parasite, and we don't have one. Learning parasites, but we have an additional reproductive biology, I don't think Shenzhen has much feeling anyway. After all, learning is also very important, and learning so much is very tiring.

43:06 For

those new classes, how have you been feeling since the class? Do you feel like it helped you a lot? I don't know how to deal with the exam, because it actually gave us a book on physiology and biology, and that book is very thick, but I feel that the teacher in the middle school has little relationship with the book when preparing lessons, just I feel It seems to be based on PPT again, but I feel that the things he talks about in PPT are different from other courses. The comparison is just the feeling that you can't find the knowledge points, and you don't know how to deal with the exam. .

43:47

Mainly because the exam is difficult. If you are not comfortable with the exam, do you feel that you have learned something? Yes, he introduced some more cutting-edge technical knowledge and so on. He thinks it will help you in which way, is it for your future employment or the direction you are interested in? For example, he should have inspired us to think about scientific research first, that is, he gave you some new ideas, and seeing those new technologies will help you develop your thinking.

44:21

You just said that after you came to do scientific research, you imagine that scientific research will be boring. From the time I talked to you, I also felt that you are still a classmate who likes to communicate with others. Can you say that? Yes, what do you think? This boring may not actually conflict with your personality.

44:40

I think there will be, because they sometimes do an experiment in the laboratory, which may take several hours, and then the space is relatively small, so I feel a little more pressure.

44:51

Have you ever thought about this? Because you just said that you want to study for a PhD, right? In the future, I will study for a master's degree. In fact, there is still a lot of time to study for a doctorate. Have you ever thought that this may be the case for a long time in the future, how to adjust it when you stay in the laboratory, have you thought about this problem ? Regulating your repressed emotions, it may be a little bit conflicting with your personality when doing experiments. Have you ever thought about the problem?

45:17

I thought about it, but at the time I thought it might be because I still have more courses to study at the undergraduate level, and then experimenting is not my job, and then there are some other pressures on it. After I impose it, I will feel more depressed , but if it comes to my graduate school, I may do experiments after my job, and I will not have so many other pressures to interfere with me, maybe not so much now Depression, part of my pressure may still come from the usual heavy schoolwork and spending a lot of time in the laboratory, which brings me invisible pressure.

45:54

So it means that you feel that you are in a state of learning, and you may not have so much pressure to study after doing experiments in school.

46:06

Do you have any? 11. I have two questions. One question is that in your current state, if you don’t do a good job in scientific research, it won’t have any impact on you. Will it have any impact? For example, you may have been working on it for a long time and have not published an article. Will it have any impact on your future studies?

46:29 It

shouldn't matter if you go to college, because after the policy reform, you won't say that because you have done a great job in scientific research and your grades are not up to standard, you will be directly guaranteed for research, and now it is still based on grade points, so I tell you The pressure of scientific research is what you put on yourself, right? You have a motivation to go in, and you want to put pressure on yourself for scientific research, and you want to learn more about scientific research methods and the like.

46:58

Can that be said? Part of it is my own pressure, and part of it is because of the students around me who are also working hard in scientific research, and then I also see it in my eyes, and this environment actually has an impact on me.

47:12

The second question I want to ask, after you said it, you will be at the master and doctoral stage. Maybe there will not be so many classes in class, and maybe the pressure of studying will not be so great, but now I am asking a hypothetical question, such as After you do a certain experiment, you enter a bottleneck period. You may need to study and read literature to solve the bottleneck. In fact, in a sense, it is equivalent to a theoretical study at this stage. During a process, have you ever thought that it is like encountering a bottleneck in the laboratory, and then going to learn theory by yourself, which will actually affect the progress of your entire experiment. Will this also be a kind of pressure?

47:57 counts

as well, but compared to what I am now, because we still have assumptions that you have thought about me before? I haven't thought about this before, what did you think about it just now? In the future, although it is said that when we encounter these difficulties when doing experiments, we may go to the literature, but now we not only need to read the literature, and then read the experiments, we usually have to go to class, and then we have to complete the homework after class, so the pressure may be more.

48:27

When I look at the literature, I think uh, because I know that I will definitely read the literature when doing experiments in the future. I already have a mental preparation, so I may not change it again. I want to ask if it is possible. Is my idea right? Will your pressure come from hard requirements, such as exams, such as homework, these things you have to do.

48:50 In

this way, maybe after you have done the experiment, you can study by yourself, and you can read the literature. In fact, it is the same whether you read it today or not tomorrow. For you, it is just that your experimental cycle becomes more and more important. only long.

49:02

But no one is asking you to write this theoretical review today. It's pretty accurate, I think, right? Mmmmmmm. The next question is um, you're in your sophomore year now, right? In fact, it might be soon. It's not too soon, and the jelly class may not start preaching until next month.

49:34 Are

there any schoolmates coming to ask you this question now? There are two things about the middle school class. What did he ask before? He asked about the requirements for entering the country as a junior high school student. In terms of grades, and how the junior high school class was, he would tell them about the main points. This The question is so hard to answer, how do you answer it? I'd like to tell them that you are an official introduction.

50:02 Let

’s talk about the more official introduction. It’s not too personal, because I think everyone feels different, so I can’t mislead them too much. Oh you're afraid of misleading them which aspect are you afraid of? They are misled by your information. If I think the junior high school class is very good, after I tell them, after they come in, if they feel bad, they may blame me for misleading them.

50:23

That's it, would you recommend them more in this class? I recommend it more, because I actually asked all the direct students from my previous major. I also hoped that there were more of them how they knew about it before. They found out through me, because they knew that a senior classmate was transferred from the previous class. Did you transfer from the 18th or 17th level before you? One was transferred at level 18, and then another was transferred to me at level 19. There were only two people in total. We asked you about the transfer student at level 18, and I asked him. I just had an in-depth exchange with him. What specific questions do I have? I asked him all the questions, and he answered all of my questions.

51:08

What do you remember when you asked him the most? I remember that because I was particularly impressed at the time, it was after the end of the major transfer exam, and then I heard that there would be a junior high school enrollment soon, a written test notification for the junior high school, and then the written test notification The result came out? ? It came out, oh, it came out, and then I didn't have it at that time, and then I started to think about it, but the notification was delayed, about 10 days later, and then I was very anxious during that period, while I was in I was thinking about whether he would stop recruiting this year, and then I kept asking the senior sister what was going on. When I asked the senior sister, he reassured me that it would not be so fast, and it might take a while before he came out.

51:51

So you were very frightened at the time, and he simply refused.

51:56

If you don't recruit, your anxiety comes from the possibility of continuing to study engineering. My anxiety at the time came from maybe ten days, because school was about to start, I couldn't play well at this time, why should I study hard here? Then, if it is said that he is not recruited, you have learned it for nothing, is it true? clear. Is it or can be said that it is a freshman year during the high school class? That is to say, what was the most impressive thing for you during the middle school class? Have it? It can be said? One piece? Not even if it seems. Nothing particularly impressive.

52:41 After

my middle class and you entered the engineering class, do you think there is any special difference from the original prevention? There is nothing particularly different, because in fact, most of the classes are still with classmates, but maybe after the third year, when everyone is going to be separated, so far there is no difference. I also wanted to ask the question you asked my senior at that time. You kept asking him why he hadn't started to sign up, and he didn't ask him anything else, so I asked.

53:18

But you just asked me if I was the most impressed, and then I said and I asked him, what was his ranking at that time? Is the exam difficult, what types of questions are on the exam, and then do you say the entrance exam? The entrance exam for middle school, the final exam for junior high school, and the written test for middle school are the selection test. Do you want to do a PhD in the future? you? I think I have asked this question in the doctoral class. Has the study in the middle class achieved your expected results?

54:08

I asked, I remembered, have you ever been abroad before? should not. I didn't say level 19, because some people just came in, and then I reminded him, but I participated in an online exchange last time, that is, if it was an epidemic or not, he should go abroad to exchange, but the last time was because of the epidemic , and then there is only one online learning.

54:34

What to learn? Whether it is a middle school class or not, it is organized by the Orthodox Academy where the school applied. What are you learning? What I reported at the time was the housing prevention and control policy for the new official epidemic. Anyway, it has something to do with public health. Who should I communicate with? Just like the University of Wisconsin invited a professor to teach us, through what channel did you learn that you can participate in the on-site counselor posting group, and then sign up for the publicity? right. Do you think that communication is the gain? I think he was not a prevention, and then a public health major, but I forgot about the other major.

55:31

Then what he said at that time actually had little to do with public health. Most of the time he invited some of his friends to introduce some characteristics of China and the United States. Also, I was particularly impressed that he once asked us to introduce his own Red Square, but in fact, it was more about his English ability to exercise, and then he felt that there was not much gain in professional knowledge. Any other hobbies? hobby. You have to say everything, you can say everything, you don't have to learn. Run, skateboard, and catch a show. I was just about to ask why when you were in contact with a research supervisor, you were in contact with a research supervisor, right? Rotation last semester.

56:35

I am now on the third and already on the third. So you are equivalent to having three tutors in one academic year, because you have one tutor who has transferred twice in a row, one transfer and one more transfer. It's like this, when you choose a mentor from three teachers later, will you choose from these three mentors, or from other mentors? I should choose from these three tutors, because the requirement of the junior high school in the three countries is that you should try to be as stable as possible, and be stable in a laboratory. Then if you haven't been to that teacher's lab, that teacher won't necessarily accept you because he doesn't know you at all.

57:11

Have you thought about who to choose now? Based on what criteria, I have thought about who to choose, but it has not been determined yet, because in fact, the third round of rounds has only just started this week. I have not met the tutors of the third round, and I am more inclined to the second round. Lun's mentor, because the brothers and sisters in the laboratory are good, they often take me to do more experiments, and then also teach me how to do literature reports, make PPT, and then read some skills in literature, and also borrow The book was shown to me, and I felt very warm, so I liked the atmosphere of the laboratory, and I am more inclined to the second one, but the final confirmation may not be confirmed until I finish the third round of rotation.

57:52

Do you think about the direction of their research when you choose a mentor? In fact, the comparison is relatively small, because I feel that there is no direction that is particularly attractive to me, and there is no direction that I particularly hate. So my current selection principle is which laboratory atmosphere I think I like better.

58:11

You said that their seniors and seniors will lend you books. What kind of books do you think they are borrowing? Molecular biology borrowed such a thick book, should molecular biology be your next course? Or why build this book? It was recommended by my sister that I need this knowledge to do experiments in the future. He thinks this knowledge is relatively basic, and then I start with knowledge first. Molecular biology should not be a course that needs to be learned. We have already finished our second year of school. But I don't think there's enough in the textbook, there's not enough stuff, there's a lot of introduction, for example, some basic technologies such as gene knockout are in the book he lent you.

59:05 Right

.

59:07

But this thing may not be mentioned in the textbook, which is more about gene knockout than the test-taking, and then there are more generalized things outside the classroom, that is, the more operational things are explained in more detail. So so far, when you are rotating, you should have done the experiment without saying that. Have you ever done it, but it is relatively rare, just talking about some basic operations. You have not carried out a project independently, not yet.

59:55

Do you have to protect the capital in a class like your Ph.D. program? right. Did he ask you to do this? Doesn't the postgraduate study mean that if you get a postgraduate place, you can apply to other schools on your own? No, the middle school class can only apply to the Southern Medical University, and then the direction of reproductive medicine. Will there be other consequences if I apply to another school? I haven't tried this, because I haven't graduated yet, so I don't know, so many people who think they don't want to continue to study in this direction have quit.

01:00:27

This is also considered a restriction, after all, he has a discount for you. Do you mean your classmates when you quit? Or your top, above, we haven't refunded this session yet. Why didn't we refund the points in the group hall before? What do you want to study again? Did your senior and senior sister tell you this news before you were, or did it tell you this year or the second half of last year? At that time, there was a meeting about a month after entering the middle school, and then I invited, er, many school leaders came to listen.

01:01:06

So after your senior and senior sister came in, did senior and senior sister tell you that they had heard the news before? That is, the top 40% may not necessarily be able to direct. No one mentioned it at that time, but there was a row of seniors and seniors sitting at the back of the meeting, and they said um, it was the first time they heard the news, not that they seemed to say that this was different from what they heard last time. It's the same, and then what you say has changed again, maybe it has changed before, and then it's changing again.

01:01:33

Then last time, we asked the vice-principal who was hired at the time and couldn't remember, and asked him to make a promise. Then the vice-principal was on a business trip out of town, and then he flew back specially, and then gave it to our middle school class. The classmates made a promise that the rules are like this.

01:01:52

should not change the promise. What did he promise? But there is no promise that the top 40% have the qualifications for the postgraduate research, but not necessarily the postgraduate research, but he will try his best to get a place for us, because he is not sure. Get that right. He didn't have a paper copy, but many students should have recorded it at that time. I think he said this, but he didn't limit himself to death. Even if you entered the top 40%, he could still let you not be guaranteed.

01:02:24

Those words he said. Yes, he did not say that if you enter the front, you will definitely be able to graduate. So the recording was useless, and he actually left a little space for himself.

01:02:40

So, can I actually say that the classmates around you still attach great importance to the qualifications for postgraduate studies? Do you think there are many classmates around you who want to study for a Ph.D., or do they just want to study? More than a graduate student? I haven't discussed it with them in detail, but at least I know that everyone definitely wants to go to graduate school.

01:03:06

Do you think your Ph.D. is big? For example, let me put it another way. Maybe this is 5+1+3. When you reach the sixth year, you may assume that your defense has failed, and then you may have to study for another three-year master's degree, plus three years for a doctoral degree. Will you continue to choose to read this way? Or is it the end of a three-year master's degree? Haven't thought about it yet. Why do you have to study for a master's degree for another three years after you fail? It was originally 5+1+3, and you successfully defended in the sixth year. Did you directly enter the doctoral level? If it is said that the defense fails, it may be postponed to the 7th year or to the 8th year and still at the master level.

01:03:43

In fact, in this case, you and those who were admitted to the postgraduate entrance examination, or other postgraduate students who were guaranteed admission, aren't the time durations of ordinary graduate students and theirs not the same? Will you continue to gamble again? If the time is too long, I may not read it, but if the time is year by year, it is acceptable. I think because because because because it took many years to read, what I actually mean, maybe I can't express it clearly, not very Clearly.

01:04:16

For example, when you finish your fifth year, and you reach a later graduate student, your master's degree is extended for another two years. In fact, when you are in your eighth year, you have a choice to continue your Ph.D. Or go to work, because for you, the length of schooling is the same for you, and there is no difference. Would you be more inclined to choose to study for a doctorate or go to work? When I graduated from graduate school, I didn't seem to have thought about this problem.

01:04:50

? If you are a teacher or an experimenter in a university, you may still try everything. The reproductive center may be my ideal plan at the moment. After entering the junior high school, there will be changes. When it comes to your career plan, it is because there was a classmate in our dormitory who paid special attention to what the national exam was called. What else is born? What is called the selection of students. That's the kind of thing, and then your roommate to roommate, and then she introduced it to our dormitory, and everyone thought it was pretty good, and then they all listed it as a path that they might want to do in the future. Direction, and then when I was studying engineering, I also thought that I might go to some big companies like Alibaba and Tencent in the future. In fact, the direction has changed a lot. At present, I want to go to the reproductive center. When you were a freshman, you also thought about going to the public examination, and you also thought that you don’t have this idea at all now, or it’s not exclusive. , but not your primary choice.

01:06:25 Right

.

01:06:26

Because I still want to finish my master's and doctoral studies, or I put it another way, have you graduated?

01:06:35

After graduating from an undergraduate degree, you passed the job entrance examination, and then you were also guaranteed a postgraduate study. Which path would you choose? It's so hard to write. I may not for the time being and I will continue to study, I feel. reason. Why? Because he can't take the exam every time he graduates, but I still want to improve my education, because I think that if you take the civil service exam now, you may find that your education is not enough after you work, and you have to continue to study, so in fact, in the end, it is still for employment services. , Is it a better opportunity to improve your education, yes. There is no other. I think you should follow me here, you can wait a while.
