# Supplementary material for: Why do you choose this program?—A decision-making model of medical students based on grounded theory
Source: PLoS One. 2023 Sep 15;18(9):e0291634. doi: 10.1371/journal.pone.0291634 (PMC10503722; doi:10.1371/journal.pone.0291634)
Supplement: S1 File — (ZIP) [file pone.0291634.s001.zip › RAW DATA/P7 CHINESE.docx]

00:00

我先想一下我们访谈的目的啊主要就是还原你从活动班的宣传到报名录取到最后的学习你现在是在读的学生吗？不是。他不是那个他是那个条件不是，对，我就想问一下你是什么类型，我是那种没报符合条件的没报的。你是哪个专业的？我是护理专业的好的。我就是想还原一下就是大一整个过程，所以从重要的事情或者你的情绪，让你印象比较深的点，你全部都可以说可以好吧？

00:37

然后在之前我要先说一下声明一下时间道德伦理须知。

00:41

本次访谈中受访者是在平等自愿的原则上参与的，受访者必须真实表达自我想法和认知，确认自己符合受访条件。访谈的过程会被录音、录音资料、将以匿名的形式用于科研，不会泄露给任何第三方。在访谈过程中及访谈结束后，受访者有权取消研究人员的录音资料使用权，你是否是小平同意同意你？一一几级的不是19级的老师。19级护理对。所以就是在去年的大概这个时候，对去年的时候，你就先说一下你对活动办的了解有哪些以及怎么了解的吧。

01:19

对于各种办法就是因为作为男生这边做一开始考进护理专业就有点不好意思了，想转专业之类的，然后也了解到我们班也是一定一个途径。然后后来听说工作班之后，然后去了解相关情况。然后后期反正以后就和同学一起商量一下关于工程班什么报名之类的。后来感觉活动班太多的不确定性，因为我们旁边身边没有这没有这方面的人，所以还是稳妥期间就没报没报名。

02:03

你在沟通的时候去是是自己选的护理专业吗？不是这个调剂的大部分都是调剂的。你开始选的是什么？我一开始选的，我一开始我也没多想，我报的是引进，因为分数不太高。你在高中时候报名的时候是是一定要报医医学类的吗？还是医学里你是文科生还是理科生？我是理科生，我学物化的。你当时是工科理科都报了，还是说就报了理科类？都报了，但是我还是偏向于医科类。

02:41

你当时应该是6是6个学校吗？你报高考的时候好像对应该是有几个医科的学校我报了我们因为我徐州人我报了我们南京医科大和徐州医科大，其他的应该都是工科的，对理工科的。你你当时在高考报名志愿的时候有跟你家里商量，还是商量，这是我们一起商量的，结果感觉医学方面还是挺不错，然后就报了大致方向他们。

03:14

有对你有什么影响吗？影响还挺大的，一开始他们想当时然后想我是报想报医学和理工科之类的他他，然后我爸妈更希望报那些示范类的，然后但是然后所以那6个志愿里我也填了1个是他的1个所以其实其实我可不可以说，其实你自己本身并不是很想上师范类，我不喜欢上师范类，父母的意见你也会接受，只是你不喜欢的。

03:51

他但是我不知道允许方面的话，我爸妈也是很同意的。我爸妈希望如果是没有被医学这些录取的话，到那些师范类的也行。

04:06

对，所以报志愿这方面还是没有很多矛盾的。你再报影像技术，后来被调剂到护理学，你觉得你说你上护理学不太好意思，是指哪方面不太好意思？感觉一开始对。高中时候对然后是这方面还是比较排斥，因为刚刚高考完之后就拿到这个通知书，就感觉还是对于男性这方面还是太不太不太舒服的，后来了解之后我发现还是不错的。

04:40

那是你我大一之后还是有转专业机会吗？当然是希望呃有更好的发展去冲一下别的专业。但是现在对护理了解还是没有什么排斥，还是感觉挺不错的。你说呃一开始对男护士不是很了解，对吧？后来觉得很不错是不错，是指哪里不错，就是指以后就业方面没有。就业方面。就业不错，对。这个方面。你一开始进入护理学专业以后，你父母对有什么反应？我父母一开始也是感觉作为一个男生，学护理方面还是有一些难以骑车，不好意思。

05:22

但是后来通过我我的了解，然后我和他们一起讲之类的一起沟通，他们对护理长也是挺印象也是挺改观了。也不是排斥他，是跟他讲了什么他们才改观的。讲什么以后的发展以后的就业方向就是好好就业，没有什么以后心思在于还有就业机会很大的。

05:48

我可不可以说你父母其实挺在意我的就业问题，因为他们一直让你上师范类的，还有医科类的，他们这些都是就业比较稳定的学科，他们以后希望有一个稳定的，他们更在意你能不能找到一个稳定的工作，可不可以这样说啊对？

06:10

你当时转专业没有申请是吗？当时转专业申请的时候申请的是医学影像学，但是没有转上。我想问一下，你加深对护理专业的认知，认知渠道大概是听老师讲的还是就是对？我们一大一课程都有很多的那种见习，还有和那些临床的老师一些沟通交流，他们就是我们通过和他的交流，还是认识到了以后的专业以后的就业，就业方面是东西感觉还是可以的。

06:48

可以说一下你在见习或者跟老师沟通的时候，让你印象比较深的事情，让你触动比较大的事情在有一次见习在应该在去海楼。51514教室，然后有一个是南南京市附属第一医院的老师，然后他也是一个护士护士，然后就和我们讲一些他讲他讲讲一些他的经历。他作为故事以后，工作之后也是很累，一方是一方面，然后他们也是有很多也是有假期的，也是，而且工资也是挺不错的，然后就还对他们也就说最重要一点就是好找工作以后工作不用愁之类的。

07:42

你当时这一次的谈话让你印象比较深刻，你有跟你的同学有一起讨论，你应该是我们同学一起上的。我们周围之之间有讨论过，你说这周围就是我高中朋友之类的是吧？校友他们有什么触动跟你一样大吗？还是？对他们一一开始大概我们一一开始9个男生，所有人对护理专业都是应该都是都是被调剂过来的，所以对护理这个专业还是不是很了解的，然后慢慢通过这些和这些临床老师的一些交流，然后就感到了护理护理状态还是不错的，以后这方面还是不错的。

08:27

你刚说转专业的时候，你申请了医学影像学对吧？对。考选择这个是基于哪几方面的考虑，这个应该我们护理方面还是虽然以后就业方面好像还是工作挺累的，我想找一个更加轻松一点的工作，然后医学影像学也是以后工作也是挺不错的，而且比护理工作还要轻松，一些嗯。

09:04

你说医学影像学以后找工作会稍微轻，可能比护理学要稍微轻松一点。但是我刚刚听你去听你老师讲话的时候，你说老师说虽然虽然护理学护理的护士的工作很累，然后但是他有假期工资很高，而且比较好好找工作，甚至一定程度上扭转了你对对于专业以后联系护士工作以后的负面评价。

09:32

可能觉得虽然那一点，但是可能有个假期或者有工资高或者好找一点工作，可能累一点也无所谓。对是我可不可以这样说，可以可以是你觉得这三点就是假期工资还有好多工作，这三点哪一点可能你更看重一点？就对于你个人而言来说，对我个人而言还是工资。假期是好的工作，怎么感觉都挺重要的，对，感觉都挺重要，排个序。其实品牌排不出来的话，也其实反正这三点都挺重要，然后排的话因为工资然后好找工作，然后第三个就是假期，假期和他们比还是稍微差一点，但也是挺重要的。

10:20

影像学其实也应该对于假期和好找工作这两点应该是可可不可以说跟护理差不多，你有去了解过，应该和他差不多，对。

10:34

但是可能工资可能会稍微低一点。比护士要低一点，对可能稍微低一点。但他轻松会会轻松很多。所以你还是比较在意工作或者工作的压力，这方面可能也是挺重视的。你不太能接不太能接受工作压力太大的一个工作对那太累了，还在学习的时候，你会不会也比较排斥比较压力比较大的学习环境或者是学习任务？对我比较排斥，就是强制性的或者压力比较大的时候，我比较喜欢轻松。

11:07

一点的学习，嗯就听听音乐之类的，然后就是找一个安静预防自己学自己的这类的。你刚刚说是了解了国中办信息，我是不是对宣讲会我也听了，然后还有那些他的发的各种文件，我也大致看了文件是指什么文件？就是活动班的他的文件。对国家发展对，所以你了解他主要渠道主要是一个文件，还有一个宣讲会，对。其他就没有了对。但我也对，应该是曾经和一个工程班的学姐聊过，但是也没有什么就没有什么了解你和他聊了啥，聊的就是以后工程班的发展前途之类的，然后就没聊几句，然后就就没了。

11:56

他为什么聊了几句就没了，我也觉得没啥前途是吗？不是和他聊之后，然后他不太愿意，对，然后就然后就不怎么说了，因为别太熟悉。所以你其实还是蛮关注国中班他出来以后的就业。是可以这样说，吗嗯感觉都是挺关注的，都是挺关注那你，除了就业还关注什么？我说这个是不止我一个，就是同学、大概是、同学都挺专注的。

12:29

你的成绩在你就是在大一，你现在是大大二，大一是大一的时候，你大概是20%。是护理学专业是吧？对。其实我比较好奇你们对于前途的定义是高工资还是说好找一个工作，还是说还是说像你自己刚刚说的一样，找一个比较轻松的工作，我感觉我是说你对于周围同学的感知啊，你觉得你作为同学，他们更在意哪一些东西？我感觉你工资高是没有必要太高，但是一定可以不一定不能不能低。

13:20

然后工作这是第一点，工资不能太低。嗯然后呃工作压力最好还是能稍微轻就是低一点，然后最好还有假期之类的，然后就业如果是就业方向特别冷门，就是不太好找工作的话，这也是很不好的。最起码就业是不能差的。你有没有想过以后要干什么，从高中到大学到现在就是我想讲，你的反正高中的时候那时候还是因为不知道自己以后什么专业，还是想的比较少一些，到了现在这个阶段，还是以后想先从事护理工作，先干先做几年，然后如果有机会和有相关的机遇的话，还是可以转行去做别的，因为他还是太累了。

14:23

转行做别的，就是你在工作期间如果有别的机遇，但是这个机遇它具体的方向是什么？我也是不不，太知道看以后发展，如果有机会的话可以去做一下。所以其实你对于各种机会还是处于一个比较开放的心态，就是你愿意接受以后工作中面临的新的机会，还是愿意辞掉像护士这样比较稳定的工作，你也是愿意接受的，对，你有没有考虑过你的父母可能会对此有反对意见呢？

15:04

对他应该会有很大的反对意见，但到那时候应该我也我也老大不小了，他应该也会很尊重尊重我的意见，你是什么样的工作会让想想啊就是辞掉远而稳定的？

15:19

肯定是也也是和前面要求差不多，应该到时候应该和朋友一起和或者和别的合作伙伴一起去做一做一门令，去做另一份工作，然后工作可能会轻松一点，可能会发展前途可能会比护理更好，而且工资也不会可能前期没有护理多，看看后期还是有很大的发展前途吗？是指创业吗？对差不多创业方面。

15:47

然后其他方面也是可以的，还是比较在意发展方向的，因为护理我感觉发展方向不是特别的好，嗯所以，其实我可不可以说，其实你现在对于护理专业或者说以后他可能联系到的职业还是不是非常的认可，因为对没有他讲不是特别满意，但是是可以接受处于可以接受的一个职业。因为刚开始排斥，现在是可以接受。

16:26

你之前有说过护理学专业学起来不太好意思，会不会有是因为别人的评价也会影响到你对他的认知，这肯定会的，因为如果是以一个不是从事医疗方面的，还是一个普通人的，就是我们作为朋友这些人的，他们对一些护理专业还是有存在，对我们这些男护士还是存在很大的那些有点误区，是有点误区。

16:56

我可不可以说你其实也会在意这些外界的对于他们护士对或多或少会在意的，但是以前比较多一点，现在可能稍微少了一点，但是或多或少会有的。你周围的就是男同学也大概是跟你一样的情况吗？对。对护理专业的排斥心理会比较重吗？可能有一些同学差不多，应该都和差不多，或多或少。你刚刚说在了解了宣讲会文件了以后和同学讨论了，觉得国中班有很多不确定的事情，你可以跟我讲具体一点，是什么东西让你感觉到不确定？

17:40

是因为首先因为周围朋友之类的，因为没周围也没有什么国中班的朋友也因为不知道他具体发展，然后通过他读文件以后相去做研究这方面多一点，我个个人这样认为，他是做研究方面多一点。然后我个人对个人感觉我还是不太适合做研究这方面，然后应该我要去做研究的话，应该没有什么前途发展，然后应该拿一个很低的工资或者之类的。

18:16

你说你觉得你不太适合科研工作，你是觉得哪方面不适合？我在实验室里那些我应该做研究我待不住。待不住，对。我还是像和人交流去或者去运动之类的，我不太喜欢在一个房间里去做研究。所以你其实还是比较喜欢做那种和人交流打交道的工作稍微多一点。来和，人交流打工作打对。偏向于这一点，但也不是说一定的对我不是特别外向。但是你假如说要让你一个人待在那儿，静下心来做研究，或者一个人做某样的事情的这种工作，你可能会接受不了就可以。

19:01

对，比如说比如说图书馆管理员这种，他可能很轻松，然后工资也不低，但是假如说要让你一个人一直坐在图书馆，坐在那你可能还是不能接受这种工作是吗？所以反正就有一些排斥，但不是说一定不可以，我感觉是挺排斥的。这种类型的工作可能会让你有抵抵触的心理。对。你说你跟周围同学也讨论过，他们有没有跟你表达他们觉得不确定的事情，或者说或者说觉得这果冻班不太好的点，可以跟我讲一两个你印象比较深的吗。

19:49

我们班原来的班长就现在去当兵了，以前就正常提及过动脉相关方面的。他就说他为什么老跟提空中班参观是上面拍下来的宣传任务不它就是他比较喜欢这些方面。学校的相关各种的他对政策了解之类的，然后他经常就提起工程班，就说你们班长是男的女的？男的，现在已经当兵去了。然后他经常提及过程班，就说以后过程班大致你要读很长时间，然后以后呃就业方面可能也不是很好，然后大致是在实验室研究，他个人认为是感觉比较适合那种比较内向，的比较可以带的住的就是坐的住的那些人适合去那些工作。

20:49

因为我和他交流也比较多，所以说他就感觉果冻班是不太适合他之类的。

20:55

然后我感觉也确实有点不太适合我，你刚说你们班长这个事情我想问一下你们班长算是他是一个特别外向，特别外向。

21:08

他跟你说这个时候是你们俩一对一的聊天，还是说在你们一群人一对一也有，然后一群人也有。因为我们就隔壁宿舍就是在宿舍对互相聊天。他说就业不他说的这些事情，比如说可能要读很长时间就业不好，需要内向的人，需要坐得住的人，他下来这些结论你们都认可吗？我们这对我们感觉还是都是挺认可的，感觉我们男生嗯女生不知道男生这方面还是是我们应该普遍的认知。

21:42

你们护理男生娜娜对。对，就我们班。我比较好奇有一点，你们说就业不好，他其实是学一个学学制很长的，一个学制与其说就业不好，就是个感觉更贴切的应该是就业方向不怎么明朗，因为不知道以后干什么，毕竟以后能找到一个很体面的工作。可不可以说就业方向你们觉得太窄了。好像给你们感觉只能做生殖方面的研究。

22:30

对，然后我们好像这种研究员好像出去不知道去哪找工作，对，因为他感觉他就就业路子比较窄，然后不知道以后的就业方向。发展方向很不明朗。然后方向不明朗，我可不可以说换一个比较直白一点的，他可能不太好找工作。对，这个路太窄了，形容准确吗？对，然后我们当时普遍的认知就是我们这一个一群人的普遍认知，就是你很有可能你读了很多年之后然后，还是很多年之后有什么读博读什么之类的，读很多年之后出来还是找到一个你一个本科生可以找到的工作，假如就是在什么一个药厂，然后或者在一个一个企业就是做一个很普通的工作，你可是一个假如你是一个药学的本科生，可能就能找到工作。

23:38

像你们包括你自己，还有像你们之前经，我看你们好像经常在一起讨论，你们中间就是护理学男生这边有有没有药物考研或者保研之类的，想有这种想法的男人会多一点？考研一开始一开始都希望考研，但是到现在一开始还是有很多同学想考研的，包括我在后期现在对于考研这个事情就感觉很鸡肋，不知道。鸡肋。不知道是否要考虑。一开始想考研就是指大一的那个时候，对大一的时候，因为大一时候还不怎么了解护理相关知识，然后就想考个研感觉更更对以后的发展可能会更好一些。

24:24

发展更好是指会更好找工作吗？还是想对以后可能正好找工作，应该就是一个本科生的身份工作也好找，但是可能会以后的各种升职之类的发展还是更好一些。你那时候想考研考的是应该不是护理的人。当时是当时我只是有一个当时只是一个模糊对一个模糊想法，没有特别说一定要考哪个对。后来怎么就觉得考研没用，考研不会给你发展带来什么影响？是有什么事情吗还是还是老师说上次说男小海楼老师最小的老师，然后就小小楼老师是男女的。

25:10

你不是，然后他就说他就说关于护理学生，假如考护理员的护理员护理员的话，如果你考别人的话，可能不怎么不太不太切合实际，不好考之类的。

25:22

然后你要考护理人员的话就感觉很鸡肋，如果你考如果你考虑的话，花费两年时间去考研，然后可能对你以后的工作没有什么太大的帮助，可能会稍微好一点点。

25:37

但是如果你不考研，你会有你不考研，你在别人考研的两年的期间，你就获得两年的工作经验，你赚了两年的钱，也会积攒了两年的人脉，就可能比那些去花费两年时间考验的人会更好一点。

25:52

所以他考不考研各有利弊，所以就很激烈了。这个是老师说的是吗。对他说的这个是大概意思，然后我给总结了一下，这是我个人看他是不是觉得考研可能出来找的工作也和本科生找的差不多。对，他就是以他们应该给你带来的感觉。对以他们医院为例，就是说本科生和他们研究研究生的工作没有什么几乎没有差别。护理学的对对对，所以像抛开你自己你周围的人的话，是不是想把这么一说跟你差不多？

26:37

也是经过这些年的就这一两年的学习，感觉可能考研并不会给你们带来更好的工作机会，更高的工资，所以可能你周围人对考研这个事情没有很大的热情，所以热油但是不是很大了现在。就是说考研的话，还是说以后再看你也是愿意去考，只是说你有以前那么想考。对热情小了一点。你大一大二现在目前学习到现在有没有什么让你觉得压力很大的事情？

27:25

压力很大，这样我想一想，因为最近最近没有什么压力，我发压力我感觉压力还是看得很轻的。我感觉因为我高中的时候可能会高中的时候很胖，然后我就没有什么自信，然后可能会压力大一点，但是随着大学的然后慢慢的变得更自信了，然后压力也有那小一点，然后现在看到最什么应该都看得很开。压力的来源是跟自信有关是吗？我感觉是因为当时可能还挺幼稚感觉。假如你考的不好，我想怎么就是非常的非常的感觉不好，然后很有很多压力，但现在看得很开了。

28:10

你是现在心态变好了，对心态心态变好了，对。反正到目前为止有没有发生过什么让你觉得比较难的事情。

28:23

很难的事情，对大一大二的学习就是当时转专业的时候也挺难的。

28:29

对大一时候涨价，因为想涨价因为我们还是还是关于小专业方面有一些压力，就是保证不怕亏啊，然后成绩考勤然后之类的，还有那些几门学科要学得很好，初三对。这些方面大概是关于学习方面的吧。有没有发生过什么让你觉得特别失望的事情？特别失望。那就准备没转成功。因为没有去到心理的专业，对这是有一些失望的，很有希望的。

29:02

你后来是怎么调节的呢？因为调节这种失望情绪就是有跟父母对肯定有，然后没转成之后父母还是也没有10分的在意，然后就说搞没转进去，然后一切向前看，然后随着时间就慢慢的转变了，就没有很大的一个释放感。有没有参加过大创挑战杯之类的？这方面没有。所以我还是喜欢那些户外的活动。

29:42

所以你上完课以后还是喜欢把时间用在活动。通过活动对打球一般下课之后再说一下歇会，吃个饭就去打打打球了，打到晚上可以谢谢他打回游戏也，可能你有参加什么学生会或者社团之类的？我大一的时候参加学生会，后来我就退了，然后现在是团支书工作也挺多的，然后就没参加大一。参加的是什么时候？大一的时候我不喜欢，但有时候我不因为我喜欢体育方面打篮球，所以我加入了体育部后来加入之后发现体育部和体育没有关系，我就给退了，我就发现体育部好像是别的部门的附属部门什么杂活都找我们，我就退了就很生气。

30:28

什么杂活都找，你们是什么杂活啊？搬桌子搬椅子，然后这边小通讯搞你给补，那边小通讯搞你给补。对后勤保障部差不多，然后只有一个运动会才算是体育部的东西。但是也是班班桌子班班椅子。你想当中提出应该干嘛？体育部你看像学校篮球赛足球赛，我感觉体育部应该多组织这些东西，但是这些东西不是在说体育部组织的，但是可能我更想的是一个参与者，而不是去斑斑一个去组织这个东西，可能对体育部这个东西还是有一个错误的认知。

31:09

它相当于是一个也是一个管理方面的我也，不太喜欢感觉里面不是我想象中的体育部，然后里面都是一些女生，也是一些女生也是一些不怎么爱体育的人在里面，然后我就退了。

31:25

你现在做团支书，你感觉你今年这个学期结束你还会退吗？还行，暂时应该不会退，但是挺累的挺烦人的真的。天天都有活动，天天要写通讯稿。嗯其实本质上来说团支书的工作和学生会的工作其实我觉得应该差不多。嗯对为什么？可能会不会是因为学生会学会就是会有很多额外的附加的跟你无关的工作。最重要的推动，对，但是团支书的话可能就是你觉得你有没有思考过为什么可能我团队还能干下去，但学会就干不下去，因为我可能对还是一个归属感，我感觉我是我们班级的一个故事馆还是挺强的，我可能为班级工作之类的，我可能会非常的没有什么怨言，然后因为对自己同学可能，但是对学生会的认同感可能就少一点，就感觉在学生会不知道在给谁工作，对感觉没有工作，没有方向，没有意义。

32:31

现在在那搬上桌子搬的，椅子感觉就是一个劳力。

32:36

说的意义是指什么意义？会不会对你刚刚说归属感的问题，还有你说学生会工作没有什么意义，就是做的工作没有什么意见，因为我可能比较强壮一点，然后就是我的主要任务就是假如是，小学校哪个比赛有去开始了，我就去买水，然后搬完之后就搬个椅子进东西，确实没有什么对个人的个人的那些成长还是没有感觉没有什么帮助。

33:10

你觉得团支书的工作对你有帮助，吗唉还是有的和因为从作为团支书，然后一个班级的相当于一个组织者和管理者，然后你和同学今天交流可能会更多，然后和同学的这些关系可能会因此而变得更加从下了解更加了解一些，然后和中介关系会变得更好一些。

33:33

对二有大二参加当这个团队之后，团支书之后就和同学会计还是有了一个很好的提升，因为大一的时候就缺乏了解，没有一个正式的途径，然后当团队社会之后就了解了很多。所以综合感觉下来其实下学期可能还是会继续干嗯。对。对，应该会干。这是它内容。刚刚这个老师问你有什么压力的事情，你说在高中时候比较胖有压力，你觉得是因为是他们是因为你觉得压力胖和压力是怎么联系在一起的，因为以前不胖，然后到高中突然就胖了，他就成为以前是初中时候，所以初中时候还是和查不清的这种体型差不多，然后高中就胖，因为这个胖本身就成了压力，因为外在形象还是挺在乎的，就是一个压力。

34:32

人自信的时候可能也会觉得做事情会成功，对你自信的时候你也会心态也可能会更好，然后对就方面会变得更好，然后你很胖的时候，没有自信的时候做什么可能都不太行。

34:44

所以外界评价其实对你带来的影响，所以说还是挺大的，对。

34:54

这个可不可以联系到你考虑专业的时候会考虑到就业，也有一部分是因为外界跟外界的一些社会评价说还是你会考虑职业地位，这个事情肯定会考虑。

35:11

我个人自尊心还是挺强的，这些东西还是有点在意的。他是了解过国中班，然后没有去的，对。对。有报有报名是吗？没报名没报名他是符合条件，我是符合条件，但没有去报，然后没有去参加。整机前20%啊啊对。对就是2%就20%是肯定没有。因为你对当时报股东的时候应该是9月份是吧？转进去之后几天？应该是暑假期间。他不是那个时候已经刚刚开学了。开学了刚开学他，那时候你已经做团支书了。那时候我即将座谈的时候，差不多已经到那个阶段，你说你们班长就当兵了，刚走了几个月，刚走大大二下才走。

36:39

下面一个问题是我刚刚问了压力比较大，还有觉得很难的问题，对，那有没有让你觉得特别骄傲的事情？特别骄傲的事情，没有话就不讲，没有我想一想特别骄傲的事情，或者说换一个可能比较好讲一点欣慰的事情，自豪或者有成就感的事情，做了一件觉得让你特别大学期间，或者你要是觉得大学期间没有什么的话，讲高中期间的我觉得应该也可以发现特别自豪或者有成就感的事情，有这个也没有就不说。

37:28

应该没有，反正我平淡其实对平静偏于平淡，没有什么特别自豪的事情，或者让你特别开心的事情。特别开心。核心就是平常不仅是学习了你的生活，也可以和学习这方面没有什么值得我说刚刚刚说什么骄傲成就感那些事情，你学生会团支书或者哪怕你什么谈恋爱，或者说你和你家里人或者或者和周围人这种都没有什么让你印象比较深的。

38:10

反正平时你说骄傲说自豪，好像缺点意思你就说开心。教学的感觉还挺不少的，就平时和各种同学开个玩笑之类的，然后还有平时在球场打球，然后和人家竞技，不管输了也好，盈利也好，也也是挺开心的。

38:29

嗯但是就是好像没有什么说一定要说有成就感自豪这种事情好像没有。对比赛打赢打比其实还挺自豪。但是最近打赢比赛比较少，有没有赢得特别漂亮的一次让你印象比较深。因为我打球也是还是比较靠身体打的，然后也不是特别厉害。然后最近前段时间三号杯，然后打了6场就赢了一场，第一场还是挺骄傲的，然后后来就没了是跟你们去同一个学院的人，不是是一个三号杯，是各个学院为一个单位，然后各个学院之间打。

39:11

对，你和你们护理学院的同学一起对。对，晋级。对。其实就是我跟你说其实除了学习这一块，就是你自己的业余时间主要是用于打球或者运动，还有一个就是团支书这两块。

39:33

对，然后我也经常出去玩出去和一些在南京的一些高中同学，南京的各种景点去玩。

39:40

这个也是经常去，然后你光打球一天也不能净打球太累了。

39:48

我这个问题你可以不回答你有没有女朋友？再没有暂时没有。所以其实你的业余时间主要是和朋友在一起。对，跟朋友在一起，朋友同学在一起。你刚刚说转专业没有转成功，会让你比较失望。你刚刚老师问我，我没有记太清楚你是自我排解掉的吗？就是失望的，情绪。自我就消化掉了，就应该还是自我是一方面，然后还有家长我爸妈一般怎么处理负面情绪？不开心的时候。

40:54

不开心的时候就是一方面就和我爸妈还有这些朋友们交流，然后是一方面他们也会安慰我呀我，然后我还有前面同学和我一样去报转专业也没有成功的，然后我们互相互相互相他也没转成功也没成功，没转成功有一种慰藉，然后业余时间呢也去去一些娱乐活动，就是打打球，然后或者在宿舍和同学打游戏啊，然后慢慢就消消解掉了。

41:27

主要还是和别人沟通那，如果自己闷着的话可能会慢一点。你说在大一的时候，因为有转专业的目标在那边，所以你觉得考得不好，可能会给你带来很大的压力，你现在其实对于现在你来说已经没有再考虑全专业了，对吧？应该已经过了时机了。现在假如说你在学业这方面，你还会对自己的施加压力吗？还是会的？

41:54

因为我现在我已经不太心里也内心深处已经不是很在在乎成绩了，我更多的可能就是一些以后你就业相关的知识，如果你以后就业的话，你不这些知识你是不能少的。对。然后我已经是成绩是方便你，最好是不要把课程，然后这样会给你以后的生活一方面会带来很大麻烦。对另一方面如果你这些相关知识如果你没有掌握好的话，对你以后的工作也是不太有利的。你说我可不可以说其实你现在和大一相比，你在大二期间你的学业压力可能没有大一那么。

42:35

对，我以为你对学业没有那么的看重。对。大一是比较看大，一是比较注重成绩，现在对是他不一定是不重视成绩的，但是我还是感觉具体是挺重要的，能能达到自己毕业的要求，对。达达到必要的时候就是一个这是一个最基本条件，然后为以后工作，然后专业知识必要的知识，对学校必要知识以后不能这方面你不能不可能是很瘸腿的，这方面的不行。

43:13

除了学到必要的知识这一块，对于学业的成绩来说，其实没有什么太大的压力。对，但是挂科是最基本的，不挂科就在不挂科的基础之上，我能学到自己以后工作能需要的知识技能，我觉得就够了。考得高不高其实无所谓，对，这很正确。你比方说以后找找了护理的工作，很有可能我可不可以说很有可能是会转行做别的？

43:45

我觉得现在说现在在学习的时候是想学习以后工作所需要的技能，但其实你现在所学的东西可能以后还是跟护理职业护士这个职业是相关的。

44:00

你刚刚又说你有可能会转行，会不会就会可不可以说现在开始觉得现在学的东西可能对以后的帮助其实没有那么的大。

44:11

我说我没有这样觉得，我就是因为现在你退以后的就业，你还是可能转行，还有其实有很大的概率变化，但是护理这个工作你还肯定还是要做几年的，这几年你也不能是因为因为这些专业知识缺乏，让自己的工作变得很颓废之类的，肯定是在你以后在从事护理工作的几年之中，你已经是一个好好做的心态。

44:38

然后就这样肯定。

44:42

我可以问一个假设的问题，假设你现在是处于大一期间，其实还是有答有很大的学业的压力在的。你学了很久学了一下你一直想得到的一个技能，这个技能可能可以是大学的技能，也可以是学习上面实验操作的一个技能，或者说某一个护理上的操作的技能。你学了很久终于学会了。另外一件事情是你学了很久，终于考到了班级的前几名，你觉得哪一件事情会让你更加兴奋？

45:18

一个是学到一项技能，一个是考到了班级情形。都是努力了很久才拿到了这个结果。你觉得哪件事情会让你更兴奋？我感觉还是一个渠道模像技能，我感觉我大一的时候对成绩要求没有必要是没有说那种那种特别靠前，那种有一个转专业资格，我大一的时候是只要一个转专业资格就可以。

45:47

转专业资格不是看成绩排名吗？对，他没有硬性要求，就是你博华科是一个非常硬性的，然后如果你博华科你还会有不管你是多少名，你就可以去报一些专业，但你成绩靠前的话，你可以去报另一些更好的专业。

46:05

所以其实对于现在你来说，可能我觉得应该是学习技能会更加更加的对学习技能，然后学习，但是在大一那个时候，其实你可能和现在想法还是差不多，可能也没有一定要考到前几名才让你产生比较兴奋，对有成就感，对。

46:37

其他的我好像没有。你刚刚说还有一个事情，你刚刚说你们班长说啊时间很长，这个因素会不会影响到你可能学习的一个预判，就是可能觉得学制太长了，我可能学不下去，会不会有这种感觉？

47:02

在呢他就说很长时间为什么会成为一个你学学很长，你学习很长就是你你就是一个还是旁边如果是单纯的只是学制很长的话，其实并没有影响，但是考虑到一些你社会方面就是家庭方面，还有一些朋友之类的，别人都已经工作好多年了，然后已经已经算算事业有成了，然后你还在读书，可能这些感觉会自己还是挺不舒服的，还有一些家庭家庭方面的原因，你不可不可能一直在学习，可能会影响自己成家，是这个意思吗？

47:41

还是成家也是一个原因，就是不学可能学学了很久了。

47:45

家你说的家庭主要是家庭是哪？一方面的对。父母的一方面。你我遇到工作可能家里的我爸妈会更轻松。这方面的你说的社会是指社会周围人对你的评价，对对就是，别人已经工作好几年了，对事业有成了，然后你孩子上学就感觉不太好。所以很很长的学制对于你来说其实算是一个蛮大的一个负面影响。算可以这样说吗你刚说了好几个负面影响，我再给你列一下，也是很长的一个学制，还有就业不好，或者说就业方向比较窄，就业不明朗。

48:30

还有一个是这可能需要一个比较内向的学生，比较坐得住的人才能继续学下去。你觉得这三个对你来说哪一个是最大的，一个就是劝退你的一个就业不明朗。就业不一样，如果你就业很明亮的话，就是你花费一些时间长的就是比较长的学制的话感觉也是有回报的，但是如果你就业不明朗，然后还花了很长时间去学，它就感觉是代价还是挺大的。

49:00

你觉得很长时间和需要一个坐得住的一个人，这两个点你觉得哪一个点你觉得更让你无法接受？常学制和需要一个比较内向的人来做的住来做可以还是长学制？感觉代价挺大的，假如说我现在说的是一个假设题，现在股东班已经出了一个在你们转专业那个时候出了一个政策说毕业包装备就是前提是你博士毕业，博士毕业以后留在学校，其实这个时候对你来说是不是算是就业比较明朗的一个学学学习方案可能会好一些，你可能会去选择他们，我可能我感觉是有可能选择他对。

49:49

还是因为但是他可能会会要你花费你很长的时间，就要读很长时间的书，你加一加上5+1+3还是最好的情况。不是，这是最短的吗，对最短的情况就是你六年级假如没有答辩通过的话，可能还要更需要更长的时间读研究生。

50:10

读硕士我觉得还是可能一下会考虑一下他，还不一定说一定要去或一定不去，对，但是不会像大一那个时候说我肯定不去。

50:24

嗯差不多对你考虑的时候，你可能会会听外界的意见吗？会和外界交流吗？肯定会的，我一般他们会动摇你的意见吗？你觉得动摇就是回想自己做每一次做决策的时候，包括报告中考高中的时候考，大学的时候，还是影响会采纳他们的意见，会参考的嗯，其他没有什么问题。其他没有什么问题。谈了多久？谈了。对。
