# Supplementary material for: Why do you choose this program?—A decision-making model of medical students based on grounded theory
Source: PLoS One. 2023 Sep 15;18(9):e0291634. doi: 10.1371/journal.pone.0291634 (PMC10503722; doi:10.1371/journal.pone.0291634)
Supplement: S1 File — (ZIP) [file pone.0291634.s001.zip › RAW DATA/P7.docx]

00:00

Let me first think about the purpose of our interview. The main purpose is to restore your promotion from the activity class to the registration and admission to the final study. Are you a student now? no. He is not that he is that condition is not, yes, I just want to ask what type you are, I am the kind of person who did not report and did not meet the conditions. Which major are you? I am a nursing major. I just want to restore the whole process of the freshman year, so from the important things or your emotions, to the points that impress you more deeply, you can all say yes, okay?

00:37

Then before I want to say a statement about the moral and ethical requirements of time.

00:41

In this interview, the interviewees participated on the principle of equality and voluntariness. The interviewees must truthfully express their thoughts and cognitions and confirm that they meet the interview conditions. The interview process will be recorded and recorded, and will be used for scientific research in an anonymous form, and will not be disclosed to any third party. During the interview and after the interview, the interviewee has the right to cancel the researcher's right to use the audio recordings. Are you Xiaoping agreeing to you? Grade 11 is not a grade 19 teacher. Level 19 Nursing Pair. So it was around this time last year, for last year, you should first talk about what you know about the event and how you know it.

01:19

Regarding the various methods, I was a little embarrassed to be admitted to the nursing major as a boy, and I wanted to change majors, and then I also learned that our class is also a certain way. Then after hearing about the work class, I went to find out about the situation. Then in the later stage anyway, I will discuss with my classmates about the registration of the engineering class and so on. Later, I felt that there was too much uncertainty in the activity class , because there were no people around us who did not have this aspect, so we did not sign up or sign up during the safe period.

02:03

Did you choose the nursing major when you communicated? Not that most of this adjustment is made up. What did you start with? I chose it at the beginning, I didn't think much about it at first, I applied for the introduction, because the score was not too high. Did you have to apply for medicine when you signed up in high school? Or are you a liberal arts student or a science student in medicine? I'm a science student, I study physical chemistry. Did you apply for both engineering and science at that time, or did you apply for science? All reported, but I still prefer the medical class.

02:41

Are you supposed to be 6 or 6 schools? When you applied for the college entrance examination, it seemed that there should be several medical schools. I applied to us. Because I am from Xuzhou, I applied to Nanjing Medical University and Xuzhou Medical University. The others should be engineering, and science and engineering. You, you discussed with your family when you signed up for the college entrance examination, or discussed it. We discussed it together, and it turned out that the medical aspect was quite good, and then I reported the general direction to them.

03:14

Has it affected you in any way? The influence was quite big. At first, they thought about it and then thought that I wanted to apply for medicine and science and engineering, and then my parents wanted to apply for demonstration classes, but then so in those 6 volunteers, I I also filled in 1 of his, so in fact, can I say, in fact, you don't really want to go to the normal class, I don't like the normal class, you will accept the opinions of your parents, but you don't like it. .

03:51

He, but I don't know about permission, my parents agree. My parents hope that if they are not admitted to medicine, they can also go to those teachers.

04:06

Yes, so there are not many contradictions in applying for volunteers. You applied for imaging technology again, and later you were transferred to nursing. Do you think you were embarrassed when you went to nursing. What do you mean by embarrassment? It felt right at first. When I was in high school, I was quite repulsive to this aspect, because I got this notice just after the college entrance examination, and I felt that it was still too uncomfortable for men. After I got to know it later, I found that it was not bad.

04:40

Is that you and I still have the opportunity to change majors after freshman year? Of course, I hope that, er, there will be better development and go to other majors. But now I don't know anything about nursing, and I still feel pretty good. You said uh didn't know much about male nurses at first, right? Later, I felt that it was very good. It meant that it was good, and it meant that there was no future employment. employment. Good job, yes. this aspect. How did your parents react when you first entered nursing? At the beginning, my parents also felt that as a boy, it was still difficult to ride a bicycle in terms of nursing. I'm sorry.

05:22

But later through my understanding, and then I communicated with them and so on, and their impression of the head nurse was also quite changed. It's not that they rejected him, they changed their minds because of what they told him. Talking about future development, the future employment direction is good employment, and there is nothing in the future that there are still great employment opportunities .

05:48

Can I say that your parents are actually quite concerned about my employment problem, because they have always asked you to study in the normal school and medical school. These are subjects with relatively stable employment. They hope to have a stable career in the future. , they care more about whether you can find a stable job, can you say that right?

06:10

You didn't apply for changing majors at that time, did you? At that time, when I applied for a major transfer, I applied for medical imaging, but I did not transfer it. I would like to ask, if you deepen your understanding of the nursing profession, is the cognitive channel probably listening to what the teacher says or is it just right? We have a lot of apprenticeships in our freshman course, and we also have some communication with those clinical teachers. They are through our communication with him, we still realize the future employment after the major, and the employment is something that feels good or okay. of.

06:48

You can talk about the things that impressed you deeply when you were apprentices or communicated with teachers, and the things that touched you more deeply. You should go to Hailou during an apprenticeship. In classroom 51514, there was a teacher from the First Affiliated Hospital of South Nanjing City, and he was also a nurse, and then he told us some of his experiences. After he became a story, he was very tired after work. One of them was on the one hand, and many of them also had vacations, too, and the salary was quite good. Then he told them that the most important thing was to find a job. Don't worry about work in the future.

07:42

Your conversation this time left a deep impression on you. You discussed it with your classmates, and you should have attended with our classmates. There was a discussion between us around, you said this is around my high school friends or something? Alumni, do they touch you as big as you? still? For them, we started with 9 boys one by one. All of them should have been transferred to the nursing profession, so I still don’t know much about the nursing profession, and then slowly passed through these and these clinical trials. After some exchanges with the teacher, I felt that the nursing status is still good, and this aspect is still good in the future.

08:27

When you just said you changed your major, you applied for medical imaging, right? right. What are the considerations for choosing this test? It should be in nursing or in the future, although the employment seems to be quite tiring, I want to find a more relaxed job, and medical imaging is also a good job in the future. And it's easier than nursing, some um.

09:04

You said that finding a job in medical imaging will be a little easier, maybe a little easier than nursing. But when I just listened to you to listen to your teacher's speech, you said that the teacher said that although the nursing nursing nurse's job is very tiring, he has a high vacation salary, and he looks for a job better, and even reversed to a certain extent Your negative comments on the future of professional contact with nurses.

09:32

may feel that despite that, it may not matter if there is a vacation or a higher salary or a better job, and it may be a little tired. Yes, can I say this? Can it be that you think these three points are vacation pay and there are still a lot of jobs. Which of these three points may you value more? As far as you are concerned, it is the salary for me personally. Vacation is a good job. No matter how you feel, it is very important. Yes, it is very important to feel. Sort it out. In fact, if the brands can’t be ranked, in fact, these three points are very important anyway. Then, if they are ranked because of the salary, it is easy to find a job, and then the third is the holidays. The holidays are slightly worse than theirs, but they are also very important.

10:20

In fact, imaging studies should also be about the two points of vacation and finding a job. Can it be said that it is similar to nursing. You have gone to understand it, and it should be similar to him, yes.

10:34

but maybe the salary could be a little lower. A little lower than a nurse, yes maybe a little lower. But he will be a lot easier. Therefore, you are still more concerned about work or the pressure of work, and this aspect may also be taken seriously. You are not very able to accept a job that is too stressful. It is too tiring. When you are still studying, will you also reject a more stressful study environment or study task? It is more repulsive to me, that is, when it is mandatory or the pressure is relatively high, I prefer to relax.

11:07

Study at one o'clock, um, just listen to music and the like, and then find a quiet place to prevent yourself from learning your own. You just said that you learned about the information from the Central Office of the Central Committee of the Communist Party of China. Did I also listen to the briefing session, and then there are various documents issued by him, and I also roughly read what the documents refer to? It is his document of the activity class. Right for national development, so you understand that his main channel is mainly a document, and a briefing session, yes. Nothing else is right. But I'm also right, I should have talked to a senior from an engineering class, but I didn't know anything about what you talked to him about. The conversation was about the future development of the engineering class and so on, and then I didn't talk. A few words, and then it's gone.

11:56

Why did he disappear after a few chats? I don't think he has a future, right? It's not after talking to him, and then he doesn't want to, yes, and then he doesn't say anything, because don't be too familiar. So you are actually quite concerned about his employment after he leaves the middle school class. Is it possible to say this, um, I feel that I am very concerned about you, so what else are you concerned about besides employment? I said this is not only me, but my classmates, probably, my classmates are very focused.

12:29

Your grade is in your freshman year, you are a sophomore now, and your grade is about 20% when you are a freshman. It's nursing, right? right. In fact, I'm more curious about whether your definition of the future is a high salary or a good job, or is it better to find a more relaxed job as you just said, I think I'm talking about your perception of your classmates around you. , What do you think you, as a classmate, care about more? I feel that your salary is not necessary to be too high, but it must be possible, not necessarily low.

13:20

Then work This is the first point, the salary can not be too low. Well, er, the work pressure is better to be a little lighter or lower, and then it is better to have vacations and the like, and then if the employment direction is particularly unpopular, even if it is not easy to find a job, this is also very bad. At least employment is not bad. Have you ever thought about what you want to do in the future? I want to talk about it from high school to university to now. Anyway, when you were in high school, you still thought less because you didn't know what major you wanted in the future. At this stage, it is still In the future, if I want to engage in nursing work first, I will do it for a few years first, and then if there are opportunities and related opportunities, I can still change careers to do other things, because he is still too tired.

14:23 Change

careers to do other things, that is, if you have other opportunities during your work, but what is the specific direction of this opportunity? I also don't, I know too much about the future development, if there is a chance, I can do it. So in fact, you are still relatively open to various opportunities, that is, you are willing to accept new opportunities in your future work, or are you willing to quit a relatively stable job like a nurse, you are also willing to accept, yes, you have Haven't considered that your parents might have objections to this?

15:04

There should be a lot of objections to him, but by then I should be the boss, and he should also respect my opinions. What kind of job do you think about? Just quit. Falling far and stable?

15:19

is definitely the same as the previous requirements. It should be done with friends and or with other partners to do one order, another job, and then the work may be a little easier, maybe The development prospect may be better than nursing, and the salary may not be as much as nursing in the early stage. Let’s see if there is still a great development prospect in the later stage? Do you mean entrepreneurship? Pretty much on the entrepreneurial side.

15:47

Then other aspects are also possible, but I am more concerned about the development direction. Because of nursing, I feel that the development direction is not particularly good. Well, in fact, can I say, in fact, you may contact the nursing professional now or in the future. The occupation is still not very recognized, because I am not particularly satisfied without him, but it is acceptable to be in an acceptable occupation. Because it was initially rejected, it is now acceptable.

16:26

You have said before that the nursing major is embarrassing to study. Could it be because other people's evaluations will also affect your perception of him? It definitely will, because if one is not engaged in medical treatment In terms of aspects, it is still an ordinary person, that is, we are friends. They still have some misunderstandings about some nursing professions, and there are still big misunderstandings about us male nurses. It is a little misunderstanding.

16:56

Can I say that you actually also care about these outsiders who will care more or less about them, but they used to be a little more, now maybe a little less, but there are more or less. Are the male classmates around you probably in the same situation as you? right. Will the rejection of the nursing profession be heavier? There may be some students who are similar, and they should all be similar, more or less. You just said that you discussed with your classmates after learning about the documents of the seminar, and felt that there are many uncertain things in the middle school class. Can you tell me more specifically, what makes you feel uncertain?

17:40

Because first of all, because of the friends around, because there are no friends in middle school around, and because I don’t know his specific development, and then through his reading documents, I will do more research on this aspect, and I am personally like this. I think he is doing research a little more. Then I personally feel that I am still not suitable for doing research. If I want to do research, there should be no future development, and then I should get a very low salary or something.

18:16

You said that you don't think you are suitable for scientific research work. What aspects do you think you are not suitable for? I can't wait in the lab where I'm supposed to be doing research. Can't wait, yes. I still like to communicate with people or go to exercise and so on, I don't really like doing research in a room. So you actually prefer to do the kind of work that communicates with people a little more. Come and communicate with people and fight for work. Biased towards that, but not necessarily to me not being particularly extroverted either. But if you say that you are asked to stay there by yourself, calm down and do research, or do some kind of work by yourself, you may not be able to accept it.

19:01

Yes, for example, for example, a librarian, he may be very easy, and the salary is not low, but if you are asked to sit in the library all the time, you may not be able to accept this. kind of work? So anyway, there are some exclusions, but it doesn't mean that it is absolutely impossible. I feel that it is quite exclusionary. This type of work can make you resistant. right. You said that you have also discussed with the classmates around you. Have they expressed to you anything they feel uncertain about, or that they think this jelly class is not very good. Can you tell me one or two things that make a deep impression on you? .

19:49

The original squad leader of our class is now enlisting in the army, and he has normally mentioned arterial-related aspects before. He just said why he kept mentioning the air shift visit was the propaganda mission photographed above, not because he liked these aspects more. He knew about the policies related to the school and so on, and then he often mentioned the engineering class, saying that your monitor is a male or a female? The man is now in the army. Then he often mentioned the process class, saying that in the future, the process class will generally take a long time, and er, the employment may not be very good in the future, and then it is generally researched in the laboratory. He personally thinks that it is more suitable for that kind of comparison. Those who are introverted, those who can live with them are those who are sitting and living are suitable for those jobs.

20:49

Because I communicated with him a lot, he felt that the jelly class was not suitable for him.

20:55

Then I feel that it does not suit me a bit. You just said about your squad leader. I want to ask your squad leader if he is a very outgoing, especially extroverted person.

21:08

He told you that this time is a one-on-one chat between the two of you, or it is also a one-on-one chat among a group of you, and then a group of people. Because we are in the dormitory next door, we chat with each other in the dormitory. He said that employment is not what he said. For example, he may have to study for a long time and not be able to get a job. He needs introverted people and people who can sit still. Do you agree with his conclusions? We still feel that we are quite recognized by us. It feels that we boys and girls don't know boys. This aspect is still our general understanding.

21:42

You nursing boy Nana is right. Yes, just our class. I'm a little curious. You said that the employment is not good. In fact, he has studied a school with a long academic system. Rather than saying that the employment is not good, it feels more appropriate that the employment direction is not very clear, because he does not know what to do in the future. , after all, you can find a decent job in the future. Can you say that the employment direction is too narrow for you? It seems to you that you can only do reproductive research.

22:30

Yes, then we are like this kind of researcher who doesn't know where to find a job when he goes out, yes, because he feels that his career path is relatively narrow, and then he does not know the future employment direction. The direction of development is unclear. Then the direction is not clear, can I change to a more straightforward one, he may not be able to find a job very well. Yes, this road is too narrow, is the description accurate? Yes, then our general perception at that time was the general perception of our group of people, that is, it is very likely that after you have read for many years, you will have a Ph.D. or something after many years, and you will read for many years. After you come out, you will still find a job that an undergraduate student can find. If you are in a pharmaceutical factory, or in a company, you will be doing a very ordinary job. If you are an undergraduate student of pharmacy, you may be able to do it. Can find work.

23:38

Like you, including yourself, and like your previous experience, I see that you often discuss together, among you are the boys in nursing, whether there is any drug entrance examination or insurance research, etc., I want to have this idea The men will be a little more? At the beginning of the postgraduate entrance examination, I hoped to take the postgraduate entrance examination, but now there are still many students who want to take the postgraduate entrance examination, including me in the later stage. Chicken ribs. Don't know if it should be considered. In the beginning, I wanted to take the postgraduate entrance examination, which meant the time of my freshman year. When I was a freshman, because I didn’t know much about nursing related knowledge in my freshman year, I wanted to take the postgraduate entrance examination and felt better for my future development.

24:24

Does better development mean better job search? I still think that I might be looking for a job in the future. It should be a job as an undergraduate, but it may be better for various promotions in the future. At that time, the person you wanted to take the postgraduate entrance examination should not be a nursing person. At that time, I just had a vague idea of a fuzzy pair, and I didn't specifically say which pair must be tested. Later, why do you feel that the postgraduate entrance examination is useless, and that the postgraduate examination will not have any impact on your development? Is there something wrong or is it still the teacher who said last time that the male Xiaohailou teacher was the youngest teacher, and then the Xiaoxiaolou teacher was male and female.

25:10

You are not, and then he said he was talking about nursing students. If you are a nursing assistant, if you are a nursing assistant, if you take someone else's exam, it may not be very realistic, and it is not easy to take the exam. .

25:22

Then you feel very tasteless if you want to take the exam for nursing staff. If you take the exam, if you think about it, it takes two years to take the postgraduate exam, and then it may not be of much help for your future work, and it may be slightly better. .

25:37

But if you don't take the postgraduate entrance examination, you will not be admitted to the postgraduate entrance examination. During the two years that you are in the postgraduate entrance examination for others, you will gain two years of work experience, and if you earn two years of money, you will also accumulate two years. Networking, it may be better than those who spend two years on the test.

25:52

So there are pros and cons for him to take the postgraduate entrance exam or not, so it is very intense. Is this what the teacher said? What he said to him was roughly what he meant, and then I summed it up. This is whether I personally see whether he thinks that the postgraduate entrance examination may find a job that is similar to that of an undergraduate student. Yes, he's the way they're supposed to make you feel. To take their hospital as an example, it means that there is little difference between the work of undergraduates and their research graduate students. Nursing is right, so it's like putting aside yourself and the people around you, don't you want to say this to you?

26:37

After these years of study for one or two years, I feel that the postgraduate entrance examination may not bring you better job opportunities and higher wages, so maybe people around you do not have a great deal about the postgraduate entrance examination. Enthusiasm, so hot oil but not much now. In other words, if you are going to take the postgraduate entrance examination, or you are willing to take the exam in the future, it is just that you want to take the exam as much as you used to. A little less enthusiastic. Are there any things that make you feel a lot of pressure?

27:25

There is a lot of pressure, so I think about it, because there is no pressure recently, I feel the pressure is very light. I feel that because I may have been fat in high school, and then I have no confidence, and then I may be a little more stressed, but gradually become more confident as I go to college, and then the pressure is also less. , and now see the most everything should be very open. Is the source of stress related to self-confidence? I feel like it might have been childish at the time. If you don't do well in the exam, I think it's because you feel very, very bad, and then there is a lot of pressure, but now you can see it very well.

28:10

Your mentality has improved now, and your mentality has improved, yes. Anyway, has anything happened so far that made you feel more difficult.

28:23

It's a very difficult thing, even in the first and second year of the freshman year, it was also very difficult when changing majors.

28:29

For the price increase during the freshman year, because we want to increase the price because we still have some pressure on minor majors, that is to ensure that we are not afraid of loss, then grades, attendance, etc. There are also a few subjects to learn very much. Okay, the third grade pair. These aspects are probably about learning. Has anything happened that made you feel particularly disappointed? Extremely disappointed. Then prepare to fail. Because I didn't go to the major of psychology, I was a little disappointed and hopeful about this.

29:02

How did you adjust later? Because to adjust this disappointment is to have a right with the parents, and then the parents still don't care about 10 points after the transfer, and then they say that the transfer is not in, and then everything looks forward, and then slowly over time After the transformation, there is no great sense of release. Have you ever participated in the Daiso Challenge Cup or something? Not in this regard. So I still like those outdoor activities.

29:42

So you still like to spend time on activities after class. Through activities, we usually talk about taking a break after class, and then go to play and play after a meal. You can thank him for playing games in the evening. Maybe you have participated in any student unions or clubs? When I was a freshman, I participated in the student union, and then I retired. Now I have a lot of work as the secretary of the Communist Youth League, and then I didn't participate in my freshman year. When did you participate? I didn't like it when I was a freshman, but sometimes I don't play basketball because I like sports, so I joined the sports department and later found out that the sports department had nothing to do with sports, so I quit, and I found that the sports department seemed The affiliated departments of other departments are looking for us for all kinds of chores, and I am very angry when I quit.

30:28

Looking for all kinds of chores, what kind of chores are you? Move the table and move the chair, and then the small communication here will help you to supplement, and the small communication over there will help you. It is similar to the logistics department, and then only one sports meeting can be regarded as something of the sports department. But it's also a class table and class chair. What do you think it should do? The sports department looks like a school basketball game and football game. I feel that the sports department should organize more of these things, but these things are not meant to be organized by the sports department, but maybe I want to be a participant rather than a person to organize. This thing, there may still be a misunderstanding of the sports department.

31:09

It is equivalent to a managerial me. I don't like the feeling that it is not the sports department I imagined, and there are some girls in it, some girls and some people who don't like sports very much, and then I back off.

31:25

You are working as a group secretary now, do you think you will retire after this semester this year? It's alright, I shouldn't go back for the time being, but it's really tiring and annoying. There are activities every day, and newsletters are written every day. Well, in fact, in essence, the work of the League Party Secretary and the work of the Student Union should be similar. Um why? Maybe it's because the students will learn that there will be a lot of extra work that has nothing to do with you. The most important motivation, yes, but the words of the League Party Secretary may be that you think you have thought about why my team can still do it, but I can't do it after learning, because I may still have a sense of belonging, I feel that I am our class. One of the story halls is still quite strong. I may work for the class, etc., and I may have no complaints. Then, because I may be with my classmates, but the recognition of the student union may be less, I feel that the student union does not Knowing who is working for, has no job, no direction, no meaning to feeling.

32:31

Now move it on the table there, the chair feels like a labor.

32:36

mean? Will it be the issue of belonging to you just now, and you said that the work of the student union is meaningless, that is, there is no opinion on the work done, because I may be stronger, and then my main task is, if so, which competition in the primary school? When I started, I went to buy water, and after moving, I moved a chair and put things in. There is really nothing to the personal growth of the individual or I don't feel that it is not helpful.

33:10

Do you think the work of the League Branch Secretary is helpful to you? Well, there are still some and because from being the League Branch Secretary, then a class is equivalent to an organizer and manager, and then you and your classmates may communicate more today, Then these relationships with classmates may become more understandable from the bottom, and then the relationship with the intermediary will become better.

33:33

After I joined the team as a sophomore, I had a good improvement in accounting with my classmates after the League Branch Secretary, because I lacked understanding in the freshman year, and there was no formal way, and then when the team became social Learned a lot. So in general, I feel that I may continue to work in the next semester. right. Yes, it should work. This is its content. Just now this teacher asked you about pressure. You said that you were overweight and under pressure in high school. You think it was because of them because you felt pressure. Suddenly he became fat, he became a junior high school student, so he was still similar to the unidentified body shape in junior high school, and then he became fat in high school, because the fat itself became a pressure, because the external image still cares a lot, is a pressure.

34:32

When people are confident, they may also feel that they will succeed in doing things. When they are confident in you, their mentality may also be better, and then they will become better in terms of things. Then, when you are fat, you have no self-confidence. may not be able to do anything at the time.

34:44

Therefore, the impact of external evaluations on you is actually quite large, yes.

34:54

Could this be related to the fact that you will consider employment when you consider a major, and it is also partly because the outside world and some social evaluations from the outside world say that you will still consider occupational status, and this matter will definitely be considered.

35:11

My self-esteem is still quite strong, and I still care a little about these things. He knew about the middle school class, but he didn't go there, yes. right. Is there a sign up? He was eligible if he didn't sign up and didn't sign up. I was eligible, but I didn't sign up, and then I didn't attend. The first 20% of the whole machine ah ah yes. Yes, 2%, 20% is definitely not. Because it should be September when you are reporting to the shareholders of the Times, right? How many days after moving in? It should be during summer vacation. He didn't just start school at that time. When the school started, he had just started school. At that time, you had already been the secretary of the Communist Youth League. At that time, when I was about to have a discussion, it was almost at that stage. You said that your squad leader was in the army.

36:39

The next question is that I just asked a very stressful and difficult question. Yes, is there anything that makes you feel particularly proud? If you have nothing to say about something you are particularly proud of, don’t talk about it. If you don’t think about something that you are particularly proud of, it might be better to talk about something that is a little more gratifying, something you are proud of or a sense of accomplishment, and you do something that you think makes you special. During college, or if you feel that there is nothing in college, I think during high school, I think you can also find things that are particularly proud or fulfilling.

37:28

Shouldn't, anyway, I'm flat. Actually, I'm more flat than calm. There's nothing particularly proud of, or something that makes you particularly happy. Very happy. The core is usually not only learning about your life, but also about learning. There is nothing worthwhile about it. I just said what I just said about pride and accomplishment. Your student union secretary or even if you fall in love, or you and your family Or maybe there is nothing that impresses you more deeply with the people around you.

38:10

Anyway, you usually say pride when you talk about being proud, and you say you are happy when you seem to be short on something. I feel quite a lot of teaching. I usually make jokes with various classmates, and then I usually play on the court and then compete with others. No matter whether I lose or make a profit, I am also very happy. .

38:29

Well, but it seems that there is no such thing as a sense of achievement and pride. I'm actually quite proud of winning the game. But recently, I have won few games, and you have been impressed by whether you have won a particularly beautiful one. Because I still rely on my body to play, and I'm not particularly good at it. And recently, the No. 3 Cup some time ago, and then I won one after 6 games. I was very proud of the first game, and then it disappeared. It was the people who went to the same academy as you, not a No. 3 cup, yes Each college is a unit, and then each college is played.

39:11

Yes, you and your nursing school classmates are correct. Yes, advance. right. In fact, I told you that in addition to learning, your spare time is mainly used for playing or sports, and the other is the two parts of the League Party Secretary.

39:33

Yes, and then I often go out to play with some high school classmates in Nanjing and various attractions in Nanjing.

39:40

This is also a frequent visit, and then you are too tired to play if you can't just play for a day.

39:48

Can you not answer my question do you have a girlfriend? No more not yet. So in fact your spare time is mainly with friends. Yes, with friends, with friends and classmates. You just said that the transfer of majors did not succeed, which will make you more disappointed. Your teacher asked me just now, I didn't remember too clearly, did you solve it yourself? Just disappointed, emotional. Once the ego is digested, the ego should still be one aspect, and then there are parents, how do my parents generally deal with negative emotions? when unhappy.

40:54

When I was unhappy, on the one hand, I communicated with my parents and these friends, and on the other hand, they also comforted me, and then I and my former classmates went to apply for a major change like me, but it was not successful. Yes, and then we communicated with each other. He didn't succeed and didn't succeed. It's a kind of consolation that he didn't succeed. Then, in his spare time, he also went to some recreational activities, such as playing ball, or playing games with classmates in the dormitory, and then slowly It slowly dissipated.

41:27 The

main thing is to communicate with others. If you are bored, it may be slower. You said that when you were a freshman, because you had the goal of changing majors there, you felt that if you didn’t do well in the exam, it might bring you a lot of pressure. In fact, you have not considered the whole thing anymore. Professional, right? The time should have passed. Now if you say that you are academically, do you still put pressure on yourself? Still will?

41:54

Because I don’t care about grades much anymore in my heart and deep down in my heart. I may be more about some knowledge related to your employment in the future. If you are employed in the future, if you don’t have this knowledge, you can’t. Less. right. Then I have already made the grades convenient for you, it is best not to take the course, then this will bring you a lot of trouble in your future life on the one hand. On the other hand, if you do not master these relevant knowledge, it will not be beneficial to your future work. You said, can I say that in fact, compared with your freshman year, your academic pressure during your sophomore year may not be as high as your freshman year.

42:35

Yes, I thought you didn't take your studies so seriously. right. The freshman year is more about the big, and the first is more attention to the grades. Now, it is not necessarily that he does not pay attention to the grades, but I still feel that it is very important to be specific, so that I can meet my graduation requirements, yes. Reaching the necessary time is a basic condition, and then for the future work, then the necessary knowledge for professional knowledge, and the necessary knowledge for school.

43:13

In addition to learning the necessary knowledge, there is not much pressure on academic performance. Yes, but failing a course is the most basic, and failing to fail a course is on the basis of failing a course. I can learn the knowledge and skills that I can need in my future work, and I think it is enough. It doesn't really matter if you get high or not, yes, that's correct. For example, if you look for a nursing job in the future, it is very likely that I will change careers to do something else?

43:45

I think that when you are studying now, you want to learn the skills that you will need to work in the future, but in fact, what you are learning now may still be related to the profession of nursing professional nurse in the future.

44:00

You just said that you might change careers, whether it will be possible or not. Can you say that what you have learned now may not be of great help in the future?

44:11

I said that I didn’t think so. I was just because you may still change careers after you retire from employment. In fact, there is a high probability of change, but you still have to do nursing for a few years. In a few years, you can't make your work very decadent because of the lack of professional knowledge. It must be in the next few years of your nursing work, you already have a good attitude.

44:38 And

then that's for sure.

44:42

I can ask a hypothetical question. Suppose you are in the freshman year, but you still have a lot of academic pressure. You have studied for a long time and learned a skill that you have always wanted. This skill may be a skill in university, or a skill in learning the above experimental operation, or a skill in a certain nursing operation. You have studied for a long time and finally learned it. Another thing is that you have studied for a long time and finally got into the top of the class. Which thing do you think will make you more excited?

45:18

One is to learn a skill, the other is to pass the class situation. It took a long time to get this result. Which thing do you think excites you more? I feel that it is still a channel model skill. I feel that when I was a freshman, there was no need for grade requirements. I didn’t say that kind of special advanced, that kind of professional transfer qualification, when I was a freshman, I only needed one to transfer majors. Qualification will do.

45:47

Isn't the transfer of professional qualifications based on the grades? Yes, he has no hard requirements, that is, you Bohuake is a very hard one, and then if you are Bohuake, you will still have no matter how many names you are, you can apply for some majors, but if your grades are high, you can go to Sign up for other better majors.

46:05

So in fact, for you now, maybe I think it should be more about learning skills, and then learning, but in the freshman year, you may actually think the same as you are now, and you may not have to. Only when you get to the top few in the exam will you feel more excited, and you will have a sense of achievement, yes.

46:37

I don't seem to have anything else. You just said that there is one more thing. You just said that your monitor has been talking for a long time. Will this factor affect a prediction that you may study, that is, you may feel that the school system is too long, and I may not be able to study. Will it feel this way?

47:02

When he was there, he said why for a long time to become one. You study for a long time. If you study for a long time, you are the one or the next one. Some of your social aspects are family aspects, and there are some friends and so on. Others have worked for many years, and they have already considered their careers, and then you are still studying. Maybe these feelings will make you feel uncomfortable. , There are also some family and family reasons. It is impossible for you to study all the time, which may affect your own family. Is this what you mean?

47:41

Still getting married is also a reason, that is, if you don't study, you may study for a long time.

47:45 The

family you are talking about is mainly the family? Right on the one hand. one side of the parent. When you and I meet work, it may be easier for my parents at home. In this regard, the society you are talking about refers to the evaluation of you by the people around you in the society. It is true that others have been working for several years and have succeeded in their careers, and then your children will not feel well when they go to school. So a very long school system is actually a big negative impact on you. Can you say that? You just mentioned several negative impacts. Let me list them again. It is also a long school system, and the employment is not good, or the employment direction is relatively narrow and the employment is not clear.

48:30

Another one is that this may require a more introverted student, someone who is more sedentary to continue learning. Which of the three do you think is the biggest for you, and one is the one that persuades you to quit your job is unclear. Employment is different. If your employment is very bright, it will feel rewarding if you spend a long time or a longer school system, but if your employment is not clear and then you spend a long time studying, it will feel rewarding. The price is still quite high.

49:00

Do you think it takes a long time and needs a person who can sit still. Which of these two points do you think is more unacceptable to you? Is it possible to do a regular school system or a long-term school system? I feel that the price is quite high. If I say that what I am talking about is a hypothetical question, and now the shareholder class has already issued a policy when you change majors, there is a policy saying that the graduation package equipment is the premise that you graduate with a doctorate and stay after the doctorate. School, in fact, at this time, it may be better for you to have a more clear employment and study plan. You may choose them. I may I feel that it is possible to choose him.

49:49

Or because but he may want you to spend a long time, you have to read a book for a long time, you plus one plus 5+1+3 is the best case. No, is this the shortest? In the shortest case, if you do not pass the defense in the sixth grade, it may take longer to study for graduate school.

50:10

I think I might think about him for a while when I am studying for a master's degree. I don't necessarily say that I must go or I must not go, yes, but it will not be like when I was a freshman when I said I would definitely not go.

50:24

Well about the time it's considered for you, are you likely to listen to outside opinions? Will you communicate with the outside world? Sure they will, I generally do they sway your opinion? Do you feel shaken is to recall every time you made a decision, including the report when you took the test in high school and high school, and when you were in college, you still influenced to adopt their opinions and refer to them. Well, there is no problem with other things. Others are no problem. How long have you been talking? talked. right.
