# Supplementary material for: Why do you choose this program?—A decision-making model of medical students based on grounded theory
Source: PLoS One. 2023 Sep 15;18(9):e0291634. doi: 10.1371/journal.pone.0291634 (PMC10503722; doi:10.1371/journal.pone.0291634)
Supplement: S1 File — (ZIP) [file pone.0291634.s001.zip › RAW DATA/P8 CHINESE.docx]

00:01

那么首先要先跟你讲一下我们这次访谈的一些伦理须知。首先本次访谈受访者是在平等自愿的原则上参与的，受访者必须真实的表达自我的想法和认知，确认自己符合受访条件。访谈过程我们会录音，但这个录音资料我们会以匿匿名的方式用于科研，不会泄露给第三方。在访谈过程及访谈结束后，你有权取消我们研究人员录音资料的使用权，你是否知晓并同意？

00:33

知晓并同意嗯，好，我们就开始了。

00:37

首先我刚刚问你的，你是原来讲现在还是基础的，然后之前有报名了国中班对，但是后来取消了是吗？你是几年级的？现在是大四，我是第一届国中班。

00:53

你是第一届国中班，你是已经进入国中班之后取消了，对，大概是待了一个学期左右。大概一个学期然后退出了是吗？我能问问是是因为什么原因退出？可能还是之前一开始宣传的时候，因为是第一届前面也没有人可以了解，然后当时看了宣传材料，也没有想太多就去报名了，然后后来还中了以后，但是进去以后和我自己的因为我是从进学校的那一刻起，我以后是打算去其他学校读研究生的，然后国中班他主要是想为设计学国家重点实验室培养自己的研究生，然后因为我如果回到接触专业的话，我也是可以保研，所以当时是觉得假如说我占了国中的保研名额，然后没有留在国中的话，其实也是不太好的。

01:59

所以我退出播种。

02:02

所以你其实最主要考虑的是你是否能保研是吗？主要是就是保研。

02:08

因为国中班他作为第一届前面有没有人前面也没有人有相关的经验，所以不是很了解他以后的政策是怎么样的，他大概有他当时宣传的时候是说只要挂科，只要过6级都可以保研，但是感觉保研率已经过高了，然后也不是很清楚它到底就是能保本校还是保外校，有没有这样的限制？

02:35

嗯所以你是担心在国中班留下去，会影响你保外校是吗？

02:42

我们先深入的从头开始捋一下，我们最主要的是想要知道你从包括学医，还有加入国中班就一路上的一个心路历程，我们想就从头还原一下，一起回忆一下，首先你是高中的时候填志愿就填的，一开始想学医都填的是医学院。

03:10

其实是这样的，我觉得这是一个比较巧合的事情，我其实高中的时候我是一心想学生物的，嗯就当时就对生物非常感兴趣，然后然后高考没考好，然后当时就觉得嗯好像也就是而且家里人好像也比较觉得比较保守，生物这他们就觉得比较坑的专业，然后后来填的时候也没有往这方面去填嗯，然后当时有正好高考完了以后牙坏了去补就补牙好像是，然后当时牙医正好是我们家亲戚，他就说推荐我去学口腔，然后当时就我第一志愿好像报的是华四川大学的口腔患儿，然后反正前几个，但是前几个都是医学相关的专业，然后但是最后是被南医大基础给录取了，其实一开始当时填的时候我也不知道基础医学是什么有没有去了解过，然后但是觉得基础医学可能和医学还是有点关系，毕竟也有医学这两个字，现在想想当时真的明明是可以去查清楚了，但是其实感觉还是非常巧合，因为到了基础医学以后，发现正好它其实是我一直想要去做的一个事情，它是生物相关的一个专业，刚刚好就是贴合了你的预期，你填哪一大写哪报报哪一大这个专业，就是你前面也填了很多别的医学院，对。

04:45

那些医学院你都有深入了解过吗？然后再写的他们还是说拿着排一排这样子。哪一大是你的第几选择？4第4个选择第4个，然后报的是基础是我的第6个志愿，前面一个是什么？反正我记得报了8口80550，然后后来专业级差又被插下来了。然后后来最后一个是基础，然后因为好像报基础的人也比较少，所以最后就去了基础。

05:21

你填志愿这个过程当中，你父母有没有给你什么影响？主要是我自己看自己填的。你爸爸妈妈就是没有什么意见吗？没有。嗯他们有没有跟你讨论过？

05:35

好像我当时填完以后是跟我们班主任讨论过，父母没有过多参与以前，志愿之前爸爸妈妈没有跟你讨论一下你将来想要干什么，主要当时也不知道，也不是很清楚自己到底要干什么。主要是因为高考本来考得也不是很好，也想看看情况，当时也没有说特别清清楚的职业规划这样子，然后只是因为去看了个牙，然后现在觉得还是非常巧。

06:15

对牙医跟你讲，你可以考虑一下口腔是吗？然后当时就萌生了学医的想法，对。谋生这样的想法主要是基于什么样的考虑？比如说你是有对这方面有兴趣，首先是你对生物有兴趣，吗是的，然后因为有对生物有兴趣，所以想到了医学专业，还是说也考虑到了其他的比如说比方说你有跟班主任讨论班主任也，也有建议你去考虑一下医学专业。

06:49

这好像并没有感觉当时主要是感觉现在大部分人觉得比较好的两个职业，要么是医生，要么是老师。然后当时我高考完了以后，因为我姑姑她是小学老师，当时我考完试以后我就去她小学，就是帮忙，因为真的没有什么事情，在家里很无聊，然后去了小学以后，我发现我真的是不想做教师这个职业，然后就排除了一下。

07:20

也就是说当老师和当医生这两个是在你的观念里面，包括有没有家庭给你的一些观念成分，对，这个应该是就是是一件比较有面子的事情。

07:35

感觉现在一份体面的工作，对应该是这样在老师和医生就是在，你和你的家庭心目当中是两份比较好的工作，这个好主要是因为是因为觉得体面吗？还是说收入还是就业还是？有兴趣。

08:01

感觉现在社会择业的一个标准，应该是工作强度医生肯定大，但是医生的话一个是家里就是家里人以后，假如说身体方面可能比较好照顾这样子，然后教育方面的话，也可能是觉得以后为以后的小孩就是大家更好的基础，因为我们家真的感觉教师特别多，然后可能当时我母亲也是想让我去当教师，但是我体验了一下，发现不是很喜欢，也就是说你父母其实有跟你讨论过，可能是一种潜移默化的影响吧嗯，但是我印象中他们没有说特别特别明确的跟我讲过，就是说你可以去试试看，要不要去考虑一下做个老师嗯，然后你去体验了一下，就发现自己可能不太适合的。

09:06

你产生的我刚刚听你说的有一些现实的因素就是说你要考虑到以后家庭的一些方向，希望给家庭带来一些便利去对吧？然后考虑的要不要学医或者当老师，这两者对家庭而言都是一个比较好的职业，而且社会认可度也比较高。

09:25

社会认可度是吗嗯，也就是说你其实比较在意未来职业的一个实际性的作用，然后社会认可度，其实也就是我们比较传统的经常说的，就是父母这一辈的觉得比较体面的工作，社会地位又高，然后收入也还不错，也比较稳定。对吧嗯应该是。

09:54

你在报考之前，就是你填完志愿，才跟你的父母就是讲让我志愿填好了是吗？没有。我填的过程中他们是一直会看看，但是也不会说过多的干预。基本上是你拿主意，对。你还是比较独立的一种性格是吗？是的，家里面也比较支持你的选择，你选择学医之后，你是通过哪些渠道，哪些途径去了解就是医学院的相关信息？

10:30

记得之前有一个什么APP填报志愿的面向高考生的，对感觉没有特别深入的去了解，主要还是当时基于我的高考分数和他们往年的一个录取名次这样去排的，所以主要是看你的分数来定的是吧？你问一下 App叫什么吗？什么江苏招考非常官方的一个APP，可能是最近才出来的，我是17年高考的，17年我们15年的时候还没有真的没有，我15年还没有听说过。

11:14

你是理科生对吧对？你考上南医大之后，就因为前面几个都没有进了，可就上了哪一大基础，之后一开始有没有失望？

11:25

还好，因为因为当时一开始就是说南医大是可以转专业的，我当时报的时候其实就已经因为我的高考分数其实真的不算特别高，虽然当时打电话的时候，招办老师跟我说我的分数都可以50可以保底可以冲一下80当时，然后就那样停了，但是其实心里是做好准备的。

11:52

可能我来了以后可能是要转专业的，看中哪一大，还有一点觉得他好好转专业是吧？80和50你有做过了解吗？为什么就觉得一定比基础好？不是。因为当时我是不了解基础，当时因为还是一个传统观念，想当医生这个想法，或者说想当口腔医生，进入一基础之后，大概过了多久？你开始转变了自己的想法，觉得基础开始认可基础。

12:32

我大概大一年就是一直处于一个徘徊的状态，因为到了一个我们当时不是建了基础的群，建群以后进去大大部分人问的第一句话就是关于专业的，我感觉我们学校好像很多这样的专业氛围，就是对专业的氛围的，就是想刘对感觉能转的其实大部分人都转了，然后在这样一个大大的趋势下，感觉如果说我留下来会不会是好的？

13:07

但是嗯自己也不是很清楚到底想不想学医，就是但，而且我的兴趣感觉是在基础这一块。然后感觉就是大一就一直在纠结我是应该跟着大家走，还是跟着我自己的内心走。

13:24

嗯所以其实也有受到环境的影响，是的。

13:28

然后后面大概到大一第二个学期期中左右，当时就觉得不转了。然后最后我没有交准专业的申请，辅导员特地问我，我是确定不转吗？你身边有转成功的同学吗，我们班转专业可能很多，对，因为我们基础学的东西是和临床差不多的，然后我们大概20%感觉大概有五六个或者七八个人可以转成功。他们都去哪些专业你知道吗？

14:11

部分就去临床。

14:12

都大部分去临床。

14:15

其实基础和临床还是有一定区别的，主要一个就是比较专心于科研，一个比较搞临床设计的转变其实挺其实其实挺大的。你突然不去做医生，而是专心去做科研了，这跟你之前想要去口腔做医生的想法不是有一点点有一点点背道而驰了，对吧？这个转变你主要是自己的兴趣，就了解基础之后，因为自己的兴趣主要是影响因素，主要是兴趣呢，还是说老师同学们的影响，还是说对职业规划的一个考虑？

15:09

主要应该是兴趣，然后而且当时我们大一的时候就是那个呃解剖老师，他正好他跟我们上课的时候，我感觉他好像是唯一一个就是说觉得基础就是非常棒的一个专业，当时我感觉我身边所有的人都是在都是在说基础不行想要去临床这样子，所以当时可能老师也是给了我一点留在基础的信心。

15:42

是你给你们上课的大部分老师也都会支持你们转去临床是吗？是的，这样子的，对。我们甚至老师都会说你们以后是要转专业的，感觉很多同学他们自己也有这方面的因素，然后父母也有这方面的因素，就是感觉所有的所有的手都在推着他们，就是想转专业，对你怎么没有？你反而因为其实虽然我我当时其实放弃的时候，我父母还是有一点不支持的，但是放弃转专业，你父母其实不支持，对。

16:18

但是我觉得这毕竟是自己的事情，还是更多的想从自己的角度出发，你父母当时不支持的时候是有没有跟你讨论过，我想知道讨论的具体的一些细节。

16:34

他们就是说让我考虑清楚吧嗯他们不支持是因为什么原因你有知道吗？我觉得还是他们不了解基础。因为你觉得是因为他们对基础的了解程度不够，所以可能对基础有点误解，他们觉得基础不好找工作。这样，然后但是然后你接触之后你觉得还蛮好找，工作的时候我是去找了辅导员去聊过。当时然后因为基础一般读到博士出来才能找工作，当时其实我也很担心这个问题，然后我们辅导员跟我说，临床的也有找不到工作的基础的，就是只要做的足够好，不会找不到工作，没有必要去过度担心这些嗯。

17:22

也就是说对你产生比较重要影响的首先是那一位，第一个在你面前认可基础专业上课的老师，然后是你的辅导员给了你职业信心。对。然后再觉得自己职业归职业道路上没有什么太大的阻碍，再加上自己的兴趣，就觉得没有不选择基础的理由了是吗？

17:45

是的，而且当时我们高中的时候当时看过一个视频，我们是闫宁老师讲的开讲，当时有一个就是关于女科学家都去哪了，我当时对我的触动就很大，因为我高中本来就对生物非常感兴趣，然后当我后来决定留在基础以后，我就突然想到了这个事情，我又去把那个视频翻出来看了一遍，感觉更加坚定了。

18:15

可不可以给我讲讲这个视频给你坚定的一些具体的内容，就是为什么这个视频讲了哪些内容有触动到你？

18:27

我现在印象深刻的就是觉得女孩子会容易因为各种事情去放弃就是科研这条路，因为可能觉得家里人说要去找一个工作，他当时我记得非常清楚的是他有一个博士还有几个月就可以毕业了，但是他在那个时候家里人说让他去找工作，然后他就是最后放弃了他的博士学位，嗯就是感觉还是女孩子们不够勇敢去做自己想做的事情，就是会受到大环境当中的很多因素的影响。

19:05

所以基础是你想做的事情，是吗对已经明确了当时父母就是他们考虑到希望你继续整专业，主要是觉得主要是考虑到一个就业的因素是吗？对。然后你跟辅导员聊过之后有跟他们沟通讲过你就是知道的这些事情。我不太记得你是怎么说服他们的，你记得。没关系，不记得就不说。因为我感觉其实就是我一旦做了决定，一般他们也不太容易改变。

19:50

你爸爸妈妈还是比较了解你的性格嗯。补中班你是怎么了解到的呢？当时宣传其实是非常到位的，我们班首先我大一的时候，进实验室其实是在进了国中的实验室，但是当时是因为老师教我们组配的，然后当时叫他们国中实验室非常厉害，于是我就联系老师进去了，然后这个当时国中班又是第一届，然后老师们也推的比较他也比较勤，然后就了解到，而且他在我们班群里好像也发了相关的通知，所以我们每个人都是知道的。

20:39

也就是说宣传还是比较到位的，你觉得对。我记得他当时应该有开宣讲会对吧？对你有的参加了，当时还是沙老师讲的，而且有给你什么触动吗？主要是当时是因为本身我就是在国中班，然后我本身是在国中实验室，不是在国中班，然后你在基础大一的时候就在实验室工作实验室是吧？然后当时又出来国中班，我就是一开始可能也是没有想到后面会发生那么多事情，直接就加入了，而且他宣传的确讲得非常好。

21:25

当时选择国中班，他当时应该就是给你们列出了几个加入国中班之后的一个优势，条件什么的，其中哪一点是最吸引你？最吸引我的可能还是每年1万块钱的奖学金。奖学金吗？当时应该是有什么？我这边比如说有特殊课程，然后你刚刚说奖学金，还有他5+1+3的学制对吧？还有很多我这边还有什么出国交流，对。就在几个条件里面最吸引你的是每年1万块钱奖学金是吗？

22:19

当时应该最直接的是这个因素，然后我记得还有他还说国中班的同学不参与学校的一些活动，因为我可能大一的时候比较懒，就是大一的时候其实参加了很多，这些活动，但是我觉得对我做科研其实是是比较浪费时间的。然后当时就想可能可以让我更加专心做科研，但是其实后来后来但是那些活动也不是必要参加的，如果你有很多尝试这样。

22:57

对。然后但是后来其实感觉尤其是现在年年纪越来越高以后，然后其实很多当时强制参加的活动还是非常有必要去参加的，是因为按照我们当时的一个见解，可能不是特别愿意去，但是对我们以后的发展还是非常有用的。

23:17

比如说某一些宣讲会这种，他会要求我们辅导员会要求我们低年级的也去参加，我中专的宣讲会是必须强制性，不是觉得他会有其他学校的宣讲会过来。也就是说通过这些渠道，你信息获取对会对你的就是未来的一些选择都比较有利。了解到国中班的一些信息之后，你觉得最幸运的点是奖学金是吗？然后你就报名了吗？而且当时因为我在本身在国中实验室，然后老师感觉也是比较他当时还问我有没有报名，然后可能他也是比较希望我去报名的，然后感觉其他的我当时就没有考虑那么多，就报名了没有考虑那么多，主要考虑的就是反正有钱先去，然后他其实是中途你不愿意是不是还可以退出的？

24:28

对，他当时我们进的时候他就是这么说的，所以觉得反正入股不亏，你觉得对你产生最大影响的是是什么因素？就是加入过中班加入过中班。主要还是就是想嗯。把大部分的精力就放在因为我大一的时候，那时候老师他已经给了我一个小课题了，我就想把后面的精力主要就放在科研上面。我当时觉得国中班是可以给我这样的一个环境，让我就是安心做科研的实验室老师。

25:14

对你的影响大吗？他对我也挺好的。而且他的一些思维其实对我现在都是有很大的影响的。所以你还是是因为有没有因为这个老师比较因为这个老师更想加入国中班的，对这个是有的。有通过老师的形象就各方面就觉得对国中班印象加深了就好感加深了。是有的是吧？加入国中班之后跟你想的有什么出入，符合你的预期吗？但是我觉得大部分其实还好，但是主要有一点好像是当时好像就是进了国中班以后，就是又归学工办管，又归国政管，就感觉反而杂事更加多了一点，这样对。

26:16

大一的时候参加什么社团或者院会学生会。对，有参加哪个？我当时又在校会又在约会。两个都参加了dhv，在相互的什么部门科协院会是文娱部很忙吗？是挺忙的，事情比较多。然后我大一的时候在实验室主要就是周末去听听组会也，没有说花很多时间去接触一个课题，但是我知道假如说真的我要去自己做一个课题的话，那些时间我肯定是要腾出来的。

26:59

你但在校会和学生会你有没有觉得自己学到什么东西？嗯有我觉得对我的一个思维是很大的提升，尤其是我大一的时候，我大一就进实验室，很大程度上是因为我带着学生会，我那个时候大概我第一个参与的事情，科研兴趣小组的一个选拔，当时我就发现真的大家非常优秀，然后我不进学生会，我是不会了解到这么多优秀的人，然后不会跟他们有所接触，也不会说就在我大一就就，在我的同学们都在单纯的学习，或者说上课或者在宿舍待着打游戏的时候，我自己去见习导师进实验室，嗯所以就是学生会和院会，其实甚至是把你往科研的道路上更推了一把对，那就是加入国中班之后啊。

28:03

在学习的过程当中，除了你刚刚说的就是他管理上的有一个跟你想的不太一样，其他方面有跟你比如说科研做课题什么的，有符合你的预期吗？他当时是要求要轮转，然后除了在我一开始在老师实验室，我还去了，其他老师实验室就是感觉多了解一些东西。

28:33

跟你本来想的一样吗？本来你觉得进入股东班之后应该有一个更好的环境做科研嗯，对，这个也是有的，还是符合你的预期的是吧？加入股东班之后有没有什么发生过什么让你印象特别深刻的事情？国政班他们我记得我们当时好像国众他会自己组织一些非常有意思的活动，就是增加老师和学生之间的一个联系。

29:15

因为当时好像他因为他经费比较充足，他有一次弄了一个什么包饺子比赛，然后好像一系列的活动反正还是非常有意思的。这是我第一次以这样的一个形式去和老师相处嗯在社团还有学生会没有过这样的经历，社团学生会感觉学生和老师的还是比较有阶级差异还是比较有距离，感对在国中班的话就是让你体验了一下，跟老师的关系感觉更亲近了是吗？有没有发生过什么让你特别失望的一件事？

30:03

然后什么当时我们好像是那个时候就有一些课程，中国中班是会给我们砍掉的，是因为他要给我们加新的课程，然后加科研的轮转，他也是算学时的。

30:20

然后当时主要是一个是就是因为我们在不同的实验室轮转，是由导师给我们打分，也算主干课，然后感觉各个老师之间的打分的标准是有非常大的差别的。感觉有的同学他们的老师打分就会高得非常离谱，可能觉得自己在觉得自己的表现会比他们好，但是分数却没有他们高。

30:51

然后后来的话，他们砍掉了高级生化，我是觉得这是一门非常重要的课，当他们说要把内科外科也要砍掉，但是我觉得对我们以后是还是比较有用的，我是想争取把这些课给我们留下来，然后跟老师也谈讨论过好几次，但是感觉最后也没有什么应答，后来还是砍掉了，后来我就出来了。

31:20

但是最后他们好像这些课还是上了高级生活好像没有。应该还是上了。这个跟你这就这个课程设置跟你的预期有点不太一样，然后有很多你觉得有意义的课。但是被老师删掉了是吗？对。是导致你想想要离开国中班的一个原因。对，也是一个原因。这应该是最直接的因素，因为当时这个事情我们跟老师提过好几次，但是其实就是国中班内部也有一个矛盾在这儿，因为有的同学觉得课少了以后会轻松一点，他们也不是不想加课，但是从我的角度来看，尤其是高级神话，它里面讲到很多分子生物学相关的，就是因为我们基础大二的时候生化是分两部分上的，前一部分是生物化学，然后一部分子生物学，但是和我们实验相关的很多是在后一部分上的，假如说我们没有上这个课，很多理论知识我们相当于只是在做实验，而不是去更加深入的去了解。

32:33

我是觉得我们虽然也可以说通过自学去弥补这一方面，但是感觉还是非常需要老师系统的讲解，然后这是最直接的因素。

32:47

对。再等等其他的因素，就是你是大姐离开国中吗？二大二。刚加进去，我是在那里面待了一个学期，第二个学期，开学以后没多久我就离开了那，这个就是你刚刚说了一个课程设置是最直接的原因，然后你一开始的时候我就问了，然后你当时说了一个就是可能会影响你保外销是吗？还有没有别的原因？

33:23

你们大二的时候就已经开始研究就是保研的事项了，不是这个得问清楚，不然到后面那不是就是来不及了。当时好像不光我再问，好像还有好几个同学也在问这个事情，但是一直没有比较明确的答复嗯。你想保的外校是哪个学校？北大。当时有没有跟有老师讨论过就是你关于保研的一些想法？跟国中班的老师有没有跟他们讨论过，你就是想要离开过什么班？

34:10

我们当时是和国中班就是管理的老师有讨论过，然后管理的老师也上去，和上面的领导也在讨论，当时除了我以外，还有很多同学有这样一个想法，但是后来他们给的答复大概国中班主要还是为国中培养研究生的，研究生还是要留在锅中的，但是具体到底怎么样他们也没有明说，但是你研究生其实不太想留在锅中。

34:42

对。留在国中是完全不能接受的是吗？研究生。我大概在我决定留在基础那一刻，我也差不多决定了我已经想好了，我以后大概这条路该怎么走。你的规划其实已经很明朗了，你有没有跟身边的同学还是有好朋友们聊过，就是国中班从你还没有加入到加入后想离开？应该有讲过。但是因为我们宿舍主要就是我们宿舍只有我一个人去了活动，嗯但是感觉他们主要还是就是一个倾听的。

35:34

主要是听你讲什么，不会对你产生什么影响，对。你有跟父母讨论过吗？你觉得他们还是因为他们也不是很了解，然后当时我说我要退国中班的时候，老师还问我说有没有问我的父母是什么反应，但是当时的确他们也不是很了解，就是退国中班和不退国中班对我以后有什么样的影响，然后最主要还是我自己做的决定你在选择离开国中班的时候，我可不可以理解为就是你考虑的最主要的因素，一个是你的职业规划，就是你的学校应该是算学业规划，比如本科生本科我要在哪里读，研究生我要在哪里读，影响到了你规划嗯，然后还有他学的东西，课程的一个设置是一个导火索。

36:35

对，主要就是这两个因素，而且当时这个事情因为我们一直在跟管理的老师沟通，但是感觉一直都没有得到解决，然后后来老师感觉我们提问题好像也得不到解决，就是因为有这种感觉，因为得不到回应，所以加深了一个这样的事有回应，但是回应就是不会给我们加这个课，得不到满意的回去。后来甚至我自己去找了教务的老师，当时好像也说，当时我不太记得他的回复了，但是我感觉国中老师好像并没有把我们的想法往上传达，他们自己跟我们说不能这样。

37:27

你觉得国中班的老师们没有为你们去帮你们解决这个问题是吗？就都没有试图帮你们解决，对，我当时是这个感觉。这个有让你产生很大的失望。在国中班期间有没有发生过什么让你觉得特别满意的事情？就对国中班最满意的点有没有？可能让因为我们大概轮转完了以后，每次轮转完都有一个汇报，我觉得汇报的话可能是让我们不光可以了解我们同学们学的怎么样，然后我们去了解其他的实验室是大概一个什么样的环境，我觉得这个是做得非常好。

38:14

嗯那有没有什么让你特别骄傲的事情？你自己做的让你自己非常骄傲的一件事情，在国中班或者大一或者整个你的现在本科期间，我可能还是在老师实验室。当时活动实验室对。他是呃，老师我学了实验技术以后，当时老师对我是非常满意的，他甚至说让我做一个实验，就是实验成果的一个汇报跟他讲，因为当时是他们研一新生进来以后进行一段时间学习或者有这样一个汇报，我是大概自己花了一个多月的时间，就把这些实验全部做完，然后整理成图表去跟老师汇报。

39:07

嗯我觉得这个是一个非常有成就感的事情。这是大几大一大一。一不对，应该是大二上学期。这个时候加入国中，好像加入了。成就感有没有让你觉得啊留在国中班是蛮好的。

39:35

主要是让我觉得让我觉得我可能还是比较适合做实验做科研嗯。因为感觉我觉得我是非常喜欢老师对我的培养方式的嗯然后他其实也非常重视，然后当时给我一个课题，让我师兄带着我做，然后他也会主动和我们一起讨论这个课题吗？但是后来觉得还是权衡利弊以后，因为当时我其实就是退出的时候也很纠结，因为老师对我也很好，因为师兄师姐对我也很好，我觉得退出了以后他们会不会以后我也不太想带本科生，会有这样的一个顾虑。

40:26

你还是坚定的选择特殊的终端。当时的顾虑你提到了一个可能会造成老师们会不太敢说，本科生我们大家就走了。还有没有其他的顾虑，就是让你犹豫，要不要走？还有我当时手头那个课题，大概也做了一段时间了，也没有做完，我走的话这个课题相当于是就废在这里，其实也是比较熟的。

41:02

你后来又把课题做完了没有？没有，对，留给师兄师姐们，对，后来我师兄毕业的时候好像本来打算做，但是他也没有来得及做。但那个课题主要还是就是偏数据分析一点，如果是实验的话，可能影响会更大一点偏。

41:26

课题分析是吗？

41:29

当时你走了之后要决决定，要走的时候，你的老师们和师兄师姐们有跟你讲什么，你们有聊聊天或者送给别人什么的，当时说师姐他们是跟我说，因为我走了以后的话，他们其实做实验会少一个人一起帮忙，但是师姐她当时也是就是说还是让我按照自己的想法去什么样对我的发展更好，我觉得他们还是对我非常好。

42:08

让我嗯走一条更适合我的路，他们还是比较支持你去追逐自己的理想对。

42:19

当时你有没有跟他们提到你当时的一些顾虑或者是实验什么？还有会不会你们以后就不太愿意招本科生了？有没有问过他们的想法，还是说你决定了之后，你在做决定之前有没有跟他们讨论过？我可能想走我当时好像没有讲到这么深入，稍微讲了一下大概的情况，他们有没有劝你留下来？

42:55

没有，对，因为师姐她也是比较尊重我的感觉，我也跟他跟他讲了一下，因为当时我有一段徘徊期的时候，当时正好我们在上生化课，然后我们生化老师他那边正好有个大众团队，我看他们只有三个人，然后我就去联系了他，我说我想跟他们一起做大创，因为我在国重做的课题是主要偏生性，但是我其实从基础方面来说，其实最主要还是做实验的，我觉得当时没有一个实验的这样一个课题，我是想要从其他方面去弥补一下。

43:42

嗯然后后来在那边实验室待了以后，我发现那边的实验室我去了以后是老师他没有让我做大创的课题，他反而给我另一个课题做而且当时我上面有个师兄，他也是本科生，自己也差不多完整的做了一个课题，我觉得我看到了一个我可以走的这条路，然后有让你我对本科生做科研有了更多的信心。

44:20

你是有参加大创还有参加挑战杯什么的？

44:28

当时是跟国中班的老师吗？没有跟我现在的老师我，现在的老师你是大几的时候参加大创？大二的时候参加过。

44:41

时候不是在国中班吗？我大二下学期不就出来了，出来之后有再参加一个大创的课题，有感觉学到东西对自己做课题和跟师兄师姐做是完全不一样的，就在自己会遇到各种问题，然后自己去想办法解决。都可以谈一下具体的比如说有让你印象最深刻的在做大创。

45:10

我记得当时考试月的时候，大概我们一个礼拜考一门，大概是这样一个频率，然后当时我老师他有一篇文章正在修回，然后他就让我帮他就是染片子染明眼光，但是需要染好几个好几种抗体，然后其他的都可以染的很好，但是我做的目标蛋白就很奇怪，就染不出来。

45:42

大概重复了好几次，就一直效果都不是很好，然后当时也觉得其实挺难受的，尤其是在考试的时候，大家在复习我在那做实验，然后当时我还记得我甚至一些资料我复印两份，一份就是可以戴着手套看的那种，然后但是最后非常巧的是有一次有一管抗体，它里面不太多了，我大概加了那么多，PPS进行稀释，而且那次是可以染出来的。

46:15

然后后来发现原来是抗体可能是放的时间有点久了，效价降低了，然后重新摸一个浓度梯度，是这样一个原因导致没有染出来，他试了好多因素，从脱水脱落的问题，可能然后抗原修复，也有可能有问题，当时当时就是因为因为其实流程的话还是比较当时还是觉得流程是比较多的，就是要一步一步找哪里出了问题，就找了好几次都没有什么好转。

46:53

感觉这个事情让你有一个什么样的感受？像我觉得做科研这其实真的不是说特别容易的事情，然后但是可能又在某一些机缘巧合之下就可以解决它。

47:13

但是也是要不断尝试，你觉得是什么事情？

47:19

有没有什么具体的事情让你非常坚定的选择了要走科研这条路，我们应该最主要是当我决定说不转专业的时候，那个时候我自己主动放弃这一次机会，我后面的道路大概就是这条路了。

47:50

嗯那有没有什么事情让你特别认可做科研，因为你本来是想要去做临床的，然后转变到要去做科研，其实是两个就是不太性质不太同的事情。

48:11

我们可能说讲到做医生，我是要去治病救人，那也可能是我考虑到职业它相当于是它不是那种非常静态的，它是一个很动态很多变的，然后做医生的话，包括个人的性格，那么职业的就业的影响因素，比如说医生的他的收入还有包括什么的，跟科研相比较，你是有考虑到这些因素吗？

48:49

嗯就是其实做科研虽然说他时间比较自由，但是他的收入是真的没有医生高的。然后可能精神压力也会比较大，因为经常要写标书申请经费这样子。但是我当时可能还有一个因素，就是有很多疾病虽然说临床上这么治那样治，但是还是有很多疾病它是没有治疗的方法的。感觉现在还是觉得医疗还是有一些瓶颈需要去突破，但这些事情就光靠临床医生是是不够的，还是需要基础科研去进一步的完善巩固。

49:40

你是什么时候产生这样的想法？

49:45

觉得有些问题是需要我科研人员去解决的，可能和我那个课题有有点关系，因为我做我做的蛋白它是一个罕见病相关的一个基因，然后尤其是这种罕见病，它的不太会有公司去研发药物去治疗它，因为就算他花了很大的成本去研发出的药物，但是它作为罕见病，它的就是受体是非常少的。

50:23

我当时觉得一个是觉得可能感觉是一种社会问题，然后做罕见病药物的一个研究，有给你带来一些冲击什么对。

50:43

你有有有跟朋友们讨论过，你想要做科研的初心吗？

50:50

嗯或者、朋友、老师，好像跟我现在老师讨论过，当时我为什么嗯因为我去找他的时候，我就跟他说我就想做科研，然后所以我感觉他因为我的这样一个想法，所以才在认识我没多久，那时候他就给我这样一个课题让我去做。

51:16

你寒假病的课题是什么时候接受的？

51:22

也是在我大二的时候就是大二大概是在退国中班前后是什么老师给你提供了这样一个课题，是我们当时的生化老师，生化老师是国助班的老师或者是生化系的基础的老师，然后你跟着做了这个课题之后，更加坚定了你要做科研的一个决心是吗？离开国中班之后，有回想过离开国中班这个事情有没有觉得后悔过？

52:08

没有，但是当时其实是心理压力非常大的，因为我是第一个就是我离开国中的同学嘛，我当时也不知道要怎么弄然后，大概有一段时间一直在和国中班的领导，然后基础医学院的领导，然后教务部的领导在这边到处跑，自己在这边打交道然后最后，学籍也回去了，然后甚至当时老师说要让我补修一门检索课，嗯就是，然后当时好像因为辅导员说我好像少了一个学时，让我补休以后可以参加这一学年奖学金的评比，然后但是当时我们班的课其实是非常满的，然后后来安排呃检索课我是也没有说有非常合适的时间去，我后来实在没有办法检索，好像是三节课，我只能上前两节，我第三节课我得去上我自己的课，就是这样。

53:22

当时刚刚讲到压力，当时你有面临过压力很大的一段时间，主要是因为从国中班回到基础的这个过程当中就有很多要处理的事情，比如说你刚刚说的要补学时，这是一方面的压力，还有没有其他的压力？

53:46

我觉得最主要的是觉得其实和那么多领导打交道，自己心里是其实还是感觉挺有压力的，而且国中班他就是成立的第一年，然后我就退出了，我其实觉得是对国中办也是不太好吧。

54:06

然后甚至我在路上碰到过中班的老师，我也想绕着走，一时有什么不好，我感觉从我的角度来讲，可能是一种不认可，但是我其实是非常喜欢国中班的老师，但是因为我自己的一些因素导致了退出以后，我会觉得有点就是就是，有点那种愧对于他这种感觉。

54:38

你觉得离开国中班，你离开国中班的原因，其实是因为你自己的因素，并不是因为觉得对，主要还是我自己的因素，对。你觉得如果有哪些条件在符合的情况下，你会愿意继续留在国中吧？这个应该不会。都不会对，因为我觉得做科研还是我其实还是想去更好的平台做科研。我就打个比方，比如说他允许保外销，就算他允许保外销，我觉得我占用了国中4年的名额，然后走了我觉得是更加不好。

55:28

你就是离开国中班之后，就到现在都大四了，有没有在跟股东班老师合作过？我这个课题其实是我的老师和沙老师有一些合作，但是我个人方面没有过，但是后来路上碰到以后还是会比较开心的打招呼，然后不能说之前也会感觉大家还是非常和善。那么离开国中班之后的学习有在符合你预期当中的进行，对。

56:08

还有你保卫校现在这个事情在知道目前在准备夏令营暑假的时候去的时候，离开国中班之后就是做其他的课题，跟着老师做，你是觉得都是符合你预期的吗？有没有遇到过什么困难？

56:36

有就是因为，我现在老师他是一个讲师，他没有研究生，虽然我上面有个师兄也是本科生，然后但是有很多我要做的东西，其实他也是不会的，我们现在实验室它是分各个小组，我上面是没有直线的世界，但是我在以前我中的时候就是做什么事情都是有师兄师姐带着我的，然后到这边以后感觉只要自己独当一面了，其实还是非常害怕，然后但是其他组的师兄、师姐感觉、人还是非常不错的，我们去问他们的时候，他们还是非常愿意跟我们讲，有没有有没有应对过？

57:22

有什么竞争方面的关系在吗？小组之间，对。可能每个小组的小老师之间会有一些竞争吗？你有没有应对过？那是我们本科生的话其实还好，他们不太会把矛头放到我们身上。那就是在大三大四这段时间的学习，你觉得有发生一些什么改变吗？跟之前相比。

58:02

我觉得我的话是做科研的一个能力是有提高的，因为很多事情没有人带着我去做的话，我要自己想办法我要看着文献，或者说对这一份不是脱口，我就可以完全的把它自己做出来，感觉越来越独立了。

58:21

自己做科研，自己在忙碌的时候，如果遇到一些就觉得自己不太能解决的问题，你主要是求助老师还是说你是没有上面没有学长学姐在带你，对。

58:41

但是我有一个小老师主要是跟他讨论交流，对，我会和他讨论，我也会和其他组的直接讨论，他有没有跟你聊过国中班当时。

58:52

我关于退过中班的事情我也问过他，然后他你有没有跟他讲过，你是考你考虑到哪些因素要退步中吧？

59:03

对我好像当时退的时候，我还写了一个画了一个表格，说留在国中班的原因和退国中班的原因，但是具体的东西我已经不太记得了，这个表格还在吗？

59:15

这个应该没有了。

59:17

能大概讲一讲吗留在国中班的原因和退国中的原因，我就不太记得了，但是当时我记得那个老师他就看了我那个表格，我给他看了，然后他就说退口中班这么挑，原因这么多条就是因素支持我推广状态，当时我这么一说，我其实内心就有点清楚了，可能我内心还是更想说出来，因为感觉留在国中班也像是一个舒适圈，其实跳出来还是需要进行一些思想斗争。

59:57

有没有学弟学妹们来问过你说学姐我想要去浦东班？有。他们你你当时是怎么跟他们讲的？我会大概介绍一下啊，但是我尽量不把我自己的一些主观情，我会尽量讲的比较客观一点，最终还是让他们自己去做决定。

01:00:24

你比较你推荐他们去吗？你就是在考虑要不要推荐他们去的时候，主要是想到哪几点？

01:00:34

我觉得对于本科生来说，假如说能够在其实我们的确有同学他们国中班的现在已经找到了很好的导师，研究生就打算跟着他这样做的，其实是非常有优势的，就是他现在跟这个老师大概可以提前4年，或者说至少3年就开始做他研究生的课题。对，他以后就相当于节省了三年的时间。而且他已经融入了这个实验室对他以后的研究生期间的一个发展也是非常有利的。

01:01:10

所以你觉得还是假如说本科能够和以后连贯起来的话是很好的，就没有必要再去过这个班了是吗？国政班。看能不能在国中班找到这样一个老师，最主要还是看老师。

01:01:30

我的意思是嗯，比如说大一的学弟学妹们，他们还没有进入补充班的，然后如果他们来咨询你你，主要是考虑他们会不会推荐他们来补中班。

01:01:49

你刚刚讲的是如果他们已经处在一个我是说他们可以在国中没找到这样的老师，从因为博士生班不是可以直接，他们不是鼓励在国中读研，可以连着做我就跟他们讲有这样的一个优势在这。所以其实是建议他们如果有这样的想法，有想做科研的想法是可以加入我们班的。

01:02:16

你是觉得主要是要看他们有没有做科研的心是吗嗯是的？你在国中班的时候，这个是你是在国中班的时候会有学弟学妹来问你是吗？你退了之后还会主要还是我退出来以后，因为我在国中班也就待了一个学期，当时问我的人还是比较少的。

01:02:42

后来我感觉就是你退出来之后，他们有没有来问你你怎么退了，你为什么要退，对也会有人问我怎么退，然后总会你问你怎么特别对他们。是为什么？可能也会有和我差不多的情况，然后当时我们班有另一个同学，他是因为想做免疫的课题，然后国中是要求他只能做国中的课题，他也是退了国中班，但是他还是在他原来老师那边他想做的课题，所以其实你们主要考虑的还是比较自己的兴趣，对。

01:03:20

你在国中班就退了之后，有同学要问你你为什么要退？你是怎么说的呢？主要我应该一般就是说个人兴趣因素，他们如果问你啊我要不要加入国中班会不会有什么不好的地方，比如说你之前说的课程设置什么那些，我不会说。

01:03:50

因为我觉得其实国中班它其实还是非常好的一个设置本身，而且我们作为第一届肯定问题是比较多的，那么后面应该会不断的去解决这个问题。但是我那个时候已经相当于是比较脱离过这种班了，我不会再用我那个时候见到的国中班去评判现在的国中班，我不会把我当时遇到的问题再跟他们讲，整体来讲你对博士班印象怎么样，我觉得还是非常不错的。

01:04:19

哪方面不错？

01:04:19

其实我觉得他们对学生的一个培养的一个模式，5+1+3对啊，而且他们就是科研轮转汇报还是非常系统的，让学生他当然是比大家学同学们散着，在各个实验室也没有老师管，你想去就去，不想去就不去，他其实可能我觉得对从一个小白往上走，其实是非常有利的嗯。

01:05:13

好像就没有什么太多的问题。你大概是从什么时候开始觉得基本上父母已经不做你的主了？

01:05:26

基本上都是自己拿主意嗯。

01:05:30

我觉得进大学以后好像就是这样，进大学的时候比较明显，今天填志愿的时候已经开始了，对，主要他们也不是很了解。然后我当时自己在家，反正高考完了也没什么事情，会去找各种资料看找各种资料找主要是你刚刚讲了一个APP，除了APP以外，你还会通过哪些途径去了解？

01:06:00

我好像当时是在百度还是哪，就找了一下我，忘了你的分数对应的排名，然后因为感觉看分数的话就是波动太大了，没有什么参考意义，但是就是往年的排名就没有人能够给我们整理好，然后我就去自己整理好。我们今天就先到这里，对。
