# Supplementary material for: Why do you choose this program?—A decision-making model of medical students based on grounded theory
Source: PLoS One. 2023 Sep 15;18(9):e0291634. doi: 10.1371/journal.pone.0291634 (PMC10503722; doi:10.1371/journal.pone.0291634)
Supplement: S1 File — (ZIP) [file pone.0291634.s001.zip › RAW DATA/P8.docx]

00:01

So first of all, let me tell you some ethical considerations for our interview. First of all, the interviewees participated in the interview on the principle of equality and voluntariness. The interviewees must truthfully express their thoughts and cognitions and confirm that they meet the interview conditions. We will record the interview process, but this recording will be used for scientific research in an anonymous way and will not be disclosed to third parties. During the interview process and after the interview, you have the right to cancel the right to use the recording materials of our researchers. Do you know and agree?

00:33

Know and agree Well, well, here we go.

00:37

First of all, I just asked you, you originally said that it is still basic, and then you signed up for the middle school class before, but then canceled it? What grade are you in? I'm a senior now, and I'm in the first middle school class.

00:53

You are in the first junior high school class. You canceled it after you entered the junior high school class. Yes, you probably stayed for about a semester. About a semester and then dropped out? Can I ask what was the reason for the withdrawal? Maybe it was when the promotion started before, because it was the first session and no one could understand it. Then I read the promotional materials and signed up without thinking too much. After that, I was awarded, but after I entered, I signed up for it. My own because from the moment I entered the school, I planned to go to other schools for postgraduate studies in the future, and then in the middle school class, he mainly wanted to train his own postgraduate students for the State Key Laboratory of Design, and then because if I returned to If I get into a major, I can also be guaranteed a postgraduate study, so at that time I thought that it would not be very good if I occupied a postgraduate place in a junior high school and then did not stay in a junior high school.

01:59

So I quit sowing.

02:02

So your main consideration is whether you can guarantee research, right? Mainly research.

02:08

Because he was the first class in the middle school class, did anyone in front of him have relevant experience, so he didn't know very well what his future policies would be. Department, as long as you pass the 6th grade, you can guarantee the research, but I feel that the rate of guaranteeing the research is too high, and it is not very clear whether it can guarantee the main school or the foreign school. Is there such a limit?

02:35

Well, so you are worried that staying in the middle school class will affect your bail out of the school, right?

02:42

Let’s start from scratch. The main thing we want to know is your mental journey from studying medicine to joining the junior high school. We want to restore it from scratch and recall it together. , First of all, you fill in the volunteers when you are in high school. At the beginning, if you want to study medicine, you fill in the medical school.

03:10

That's actually the case. I think it's a coincidence. In fact, when I was in high school, I was all about biology. Well, I was very interested in biology at that time. Then I failed the college entrance examination. I just thought it was the same, and my family seemed to think it was more conservative. Biology was considered a more pitiful major, and then they didn't fill in this area when filling in, and then there was a broken tooth after the college entrance examination. Filling my teeth seems to be the case, and then the dentist happened to be a relative of our family at that time, and he said that he recommended me to study oral cavity, and then it seemed that my first choice was to apply for the oral child of HuaSichuan University, and then anyway, the first few, But the first few were all medical-related majors, and then I was accepted by the Foundation of Southern Medical University. In fact, I didn’t know what basic medicine was when I filled it in at the beginning. I didn’t know it, but I felt that basic medicine might be possible. It has something to do with medicine. After all, there is also the word medicine. Now that I think about it at the time, it was obvious that I could check it out, but it actually felt like a coincidence, because after I got to basic medicine, I found that it was actually what I always wanted. One thing to do, it is a biology-related major, and it just fits your expectations. Which major you fill in should be reported to which major, that is, you have filled in many other medical schools in front of you. right.

04:45

Do you have a deep understanding of those medical schools? Then when they wrote it again, they still said that they were holding a row in a row like this. Which major is your first choice? 4The 4th choice is the 4th, and then the basis is my 6th choice, what is the first one? Anyway, I remember reporting 8 ports of 80550, and then the professional grade was plugged in again. Then the last one was the foundation, and then because there seemed to be fewer people reporting for the foundation, so I finally went to the foundation.

05:21

Did your parents have any influence on you during your volunteering process? Mainly I fill it out by myself. Do your parents just have no opinion? No. Well have they discussed it with you?

05:35

It seems that I discussed it with our head teacher after filling it out. Before my parents participated too much, my parents didn't discuss with you what you want to do in the future before volunteering. Mainly, I didn't know it at the time, and I didn't know exactly what I was doing. what to do. The main reason is that the college entrance examination was not very good, and I wanted to see the situation. At that time, I didn't say a clear career plan. Then it was just because I went to see a tooth, and now I think it is very coincidental.

06:15

Tell the dentist, can you think about the oral cavity? Then the idea of studying medicine came into being, yes. What kind of considerations are mainly based on the idea of making a living? For example, are you interested in this aspect, first of all, you are interested in biology, yes, and then because you are interested in biology, you think of medicine, or you also consider other things. For example, you have Discuss with the class teacher and also suggest that you consider a medical major.

06:49

It doesn't seem like I felt that at the time, I mainly felt that there are two occupations that most people think are better, either a doctor or a teacher. Then after I finished the college entrance examination, because my aunt was an elementary school teacher, I went to her elementary school after I finished the exam, just to help, because there was really nothing to do, and it was boring at home, and after I went to elementary school, I found that I really didn't want to be a teacher, so I ruled it out.

07:20

In other words, being a teacher and being a doctor are two things in your concept, including whether there are some concept elements given to you by your family. Yes, this should be a relatively face-saving thing.

07:35

I feel like I have a decent job right now. It should be like this. Teachers and doctors are there. You and your family have two better jobs. Is this good job mainly because you feel decent? Or is it income or employment? interested in.

08:01

I feel that one of the criteria for choosing a career in society today should be that the work intensity of the doctor must be high, but the doctor's word is that one is at home and after the family, if it is said that it may be better to take care of it in terms of health, and then in terms of education, it may also feel that In the future, it will be a better foundation for everyone in the future, because our family really feels that there are many teachers, and maybe my mother also wanted me to be a teacher at that time, but I experienced it and found that I didn’t like it very much, that is to say, you Your parents have actually discussed it with you, maybe it's a subtle influence, but I don't think they told me that it was particularly clear, that is to say, you can try it out and consider whether you want to be a teacher. Well, then you go to experience it, and you find that you may not be suitable.

09:06

I just heard from you that there are some realistic factors, that is, you have to consider some directions of the family in the future, and hope to bring some convenience to the family, right? Then consider whether to study medicine or become a teacher, both of which are good careers for the family, and the social recognition is also relatively high.

09:25

Is the social recognition degree? Well, that means you are actually more concerned about a practical role of your future career, and then the social recognition degree, in fact, is what we often say in a more traditional way, that is, the parents’ generation feels more Decent work, high social status, and then a decent and stable income. Right, it should be.

09:54

Before you apply for the exam, you are the one who fills out the application, and you tell your parents that I will fill in the application, right? No. They will always take a look during the process of filling in, but they will not say too much intervention. Basically you make up your mind, yes. Are you still quite independent? Yes, your family is also more supportive of your choice. After you choose to study medicine, which channels and channels do you use to learn about medical school?

10:30

I remember that there was an APP that was for college candidates to fill in the application. I didn't have a deep understanding of the feeling. It was mainly based on my college entrance examination score and one of their admission rankings in previous years. Is your score determined? Do you ask what the app is called? What is a very official app for Jiangsu entrance examination, which may have only come out recently. I took the college entrance examination in 2017. In 2017, when we were in 2015, there was really no one. I haven't heard of it in 15 years.

11:14

You're a science student, right? After you were admitted to Southern Medical University, because you didn't get into the previous ones, which major foundation did you get into, did you feel disappointed at the beginning?

11:25

is not bad, because it was said from the beginning that Southern Medical University can transfer majors. When I applied at the time, it was already because my college entrance examination score was not really very high, although when I called at that time, recruited The teacher in the office told me that my score could be 50, and I could guarantee the bottom line and I could reach 80 at that time, and then I stopped like that, but in fact, I was prepared.

11:52

Maybe after I come, I may want to change majors. Which major is my favorite, and I think he should change majors well, right? Have you ever done 80 and 50? Why do you think it must be better than the basics? no. Because I didn't understand the basics at that time, and because it was still a traditional concept, I wanted to be a doctor, or I wanted to be a dentist. After entering the basics, how long did it take? You start to change your mind and feel that the basics are starting to recognize the basics.

12:32

I was in a lingering state for about a year in my freshman year, because I arrived at a group where we didn’t build a foundation at that time. After the group was established, the first question most people asked was about majors. I feel that our school It seems that many of these professional atmospheres are for the professional atmosphere, just want Liu to feel that he can switch, in fact, most people have switched, and then under such a big trend, I feel that it would be good for me to stay. of?

13:07

But um, I don't know whether I want to study medicine or not, but, and my interest seems to be in the basics. Then I feel that I have been entangled in my freshman year whether I should follow everyone else or follow my own heart.

13:24

Well, so it's actually influenced by the environment, yes.

13:28

and then around the middle of the second semester of the freshman year, I felt like I couldn't change. Then in the end, I didn't submit the application for the major, and the counselor asked me specifically, am I sure not to transfer? Do you have students around you who have successfully transferred? There may be many students in our class who have transferred majors, yes, because our basic learning is similar to that of clinical practice, and about 20% of us feel that there are maybe five or six or seven or eight people who can transfer successfully. Do you know what majors they go to?

The 14:11

part goes to the clinic.

14:12

are mostly going to the clinic.

14:15

In fact, there is a certain difference between basic and clinical. The main one is to concentrate on scientific research, and the other is to focus on clinical design. The change is actually quite big. You suddenly stopped being a doctor, but concentrated on doing scientific research. This is not a little bit contrary to the idea of you want to be a doctor in the oral cavity, right? This change is mainly about your own interest. After you understand the basics, because your interest is mainly an influencing factor, is it mainly interest, or is it the influence of teachers and students, or is it a consideration for career planning?

15:09

The main thing should be interest, and then we were the anatomy teacher in our freshman year. He happened to be the only one when he was in class with us, that is, he felt that the foundation was a very good major. I feel that all the people around me are saying that the basics are not good enough and want to go to the clinic, so maybe the teacher gave me a little confidence to stay in the basics.

At 15:42

, most of the teachers you teach you will also support your transfer to the clinic, right? Yes, like this, yes. We even teachers say that you will change majors in the future. We feel that many students have this factor themselves, and then parents also have this factor, that is, they feel that all hands are pushing them, just want to change majors, Why not to you? Instead, you are because, although my parents did not support me a little when I actually gave up at the time, but if I gave up changing majors, your parents did not support it, yes.

16:18

But I think this is my own business after all, or I want to start from my own point of view. Did your parents discuss it with you when they were not supportive at the time? I want to know the specific details of the discussion.

16:34

They just said let me think about it. Well, why do you know why they don't support it? I think they still don't understand the basics. Because you think it's because they don't know enough about the basics, they may have a little misunderstanding of the basics, and they think the basics are not easy to find a job . This way, but then after you get in touch, you think it's pretty easy to find. When I was working, I went to a counselor to talk. At that time, because of the basics, I usually read a doctorate before I can find a job. At that time, I was actually very worried about this problem, and then our counselor told me that there are also clinical foundations who can't find a job, that is, as long as they do well enough, they will not Can't find a job, there's no need to worry too much about these um.

17:22

That is to say, the one who has a more important influence on you is the first one, the first teacher who recognized the basic major in front of you, and then your counselor gave you professional confidence. right. Then I feel that there is not much obstacle to my career and career path, plus my own interests, I feel that there is no reason not to choose the basics, right?

17:45

Yes, and we watched a video when we were in high school. We were giving a lecture by Mr. Yan Ning. At that time, there was a video about where all the female scientists went. I was very touched at that time, because I was very interested in biology in high school, and then when I decided to stay in the foundation, I suddenly thought of this, I went to the video and watched it again, and I felt more determined.

18:15

Can you tell me some specific content that this video has given you firmness, that is, why what content this video talks about touches you?

18:27

What I'm impressed by now is that girls will easily give up because of various things, which is the path of scientific research, because I may think that family members say they want to find a job. I remember him very clearly at the time that he had a Ph.D. He was still a few months away from graduating, but at that time his family told him to look for a job, and then he finally gave up his Ph.D. Well, it just felt like the girls weren't brave enough to do what they wanted to do Things are affected by many factors in the environment.

19:05

So the basics are what you want to do, right? It's been clear that at that time, the parents were thinking that they wanted you to continue your major, mainly because it was mainly an employment factor, right? right. Then after you talk to the counselor, you communicate with them about these things that you know. I don't really remember how you convinced them, you remember. It doesn't matter, don't say it if you don't remember. Because I feel that once I make a decision, it is generally not easy for them to change.

19:50

Your parents still know your character well. How did you find out about middle school? At that time, the publicity was actually very good. First of all, when I was a freshman in my class, when I entered the laboratory, I was actually entering the laboratory of the middle school, but at that time, it was because the teacher taught us to assemble, and then they called them the laboratory of the middle school. It was very good, so I contacted the teacher to go in, and this was the first time for the middle school class at that time, and the teachers also pushed him to be more diligent, and then I learned that, and he seems to be in our class group. related notices, so each of us is aware of it.

20:39 In

other words, the publicity is still in place, you think it is right. I remember he should have held a lecture at the time, right? Some of you participated, but it was still taught by Teacher Sha at that time, and did it touch you in any way? Mainly because I was in the middle school class, and then I was in the middle school laboratory, not the middle school class, and then you worked in the laboratory when you were a freshman, right? Then I went to the middle school class again at that time. I didn't think that so many things would happen in the beginning, so I joined directly, and his publicity was indeed very good.

21:25

At that time, he chose the junior high school class. He should have listed a few advantages for you after joining the junior high school class. What are the conditions, which one is the most attractive to you? Perhaps the most attractive thing to me is the scholarship of 10,000 yuan per year . Scholarship? What should have been there then? For example, I have special courses here, and then you just said scholarships, and his 5+1+3 academic system, right? There are many other things that I have to exchange abroad, yes. Among the few conditions, what attracts you the most is the annual scholarship of 10,000 yuan, right?

22:19

At that time, this factor should be the most direct one. Then I remember he also said that the students in the middle school class did not participate in some activities in the school, because I might be lazy when I was a freshman, but I actually participated in my freshman year. There are many, these activities, but I think doing scientific research for me is actually a waste of time. Then I thought at the time that I might be able to concentrate more on scientific research, but in fact later, those activities are not necessary to participate in, if you have a lot of attempts.

22:57 Right

. But then, I actually felt that especially now that I was getting older and older, it was actually necessary to participate in many activities that were compulsory at that time, because according to one of our opinions at the time, we might not be particularly willing to go, but for us Future development is still very useful.

23:17

For example, for some seminars, he will ask our counselors to ask our juniors to participate. The seminars of my secondary school are mandatory, I don’t think he will come to seminars from other schools. . That is to say, through these channels, your information acquisition will be beneficial to your future choices. After learning some information about the middle school class, do you think the most fortunate point is the scholarship? Then did you sign up? And at that time, because I was in the middle school laboratory, the teacher felt that he also asked me if I had signed up at that time, and maybe he also hoped that I would sign up, and then I felt that I didn't think so much about other things at that time. After signing up, I didn’t think much about it. The main consideration is that anyway, if you have the money, you can go first, and then he is actually halfway through and you don’t want to. Can you still quit?

24:28

Yes, that's what he said when we entered, so I think it's not a loss to become a shareholder anyway. What factor do you think has the greatest impact on you? Just joined the middle class and joined the middle class. Mainly just thinking about it. I put most of my energy on it because when I was a freshman, the teacher gave me a small topic, so I wanted to focus on scientific research. At that time, I felt that the middle school class could give me such an environment, so that I could be a laboratory teacher who can do scientific research with peace of mind.

25:14

have a big impact on you? He was also nice to me. And some of his thinking actually has a great influence on me now. So you still want to join the junior high school class because of this teacher. Yes, there is. Through the image of the teacher, I feel that the impression of the junior high school class is deepened in all aspects. Yes there is, right? After joining the middle school class, what is different from what you thought, does it meet your expectations? But I think most of them are actually fine, but the main point seems to be that after entering the junior high school, it seems that after returning to the school to work and manage, and then returning to the national government, I feel that there are more chores, so that's right.

26:16

What club or college student union did you join when you were a freshman? Yes, which one did you participate in? I was at school and dating again. Both of them participated in dhv, is the Ministry of Culture and Entertainment very busy in what department of each other? It's very busy, there are a lot of things to do. Then when I was a freshman in the lab, I mainly went to listen to group meetings on weekends. I didn’t say that I would spend a lot of time on a subject, but I knew that if I was going to do a subject by myself, I would definitely spend that time. To be freed up.

26:59

You, but do you feel like you have learned anything in the school and student council? Well, I think one of my thinking is a great improvement, especially when I was a freshman, I entered the laboratory in my freshman year, largely because I brought the student council, I was probably my first at that time. Participation, a selection of scientific research interest groups, at that time I found that everyone was really excellent, and then I did not join the student union, I would not know so many excellent people, and then I would not have contact with them, nor would I I would say that as soon as I was a freshman, when my classmates were simply studying, or when they were in class or staying in the dormitory to play games, I went to the trainee tutor to enter the laboratory by myself. Well, it is the student union and the college union. , In fact, it even pushed you further on the road of scientific research, that is, after joining the junior high school class.

28:03

During the learning process, apart from what you just said is that one of his management is different from what you think, there are other aspects that you are doing, such as scientific research. Does it meet your expectations? He asked for a rotation at the time, and then I went to the teacher's laboratory except for the first time I was in the teacher's laboratory. The other teacher's laboratory just felt to know more about something.

28:33

Is it what you thought it would be? Originally, you thought that after entering the shareholder class, there should be a better environment to do scientific research. Well, yes, this is also available, and it still meets your expectations, right? Is there anything that has happened to you after joining the shareholder class that impressed you? I remember them from the National Affairs class. We seemed to organize some very interesting activities by themselves, which was to increase a connection between teachers and students.

29:15

Because at the time, it seemed that he had sufficient funds. He once held a dumpling making competition, and then it seemed that a series of activities were very interesting anyway. This is the first time for me to get along with the teacher in such a way. I have never had such an experience in the club or the student union. The students in the club feel that there is still a class difference between the students and the teacher. The word is to let you experience it, and the relationship with the teacher feels closer, right? Has there ever been one thing that disappointed you in particular?

30:03

Then we seem to have some courses at that time. The Chinese middle school will be cut off for us because he wants to add new courses for us, and then add the rotation of scientific research. He also counts school hours.

30:20

Then at that time, it was mainly because we were rotating in different laboratories, and the tutors gave us grades, which were also considered as the main course, and then I felt that the grading standards of each teacher were very different. I feel that some students and their teachers will give very high marks. Maybe I think I will perform better than them, but my grades are not as high as theirs.

30:51

Then later, they cut off advanced biochemistry. I think this is a very important course. When they said that internal medicine and surgery should also be cut off, I think it will be more useful for us in the future. I wanted to try to keep these classes for us, and then I discussed with the teacher several times, but I felt that there was no answer in the end, and then I cut it off, and then I came out.

31:20

But in the end they seem like these classes are still on senior life as if they didn't. Should still be up. This course setting is a little different from what you expected, and there are many courses that you think are meaningful. But it was deleted by the teacher, right? right. It's one of the reasons why you want to leave the junior high school. Yes, it is also a reason. This should be the most direct factor, because we mentioned this matter to the teacher several times at that time, but in fact, there is a contradiction in the middle school class, because some students feel that it will be easier after less classes, and they do not want to. Extra class, but from my point of view, especially the advanced mythology, it talks about a lot of molecular biology related, because when we were in the second year of basic sophomore, biochemistry was divided into two parts, the first part was biochemistry, and then A part of sub-biology, but a lot of things related to our experiments are in the latter part. If we don't take this class, we are just doing experiments, rather than going to a deeper understanding of a lot of theoretical knowledge.

32:33

I think that although we can also say that we can make up for this aspect through self-study, I still feel that the teacher's systematic explanation is still very much needed, and this is the most direct factor.

32:47 Right

. Wait for other factors, that is, are you the eldest sister who left the country? Second sophomore. Just joined, I stayed there for one semester, the second semester, and I left not long after the semester started. This is the most direct reason you just said that a course setting is the most direct reason, and then when you started I asked, and then you said one thing at the time, that it may affect your export guarantee, right? Is there any other reason?

33:23 When

you were a sophomore in high school, you had already started to study the matter of guaranteeing research. You have to ask this question clearly, or else it will be too late later. At that time, it seemed that it was not only me who asked again, but several classmates were also asking about this, but there was no clear answer. Which school do you want to protect? Peking University. At that time, did you discuss with any teachers about your ideas about Baoyan? Have you discussed with the teachers of the junior high school class, what class do you want to leave?

34:10

At that time, we had discussions with the junior high school teacher who was the management teacher, and then the management teacher also went up and discussed with the leaders above. At that time, besides me, there were many students who had this idea, but later they The answer given is probably that the middle school class is mainly for the training of graduate students for the middle school, and the graduate students still have to stay in the pot, but they have not stated exactly how, but your graduate student does not really want to stay in the pot.

34:42 Right

. Is it totally unacceptable to stay in the country? postgraduate. I was probably at the moment when I decided to stay in the foundation, and I almost decided that I had already thought about it, and I would probably take this road in the future. Your plan is already very clear. Have you talked to your classmates or good friends, that is, from the time you haven't joined the junior high school class, you want to leave after joining? Should have spoken. But because our dormitory is mainly my dormitory, only I go to the activities, um, but I feel that they are mainly listening.

35:34

is mainly to listen to what you say, it will not have any effect on you, yes. Have you discussed it with your parents? Do you think it was because they didn’t know much about them. Then when I said I was going to leave the middle school, the teacher asked me if I had asked my parents what their reaction was, but they didn’t know much about it at the time. What kind of impact will the junior high school class and the non-resigning junior high school class have on me in the future, and the most important thing is my own decision. When you choose to leave the junior high school class, can I understand that it is the most important factor you consider? , one is your career plan, that is, your school should be considered as an academic plan, such as where do I want to study as an undergraduate, where do I want to study as a graduate student, which affects your plan, um, and then there are things he learns, courses One of the settings is a fuse.

36:35

Yes, mainly because of these two factors, and at that time, we had been communicating with the management teacher, but we felt that we had not been able to solve it, and then the teacher felt that our questions could not be solved. It was because I have this feeling, because I can't get a response, so I deepened a response to such a thing, but the response is that I won't add this class to us, and I can't go back with satisfaction. Later, I even went to the teacher of teaching affairs myself, and I seemed to have said at the time, I don't remember his reply at that time, but I felt that the middle school teachers didn't seem to convey our thoughts to the top, and they themselves told us that we couldn't do this. .

37:27

Do you think the teachers in the middle school class didn't help you solve this problem for you? I didn't even try to help you solve it, yes, that's how I felt at the time. This has brought you great disappointment. Did anything happen that made you feel particularly satisfied while you were in the middle school? Is there anything you are most satisfied with about the middle school class? Maybe it's because after we have finished the rotation, there will be a report after each rotation. I think the report may let us not only know how our classmates are learning, and then we can find out what the other labs are like. environment, I think this is done very well.

38:14

Well, is there anything you're particularly proud of? One thing you have done that makes you very proud, in the middle school class or freshman year or the entire current undergraduate period, I may still be in the teacher's laboratory. At that time the active laboratory pair. He is uh, teacher, after I learned the experimental technique, the teacher was very satisfied with me at that time, he even said to let me do an experiment, that is, a report of the experimental results to tell him, because at that time, they were a freshman after they came in. Time to study or have such a report, it took me about a month to complete all these experiments, and then organize them into charts to report to the teacher.

39:07

Well I think this is a very fulfilling thing. This is the big one. One is wrong, it should be the second semester of sophomore year. This time to join the country, it seems to have joined. Does the sense of achievement make you feel that it is good to stay in the middle school?

39:35

is mainly to make me feel that I may be more suitable for doing experiments and scientific research. Because I feel that I really like the way the teacher trains me. Well, he also attached great importance to it. Then he gave me a subject and asked my senior brother to take me to do it, and he would also take the initiative to discuss this subject with us? ? But later I felt that I had to weigh the pros and cons, because at that time I was actually very entangled when I quit, because the teacher was also very good to me, because the brothers and sisters were also very good to me, and I thought that after quitting, they would not be able to do me in the future. If you want to bring undergraduate students too much, there will be such a concern.

40:26

You are still determined to choose a special terminal. At that time, you mentioned a concern that may cause teachers to be reluctant to say, and we all left as undergraduates. Are there any other concerns that make you hesitant to go? And the subject I was working on at the time, I had probably been working on it for a while, but I didn’t finish it. If I left, the subject would be abandoned here, but I was actually quite familiar with it.

41:02

Did you finish the project later? No, yes, I leave it to my brothers and sisters, yes, my brother seemed to have planned to do it when he graduated, but he didn't have time to do it. But that subject is mainly about data analysis. If it is an experiment, the impact may be a little more biased.

41:26

Topic analysis yes?

41:29

At that time, after you left, you had to make a decision. When you were leaving, what your teachers and brothers and sisters had to tell you, whether you chatted or gave something to others, when they said that they were talking to me. , because after I left, they would actually have one less person to help with the experiment, but at that time, Sister, she also said that she still asked me to do what I think would be better for my development. I think they still treat me very much. it is good.

42:08

Let me take a path that is more suitable for me. They still support you in chasing your own ideals.

42:19

Did you mention to them some of your concerns or experiments at the time? Also, will you be less willing to recruit undergraduate students in the future? Did you ask them what they thought, or did you discuss it with them after you made a decision before making a decision? I might want to leave. I didn't seem to have gone so far at the time. I briefly talked about the general situation. Did they advise you to stay?

42:55

No, yes, because Senior Sister respects my feelings more, I also told him about it, because when I had a period of wandering, we happened to be taking a biochemistry class, and then our biochemistry teacher he There happened to be a Volkswagen team over there. I saw that there were only three people in them. Then I contacted him. I said that I wanted to do a big innovation with them, because the subject of my rework in China was mainly partial, but I actually From a basic point of view, the main thing is to do experiments. I don’t think there is such a subject for experiments at that time. I want to make up for it from other aspects.

43:42

Well, then after I stayed in the laboratory over there, I found that the teacher in the laboratory over there was a teacher after I went there. He didn't ask me to do the subject of Daiso, but he gave me another subject to do it and I was on top of it at that time. There is a senior brother who is also an undergraduate, and he has almost completed a project himself. I think I have seen a path that I can take, and then you and I have more confidence in undergraduate scientific research.

44:20

Did you participate in Daiso and the Challenge Cup?

44:28 Was

it the teacher of the junior high school class at the time? Not with my current teacher, when did you participate in Daiso? Participated in sophomore year.

44:41 Was

n't he in the middle school class? I won't come out in the second semester of my sophomore year. After I come out, I will participate in another Daiso project. I feel that what I have learned is completely different from doing the project for myself and doing it with my brothers and sisters. I will encounter various problems. , and then figure out a solution yourself. You can talk about specifics. For example, the one who impressed you the most is doing big innovation.

45:10

I remember that during the exam month, we took one exam a week, probably at such a frequency, and then my teacher had an article in the process of revising, and then he asked me to help him with his vision. , but it needs to be stained with several antibodies, and then others can be stained very well, but the target protein I made is very strange, and it cannot be stained.

45:42

I repeated it several times, but the effect was not very good, and then I felt it was actually quite uncomfortable, especially during the exam, everyone was reviewing me and doing experiments there, and then I remembered that I even had some I made two copies of the information, one is the one that can be viewed with gloves, and then but in the end, it was very coincidental that there was once a tube of antibodies, which was not too much, I probably added that much, PPS for dilution, and It could be dyed that time.

46:15

Then it turned out that the antibody may have been put for a long time, and the titer has decreased, and then re-touched a concentration gradient. It was such a reason that it was not stained. He tried a lot of factors, and the problem of falling off from dehydration , maybe then antigen retrieval, or there may be problems. At that time, it was because the process was still relatively simple. At that time, I still felt that there were more processes. I just had to find out what went wrong step by step.

46:53 How

do you feel about this thing? As I think, doing scientific research is really not an easy thing, but it may be solved by some coincidences.

47:13

But also keep trying, what do you think?

47:19

Is there any specific thing that made you choose to take the path of scientific research very firmly? We should mainly focus on when I decided not to change my major. At that time, I voluntarily gave up this opportunity. The road is probably this way.

47:50

Well, is there anything that makes you particularly approve of doing scientific research, because you originally wanted to do clinical research, and then changed to do scientific research. In fact, they are two things that are not very different in nature.

48:11

We may say that when we talk about being a doctor, I am going to treat diseases and save people. It may also be that I consider the occupation is equivalent to that it is not very static, it is a very dynamic and changeable one, and then if you are a doctor, Including personal character, then occupational employment factors, such as a doctor's income, what else is included, compared with scientific research, have you considered these factors?

48:49

Well, in fact, although he has more free time for scientific research, his income is really not as high as that of a doctor. Then there may be a lot of mental pressure, because you often have to write tenders to apply for funding. But I may have another factor at that time, that is, although there are many diseases that can be treated in this way in clinical practice, there are still many diseases for which there is no cure. I still feel that there are still some bottlenecks in medical care that need to be broken through, but it is not enough to rely on clinicians for these things, and it still needs basic scientific research to further improve and consolidate.

49:40 When did

you come up with this idea?

49:45

I feel that some problems need to be solved by my researchers, which may have something to do with my topic, because the protein I make is a gene related to a rare disease, and especially this rare disease, it It is unlikely that a company will develop a drug to treat it, because even if he spends a lot of money to develop a drug, as a rare disease, it has very few receptors.

50:23

At the time, I thought one was that it might feel like a social problem, and then I did a research on drugs for rare diseases, which would give you some shocks.

50:43

Have you discussed with your friends, do you want to do scientific research?

50:50

Well or, friends, teachers, I seem to have discussed with my current teacher, why did I at that time because when I went to him, I told him that I wanted to do scientific research, and then I felt that he was like this because of me I had an idea, so I didn't know me for a long time. At that time, he gave me such a subject for me to do.

51:16 When was

your winter sickness subject accepted?

51:22 It

was also when I was a sophomore in my sophomore year that I was a sophomore, probably before and after I left the middle school. What kind of teacher gave you such a topic, it was our biochemistry teacher at that time, and the biochemistry teacher was the teacher of the state-aided class or the biochemistry teacher. The basic teacher of the department, and after you followed this topic, you have strengthened your determination to do scientific research, right? After leaving the junior high school, have you ever thought about leaving the junior high school and have you regretted it?

52:08

No, but it was actually very stressful at the time, because I was the first classmate who left the middle school. I didn't know what to do at the time. Then, I was in the middle school class for a while. The leader of the school, then the leader of the Basic Medical College, and then the leader of the Academic Affairs Department ran around here, dealing with myself here and then finally, the student status also went back, and even then the teacher even said that I would make up a retrieval course, um, that's it , and then at that time, it seemed that because the counselor said that I seemed to be missing one credit hour, they asked me to participate in the scholarship evaluation for this academic year after making up the time off. However, our class was actually very full at that time, and then the retrieval class was arranged. I was It didn't say that there was a very suitable time to go. I really couldn't search it later. It seemed that there were three classes, I could only attend the first two classes, and I had to go to my own class for the third class, and that was it.

53:22

At that time, I just talked about stress. At that time, you faced a lot of stress for a while, mainly because there were a lot of things to deal with in the process of returning from the junior high school to the basics. For example, what you just said When making up school, this is one aspect of the pressure. Are there other pressures?

53:46

I think the most important thing is that I actually feel a lot of pressure when dealing with so many leaders, and the middle school was in its first year of establishment, and then I quit, I actually think it was It is also not good for the Central Office.

54:06

Then even I met the teacher of the middle class on the road, and I wanted to walk around. What is wrong for a while, I feel that from my point of view, it may be a kind of disapproval, but I am actually very I like the teacher of the middle school class, but after quitting due to some factors of my own, I will feel a little just that, a little bit ashamed of him.

54:38

You think you left the junior high school. The reason you left the junior high school was actually because of your own factors, not because you felt right, but mainly because of my own factors, yes. Do you think you would be willing to stay in the country if you meet certain conditions? This should not be. Neither is right, because I think doing scientific research or I still want to do scientific research on a better platform. I will use an analogy. For example, if he allows foreign sales, even if he allows foreign sales, I think it is even worse for me to occupy a place in the middle school for 4 years and then leave.

55:28 After

you left the junior high school class, you are now a senior. Have you cooperated with the shareholder class teacher? My topic is actually that my teacher and Teacher Sha have some cooperation, but I haven't personally experienced it, but after meeting on the road, I would still be happy to say hello, and I can't say that before, I felt that everyone was still very kind. Then the study after leaving the junior high school is in line with your expectations, yes.

56:08

And you are defending the school. When you know that you are going to prepare for the summer camp during the summer vacation, after you leave the junior high school, you will do other subjects and follow the teacher. Do you think it is all in line with your expectations? Have you encountered any difficulties?

56:36 The reason

is that my current teacher is a lecturer and he does not have a graduate student. Although I have a senior brother who is also an undergraduate student, there are a lot of things I want to do. In fact, he can't. We are now in the laboratory. It is divided into various groups, and there is no straight world above me, but when I was in my middle school, I always had my brothers and sisters take me with me in everything I did, and then when I came here, I felt that as long as I was on my own, it was actually very good. I was afraid, but the senior brothers and sisters in other groups felt that they were very good people. When we asked them, they were still very willing to tell us, have they dealt with it?

57:22

Is there a competitive aspect to it? Between groups, yes. Maybe there will be some competition between the little teachers in each group? Have you ever dealt with it? It's okay if it's our undergraduates, they're not very likely to blame us. That is the study during the third and fourth years of the junior year. Do you think there has been any change? compared to before.

58:02

I think that my ability to do scientific research has improved, because if no one leads me to do many things, I have to find a way by myself, I have to look at the literature, or I don’t talk about it. I can completely make it myself and feel more and more independent.

58:21

Doing scientific research by yourself, when you are busy, if you encounter some problems that you feel that you are not able to solve, you mainly ask the teacher for help or say you have no seniors and seniors to guide you, yes.

58:41

But I have a small teacher who mainly discusses and communicates with him. Yes, I will discuss with him, and I will also discuss directly with other groups. Has he talked to you about the middle school class at that time?

58:52

I also asked him about the matter of dropping out of middle school, and then did you tell him, you are testing what factors you should consider to drop out?

59:03

It seems to me that when I retired, I also wrote a table and said the reason for staying in the middle school and the reason for leaving the middle school, but I don’t remember the specific things. This table still there?

59:15

This should be gone.

59:17

Can you briefly talk about the reasons for staying in the junior high school and the reasons for leaving the country? I don’t remember much, but at the time I remembered the teacher and he read my form, and I showed him, Then he said that I was so picky about quitting the middle class. The reasons were so many factors that supported my promotion status. At that time, when I said this, I actually felt a little clear in my heart. Maybe I still wanted to say it more because I felt like I stayed in China. Class is also like a comfort zone, in fact, jumping out still requires some ideological struggle.

59:57

Have any juniors and seniors come to ask you that I want to go to the Pudong class? Have. What did you tell them then? I will give a brief introduction, but I will try not to express some of my own subjective feelings, I will try to be more objective, and ultimately let them make their own decisions.

01:00:24

Do you compare you recommend them to go? When you are considering whether to recommend them to go, what are the main points that come to your mind?

01:00:34

I think for undergraduates, if we do have classmates who have already found a good mentor in their middle school, and graduate students plan to follow him, it is actually very advantageous. , that is, he and this teacher can start his postgraduate project about 4 years in advance, or at least 3 years. Yes, he will save three years in the future. And he has been integrated into this laboratory for a development during his later graduate studies is also very beneficial.

01:01:10

So you think it would be good to say that the undergraduate course can be coherent with the future, so there is no need to go to this class again, right? State Administration. To see if we can find such a teacher in the middle school class, the most important thing is to see the teacher.

01:01:30

I mean, for example, freshmen and juniors, they have not yet entered the supplementary class, and if they come to consult you, it is mainly to consider whether they will recommend them to supplement the middle school. class.

01:01:49

What you just said is that if they are already in a middle school, I mean they can't find such a teacher in the middle school, because the doctoral class is not direct, they don't encourage studying in the middle school, they can even I will tell them that there is such an advantage here. So it is actually suggested that if they have such an idea and want to do scientific research, they can join our class.

01:02:16

Do you think the main thing is to see if they have the will to do scientific research, right? When you are in the middle school class, this is when you are in the middle school class, there will be younger brothers and sisters who will ask you if you are? After you quit, it will mainly be after I quit, because I only stayed in the middle school for one semester, and there were still relatively few people who asked me at that time.

01:02:42

Later, I felt that after you quit, did they come to ask you how you quit and why you quit? Yes, some people will ask me how to quit, and then you will always ask how you are special to them. why? There may be a situation similar to mine, and then there was another student in our class at that time, because he wanted to do the subject of immunization, and then the middle school asked him to only do the subject of the middle school, and he also dropped out of the middle school class, But he was still on the subject he wanted to do on his original teacher's side, so in fact, your main consideration is to compare your own interests, yes.

01:03:20 After

you dropped out of your junior high school class, some classmates asked you why you dropped out? What do you say? Mainly, I should usually talk about personal interest factors. If they ask you if I want to join the junior high school, will there be any disadvantages, such as the curriculum you mentioned before, I will not say.

01:03:50

Because I think the middle school class is actually a very good setting itself, and as the first class, we definitely have many problems, so we should continue to solve this problem in the future. But at that time, I was already out of this kind of class. I will no longer use the junior high school class I saw at that time to judge the current junior high school class, and I will not tell them the problems I encountered at that time. Speaking of, what is your impression of the doctoral class as a whole, I think it is still very good.

01:04:19

Which aspect is good?

01:04:19

In fact, I think they have a model for cultivating students, 5+1+3 is right, and they are very systematic in the scientific research rotation report, so that students are of course more scattered than everyone else. There is no teacher in each laboratory. You can go if you want, and you don't want to go. He may actually I think it is very beneficial to go from a novice to the top.

01:05:13

It seems that there are not too many problems. About when did you start to feel that basically your parents are no longer your masters?

01:05:26

basically make up your own mind.

01:05:30

I think that’s what it seems like after entering university. It’s more obvious when entering university. Today, the application for volunteers has already started. Yes, mainly they don’t know much about it. Then I was at home by myself. Anyway, after the college entrance examination, I had nothing to do. I would look for various materials to find various materials. The main reason is that you just talked about an APP. Apart from the APP, what other ways would you use to learn about it?

01:06:00

I thought I was on Baidu or somewhere, so I looked for me, but I forgot the rank corresponding to your score, and because I felt that the score fluctuated too much, there was no reference, but it was from previous years. No one can sort out the ranking for us, and then I will sort it out myself. We'll be here first today, yes.
