# Supplementary material for: Why do you choose this program?—A decision-making model of medical students based on grounded theory
Source: PLoS One. 2023 Sep 15;18(9):e0291634. doi: 10.1371/journal.pone.0291634 (PMC10503722; doi:10.1371/journal.pone.0291634)
Supplement: S1 File — (ZIP) [file pone.0291634.s001.zip › RAW DATA/P9 CHINESE.docx]

05月17日_1.wav

00:01

可以开始了，你好。我们是一直教育研究所的老师，主要是想做一项课题项目，所以是开展一个访谈，这次访谈我们主要的目的是还原你从活动班的宣传到报名到录取的所有环节，主要是想听听一下对你影响比较大的事情或者印象比较深的事情，包括你自己的感受，你的情绪，这些东西都可以跟我们聊，尽可能聊出你的想法和影响你的每一件事情。

00:31

在这个开始之前，我需要读一下实验道德伦理须知，本次访谈中受访者是在平等自愿的原则上参与的，受访者必须真实的表达自我想法和认知，确认自己符合受访条件，访谈的过程会被录音，但录音的资料仅以匿名的形式用于科研，不会泄露给任何的第三方，在访谈的过程中和访谈结束后，你都有权取消我们录音资料的使用权，你是否知晓并同意？

00:59

同意嗯好在开始之前你先告诉我们一下你是哪一级哪个专业的吧，我是19级预防国重的。1972年。你在大一的时候也是预防的吗？还是不是，我大一的时候是医政学院的。你真的对。为什么真的我们要问的第一个问题就是想先问一下你在大一的时候是怎么了解到公用班的有什么渠道？我觉得这是一个非常偶然的事情。其实我是觉得国中的宣传可能在对医政学院的学生来说，他是宣传不是很到位的。

01:48

我之所以知道有国中班这么一回事，是因为当时我看我很多同学他们都转专业转走了，然后像我们这一届医生的话，前20%大部分都已经转走了，然后我又看了很多，因为我成绩不算差，然后有些就是成绩没有我好的同学他们也转走了，但是我当时没有转专业，我就心里很难受，然后我每天都去上教务处的网去看有没有寻求机会算是，然后后来就很偶然的在开学的时候在我记得特别清楚是9月3号的下午，我们辅导员就发了一个可以转国中班这样的一个通知，然后我就想还是虽然时间非常紧，因为它是10号就要考试，然后我要准备的话一个星期都不到，而且我还没有书没有资料什么的，但后来想一下还是要试一试。

02:44

反正转专业就转走了。大概是这样。你说是很偶然之间知道，你说很多同学在前面20%都转走了，他们一般去转转去什么专业了，成绩比较拔尖的去了临床，然后预防药学这几个然后像成绩相对一般的去了基础，然后声筒，还有护理，英语都有。

03:17

你说你很难受试试，你有没有想过是为什么是觉得他们转到了你觉得比管理更好的专业是对，因为我其实一开始是不是很想进专业的，你当时为什么没有考虑转专业？因为我的学科有限制。你是文科对文科只能报英语管理。还有什么？护理。对，但是国重我特地去看了，他招生简章上他写的是就那种学科不限，然后我就觉得是一个机会。

03:51

所以当时你要是转专业去选预防可能还转不上。是可是这个意思吗？不是转不上，是没有资格转，没有资格转。就是条件上去砍死你，但是你有这么一个机会去你在我我想了解一下，你在高中时候报专业的一个过程是怎么样的，你当时不是也是非常曲折的一个事情。

04:21

怎么说我是南京的，不知道你知不知道是中华中学的我知道，然后当时我们那一届非常特殊，他是在大一上下学期的时候，刚下学期开始就分班了，也就是说正常的分班那是在高二开始的，但我们相当于只上了一个学期，一个学年都没上完就开始分班了，然后当时我的成绩上怎么说，就历史非常的拔尖，历史、历史、政治、历史、地理，还有化学是非常拔尖的，基本上都是年级前几名，但是我的物理非常的差，不能说非常重要，就是趋势让人很担忧。

05:05

我第一次考差不多年级100多名，第二次又是200多名，第3次300多名，第4次500多名，趋势非常的不乐观，对的，然后我的老师可能也为我好吧，我当时报的是理科，然后他说你这个成绩完全报文科是非常有优势的，因为我政治历史都是年级前几名，然后他就觉得可能一方面为学校升学率考虑，一方面为我自己考虑，他就觉得我报文科会比较好，反正后来就被说动了，但是我当时其实非常的难过我，回家还找我妈哭了一场。

05:44

但是怎么说你为什么要接受老师意见，你就说因为我当时其实看自己的物理的情况也不是很乐观，我觉得你可能还是对他感兴趣，但是你可能我选不好就很难了很吃力，但是像政治历史，就说说一个离谱一点的例子，就是在一模的前一晚上，只要因为第二天只要考政治历史了，我晚自习一直在跟别人共谈过的，然后但是我第二天还是双月家，可能很适合就比较适合，但是我自己并不是很喜欢，我在他学的过程中虽然很轻松，但是我觉得我并没有体会到很多乐趣。

06:26

你当时在高中时候有想过转理科吗？在后面学习，包括你们那个时候还有小高考吗？就那4门。学那4门的时候，你没有考虑过要转转一下吗？就是没没办法转了。对我们学校就不给转卡死，然后我现在了解到我大学同学当中居然还有选历史和化学这样的组合的，我就觉得非常的奇怪。

06:55

所以你当时选了文科，就学校就不让你转了。对不让我转就相当于被小孩这个样子了。我们高中还能转很的卡的很紧我觉得。

07:08

所以其实我个人觉得分科来说，对于后面的专业选择，其实差别是很大，但是你说它有没有必要，我觉得我觉得他不是很必要，就像我之前有看过像美国他们那边的教育的话，它虽然有文理之分，但是它的文理它的分科跟中国完全是不一样的，我觉得一种它是偏向于那种理论艺术那些，然后另外一种就是偏向于运用实践的，但是你说这两种跟中国的我觉得不太一样，我不是很喜欢中国的分科方式，其实因为我觉得比如说你一个文科的学生，感觉你好像进了文科班之后，人家就给你一个有一种潜意识好像说你学生数学理科就不强，但是你如果去了一个理科班的话，人家给你的那种第一个刻板印象，就觉得你这个人肯定文学造诣不高，感觉你可能做事很严谨，但是嗯不没有学文科的那种天赋可能会这么想，但是其实不是的，我觉得每个人应该是那种全面综合的发展，就是你选科什么的，我觉得不应该用文科和理科去把你卡死。

08:24

就像比如说我现在转到国中，我一开始可能会有一点不适应，因为大家都是理科，他们可能比如说学生理生化可能比我上手快，但是实际上最后考试结果出来，我可能考得还比他们高，就可能我对他的这个东西的理解可能比他们还有可能更深刻一点，我觉得我想回到刚刚高中时候，你被迫报了一个历史，应该叫你还说可以这么说，被迫选文科应该算是被迫选了一个文科。

08:59

你在报专业其实就已经很受限了，因为可能起码我知道我们学校可能就像那些理科专业你可能就报不了，你当时报的时候是报的都是管理类专业吗？因为其实我进医院是我第一志愿进来的，因为为什么？这怎么说，其实我一开始并不是想报南医大的，我的成绩其实可以有其他更好的学校，但是因为我妈妈不是很让我出初审，然后我就更被限制了。

09:32

然后再加上他们觉得正好议政网说起来以后，可能还能去什么卫健委考个公务员之类的，然后说出路也比较好，比较适合女孩子，就还是听从家人的意见，报的。你说听从家里的意见比较多，当时你在选文科的时候，你说跟你妈妈哭了一场，你妈妈当时有什么反应？你你还记得吗？她说的啥？我妈她当时就安慰我，因为我妈是文科的，我爸爸学的是理科，然后我妈她觉得其实文理并不重要，就是她其实就是意思就是行行能出状元，而且让我不要太难过，他就觉得文科其实也会很适应。

10:15

但怎么说，其实我是一个适应能力比较强的人，你说我要真的在文科班里有多痛苦，其实也不是只是有的时候有些受限的时候，就会让我觉得心里比较难受。比如说报专业，对，但其实怎么说，后来我就有点想通了，其实。你选择什么？基本上往回看，也不太可能还是向前看比较好。像你比如说在选文理科选专业这些时候，包括你初中报高中这些比较重大的抉择的时候，是不是你爸妈可能你妈妈给你的意见会稍微多一点，也不是吧，他们都会给意见，但是其实最终的决定权还是在我手上，但是我比较依赖听别人的意见，我比较愿意接受你爸妈。

11:05

是的，因为我觉得有的时候我的判断可能不会很准确，所以当时决定在江苏省在这儿上学，然后就选了南医大是吧？当时假如说就是说你妈妈对你上学地域没有限制的话，你本来是想报哪个学科的，比如说哪个学校？我本来想报的是西安外国语外国语大学，想学语言类的。对，因为也不是说想学语言类，因为它毕竟就已经受限制了，其实我比较喜欢的是我在高考结束之后填专业的时候，我比较喜欢的是社科类的东西但是，其实感觉社科类以后的发展就对很窄，然后就感觉还是语言类算文科类就业稍微好就业一点的。

12:06

对语言和管理这两个比较好，就有一些感觉，你和我那个时候很像想法，真的。

12:13

我也是学语言的，所以我那个时候选这个考虑就业，你那个时候说9月3号才看到辅导员发通知，然后没有资料，一个星期以后去考试，你是而且那个时候考的是应该是西西生还是啥？西生和组配。其实和祖辈其实你们管理专业大一有项目上过没有，我完全自学的，你用一个星期的时间学了，希望确切的说是4天，从4天的时候学了细胞和组胚是的。真的。我懂我。懂。真的，然后考试通过了，在上学的时候，你说不适应是指哪方面不太适应？

12:59

是你觉得理科的思维你可能感受不到，还是说知识不是我觉得是我自己有的时候对会给自己一种限制。其实有的时候心理暗示是很重要的，我为什么物理不好，其实我后来就有想过很多遍，我之前初中是在二十九中的特招班上的比较好的班级吧因为，我现在班上的初中同学上北大清华都有，他们都很厉害。然后我们班风气用现在话来讲说内卷非常的严重，然后我爸爸妈妈就在我初中的时候，其实他们一直是崇尚快乐教育的，就是我从来没有上过任何的辅导班。

13:41

然后在初二的时候我们刚要开始上物理课，我所有的同学好像我记得我们班上除了我以外，就没有人没有上过辅导课。

13:52

我我就觉得跟不上，当时然后其实一开始的东西都非常的简单，比如说什么声波震动什么的，然后我看大家在课堂上就很踊跃，然后考试也每次都考得很好，作业他们都提前写，然后我就觉得我我压力很大，然后我又没有上过课，我就觉得我就比别人弱很多，然后有这种心理在之后，我后来的物理不能说差一直就不晕，不火的状态就是很很中等水平了，但到高中之后就更加痛苦了。

14:27

然后所以就一下子很不好，然后现在转到国中之后，我总是觉得我身边的人他们高中的时候不是学的物化，就学的物生，甚至有些学物地，我觉得他们学的是理科，可能这三年培养的是一种理科的思维，然后我可能就缺陷那种思维，我总是觉得因为我是文科，所以所以可能比他们弱，然后这种思维就会让我有的时候就很痛苦。

14:56

对就是一种很不好的心理暗示，但是你可以说是你在暗示自己，可能我那个时候没有这个基础，对是的，但是所以我你往好了说，他有的时候会促使你内卷卷的比人家更加厉害一点，但是你往坏了说你就不想卷的时候，你想到这你就觉得感觉心里惴惴不安，就是有一种比别人弱的感觉。

15:21

我想想详细的问一下初中的那个时候的事情，你说周围同学都可能提前学过了，对于这个东西都很了解，因为造成了很大的压力，然后是不是因为周围同学好像学学的都比你快，这样一个环境让你感觉自己可能不太适合学物理，或者说自己不适合学，或者说自己不擅长学物理，可不可以这么说？

15:51

我这个解读这个我是觉得怎么说呢，其实我觉得之就之前我跟你讲了，我学政治历史很轻松，但其实我是觉得我是达到一种心流的状态，你知道是什么？是心流的状态吗？心流比如说一个人他在学习这个心吗？对，就是心脏的心流是流水的流，然后它这种状态是什么意思？其实是说你在学习当中能享受到乐趣，并且他一直能回报给你一些积极的东西。

16:25

然后你再去学的时候，你其实不知道自己在学你你，就觉得自己是一种很享受的过程，然后反而其实最后得出来的这种结果也往往比较好，可能我对学文科就学政治历史，我可能就处于这样的一种状态，所以我就觉得嗯比较轻松，但是像物理的话，我就想要试图进入心流的状态，却一直在被别人打扰，就反而成为了一种产生一种副作用，我就觉得我得不到乐趣，我觉得我再怎么学再怎么努力也超越不了别人，可能我就觉得我没有天赋，然后我就觉得学得很累，然后就恶性循环。

17:02

我觉得我学物理的状态是这样子嗯可能受到了可能叫班级排名的压力，影响到了你学习的一个状态，对，然后其实我有这种感觉。

17:17

最明显的其实就是我初三的时候，因为初三的时候不是又要加一门新的学科化学，然后我就强烈要求我爸带我去上化学的提前的那种，就暑假提前去上课。对我就很害怕对是的就是出现物理这样的事情。然后其实我暑假也没有上多久，就顶多上了一个星期的课，然后我后来的化学基本上全是年级第一，然后我中考还是南京市第一化学。

17:44

就就是，我觉得人的一个心理暗示是非常重要的。因为物理的话我自己我觉得是我自己在阻止我变好那种感觉嗯。

17:57

所以我可不可以说其实你还是挺在意自己在班级内的排位的这件事情。就是你其实学习的时候还是更更想通过考得比别人高或者之类证明一下自己的能力，你会让有一种成就感，那也不是完全像你说我要卷吗？

18:20

他是比较在意，但他可能没有我说的那么严重，比较在意的一个点。是对，我会在意他，但我不会特别在意，而且我觉得我现在国中应该不算特别卷的，我觉得他们都比我卷。真的他们太卷了。

18:39

所以之前有辅导员跟我们讲过，说有没有想过要国中班的同学，因为我们都是来自各个专业的，要不要换宿舍换到一起，然后我就不是很想跟他们换到一起，原因主要首先我在9栋是南医大最好的宿舍，听见条件就是我们是上床下桌独立卫浴，而且非常的大，就很好，你知道吗？然后如果要一起换的话，可能会换取一个硬件设施没那么好的宿舍，这是第一个。

19:10

第二个就是我觉得如果跟国中的同学都在一个宿舍的话，比如说如果他们在宿舍里学习，而我在追剧，然后我看到他们学习之后，我可能心里我就不会太想追剧了，或者我不想学习的情况下，我追剧也追得没有那么快乐了。您能理解吗？他们是不是很容易受到别人影响的那种？也不算，我觉得我跟相比这种环境会让你自己给你自己，对，但是你说比如说刚开学的时候，如果他们在疯狂的卷的话，可能对我没有任何压力，我该看电视还看电视，我该玩还是玩，他如果快临近考试月的时候，他们已经提前开始卷了，我就会开始紧张，是有条件的，其实我们就是出于上进心的一种不是我想，不是说你我其实他不是想去卷过他们什么的，是他们那种无形的压力让我不得不卷起来，其实你说我要真的卷了，然后我好像还没有真的卷过，我觉得你还是比较有自己的节奏的，不太喜欢被别人打扰。

20:19

是的，所以我比如说像学习，我之前有同国中同学他们就喜欢约那种学习小组，在比如说周末大家一起去学习什么的，我就不是很愿意跟他们一起去，因为这种学习小组会给人带来什么帮助吗？或者他们的目的是什么？就约出来去学习，陪陪陪伴，我觉得学图书馆学学习氛围就非常好，我就非常喜欢一个人在你有了解过他们为什么要这样做吗，或者说你感觉他们这样做的目的是什么？

20:52

学习小组可能关系好吧，可能大家各自在比如说这一个桌子，我坐这儿你坐那儿，然后我们都在学习不说话，我觉得其实很无聊的事情，我一般其实我学我学习一般喜欢一个人去学，就是一个人的话不容易受打扰，不然比如说他要走了，他还得跟我讲，我走了可能我本来在专注于一个事情，然后一他一说他要走了，然后或者说让我看一下他手机上给我发的信息，然后我就玩一下手机，然后我拿起手机开始可能就45分钟过去了。

21:25

确实有那种同学喜欢把大家聚在一起，对是有的像研究生开组会一样，有的有的同学研究生也喜欢拉着同门一起，肯定他们其实就是想看看你进度，做到哪了，你现在有这种思路，他要把握周围的人。

21:46

对我明白了，就看周围人学到什么程度了，我就不是很喜欢，如果比如说上个礼拜才考完免疫，然后就期末考试，怎么说有的同学他就会到处问你你看几遍书了，你题库还刷了，你理解背了吗？

22:08

你大题背了吗？然后他一问我我就很紧张，然后我都不知道该怎么跟他说，然后万一他就又比较热情，可能然后他就跟我讲说我听听谁，那书都背了好几遍书都翻烂了，我一听我就觉得很紧张，然后我就觉得我这一遍还没看完我怎么办，然后其实我是非常不喜欢接受这种信息的，我觉得要保护我的不知情权，他让我被迫知情我就会很难受，然后就影响我的节奏，对是的嗯。

22:37

但是其实我觉得我在正儿八经考试的时候，我自己的节奏把控一般还是可以就。

22:44

差不多提前两个星期左右，我就知道我可能我要开始复习什么，他们再怎么跟我讲，顶多我紧张一下，但是你说他一跟我讲他书看了两遍了，我就说我今天一定要把这本书看完，那不太可能，因为我觉得欲速则不达，你看可能就只是进度赶完了，实际上没什么用，可能会对你的心情造成影响，但是对你的实际行动和你的计划来不会造成这肯定不会的，因为我们每天都会列计划，我要做什么我就很明确，他一讲他打断了我计划，我后面的计划就全部散了，你每天列计划这个事情是从什么时候开始的？

23:19

初中。是你父母让你做的吗？还是你不是，我觉得这个是我在我北大同学的好朋友身上学到最大的一件事情，他就是一个非常有计划的人，是他教你的小小学校，也不是我们的方式不一样，像他把每天要写什么列起来，而我觉得我比较喜欢这个时间段，比如说像今天今天我上午有9:40开始有课，然后你看下午我要接受你们的访谈，就2:00，然后再到下午的4:30，李朝军老师要喊我过去讨论我们创新计划的事情，就是我要把那种限死的时间先画出来，然后剩下的时间就是我空余的时间，然后可能我会在这些空余的时间里面安排一些我认为近期需要做的事情，然后这样子就也比较好把控，如果像那种他是属于效率特别高的人那，我跟他就不是很一样，我是那种被迫效率高的，比如说我要考试要的，我不得不效率高，我可能效率率很高。

24:22

像这种平常的时候，我要把控的时间列出来，然后调整一些动态的计划，我可能就会 nh一天过得比较充实，毕竟是4天看完细胞和组织的人，你在我另外一个问题就是我知道你高中时候学的是文科，然后包括到大一学的管理，然后再在大一结束的时候，9月份的时候突然要转向学国重，你有跟你的父母讨论过吗？

24:54

事情这个是非常极限的一次讨论，我当时看到转专业的这就是有这个机会之后，我非常的激动，然后我当时心情是非常复杂的，我觉得我又觉得没有希望，因为我知道有很多人肯定是提前已经知道消息的了，我觉得他你说9月10号就要考试，怎么可能9月3号才说有这个事情对吧？也不可能有这7天正常人。

25:26

我觉得都不会能够准备的特别的好，其实我心里是非常没有底的，我觉得首先我这个基础也不算特别好，然后又是两门我完全陌生的学科，我就非常的纠结，我当时就打电话给我我，爸妈就跟他们讲说，我觉得我面临一个很重要的决定，我就跟他们讲，然后他们当时被我吓到了，因为我讲着我就哭了，因为我是觉得我是不是很想在医政的，然后这又是我现在能够抓住的一次机会，但这个机会能不能抓住？

26:07

其实也不一定，因为如果他这个消息但凡我是在6月份或者是7月份知道，我觉得我肯定很稳了，我就觉得我肯定会认真去复习，可能就不会那么急吼吼的，所以你哭了主要还是觉得对消息知道太迟了，然后我觉得怎么说，我对这两门考试是非常没有把握的，没有把握，但我又希望我成功，所以我就内心非常的难痛苦，然后我就很难受，然后所以怎么说跟他们也讲了，他们还是让我自己决定，因为他们首先对这个领域也不是很了解，因为我爸妈其实他们的工作跟医疗卫生，他们都是国家电网的，其实没有什么关系，他们也不了解，所以他们就觉得我应该自己去考虑这个事情。

27:03

然后我妈当时就问我，你为什么她就问我，我到底非是有多想转这个专业？然后当时就我记得特别清楚，我就说我是非常的想转，因为我一点都不想留在一阵，不要被内涵到他他无所谓，他无所谓，你不用担心，可能个人然后我就我就跟我妈讲，我说我非常的想转走，然后她就跟我讲，他说你知道这个专业以后就业的方向怎么样吗？

27:37

然后我就说我根本就没有考虑这么多，然后他就说你这个是不是考虑的太仓促，但是后来我妈又问我，她又问我为什么她转我转专业这个目的是什么？其实我当时的目的非常的单纯，就是我我就是不想在一阵因为首先我觉得学院我不对老师做任何评价，我觉得我这些同学的氛围是让我非常不喜欢的。

28:03

然后我真的我觉得我有些同学他们就像邪教非常的恐怖，然后我就我就很是的我跟你说真的非常非常的吓人，就怎么说呢，这一段一定要保密，我不想被邪教头头找到，没事保密肯定太好奇了，就我跟你说就怎么说，我隔壁有一个舍友，我没有任何地域黑的意思，我们都是江苏的，因为8个人虽然是独立卫浴独立的，但是它卫生间是通的，然后我知道。

28:36

对，然后他广西人，然后是侗族，反正他的素质我真的我觉得我就跟他们用洗手的就对，因为有他在那边洗衣服，他每天晚上都会在那里骂人，到现在也是只要他进来了，就他妈，另外就是医政的另外一个同学，我都不知道他们哪能有这样的深仇大恨，是骂隔壁宿舍还是他们宿舍内部是不是是骂4楼的，我是5楼的，然后骂4楼，就一边搓衣服一边骂各种难听的话，我觉得我这辈子都没有听到过这么多丰富多彩的骂人的话，再阐述一些负面的情绪，是的，就是非常的恐怖，然后而且他们那边就勾心斗角很严重，而且其实都不是什么有本事的人，就讲出来的东西都是很多都是无稽之谈，没什么。

29:32

上下嘴唇一碰，就编了一个东西，然后讲了锤死了，跟你讲这个瓜怎么样保证，然后其实我就觉得这是你隔壁宿舍的事情。

29:43

我隔壁宿舍还有其他班上也会有，然后我舍友都会讲一点给我们听听，但其实讲出来一些事情，我不是说我舍友怎么样，我我可能就是你们同一栋楼，这种传到处传这些对对，然后还有是因为原来我是一正的，他们就会讲一些呃，就不是讲我讲其他同学，包括老师一些很无聊的事情，然后就是八卦小八卦，然后有一个同学，比如说他在学习什么，他就是要打击你，懂吗？

30:18

那种考试都没到就开始学了。这么着急这种。你觉得听了会很舒服吗？就会很难受我觉得。

30:26

然后还有的就比较讨厌的可能，但是我觉得这种现象可能还比较普遍，因为我在国中班也看到了，有一个同学在学习的时候有什么资料，然后他就会过去你这个资料能不能发我一份，就这种，其实有的时候是买来的资料，他就讲白嫖你，我就觉得这个就有点烦。我觉得不太好，对。但我没有在我身上发生过，是我舍友跟我讲的。

30:53

因为我我是属于资料不是很多的人，我基本上只看书的那种，你转专业主要是因为觉得这个氛围你不喜欢，对最大的原因就是氛围，然后可以让你转宿舍吗？不是，其实第一是这个氛围我不喜欢，第二就是他怎么说，我觉得它的前景也不是很好，就业对前景我觉得不是很好。

31:18

第三我觉得学校对专业的重视程度其实也不高。然后第4个怎么说？第四的话，就他的师资力量其实也很一般，我觉得这个平台来说，我觉得我是被限制住的，而且我觉得在这个专业不是因为我不够出色被限制住，而是仅仅是因为我的高中是文科，我被限制住，所以我是内心很不甘的。

31:46

然后后来还是决定转国重。

31:50

你是什么时候开始觉得我想要转专业，我非常不认同这个问题。是逐渐的有这种感觉，还是说像侗族学生那个事情，让你一瞬间有那个就是，怎么说，我觉得我在大一上的时候，没有特别强烈的转专业的意愿，为什么？因为首先我觉得我自己可能不能转专业，所以你说我要是让我自己活得痛苦的话，我就想我为什么不能转。

32:19

但是你毕竟还对我那个时候想的我还要在这待4年，如果我一直想着我为什么不能这样，我太痛苦，了我还不如说我就接受这个专业，我就在我们专业比如说我就成为年级前几名对吧？

32:33

然后但是后来我我是进了院学生会，然后我觉得这个院学生会真的非常的离谱，可能我不知道是每个学院都这样，还是这种学生组织都这样。

32:50

我觉得我觉得我帮老师在打白工的就是工具，我也首先我不会提升自己，我这在帮他干活，比如说我是技术部的，然后院里面要搞活动，我每次都得拿着相机去拍照、剪辑，然后做视频，而且我得不到任何好处，对吧？有的时候甚至你做的不好，老师就觉得你唉你怎么这都做不好。

33:17

这种然后然后我就觉得后来我是什么时候泛然醒悟，是在12月31号，就是19年我真的我那次非常的生气，你可能我其实是一个非常有个性的人，怎么说他他又不惹我，也不可能那样子对他。

33:38

我老师竟然要求我元旦就是12月31号有在学工办陪他加班，你知道我要做的是什么吗？我要帮他做什么？年终考核的p我觉得非常的离谱，这明明就是他自己的事情，问一下是辅导员老师吗？肯定是辅导员，我知道我做过辅导员，还有学院的一个书记，他现在已经转走了好像。对是的，我觉得我知道他已经被调走了，我真的非常的生气那一次。

34:12

他就林林明老师和高老师挺好，林明老师真的我觉得他有的时候可能他心并不坏，但是他做的事情是一些非常低情商的事情。

34:22

我他就他就跟我讲说你今天必须留下来帮我把我的PPT做完，他是说你必须要留下，对，我就觉得非常的奇怪，当时你在学生会是什么位置，我就是一个非常普通的技术部干事，为什么一定要有一个干事？

34:37

因为他可能觉得我的能力比较强，因为像我之前做的策划什么都比较好，遇到过。

34:46

妈呀，你不知道我上个月一直在帮他就保卫处心理什么防诈骗什么吗？二级培养单位应该做的事情全被我做了，真的非常的奇怪的，PPT我做网上的视频，我做保卫处的内容我下然后再给大家做演讲，要老师干嘛承诺书都是我写的，真的非常的奇怪的，你可能没有到那个环境当中去，你觉得我讲的一些可能都是非常匪夷所思的事情，但是它的确真的是真实在发生了。

35:14

我一个技术部的，我进去之后，我觉得我可能只是需要拍照片做美工甚至检视频，我觉得这都是我工作范围内的，我也是愿意去做的。

35:25

但是你说我要帮你做你年终这种工作考核的p那是不是太不合适了，而且我根本就不知道你做了哪些工作，我怎么可能把它吹得像朵花一样的，对吧？

35:37

然后他就要求我一定要做好给他看，然后就跟我一起的还有邢朝阳学长，就我跟他两个人，他一个大师他好像是什么？校团委的领导就是向学生干部一样吗？

35:52

他大四我记得他是大四不是现在应该要毕业了，我不太清楚，然后他就要我做我当时就很生气，而且我当时在上课，我在上高数课，他给我发信息，让我立刻去他办公室帮他做这些东西，我就觉得很奇怪，然后我，反正我后来我一一气之下我把手机就关机了，然后我就直接回家了，因为我是南京的然后我回家了，我到家之后我又打开我手机，我看到17条未接来电，还有高玉宽，然后学长学姐那什么，张雨迅什么的，他们都给我打电话。

36:29

我说为什么要给我打电话？他说你赶快去什么去学工办，他你找很久了再不去你就完蛋了什么的。然后我就说就不会完蛋了，我马上法定假日我还能不休息吗？

36:40

我就很生气，然后第二到1月4号我又去他办公室了，然后他就在那我真的很生气那次，他就叫我去他办公室了，我就觉得非常有意思，他觉得还有点找我去他办公室，我进去之后我所以越说不是你主动去，他喊你去的，是的，我去了之后他也没讲，我就问他老师，老师你觉得一个学生他的本职工作是什么？他说一个学生本职工作应该好好学习，我说是的应该好好学习，所以我现在要开始好好学习了。然后他反正我就怼了他一番，然后我就走了。他肯定也很不高兴，但是后来因为还是下半，学期正好就正好就疫情了。然后疫情的话也在家里也没什么东西要做，也就相当于跟学生组织差不多，没有什么瓜葛，我就基本上是退了，因为我那次好像还挺高的，大家都有点害怕，大家还有我另外几个同学可能就知道了，是不是？

37:40

因为他正面刚老师同学们就比较觉得啊，这个人很好，是的好高，他们就觉得很高，是的。因为我觉得你说要是我那种站不住脚的事情，我觉得他刚刚没什么，我觉得这完全你说出去就是一件非常离谱的事情，我要再不高我也太软弱了，对吧？而且因为因为他这个事情再加上我大大一上学期的不努力，我的高数只考了60分，我就非常的难受。

38:10

我刚出来考了60分，说明你们都是考了五十几分，老师把你们努力全都是，但是你觉得你高数是因为对是的，因为我高期中考试的时候还没有受影响，我考试满分。

38:25

嗯后面就是他经常在我上课的时段让我过去，我就没有这，而且是理科这种东西，这种事情在12月31号那件事情之前，你上课的时候去，你都是直接会去的吗？接受吗？

38:39

他让你上课的时候去你就去，有的时候比如说他比较水的课，我可能会去对那种我可能就打个假条我就去了，但是后来这种主干课他侵犯到我当时当时就比较警觉，因为他基本上就是那种我一进去我可能看着我进去的时候太阳当空照，我出来的时候已经是月亮当空照了，我就觉得这个也太奇怪了，有好多词只是一个干事，是你们整个部门都在那里，不是我还有另外一个干事，非常奇怪的，我觉得我那时候做辅导员都是把部长拎过来，我从来不会拎干部长会把干事推过来，我我当时我太清楚了，当时李敏老师我有一个后台编辑的，那个号码是已经毕业的一个去在鼓楼医院工作的一个学习的账号，然后他是私人账号，我们就想说把它搞成企业版本，这样就不用每次都回去找最高权限的学姐让她报发东西，要她就把她读的时候就换到换成辅导员号码，因为李敏不想管这个事情，我们去找李敏的时候，老师可不可以把后台的号码变成你个人的？

39:52

这样以后换届的时候就不需要找上一届的学姐直接来找你就行了。他就说我聋了，我听不见，你不要跟我讲这个事情，我建议你们也不要管这个事情。我觉得李敏老师真的我对她就直接我聋了，原话我聋了，我对她印象是非常不好的。

40:11

因为我我们我们刚来的时候是怎么说他是去怀孕，然后休产假了，所以一开始是高玉宽老师接收我们。然后这个叫什么高玉宽老师，我觉得他就是一个非常憨厚的，好人老好人，然后反正也没过多久也比较风平浪静，没过多久生完孩子黎明老师回来了，我就觉得他这个人一个人我没有见过他，因为他回来的时候正好生完产我第一次见到，他真的非常的奇怪，我记得你说是变好还是变坏了，他在之前怀孕休假之前还可以，还是一个非常谦逊，特别有礼貌，就特别关心学生的一个老师，我对他的印象真的不好。

40:59

我除了PPT的事情以外，最主要的是我跟他讲疫情期间不是那种要申请才能出学校吗？然后我当时是4月份左右我的身份证过期了，然后就一直都拖着没有去换，然后在好像是银行有什么就身份证不更新会把你账户冻结，知道吗那种冻结，然后我到6月份的时候，我账户被冻了，我没办法就是花钱了，我就说老师我身份证不得不出去换了，我之前是我自己拖着，因为我不想给他造成麻烦，我说这没办法，我必须要出去了他死活都不让我出学校办身份证。

41:43

然后我到后来我身份证还是我考完试，然后暑假再去换了，就给我造成了很大的不便。我钱我妈打到我微信里面来，然后从微信再转出去用的，然后就他只本来是打到我银行账户，然后现在只能打到我微信里面。然后当时如果你单纯一个就是因为疫情防控不让你出去办身份证，我觉得我还能够理解。但是不是不是我们学校我们当时院里面有几个男生，他们就打着要办身份证回家看病，还是什么个洗头，甚至是出去吃饭这种乱七八糟的借口，就出去玩。

42:20

她都批了，就唯独女生她就经常不批。那你有没有试着找，比如说高老师他们高玉宽会不会因为他那个系统他就只能李敏过了之后才能太高，他没有权限。

42:35

然后我当时就非常的愤怒，我就觉得你这个也太双标了。那些男生他们还比较不收敛，正好我就微信好友看到他朋友圈里，就在朋友圈里，大家那又是唱k了，又是帅火锅了，我天天在学校吃食堂，连办个身份证都不行，我又很难受。

42:58

然后反正我后来又去跟李敏老师讲，我说为什么他们能出去，我不能出去，他就说你这个还是到什么就跟我打你懂吗？不说重点，然后就想糊弄我那种意思，然后正好又是考试了，我不高兴跟他烦了。我又还是对，不敢啰嗦了，是太浪费时间了。

43:17

你当初加入学生会有什么就是目的吗？或者说是想想出于一个什么目的想锻炼一下自己？

43:25

学生会的话是当时加入还记得吗？

43:29

我记得我当时是刚进学校是临招就是那种临时班长，然后我就不是很想做这些东西，因为我进这个学校我知道我铁定是一阵之后，我的目标就只有保考研和出国了，我的目标还是比较明确的。然后我我一开始是觉得我就好好学习，我就不太想让这些社会活动的这些身份。但是后来赵高老师他有一次找我就是单独面面谈，就是说还是建议我要去参加一些社会活动，然后让自己履历丰富一点，然后我感觉他说的也很有道理。

44:11

然后他就跟我讲说学生会进来之后以后对什么评优评先奖学金什么都是有帮助的，可以也锻炼自己。然后他也说得非常的真诚，然后我就同意加入了大概是这样嗯，可能还是会对你保考研其实还是有一些帮助的。

44:32

所以但是进入以后发现很失望，和自己想的不一样，做了一些事情没有什么技术含量，也不是也不是很失望。我进去之后，我是非常感谢我部门里的学长学姐也好，还是大家同僚也好，就是因为非常的苦，所以我们那个部门相当的团结，我们到现在就即使我转专业转走了，我们的友谊坚如磐石，真的。

44:59

比如说学院里面有什么大事，他们还是会跟我讲，然后就要心情不好的时候也会找我聊，就关系真的非常的好。

45:07

我觉得我最大的收获就是收获了这批朋友，然后你要问我对我自己个人提升有什么帮助的话，我觉得可能我也不一定个人提升有没有达到当初的预期，对。

45:22

或者说对加入学生会有没有感到怎么说，我觉得是一种对半开的对半开嗯还是收获朋友还是有收获，是的，你不能一味的否定收获的东西，因为你遭受到的磨难对，你就除了这些朋友的话，可能觉得好像也没有什么收获。

45:40

也是有，我觉得我剪视频的能力好像就精进不少，因为被迫要学习吗？然后就也是学到一些，但是你说他有多么多好像也不是，你要两个一起说的话，更多的还是我觉得是对我学习生活，包括精神上的一种负担。我一开始不是很会拒绝，他有什么事情都说，赵晨你去搞一下去拍个照片，你去录个视频，你去剪个视频。

46:11

我当时还盲目的认为这是我生活充实的一种表现，但后来我发现他这种充实并不是我希望得到的充实。你表面上看着我非常的忙，但其实我内心很空虚，我觉得在打工，然后你去做这些事情，我得到了什么收获什么，可能就打发的时间。这里面哪件事情是让你坚定地要转专业的，就是liming那个事情。是叫黎明，对，不是他还没有这么强大的威力。

46:48

我也觉得应该不是，我觉得要说我转专业的话，其实大一上的时候，我说我没有这种想法，其实是因为我不了解政策啊，我觉得我可能就只能烂在这里了，那就没有办法，那我就好好做我医政学院的学生对吧？也就做个鸡头号了对吧？

47:14

然后到后面的话，我是发现那些成绩没有我好的人，甚至那些很游手好闲的人，他们因为是理科，然后就转走，并且还在那种耀武扬威的感觉让我很不爽，然后然后怎么说，因为他们这种变相的对我的刺激，导致我他们跟你说了什么或者做了什么，或者说发到什么比如说发朋友圈，然后大家都在那个群里面就说我又转走了，然后大家好多人都恭喜你种鱼塘什么，然后还大家说什么，恭喜你终于逃了，是大家都不认可这个专业吗？

48:02

怎么说大家应该心里都有数，也不能说都不认可。

48:06

所以我说我这个专业啥样，在你看来，班子里面就是身边同学们的感觉都是对专业的认可度不高的，假如说没有一些限制级，你感觉周围同学都是想走的，是有这个感觉，但是但是但是有些想走的，他只不过对未来就是那种对未来的一种美好的憧憬，就是我希望获得一个美好的感觉，但是我又躺倒懒得动不想动。

48:34

这种感觉大医生里面大部分的人都是给我的感觉都是不能说大部分有一部分人可能是想好的，但是他不愿意努力，还有一部分是想好也愿意努力的，就是我的舍友。自从我转完专业之后，你知道他们上学期排名提升的有多猛吗？我转专业之后，他们原来的排名有一个是前20%，其他几个都是50%开外的，现在他们变分别变成了一镇的第一第五和第十。为什么？

49:05

因为现在他们已经没有办法转专业了，现在为什么突然就开始，我觉得可可能是有我的影响，因为果冻我个人觉得是学习强度是比医政肯定是要高的，然后像我一到考试月，我就基本上在图书馆，他们基本上都是很难见到我的，因为因为我早上走的比他们早，然后晚上回来的时候他们都在床上，基本上都看不到我。然后可能有这种他们觉得可能我很努力，然后他们也要好好学习，可能有这种影响在吧？

49:43

一个宿舍如果有一个人考研，其他的基本上都会考，然后像我上个学期是学生理生化，然后基础化学那些，然后我大部分的课我我好像我记得没错的话，应该是我每天都是早吧，然后很累，然后他们就觉得其实我有时候早之后呃早上8:00上课就是第一节课，然后就得早起。然后他们上个学期还比较闲，然后可能就觉得看上去我很努力，然后他们也就努力了起来。

50:20

就是被击了。

50:21

有可能我。

50:25

怎么没有你这样的事儿？

50:28

你当时觉得管理学可能就业不是很好，对吧？觉得太宽了嗯，不能说不好吧。

50:38

我的真实想法是这样的，我觉得它是一门万金油式的专业，哪里都需要关，你有没有想过其实语言类也是万金油专业。

50:45

是的，因为这这当时我当时高考最后选专业选语言或者是这种管理就是这样出于以后就业的一种考虑，但是我上了大学之后，我就更加发现，其实你万金油的这种东西它是可替代率是很高的，你要么就做到顶尖做得非常的好，你就成为管理学大师。然后要么你就只能是个普通的职员，如果在没有什么那种上进上进心之类的话，可能也就一直就这样子温水煮青蛙一辈子过去了。

51:24

我是觉得我在一镇这边可能没办法像我自己预想的那么好，因为其实我之前还参加了一镇的职业规划大赛，然后还得了蛮好的名词。然后但是我对比我父亲和我母亲他们的职业，我觉得我爸爸的职业更不可被替代，它是有技术含量在里面，父亲的职业方便说吗，他是 it工程师可以这么说，因为我之前小时候经常去做什么英语演讲，就会问问你爸爸直接怎么样，然后我就脑子刚一蹦出来it，安吉尔尼尔。

52:10

然后我就问了一下，然后对他软件工程师了，然后他的其实在你觉得你这种想法是会受父亲职业影响的，想做那种比较有可以说是高技术含量的工作对，我不仅是他吧，然后还有就是嗯，可能还是对于就业的一种考虑。

52:34

对，然后像我我之前在高中的时候，急性阑尾炎，然后非常这情况很不好，当时就是很糟糕，反正后来医生就把我救回来了。

52:48

然后我我当时就对职业充满向往，然后所以这可能也是后来我明知道自己文科可能找不到专业，还是报了南京医科大学的这种一种素质，是的，我觉得很多东西都是一早都注定，像我在初中的时候，我的生物非常的好，就跟化学生物跟化学非常的好，然后还参加了生物的竞赛然后，但到然后就到了高中的时候，分科我又没办法分到理科，我当时就很难过。

53:22

但是其实在我初中的时候，我经常喜欢去那种看一些公众号，比如说像生物探索之类的，然后再加上我那次就就是期末考试，还有中考的时候就生物不是提前考的，也是当时南京市然后我是考的满分，我妈就送了我一台显微镜，我当时特别的高兴，我就跟我妈讲说妈妈我以后肯定是人生武功学家，然后我就说我肯定是搞这种研究的，然后我妈说好太好了，你以后肯定可以，然后说他就鼓励我，然后后来我到高中的时候就分科，然后我就觉得我自己跟生物可能这辈子都不会有关系的，我很难过。

54:11

但是后来又就是怎么说又转到了国重，然后像我导师上一次在公开的大课里面给我们推荐那几个公众号，全是我初中就关注的，然后我当时就有一种冥冥之中的那种注定的感觉，然后我就很感动，可能被自己感动了应该是很我觉得我是你的话，我应该会很开心很感动，感觉应该很多成分是感觉很幸运。

54:39

是的。

54:41

正好有个这样的一个看法，这样看漏了，你可能是到现在都不会走到那条路，是的，我觉得冥冥之中很多东西有那种注定的感觉，可能也是跟这边比较有缘分。另外一个还想问的就是你你，你也可以不说，这可能就是你随口一说，你还说这个学校对他重视度不够，或者说学校的管理这类专业的老师平台可能不够高。

55:11

是可以讲讲具体一点，或者说有什么样有什么事情让你有一种感觉，或者说或者类似的有什么情绪或者导致了你有这种判断，或者说你只是随口说的也可以。

55:25

我我我觉得不是的怎么说呢？我觉得在南医大就存在感比较低的专业有两个，一个是医政，另外一个就是英语嗯。然后我为什么会觉得这个学校对他重视度不够高？是因为怎么说？你看他发的一些推送，我觉得它的推送上面就像我现在转到工位来，我们这边有很多注注重于学生培养的一些东西，比如说我们有大创，我说的是不能说工位就是我国重，因为其实我觉得国重跟公卫还是有有有点区别的。

56:11

像我现在国中的话，你看我觉得学校就很重视，我们首先我们的课程跟其他的专业预防的专业也好，基础的专业也好，我们的课程安排都不一样，我们会有很多自己的课程，比如说生殖生物学，然后金石，然后还有那种生命科学前沿进展，这些都是学校想要培养你，所以才会投入精力让老师来对来特质。有这种课程。

56:40

但是你说在管理那边的话，从他们课程安排上来讲，我觉得让我的感觉更像是在没课找课上的感觉。

56:50

你比如说我舍友她现在在学内外妇儿流行病这些，对他们来说也是一种折磨，对老师来说也是一种折磨。他们给我给我的反馈就是那种他们老师不知道怎么给管理类学生上这种课是地震在上内外妇儿内外妇儿流行病，超级厚那个书，那个数这几门都是我大三大四甚至大五，我分开来学的课程就是。

57:20

一一一次一个学期最多上2门的课程，他们5门不是。他们应该也上，就是说要求不会像他给他们那么高的要求，但是你还是要去了解。我觉得我室友好可怜，她一点都不喜欢这些。但是她还得一直在看这些东西，而且老师也不想给他们那种讲得多讲太多。

57:47

老师也嫌烦，就觉得你们学管理学医政的，对吧？以后也不是要去做临床医生的这种了解，就好了还安排这么多课程都不知道该怎么讲。

57:59

然后你看他们快要考试了，考试也不知道该复习什么资料，因为临床的像我这边的资料那么多，全是题库，几千道题不可能那么多的大题要背，那么多明确要背，他们就觉得这么多没有必要刷那么多，他就不知道该怎么学就很痛苦，然后像这种课我就觉得其实你全部给它归成一一门，比如说什么临床基础这样子，然后让他们去学，我觉得反而比拆成这么多5门课去学要效果要好得多。

58:32

对吧？

58:33

然后你看他们这学期除了这几门课以外，就没有其他的课，而且这几门课对他们这个学期都在上那个f另外-2就性病没了，这强度很大的，他们一天他们这学期我知道这个课很难，但是没有必要给他台那么多的课时，但是他们这学期就只有这几门课，就特别多的课，我可以把他的课表给你看，你想看课表啥安排，我们只有流行病，没有内外妇儿。

58:58

你想看课课表吗？

59:00

非常的困难，我真的非常的可怕，就是他们全是走吧，全是早上8:00上，然后有的时候因为我这学期有几门免修的课，然后我就相对比较宽松有的时候，我回宿舍比较早，他们还没回来就很辛苦啊，而且像我的舍友就是他们在一直已经算成绩很好的几个了，表现出来，每次跟我讲到这几门课的时候，表现出来的，情绪都是很负面的情绪，他们就觉得也没有必要学，也不知道该怎么学，老师也不太愿意教，就觉得课程安排非常的不合理。

59:35

然后从课程安排上来，我觉得不是很科学，也体现了学校可能没有那么重视。然后在大一的时候就我自己上的那些课给我的感觉很水。

59:53

水是指太考试太容易过了，还是没什么实质性内容？

59:57

老师上课也是念PPT，念完就过，你其实就说直白一点，我这节课认真听和我这节课不听，最后我考试成绩可能都是一样的，这种感觉。像一般这种水课它的期末考试它不叫水课，是微观经济学管理学那些我大一上真的是很很，然后他们没怎么学，然后但是这几门我都还挺好的。

01:00:29

这都85分以上。

01:00:30

背下来就高，我都没背，我觉得就吃高中老本。

01:00:38

卫生经济学，你高中老板卫生经济学没有学他们大三的课，学的是管理学，管理学就三个字，管理学就是橙色的那种，我觉得非常简单，橙色那种叫医院管理。可能课程不一样，我那我那本就叫管理学。真的我觉得特别简单，管理学原理好像没有人就管理体系。

01:01:05

然后他教学安排都是在不停的变。

01:01:10

对，可能跟你那个时候上的都不一样了，然后我当时上的时候，我一开始还特别有兴趣，因为我记得第一次给我们上课的老师叫对。

01:01:22

对陆芳还是方老师还是陆芳老师，然后我觉得他上来很有意思，因为他讲三个和尚挑水吃故事，然后我就觉得这课还挺有意思的，我还很认真去打击了你的兴趣，后来换不是陆芳老师上了换了其他的老师，然后什么负责一段课时，对什么唐伟伟唐伟老师，他上课进来就说大家聊聊天，然后他又说大家来有什么问题问问我就感觉在我感觉在上综艺啊。

01:01:58

开心又不是不开心，我就觉得你好歹是那节课，然后感觉没那位张老师油油的。对，然后我不是说他不好，他后来画重点什么的，画的都很精准，对吧？然后讲讲的也还可以，但是陆芳老师他的授课形式我就比较喜欢，然后后来他还给我们搞的辩论会，我记得我还是最佳辩手，然后呃我就很喜欢这个课，很有意思，然后后来他要考试的时候也没有什么资料，大伙也没资料，然后学长学姐那边也都说没有特别多的那种内容，就看看PPT什么的，也也就过了一遍，反正就直接去考，我觉得我写的都是大白话，但其实老师上课如果没有讲什么特别多的知识点的话，说明他还是会就直接看见答案给嗯看，你的理解程度有可能吗？

01:02:57

然后除了课程安排上面，我说老师给我的感觉就是学校没有很好的资源，可能主要就是因为我觉得跟基础工位然后临床这边对比起来看的话，就师资力量就显得比较薄弱。

01:03:16

然后简单这来源于你对薄弱这个词的定义，可能就是来源于比如说刚刚老师念PPT，有很多老师念PPT，对，然后像我现在上课，很少有老师能让我觉得这个课我听不下去，但是我在一镇那边我就觉得这个课我听不下去，还不如我自己看。

01:03:35

听完就觉得这个老师上和我自学其实效果差不多的，对，我举个例子，比如说像我现在在上病理，然后我那个老师叫马娟，我不太记得了，病理的老师我觉得他讲的特别的好。

01:03:53

他条理很清晰，首先比如说你这个病灶它是什么病变，然后它的镜下观特点，它的大眼大体观的特肉眼观特点，然后他的主要的一些特征什么，他的条理就很清楚，我就很容易get到他说的重点在哪里，我就知道我应该怎么学怎么背，然后怎么去刷题。

01:04:16

但是可能也是学科之间有那种不一样的地方，比如说我在上微观经济学的时候，我觉得是一门很理科的课程，但是老师讲出来感觉我觉得我在听历史课，微观经济学是你大一上的，对大一上的。我就有一种上历史课的感觉，真的就是宏观经济学才应该上升的历史。宏观经济学是大二上的，那个时候我转专业了，微观不应该上成这个样子。

01:04:47

微观其实微观我觉得很多还是需要计算的，不是要看那个线什么，但是他讲出来的感觉就是他纯在念PPT，然后而且而且陈明生老师是院长，他他讲的其实还是很好的，到后面换了一个老师来之后，我根本听不下去了，就感觉听他上课跟自学没什么区别。

01:05:13

对，而且我不知道他的重点在哪里，就给我一种满整本书都是重点，但是整本书好像都不是重点的感觉嗯。你有什么我到下一个问题。没有。

01:05:35

问题我就问下一个问题，我其实聊是聊挺多的，你还是没有回答是什么事情坚定了你要转专业对吧？其实我觉得当时大家都转走了，对我冲击很大，我就觉得我因为文科限制让我不掌握，很不敢发现又有政策又有这个机会，而且其实我觉得他很有意思，就是不孕不育它现在已经是一个非常热点的问题，可能也是因为我现在能说出这个话，可能也是因为我现在在升职这个领域在学习，然后我对他的接触就很多，然后我现在就发现真的是一个非常严重的不仅是医学问题，也是社会学的问题了。

01:06:24

你看它中国的经济它也面临着压力，而人口这第7次人口普查数据都出来了，你看人口又减少了对吧？而且那就说明老龄化又加重了。但是现在很多人就排除那些社会因素，他们本身比如说丁克不想生，有些人是想生生不出来的，可能这就跟环境压力，然后包括其他一些因素有关，我觉得我现在去学这个升值其实是非常有意义的。

01:06:57

它就不仅是对我自己，我觉得他可能对我自己未来就业会非常好，而且它是对这种可能说到对人类的未来发展是有意义的。

01:07:10

我记得我之前跟我那个实验室的师兄交流过，说你师兄你在这里做博士，你觉得快乐吗？他说他好不假思索的回答我说快乐当然快乐，我特别做实特别喜欢做实验，他们每天都觉得非常有意义，因为可能今天做这个实验，对明天来说推动社会甚至是医学的发展，他就他就激情饱满，我就就受他的感染，我觉得的确是很有意义的事情，然后你就回到刚刚你问我的，我为什么坚定要转那，其实说白了的话，那个环境我不喜欢，然后他们就是那些比我菜的他们也转走了，我心有不甘，我就想我也想试试看，我总结下来的话，本来就是一进来的学院的环境就不是很喜欢学生工作，嗯嗯嗯，还有课程设置的一些失望，达到了一个也算是达到一个顶峰了，然后这个时候发现大家都转专业转走，我就更难受，然后又发现有政策。

01:08:14

对，而且其实当时我舍友现在一这年级第一的同学，他跟我是非常好的朋友，又是舍友。

01:08:24

我当时看到这个消息之后，我第一时间就转给他，我说要不要我们俩一起试试，因为我们俩成绩在一镇都算比较好的，然后两个人都是因为受限于文科没有转遗留下来的前20%，然后我就跟他讲说是不是你们前20%理科都差不多，该转都转走了，基本上全走了，我们当时招生进来有120多个人，现在医生就九10几个人，然后我就说我们要不要一起试试，然后他就觉得他他是不是很喜欢医学类这种东西，他觉得学得很枯燥，然后他就不是很让人，然后又说这时间也比较匆忙，可能准备了也打水漂，就是没有什么收获，还是说你要转的话就我自己转，我说那行，我就我自己转了。

01:09:15

大概好，前面过程我们了解的差不多了，然后现在想想问问你现在读国中班开始，有没有让你比较开心的事情，或者让你比较沮丧的事情都可以？开心的事情。开心沮丧都可以，让你印象比较深的事情，我觉得印象比较深的事情就是完全是不一样的环境。你像我现在到觉得环境不一样，给你最带来最大的冲击是学生还是老师，还是说整个上课的氛围或者说课程设置这些东西，你觉得哪个给你去赶紧带我，我觉得都有。

01:09:59

最多的还是我觉得老师对学生的重视程度，让我觉得转变非常明显的。

01:10:09

我到国中之后，你像我们现在要进实验室轮转，要选导师，然后还有各种各样的实验室的课程，我觉得这个就让我觉得我在这个学校的一些时间是被就充分利用起来，我觉得我是在学正儿八经在学到东西，我就有一种那种满足感。

01:10:33

然后特别是每次像考试结束，然后出成绩，然后轮转进行轮转，一学期轮转结束，然后进行汇报，汇报的时候我就觉得我这学期没有白努力，我就觉得还是有收获的，我就觉得日子过得很充实。

01:10:51

每天都有事情要做，也不可能有的时候接下来也不会像在以前学院一样，觉得你今天又无所事，我现在就觉得接下来就是一种放松休息，然后其他的时间很安排的很紧，然后我就觉得很充实很时间很富，对于我来说利用起来利用的很好，然后就很开心。

01:11:19

有没有让你感到比较不开心的事情，可以是自己个人或者说其他的跟国优班相关不相关的都可以是吧？有的，其实我觉得我进国重到现在不能算两个学期，也差不多要两个学期了。

01:11:37

然后其实我心情还是有一定变化的，就从我刚进国中我是兴奋而又期待的，我是想在这里我能遇到什么样的人，然后遇到什么样的，老师学到什么东西，我会成为什么样的人，我是充满好奇的，然后但是我就觉得随着时间这样子不断的迁移，我觉得现在看看国中班也不过如此，最大最为什么我现在突然会有这种想法？

01:12:06

是因为之前发生一些不太好的事情，在国中班里面。我不知道前面的那些受访谈的同学有没有跟你讲，因为我没关系方便讲，我觉得他可能已经讲过了，我我知道戴安庆也说了访谈，因为我跟他关系很好，然后怎么说我们有特色课程，就是叫生命科学十大进展。

01:12:34

然后上这门课的老师可能在授课过程中说了一些跟政治相关的东西，比较敏感的话题，我就直接直接直接说这个老师他说了一些关于棉花新疆棉花事件的事情，它有不同的立场，它讲说是说什么来我都不太记不太清楚，但是大概意思可能跟国家宣传的。

01:13:04

不是，其实我是觉得他说的是非常有道理的。不是他其实讲的意思不是说中国不好，他的意思其实是说我们中国强大了，但是我们的外交的政策，包括是国民的素质都应该与时俱进，都应该一起强大起来，而不是他觉得外交部的发言人叫赵立坚嘛嗯，赵立坚他在就是回应hm事件的时候，就有一种有一种农家妇人的那种小格局的感觉，他觉得那种那种说什么什么中国已经不是原来那些受你们欺负的中国了，然后我们现在强大了怎么然后我们已经有能力跟你唱反调那种感觉，你懂吧？

01:13:54

然后像老因为他着急今天具体发言了什么，我肯定现在是记不清楚了，然后我觉得老师他的意思就是说中国现在强大了，但是我们的外交其实还是有一种就不够大气，我其实觉得他说的是很对的，因为很多网友包括像有些主流媒体，什么人民日报新华社什么的，在表达对中国这种发展的承认认可的时候，难免会去带一些历史上，比如说中国曾经很弱被列强欺负这样子的这种历史的这种东西，可能大家现在就就这么看，其实是没什么的，无可厚非的，但是如果你在每一次外交的时候，都要讲出自己曾经是受害的弱者，然后然后跳出来说你曾经欺负我，但是我现在强调这种这种感觉我就觉得怎么说呢，感觉有点自卑，对有自卑感似的，就是不够大气，我觉得既然我们强起来了，我们就要学会包容，过去我们我们过去是那样子的，但是没有必要拿在外交场场合上一直来说这些。然后美国他们之前说hm的，这种新疆棉花事件主要是因为说我们汉人奴隶那些维吾尔族的一些像反华分子那些人，他们自己包括他们的家庭里面的一些人去采棉花之类的。然后又有人在网上就说出，比如说美国你们十年几百年前还奴役黑人的这种东西，其实他的反应出来就感觉好像是你曾经犯过错，你就不能指责我现在错了。

01:15:40

就这种思想我觉得是不太对的，你错就是错的，你无论是谁都能指出你是错的，不管他曾经是怎么样，顶多是他对吧？这种底气不足一点，但是我以前还没意识到这个问题，你说的很对。

01:15:53

对我觉得这样这个是老师给你们说的，还是你自己的思路是我对老师的话的一种解读，我是这样子，所以老师也在传达这个意思，对我觉得他传达是这种意思，但是其实有些学生可能在因为毕竟是生命科学十大进展的课，我们不可能花很多时间说这种政治上的东西，他老师可能一笔带给我讲了这种话，他说可能是我们的外交不够大气什么的，然后他就讲了棉花的事情，然后说黑奴的事情。

01:16:20

其实我一听我觉得因为我也是这种想法，可能我就觉得老师说的唉很很有道理，我觉得他这种想法是比较对吧？

01:16:31

前沿的可能，但是其实它是跟一些主流媒体宣传的思想是背道而驰的。它其实是在批评一些主流媒体，它的宣传的方式可能有些学生他就觉得你说中国不好，你屁股歪了，你汉奸你就是在美国时间太长了，然后就在果中我们自己的班群里面，这个反应是你周围国中班的同学的反应是吗？我怎么知道的，他们在国中班班群里面看匿名骂老师骂得非常的难听，我当时就觉得天哪，国中班的学生竟然就这种素质吗？

01:17:08

我当时有一种非常难受，就说不出来的难受，我觉得我当时辛辛苦苦去备考考国中班，我希望遇到一群这种杰杰出英才，结果遇到了竟然还是那种有一群有小市民心态的那种那种人，我就想到广西人我就非常的难受，然后我就很难过，我就跟我另外的同学我就说为什么国中居然还有这种素质这么差，的人？

01:17:37

你想看聊天记录大概跟我讲一下是什么内容，这是纯骂人是吧？就骂得很难听，我觉得我可以看一下，你比较好奇是吧？我可以等一会找给你看一下。他们就是那种艳气很重，感觉我是中华人民共和国的好公民，我要打倒你大案件的感觉，这关键是不是一两个人这样，好多人都这样。

01:18:08

然后后来居然还有人说，看看谁以后还去老师的实验室这样子。我觉得这没有必要，而且我觉得对于政治上这些东西，不光是对于政治，对于生活社会上的各种事情，每个人有不同的看法是非常正常的事情。

01:18:27

我觉得你作为国中班的学生，更需要有一种包容的心态去看待这个世界，对吧？你说如果你的思想就被主流的媒体或者你现在主流的这些科学产生的一些东西限定住了，而没有大胆创新突破，有自己想法的话，我觉得培养出来的基本都是华丽的机器而已，就你没有自己的想法对吧？

01:18:50

就发现华丽的机器你的用词我真的很然后我是觉得你国重不仅是要培养技术人才，更重要的是要培养一种这种开拓创新的思想的人才。你说国中你说我们去实验室做实验，你其实这些实验练个10天半个月的，谁不会我相信一正的所有同学来正儿八经培规培一下，肯定上岗证也都能考到。

01:19:18

但我觉得你在实验室里学的学的东西，真的是实验吗？我觉得那种我觉得应该学习导师的那种思想，然后他的那些实验的思路，然后但是你看就这一件小事，我就觉得他们的思想很被禁锢住的，也不愿意接受新的一些特别有冲击性的思想。

01:19:39

那么你以后在做实验的时候，你比如说你就我就讲讲的离谱一点，讲贺建辉他经营编辑那个人类，人类胚胎的婴儿在学术界是有非常大的争议的。

01:19:56

但是其实有争议说明它这个东西它是值得关注的，不然的话不然的话你像今天市场上李家青菜卖三块，然后什么陈家，青菜卖5块，这种小事能叫争议吗？

01:20:11

它价格这种落这种差别就不能叫争议，有争议的事情说明它是值得去被讨论的，就像贺建奎的事情，他精英编辑 Hiv感染的这种这种胎儿，他的确是不太符合伦理道德的，但是你说如果他成功了，它的确是一种很大的突破，可能我思想说到这已经有点危险了，但是我我觉得我还是不会去搞这种这种触犯伦理道德，有的人这就是跟我们访谈没关系，对可能会他的成功可能会激励某些人，比如说让更多的Tei而故意演上HIV，对是的，我觉得有的时候你讨论对你有的时候你讨论的点不对的话，你可能比如说贺建奎这个事情是好是坏，现在大家都说他是坏，但是如果你坏打错靶子，那也是不好的。

01:21:11

比如说有人觉得他这个坏是伦理的坏，我觉得这的确是伦理不太好，但如果你说他的坏，但你说他的坏是你他开拓创新的话，我觉得这个靶子就打错了。

01:21:24

我是这样子想的就，所以你作为真的是培养的学校培养的这些科研的学生，这种果种班的学生，我觉得思维是非常重要的。还是应该更加包容一点，然后更加开拓创新也，不论是对原来的思想还是新有的一些比较激进的思想，我觉得都应该是以包容的态度去看待它，我不是说你必须去接受，至少你不能因为我想到之前陈珊妮她有讲过说有些人在讨论就想一件事情的时候，他的说的在交流的时候就要你等于我即等于正确等于正义，我觉得这就是不对的。

01:22:08

你就说你比如说他老师他在上课时候说的这些关于政治的话题，你不认同对吧？你可以不认同保留自己意见，但没有必要把它抹杀掉。我觉得这就是两种不一样的态度。你有没有思考过可能来源于哪里？什么来源？就是他们的这种思维模式可能就不说很多了，就说你周围的同学他们的思维模式可能是非黑即白的，可能你觉得说中国不好，你就是一个汉奸，这种可能对于矛盾的观点矛盾是普遍存在的，像这种哲学理念他们可能并没有接触的很深。

01:22:50

你觉得这种可能来源于哪里噢，我觉得很大一部分是来源于家庭，还有属于的环境。

01:22:58

你有思考过这个问题是的，因为我觉得一个家庭对你灵魂的塑造是有非常重要的意义的。我觉得像我的爸爸妈妈，他们就非常注重于我内在的培养，可能这就是我跟我另外几个同学他们怎么说，他们非常善于考试，我考试经常考不过他们。

01:23:21

我国中的同学我真的非常的佩服他，上课他就在看时代少年团，然后下课他就开始追剧，然后考试月前就问我借笔记，然后我笔记借给他之后，他考88，我考八十几，然后我就非常的难受，然后然后但是你说比如说我想跟他交流一些比较可能有一些思想性的东西，他就懒得理你，会在你的班级里找到这种知己吗？

01:23:54

就是可以和你深度交流这些思想文史这些东西的同学。

01:23:58

目前或者说你的舍友有吗？你会跟你的舍友讨论这些吗？

01:24:04

没有，我是对，我想跟他们讨论这些，但是我觉得可能就不会讨论的起来，其实怎么说人家都说人生难遇，知己的确是这样子，我就觉得我觉，我觉得我现在最好的朋友其实可能就是我初中的那几个同学，他们都可以你你会和他们讨论这种事情。

01:24:26

我会跟他们说，然后他们也会发表他们的想法，包括还有我一个高中同学他也在南医大，然后虽然他是英语专业的，然后但是他其实是一个非常有思想的人，我觉得就虽然他不爱学习，但他喜欢看那些我觉得他就应该去哲学，他思想很浪漫，也非常有个性，你可以建议语言类考哲学还是不难的，然后他他想学什么人类学之类的，然后我就觉得跟他这种交流我就会很高兴，像有老师的事情出来之后，我直接就约我就跟他讲，因为都是在南京大，然后我们俩就去压操场，我就跟他讲，我说真是太离谱了，真没有想到还有这种事情。

01:25:12

然后他就跟我讲说其实哪都是这种人像我一样，我们俩这样的人很少，我说那好吧，就是嗯我觉得真的我听你讲话我再起鸡皮疙瘩，我觉得你讲得非常的好。为什么？因为其实我也思考过这个问题，就已经跟我们的访谈没有关系了，其实我在那里做访谈，我觉得我自己受益匪浅，真的吗？

01:25:37

我在和别人交流的时候，其实我也会发现很多人思维跟我是不一样的，而且我在和他们交流的时候可能会你跟他讲了很多，但他有时候可能也会意识到自己可能有一些不对，但他还是不会去接受你的思维，这种情况下还是很多的，所以其实你想这个情况，我觉得我个人的观点，我觉得可能还是他们的人文思维可能还是不够，比如说像那些最基本的教学观念，很多人的观点，包括现在影视作品也是，他对一个人的塑造，就是他要么就是一个纯好人，要么就是100%的坏人，他没有这种好坏杂有，因为每个人都有自己优点，每个人都有缺点，他在做一个决策的时候，肯定是对于他自己来说最优的一个决策。

01:26:26

你不能说他做决策他是坏的或者他是好的，所以说其实我觉得我的个人感觉，包括我和周围人交流，包括我上网和网友的交流，我都觉得大部分人其实你说的那种同学他们没有办法去说这个人是好人好多一点还是坏多一点，他们觉得你做的这些坏事你就是100%的坏人。

01:26:49

他做了一件好人的100%就是好人，所以他们没有这种矛盾的观念，他们不能接受一个人即使做了一件好事，他们不能接受一个杀人犯去就跳到河里，就有一个小女孩子这种事情，可能包括像比如说在网上看到那种怎么讲很难说碰到那种杀人犯，但是可能他有非常合理的理由，但是很多网友就是我在网上看那些评论的时候，我就会感觉他们不太能接受，这个人去做了一件坏事是有合理的理由的，他们都会去想他做这个事情肯定是有别的比较坏的原因，因为他是一个坏人，所以很多人是不能不太能接受。

01:27:32

一个人可能好坏掺杂了这种这种理念其实讲到根本上可能对于矛盾哲学观念可能他没有开始到，而且其实我觉得你要真的去做这些人文教育，给这些理科生做这些我觉得，你能有这个思想是因为你是一个文科生，而且你可能本身历史政治学的也很好，你有思想关系。

01:27:57

这些跟文理都不是很有关系，我觉得是跟自己的家庭教育是很有关系的，像我的父母说我爸是个理科生，我妈是个文科生，他们两个他们俩我觉得思想上就是很包容的，而且他们是非常积极地引导我的。

01:28:18

我觉得我现在之所以能够有我自己独立的思想自由之灵魂，主要是因为我爸爸妈妈在我小的时候就非常喜欢让我发表自己的一些想法，比如说有些事情可能对于家长来说，你没有必要去咨询孩子的意见，但是我爸妈他们事无巨细都会问我的事情，只要跟家里面有关的，比如说我爸妈最近准备要再买一套房子，然后把家里老的房子就是卖掉，然后他是像这种事情，我觉得其实完全就是他们的事情没必要过问我的，他们都会来问问我的意见。

01:28:59

然后你久而久之就是我就被养成了一种发有自己的想法的这种习惯，我会去思考，我看到这个事情我会去想这些事情，包括我觉得我妈妈对我的教育就像是一种饭桌，谈话的教育就是每次在家里面就是吃晚饭的时候，他就经常会跟我探讨一些比如说微博上的一些热搜底的社会事件，比如说之前有四十九中的事情，然后他就会跟我去探讨说究竟是这个孩子是心理问题还是怎么回事，还是你看一开始大家都说是学校校方有问题，然后后来又变成是孩子自杀。

01:29:38

然后你看这舆论就带就一开始是被那个笑，大家都觉得是校方有问题，舆论都被带跑偏了，大家都在骂学校什么的。

01:29:46

其实我觉得就经过我这么长达19年的这种思想上的这种影响，我看到这个事情的时候，我的想法是我觉得我是有那种批判性思维在里面，我我会想真的是学校就这样这么恶劣吗？我说有什么事情需要一定要去杀掉这个孩子吗？我我会？先打一个问号，就我不会因为看了评论就说这锤死了怎么样，包括我舍友跟我一起讲瓜什么的，都说什么垂死什么的，你真的锤死了吗？你是当事人吗？有些东西其实并不是我们看到那样的。

01:30:21

要有点辩证的那种感觉，你应该好朋友，就是你觉得适合觉得哲学好朋友，他的家庭环境也是这个样子，你有了解过吗？她的家庭环境的话，她我知道她的爸爸是哪一大老师，然后也在这个楼里，我今天才看到他，然后然后他他的妈妈是教英语的就是高初中还是高中，我不太记得是老师，然后我觉得他之所以有这种思想，那就是他看的书是我的好多倍。

01:30:55

我觉得我应该已经算看书比较多的人，因为我非常喜欢看书，可能受益于我爸妈，因为他们俩也非常喜欢看书，所以我从小就很喜欢看书。

01:31:06

然后像尤其是我到考试也特别喜欢看书，然后像我那个同学我就叫他皮皮，因为他就他的小名，然后皮皮他就特别喜欢看书，也但是他看的书跟我看的书又不一样，我觉得我看的书就是那种比较正统可能，然后但是他看的书就非常的叛逆。

01:31:31

他看的那些都是像西班牙，然后葡萄牙那边的一些小众作家写的书，我都没有听过的名字。

01:31:38

然后像我一般看的可能就比较的大众化嗯，可能看的比较多，但是涉及的面积可能就没有像他那样广，他看着又多又杂又广，然后我又一直觉得他的思想境界要比我更高一层，他真的很适合去抢钱。但是我我我就觉得他如果学哲学他就不会幸福，还是幸福，他就不会幸福幸福，因为我都怕他自杀，他想的太多了，我觉得。

01:32:12

你说为什么？有那些人都觉得这个世界非黑即白，因为中间那段因为非黑即白简单，中间那段灰色是复杂是痛苦，你如果一直在复杂的这一段区间里面，如果一直在复杂的这块灰色领域去看待这个世界的话，你就觉得你什么都看不透，可能我层次还不到，你就觉得什么都看不透，然后你就觉得好像都很痛苦，比如说如果我悲观一点，我就觉得我转到国中就一定好吗？

01:32:44

我找到国中我的未来就一定会比在医生那边好吗？你对成功的定义又是什么呢？就这个问题我前几天才跟我另外一个国中的同学讲过，他他就想得很简单，所以他就比较快乐，像我有的时候想的多一点，我就会很痛苦，你个人对成功的定义是什么？我觉得我觉得我对成功的定义现在来说的话，我能够实现自己的价值，可能我在某个地方某对某个领域甚至对某些人有一定贡献，我觉得我就挺成功的。

01:33:18

但是我觉得回到你之前问我为什么会就对。对。国中班有这种情绪的变化。其实还有一个很重要的原因，就是除了他们匿名骂老师的事情，让我觉得他们就是一群有一部分人是有那种闭塞思想的人以外，还有我觉得大家对于成功的想法，还有价值观其实都是不一样的人。

01:33:48

大部分的同学我觉得他们非常的看重考试，你说我要是看重考试，我的确看重考试，但是是不一样的，我觉得。

01:33:58

像我是把成绩考试作为一种实现自我的手段，我不会因为我这次考试考得很好，我就非常的高兴也，不会因为我这次考试考得非常的不好，我又非常的难受，但是我有些同学他们就觉得他们的成功与否就是有没有拿到奖学金，有没有得奖，成绩有没有变得更好，看这点有没有poss之类的。我觉得他们可能这样子奋斗比较简单，也比较容易快乐，但是如果是我的话，我这点破4固然好，但是我我就觉得并不只是这么简单，就是我我是这么想的。

01:34:36

我有一个小问题比较好奇，问一下，你说你周围同学比较想要去争奖学金，你觉得他们是对奖学金的荣誉比较在意，还是对奖学金的这个钱会更在意还是都有？

01:34:49

我觉得因为国中版也有奖学金，但我估计这个东西对你可能没有那么大的吸引。我想知道这个还好，吧还好怎么说，我觉得晋国中的同学对他们我了解到的，因为我们现在没办法评额外奖学金了，他们都是在大一的时候评的，然后你们大二就不能再评比，对大二就不能再评，但是他们对于很多事情都非常的准，比如说我们今天发了一个实验操作技能的考试，当然我想到这我还没洗，然后他们就这种小的考试都想到要卷到100分什么的，我觉得有的时候没有必要，这样子可能就是三观不一样。

01:35:33

他们对于奖学金到底是对这个奖更看重，还是钱更看重的问题，我觉得应该都是两者都有的，看他们的家庭，你们不能再评别的奖学金了，他们为什么还一定要考的那么高，对，这就是我疑惑的地方。

01:35:48

我如果这个问题如果你问我，我为什么都进国中了，能拿奖学金了，你保研直博什么的基本上也都这样了，因为我们的保研直播条件是比较宽松的，然后我觉得正常国中的同学都是能够达到的，为什么还要就这么卷？

01:36:09

我觉得如果是我的话，我这学期是真的开始卷了，我是能感觉到我自己在卷，我是被迫开始选的，因为大家都在卷。

01:36:18

首先是环境，你这个班上大家就是成绩普遍在三点几分，而且你像我我们，班上个学期的均分是3点7左右，但是你大家都很厉害，但是你真正拔尖的破4的同学只有一位，也就是说我们班的现状就是拔尖的少，优秀的多，但是在这种情况下，优秀的人都想成为拔尖的人，大家都会就会开始就卷起来了。

01:36:51

没有办法大家都卷起来了，我如果躺倒的话，我很有可能都连40%都保不了，所以其实对你带来一定的困扰。

01:37:01

这个环境有的时候是有的，但是你说他要非常的困扰我的话，好像也不至于让你压力最大的一件事在国中班。

01:37:13

不是，我觉得我在国中班压力最大的事情，应该算是我平衡不了科研和学习这两者的关系。因为像我们国中的话，它不仅是要学习，它还要实验室里有科研的任务。你说其实我觉得我们本科刚进来，对科研其实是了解不多，瞎子摸象，很抽象的，我们也不知道就看文献，你说老师说你要看文献，你看那么多文文献，你仿佛懂了，其实你根本就没有懂。

01:37:44

我觉得这个东西它是应该细而精的，就是你确定方向之后再往那个方向去钻研，可能会比较好。

01:37:52

然后但是你像我们现在又有这种实验的要求，我们而且它是有硬性的次数要求，就是你一个星期里面至少要去三次。但是你像到考试的时候，背书都来不及了，你现在要再去，我觉得压力会比较大，这个可能是比较困扰我的。

01:38:11

然后像比如说像那些同学比较卷，或者是有些同学可能思想素质不是很高，我觉得这些对于我来说顶多是有点影响，因为不可能造成困扰，我觉得人还是独立的个体，他们怎样？其实我是觉得关我什么事，我只要做好我自己就可以了。

01:38:33

其实最主要的困扰还是你自身平衡科研和学习之间的关系的一个问题。下面一个问题是不是你有没有？我先问一下你轮转，轮转的话和你就是后面确定方向有关系吗？

01:39:05

有关系的。你现在选的导师是你当时沦陷的其中之一吗？我还没选导师，我们要到大三才选导师。但是你说现在轮转是不是为了选导师？那肯定是的，因为轮转的过程就是在摸索你自己兴趣，然后再选实验室的过程。像我们这边有三个方向，就是精子发生，卵子发生，然后出生队列，然后除了出生队列以外，我其他两个都很详细的轮转过了，但是其实我针对也算轮转了，但当时不是写海鸥搞赛默飞的事情，你知道吗？

01:39:42

我知道，因为这个事情是我们后来封锁消息的比较严重的一次事情，就是我们查出来，对我们实际上都有新冠病毒，然后我们全去核酸了。对，就是这个事情，所以当那一次寒假我们的轮转学习就直接被取消了。

01:40:01

所以我那一轮其实报名的是出生队列的省校的实验室，然后本来都要去学习的，然后吹了，因为不让你留在学校了。什么叫不让你？是因为他对有三部分事情就不让你在学校继续学习，就是你本科生就直接放寒假回去了，然后后面也没有补，所以像出生就业这边是空缺的，像精子和卵子都是有轮转过的。

01:40:35

所以接下来还有一个小问题要问的是你现在已经是大二对吧？大二的时候现在有没有学弟学妹来问你，有关于活动班的事情，有今天还有今天正好有一个学妹来问我，他们问的是主要给你印象最深或者问的最多的问题，大概是什么？大概基本上都是学姐你转成国中成功了，我也想管转国中有什么好的建议等这个问题怎么回答？

01:41:07

很难回答，所以我给他们的我现在这个回答已经有套路了。

01:41:12

不是，我首先会问他们你的专业排名和绩点大概是什么位置，有没有合格，对，首先要问他们有没有达硬性标准，然后如果有我就说你接下来这个考试先好好考试，你考完试再来找我，然后就直接推走了，然后没有达到的话，我就说没事的再继续努力，你这个学期考完试再找我说你弄了，等他们到了，你会给他们什么建议呢嗯。

01:41:43

看他们问我什么吧。如果我觉得股东班比较适合什么样的水底学，我觉得比较适合，我觉得肯定是真正有兴趣的，对吧？你不然你没有兴趣，你就为了我当时其实不是不你说我要真的要逃离一正是，我的最主要的目的以外，我为什么就一定会选择国重，我其实完全是可以放弃这个机会的。

01:42:10

其实还是因为我对的确是还是有一点兴趣的，所以我才会觉得我转过来之后，我会努力坚持下去，就在这个地方这样子学什么的。然后我觉得你说你问我会给他们什么样的建议？

01:42:26

首先我会问他们到底是有没有兴趣，真的想好要转了吗？然后我觉得如果他们真的要想好要转了，我就觉得你应该抱有那种怎么说背水一战的气势，对，我当时我考的时候，我就完全没有想过，我还会回忆着我就拼了命的看。

01:42:47

你想2本那么厚的书，我又花了4天，我现在回想起来都有一种就有种那种就很英雄主义的色彩，然后怎么说，当时其实真的没有想特别多，我跟每一个想咨询我转专业的学弟学妹，我讲的都是你的目的，一定要单纯。

01:43:06

你要想转，你想好了要转，那你去努力就去做，你就背水一战对吧？你就不要想我要转不上怎么办，要怎么要不要想着寻求，现在就开始寻求我的安慰。

01:43:18

我是觉得他们在给你寻求安慰，有的是有的，我觉得有些医政的学妹，因为大部分咨询我还是医政那边的学妹，给我一给我一种他们不喜欢医政，想要去更好的平台，然后感觉从国重好像又因为他们觉得我考得很轻松，因为我也不知道为什么，可能是我的原来同学给我宣传的那样，他们觉得我考得很轻松，其实我考得非常的痛苦。

01:43:50

然后他们就觉得要问问我，国重感觉你考得还很轻松，有没有时候敲门什么的。

01:43:58

其实他们可能想有个诀窍什么的，对，然后包括想要了解一下国重要怎么考试，考试考什么内容，考哪个科目，然后比如说呃面试会问什么问题，然后还有没有什么额外的测试什么，可能就咨询一下。反正现在我还是咨询一下整个过程，对是的。

01:44:20

然后主要还是怎么说呢，想要来问问我的就是那种有没有希望转那种感觉懂想想寻求一种精神上的安慰？

01:44:33

我反正每次基本上都是讲的，你们这学期好好学了之后都有希望，然后考完试再来找我。

01:44:41

嗯但是你说我现在要跟他们讲那么多，把未来描绘得很好，其实对他们是很残酷的，万一考不上，万一我说的这些对他们没有帮助，不都浪费彼此时间吗？对吧？你觉得国中班学了一年下来，有达到你当初进来的预期吗？有。有，因为我是到二进来的。我觉得我其实是想在班级里遇到更好的自己。

01:45:12

我觉得你说我从到二到第14名，其实是因为我上学期节点，而且我上学期我我都没有觉得我只是很平常的对待每一门科目，然后都能考到第14名，我觉得我是非常满意这个结果。然后怎么说你也可以让你保研了，肯定是可以的。我我觉得应该是可以的，我没有了解过，因为应该还行。季节其实你要说保研这个事情，我其实是对国重政策，这也是我比较不满意的地方，他非常的不公开不透明，他说你达到前40%，那是你这个季节是哪一种算法的40%，就有个非常好玩的事情。

01:45:56

在这个学期刚开始的时候，我辅导员给我发发信息，就这个学期刚开始3月份三四月份的时候，我辅导员给我发信息，说你纪检不行，我当时一一听我都愣住了，我说为什么？我说我国中这边有很多专业课，我都95 97分这种分，你怎么可能会是他说我是倒一，我说怎么可能是倒一呢？然后他就给我发了一个表格，说什么就看似好像是道义，因为我姓赵，然后最后一个其实他有比我借鉴低的还在我前面，然后他可能没有看见，然后我就非常的纳闷，但是他绩点跟我自己算的，还有我学位算的都不一样。

01:46:38

我当时就觉得你没有统一的标准，比较怎么去画40 40%的线，我就打电话给教务处的科长，我就问他你这个线到底是怎么画的，然后他就跟我讲说这个线有三种不同的画法，我当时就觉得非常奇怪，我说你说说有哪几种画法，他说第一种是你学什么什么毕业的节点，就只算主干课的绩点，然后另外一种是你保研的绩点，就是你主干课的绩点乘以70%加上非核心课程的绩点乘以30%这样子加权计算。

01:47:21

然后还有第三种是你们国重自己还没有有那种算法，机器就是程序出来的那种算法。所以说你们的算法还没出来。他只是口头承诺过了，但是其实正儿八经我要到官网上去查的话是查不到的。

01:47:38

所以我就觉得很非常舒服你，刚刚说的主干科70%，非主干科30%，这是针对国中班以外的保研的学生，对，因为你像我们画40%是怎么画，是跟预防专业或者是基础专业的同学去画，你像我们的课程都是不一样的，你这个划线其实就不公平，是吧？所以我们其实我觉得每一个国中的学生想去查绩点或者是争40%的线，因为我们已经开过好多次争论40%的线问题会了。

01:48:11

然后这是老师主动给你们开的吗？还是我们要求的，因为我们17 18 19三届学生都要求过这个问题，因为他们年年都在变化，然后上次就因为这个线一直在变化，所以17和18都有学长学姐陆陆续续退出了，觉得保研政策太不明朗，不仅不明朗，就有一种给你画大饼的感觉。

01:48:34

就是因为在17级的时候，我有了解到他们的保研政策是不看你学科绩点，仅看你 sci的发表文章的问题。

01:48:44

比如说我有一个很好的17级的学长朋友，他很厉害他就发文章了，但是他现在新出的政策对于他来说是非常不利的，因为他并没有去抓自己主干课的这些绩点的问题，而是去发就是把重心放在了发文章上，就导致他文章发了，但是g点达不到。

01:49:06

但现在的新的政策就是不看你的文章，只看你的绩点，就很离谱啊。像你刚刚说的学生他退了吗？现在还没退还。没退。因为你像我们现在国重只要6级过了，英语是不用上的，就是那个课。然后再加上我们自己像有生殖生物学，然后有高级科研实验，然后初级科研实验，然后还有基因x然后生殖生物学前沿论坛，这些都是我们自己的特色课程，也是核心课程。

01:49:46

你说我跟预防的同学他们都不学，然后去画这个线的话，真的让我这几门课程学得很好。其实是不包含在我的纪念里面的，这也是为什么我辅导员上次给我发信息说觉得我绩点不高，那是因为我这几门考得非常好的都没有算进去。像那些进来又退出去的，其实你们那些特色课程、叫什么、生物学都已经全部作废了，是这个意思。

01:50:11

你也没仔细过。

01:50:12

我是，我记得他上次有上课，就是开会的时候讲说可以那种课程认定，但是你说你这个课程认定认定下来之后，肯定还有很多是需要自己再去补的，所以我们现在就国政办一个房间流程认定，哪些课能认成，哪些课就有具体的方案，出来啊有的。

01:50:32

其实他上次是，但是其实你说要在官网上要查到这些是没有的，只是我们上次 P有的那是，你说到底是有还是没有？

01:50:43

所以你们房间流传什么，我们房间流传就是要退国重要挣走，要不然你后面要补课太多了，因为我们的课都是不一样的，到大三基本上全是专业课的。比如说大三的时候预防他们要学自己的专业课，我们国中也有自己的专业课，那就不要补死，退掉之后相当于重新复读了，你那你有没有想过要退？

01:51:06

我觉得怎么说呢，我我就回去了，我不用回医生在预防就是预防。这怎么说，我觉得目前来说国用的平台对于我来说还是有利的，我就不会想退，而且你说我现在不想退，我大三就更不想退了，因为我大三要退我就要读大二的课程，压力还是比较大的，你说那些退的可能就是大二的是吗？你的同学我同学现在目前没有退，大三的好几个学长学姐都退了，就是在进大三之前应该退掉。对有是的。宁愿补课也要退。对他那个政策的确。

01:51:45

然后所以其实你们像刚刚我说你说的保研的会是这个学期开的吗？还是上学期开的？上学期开的上学期。其实他们讲的你们就是你自己的感觉或者你对同学的感觉，其实他讲的话，其实你们不是很认可，就隔壁的科长讲，上次我那几个学姐把他怼的拿着话筒，他说我们在稍作讨论再告诉你们，后来有真的告诉你们吗？有告诉他把胡笑都请过来了，无效做了什么承诺，无效就承诺到华华县到40%，就保证你们一定保研，那就是40。

01:52:28

就像你刚刚说的其实就是很不透明，现在的意思就是说只算重叠课程其实也是非常不合理的，会不会你就讲你自己个人感觉保研政策的不明确，会不会让你或者你的很多同学有退出的想法，其实是一个非常主要的原因。

01:52:46

是的，而且他这种政策一出来之后，就是国中班一些卷望，直觉那些重叠课程，把那些重叠的课程再修得高一点。不是我们现在不能重修了，比如说我这个学期4门课，ABCD预防4门课，bcdf他就只卷bcd了。我明白意思，然后他AA级是核心课程，他们也就不想学。其实我觉得你要跟预防去画这40%的线，是非常奇怪的。怎么说呢？我也不懂学校当时的这种政策是怎么样的，因为我当时考进来之前，他的政策，我记得是他只要没有挂科给你保研的，没不存在于预防或者基础的学生画线的这种说法。

01:53:46

就很奇怪，我觉得其实你其实跟学校有关系，学校之前整个学校层面的保研政策在变，之前是有那种特殊保研通道，现在好像要把通道给收紧，对于更公平一点。

01:54:05

所以其实有影响到你们的保研政策啊，我觉得我们的保研政策其实比如说改成你平均季节就得达到3或者是3.5这样子，反而更加合理，就是你硬性卡一个线，你不要去跟什么跟其他的什么学生，就跟我们专业学的东西都不一样的学生，就是说画一条线4040%看看似上上去是有点公平的，但实际上我觉得是没什么逻辑的。

01:54:34

因为你本来就是学我现在说出来是预防国重，但实际上我的课程就是国重，只不过我的本科的挂名挂在了预防这边而已。

01:54:46

你说我因为本科的挂名挂在预防这里，或者甚至有些倒霉的国中的同学，本科的挂名挂在基础那里，那就非常的惨，其实如果他们挂在基础那里的话，因为基础权利因为他们太卷了，真的卷死了的课又多，又卷。

01:55:05

基础活动比你们课更多是吗？因为现在其实这也是分配的不合理，因为像我们真正要学的课都是一样的，因为都是国重，但是他们要学的课就是安排的不是很容易，他们就把很多很难的课提前都安排给他们，然后比如说他们这学期学，其实这节课可能你们也会学习，你们放到后面对我们的课程是比较平均的，他们后面就比较松，主要是科研时间，其实你说说出来都是国中班的，为什么要有这么大的差别？

01:55:35

对吧？

01:55:37

而且其实还是跟刚刚讲的一样，你预防果重的同学跟预防的同学去拉线，不是很合理的，因为其实本来就不不会存在竞争的，你现在就搞得大家都觉得很奇怪，而且我们国中的学生是不参与任何排名的，我们只看线，所以有的时候也不是很难理解为什么他没有绝像你们这些事情会跟国中班以外的同学讨论，你们莫名其妙的保研政策嘛不会因为不会有共鸣。

01:56:13

因为我说的比如说跟我舍友我说他也听不懂，嗯他也觉得跟他没有什么关系，比如说你说说一阵的八卦，可能大家还是有点意思，因为毕竟还是原来一个专业的你还有啥？没有很好讲讲其他的。

01:56:42

我们应该没有什么太我觉得我已经问到了我要的东西，了好的，还有刚刚是还是私人的话，是你刚刚说那些什么非黑即白，其实我觉得你说的那一部分人可能触碰到比较社会上比较灰暗的那一面，可能又回到了觉得想回到比较简单的思维模式上，我觉得可能这些人可能还是少数，更多的人可能是他没有能力去思考到这么多的东西，有可能说你说的家庭影响就是要思考到他也没有办法，或者说从他的个人的，价值观上说，他没有办法去接受这种事情的存在，或者说或者说对于一个普通个体来说，非黑即白的思考模式是最简单的、最省力的最轻松的，我觉得大部分人应该是是这样子，个人个人的是的就是他这种思维模式让他活得轻松简单，不用想那么多事情，他就以这种模式和。
