# Supplementary material for: Why do you choose this program?—A decision-making model of medical students based on grounded theory
Source: PLoS One. 2023 Sep 15;18(9):e0291634. doi: 10.1371/journal.pone.0291634 (PMC10503722; doi:10.1371/journal.pone.0291634)
Supplement: S1 File — (ZIP) [file pone.0291634.s001.zip › RAW DATA/P9.docx]

00:01

can start, hello. We are teachers of the Institute of Education, mainly want to do a project, so we are conducting an interview. Our main purpose of this interview is to restore all the links from the publicity of the activity class to the registration to the admission. Listen to the things that have a great influence on you or things that have a deep impression on you, including your own feelings, your emotions, you can talk to us about these things, and try to talk about your thoughts and everything that affects you. .

00:31

Before starting this, I need to read the experimental ethics guidelines. In this interview, the interviewees participated on the principle of equality and voluntariness. Interview conditions, the interview process will be recorded, but the recorded data will only be used for scientific research in an anonymous form and will not be disclosed to any third party. During the interview process and after the interview, you have the right to cancel our recording. Do you know and agree to the right to use the data?

00:59

Agreed, well, please tell us which level and which major you are in before we start. I am a level 19 prevention national. 1972. Did you also prevent it in your freshman year? Or not, I was in the School of Medical and Political Science during my freshman year. You are really right. Why is it really the first question we want to ask is to ask how did you learn about the public classes in your freshman year? I think it's a very accidental thing. In fact, I think that the publicity of the middle school may be for the students of the medical and political college, and his publicity is not very good .

01:48

The reason why I knew there was a middle school class was because at that time I saw that many of my classmates had changed majors and left, and like our current class of doctors, most of the top 20% have already transferred. Then I watched a lot, because my grades were not bad, and some of my classmates were not as good as me, and they also transferred, but I didn't change majors at that time, I felt very sad , and then I went to the school every day. Check the website of the Academic Affairs Office to see if there is any opportunity to seek it. Then, by accident, when the school started, I remember clearly that it was the afternoon of September 3rd. I was informed, and then I thought that although the time was very tight, because it was the 10th, the test was going to take place, and if I had to prepare for it, it would take less than a week, and I didn’t have any books or materials yet, but after thinking about it, I still had to try it. try.

02:44 I

changed my major anyway and left. Probably so. You said it happened by accident. You said that many students have transferred in the first 20%. They usually transferred to some major, and those with top grades went to the clinic, and then the preventive pharmacy, and then the scores were relatively high. In general, I went to the basics, then the voice tube, as well as nursing, and English.

03:17

You said that you are very uncomfortable. Have you ever thought about why you think they transferred to a major that you think is better than management? Because I actually wanted to enter the major at the beginning , why did you Thinking of changing majors? Because my subject is limited. You are liberal arts, and you can only report English management. what else? care. Yes, but I went to see Guozhong specially. In his admissions brochure, he wrote that there are no restrictions on that kind of disciplines, and then I thought it was an opportunity.

03:51

So at that time, if you changed your major to choose prevention, you might not be able to. Is that what it means? It’s not that I can’t transfer, it’s that I’m not qualified to transfer, I’m not qualified to transfer. It's just the conditions to hack you to death, but if you have such an opportunity to go to me, I want to know how the process of applying for a major in high school was like. You were not a very tortuous thing at that time.

04:21

How do you say I'm from Nanjing, I don't know if you know I'm from Zhonghua Middle School, I know, and then our class was very special at that time. He was in the first and last semesters of freshman, and he was divided into classes at the beginning of the next semester. , that is to say, the normal class placement started in the second year of high school, but we only took one semester, and we started class placement before finishing the school year. Then, what I said on my grades at that time was very historical. Top-notch, history, history, politics, history, geography, and chemistry are very top-notch, basically the top few in the grade, but my physics is very poor, I can't say it's very important, but the trend is very worrying.

05:05

The first time I took the test, there were more than 100 grades, the second time was more than 200, the third time was more than 300, and the fourth time was more than 500. The trend is not optimistic, yes, then my teacher Maybe it's good for me too. I applied for science at the time, and he said that it is very advantageous for you to apply for liberal arts, because my political history is in the top few grades, and then he thought that on the one hand, he might be able to advance to the school. On the one hand, he thought about me for himself. He thought it would be better for me to apply for liberal arts. Anyway, he was persuaded later, but I was actually very sad for me at the time, and I went home and cried to my mother.

05:44

But why do you want to accept the teacher's opinion? You say because I wasn't very optimistic about my physical condition at the time. I think you may still be interested in him, but you may be very interested in me if I don't choose well. It’s hard and hard, but like political history, let’s talk about an absurd example, that is, the night before the model, as long as I only have to take the political history test the next day, I have been talking with others in my evening self-study. , and then the next day I was still at the Shuangyue home, which may be more suitable, but I don't like it very much. Although I was very relaxed in the process of his learning, I don't think I experienced a lot of fun.

06:26

Did you ever think about switching to science when you were in high school? Studying later, including when you had a small college entrance examination? Just those 4 doors. When you were learning those 4 subjects, didn't you think about turning it around? Just can't turn around. We don't get stuck in our school, and now I know that some of my college classmates still choose a combination of history and chemistry, and I feel very strange.

06:55

So you chose liberal arts at that time, and the school won't let you transfer. If you don't let me turn, it's like being kidnapped. Our high school transfer card is very tight, I think.

07:08

So in fact, I personally think that in terms of subjects, the choice of majors in the following is actually very different, but you say whether it is necessary or not, I think I don't think it is necessary, just like I have seen things like the United States before. In terms of education on their side, although there are arts and sciences, the arts and sciences are completely different from those in China. I think one of them is biased towards those kinds of theoretical arts, and the other is biased towards It uses practice, but you said that these two are different from the Chinese ones. I don't like the Chinese way of dividing subjects. In fact, because I think you are a liberal arts student, for example, it seems that after you enter the liberal arts class, others Just give you a subconscious that seems to say that your student is not strong in mathematics and science, but if you go to a science class, the first stereotype that people give you is that you must have low literary attainments. I feel that you may be very rigorous in your work, but um, you may think so without the talent of studying liberal arts, but in fact it is not. I think everyone should develop a comprehensive and comprehensive development. You should be stuck with liberal arts and sciences.

08:24

It's like, for example, I'm transferring to a junior high school. I may feel a little uncomfortable at first, because everyone is a science major. They may, for example, students' physical and biochemistry may be able to get started faster than me, but in fact, the final exam results come out. , I may be higher than them in the test, maybe my understanding of this thing may be a little deeper than theirs, I think I want to go back to when you were just in high school, you were forced to apply for a history, it should be called You also said that it can be said that being forced to choose liberal arts should be regarded as being forced to choose a liberal arts.

08:59

In fact, you are already very limited when applying for majors, because at least I know that our school may be like those science majors, and you may not be able to apply. When you applied, did you apply for management majors? Because in fact, it was my first choice to enter the hospital, because why? How can I say this? Actually, I didn't want to apply to Southern Medical University in the first place. My grades could have been better in other schools, but because my mother didn't really let me go to the first trial, I was even more restricted .

09:32

And then they think it's good to talk about the political network, maybe they can go to the health and health commission to take a civil servant exam or something, and then say that the way is better, and it is more suitable for girls, so I still listen to the opinions of my family, Reported. You said that you listened to your family's opinions more. When you were choosing liberal arts, you said that you cried with your mother. What was your mother's reaction at that time? do you remember? what did she say? My mother comforted me at the time, because my mother was a liberal arts major, and my father studied science, and then my mother felt that the arts and sciences were not important, that is, she actually meant that she could become the champion by doing her best, and she asked me not to be too much. Sadly, he felt that the liberal arts would actually adapt very well.

10:15

But how to say, I am actually a person with relatively strong adaptability. You say how painful it is for me to be in the liberal arts class, in fact, it is not just when I am a little limited sometimes, it will make me feel Heart is more uncomfortable. For example, applying for a major, yes, but in fact, I figured it out a little later, actually. What do you choose? Basically, looking back, it is unlikely that it is better to look forward. For example, when you are choosing a major in the arts and sciences, including when you are making relatively important decisions such as junior high school and high school, maybe your parents will give you a little more advice, right? They will give you a little more advice. Opinions, but in fact the final decision is still in my hands, but I rely more on listening to other people's opinions , and I am more willing to accept your parents.

11:05

Yes, because I think sometimes my judgment may not be very accurate, so I decided to study here in Jiangsu Province, and then I chose Southern Medical University, right? At that time, if it meant that your mother had no restrictions on where you could go to school, which subject did you want to apply for, for example, which school? I originally wanted to apply for Xi'an Foreign Studies University, and I wanted to study languages. Yes, because it's not that I want to learn languages, because it is already restricted after all. In fact, what I like more is that when I fill in a major after the college entrance examination, I prefer social sciences. However, in fact, I feel The future development of social sciences will be very narrow, and then I feel that language-related subjects are considered as liberal arts, which are slightly better for employment.

12:06

I am good at language and management, so I have some feeling, you and I are very similar at that time, really.

12:13

I also studied languages, so I chose this to consider employment at that time. At that time, you said that you only saw the counselor's notification on September 3, and then there was no information. You will take the test a week later, and you will take the test at that time. Is it supposed to be sissy or what? Xisheng and grouping. In fact, with my grandparents, have you ever taken a project in the first year of management major? I taught myself completely. You learned it in a week. I hope it is 4 days to be exact, and you have learned cells and embryos in 4 days. Yes . real. I understand me. Understand. Really, then I passed the exam. When you were in school, what did you mean when you said you didn't fit in well?

12:59

Do you think that you may not feel the thinking of science, or is it that knowledge is not what I think is that I sometimes have a limitation on myself. In fact, sometimes psychological hints are very important, why I am not good at physics, in fact, I have thought about it many times later, I used to be a better class in the special enrollment class of No. 29 Middle School in junior high school because, I Now the junior high school classmates in the class all go to Peking University and Tsinghua University, and they are all very good. Then, the ethos of our class can be said that involution is very serious in current words. Then, when my parents were in my junior high school, in fact, they always advocated happy education, that is, I never attended any remedial classes.

13:41

Then in the second year of junior high we were just about to start physics class and all my classmates seemed like I remembered that there was no one in our class who didn't have tutoring class except me.

13:52

I felt that I couldn’t keep up. At that time, the things at the beginning were very simple, such as sonic vibration or something. Then I saw that everyone was very active in the class, and then they passed the exam every time. Very good, they all wrote the homework in advance, and then I felt that I was under a lot of pressure, and then I didn’t take any classes, and I felt that I was much weaker than others. It is said that the poor state has never been dizzy, and the state of not being hot is very moderate, but after high school, it is even more painful.

14:27

Then all of a sudden it was very bad, and now after transferring to middle school, I always feel that the people around me didn't study materialization in high school, they studied biology, and even some things, I think They studied science, maybe they cultivated a kind of science thinking in the past three years, and then I may be defective in that kind of thinking, I always feel that because I am a liberal arts, I may be weaker than them, and then this kind of thinking will become Makes me miserable sometimes.

14:56

Yes, it is a very bad psychological suggestion, but you can say that you are implying yourself, maybe I didn't have this foundation at that time, yes, but so I say you better, he sometimes will It motivates you to roll inwards a little more than others, but when you go bad and say you don’t want to roll, you feel uneasy when you think about it, that is, you have a feeling of being weaker than others .

15:21

I want to ask in detail what happened in junior high school. You said that the students around you may have learned it in advance, and they are very familiar with this thing, because it caused a lot of pressure, and then is it because the students around you seem to have You learn faster than you, and such an environment makes you feel that you may not be suitable for learning physics, or that you are not suitable for learning, or that you are not good at learning physics, can you say that?

15:51

What do I think about my interpretation of this? Actually, I think I told you before that it is easy for me to study political history, but in fact I think I have reached a state of flow, you know what is it? Is it a state of flow? Flow, for example, is a person learning this mind? Yes, the flow of the heart is the flow of flowing water, then what does this state mean? In fact, it means that you can have fun in learning, and he can always give you something positive in return.

16:25

Then when you go to study again, you don't actually know that you are studying you and you, and you feel that you are enjoying the process. In fact, the final result is often better. Maybe I am not interested in learning. The liberal arts majored in political history. I may have been in such a state, so I thought it was easier, but in physics, I wanted to try to enter a state of flow, but I was constantly disturbed by others, so I became a One has a side effect, I feel that I can't have fun, I feel that no matter how hard I learn, I can't surpass others , maybe I feel that I have no talent, and then I feel very tired, and then I will be vicious. cycle.

17:02

I think that my state of studying physics is like this, um, maybe I have been under pressure from what may be called class ranking, which affects a state of your study, yes, and then I actually feel this way.

17:17

The most obvious one is actually when I was in the third year of junior high school, because I didn't have to add a new subject chemistry in the third year of junior high school, and then I strongly asked my dad to take me to the early kind of chemistry, just the summer vacation. Go to class early. It's scary to me, yes, there is such a thing as physics. Then, in fact, I didn't take long in the summer vacation, I took classes for a week at most, and then my chemistry was basically the first in the grade, and then my senior high school entrance examination was still the first chemistry in Nanjing.

17:44

That is, I think a psychological suggestion of people is very important. Because of the physics, I feel that I am preventing me from getting better.

17:57

So can I say that you still care about your ranking in the class? That is, when you actually study, you still want to prove your ability by passing the test higher than others or something. You will have a sense of achievement. Isn't that exactly like you said I want to take the test?

18:20

He is more concerned, but he may not be as serious as I said, and he is more concerned about a point. Yes, I will care about him, but I don't particularly care, and I don't think I'm particularly good at middle school now, I think they're better than me. Really they are too curly.

18:39

So, a counselor told us before, if you have ever thought about asking for students in the junior high school, because we are all from various majors, should we change dormitories and change them together? Then I don’t really want to talk to them. The main reason is that first of all, I was in Building 9, which is the best dormitory of Southern Medical University. I heard that the condition is that we have separate bathrooms on the bed and under the table, and it is very large, so it is very good, you know? Then if you want to change together, you may exchange for a dormitory with not so good hardware facilities. This is the first one.

19:10

The second is that I think if I share a dormitory with my classmates from high school, for example, if they study in the dormitory, and I am chasing dramas, and then I see them studying, I may not think in my heart. If I want to follow dramas too much, or I don’t want to study, I am not so happy chasing dramas. Can you understand? Are they the kind that are easily influenced by others? It doesn't count, I think I'm going to let you give yourself to yourself compared to this environment, yes, but you said, for example, at the beginning of school, if they were going crazy, there might not be any pressure on me, I should Watching TV or watching TV, should I play or play, if he is approaching the exam month, and they have already started the exam in advance, I will start to get nervous, it is conditional, in fact, we are motivated. I think, it's not that you and I actually don't want to roll them or anything, it's their invisible pressure that makes me have to roll up, in fact, you said I want to roll it, and I don't seem to have it. However, I think you still have your own rhythm and don't like being interrupted by others.

20:19

Yes, so I like studying. I used to have classmates in the same middle school and they liked to have a study group. For example, when we go to study together on weekends, I am not very willing to go with them, because Would such a study group help anyone? Or what is their purpose? Just make an appointment to study and accompany you. I think the learning atmosphere of studying library science is very good. I like a person very much. Have you ever understood why they do this, or do you think their purpose is to do it? What?

20:52

The study group may have a relationship, maybe everyone is at this table, I sit here and you sit there, and then we are all learning not to talk, I think it's actually very boring, I usually I learn and I learn Generally, I like to study alone, that is, a person’s words are not easily disturbed. Otherwise, for example, if he is leaving, he has to tell me. I am leaving, maybe I was focusing on one thing, and then he said that he wanted to Go, and then let me look at the message he sent me on his phone, and then I play with the phone, and then I pick up the phone and it may have been 45 minutes.

21:25

There are indeed some classmates who like to bring everyone together. Yes, some are like graduate students holding group meetings, and some classmates and graduate students also like to take their classmates together. Surely they just want to see your progress and do it. Where are you, you have this kind of thinking now, he wants to grasp the people around him.

21:46

I understand, it depends on how well the people around me have learned. I don't like it very much. If, for example, the immunization test was completed last week, and then the final exam was taken, some students would ask questions everywhere. You have read the book several times, and you have also brushed the question bank. Do you understand me?

22:08

Are you over the top? Then when he asked me, I was very nervous, and then I didn't know how to tell him, and in case he was more enthusiastic, maybe then he would tell me who I listened to, and I had memorized the book several times. The books are all rotten, I feel very nervous when I hear it, and then I think what should I do if I haven't read it this time, and in fact, I really don't like receiving this kind of information, I feel that I need to protect my ignorance. Emotional rights, it makes me feel bad when he forces me to know, and then it affects my rhythm, yes yes um.

22:37

But in fact, I think I can control my own rhythm when I take the exam.

22:44

About two weeks in advance, I knew that I might start reviewing something. No matter what they told me, I would at most be nervous, but you said that once he told me about his book, he read it twice, I just said that I must finish reading this book today, which is unlikely, because I feel that haste is not enough, you may just see that the progress is over, it is actually useless, and it may affect your mood. , but your actual actions and your plan will not cause this, it will definitely not, because we make plans every day, I am very clear about what I want to do. When he talks, he interrupts my plan. The plans are all scattered. When did you start making plans every day?

23:19

Junior High. Did your parents make you do it? Or you are not. I think this is the biggest thing I have learned from my good friend of my Peking University classmate. He is a very planned person. It is the elementary school he teaches you, and it is not because our methods are different. Like he lists what to write every day, and I think I prefer this time period, for example, today I have classes at 9:40 in the morning, and then you see I will be interviewed by you in the afternoon, just 2:00 , and then at 4:30 in the afternoon, Mr. Li Chaojun wanted to call me in the past to discuss our innovation plan, that is, I should draw the dead time first, and then the rest of the time would be my spare time, and then maybe I will arrange some things that I think need to be done in the near future in these spare time, and then it will be easier to control. If he is a very efficient person, I am not very the same as him. I am the kind of person who is forced to be efficient. For example, if I want to take an exam, I have to be efficient. I may be very efficient.

24:22 In

a normal time like this, I have to list the time I control, and then adjust some dynamic plans. I may have a more fulfilling day. After all, I am a person who has watched cells and tissues in 4 days. You Another question for me is that I know that you studied liberal arts in high school, and then included management in your freshman year, and then at the end of your freshman year, in September, you suddenly switched to studying national weights. Did the parents discuss it?

24:54

This is a very extreme discussion. I was very excited when I saw the opportunity to change majors. Then my mood was very complicated at the time. I felt that I felt hopeless because I I know that many people must have known the news in advance. I think he said that the exam is going to be on September 10. How could it be possible that this happened on September 3, right? Nor can there be a normal person for these 7 days.

25:26

I don't think I can prepare very well. In fact, I am very clueless. I don't think my foundation is particularly good. Then there are two subjects that I am completely unfamiliar with. The entanglement, I called me at the time, my parents told them, I thought I was facing a very important decision, I told them, and then they were frightened by me, because when I talked, I would I cried, because I thought that I really wanted to be in medical administration, and then this is another opportunity that I can seize now, but can I seize this opportunity?

26:07

Actually, it’s not necessarily, because if I knew about his news in June or July, I think I must be very stable, I think I will definitely study hard, and maybe I won’t be so anxious. Whoops, so you cried mainly because you thought it was too late to know the news, and then I thought how to say, I am very unsure about these two exams, I am not sure, but I hope I succeed, so I feel very inwardly. The pain and pain of my life, and then I was very uncomfortable , and then I told them how to say it, but they still let me decide, because they didn’t know much about this field first, because my parents actually had their work related to medical and health care. They are all from the State Grid, and it doesn't really matter, and they don't understand, so they think I should think about it myself.

27:03

Then my mother asked me at the time, why did she ask me, how much do I really want to change this major? Then I remembered it very clearly at that time, I said that I really wanted to change, because I didn’t want to stay for a while, don’t be connoted that he doesn’t care, he doesn’t care, you don’t have to worry, maybe it’s personal and then I’ll just follow My mother said, I said that I really want to leave, and then she told me, he said, do you know what the future employment direction of this major will be?

27:37

Then I said that I didn't think about it so much at all, and then he said whether you thought about it too hastily, but then my mother asked me again, and she asked me why she switched me to major ? In fact, my purpose at the time was very simple, that is, I just didn’t want to stay for a while because first of all, I felt that the college did not make any evaluations of teachers, and I felt that the atmosphere of my classmates made me very dislike.

28:03

Then I really think that some of my classmates are like a cult and they are very scary, and then I am very, very true. I tell you it is really, very scary. How should I say, this paragraph must be kept secret, I don’t want to be found by the cult leader. I must be very curious about keeping it a secret. I’ll say what I tell you. I have a roommate next door. I don’t mean to be black in any area. The bathroom is independent, but the bathroom is connected, and then I know.

28:36

Yes, then he is from Guangxi, and then he is from Dong nationality. Anyway, I really think his quality is right. I just wash my hands with them, because he washes clothes over there, and he will scold people there every night. Up to now, as long as he comes in, it's his mother, and another classmate of medical administration. I don't know how they can have such a deep hatred, whether they scold the next dormitory or whether they scold the 4th floor in their dormitory, I I was on the 5th floor, and then I scolded the 4th floor. I rubbed my clothes and scolded all kinds of ugly words. I don't think I have heard so many colorful swear words in my life, and then elaborate some negative emotions, yes, that is It's very terrifying, and their intrigue is very serious, and they are not really capable people, so many of the things they say are nonsense, nothing.

29:32

As soon as the upper and lower lips touched, they made up something, and then they said that the hammer was dead, and I told you how to guarantee this melon, and then I actually thought it was a matter of your dormitory next door.

29:43

There are also other classes in the dormitory next door to me, and then my roommates will tell us a little bit, but in fact, some things are told, I am not saying how my roommates are, I may be in the same building as you. Lou, this kind of rumors spread all over the place, and also because it turns out that I am a positive person, they will talk about some, uh, not about me talking about other students, including the teacher, some very boring things, and then gossip little Gossip, and then there is a classmate, say he is studying something, he is just going to hit you, understand?

30:18

I started studying before that kind of exam. So anxious about this. Do you feel comfortable listening to it? It would be hard, I think.

30:26

and then there are some that may be annoying, but I think this phenomenon may be more common, because I also saw it in the middle school class, a classmate has some materials when he is studying, and then he will come over Can you send me a copy of this information? That’s all. In fact, sometimes it’s just a purchased material, and he would say prostitute you for free. I think this is a bit annoying. I don't think so, right. But it didn't happen to me, my roommate told me.

30:53

Because I am a person who does not have a lot of information. I basically only read books. You switch majors mainly because you don’t like the atmosphere. The biggest reason is the atmosphere. Can you transfer to the dormitory? ? No, in fact, the first is that I don't like the atmosphere, and the second is what he said. I don't think its prospects are very good, and I don't think the employment prospects are very good.

31:18

Third, I think the school doesn't pay much attention to majors. And what about the fourth one? Fourth, his faculty is actually quite average. I think this platform, I feel that I am limited, and I feel that I am limited in this major not because I am not good enough, but simply because My high school was liberal arts, I was restricted, so I was very unwilling.

31:46

Then later decided to switch to national weight.

31:50

When did you start thinking that I wanted to change majors, I strongly disagree with that question. Do you have this feeling gradually, or is it something like a Dong nationality student that makes you feel that way in an instant? How to say, I don't think I had a strong desire to change majors when I was a freshman. Why? Because first of all, I think that I may not be able to switch majors, so if you say that if I make myself miserable, I wonder why I can't switch.

32:19

But after all, you still think about me at that time, I will stay here for 4 years, if I keep thinking about why I can't be like this, I am too painful, I might as well accept this major, I am in In our major, for example, I will be the top few in the grade, right?

32:33

Then but then I joined the student union of the college, and then I thought the student union of this college was really outrageous. Maybe I don't know if it's like this in every college or this kind of student organization.

32:50

I think I feel that what I help the teacher do white work is a tool. First of all, I will not improve myself. I am helping him with his work. For example, I am in the technical department, and then there will be activities in the courtyard. I have to take the camera every time to take pictures, edit, and make videos, and I don't get any benefit, right? Sometimes even if you don't do well, the teacher thinks you can't do it well.

33:17

This and then I think when did I suddenly wake up, it was on December 31st, that is, in 19 years, I was really angry at that time, you may I am actually a very personable person , how to say he doesn't provoke me, and it's impossible to treat him like that.

33:38

My teacher asked me to accompany him to work overtime at the Student Affairs Office on December 31 on New Year's Day. Do you know what I am going to do? What am I going to do for him? I think the p in the year-end assessment is very outrageous. This is obviously his own business. Is it the counselor teacher? It must be a counselor. I know I have been a counselor. There is also a secretary of the college. He seems to have passed away now. Yeah, I think I knew he had been transferred, and I was really pissed off that time.

34:12

Mr. Lin Ming and Mr. Gao are very good. Mr. Lin Ming really I think sometimes he may not have a bad heart, but what he does is some very low emotional intelligence.

34:22

I just told me that you have to stay today to help me finish my PPT, he means you have to stay, yes, I feel very strange, what were you in the student union at that time Location, I am a very ordinary technical department officer, why must there be an officer?

34:37

Because he may think that my ability is relatively strong, because the planning I have done before is better, and I have encountered it.

34:46 Omg

, don't you know that I have been helping him for the last month, what is the defense department's mentality, and what is fraud prevention? I have done all the things that the secondary training unit should do. It's really strange. I make PPT videos online, I make the content of the security department, and then I will give you a speech. What is the teacher's commitment? It was written by me. It is really very strange. You may not have been in that environment. You may think that some of the things I am talking about may be very bizarre, but it is really happening.

35:14

I am from the technical department. After I entered, I felt that I might just need to take pictures, do art work, or even check videos. I think this is within my scope of work, and I am willing to do it.

35:25

But you say that I want to help you do your year-end work assessment, isn't it too inappropriate, and I don't know what work you do at all, how can I blow it like a flower yes, right?

35:37

Then he asked me to make sure to show it to him, and then with me was Senior Xing Chaoyang, just me and him, he was a master, what did he seem to be? Is the leadership of the Youth League Committee the same as the student leaders?

35:52

He was a senior, I remember he was a senior, should he be graduating now, I don't know, and then he asked me to do it. I was very angry at the time, and I was in class at the time, I was in advanced mathematics class, he Send me a message and tell me to go to his office immediately to help him make these things, and I think it's weird, and then I, anyway, I turned off my phone in a fit of rage, and then I went straight home because I was Then I went home from Nanjing. After I got home, I turned on my mobile phone again. I saw 17 missed calls, as well as Gao Yukuan, seniors and seniors, Zhang Yuxun and so on. They all called me.

36:29

I said why are you calling me? He said you hurry up and go to the school, and if you don't go for a long time, you will be finished. Then I said it wouldn't be over, I'm going to be on a statutory holiday, can I still take a rest?

36:40

I was very angry, and then I went to his office again on January 4th, and then he was there. I was really angry. At that time, he asked me to go to his office, and I found it very interesting. , He felt that he still wanted me to go to his office. After I went in, I said more and more that it was not you who took the initiative to go. He told you to go. Yes, he didn't say anything after I went, so I asked his teacher. Teacher, what do you think? What is the job of a student? He said that a student should study hard for his own job, and I said yes, he should study hard, so I have to start studying hard now. Then he slapped him anyway, and I left. He must have been very unhappy, but because it was still in the second half of the semester, the epidemic happened just in time for the semester. Then, in the case of the epidemic, there is nothing to do at home, which is similar to the student organization. I have nothing to do with it, so I basically quit, because I seemed to be quite high that time, everyone was a little scared, and everyone still Some of my other classmates might know it, right?

37:40

Because when he was in front of him, teachers and classmates felt that this person was very good, yes, he was very tall, and they thought he was very tall, yes. Because I think if you say something like my untenable, I think he's just nothing, I think it's a very outrageous thing for you to say it out, I'm too weak if I'm not tall, right? And because of him and my lack of effort in the last semester of my freshman year, I only got 60 points in the high school test, and I was very uncomfortable.

38:10

I just came out and got a score of 60, which means that you all scored 50 points in the test. The teacher put all your efforts into it, but you think your high number is because you are right, because I didn't have it when I took the midterm exam. Affected, I got full marks in the exam.

38:25

Well, after that, he often let me go during my class time. I don't have this, and it's a science thing. This kind of thing is before the December 31st event. You go during class, you all Are you going directly? do you accept?

38:39 When

he asks you to go to class, you can go. Sometimes, for example, for his more watery class, I may go to the class that I may make a fake note and I will go, but then he will go to the main class. I was more alert at the time when he violated him, because he was basically the kind of person that I might watch when the sun was shining when I went in, and the moon was already shining when I came out, and I thought this was too strange. , there are many words that are just a clerk, your whole department is there, not me and another clerk, very strange, I think when I was a counselor at that time, I always brought the minister over, and I never brought the minister. I will push the clerk over. I, I, I was too clear at the time. At that time, Mr. Li Min and I had a background editor. That number was an account of a graduate who went to work in the Drum Tower Hospital to study, and then he was a private account, we I just want to make it an enterprise version, so that I don't have to go back to the senior senior with the highest authority every time to ask her to report things. If she wants to, she can change it to a counselor's number when she reads it, because Li Min doesn't want to. Regardless of this, when we go to find Li Min, can the teacher make the number in the backstage your personal?

39:52 In

this way, when you change the term in the future, you don't need to find the senior sister from the previous term to come to you directly. He just said that I am deaf and I can't hear. Don't tell me about this, and I suggest you don't care about it. I think Mr. Li Min is really deaf to her. I am deaf in the original words, and I have a very bad impression of her.

40:11

Because I said when we first came here that he was going to be pregnant and then took maternity leave, so it was Mr. Gao Yukuan who accepted us at first. Then what is this teacher called Gao Yukuan? I think he is a very simple and honest, a good man and a good person, and it didn't take long anyway. After giving birth to the child, Teacher Liming came back, and I felt that he was alone. I haven't seen him because he came back just after giving birth. I saw him for the first time. He was really weird. I remember you said it got better or worse. He was ok before the pregnancy vacation, or A very humble, very polite, and very concerned about a teacher, I really have a bad impression of him.

40:59

In addition to the PPT thing, the most important thing is that I told him that during the epidemic, isn't it the kind of person who needs to apply to leave the school? Then I was around April when my ID card expired, and then I kept dragging it on without changing it, and then if the bank has something, if the ID card is not updated, it will freeze your account, you know that kind of freeze, and then I By June, my account was frozen, and I had no choice but to spend money. I told the teacher that I had to go out and change my ID card. I dragged it myself because I didn't want to cause trouble for him. Saying that there is no way, I have to go out, and he will not let me go out of school to apply for an ID card.

41:43

Then I got my ID card after I took the test, and then I changed it during the summer vacation, which caused me a lot of inconvenience. My money was sent to my WeChat by my mother, and then transferred from WeChat to be used. Then he just transferred it to my bank account, and now it can only be transferred to my WeChat. Then at that time, if you were simply not letting you go out to apply for an ID card because of epidemic prevention and control, I think I could understand it. But it wasn't because there were a few boys in our school at the time, and they went out to play under the pretext of asking for ID cards to go home to see a doctor, shampooing their hair, or even going out to dinner.

42:20

She approved all of them, but only girls she often did not approve. Then have you tried to find, for example, Teacher Gao and the others, will Gao Yukuan be too high because of his system. He can only be too high after Li Min has passed. He has no authority.

42:35

Then I was very angry at that time, I thought you were too double standard. Those boys are still relatively unrestrained. It happened that I just saw his WeChat friends in his Moments, and in Moments, everyone was singing K again, and they were handsome hotpot again. I eat in the school cafeteria every day, and even hold a I don't even have an ID card, and I'm very uncomfortable.

42:58

Then anyway, I went to talk to Mr. Li Min later. I said why they can go out, but I can't go out, and he just said that you are still going to hit me, you know? I didn't talk about the main point, and then I wanted to fool me like that, and then it happened to be the exam again, and I was not happy to bother him. I'm still right, I don't dare to be long-winded, it's a waste of time.

43:17

Was there any purpose for you to join a student? Or think about what purpose you want to exercise yourself for?

43:25

Do you remember when you joined the student council?

43:29

I remember when I was just entering the school, I was recruited as a kind of temporary monitor, and then I didn't really want to do these things, because I entered this school and I knew that after a while, my goal was only to guarantee the postgraduate entrance examination. And going abroad, my goals are still relatively clear. Then I thought at first that I would study hard, and I didn't want to have these identities of these social activities. But then Mr. Zhao Gao once asked me for a face-to-face interview, that is to say, he still suggested that I go to some social activities and enrich my resume, and I felt that what he said was very reasonable.

44:11

Then he told me that after the student union came in, it would be helpful to evaluate the merits and scholarships, and that he could also exercise himself. Then he also said it very sincerely, and then I agreed to join it. Well, it may still be helpful for you to guarantee the postgraduate entrance examination.

44:32

So, after entering, I found that I was very disappointed. It was different from what I thought. There was no technical content in doing some things, and I was not very disappointed. After I went in, I was very grateful to the seniors and seniors in my department, and to all my colleagues. It was because of the hardships that our department was quite united. Even if I changed my major and left, Our friendship is rock solid, really.

44:59

For example, if there is something important in the college, they will still tell me about it, and then they will talk to me when they are in a bad mood. The relationship is really very good.

45:07

I think my biggest gain is to gain this group of friends, and then if you ask me how I can help me improve myself, I don’t think I may have achieved my personal improvement as expected, yes.

45:22

Or how do you feel about joining the student union? I think it's a half-open half-open. Well, it's still profitable to gain friends. Yes, you can't blindly deny the gain, because you suffer from The ordeal is right, except for these friends, you may feel that there is nothing to gain.

45:40

is also there. I think my ability to cut videos seems to be a lot better, because I am forced to learn? Then I learned some things, but it doesn't seem like you say how much he has. If you want to talk with the two, I think it's more of a burden on my study and life, including my spirit. I wasn't very good at rejecting it at first. He said anything, Zhao Chen, go and take a photo, record a video, and cut a video.

46:11

At the time, I blindly thought it was a manifestation of my fulfillment, but then I found out that his fulfillment was not the fulfillment I hoped to get. On the surface, you look at me very busy, but in fact I am very empty inside, I feel like I am working part-time, and then you do these things, what I get and what I get, may just pass the time. Which of these things makes you firmly want to change majors is the matter of liming. It's called Dawn, yes, it's not that he is not so powerful yet.

46:48

I also don’t think it should be. I think if I want to change my major, in fact, when I was a freshman, I said I didn’t have such an idea, but it was because I didn’t understand the policy. I thought I might just be rotten. Here, there is no way, then I will be a student of my medical and political college, right? Just be a chicken number one, right?

47:14

When I went to the back, I found out that those people whose grades were not as good as me, even those who were very idle, they moved away because they were science subjects, and they were still showing off their power, which made me very uncomfortable, and then Then how to say, because of their disguised stimulation to me, I said or did what they said to you, or said something to a circle of friends, and then everyone in that group said that I was After turning away, many people congratulated you on planting fish ponds, and then they said something, congratulations, you finally escaped, is it that everyone does not recognize this profession?

48:02

How to say that everyone should have a good idea, and it can't be said that they don't agree.

48:06

So I said what my major is like. From your point of view, the classmates around you in the team feel that they do not recognize the major very well. If there are no restricted levels, you feel that the students around you all want to. I have this feeling when I leave, but some people want to leave. He just has a good vision for the future, that is, I hope to get a good feeling, but I am too lazy to move. verb: move.

48:34

This feeling is that most of the people in the big doctor give me the feeling that I can't say that most of them may want to, but they are not willing to work hard, and some are willing to work hard. , is my roommate. Do you know how much their rankings improved last semester since I changed my major? After I changed majors, one of their original rankings was in the top 20%, and the others were outside the 50%. Now they have become the first, fifth and tenth in a town respectively. Why?

49:05

Because now they have no way to change majors, why did they suddenly start now? I think it may be influenced by me, because I personally think that the learning intensity of jelly is definitely higher than that of medical administration, and then like me As soon as the exam month came, I was basically in the library, and it was difficult for them to see me because I left earlier than them in the morning, and when I came back in the evening, they were all in bed and basically couldn’t see me. to me. Then maybe there is this, they think maybe I work hard, and then they have to study hard, maybe there is such an influence, right?

49:43

If there is one person in a dormitory to take the postgraduate entrance exam, the others will basically take the exam. Then, like last semester, I was a student in physical and biochemistry, then basic chemistry, and then most of my classes, I think I remember correctly, it should be I have breakfast every day, and then I am very tired, and then they think that I sometimes have to get up early after class at 8:00 in the morning is the first class. Then they were more idle last semester, and then maybe they thought it looked like I was working hard, and then they worked hard.

50:20

is hit.

50:21

Possibly me.

50:25

How come there is no such thing as you?

50:28

You thought management might not be very employable, right? I think it's too wide, so I can't say bad.

50:38

My real thoughts are like this. I think it is a panacea-style major, and it needs to be closed everywhere. Have you ever thought that the language major is also a panacea major?

50:45

Yes, because at that time, I chose the language of the major in the college entrance examination or this management was just a consideration for future employment, but after I went to college, I found out even more, in fact, you are a genius. This kind of thing has a very high replacement rate. You can either be at the top and do very well, and you will become a master of management. Then you can only be an ordinary employee. If there is no such thing as self-motivation, you may have been boiling frogs in warm water for a lifetime.

51:24

I think I might not be as good as I expected in One Town, because I actually participated in the One Town career planning competition before, and I got a pretty good term. Then but I compared the occupations of my father and my mother. I think my father's occupation is even more irreplaceable. It has technical content in it. Is it convenient to say my father's occupation? He is an IT engineer. When I was a child, I used to do English speeches, and I would ask your father how it was, and then I just popped out of my mind, Angel Neil.

52:10

Then I asked him a question, and then I asked him a software engineer, and his opinion is that you think your thinking will be influenced by your father's occupation, and you want to do that kind of work that can be said to be high-tech. Yes, I am not only him, but also, um, it may be a consideration for employment.

52:34

Yes, and then when I was in high school, I had acute appendicitis, and then it was very bad, it was very bad at the time, anyway, the doctor rescued me later.

52:48

Then I was full of yearning for my career at the time, so this may be because I knew that I might not be able to find a major in liberal arts, but I still applied for this quality of Nanjing Medical University, yes, I think a lot of things It's all predestined early in the morning, like when I was in junior high school, my biology was very good, just like chemical biology and chemistry, and then I participated in the biology competition and then, but then in high school, I was divided into subjects. There was no way to be assigned to science, and I was very sad at the time.

53:22

But in fact, when I was in junior high school, I often liked to go to that kind of public account, such as biological exploration and so on, and then I was in the final exam, and in the middle school entrance examination, I took biology I didn't take the test in advance, it was also the city of Nanjing at that time and I got a perfect score in the test. My mother gave me a microscope. I was very happy at that time. I said that I must be doing this kind of research, and then my mother said it was great, you can definitely do it in the future, and then he encouraged me, and then when I got to high school, I divided it into subjects, and then I felt that I was with myself. The creatures probably won't matter in this lifetime, and I'm sad.

54:11

But then I said that and transferred to Guozhong again, and then like the last time my instructor recommended us those public accounts in a big public class, all of which I paid attention to in junior high school, and then I had a kind of The feeling of doom in the dark, and then I was very moved. Maybe I was moved by myself. I should be very happy. If I think I am you, I should be very happy and moved. It should be a lot of things. I feel very lucky.

54:39

Yes.

54:41

There is just such a view. If you overlook it, you may not go that way until now. Yes, I feel that many things are destined to feel that way. Maybe it is also It's relative to here. Another thing I want to ask is you and you. You can leave it alone. You may just say something casually. You also say that the school doesn't pay enough attention to him, or that the school's management platform may not be high enough.

55:11 It

is possible to talk about something specific, or to say that there is something that makes you feel a certain way, or that there is a similar emotion or cause you to have this judgment, or you can just say it casually. .

55:25

How do I say I don't think so? I think there are two majors with a low sense of presence in Southern Medical University, one is medical administration, and the other is English. Then why do I feel that this school does not pay enough attention to him? Because how to say? Look at some of the pushes he sent. I think the pushes are like I'm moving to the workplace now. There are a lot of things on our side that focus on student training. For example, we have a big innovation. I said no It is said that the workstation is the national weight, because in fact, I think there is still a little difference between the national weight and the public health.

56:11

Like my current middle school, you see, I think the school attaches great importance to it. First of all, our curriculum is different from other professional prevention majors or basic majors. Our curriculum arrangements are different. We will There are many of their own courses, such as reproductive biology, then gold and stone, and then the cutting-edge development of life sciences. These are the characteristics that schools want to cultivate you, so they will put energy into getting teachers to match. There is such a course.

56:40

But what you said about the management side, in terms of their curriculum arrangement, I think it makes me feel more like I am looking for a class without a class.

56:50

You, for example, my roommate, she is currently in school and outside of school. Women and children are suffering from epidemics. It is a torture for them, and it is also torture for the teacher. The feedback they gave me was that their teachers didn't know how to teach this kind of class to management students. It was an earthquake in the school. Three, four or even five, the courses I took separately were.

57:20

One at most 2 courses in one semester at a time, 5 of them are not. They should also go up, which means that the requirements will not be as high as he gave them, but you still have to understand. I feel sorry for my roommate, she doesn't like this at all. But she still has to keep looking at these things, and the teacher doesn't want to talk too much about them.

57:47 The

teacher is also annoying, so I think you are studying management and medical administration, right? In the future, I don’t want to do this kind of understanding as a clinician. It would be good to arrange so many courses that I don’t know what to say.

57:59

Then you see that they are about to take the exam, and they don't know what materials to review for the exam, because there are so many clinical materials like mine, all of which are question banks, and there are not so many big questions to memorize for thousands of questions. There are so many things to memorize, they feel that there is no need to learn so much, and it is very painful for him to not know how to learn, and then I think that you can actually classify all of them into one subject, such as what? The clinical foundation is like this, and then let them learn, I think it is much more effective than splitting it into so many 5 courses.

58:32

right?

58:33

Then you can see that they have no other courses this semester except these courses, and these courses are all for them to take this semester. If the other -2, the sexually transmitted diseases are gone. This is very intense. I know that this class is difficult for them this semester, but there is no need to give him so many class hours, but they only have these few courses this semester, and there are so many courses, I can show you his class schedule, What do you want to see in the timetable? We only have epidemics, and we don't have women or children.

58:58

Do you want to see the class schedule?

59:00

is very difficult, I am really very scary, that is, they all go, they all start at 8:00 in the morning, and sometimes because I have a few exempted courses this semester, then I am relatively relaxed. Sometimes, I go back to the dormitory earlier, and they are very hard before they come back, and like my roommates, they have been counted as good grades, show it, every time I tell me about these When taking a class, the emotions expressed are all very negative emotions. They feel that there is no need to learn, and they don’t know how to learn. The teachers are not willing to teach, and they feel that the course arrangement is very unreasonable.

59:35

Then from the curriculum arrangement, I don't think it is very scientific, and it also shows that the school may not pay so much attention. Then in my freshman year, those classes I took on my own made me feel very watery.

59:53 Does

the water mean that the exam is too easy, or is there no substance?

59:57 The

teacher is also reading PPT in class, and it’s over after reading. In fact, you should be more straightforward. I listened carefully in this class or I didn’t listen in this class. In the end, my exam results may be the same, this feeling. Like the general water course, its final exam is not called a water course, it's microeconomics and management. I was really good in freshman, and then they didn't learn much, but I'm pretty good at these subjects. .

01:00:29

This is above 85 points.

01:00:30 When

I recite it, it will be high. I didn't recite it. I think it's just my high school.

01:00:38

Health economics, your high school boss didn't take their third year's class on health economics. He studied management, and management is just three words. Management is the orange kind. I think it is very simple, orange. That's called hospital management. Maybe the courses are different, my name is management. I really think it's very simple. The principle of management seems to be that no one manages the system.

01:01:05

Then his teaching arrangements are constantly changing.

01:01:10

Yes, it may be different from what you took at that time. Then when I took it at that time, I was very interested at first, because I remember the teacher who taught us the first time called right.

01:01:22

To Lu Fang or Teacher Fang or Teacher Lu Fang, then I thought it was very interesting that he came up because he told the story of three monks carrying water to eat, and then I thought this class was quite interesting, and I was still serious I went to attack your interest. Later, it was not Mr. Lu Fang who went to another teacher. Then he was in charge of a class, and for Mr. Tang Weiwei. When he came into class, he told everyone to chat, and then he said that everyone came here. When you ask me any question, I feel like I'm on a variety show.

01:01:58

Happy but not unhappy, I thought you were in that class anyway, and then I felt that you were not as good as that teacher Zhang. Yes, then I'm not saying that he is not good, he painted the key points later, and the paintings were very accurate, right? Then the lectures are okay, but I like Mr. Lu Fang's teaching style. Then he gave us a debate session. I remember that I was still the best debater, and uh, I liked this class very much. It's very interesting, and then when he was going to take the test, he didn't have any information, and everyone didn't have any information, and then the seniors and seniors also said that there was not much of that kind of content, so I just looked at the PPT and so on, and I went through it again. , anyway, just go straight to the test. I think what I write is in the vernacular, but in fact, if the teacher doesn't talk about any special knowledge points in class, it means that he will still see the answer directly and show it to you. Your understanding level may be ?

01:02:57

In addition to the curriculum arrangement, I said that the teacher gave me the feeling that the school did not have good resources, probably mainly because I felt that compared with the basic work station and the clinical side, the strength of the teachers appeared. relatively weak.

01:03:16

Then simplicity comes from your definition of the word weak, maybe it comes from, for example, the teacher just read the PPT, there are many teachers who read the PPT, yes, and then like me in class now, few teachers can make me I feel that I can't listen to this class, but I feel that I can't listen to this class in a town, so I might as well watch it myself.

01:03:35

After listening to it, I felt that the effect of this teacher and my self-study were similar. Yes, I will give an example. For example, I am currently studying pathology. Then my teacher is called Ma Juan, I don’t remember much. The pathology teacher I think he speaks very well.

01:03:53

He is very organized. First, for example, what kind of lesion is your lesion? Then, its characteristics under the microscope, the macroscopic characteristics of its large eyes, and its main characteristics? His organization is very clear, I can easily get where the key points he said, I know how I should learn how to memorize, and then how to brush the questions.

01:04:16

But there may be differences between disciplines. For example, when I was taking microeconomics, I thought it was a very science course, but when the teacher spoke it, I felt like I was listening. History class, microeconomics is what you take in your freshman year, for your freshman year. I have a feeling that taking a history class is really the history where macroeconomics should rise. Macroeconomics was a sophomore in my sophomore year. At that time, I changed my major. Microeconomics should not be like this.

01:04:47

In microcosm, I think a lot of microcosm still needs to be calculated, not depending on the line, but what he said was that he was just reading PPT, and moreover, Mr. Chen Mingsheng was the dean, he said what he said In fact, it's still very good. After a new teacher came later, I couldn't listen to it anymore. I felt that listening to him in class was no different from self-study.

01:05:13

Yes, and I don't know where his focus is, so he gave me the feeling that the whole book is the focus, but the whole book doesn't seem to be the focus. What do you have for me to the next question. No.

01:05:35

Question, I will ask the next question. I actually talk a lot, but you still haven't answered what is it that you are determined to change your major, right? In fact, I think that everyone turned away at that time, which had a great impact on me. I felt that I didn’t grasp it because of the restrictions on liberal arts, and I was afraid to find out that there were policies and this opportunity. In fact, I thought he was very interesting, but he didn’t. Infertility is now a very hot issue, maybe because I can say this now, maybe also because I am studying in the field of promotion, and I have a lot of contact with him, and then I am now Discovery is really a very serious problem not only in medicine, but also in sociology.

01:06:24

You can see that China's economy is also under pressure, and the 7th population census data has come out, you see the population has decreased again, right? And that means aging is getting worse. But now many people rule out those social factors. For example, DINK does not want to give birth, and some people want to be born. Maybe this is related to environmental pressure and other factors. I think I am going to learn this appreciation now. is very meaningful.

01:06:57

It is not only for myself, I think he may be very good for my future employment, and it is meaningful for the future development of human beings.

01:07:10

I remember that I communicated with my senior brother in that laboratory before, saying that your senior brother, you are doing a Ph.D. here, do you feel happy? He said that he answered me without hesitation, saying that happiness is of course happy, and I especially like to do experiments. They find it very meaningful every day, because maybe doing this experiment today will promote the development of society and even medicine tomorrow . As long as he is full of passion, I am influenced by him. I think it is indeed a very meaningful thing, and then you go back to what you asked me just now, why I am determined to change that, in fact, to put it bluntly, in that environment I am I didn't like it, and then they were the ones who were better than me. They also turned away. I was unwilling. I thought I would also like to try it. If I came to a conclusion, the environment of the college that I came in was not very fond of student work. , um, um, there are some disappointments in the curriculum, reaching one is considered to have reached a peak, and then I found that everyone changed majors at this time, I was even more uncomfortable, and then I found out that there are policies.

01:08:14

Yes, and actually my roommate at that time is now the first classmate of the first grade. He and I are very good friends and roommates.

01:08:24 After

I saw the news at the time, I immediately forwarded it to him. I asked whether we should try it together, because both of us had good grades in one town, and both of us were Because it was limited by the top 20% left over from the liberal arts, I then told him whether the top 20% of your science subjects were similar, and all the students who should be transferred were transferred, basically all of them. We recruited 120 There are many people, and now there are only nine to 10 doctors, and then I asked whether we should try it together, and then he wondered if he liked medicine very much. He felt that it was very boring to learn, and then he was not very interested. People, and then said that the time is relatively rushed, and it may be a waste of time to prepare, but there is no gain, or if you want to transfer, I will transfer by myself, if I say yes, I will transfer by myself.

01:09:15 It's

probably good. We have almost understood the previous process. Now think about it and ask if you are starting from the middle school class, whether there is anything that makes you happier, or something that makes you more depressed? happy things. It’s okay to be happy or depressed. The things that impress you deeply, I think the things that impress you the most are completely different environments. You feel that the environment is different like I am now. The biggest impact on you is the student or the teacher, or the whole class atmosphere or the course setting. Which one do you think is for you to take me quickly? .

01:09:59 The

most important thing is that I think the teacher attaches great importance to students, which makes me feel that the change is very obvious.

01:10:09 After

I arrived in the middle school, you are like we are going to rotate into the laboratory, choose a tutor, and then there are various laboratory courses. I think this makes me feel that I am in this school. Some of the time is fully utilized, I feel that I am learning something serious, and I have that kind of satisfaction.

01:10:33

Then, especially every time like the end of the exam, then the results are released, and then the rotation is carried out. The rotation of the semester is over, and then the report is made. When I report, I feel that I have not worked hard this semester, and I feel that I still have it. After harvesting, I feel that my days are very fulfilling.

01:10:51

There are things to do every day, and it is impossible that sometimes it will not be like in the previous college. I feel that you have nothing to do today. I now feel that the next step is to relax, and then The rest of the time is very tight, and then I feel very fulfilled and rich in time. For me, I make good use of it, and then I am very happy.

01:11:19

Is there anything that makes you feel relatively unhappy? It can be your own person or other things that are not related to the Guoyou class, right? Yes, in fact, I don't think it can be counted as two semesters since I entered China, and it will be almost two semesters.

01:11:37

In fact, my mood has changed to a certain extent. Since I first entered the country, I was excited and looking forward to it. I was thinking about what kind of people I can meet here, and what kind of people I will meet. , what the teacher has learned and what kind of person I will become, I am full of curiosity, and then I feel that it is constantly moving with time. I think it is only the same when looking at the middle school class now. The biggest and the most why I suddenly have this thought now?

01:12:06

Because some bad things happened before, in the middle school class. I don't know if the previous students who were interviewed have told you, because I don't care to talk about it easily. I think he may have already talked about it. I know that Dai Anqing also talked about the interview, because I have a good relationship with him, and then How to say we have a special course, it is called the ten major advances in life sciences.

01:12:34

Then the teacher in this class may have said something related to politics or a more sensitive topic in the course of the class. I will directly say that the teacher said something about the cotton incident in Xinjiang. , it has different positions, I don't quite remember exactly what it said, but probably the meaning may be related to national propaganda.

01:13:04

No, in fact, I think what he said is very reasonable. It's not that what he actually means is not that China is not good, what he actually means is that our country is strong, but our foreign policy, including the quality of our citizens, should keep pace with the times and become stronger together, not He thinks that the spokesperson of the Ministry of Foreign Affairs is called Zhao Lijian. Well, when Zhao Lijian was responding to the hm incident, he had the feeling of a peasant woman's small pattern. It turns out that those China who were bullied by you, and then we are stronger now, why and then we have the ability to sing the opposite of you, you know?

01:13:54 And

then like Lao because he was anxious about what he said today, I must not remember it now, and then I think the teacher said that China is now strong, but our diplomacy is still not enough. Atmosphere, I actually think what he said is quite right, because many netizens, including some mainstream media, the People's Daily, Xinhua News Agency, etc., when expressing their recognition and recognition of China's development, will inevitably bring some historical facts. For example, China used to be very weak and was bullied by foreign powers. Maybe everyone sees it this way now. In fact, it's nothing, and it's understandable, but if you talk about it every time you conduct diplomacy I found out that I used to be a victim of weakness, and then jumped out and said that you used to bully me, but now I emphasize this kind of feeling, I feel a little inferior, I feel inferior, just not enough atmosphere, I I feel that since we have become stronger, we must learn to be tolerant. We used to be like that in the past, but there is no need to keep saying this in the diplomatic arena. Then the United States said that the Xinjiang cotton incident was mainly because they said that we Han people slaved to some Uyghur people like anti-China elements, and they themselves, including some people in their families, went to pick cotton and so on. Then someone said on the Internet, for example, in the United States, you enslaved black people ten or hundreds of years ago. In fact, his reaction seems to be that you have made mistakes in the past, and you can't accuse me of being wrong now.

01:15:40

I don't think this kind of thinking is right. You are wrong, you are wrong. No matter who you are, you can point out that you are wrong. No matter what he used to be, at most he is right, right? This kind of confidence is not enough, but I didn't realize this problem before, you are very right.

01:15:53

I think this is what the teacher said to you, or is it your own way of thinking that is my interpretation of the teacher's words, I am like this, so the teacher is also conveying this meaning, to me I think he conveys This is what it means, but in fact, some students may be in the course of the ten major advances in life science after all, we can't spend a lot of time talking about such political things, his teacher may have brought me this kind of remarks, He said that maybe our diplomacy was not strong enough, and then he talked about cotton, and then about black slaves.

01:16:20

Actually, as soon as I heard it, I felt that because I was thinking the same way, maybe I thought what the teacher said was very reasonable. I think his thinking is more correct, right?

01:16:31

The possibility of cutting-edge, but in fact it runs counter to the ideas of some mainstream media propaganda. It is actually criticizing some mainstream media. The way of its propaganda may be that some students think that you say China is not good, your ass is crooked, you are a traitor, you have been in the United States for too long, and then it is our own. In the class group, is this reaction the reaction of the classmates in the middle school class around you? How did I know that, in the middle school class, they watched anonymously and scolded the teacher very badly. I thought at the time, God, do the students in the middle school class have this quality?

01:17:08

At that time, I had a very uncomfortable feeling that I couldn't describe. I felt that I worked hard to prepare for the national high school entrance examination. I hoped to meet a group of such outstanding talents, but it turned out to be the same. There is a group of people who have the mentality of a petty citizen. I think of Guangxi people and I feel very uncomfortable, and then I feel very sad. I will tell my other classmates why there is such a quality in the middle school. Poor, man?

01:17:37

Do you want to see the chat log and tell me what it is about? This is pure swearing, right? Just scolded it very hard, I think I can take a look, you are more curious, right? I can wait a while and find it for you. They are the kind of flamboyant people who feel that I am a good citizen of the People's Republic of China and that I want to overthrow your major case. The key is whether one or two people are like this, and many people are like this.

01:18:08

Then someone said, let's see who will go to the teacher's laboratory in the future. I don't think it's necessary, and I think it's normal for everyone to have different views on these things in politics, not just politics, but also various things in life and society.

01:18:27

I think you, as a middle school student, need to look at the world with an inclusive mentality, right? You said that if your thoughts are limited by the mainstream media or some things produced by your current mainstream science, without bold innovations and breakthroughs, if you have your own ideas, I think what you cultivate are basically gorgeous machines. You don't have your own ideas, do you?

At 01:18:50

, I found a gorgeous machine. I really like your words. Then I think your country is not only about cultivating technical talents, but more importantly, cultivating talents with such pioneering and innovative ideas. You say middle school, you say we go to the laboratory to do experiments, you actually practice these experiments for 10 days and a half month, who wouldn't believe that all the students from Yizheng come to Zhenger Bajing for training, and they must have a job certificate. can be tested.

01:19:18

But I think what you learn in the lab is really an experiment? I think the kind of thinking that I think should be learned from the tutor, and then those experimental ideas of his, and then, if you look at this trivial matter, I feel that their thinking is very imprisoned, and they are not willing to accept new ideas. Some particularly striking ideas.

01:19:39

Then when you are doing experiments in the future, for example, you may say that I am a little outrageous, saying that He Jianhui manages and edits the human being. Human embryo babies are very controversial in academia.

01:19:56

But the fact that there is a controversy means that it is something that deserves attention. Otherwise, if you don’t sell green vegetables from Li’s family for three yuan in today’s market, then Chen’s family sells green vegetables for five yuan. This kind of trivial thing can be called Controversial?

01:20:11

The difference in price and price can't be called controversy. Controversial things show that it is worth discussing, just like the case of He Jiankui, his elite editor HIV infected this kind of fetus, he It's really not very ethical, but you said that if he succeeds, it is indeed a big breakthrough, maybe I think it's a bit dangerous to say that, but I don't think I'll do this This kind of violation of ethics and morality, some people have nothing to do with our interviews, but maybe his success may inspire some people, for example, let more Tei deliberately act HIV, yes, I think some Sometimes what you discuss is right, and sometimes what you discuss is wrong. You might say, for example, whether He Jiankui is good or bad. Now everyone says he is bad, but if you are bad and hit the wrong target, that's not good either.

01:21:11

For example, some people think that his bad ethics are bad. I think this is because ethics are not very good. But if you say that his bad is bad, but you say that his bad is your pioneering innovation, I think The target was wrong.

01:21:24

I think like this, so as you are a student of these scientific researches and students of this kind of fruit seed class that are really cultivated by the school, I think thinking is very important. It should be more inclusive, and then more pioneering and innovative. Whether it is the original idea or some new and more radical ideas, I think it should be treated with an inclusive attitude. I am not saying that you have to accept it, at least You can't just because I think of Chen Shanni before, when she said that some people just think about one thing when they are discussing, what he said is that you are equal to me when they communicate, which is equal to right and equal to justice. I think this is wrong.

01:22:08

You just say that you, for example, his teacher talked about these topics about politics in class, you don't agree, right? You can disagree and reserve your opinion, but there is no need to kill it. I think these are two different attitudes. Have you thought about where it might come from? What source? It is their thinking mode that may not say much, just say that the thinking mode of the classmates around you may be black and white, maybe you think that China is not good, you are a traitor, this may be contradictory. Contradictions of views are common, and they may not have had a deep exposure to such philosophical ideas.

01:22:50

Where do you think this might come from? I think a large part of it comes from family and the environment to which it belongs.

01:22:58

You have thought about this, yes, because I think a family is very important in shaping your soul. I feel like my parents, they pay great attention to my inner cultivation, maybe this is what I and my other classmates said. They are very good at exams, and I often fail them in exams.

01:23:21

I really admire his classmates in our country. He is watching Times Youth League in class, and then after class he starts watching dramas. Then he asked me to borrow notes a month before the exam, and I lent him the notes. After that, he took 88, I took 80, and then I felt very uncomfortable, and then but you said, for example, I want to communicate with him about some ideological things, he didn't bother to care about you, he will take care of you. Did you find this kind of confidant in the class?

01:23:54

is a classmate who can deeply exchange ideas, literature and history with you.

01:23:58

Do you or your roommate have any? Do you discuss this with your roommate?

01:24:04

No, I'm right, I want to discuss this with them, but I don't think it's possible to discuss it. In fact, people say that life is hard to come by, and confidants are indeed like this, I think I feel , I think my best friends now may actually be my classmates in junior high school. They can all discuss this kind of thing with them.

01:24:26

I will tell them, and then they will also express their thoughts, including one of my high school classmates, who is also in Nanjing Medical University, and although he is an English major, he is actually a very thoughtful person. I think even though he doesn't like to study, he likes to read philosophy. I think he should study philosophy. He is very romantic and has a lot of personality. I want to study anthropology or something, and then I feel that I will be very happy to communicate with him like this. After the teacher's matter comes out, I directly ask me to talk to him, because they are all in Nanjing University, and then The two of us went to press the playground, and I told him, I said it was outrageous, I really did not expect such a thing to happen.

01:25:12

Then he told me that in fact, there are people like me everywhere, and there are very few people like us. I said that's okay, that's right, I think it's true that I listen to you and I get goosebumps again. , I think you speak very well. Why? Because in fact, I have also thought about this issue, and it has nothing to do with our interview. In fact, I am doing interviews there, and I think I have benefited a lot, really?

01:25:37 When

I communicate with others, I actually find that many people think differently from me, and when I communicate with them, you may tell him a lot, but sometimes he may He also realizes that there may be something wrong with him, but he still won't accept your thinking. There are still many cases in this case, so in fact, if you think about this situation, I think my personal point of view, I think it may be their humanities. Thinking may not be enough. For example, like the most basic teaching concepts, many people's opinions, including the current film and television works, his shaping of a person is that he is either a pure good person or a 100% bad person, he does not This kind of good and bad is mixed, because everyone has their own advantages and everyone has their own shortcomings. When he makes a decision, it must be the best decision for himself.

01:26:26

You can't say that he is bad or good when he makes decisions, so in fact, I think my personal feelings, including my communication with the people around me, including my online communication with netizens, I feel great. In fact, some people have no way of saying whether the classmate you mentioned is a good person or a little bit more bad. They think that you are 100% bad for the bad things you do.

01:26:49

100% of what he does a good person is a good person, so they don't have this contradictory concept, they can't accept a person who does a good deed, they can't accept a murderer and jump into a river, There is such a thing as a little girl, which may include, for example, seeing that kind of murderer on the Internet, it is hard to say how to say it, but maybe he has a very reasonable reason, but many netizens are just me reading those comments on the Internet. When I feel that they are not very accepting, this person has a reasonable reason for doing a bad thing, and they will think that there must be other bad reasons for him to do this thing, because he is a Bad people, so many people can't quite accept it.

01:27:32

A person may be mixed with this kind of concept. In fact, he may not have started to contradict philosophical concepts. In fact, I think you should really do these humanistic education, and give these Science students do this, I think, you can have this idea because you are a liberal arts student, and you may be very good in historical and political science yourself, and you have an ideological relationship.

01:27:57

These have nothing to do with the arts and sciences. I think it has a lot to do with my family education. Like my parents said that my father is a science student and my mother is a liberal arts student. I think they are very tolerant intellectually , and they are very active in guiding me.

01:28:18

I think the reason why I can now have my own free-thinking soul is mainly because my parents liked to let me express some of my own ideas when I was young, for example, some things may As a parent, you don’t need to consult your child’s opinion, but my parents will ask me about everything, as long as it is related to the family. For example, my parents are going to buy another house recently, and then put the The old house in the family is sold, and then he is like this kind of thing. I think it is completely their business and there is no need to ask me. They will all come to ask my opinion.

01:28:59

Then over time, I have developed a habit of having my own thoughts. I will think about it. When I see this thing, I will think about these things, including my feeling that my mother is right. My education is like a kind of dining table. The education of conversation is that every time I have dinner at home, he will often discuss with me some social events such as some hot searches on Weibo. Forty-ninth High School, he would then discuss with me whether the child had a psychological problem or something, or if you see at first everyone said that the school had a problem, and then it turned into the child committing suicide .

01:29:38

Then you see this public opinion, and you are laughed at by that person at first. Everyone thinks that there is a problem with the school, and the public opinion has been led astray, and everyone is scolding the school or something.

01:29:46

Actually, I think that after 19 years of this kind of ideological influence, when I saw this, my thought was that I felt that I had that kind of critical thinking in it. , I will think that is the school really so bad? Did I say that something needs to be done to kill this child? I will? Put a question mark first, just because I read the comments, I won’t say how about this hammer death, including my roommate and me talking about melons and so on, all about dying, are you really hammered to death? Are you a party? Some things are not really what we see.

01:30:21

You should have a dialectical feeling. You should be a good friend, and that is what you think is a good friend in philosophy. His family environment is also like this. Have you heard of it? In terms of her family environment, she and I know which big teacher her father is, and then he is also in this building. I only saw him today, and then whether his mother teaches English, high school or high school, I don't know. I remember being a teacher too, and then I think the reason why he has this kind of thinking is that he reads many times more books than I do.

01:30:55

I think I should already be a person who reads books a lot, because I like reading very much, which may benefit from my parents, because they both like reading very much, so I like reading very much since I was a child.

01:31:06

Then I especially like to read books when I get to the exam, and then like my classmate, I call him Pippi because he is just his nickname, and then Pippi likes to read books, but he doesn't. The books I read are different from the books I read. I think the books I read are more orthodox, but the books he reads are very rebellious.

01:31:31

The ones he read were like books written by some minority writers in Spain and Portugal, with names that I have never heard of.

01:31:38

Then, like me, it may be more popular. Well, it may be seen more, but the area involved may not be as wide as his. I think his ideological realm is higher than mine, and he is really suitable for grabbing money. But I, I, I feel that if he studies philosophy, he will not be happy. If he is still happy, he will not be happy and happy, because I am afraid that he will commit suicide. He thinks too much, I think.

01:32:12

Why do you say? There are those people who feel that the world is either black or white, because the middle part is simple because it's black and white, and the gray part in the middle is complicated and painful. If you have been in the complicated section, if you have been in the complicated section If you look at the world in the gray area, you will feel that you can’t see through everything. Maybe I’m not at my level, so you can’t see through everything, and then you feel like it’s all painful. For example, if I’m a little more pessimistic, I thought I would be fine if I transfer to a high school?

01:32:44 If

I find a middle school, my future will definitely be better than that of a doctor? What is your definition of success? I just talked to my classmate from another middle school a few days ago about this issue. He thinks very simply, so he is happier. Like I think a little more sometimes, I will be very miserable. You What is your personal definition of success? I think I think that my definition of success is now, I can realize my own value, maybe I have a certain contribution to a certain field or even some people in a certain place, I think I am quite successful.

01:33:18

But I think it's right to ask me why before going back to you. right. There is such a change of mood in the junior high school class. In fact, there is another very important reason, that is, in addition to the fact that they scolded teachers anonymously, which made me think that they are a group of people with that kind of closed-mindedness, I also think that everyone has ideas about success and values. Actually they are different people.

01:33:48

I think most of my classmates attach great importance to examinations. If you say that I attach importance to examinations, I do attach importance to examinations, but they are different, I think.

01:33:58 It

seems that I use the grade test as a means of self-realization . I will not be very happy because I did well in this test, not because I did very well in this test. No, I am very uncomfortable, but some of my classmates think that their success depends on whether they get a scholarship, whether they win an award, and whether their grades have become better. . I think it may be easier for them to struggle like this, and it is easier to be happy, but if it is me, it is good for me to break 4, but I think it is not just that simple, it is me and I think so.

01:34:36

I have a small question. I'm curious. If you say that your classmates want to compete for scholarships, do you think they care more about the honor of the scholarship, or the money of the scholarship, or both? Have?

01:34:49

I think because the Chinese version also has scholarships, but I guess this thing may not be so attractive to you. I want to know that this is okay, so how to say it, I think the classmates in Jin Guo Middle School know about them, because we can't evaluate additional scholarships now, they are all in the freshman year, and then you Sophomores can't be evaluated again, sophomores can't be evaluated again, but they are very accurate in many things. For example, we sent out an experimental operation skills test today. Of course, I thought that I haven't washed it yet, and then they This kind of small exam always thinks about getting 100 points or something. I don't think it's necessary sometimes. This may be because the three views are different.

01:35:33 Whether

they value the scholarship more, or the money, I think it should be both, depending on their family, you can no longer judge other scholarships, why do they It must be so high in the test, yes, this is where I doubt it.

01:35:48

If you ask me this question, why did I go to high school in China and get a scholarship? Basically, it’s the same with your postgraduate postgraduate program, because our postgraduate postgraduate live broadcast conditions are relatively loose. Yes, and then I think that normal middle school students can achieve it, why do they still need to do this?

01:36:09

I think if it were me, I would have really started to take the exam this semester, I could feel myself taking the exam, and I was forced to start choosing because everyone was taking the exam.

01:36:18 The

first is the environment. In your class, everyone's grades are generally around 3:00, and you are like me and us. The average grade in the class last semester was around 3:7, but you are all very good, but You have only one classmate who is truly top-notch, which means that the current situation of our class is that there are few top-notch students and many excellent students. However, in this case, all excellent people want to become top-notch people, and everyone will start. Just rolled up.

01:36:51

There is no way for everyone to roll up. If I lie down, I will most likely not be able to protect even 40%, so it will actually cause you some trouble.

01:37:01

This environment is sometimes there, but if you say that he wants to trouble me very much, it doesn't seem that the most stressful thing for you is to study in a junior high school.

01:37:13

No, I think the most stressful thing in my junior high school class is that I can't balance the relationship between scientific research and study. Because, like our middle school, it is not only about learning, but also about scientific research tasks in the laboratory. You said that in fact, I think we just came in as an undergraduate, and we actually don’t know much about scientific research. We are blind, and it is very abstract. We don’t know and just read literature. You said that the teacher said that you should read literature. You seem to understand, but in fact you don't understand at all.

01:37:44

I think this thing should be fine and precise, that is, after you determine the direction and then study in that direction, it may be better.

01:37:52

Then you have the requirement of this kind of experiment like we now, and we also have a hard requirement for the number of times, that is, you go at least three times a week. But you seem to be too late for the endorsement when you take the exam. If you want to go again now, I think the pressure will be greater, which may be more troublesome to me.

01:38:11

Then, for example, those classmates compare papers, or some classmates may not have very high ideological quality. I think these are at most a little influence on me, because it is impossible to cause trouble, I think people are still independent individuals, how are they? In fact, I think it has nothing to do with me, I just need to be myself.

01:38:33

In fact, the main problem is your own balance between research and study. Do you have the following question? Let me first ask you to rotate. If you rotate, does it have anything to do with you determining the direction from behind?

01:39:05

is relevant. Was the mentor you chose now one of the ones you fell for at the time? I haven't picked a mentor yet, we won't pick a mentor until our junior year. But you said that the rotation is now to choose a mentor? That's definitely true, because the process of rotation is the process of exploring your own interests and then choosing a laboratory. For example, there are three directions on our side, that is, spermatogenesis, ovogenesis, and then the birth cohort. Except for the birth cohort, I have rotated the other two in detail, but in fact, I also considered the rotation of the target, but I did not write at that time. Seagulls do the Thermo Fisher thing, you know?

01:39:42

I know, because this incident was a more serious incident that we blocked the news later, that is, we found out that we actually had the new coronavirus, and then we all went to the nucleic acid. That's right, that's what happened, so our rotation study was cancelled during that winter vacation.

01:40:01

So in my round, I actually signed up for the laboratory of the provincial school that was born in the cohort, and then I had to study, and then I blew it, because you were not allowed to stay in the school. What do you mean by not letting you? It's because he doesn't let you continue to study in school because of three things, that is, your undergraduate student just went back for winter vacation, and then he didn't make up for it later, so there are vacancies in birth and employment, such as sperm and eggs. rotated.

01:40:35

So the next little question to ask is that you are a sophomore now, right? When you were a sophomore, did any juniors come to ask you about the activity class, and today and today, a junior came to ask me. They asked about the ones that impressed you the most or asked the most. Question, what is it? Basically, it's basically because you have successfully transferred to a junior high school. I also want to know if there are any good suggestions for transferring to a foreign country. How to answer this question?

01:41:07

It is difficult to answer, so I have given them a formula for my answer now.

01:41:12

No, I will first ask them what your professional ranking and GPA are, and if you are qualified, yes, first ask them if they have met the hard standards, and then if they do, I will tell you the next one Take the exam first, come back to me after the exam, and then just push it away, and if you don't reach it, I'll say it's okay and continue to work hard. After the exam this semester, come back to me and tell me you got it, wait for them When it arrives, what advice would you give them?

01:41:43

Look what they ask me. If I think the shareholder class is more suitable for what kind of underwater science, I think it is more suitable, I think it must be really interested, right? Otherwise, you are not interested, you are for me opportunity.

01:42:10

Actually, it was because I was still a little interested in it, so I felt that after I turned around, I would try my best to persevere and learn something like this in this place. Then I think you said you asked what advice I would give them?

01:42:26

First, I will ask them if they are interested, do they really want to switch? And then I think if they really think about turning, I think you should have that kind of aura of fighting back, yes, when I took the test, I didn't think about it at all, and I still remember I desperately watched.

01:42:47

If you think about two books that thick, it took me another 4 days. When I think about it now, they all have some kind of heroic color, and then how to say, I really didn't think about it at the time. There are a lot of them. I talk to every younger brother and younger sister who wants to consult me about changing majors. What I talk about is your purpose, and it must be simple.

01:43:06

If you want to make a move, if you want to make a move, then if you work hard, just do it, and you will fight back, right? Don't think about what I can do if I can't change, what I can do or not think about seeking, and start seeking my comfort now.

01:43:18

I think they are looking for comfort for you, some of them are, I think there are some medical and political school girls, because most of the consultation I am still a medical school girl, give me a kind of them. I don't like medical administration, I want to go to a better platform, and then I feel like from Guozhong because they think my exam is easy, because I don't know why, maybe it's what my former classmates advertised to me, they think I took the exam very easily, in fact, I took the exam very painfully.

01:43:50

Then they felt that they had to ask me, Guozhong felt that you had an easy test, did you ever knock on the door or something?

01:43:58

In fact, they may want to have a trick or something, yes, and then they want to know how to take the national important exam, what the exam will take, which subjects to take, and then, for example, what questions will be asked in the interview, and then there are There are no additional tests, maybe just ask. Anyway, now I still consult the whole process, yes.

01:44:20

Then the main thing is how to say it. What I want to ask me is whether there is hope to change that feeling, think about it and seek a kind of spiritual comfort?

01:44:33

Anyway, I basically talk about it every time. After you study hard this semester, there is hope. Then come to me after the exam.

01:44:41

Well, but you said that I have to talk to them so much now, and portray the future very well, which is actually very cruel to them. Don't you both waste each other's time? Right? Do you think that after a year of studying in the middle school class, did you meet your expectations? Have. Yes, because I came in at No. 2. I think I actually want to meet a better version of myself in the class.

01:45:12

I think you said that I went from 2nd to 14th, but it was actually because of my last semester, and in my last semester, I didn't feel that I just treated every subject very ordinary, and then I could take the test. By the 14th place, I think I am very satisfied with the result. Then you can say that you can also let you do research, which is definitely possible. I think it should be possible, I haven't figured it out, because it should be ok. In fact, you want to talk about Baoyan in the season. I am actually focusing on the national policy, which is also what I am not satisfied with. He is very opaque and opaque. He said that you reached the top 40%, which is your current season. 40% of the algorithms, there is a very interesting thing.

01:45:56

At the beginning of this semester, my counselor sent me a message. When the semester started in March, March, April, my counselor sent me a message saying that your discipline inspection is not good. One by one I was stunned, I said why? I said that there are many professional courses here in our country, and I have a score of 95-97. How could you possibly be the one who said that I was the top one? How could it be that I was the top one? Then he sent me a form, and what he said seemed to be moral, because my surname was Zhao, and then the last one actually he had a lower reference than me was still in front of me, then he might not have seen it, and then I was very I was puzzled, but his GPA was different from what I calculated by myself and my degree.

01:46:38

At the time, I thought you didn't have a unified standard, so I compared how to draw the 40-40% line. I called the section chief of the Academic Affairs Office, and I asked him how you drew the line, and then He told me that there are three different ways to draw this line. I thought it was very strange at the time. I asked you to tell me which drawing methods there are. He said that the first one is the node of your graduation. Calculate the grade point of the main course, and then the other is the grade point of your postgraduate study, that is, the grade point of your main course multiplied by 70% plus the grade point of non-core courses multiplied by 30%.

01:47:21

And the third one is that your country does not have that kind of algorithm, and the machine is the kind of algorithm that comes out of the program. So your algorithm has not come out yet. He just promised it verbally, but in fact, I couldn't find it if I went to the official website to check it.

01:47:38

So I feel very comfortable with you. You just mentioned 70% for main subjects and 30% for non-main subjects. This is for postgraduate students outside the middle school class. Yes, because you draw 40% like us. How do you draw? You draw with students from prevention majors or basic majors. You have different courses like ours. Your line drawing is actually unfair, right? So we actually think that every middle school student wants to check the grade point or compete for the 40% line, because we have held many meetings about the 40% line.

01:48:11

Then is this what the teacher volunteered for you? It's what we asked, because our 17th, 18th, and 19th students have asked this question, because they change every year, and last time because this line has been changing, so 17 and 18 have seniors and sisters one after another. After quitting, I feel that the policy of guaranteeing research is too unclear. Not only is it unclear, but there is a feeling of drawing a big cake for you.

01:48:34 It

's because when I was in grade 17, I learned that their policy for postgraduate research is not to look at your academic grades, but only for your published articles in SCI.

01:48:44

For example, I have a very good senior friend of grade 17. He is very powerful and he will post articles, but his new policy is very unfavorable for him, because he did not arrest him. The problem of these grade points in his main course, but to publish is to focus on publishing articles, which led to his articles being published, but the G-spot could not be reached.

01:49:06

But the new policy now is not to read your articles, but only to see your GPA, which is outrageous. Like the student you just said, did he retire? Not yet refunded. did not return. Because you are like us now, as long as you have passed CET 6, you don't need to take English, that's the class. Then we add that we have reproductive biology, then there are advanced scientific research experiments, then primary scientific research experiments, then there are gene x and then the frontier forum of reproductive biology, these are our own characteristic courses and core courses.

01:49:46

You said that I and my classmates of prevention did not study, and if I went to draw this line, I really learned these courses very well. In fact, it is not included in my memorial, which is why my counselor sent me a message last time saying that I think my GPA is not high. Like those who come in and leave, in fact, your characteristic courses, what they are called, and biology have all been abolished. That's what it means.

01:50:11

You haven't been careful.

01:50:12

I am, I remember he had a class last time, that is, when he was in a meeting, he said that he could be certified for that kind of course, but you said that after you have been certified for this course, there must be many more that you need to make up for yourself. , so we will now determine the process of a room in the State Affairs Office, which classes can be recognized, and which classes have specific plans, and there are some when they come out.

01:50:32

In fact, he was last time, but in fact, you said that you need to find these on the official website, but we have it last time. Do you have it or not?

01:50:43

So what is going on in your room? Our room is saying that it is important to leave the country and earn money. Otherwise, you will have to make up too many classes later, because our classes are all different. of. For example, in the third year of junior year, it is necessary to prevent them from taking their own professional courses. Our country also has its own professional courses, so don’t make up for it. After dropping out, it is equivalent to re-reading. Have you ever thought about dropping it?

01:51:06

What do I think? I will go back. I don't need to go back to the doctor. Prevention is prevention. How can I put it this way, I think the platform used by the country is still beneficial to me at present, so I don't want to quit, and if you say I don't want to quit now, I don't even want to quit in my junior year, because I want to quit in my junior year. If I quit, I would have to take the sophomore course. The pressure is still relatively high. You said that those who quit may be the sophomores, right? Your classmates and my classmates have not withdrawn yet. Several seniors and sisters in the junior year have withdrawn, that is, they should withdraw before entering the junior year. Yes there is. I would rather withdraw from the make-up class. True to his policy.

01:51:45

So actually, as I said just now, did you say that the postgraduate program will start this semester? Or last semester? The last semester that started last semester. In fact, what they talk about is your own feelings or how you feel about your classmates. In fact, you don't really agree with what he said. Just the section chief next door said that last time my senior sisters held him on the microphone. , he said that we will discuss it for a while and then tell you, did we really tell you later? Someone told him to invite Hu Xiao to come, and if he made a promise if he was invalid, he promised to go to Huahua County to reach 40%, and he would guarantee that you will guarantee the research, which is 40.

01:52:28

Just like what you just said, it is actually very opaque. What it means now is that it is very unreasonable to only count overlapping courses. Would you say that you personally feel that the policy of guaranteeing research is not clear? It will not make you or many of your classmates have the idea of quitting, which is actually a very main reason.

01:52:46

Yes, and as soon as his policy came out, he was looking at some of the middle school classes, intuitively taking those overlapping courses, and taking those overlapping courses to a higher level. It's not that we can't retake it now. For example, I have 4 courses this semester, and 4 courses on ABCD prevention. For bcdf, he will only take bcd. I get the point, and then his AA level is a core subject and they don't want to take it. In fact, I think it is very strange that you have to draw the 40% line with prevention. how to say? I don't know what the school's policy was at that time, because before I was admitted, his policy, I remember that as long as he didn't fail the course, he would give you a postgraduate study, and there were no student drawings that didn't exist in prevention or foundation. line of this statement.

01:53:46 It

's very strange. I think you are actually related to the school. The school's entire school-level policy for guaranteeing research is changing. There used to be a special channel for guaranteeing research. Now it seems to be tightened. For a bit more fair.

01:54:05

So it actually affects your research insurance policy. I think our insurance research policy is actually changed so that your average season must reach 3 or 3.5, which is more reasonable. It is your hard card. A line, you don’t want to go with other students, just students who are different from our majors, that is to say, drawing a line 4040% seems to be a bit fair, but in fact I think it is There is no logic.

01:54:34

Because you originally wanted to learn what I said now is to prevent the national weight, but in fact my course is the national weight, but the name of my undergraduate is on the prevention side.

01:54:46

You said that I am hanging here because of my undergraduate name, or even some unlucky middle school classmates, whose undergraduate name is hanging on the foundation, it will be very miserable. In fact, if they are hanging on the foundation, Because the basic rights are too convoluted, there are many classes that are really convoluted, and they are convoluted.

01:55:05 There

are more basic activities than your classes, right? Because the distribution is actually unreasonable now, because the courses we really want to learn are the same, because they are all national weights, but the courses they want to learn are not very easy to arrange, so they put a lot of difficult courses. Arrange for them in advance, and then, for example, they will study this semester. In fact, you may also study this class. You will face our courses in the later stage. They are relatively loose, mainly for scientific research time. You said that they are all from middle school classes, why is there such a big difference?

01:55:35

right?

01:55:37

In fact, it's the same as what I just said. It's not very reasonable for your classmates who are serious about prevention to pull the strings with classmates who are prevention. Because in fact, there will be no competition in the first place. It is very strange, and our middle school students do not participate in any rankings, we only watch the line, so sometimes it is not difficult to understand why he does not discuss things like you with classmates outside the middle school class, you guys The inexplicable insurance research policy is not because it will not resonate.

01:56:13

Because what I said, for example, I told my roommate that he didn't understand, and he also felt that it had nothing to do with him. Or is it a professional, what else do you have? Not very good to talk about other.

01:56:42

We shouldn't have anything too much. I think I've already asked what I want, alright, and it's still personal, you just said those things that are black and white, actually I think you said That part of people may have touched the darker side of society, and they may have returned to thinking that they want to return to a simpler way of thinking. I think these people may be a minority, and more people may be incapable of Thinking about so many things, it is possible to say that the family influence you are talking about is to think that he has no way, or from his personal values, he has no way to accept the existence of this kind of thing, or It is said that for an ordinary individual, the black-and-white thinking mode is the simplest, the most labor-saving and the most relaxing. I think most people should be like this. Personally, it is his thinking mode that makes him He lived an easy and simple life, he didn't have to think about so many things, he just lived in this mode.
