# Supplementary material for: Why do you choose this program?—A decision-making model of medical students based on grounded theory
Source: PLoS One. 2023 Sep 15;18(9):e0291634. doi: 10.1371/journal.pone.0291634 (PMC10503722; doi:10.1371/journal.pone.0291634)
Supplement: Attachment — Submitted filename: Response to Reviewers.docx [file pone.0291634.s003.docx]

**Journal requirements:**

1. We have formatted the manuscript according to the requirements listed in the <TITLE, AUTHOR, AFFILIATIONS FORMATTING GUIDELINES> and <MANUSCRIPT BODY FORMATTING GUIDELINES>.
2. We have deleted the ethics statement at the end of the manuscript and moved it to the Methods section.
3. We have included the caption for the supporting information at the end of the manuscript.
4. The supplementary Figures 1-7 were initially submitted to prove that the manuscript had been edited for language usage and had nothing to do with the content of the manuscript. Therefore, we chose to remove the figures from submission.
5. We reviewed the reference. It is complete and correct without citing any retracted article.

**Reviewer #1:**

1. We have switched to past tense for the Methods section.
2. First, we chose the way of team work to control bias in this research which has been added to the methods section. Second, we have updated the reference in the methods section according to the reviewer’s suggestion.

**Reviewer #2:**

1. According to the COREQ-checklist. We have added the items to the manuscript. The COREQ-Checklist has been added as supporting information.
2. NKL was launched in 2018. It has been added in the manuscript.
3. Table 1 has been moved to the result section.
4. We have moved some results from discussion to results and have added some content to the results section to avoid that some results are reported only in the discussion section.
5. We have added the limitation at the end of discussion section.
